# Functionalizing Thiosemicarbazones for Covalent Conjugation

**DOI:** 10.3390/molecules29153680

**Published:** 2024-08-03

**Authors:** Johannes Hohnsen, Lukas Rryci, Diana Obretenova, Joshua Friedel, Shahab Jouchaghani, Axel Klein

**Affiliations:** University of Cologne, Faculty of Mathematics and Natural Sciences, Department of Chemistry and Biochemistry, Institute for Inorganic and Materials Chemistry, Greinstraße 6, 50939 Koeln, Germany; jhohnsen@smail.uni-koeln.de (J.H.); lrryci@smail.uni-koeln.de (L.R.); diana.obretenova@mail.com (D.O.); jfried20@uni-koeln.de (J.F.); sjouchag@smail.uni-koeln.de (S.J.)

**Keywords:** thiosemicarbazones, functionalization, amino acids, glucose, phosphonate, *o*-hydroquinone

## Abstract

Thiosemicarbazones (TSCs) with their modular character (thiosemicarbazides + carbonyl compound) allow broad variation of up to four substituents on the main R^1^R^2^C=N(1)–NH–C(S)–N(4)R^3^R^4^ core and are thus interesting tools for the formation of conjugates or the functionalization of nanoparticles (NPs). In this work, di-2-pyridyl ketone was introduced for the coordination of metals and 9-anthraldehyde for luminescence as R^1^ and R^2^ to TSCs. R^3^ and R^4^ substituents were varied for the formation of conjugates. Amino acids were introduced at the *N*4 position to produce [R^1^R^2^TSC–spacer–amino acid] conjugates. Further, functions such as phosphonic acid (R–P(O)(OH)_2_), *D*-glucose, *o*-hydroquinone, OH, and thiol (SH) were introduced at the *N*4 position producing [R^1^R^2^TSC–spacer–anchor group] conjugates for direct NP anchoring. Phenyl, cyclohexyl, benzyl, ethyl and methyl were used as spacer units. Both phenyl phosphonic acid TSC derivatives were bound on TiO_2_ NPs as a first example of direct NP anchoring. [R^1^R^2^TSC–spacer–end group] conjugates including OH, S–Bn (Bn = benzyl), NH–Boc (Boc = *tert*-butyloxycarbonyl), COO*t*Bu, C≡CH, or N_3_ end groups were synthesized for potential covalent binding to functional molecules or functionalized NPs through amide, ester, or triazole functions. The synthesis of the thiosemicarbazides H_2_NNH–C(S)–NR_3_R_4_ starting from amines, including amino acids, SCCl_2_ or CS_2_, and hydrazine and their condensation with dipyridyl ketone and anthraldehyde led to 34 new TSC derivatives. They were synthesized in up to six steps with overall yields ranging from 10 to 85% and were characterized by a combination of nuclear magnetic resonance spectroscopy and mass spectrometry. UV-vis absorption and photoluminescence spectroscopy allowed us to easily trace the dipyridyl imine and anthracene chromophores.

## 1. Introduction

Thiosemicarbazones (TSC) and their metal complexes have continuously received attention from their interesting biological properties and anti-bacterial, anti-malarial, anti-parasitic, and anti-proliferative activities have been reported [1,2,3,4,5,6,7,8,9,10,11,12]. This includes TSCs covalently bound/conjugated to other biologically relevant molecules such as glucose, amino acids or peptides, or coumarin to enforce their biological activities [13,14,15,16,17,18,19,20,21]. Also, the binding of metals to the TSC moieties of molecular TSC-conjugates to generate anti-proliferative properties has previously been studied [15,16,17,18,19,20,21,22].

**Scheme 1:**
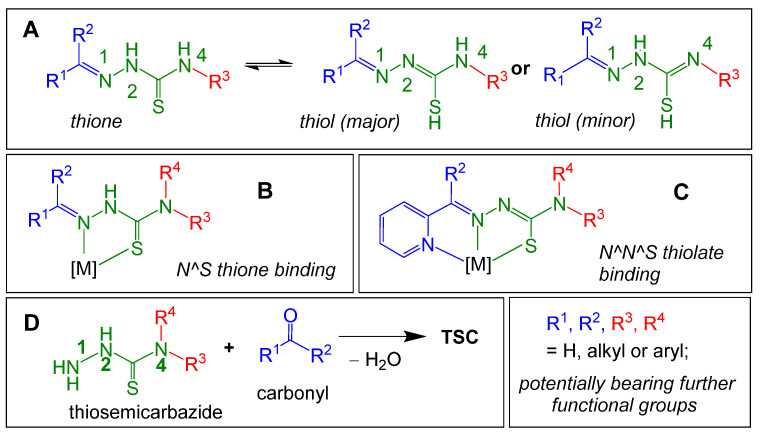
(**A**) Schematic representation of thiosemicarbazones (TSC) in their thione and thiol forms (R, R′, R″ = H, alkyl, or aryl) with numbering of the TSC backbone (in green). (**B**) N^S thione coordination of a TSC to metal fragments [M]. (**C**) 2-acetylpyridine-thiosemicarbazones (R^2^ = CH_3_) showing tridentate N^N^S thiolate binding. (**D**) Formation of a TSC from a thiosemicarbazone and a carbonyl.

In contrast to this, the idea of binding metal-TSC complexes or [drug–TSC] conjugates to surfaces of nanoparticles (NPs) is new [23,24,25]. The potential suitability of TSCs to serve as ligands for metal coordination covalently anchored on NP surfaces and conjugating them to further functional units at the same time, is obvious from the up to four possible substituents on both the *N*1 and *N*4 functions of the general TSC backbone R^1^R^2^C=N(1)–NH–C(S)–N(4)R^3^R^4^ (Scheme 1A,B, marked in green) and from the modular synthesis from the R^3^- and R^4^-substituted thiosemicarbazides and any R^1^- and R^2^-substituted carbonyl (Scheme 1D, blue and red marking, respectively). This allows a very broad variation in TSCs [1,2,11,12,17,20,26,27].

TSCs have several donor atoms in their NNC(S)N backbone that allow metal coordination (Scheme 1), with the five-ring chelate N1^S coordination being the most prominent (Scheme 1B) [7,8,9,10]. This coordination sphere can be extended using further coordination units such as pyridyl (Scheme 1C). The TSC immanent thione–thiole tautomerism (Scheme 1A) contributes to selective metal ion binding in that the TSC ligand adopts either the neutral thione or the anionic thiolate forms (after deprotonation). The latter can compensate the typical cationic charge of metal centers. Equipped with these metal-binding abilities, TSCs have been broadly used as ligands for the formation of metal complexes [1,2,4,5,6,7,8,9,15,17,18,19,20,21] or for the functionalization of surfaces, including nanoparticles (NPs) [23,24,25,28,29,30,31,32,33].

**Scheme 2:**
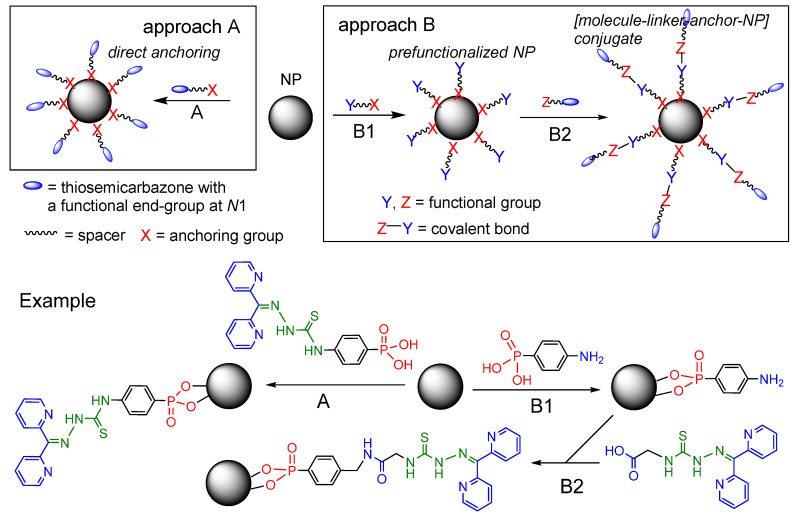
Schematic approaches to [TSC–X anchor] (approach A) and [TSC–Z–Y–X anchor] (approach B) conjugates for covalent anchoring of nanoparticles (NPs), with an example from this study. Shown is the [dipy–TSC–Ph–phos–TiO_2_] conjugate (dipy = di-2-pyridyl).

Quite generally, for the functionalization of NPs, covalent binding of surface ligands, metal complexes, drug molecules, luminescent probes or other surface functionalities is clearly superior to adsorption through non-covalent forces such as hydrogen bonding, dipolar forces, or van der Waals forces. Covalent bonding allows far more stable and selective binding of the surface functionalities with defined chemical moieties. Also, it allows for controlled release through specific reactions reversing the covalent binding [34,35,36,37,38]. This also includes coordinative bonds [39,40]. The functional molecules defining the application can be either directly anchored on the NP surface through functions like carboxylates, phosphonates, or alkylsilanes (=[molecule–anchor] conjugates) (Scheme 2, approach A) or by using [molecule–end group] conjugates combining the functional molecule and a reactive end group for covalent binding to pre-functionalized NP surfaces (Scheme 2, approach B). For both cases, multifunctional molecules allowing broad variation and good chemical stability are required [40,41,42].

TSCs have previously been used for the functionalization of NP using various non-covalent approaches, including nano formulation/encapsulation [9,10,28,29,30,31,32,33]. In contrast to this, approaches for covalent binding of TSC onto NP are scarce. Till recently, only the condensation of citrate-functionalized Au NP with *p*-methyl-carbohydrazonethioamide [23] was reported, while the binding of a phenolate–acridine–TSC molecule on an Ag NP surface forming a [phenolate–acridine–TSC–Ag] conjugate, is depending on the coordination of this molecule on the surface and not on covalent bonds [24]. In a very recent approach, Fe_3_O_4_@SiO_2_ core–shell NPs were reacted subsequently with (3-chloropropyl)trimethoxysilane (CPTMS), 4-hydroxyacetophenone, thiosemicarbazide, and CuSO_4_^.^5H_2_O to yield a fully covalently linked [NP–TSC–Cu] conjugate with the Cu(I) ions bound by two differently anchored 4-oxoacetophenone–TSC moieties [25]. In further recent reports, the surface of NPs was functionalized using materials such as dextran or chitosan before attaching TSCs or TSC complexes to them, but the character of the bonding between the TSCs and NP surface is not clear [42,43,44,45,46,47].

We embarked on a study exploring the possibility of using TSC molecules as connecting units for conjugation. The aim was to provide a toolbox for the derivatization of TSCs enabling them to form covalent connections between biologically relevant molecular entities, metal complexes, fluorescent tags or NPs in tri- or multilateral conjugates. Herein, we report on our first attempts to synthesize functionalized TSC derivatives for direct covalent/coordinative anchoring of TSC molecules on NP surfaces ([TSC–X–NP] conjugates), Scheme 2, approach A) or TSC derivatives (TSC–Z), which can be bound to NP surfaces using additional anchoring units (Y–X) for covalent connection in [TSC–Z–Y–X–NP] conjugates (Scheme 2, approach B).

**Scheme 3:**
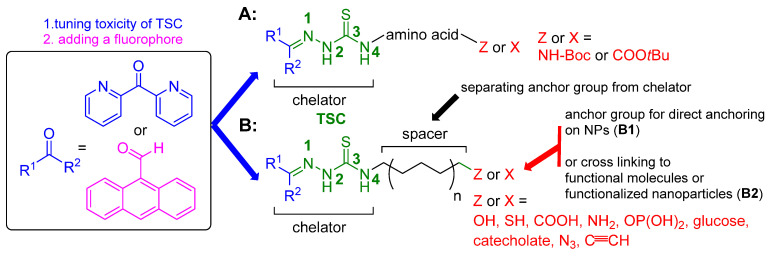
Targeted functionalization of TSC in this work. (**A**) [R^1^R^2^TSC–spacer–amino acid] conjugates (**B**) [R^1^R^2^TSC–spacer–anchor] (**B1**) and [R^1^R^2^TSC–spacer–end group] conjugates (**B2**).

The TSCs in this study were functionalized at the *N*1 position with di-2-pyridyl ketone leading to [dipy–TSC–Z/X] conjugates (Scheme 3), as the resulting di-2-pyridyl-imine group usually leads to an increased toxicity of TSCs [2,48,49,50,51,52]. Alternatively, 9-anthraldehyde (anthracene-9-carbaldehyde) was placed in this position to form [Anthr–TSC–Z/X] conjugates (Scheme 3) [22,27,53,54,55].

In the *N*4 position, various chemical functionalities were added. They were either protected amino acids with their NH–Boc protected functions terminating the conjugate, or hydrocarbon spacers to functional groups such as phosphonic acid (R–P(O)(OH)_2_), α-*D*-glucose, *o*-hydroquinone, thiol (SH), or OH for anchoring on NPs or OH, NH_2_, COOH, N_3_, or C≡CH (Scheme 3) for potential covalent conjugation via simple reactions such as amide or ester formation or click chemistry.

With the molecular TSC conjugates shown in Scheme 3 in hand, trilateral [functional-molecule–metal complex–TSC–NP] conjugates can be generated in future work. On the other hand, this approach also will allow the generation of conjugates with other functional molecules [functional-molecule(1)–TSC–complex–functional-molecule(2)] or other combinations of functional moieties. In this contribution, the successful synthesis of such molecular [TSC–spacer–anchor] and [TSC–spacer–end-group] conjugates is reported alongside a first example for anchoring a [TSC–spacer–anchor] conjugate on NPs.

## 2. Results and Discussion

### 2.1. General Approaches and Targets

**Scheme 4:**
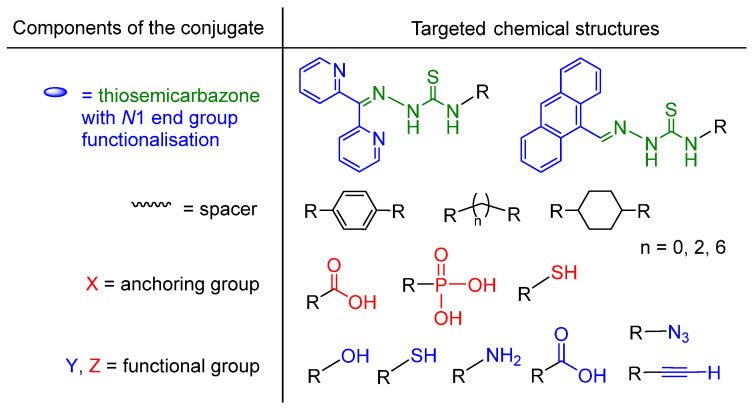
Components of the [TSC–X anchor] and [TSC–Z–Y–X anchor] conjugates in this work.

In the focus of the first group (approach A in Scheme 2) of target structures TSC–X, were TSCs containing potentially coordinating amine, carboxylate, hydroxy, phosphonate, and thiol groups (Scheme 4, anchoring group). TSCs with a thiol group can be used for direct attachment to gold or other NP with a soft character in view of the hard and soft acids and bases (HSAB) principle [56]. Hard NP surfaces such as metal oxides can be coordinated using amine, carboxylate, hydroxy, phosphonate, or silane groups.

Amine, carboxylate, and hydroxy groups together with alkyne and azide functionalities can also be used to covalently link the TSCs to pre-functionalized nanoparticles by “click” chemistry or the formation of esters and amides (TSC–Z + Y–X–NP) (approach B in Scheme 2, for details, see Section 2.2).

**Scheme 5:**
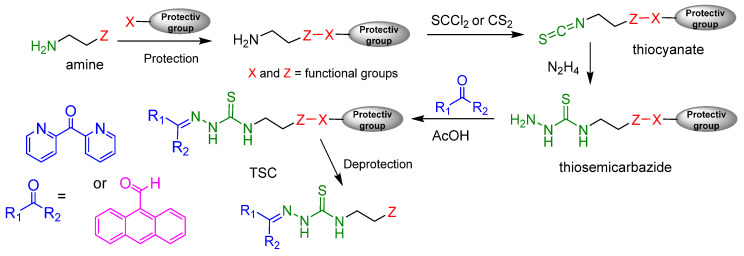
General procedure for the synthesis of TSCs in this work.

The synthesis of the *N*4 functionalized TSCs started from bifunctional, primary amines with the general synthesis shown in Scheme 5 (details in Section 2.2 and the Supplementary Materials). The reaction of the amines with thiophosgene (SCCl_2_) or carbon disulfide (CS_2_) led to thiocyanates. Reaction with hydrazine (N_2_H_4_) gave the thiosemicarbazides. They were reacted with the two different carbonyl compounds, di-2-pyridyl ketone and 9-anthraldehyde to give the TSCs. As SCCl_2_, CS_2_ or N_2_H_2_ are very reactive reagents, functional groups that should not be involved in the reactions had to be protected (Scheme 5). After condensation, the TSCs were thus obtained initially in their protected form. In some cases, they were subsequently deprotected, and in other cases, the protected TSCs are the final products.

In some cases, anchor groups were introduced to spatially separate the anchoring group from the TSC (Scheme 3). At the same time, the nature of this spacer function might also affect the biological properties of the TSC and the rigid 1,4-phenylene, the slightly more flexible 1,4-cyclohexane, and the fully flexible alkyl spacers 1,2-ethylene or 1,6-hexylene (Scheme 4) were chosen.

**Scheme 6:**
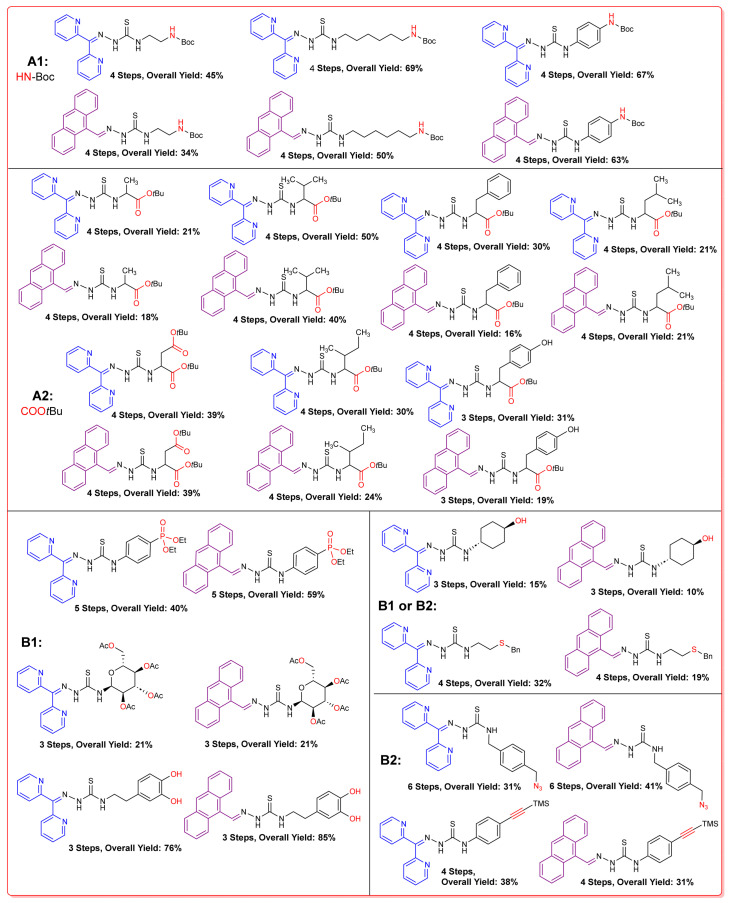
Summary of functionalized TSCs reported in this work (the labels **A**, **B1,** and **B2** refer to Scheme 3).

In total, 34 TSC derivatives with di-pyridyl ketone (Scheme 6, marked in blue) or 9-anthraldehyde (Scheme 6, marked in purple) in the *N*1 position and various functional groups (marked in red) and spacers in the *N*4 position (for details, see Section 2.2) were synthesized.

Details of the syntheses of the thiosemicarbazides and the thiosemicarbazones can be found in the Supplementary Materials—Part I. The reported products are generally air- and hydrolysis-stable. The anthracenyl derivatives seem to be light-sensitive and should be stored under the exclusion of light. All products are soluble in MeCN and dimethyl sulfoxide (DMSO) and were characterized using ^1^H and ^13^C nuclear magnetic resonance (NMR) spectroscopy and high-resolution electron-spray-ionization mass spectrometry (HR-ESI-MS) (data and NMR spectra in the Supplementary Materials—Part I. UV-vis absorption spectra for all TSC derivatives and additionally photoluminescence spectra for the anthracenyl derivatives were recorded (spectra in the Supplementary Materials—Part II). The UV-vis absorption spectra (Figures S313 and S314, Supplementary Materials—Part II) are typical for the two chromophoric groups di-2-pyridyl imine and anthracenyl. The photoluminescence spectra of the anthracenyl–TSC conjugates show broad bands peaking around 500 nm, typical for aggregation of anthracenyl moieties [57]. At dilutions into the lower nmol range, the typical resolved monomeric anthracene fluorescence is observed (Figures S315–S318, Supplementary Materials—Part II).

### 2.2. Syntheses of TSCs

#### 2.2.1. Thiosemicarbazones Derived from Amino Acids

**Figure 1:**
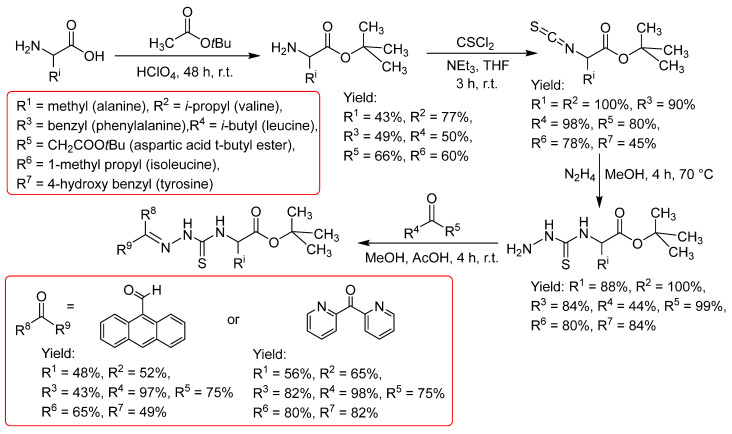
Synthesis of TSCs derived from amino acids.

The purpose of introducing amino acids at the TSC-*N*4 group (Figure 1) is i.a. motivated by our wish to use such [TSC–amino acid] conjugates for the attachment of peptides for further functionalization [40]. We have recently reported *tert*-butyloxycarbonyl (Boc)-protected *L*-lysine–TSC conjugates, [TSC–lysine–NH–Boc] for the addition of the cell-penetrating peptide sc18 and thus produced [Ptcomplex–TSC–lysine–sc18] conjugates [19]. In the frame of the same work, we found that methyl ester protection is not sufficient [58].

The Boc protection of the carboxylic acid function (Figure 1, first step), following a previously reported approach [59], is limited by lacking solubility of amino acids in *tert*-butyl acetate which is used as a solvent. This might explain in part the poor yields (43 to 77%). The yields of the following reaction with thiophosgene varied massively with the lowest values for R = 4-hydroxy benzyl. Maybe the additional hydroxymethyl function is not well tolerated. The yields for the thiosemicarbazide formation reaction (Figure 1, bottom right) are generally excellent, while the final condensation with the carbonyls requires optimization as some yields are only around 50% or even below. Overall, the final products, including both the anthraldehyde and the dipyridylketone, were obtained as Boc-protected derivatives in a four-step procedure in reasonable to good overall yields ranging from 18 to 50%.

From optical rotation experiments, we obtained specific optical rotation values [α] at 20 °C in CHCl_3_, ranging from +5° to +35 for most amino acid derivatives depending on the wavelengths (λ) and amino acid–NHBoc residue (Table S1 in the Supplementary Materials—Part I). Only for the [dipy–TSC–aspartic acid–NHBoc] conjugate, we found negative values of around –5. Since the NH_2_ function of the starting amino acid is functionalized and not the C-α atom, we assumed thus, that the stereochemistry at C-α is retained in all reaction sequences. At least we can rule out 50:50 racemates as products.

The removal of the Boc group (deprotection) of a similar TSC–*L*-lysine derivative was recently demonstrated [19] and should also not be problematic for the other amino acids.

#### 2.2.2. Thiosemicarbazones Derived from Diamines

**Figure 2:**
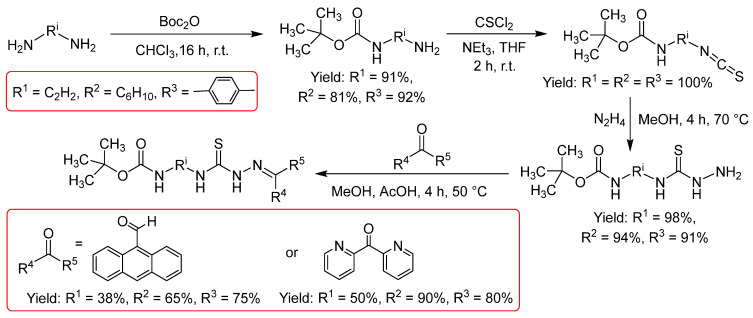
Synthesis of TSCs starting with diamines introducing hydrocarbon spacers.

The same motivation, to finally conjugate peptides to the TSCs, gave the idea to start from diamines (Figure 2). The four-step reaction sequence gave NHBoc-protected TSCs overall yields ranging from 50 to 68%. The first step, the Boc protection of one amine function, modifying established methods for ethylene diamine [60], 1,4-diaminohexane [61], and 1,4-phenylenediamine [62], was very successful with excellent yields ranging from 81 to 92%. In the next step, the thiocyanates were formed quantitatively. For the thiosemicarbazide formation from the thiocyanates, excellent yields were obtained, while some condensations require optimization as yields were as low as 38 or 50%.

The de-protection of the [TSC–phenylene–NHBoc] conjugate was achieved with a 93% yield using HCl in MeOH solution (Section S1.2.2.4 and Figure S172 in the Supplementary Materials—Part I).

#### 2.2.3. Thiosemicarbazones Derived from Acetobromo-α-*D*-Glucose

Glucose has been reported as an interesting functionality to selectively address cancer cells [51,53,56,63,64,65]. Therefore, it is not surprising, that TSCs with glucose moiety at the *N*4 position and their biological activities have previously been reported [11,66,67,68,69]. However, the *N*1 position was not functionalized with dipyridyl ketone and 9-anthraldehyde yet. Also, the possibility of using glucose-tagged TSC in polysaccharide nanoconjugates has not been studied yet. Finally, the glucose moiety might also anchor efficiently on metal oxide NP surfaces.

**Figure 3:**
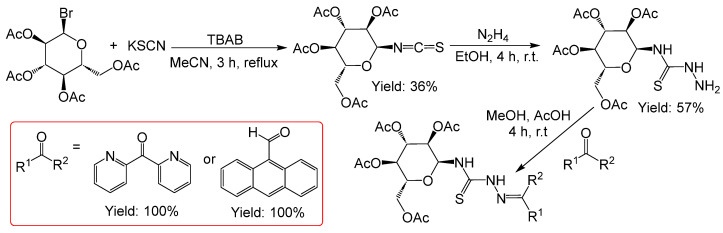
Synthesis of TSCs from acetobromo-α-*D*-glucose. TBAB = tetra-*n*-butylammonium bromide.

The synthesis was started from acetobromo-α-*D*-glucose and received the two target conjugates in a three-step process with overall yields of 20% (Figure 3). Both, the formation of the thiocyanate and the reaction with hydrazine require optimization.

The synthesis of the thiosemicarbazide had a 95% yield in a similar manner, which has previously been reported for the preparation of [*D*-glucose–TSC–isatin] conjugates [14]. For the same reaction using CH_2_Cl_2_ as solvent at ambient temperature, a 93% yield is reported [68]. The subsequent reactions with various aromatic aldehyde substrates O=CH(aryl) in 2-propanol without acid or base catalyst, gave yields ranging from 84 to 90% [68]. Our method using glacial acetic acid as a catalyst for condensation is tolerated by the protecting groups and gave quantitative yields.

Specific optical rotation values [α] in CHCl_3_ at 20° C ranging from 75 to 96, depending on the wavelength (λ), were found (Table S1). This stands in contrast to a report on [aryl–TSC–Glucose–OAc] conjugates for which [α]_D_ values in CHCl_3_ at 20° C ranging from −103 to −48 at concentrations *c*~1 were reported [69]. However, due to the strong absorption of the dipyridyl and the anthracenyl group we were not able to measure at the Na-*D*-line (as conducted in the report) but had to move to far higher wavelengths (546, 579, and 589 nm). Even in this small λ range, our [α] values varied marked. For the same reason, we had to measure at *c* ~ 0.1. Both factors (λ and *c*) can probably explain the discrepancy.

In the above-mentioned work, the de-protection of the acetyl-glucopyranosyl groups using NaOMe in MeOH was reported with yields ranging from 33 to 92% [69]. The [TSC–glucopyranosyl–aryl] conjugates show positive [α]_D_ values ranging from 41 to 68 in MeOH [69].

#### 2.2.4. Thiosemicarbazones with a Hydroxy-Alkyl Function

**Figure 4:**
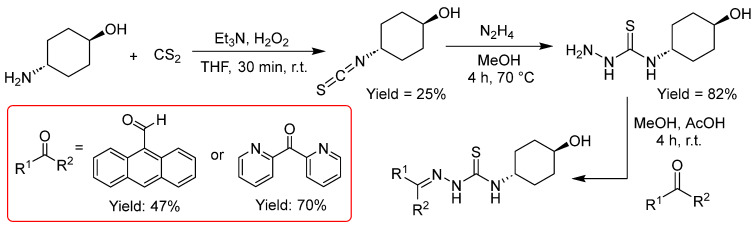
Synthesis of TSCs starting from *trans*-4-hydroxy-cyclohexanol.

Hydroxy functions are very versatile and the target HO–C_n_H_m_ spacer moiety could potentially be used for the formation of esters for further conjugation or for direct anchoring to NPs. In a three-step reaction procedure, starting from *trans*-4-amino-cyclohexanol we obtained the *N*4-4-hydroxy-cyclohexanol TSCs in overall yields of 10 and 14% (Figure 4).

The idea to use the less reactive CS_2_ compared with the highly reactive thiophosgene was derived from a report converting hydroxyalkyl amines into hydroxyalkyl isothiocyanates [70]. For the 4-hydroxy-cyclohexanol, an optimized yield of 86% was reported [70]. As the cyclohexanol end group is very interesting for further conjugation, e.g., for ester formation, especially this first reaction calls for optimization in future work.

#### 2.2.5. Thiosemicarbazones with a *o*-Hydroquinone Function

**Figure 5:**
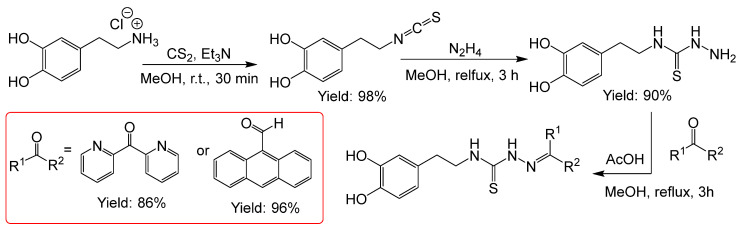
Synthesis of TSCs starting from dopamine.

Benzene-1,2-diol (*o*-hydroquinone) represents a very suitable anchoring function for iron oxides in the form of catecholate [71,72]. This moiety was introduced starting from 4-(1,2-benzene-1,2-diol)ethylammonium chloride (dopamine hydrochloride) and the two TSCs were received in very good overall yields of 76 and 85% in three steps (Figure 5). The 1,2-diol functionality is obviously fully tolerated.

This confirms a report on the synthesis of the thiocyanate, starting from the free base (1,2-benzene-1,2-diol)ethylamine under NEt_3_ catalysis in THF with a 96% yield [70]. The following reaction steps were unprecedented.

#### 2.2.6. Thiosemicarbazones Derived from Amino Thiols

**Figure 6:**
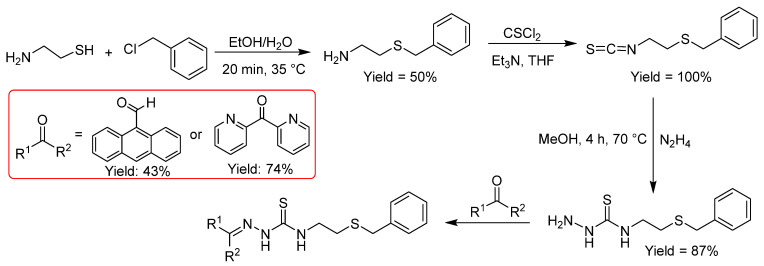
Synthesis of TSCs derived from amino thiols.

A benzyl-protected thiol was the target of the reaction starting from 2-amino-1-thiol and benzyl chloride (Figure 6). The first reaction step was derived from a report on the synthesis of β-benzylmercaptoethylamine derivatives [73]. The reported optimized yield is 90% when starting from the HS–CH_2_–CH_2_–NH_2_^.^HCl, using LiOH as a base and H_2_O/EtOH as a solvent mixture. We obtained a lower yield of 50% but started from the pristine 1,2-aminoethylthiol preventing the use of base which facilitates the procedure. Nevertheless, as the low yield for this reaction, together with that of the condensation decreases the overall yields massively to 32% and 19%, respectively, both reactions require optimization. In contrast to this, the formation of the thiocyanate using thiophosgene was quantitative.

Prior to anchoring these TSCs on thiophilic metal NP such as Au or Ag [74] via the thiolate (sulfide) function, the benzyl protecting group must be removed. Basically, this requires the use of strong acids or bases or must be conducted under reductive conditions using alkali metals or stannyl hydrides [75]. As all these methods are threading the TSC function, we are still trying to find a way to safely de-protect these TSCs. So far, we were not successful.

#### 2.2.7. Thiosemicarbazones with an Alkyne Function

Terminal alkynes are versatile groups for azide-alkyne click reactions (AAC), which have been frequently used for bioconjugation [76,77,78].

**Figure 7:**
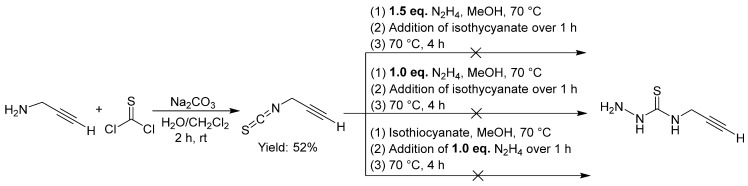
Attempted synthesis of thiosemicarbazones with an alkyne function starting from propargyl amine.

**Scheme 7:**
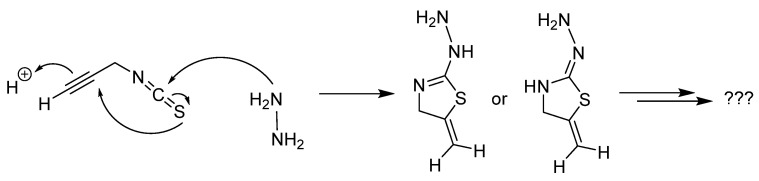
Proposed cyclization reaction preventing the formation of propargyl amine thiosemicarbazide. ??? denotes the formation of mixtures of unidentifiable reaction products.

Unfortunately, the synthesis of thiosemicarbazides derived from propargyl amine (Figure 7) was not successful. While the reaction of propargyl amine with thiophosgene gave the thiocyanate a 52% yield, the following reaction of the isothiocyanate with hydrazine hydrate failed to give the target thiosemicarbazide. Our failure is in line with a previous report using hydroxy-isothiocyanates [70] and we assume, that a rapid cyclization reaction occurs, preventing the product formation (Scheme 7) [79,80]. While thiazole cores, like those shown in Scheme 7, were plausible intermediates [80], we failed to detect them by NMR or MS. Instead, we observed unidentifiable mixtures of species.

**Figure 8:**
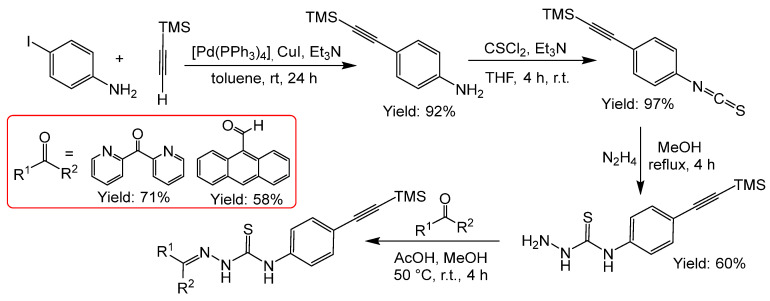
Synthesis of synthesis of TSCs with alkyne functions on rigid phenylene spacers.

Consequently, a more rigid spacer function was introduced between the thiosemicarbazide and alkyne function (Figure 8) and the two *N*4-alkyne-TMS functionalized TSCs were obtained in four steps in good to reasonable yields.

The first step was derived from a study in which the TMS–C≡C–Ph–NH_2_ molecule (synthesis with a 99% yield) is used to conjugate single-walled carbon nanotubes with polystyrene–N_3_ via a CuAAC reaction [81].

#### 2.2.8. Thiosemicarbazones with an Azide Function

**Figure 9:**
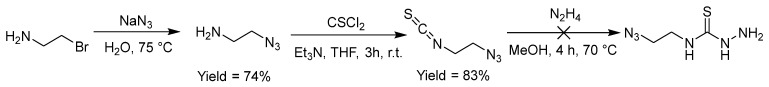
Attempted synthesis of thiosemicarbazones with an azide function.

Azide-substituted TSC derivatives are excellent candidates for covalent conjugation using the azide-alkyne click (AAC) reaction [76,77,78,81,82]. Thus, 2-amino-1-bromoethane was reacted with NaN_3_ to yield 2-azidoethylamine at a 74% yield (Figure 9). The conversion to the thiocyanate using thiophosgene was also successful (83% yield). When reacting the thiocyanate with hydrazine under our established conditions, the azido derivative was not obtained. Very probably, the flexible spacer function between the azide and isothiocyanate function probably led to cyclization reactions, confirming our assumptions for the attempts for alkyne substituted TSCs with flexible spacers (Figure 7).

**Figure 10:**
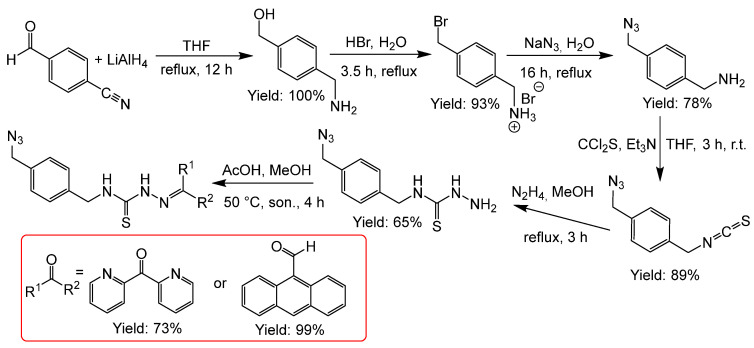
Synthesis of TSCs with azide functions using the rigid dimethyl-phenylene spacer.

When introducing the rigid phenylene spacer function between the thiosemicarbazide and azide function, the two azide functionalized TSCs were obtained in a six-step reaction sequence in remarkable 36 and 42% overall yields, starting from 4-cyano-benzaldehyde (Figure 10).

The first three steps are adopted from previous reports. The conversion of the 4-cyano benzaldehyde [83], the bromination of the hydroxymethyl group [84], and the formation of the azide [85] have been carried out in a similar manner with very similar yields.

#### 2.2.9. Thiosemicarbazones with a Phosphonate Group

**Figure 11:**
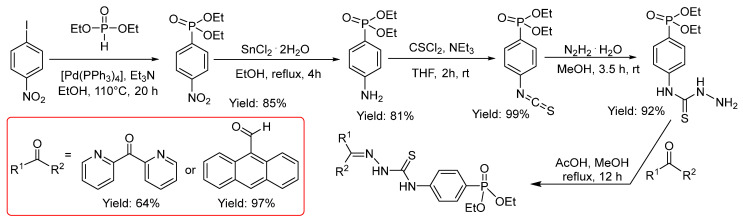
Synthesis of TSCs with a phosphonate group.

Phosphonates are suitable groups for anchoring on iron oxide [86,87,88,89] or TiO_2_ NPs [90,91,92,93]. Phosphonylation of 4-iodo-nitrobenzene allowed the introduction of a diethylphosphonato–phenyl unit as an end-group at the *N*4 terminus of the two TSCs (Figure 11) in a five-step reaction sequence with 61% and 40% overall yield, respectively.

The first step is adopted from two different reports. The first paper reports the synthesis of the 4-nitrophenyl-phosphonic ester with a 14% yield starting from the bromo derivative [94]. Our massively increased yield of 85% is probably due to the markedly superior leaving group character of iodide compared with bromide. The second report includes both our first and second steps [95]. With slightly different reaction conditions, the authors report yields of 37 and 54% for the 4-nitro- and the 4-aminophenyl-phosphonic ester, respectively which lie far below our yields. The formation of the thiosemicarbazide is very temperature-sensitive. At elevated temperatures, we found reactions of the hydrazine with the phosphonic ester groups leading to un-identifiable mixtures of species.

### 2.3. Direct Anchoring of Phenyl Phosphonium Acid TSCs on TiO_2_

**Figure 12:**
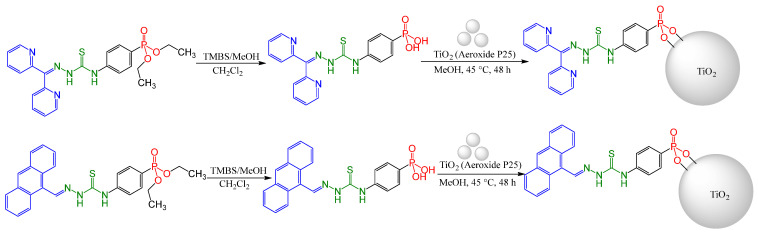
Anchoring of TSC-phosphonates on TiO_2_ forming the conjugates [Dipy–TSC–Ph–phos–TiO_2_] (**top**) and [Anthr–TSC–Ph–phos–TiO_2_] (**bottom**). TMBS = trimethylbromosilane.

As a first example of direct anchoring of a [TSC–spacer–anchor] conjugate to NPs, the phenyl phosphonic acid derivatives from 2.2.9 were chosen to be anchored on TiO_2_ (anatase) NPs (Figure 12).

In the first step, the TSC-phosphonic acid ethyl esters were deprotected using trimethylbromosilane (TMBS) in MeOH and extraction of the phosphonic acids with CH_2_Cl_2_ (Figure 12). The acids were characterized through their ^1^H NMR spectra (Figures S305–S307, Supplementary Materials—Part I). The free acids were anchored on TiO_2_ (anatase, Aeroxide P25^®^) and the NPs were carefully washed with MeOH resulting in yellowish materials (photographs in Figure S308, Supplementary Materials—Part II).

The FT-IR spectra of the two TSC-phosphonic acid conjugates, [TSC–Ph–phos–OH] (Figure S309) show marked bands in the range 1500 to 700 cm^−1^ for the TSC backbone and resonances at around 3000 cm^−1^ representing the NH and CH functions of the TSC and the OH function of the phosphonic acid group [91,92]. While the bands typical for the TSC are still found in the [TSC–Ph–phos–TiO_2_] conjugates, the OH resonances are missing, in line with the anchoring = condensation of the P–OH groups with TiO_2_ surface functions to P–O–Ti moieties. Further marked changes are found in the P–O region (1300 to 800 cm^−1^)[91,92,93] with a marked resonance at 1045 cm^−1^ (Figure S309) representing the P–O–Ti [93] as unequivocal proof for the covalent anchoring. UV-vis absorption spectra of the [TSC–Ph–phos–TiO_2_] conjugates in MeOH are superpositions of the individual spectra of TiO_2_ and the [TSC–Ph–phos–OH] conjugates (Figure S310). Photoluminescence spectra of the anthracenyl-substituted [Anthr–TSC–Ph–phos–TiO_2_] conjugate (Figure S311) show the typical broad emission of an anthracene aggregate.

Dynamic light scattering (DLS) and Zeta potential measurements support strongly the formation of the NP conjugates ([TSC–Ph–phos–TiO_2_] conjugate). Polydispersity, Z-Average and Zeta potential of the two NP conjugates are markedly different compared with the TiO_2_ starting material (Table 1).

We conclude from this that the [TSC–Ph–phos–OH] conjugates vary the surface functionalization of the original TiO_2_ material, preventing agglomeration and changing the surface potential.

## 3. Materials and Methods

### 3.1. Materials and Synthesis

The commercially available materials and all details on the synthesis of the compounds are provided in the Supplementary Materials—Part I.

### 3.2. Instrumentation

^1^H, ^13^C, and correlation spectra were recorded on a Bruker Avance II 300 MHz (1H: 300 MHz, 13C: 75 MHz) (Bruker, Rheinhausen, Germany), equipped with a double resonance (BBFO) 5 mm observe probe head with a z-gradient coil or on a Bruker Avance III 499 (^1^H: 499 MHz, ^13^C: 125 MHz) using a TCI Prodigy 5 mm probe head with z-gradient coil. Chemical shifts were reported relative to TMS (^1^H, ^13^C). The spectral assignments for ^1^H and ^13^C are based on ^1^H,^1^H COSY, ^1^H,^13^C HMQC/HSQC, ^1^H,^13^C HMBC, ^13^C-DEPTQ methods. HR-ESI-MS spectra in the positive mode were measured using a Thermo scientific LTW Orbitrap XL mass spectrometer at 70 eV (ThermoFisher Scientific, Waltham, MA, USA). UV-vis absorption spectra were recorded on a Varian Cary 60 spectrophotometer (Varian Medical Systems, Darmstadt, Germany). UV-vis photoluminescence spectra were obtained using an FLS100 spectrometer with a 450 W xenon light source (Edinburgh Instrument, Livingston, Scotland, UK). The spectrometer was equipped with a PMT-900 detector and a double-grating Czerny-Turner monochromator. All measurements were performed at room temperature in MeCN. FT-IR spectra were recorded on an ATR-FTIR (Perkin-Elmer Spectrum 100, Perkin-Elmer, Waltham, MA, USA) spectrometer. Dynamic light scattering (DLS)/Zeta potential measurements were carried out on a Malvern Zetasizer Ultra (Malvern Panalytical, Malvern, UK). Specific optical rotation was measured on an Anton Paar (Graz, Austria, MCP 200) polarimeter, in CHCl_3_ solutions at 20 °C and λ = 365 nm, 436 nm, 546 nm, 579 nm, and 589 nm.

## 4. Conclusions and Outlook

In this work, thiosemicarbazones (TSCs) were derivatized to supply them with terminal functions for covalent conjugation, including anchoring on nanoparticles (NPs). The modular character of TSCs allows us to generate thiosemicarbazides containing the functionality of conjugation at the *N*4-position of the TSC and react the thiosemicarbazides with carbonyl compounds (O=CR^1^R^2^) to form 34 functionalized TSCs R^1^R^2^C=N(1)–N(2)H–C(S)–N(4)R^3^R^4^. The functional groups di-2-pyridyl imine for metal ion coordination and 9-anthracenyl-methyl as a luminescent probe were introduced as CR^1^/R^2^ functions on the *N*1 atom through their carbonyl compounds.

In some of these 34 structures, functional groups for direct anchoring on the NPs were introduced at the terminal *N*4-R^3^/R^4^ sites, while other terminal groups were introduced for typical covalent conjugation reactions such as ester, amide or triazole formation. Amines, including di-amino alkyls or aryls and also α-amino acids as starting materials were reacted with SCCl_2_ or CS_2_ to form the corresponding thiocyanates which were transformed into the thiosemicarbazides using hydrazine. This method allowed us to synthesize a large number of TSC with Boc-protected amino acids and Boc–NH terminated diamines in the *N*4 position. It also allowed us to introduce phenyl-(ethyl)phosphonate, amino acid carboxylate, 4-hydroxy cyclohexyl, *o*-hydroquinone, S-benzyl, dimethyl-phenylene–N_3_ and phenyl–C≡C–TMS end groups. (Ethyl)phosphonates had high overall yields of 40 and 61% after five steps, and the very successful synthesis of the hydroquinone derivatives had overall yields of 76 and 85% in three steps, which opens the door to their future use as direct anchoring groups for NPs. In contrast to this, the introduction of the S-benzyl group for NP anchoring (as thiolate) requires further optimization as overall yields are moderate to low (32 and 19%). The same is true for the synthesis of the two *N*4-glucose-substituted TSCs with 20% overall yields. Unfortunately, the introduction of the trimethoxysilane group for potential direct anchoring on SiO_2_ or other metal oxides failed. However, it is only the last step, the condensation with the carbonyl compounds, that was detrimental to the silane function and thus requires further optimization.

As a proof of principle, we have de-protected the two [TSC–(ethyl)phosphonate] conjugates and anchored them on commercially available TiO_2_ NPs which was confirmed by FT-IR, dynamic light scattering and Zeta potential measurements.

Within the series of TSCs with *N*4-end groups for potential covalent conjugation, the NH–Boc and C–Boc terminated diamines and amino acids represent a huge potential. Some of them were synthesized in excellent overall yields of up to 70%, while others require optimization (yields down to 16%). The very versatile terminal N_3_ group for the important AAC (alkyne-azide click) reaction was very successfully introduced with overall yields of 36 and 42% after six steps. In this reaction procedure, it became clear that a rigid spacer is needed between the thiocyanate and the azide group for the reaction with hydrazine. The same is true for this reaction with thiocyanates with alkyne (C≡CH). For the alkynes (C≡C–TMS) and the N_3_, rigid spacers between the two functional groups allowed the successful conversion to the thiosemicarbazones, while for Br, this move did not help. For the less rigid alkyl spacers, cyclization reactions are suggested to circumvent the product formation.

Future work will include the optimization of some of the reaction protocols as described above. More importantly, with very versatile functionalized TSCs carrying either a metal-coordinating di-2-pyridyl function or a luminescent 9-anthranyl tag in hand, the NP anchoring of these TSCs either by direct coordination to the NP surface or through conjugation of the TSCs, e.g., by click reactions with surface-functionalized NPs, will be studied in depth.

## Figures and Tables

**Table 1:** Selected results from dynamic light scattering (DLS) and Zeta potential measurements ^a^.

Compound	TiO_2_ (Aeroxide P25)	[Dipy–TSC–Ph–Phos–TiO_2_]	[Anthr–TSC–Ph–Phos–TiO_2_]
Z-Average (nm)	537	185	275
Polydispersity Index	0.718	0.266	0.252
Zeta potential (mV)	10.3	–19.4	–35.7

^a^ Measured in H_2_O.

## Data Availability

Available from the authors on request.

## Supplementary Materials

The following supporting information can be downloaded at: https://www.mdpi.com/article/10.3390/molecules29153680/s1. Supplementary Materials—Part I contains the Experimental Section with details on Materials and Syntheses as well as Supplementary Figures S1–S307 showing NMR spectra. Table S1 contains specific optical rotation values for the [dipy–TSC–X] conjugates. Supplementary Materials—Part II: Figure S308. Photographs of TiO_2_ (Aeroxid P25^®^) and the [TSC–phosphonic acid–TiO_2_] conjugates. Figure S309. FT-IR spectra of pristine TiO_2_ NPs, the [TSC–Ph–phos–OH] conjugates and the [TSC–Ph–phos–TiO_2_] conjugates. Figure S310. Zoom in to the FT-IR spectra of the [TSC–Ph–phos–OH] conjugates and the [TSC–Ph–phos–TiO_2_] conjugates. Figure S311. UV-vis absorption spectra of pristine TiO_2_ NPs, the [TSC–Ph–phos–OH] conjugates and the [TSC–Ph–phos–TiO_2_] conjugates. Figure S312. Photoluminescence spectrum of the [Anthr–TSC–Ph–phos–TiO_2_] conjugate in MeOH. Figure S313. UV-vis absorption spectra of dipyridyl ketone TSCs in MeCN. Figure S314. UV-vis absorption spectra of 9-anthraaldehyde TSCs in MeCN. Figure S315. Normalized emission spectra of the 9-anthraldehyde TSCs in MeCN. Figure S316. Normalized, concentration-dependent emission spectroscopy of amino[(1*E*)-(anthracen-9-yl)methylideneamino] carbothioamide in MeCN at rt. Figure S317. Normalized, concentration-dependent emission spectroscopy of *tert*-butyl (6-{[(1*E*)-(anthracen-9-yl)methylideneaminocarbamthioyl]amino}hexyl)carbamate in MeCN at rt. Figure S318. Normalized, concentration-dependent emission spectroscopy of *tert*-butyl (4-{[(1*E*)-(anthracen-9-yl)methylideneaminocarbamthioyl]amino}phenyl)carbamate in MeCN at rt.
