# Supplementary material for: Functionalizing Thiosemicarbazones for Covalent Conjugation
Source: Molecules. 2024 Aug 3;29(15):3680. doi: 10.3390/molecules29153680 (PMC11314635; doi:10.3390/molecules29153680)
Supplement: Supplementary file 1 [file molecules-29-03680-s001.zip › molecules-3059644-supplementary Part I.pdf]

Supplementary Materials – Part I

Functionalizing Thiosemicarbazones for Covalent Conjugation

Johannes Hohnsen, Lukas Rryci, Diana Obretenova, Joshua Friedel, Shahab Jouchaghani, and Axel Klein*

University of Cologne, Faculty of Mathematics and Natural Sciences, Department of Chemistry and Biochemistry, Institute for Inorganic and Materials Chemistry, Greinstraße 6, 50939 Koeln, Germany.
* Correspondence: axel.klein@uni-koeln.de; Tel.: +49-221-470-4006; ORCID: 0000-0003-0093-9619

Contents

1. Experimental Section Page S1
1.1. Materials
1.2. Syntheses
Scheme S1. Syntheses of TSCs derived from amino acids.
Scheme S2. Syntheses of TSCs derived from diamines.
Scheme S3. Syntheses of TSCs derived from glucose.
Scheme S4. Syntheses of TSCs derived from *trans*-4-hydroxycyclohexanol.
Scheme S5. Syntheses of TSCs derived from dopamine.
Scheme S6. Syntheses of TSCs derived from amino thiols.
Scheme S7. Syntheses of TSCs with alkyne function.
Scheme S8. Syntheses of TSCs with azide function.
Scheme S9. Syntheses of TSCs with phosphonate group.

2. Supplementary Figures Page S29
Figures S1 to **S307** NMR spectra

3. Supplementary Tables Page S181
Table S1. Specific optical rotation values for the [dipy-TSC-X] conjugates.

1. Experimental Section

1.1. Materials

All chemicals were purchased from Sigma-Aldrich (St. Louis, USA), Thermo Fisher Scientific (Waltham, USA), Carbolution (St. Ingbert, Germany), BLDpharm (Reinbek, Germany), Janssen Chimica (Beerse, Belgium), Alfa Aesar (Thermo Fisher Scientific), Acros (Thermo Fisher Scientific) or Biosynth (Staad, Switzerland) and used without further purification. Thiophosgene (85%, Thermo Fisher Scientific), *tert*-butyl acetate (> 99.0%, TCI), *L*-alanine (98%, Sigma-Aldrich), *L*-valine (99%, Acros), *L*-phenylalanine (98%, Sigma-Aldrich), *L*-leucine (98%, Biosynth), *L*-isoleucine (98%, Sigma-Aldrich), *L*-aspartic acid (97%, Alfa Aesar), *L*-tyrosine *tert*-butyl ester (99%, BLDpharm), hydrazine hydrate (100%, Thermo Fisher Scientific), ethylenediamine (99%, Alfa Aesar), hexamethylenediamine (98%, Sigma-Aldrich), 1,4-phenylenediamine (98%, Sigma-Aldrich), di-*tert*-butyl dicarbonate (98%, Carbolution), acetobromo-α-D-Glucose (98%, BLDpharm), 9-antracenealdehyde (99%, Acros), di(2-pyridyl) ketone (97%, BLDpharm), *trans*-4-hydroxycyclohexanol (98%, BLDpharm), dopamine hydrochloride (99%, Sigma Aldrich), cysteamine hydrochloride (98%, BLDpharm), benzyl chloride (99%, Janssen Chimica), 4-iodoaniline (98%, Sigma-Aldrich), trimethylsilyl acetylene (98%, BLDpharm), [Pd(PPh$_3$)$_4$] (99%, Thermo Fisher Scientific), 4-cyanobenzaldehyde (97%, BLDpharm), 1-iodo-nitrobenzene (98%, Sigma-Aldrich), diethyl phosphite (98%, Sigma-Aldrich), bromotrimethylsilane (TMBS) (97%, Carbolution), titanium(IV) oxide Aeroxide® P25 (Thermo Fisher Scientific).

Reactions sensitive to oxygen or water were carried out under argon gas atmosphere (99.998%, Linde, Pullach, Germany) using the Schlenk technique. Dry THF was distilled over Na/K (alloy 3:7) before use.

Other dry solvents were dried using the solvent purification system MRBAUN MB SPS-800 (MBraun, Garching, Germany).

1.2. Synthesis

1.2.1 Synthesis of thiosemicarbazones derived from amino acids

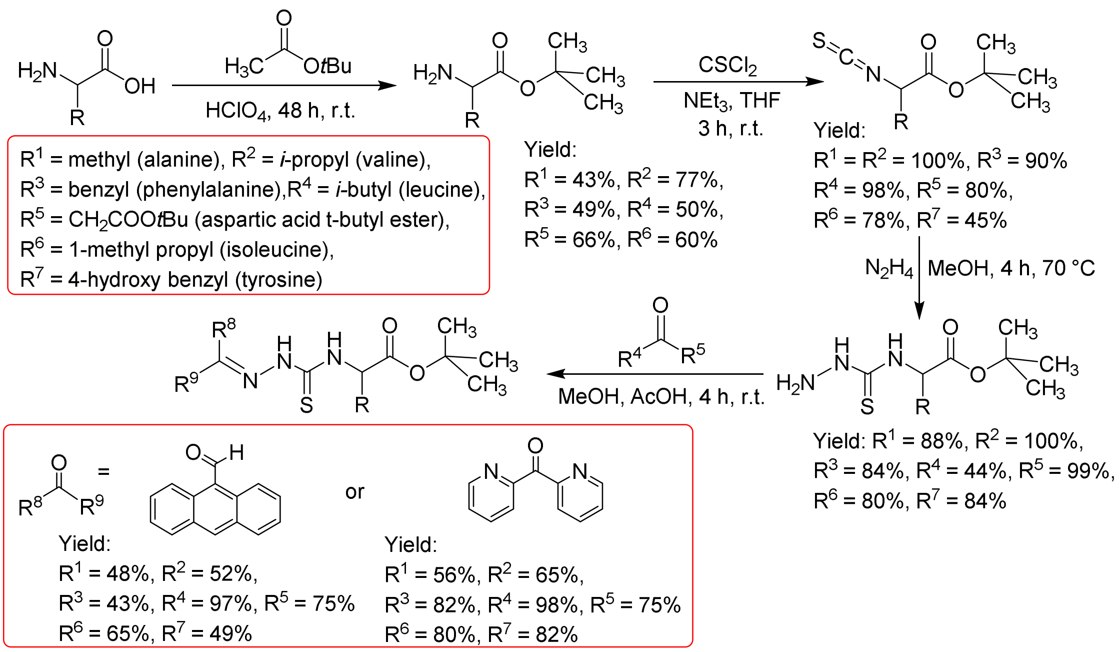

Scheme S1. Overview about the synthesis of TSCs, derived from amino acids.

1.2.1.1 Synthesis of amino acid *tert*-butyl esters – general description

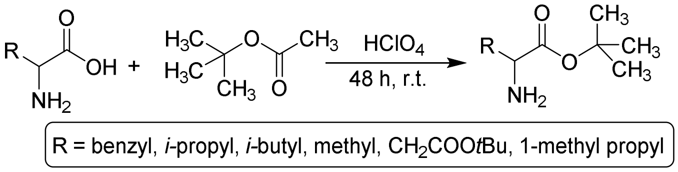

The amino acid (1 eq.) was suspended in *tert*-butyl acetate (amino acid/*tert*-butyl acetate approx. 1:30) and cooled to 0 °C. $HClO_4$ (70% in water, 1.5 eq.) was added dropwise, which led to the formation of a clear solution, which was stirred for 48 h at room temperature. Then, the reaction mixture was washed with 150 mL demineralized H_2O and 75 mL 1 M HCl and the combined aqueous phase was adjusted to pH = 9 using K_2CO_3. A white precipitate formed, which was filtered off. The aqueous solution was extracted with 3 x 100 mL CH_2Cl_2 and the organic phase was dried over Na_2SO_4. The solvent was removed under reduced pressure yielding the product as colorless oil.

***L*-alanine *tert*-butyl ester** from 6.24 g (70 mmol) *L*-alanine, 90 mL (666 mmol) *tert*-butyl acetate, 8.4 mL (106 mmol) perchloric acid. The product was obtained as colorless oil. Yield: 4.37 g (30.1 mmol, 43%). $C_7H_{15}NO_2$ (145.20 g/mol). ^{1}H NMR (499 MHz, $CDCl_3$) δ [ppm] = 3.36 (q, *J* = 7.0 Hz, 1H, H2), 1.51 (s, 2H, H6), 1.42 (s, 9H, H5), 1.26 (d, *J* = 7.1 Hz, 3H, H1). ^{13}C NMR (499 MHz, $CDCl_3$) δ [ppm] = 175.9 (C3), 80.7 (C4), 50.6 (C2), 28.0 (C5), 20.7 (C1).

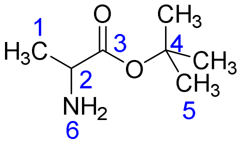

***L*-valine *tert*-butyl ester** from 5 g (42.7 mmol) *L*-valine, 90 mL (666 mmol) *tert*-butyl acetate, 5.3 mL (64.1 mmol) perchloric acid. The product was obtained as colorless oil. Yield: 5.71 g (32.9 mmol, 77%). $C_9H_{19}NO_2$ (173.26 g/mol). ^{1}H NMR (499 MHz, $CDCl_3$) δ [ppm] = 3.13 (d, *J* = 4.6 Hz, 1H, H4), 1.93 (m, 1H, H3), 1.49 (s, 2H, H8), 1.44 (s, 9H, H7), 0.95 (d, *J* = 6.7 Hz,

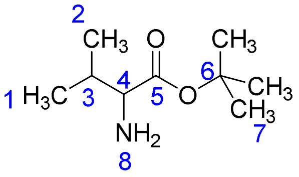

3H, H1/H2), 0.87 (d, *J* = 6.7 Hz, 3H, H1/H2). ^{13}C NMR (499 MHz, $CDCl_3$) δ [ppm] = 174.7 (C5), 80.7 (C6), 60.2 (C4), 32.1 (C3), 28.0 (C7), 19.2 (C1/C2), 17.0 (C1/C2).

***L*-phenylalanine *tert*-butyl ester** from 4.95 g (30 mmol) *L*-phenylalanine, 90 mL (666 mmol) *tert*-butyl acetate, 3.9 mL (47.2 mmol) perchloric acid. The product was obtained as colorless oil. Yield: 3.22 g (14.5 mmol, 49%). $C_{13}H_{19}NO_2$ (221.30 g/mol). ^{1}H NMR (499 MHz, $CDCl_3$) δ [ppm] = 7.28 (m, 2H, H3), 7.21 (m, 3H, H1, H2), 3.58 (m, 1H, H6), 3.04 (m, 1H, H5), 2.81 (m, 1H, H5), 1.52 (s, 2H, H10), 1.42 (s, 9H, H9). ^{13}C NMR (499 MHz, $CDCl_3$) δ [ppm] = 174.9 (C7), 138.6 (C4), 129.3 (C1/C2), 128.3 (C3), 125.4 (C1/C2), 82.0 (C8), 56.4 (C6), 41.4 (C5), 27.5 (C9).

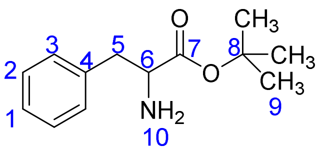

***L*-leucine *tert*-butyl ester** from 2 g (15 mmol) L-leucine, 50 mL (500.5 mmol) *tert*-butyl acetate, 1.94 mL (22.5 mmol) perchloric acid. The product was obtained as colorless oil. Yield: 1.4 g (7.5 mmol, 50%). $C_{10}H_{21}NO_2$ (187.28 g/mol). ^{1}H NMR (499 MHz, $CDCl_3$) δ [ppm] = 3.26 (m, 1H, H4), 1.71 (m, 1H, H2), 1.44–1.29 (m, 13H, H5, H3, H6), 0.86 (t, *J* = 6.8 Hz, 6H, H1).

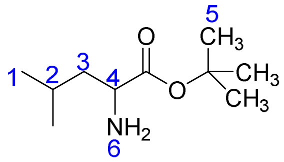

***L*-Isoleucine *tert*-butyl ester** from 2 g (15.2 mmol) L-isoleucine, 50 mL (500.5 mmol) *tert*-butyl acetate, 1.96 mL (22.9 mmol) perchloric acid. The product was obtained as colorless oil. Yield: 1.71 g (9.1 mmol, 60%). $C_{10}H_{21}NO_2$ (187.28 g/mol). ^{1}H NMR (499 MHz, $CDCl_3$) δ [ppm] = 3.28 (d, *J* = 4.5 Hz, 1H, H5), 2.87 (s, 2H, H9), 1.70 (m, 1H, H3), 1.43–1.34 (m, 10H, H2, H8), 1.14 (m, 1H, H2), 0.89–0.84 (m, 6H, H1, H4). ^{13}C NMR (499 MHz, $CDCl_3$) δ [ppm] = 173.6 (C6), 81.3 (C7), 58.9 (C5), 38.9 (C3), 28.0 (C8), 24.9 (C2), 15.4 (C1/C4), 11.7 (C1/C4).

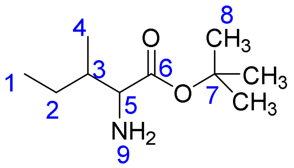

***L*-aspartic acid *tert*-butyl ester** from 0.81 g (6.1 mmol) L-aspartic acid, 50 mL (500.5 mmol) *tert*-butyl acetate, 0.6 mL (7 mmol) perchloric acid. The product was obtained as colorless oil. Yield: 1 g (4.1 mmol, 66%). $C_{12}H_{23}NO_4$ (245.32 g/mol). ^{1}H NMR (499 MHz, $CDCl_3$) δ [ppm] = 3.68 (m, 1H, H3), 2.70 (m, 2H, H2), 2.04 (s, 2H, H5), 1.45 (s, 18H, H1, H5).

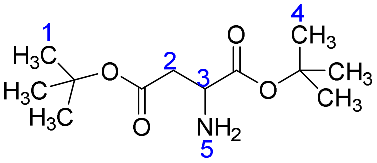

1.2.1.2 Synthesis of isothiocyanates – general description

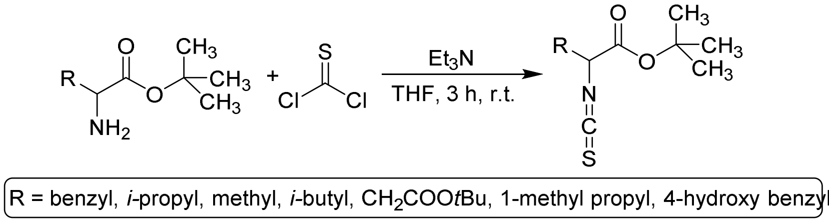

The reaction was performed under inert conditions. The amino acid *tert*-butyl ester (1 eq.) and Et_3N (3 eq.) were dissolved in 50 mL THF and thiophosgene (85%, 1 to 1.1 eq.) was slowly added at 0 °C. The reaction mixture was stirred for 3 h at room temperature, which led to the formation of a brown suspension. The reaction was quenched by adding 100 mL demineralized H_2O and the product was extracted with 3 x 100 mL Et_2O. The organic phase was dried over Na_2SO_4, and the solvent was removed under reduced pressure, yielding the isothiocyanates.

(*S*)-*tert*-butyl 2-isothiocyanatopropanoate from 1 g (6.9 mmol) alanine *tert*-butyl ester, 0.7 mL (7.6 mmol) thiophosgene, 2.86 mL (20.7 mmol) NEt_3. The product was obtained as brown oil. Yield: 1.34 g (7.1 mmol, quant.). $C_8H_{13}NO_2S$ (187.26 g/mol). ^{1}H NMR (499 MHz, $CDCl_3$) δ [ppm] = 4.17 (q, *J* = 7.2 Hz, 1H, H2), 1.53 (d, *J* = 7.4 Hz, 3H, H1), 1.49 (s, 9H, H5). ^{13}C NMR (499 MHz, $CDCl_3$) δ [ppm] = 167.6 (C3), 137.3 (C6), 83.6 (C4), 55.3 (C2), 27.7 (C5), 19.4 (C1).

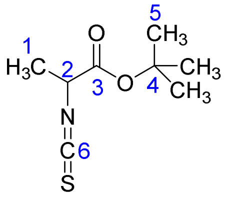

(*S*)-*tert*-butyl 2-isothiocyanato-3-metylbutanoate from 2 g (11.5 mmol) valine *tert*-butyl ester, 1.1 mL (12.7 mmol) thiophosgene, 4.8 mL (34.6 mmol) NEt_3. The product was obtained as brown oil. Yield: 2.51 g (11.5 mmol, quant.). $C_{10}H_{17}NO_2S$ (215.31 g/mol). ^1H NMR (499 MHz, $CDCl_3$) δ [ppm] = 3.98 (d, *J* = 4.6 Hz, 1H, H4), 2.28 (m, 1H, H3), 1.50 (s, 9H, H7), 1.05 (d, *J* = 7.4 Hz, 3H, H1/H2), 0.96 (d, *J* = 7.4 Hz, 3H, H1/H2). ^{13}C NMR (499 MHz, $CDCl_3$) δ [ppm] = 167.0 (C5), 135.9 (C8), 83.5 (C6), 64.5 (C4), 32.6 (C3), 28.1 (C7), 19.6 (C1/C2), 16.6 (C1/C2).

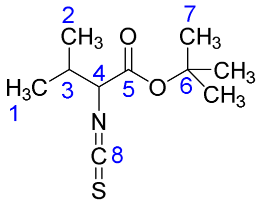

(*S*)-*tert*-butyl 2-isothiocyanato-3-phenylpropanoate from 3 g (13.5 mmol) phenylalanine *tert*-butyl ester, 1.2 mL (13.5 mmol) thiophosgene, 5.6 mL (40.7 mmol) NEt_3. The product was obtained as brown oil. Yield: 3.2 g (12.1 mmol, 90%). $C_{14}H_{17}NO_2S$ (263.36 g/mol). ^1H NMR (499 MHz, $CDCl_3$) δ [ppm] = 7.32 (m, 2H, H2), 7.27 (m, 1H, H1), 7.23 (m, 2H, H3), 4.29 (m, 1H, H6), 3.17 (m, 1H, H5), 3.09 (m, 1H, H5), 1.44 (s, 9H, H9). ^{13}C NMR (499 MHz, $CDCl_3$) δ [ppm] = 165.8 (C7), 136.5 (C10), 136.1 (C4), 129.5 (C3), 128.7 (C2), 127.7 (C1), 83.4 (C8), 61.0 (C6), 39.1 (C5), 28.1 (C9).

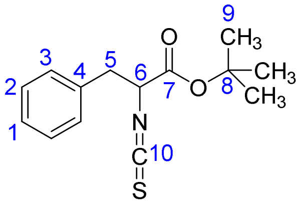

(*S*)-*tert*-butyl 2-isothiocyanato-4-methylpentanoate from 1.4 g (7.5 mmol) leucine *tert*-butyl ester, 0.68 mL (7.5 mmol) thiophosgene, 3.11 mL (22.4 mmol) NEt_3. The product was obtained as brown oil. Yield: 1.69 g (7.4 mmol, 98%). $C_{11}H_{19}NO_2S$ (229.34 g/mol). ^1H NMR (300 MHz, $CDCl_3$) δ [ppm] = 4.13 (m, 1H, H4), 1.88–1.60 (m, 3H, H2, H3), 1.50 (s, 9H, H6), 0.97 (m, 6H, H1).

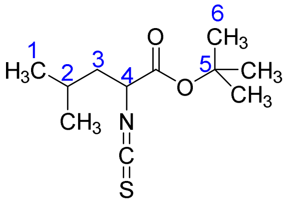

(*S*)-*tert*-butyl 2-isothiocyanato-3-methylpentanoate from 0.5 g (2.7 mmol) isoleucine *tert*-butyl ester, 0.24 mL (2.7 mmol) thiophosgene, 1.12 mL (8 mmol) NEt_3. The product was obtained as brown oil. Yield: 0.48 g (2.1 mmol, 78%). $C_{11}H_{19}NO_2S$ (229.34 g/mol). ^1H NMR (300 MHz, $CDCl_3$) δ [ppm] = 4.53 (d, *J* = 3.9 Hz, 1H, H4), 2.02 (m, 1H, H3), 1.46 (s, 9H, H5), 1.42–1.13 (m, 2H, H2), 0.97 (d, *J* = 6.8 Hz, 3H, H6), 0.89 (t, *J* = 7.4 Hz, 3H, H1).

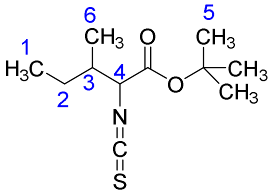

(S)-di-*tert*-butyl 2-isothiocyanatobutanedioate from 1 g (4.4 mmol) *L*-aspartic acid *tert*-butyl ester, 0.39 mL (4.4 mmol) thiophosgene, 1.82 mL (13.1 mmol) NEt_3. The product was purified by column chromatography (EtOAc) and obtained as brown oil. Yield: 1.02 g (3.5 mmol, 80%). $C_{13}H_{21}NO_4S$ (287.37 g/mol). ^1H NMR (499 MHz, $CDCl_3$) δ [ppm] = 4.48 (m, 1H, H3), 2.76 (m, 2H, H2), 1.50 (s, 9H, H1/H4), 1.48 (s, 9H, H1/H4).

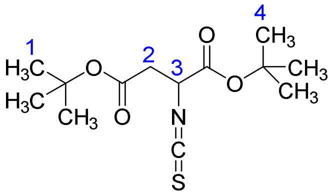

***Tert*-butyl 3-(4-hydroxyphenyl)-2-isothiocyanatopropanoate** from 0.5 g (2.1 mmol) *L*-tyrosine *tert*-butyl ester, 0.19 mL (2.1 mmol) thiophosgene, 0.88 mL (6.3 mmol) NEt_3. The reaction was cooled to −78 °C. The product was purified by column chromatography (c-hexane/EtOAc 4:1 v/v) and obtained as brown oil. Yield: 0.25 g (0.9 mmol, 42%). $C_{14}H_{17}NO_3S$ (279.35 g/mol). ^1H NMR (300 MHz, $CDCl_3$) δ [ppm] = 7.31 (d, *J* = 8.3 Hz, 2H, H3), 7.20 (d, *J* = 8.3 Hz, 2H, H4), 4.35 (s, 1H, H7), 3.20 (m, 2H, H6), 1.44 (s, 9H, H10). ^{13}C NMR (499 MHz, $CDCl_3$) δ [ppm] = 166.3 (C8), 153.1 (C2), 138.7 (C11), 133.8 (C5), 130.8 (C3), 122.3 (C2), 84.6 (C9), 60.6 (C7), 39.2 (C6), 27.8 (C10).

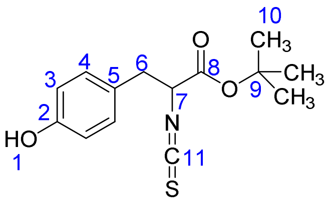

1.2.1.3 Synthesis of thiosemicarbazides – general description

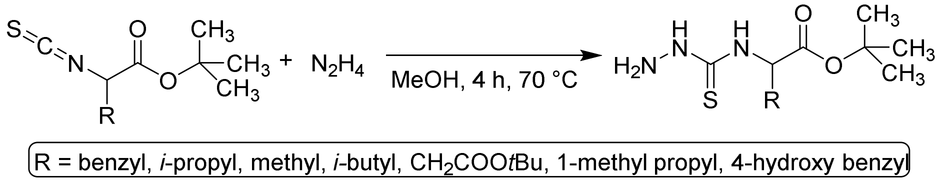

Hydrazine monohydrate (1 eq.) was dissolved in 30 mL MeOH and heated to 70 °C. The isothiocyanate (1 eq.) was dissolved in 100 mL MeOH and added to the hydrazine solution over the course of 1 h. The

reaction mixture was stirred for further 3 h, then the solvent was removed under reduced pressure. The crude product was dissolved in 10 mL $CHCl_3$ and poured into boiling petrol ether. A precipitate was formed, which was separated by decantation or filtration and the obtained product was dried under ambient conditions.

4-((*S*)-*Tert*-butyl propanoate)thiosemicarbazide from 1.29 g (6.9 mmol) (*S*)-*tert*-butyl 2-isothiocyanatopropanoate, 0.33 mL (6.9 mmol) hydrazine hydrate. The product was obtained as brownish solid. Yield: 1.35 g (6.1 mmol, 88%). $C_8H_{17}N_3O_2S$ (219.30 g/mol). ^{1}H NMR (499 MHz, DMSO-*d*$_6$) δ [ppm] = 8.90 (s, 1H, H2), 7.83 (s, 1H, H3), 4.75 (m, 1H, H5), 4.56 (s, 2H, H1), 1.42 (s, 9H, H8), 1.33 (d, *J* = 7.5 Hz, 3H, H9). ^{13}C NMR (499 MHz, $CDCl_3$) δ [ppm] = 181.0 (C3), 171.4 (C6), 81.3 (C7), 52.3 (C5), 28.1 (C8), 18.8 (C9). HR-ESI-MS: m/z $[M+H]^+$ (calc.) = 220.1114, m/z $[M+Na]^+$ (calc.) = 242.0934, m/z $[M+H]^+$ = 220.1117, m/z $[M+Na]^+$ = 242.0937.

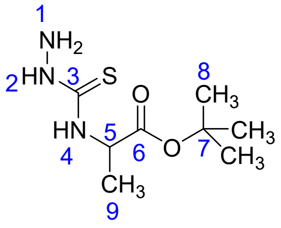

4-((*S*)-*Tert*-butyl 3-metylbutanoate)thiosemicarbazide from 2.48 g (11.5 mmol) (*S*)-*tert*-butyl 2-isothiocyanato-3-metylbutanoate, 0.55 mL (11.5 mmol) hydrazine hydrate. The product was obtained as brown solid. Yield: 3.02 g (12.2 mmol, quant.). $C_{10}H_{21}N_3O_2S$ (247.36 g/mol). ^{1}H NMR (499 MHz, DMSO-*d*$_6$) δ [ppm] = 8.96 (s, 1H, H2), 7.84 (s, 1H), 4.75 (m, 1H, H4), 4.75 (m, 1H, H5), 4.63 (s, 2H, H1), 2.13 (m, 1H, H9), 1.43 (s, 9H, H8), 0.88 (m, 6H, H10). ^{13}C NMR (499 MHz, $CDCl_3$) δ [ppm] = 181.5 (C3), 171.1 (C6), 81.6 (C7), 62.9 (C5), 31.5 (C9), 28.2 (C8), 18.6 (C10). HR-ESI-MS: m/z $[M+H]^+$ (calc.) = 248.1427, m/z $[M+Na]^+$ (calc.) = 270.1251, m/z $[M+H]^+$ = 248.1430, m/z $[M+Na]^+$ = 270.1251.

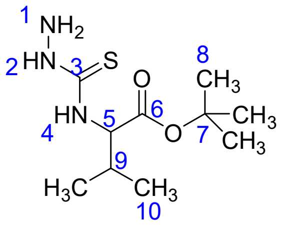

4-((*S*)-*Tert*-butyl 3-phenylpropanoate)thiosemicarbazide from 3.2 g (12.1 mmol) (*S*)-*tert*-butyl 2-isothiocyanato-3-phenylpropanoate, 0.6 mL (2.1 mmol) hydrazine hydrate. The product was obtained as brown solid. Yield: 3.01 g (10.2 mmol, 84%). $C_{14}H_{21}N_3O_2S$ (295.40 g/mol). ^{1}H NMR (499 MHz, DMSO-*d*$_6$) δ [ppm] = 8.99 (s, 1H, H2), 7.83 (s, 1H, H4), 7.30 (m, 2H, H8), 7.23 (m, 1H, H10), 7.19 (m, 2H, H9), 5.06 (m, 1H, H5), 4.56 (s, 2H, H1), 3.07 (m, 2H, H6), 1.30 (s, 9H, H13). ^{13}C NMR (499 MHz, $CDCl_3$) δ [ppm] = 181.0 (C3) 170.4 (C11), 136.7 (C7), 129.3 (C9), 128.2 (C8), 126.6 (C10), 81.2 (C12), 57.1 (C5), 37.1 (C6), 27.6 (C13). HR-ESI-MS: m/z $[M+H]^+$ (calc.) = 296.1427, m/z $[M+Na]^+$ (calc.) = 318.1247, m/z $[M+H]^+$ = 296.1432, m/z $[M+Na]^+$ = 318.1252.

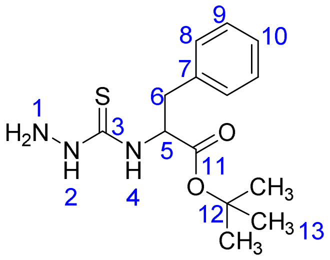

4-((*S*)-*Tert*-butyl 4-methylpentonate)thiosemicarbazide from 1.69 g (7.4 mmol) (*S*)-*tert*-butyl 2-isothiocyanato-4-methylpentanoate, 0.36 mL (7.4 mmol) hydrazine hydrate. The product was obtained as brown solid. Yield: 0.84 g (3.2 mmol, 44%). $C_{11}H_{23}N_3O_2S$ (261.38 g/mol). ^{1}H NMR (499 MHz, DMSO-*d*$_6$) δ [ppm] = 8.88 (s, 1H, H2), 7.72 (d, *J* = 7.9 Hz, 1H, H4), 4.84 (m, 1H, H5), 4.56 (s, 2H, H1), 1.70–1.49 (m, 4H, H6, H7), 1.41 (s, 9H, H11), 0.90 (t, *J* = 5.7 Hz, 6H, H8). ^{13}C NMR (499 MHz, DMSO-*d*$_6$) δ [ppm] = 181.6 (C3), 172.4 (C9), 81.2 (C10), 55.2 (C5), 41.4 (C6), 28.1 (C11), 24.9 (C7), 23.1 (C8), 22.5 (C8). HR-ESI-MS: m/z $[M+H]^+$ (calc.) = 262.1584, m/z $[M+Na]^+$ (calc.) = 284.1403, m/z $[M+H]^+$ = 262.1585, m/z $[M+Na]^+$ = 284.1405.

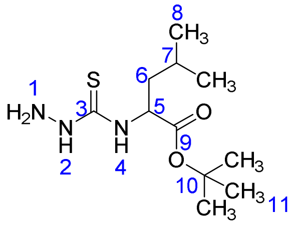

(*S*)-*Tert*-butyl 2-(hydrazidecarbothioamino)-3-methylpentanoate from 0.43 g (1.9 mmol) (*S*)-*tert*-butyl 2-isothiocyanato-3-methylpentanoate, 0.09 mL (1.9 mmol) hydrazine hydrate. The product was obtained as brown solid. Yield: 0.45 g (1.7 mmol, 90%). $C_{11}H_{23}N_3O_2S$ (261.38 g/mol). ^{1}H NMR (499 MHz, DMSO-*d*$_6$) δ [ppm] = 7.99 (s, 1H, H2), 7.90 (d, *J* = 8.4 Hz, 1H, H3), 4.88 (m, 1H, H4), 3.88 (s, 2H, H1), 1.99 (m, 1H, H5), 1.56–1.12 (m, 11H, H6, H9), 0.98–0.89 (m, 6H, H7/H8).

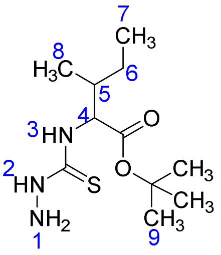

(*S*)-Di-*tert*-butyl 2-(hydrazidecarbothioamino)butanedioate from 0.43 g (1.7 mmol) (*S*)-di-*tert*-butyl 2-isothiocyanatobutanedioate, 0.08 mL (1.7 mmol) hydrazine hydrate. The product was obtained as brown solid. Yield: 0.55 g (1.7 mmol, 99%). $C_{13}H_{25}N_3O_4S$ (319.42 g/mol). ^{1}H NMR (300 MHz, DMSO-*d*$_6$) δ [ppm] = 9.01 (s, 1H, H2), 8.11 (d, *J* = 8.0 Hz, 1H, H3), 5.10 (m, 1H, H4), 4.59 (s, 2H, H1), 2.80 (m, 2H, H5), 1.42–1.40 (m, 18H, H6, H7). HR-ESI-MS: m/z [M+H]$^+$ (calc.) = 320.1639, m/z [M+Na]$^+$ (calc.) = 342.1458, m/z [M+H]$^+$ = 320.1639, m/z [M+Na]$^+$ = 342.1460.

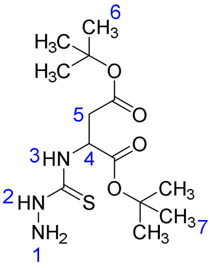

(*S*)-*Tert*-butyl 2-(hydrazidecarbothioamino)-3-(4-hydroxyphenyl)propanoate from 0.16 g (0.6 mmol) *tert*-butyl-3 (4-hydroxyphenyl)-2-isothiocyanatopropanoate, 0.03 mL (0.6 mmol) hydrazine hydrate. The product was obtained as brown solid. Yield: 0.15 g (0.5 mmol, 88%). $C_{14}H_{21}N_3O_3S$ (311.40 g/mol). ^{1}H NMR (300 MHz, DMSO-*d*$_6$) δ [ppm] = 8.95 (s, 1H, H2), 7.77 (d, *J* = 7.4 Hz, 1H, H4), 6.96 (d, *J* = 8.5 Hz, 2H, H9), 6.68 (d, *J* = 8.4 Hz, 2H, H8) 4.96 (m, 1H, H5), 4.55 (s, 2H, H1), 2.97 (m, 2H, H6), 1.35 (s, 9H, H14). ^{13}C NMR (300 MHz, DMSO-*d*$_6$) δ [ppm] = 180.8 (C3) 170.9 (C12), 156.5 (C10), 130.7 (C9), 127.0 (C7), 115.5 (C8), 81.5 (C13), 57.8 (C5), 37.4 (C6), 28.0 (C14). HR-ESI-MS: m/z [M+H]$^+$ (calc.) = 312.1376, m/z [M+Na]$^+$ (calc.) = 334.1196, m/z [M+H]$^+$ = 312.1375, m/z [M+Na]$^+$ = 334.1195.

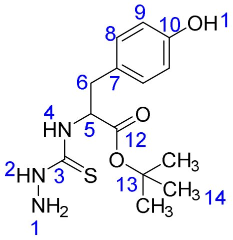

1.2.1.3 Synthesis of thiosemicarbazones – general description

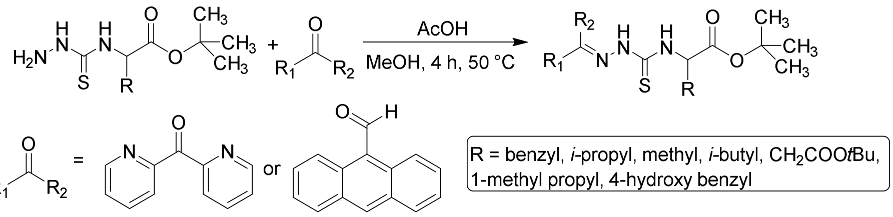

The thiosemicarbazide (1 eq.) and antracene-9-carbaldehyde or di-(2-pyridyl) ketone (1 eq.) were dissolved in a minimum amount of MeOH. A few drops glacial AcOH or HCl were added, and the reaction mixture was sonicated at 50 °C for 4 h or refluxed overnight. The solvent was removed under reduced pressure and the crude product was purified by column chromatography.

9-Anthraldehyde-4-((*S*)-*tert*-butylpropanoate)-3-thiosemicarbazone from 0.3 g (1.4 mmol) 4-((*S*)-*tert*-butylpropanoate)thiosemicarbazide, 0.28 g (1.4 mmol) antracene-9-carbaldehyde. The reaction mixture was sonicated for 4 h. The product was purified by column chromatography (*c*-hexane/EtOAc 8:1 v/v) and obtained as yellow solid. Yield: 0.27 g (0.7 mmol, 48%). $C_{23}H_{25}N_3O_2S$ (407.53 g/mol). ^{1}H NMR (499 MHz, DMSO-*d*$_6$) δ [ppm] = 11.94 (s, 1H, H10), 9.34 (s, 1H, H9), 8.73 (s, 1H, H1), 8.52 (d, *J* = 8.3 Hz, 2H, H6), 8.21 (d, *J* = 8.3 Hz, 1H, H12), 8.15 (d, *J* = 8.5 Hz, 2H, H3), 7.65–7.57 (m, 4H, H4, H5), 4.87 (m, 1H, H13), 1.44–1.42 (m, 12H, H16, H17). ^{13}C NMR (499 MHz, DMSO-*d*$_6$) δ [ppm] = 177.3 (C11), 170.8 (C14), 142.4 (C9), 131.3 (C2), 130.0 (C1), 129.9 (C7), 129.5 (C3), 127.9 (C5), 126.1 (C4), 125.5 (C8), 125.0 (C6), 82.5 (C15), 53.0 (C13), 28.8 (C16), 18.4 (C17).

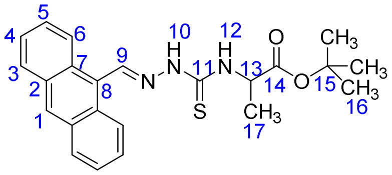

9-Anthraldehyde-4-((*S*)-*tert*-butyl 3-metylbutanoate)-3-thiosemicarbazone from 0.3 g (1.2 mmol) 4-((*S*)-*tert*-butyl-3-metylbutanoate)thiosemicarbazide, 0.25 g (1.2 mmol) antracene-9-carbaldehyde. The reaction mixture was sonicated for 4 h. The product was purified by column chromatography (*c*-hexane/EtOAc 6:1 v/v) and obtained as orange solid. Yield: 0.27 g (0.6 mmol, 52%). $C_{25}H_{29}N_3O_2S$ (435.59 g/mol). ^{1}H NMR (499 MHz, DMSO-*d*$_6$) δ [ppm] = 12.09 (s, 1H, H10), 9.38 (s, 1H, H9), 8.74 (s, 1H, H1), 8.57 (d, *J* = 7.4 Hz, 2H, H6), 8.16 (d, *J* = 7.4 Hz, 2H, H3), 7.90 (d, *J* = 7.9 Hz, 1H, H12), 7.60 (m, 4H, H4, H5), 4.81 (m, 1H, H13), 2.27 (m, 1H, H14), 1.45 (s, 9H, H18), 0.97 (m, 6H, H15) ^{13}C NMR (499 MHz, DMSO-*d*$_6$) δ [ppm]

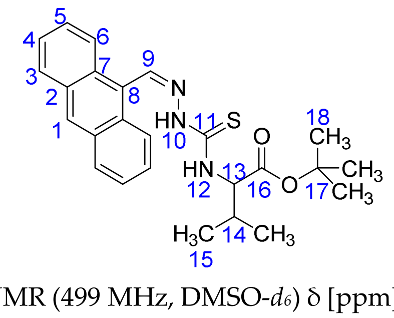

= 177.9 (C11), 170.5 (C16), 142.55 (C9), 131.7 (C2), 130.3 (C2), 130.1 (C7), 129.5 (C3), 127.7 (C4/C5), 126.0 (C4/C5), 125.1 (C8), 124.7 (C6), 82.1 (C17), 62.3 (C13), 32.7 (C14), 27.5 (C18), 18.1 (C15).

9-Anthraldehyde-4-((*S*)-*tert*-butyl 4-methylbutanoate)-3-thiosemicarbazone from 0.3 g (1 mmol) 4-((*S*)-*tert*-butyl-3-phenylpropanoate)thiosemicarbazide, (0.2 g, 1 mmol) antracene-9-carbaldehyde. The product was purified by column chromatography (c-hexane/EtOAc 8:1 v/v) and obtained as yellow solid. Yield: 0.21 g (0.4 mmol, 43%). $C_{29}H_{29}N_3O_2S$ (483.63 g/mol). ^{1}H NMR (499 MHz, DMSO-d_6) δ [ppm] = 12.01 (s, 1H, H10), 9.33 (s, 1H, H9), 8.73 (s, 1H, H1), 8.47 (m, 2H, H6), 8.15 (m, 2H, H3), 8.08 (d, *J* = 6.6 Hz, 1H, H12), 7.58 (m, 4H, H4, H5), 7.25 (m, 4H, H16, H17), 7.22 (m, 1H, H18), 5.13 (m, 1H, H13), 3.24 (m, 2H, H14), 1.36 (s, 9H, H21). ^{13}C NMR (499 MHz, DMSO-d_6) δ [ppm] = 177.4 (C11), 169.9 (C19), 142.8 (C9), 137.0 (C15), 131.4 (C2), 130.1 (C7), 130.0 (C1), 129.6 (C3), 129.1 (C16, C17), 127.7 (C4/C5), 127.5 (C18), 126.0 (C4/C5), 125.3 (C8), 125.0 (C6), 82.6 (C20), 58.1 (C13), 37.2 (C14), 28.0 (C21).

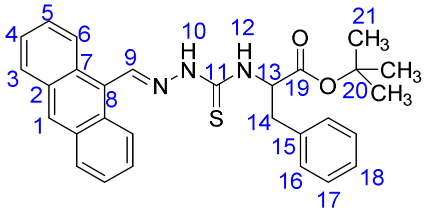

9-Anthraldehyde-4-((*S*)-*tert*-butyl 4-methylpentonate)-3-thiosemicarbazone from 0.2 g (0.8 mmol) 4-((*S*)-*tert*-butyl-4-methylpentonate)thiosemicarbazide, 0.16 g (0.8 mmol) antracene-9-carbaldehyde. The product was obtained as yellow solid. Yield: 0.36 g (0.8 mmol, 100%). $C_{26}H_{31}N_3O_2S$ (449.61 g/mol). ^{1}H NMR (499 MHz, DMSO-d_6) δ [ppm] = 11.99 (s, 1H, H7), 9.34 (s, 1H, H6), 8.72 (s, 1H, H1), 8.52 (d, *J* = 8.0 Hz, 2H, H5), 8.14 (m, 3H, H2), 7.58 (m , 4H, H3, H4), 4.96 (m, 1H, H9), 1.86–1.62 (m , 3H, H10, H11), 1.42 (s, 9H, H13), 0.93 (m, 6H, H12). HR-ESI-MS: m/z $[M+H]^+$ (calc.) = 450.2210, m/z $[M+Na]^+$ (calc.) = 472.2029, m/z $[M+H]^+$ = 450.2214, m/z $[M+Na]^+$ = 472.2032.

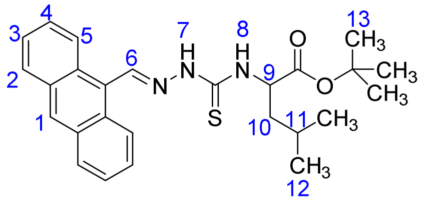

***Tert*-butyl-2-{[(1*E*)-(anthracen-9-yl)methylideneaminocarbamthioyl]amino}-3-methylpentanoate** from 0.1 g (0.4 mmol) (*S*)-*tert*-butyl-2-(hydrazidecarbothioamino)-3-methylpentanoate, 0.08 g (0.4 mmol) antracene-9-carbaldehyde. The product was obtained as yellow solid. Yield: 0.15 g (0.34 mmol, 90%). $C_{26}H_{31}N_3O_2S$ (449.61 g/mol). ^{1}H NMR (499 MHz, DMSO-d_6) δ [ppm] = 12.11 (s, 1H, H7), 9.39 (s, 1H, H6), 8.76 (s, 1H, H1), 8.61 (m, 2H, H5), 8.18 (m, 2H, H2), 8.04 (d, *J* = 8.7 Hz, 1H, H8), 7.62 (m, 4H, H3, H4), 5.04 (m, 1H, H9), 2.05 (m, 1H, H10), 1.46 (s, 9H, H14), 1.23 (m, 2H, H11), 0.92 (m, 6H, H12, H13).

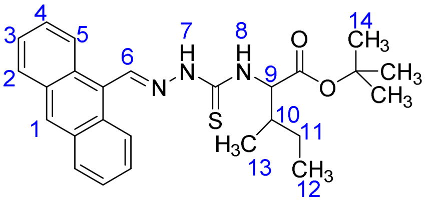

Di-*tert*-butyl-2-{[(1*E*)-(anthracen-9-yl)methylideneaminocarbamthioyl]amino} butanedioate from 0.2 g (0.8 mmol) (*S*)-di-*tert*-butyl-2-(hydrazide-carbothioamino)butanedioate**,** 0.16 g (0.8 mmol). antracene-9-carbaldehyde. The product was obtained as yellow solid. Yield: 0.29 g (0.6 mmol, 75%). $C_{28}H_{33}N_3O_4S$ (507.65 g/mol). ^{1}H NMR (499 MHz, DMSO-d_6) δ [ppm] = 12.06 (s, 1H, H7), 9.38 (s, 1H, H6), 8.74 (s, 1H, H1), 8.58 (d, *J* = 8.4 Hz, 2H, H5), 8.41 (d, *J* = 8.3 Hz, 1H, H2), 8.17 (d, *J* = 7.7 Hz, 2H, H8), 7.61 (p, *J* = 5.8 Hz, 4H, H3, H4), 5.21 (m, 1H, H9), 2.91 (d, *J* = 4.6 Hz, 2H, H10), 1.41 (s, 9H, H11/H12), 1.29 (s, 9H, H11/H12). HR-ESI-MS: m/z $[M+H]^+$ (calc.) = 508.2265, m/z $[M+Na]^+$ (calc.) = 530.2084 m/z $[M+H]^+$ = 508.2274, m/z $[M+Na]^+$ = 530.2084.

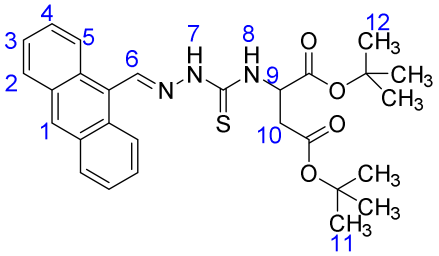

***Tert*-butyl-2-[(2*E*)-2-[(anthracen-9-yl)methylidene]hydrazinocarbamthioyl]-3-(4-hydroxyphenyl) propanoate** from 0.1 g (0.32 mmol) (*S*)-*tert*-butyl-2-(hydrazidecarbothioamino)-3-(4-hydroxyphenyl) propanoate, 0.07 g (0.32 mmol) antracene-9-carbaldehyde. The product was purified by column chromatography (*c*-hexane/EtOAc 4:1) and obtained as yellow solid. Yield: 0.07 g (0.14 mmol, 45%). $C_{29}H_{29}N_3O_3S$ (499.63 g/mol). ^{1}H NMR (499 MHz, DMSO-d_6) δ [ppm] = 11.99 (s, 1H), 9.34 (s, 1H), 9.25 (s, 1H), 8.74 (s, 1H), 8.74 (m, 2H), 8.16

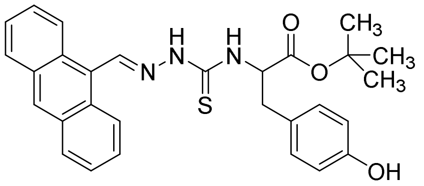

(m, 2H), 7.99 (d, *J* = 7.9 Hz, 1H), 7.04 (d, *J* = 8.3 Hz, 2H), 6.64 (d, *J* = 8.4, 2H), 5.04 (m, 1H), 3.13 (m, 2H), 1.36 (s, 9H).

Di-2-pyridylketone-4-((*S*)-*tert*-butyl propanoate)-3-thiosemicarbazone from 0.3 g (1.4 mmol) 4-((*S*)-*tert*-butylpropanoate)thiosemicarbazide, 0.25 g (1.4 mmol) di-pyridyl ketone. The product was purified by column chromatography (*c*-hexane/EtOAc 1:2 v/v) and obtained as orange solid. Yield: 0.3 g (0.8 mmol, 56%). $C_{19}H_{23}N_5O_2S$ (385.49 g/mol). ^{1}H NMR (499 MHz, DMSO-d_6) δ [ppm] = 13.53 (s, 1H, H12), 8.82 (d, *J* = 4.4 Hz, 1H, H1), 8.72 (d, *J* = 8.6 Hz, 1H, H14), 8.59 (d, *J* = 4.3 Hz, 1H, H11), 8.14 (d, *J* = 7.4 Hz, 1H, H4), 7.98 (m, 2H, H3, H3, H9), 7.59 (m, 1H, H10), 7.49 (m, 2H, H2, H8), 4.88 (m, 1H), 1.48 (d, *J* = 7.3 Hz, 3H), 1.43 (s, 9H). ^{13}C NMR (499 MHz, DMSO-d_6) δ [ppm] = 178.1 (C13), 171.5 (C16), 155.5 (C7), 151.5 (C5), 149.0 (C11), 148.7 (C1), 142.3 (C6), 138.1 (C3), 137.7 (C9), 127.7 (C8), 125.4 (C10), 124.6 (C2), 124.3 (C4), 82.2 (C17), 62.6 (C15), 28.1 (C18), 19.1 (C19). HR-ESI-MS: m/z [M+H]$^+$ (calc.) = 386.1645, m/z [M+Na]$^+$ (calc.) = 408.1465, m/z [M+H]$^+$ = 386.1650, m/z [M+Na]$^+$ = 408.1469.

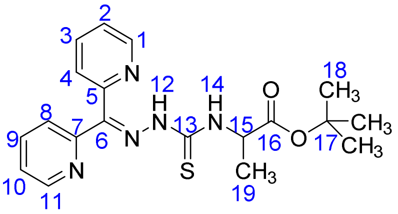

Di-2-pyridylketone-4-((*S*)-*tert*-butyl 3-metylbutanoate)-3-thiosemicarbazone from 0.3 g (1.2 mmol) 4-((*S*)-*tert*-butyl-3-metylbutanoate)thiosemicarbazide, 0.22 g (1.2 mmol) di-pyridyl ketone. The product was purified by column chromatography (*c*-hexane/EtOAc 1:3 v/v) and obtained as yellowish solid. Yield: 0.32 g (0.8 mmol, 65%). $C_{21}H_{27}N_5O_2S$ (413.54 g/mol). ^{1}H NMR (499 MHz, DMSO-d_6) δ [ppm] = 13.59 (s, 1H, H12), 8.84 (m, 1H, H1), 8.62 (m, 1H, H11), 8.36 (m, 1H, H14), 8.00 (m, 3H, H3, H8, H9), 7.60 (m, 1H, H2), 7.52 (m, 2H, H4, H10), 4.74 (m, 1H, H15), 2.30 (m, 1H, H16), 1.44 (s, 9H, H20), 0.95 (m, 6H, H17). ^{13}C NMR (499 MHz, DMSO-d_6) δ [ppm] = 178.3 (C13), 170.3 (C18), 155.5 (C7), 151.5 (C5), 149.1 (C11), 148.7 (C1), 142.3 (C6), 138.2 (C3), 137.9 (C9), 127.7 (C10), 125.4 (C2), 124.5 (C4), 123.8 (C8), 82.0 (C19), 62.6 (C15), 31.1 (C16), 28.6 (C19), 18.9 (C17). HR-ESI-MS: m/z [M+H]$^+$ (calc.) = 414.1958, m/z [M+Na]$^+$ (calc.) = 436.1778, m/z [M+H]$^+$ = 414.1961, m/z [M+Na]$^+$ = 436.1782.

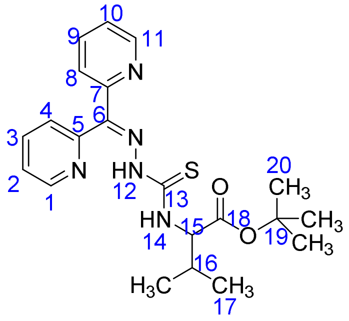

Di-2-pyridylketone-4-((*S*)-*tert*-butyl 3-phenylbutanoate)-3-thiosemicarbazone from 0.3 g (1 mmol) 4-((*S*)-*tert*-butyl-3-phenylpropanoate)thiosemicarbazide, 0.18 g (1 mmol) di-pyridyl ketone. The product was purified by column chromatography (*c*-hexane/EtOAc 1:2 v/v) and obtained as colorless solid. Yield: 0.38 g (0.8 mmol, 82%). $C_{25}H_{27}N_5O_2S$ (461.58 g/mol). ^{1}H NMR (499 MHz, DMSO-d_6) δ [ppm] = 13.57 (s, 1H, H12), 8.81 (d, *J* = 4.2 Hz, 1H, H1), 8.61 (d, *J* = 4.4 Hz, 1H, H11), 8.53 (d, *J* = 7.9 Hz, 1H, H14), 8.05–7.93 (m , 3H, H3, H8, H9), 7.61–7.48 (m, 3H, H2, H4, H10), 7.32–7.21 (m, 5H, H19, H20, H21), 5.08 (m, 1H, H15), 3.28 (m, 2H, H16), 1.38 (s, 9H, H24). ^{13}C NMR (499 MHz, DMSO-d_6) δ [ppm] = 178.2 (C13), 170.1 (C21), 155.4 (C7), 151.3 (C5), 149.0 (C11), 148.6 (C1), 142.1 (C6), 138.1 (C3/C8/C9), 137.6 (C3/C8/C9), 137.2 (C18), 129.9 (C18/C19/C20), 128.9 (C18/C19/C20), 127.7 (C2/C4/C10), 127.7 (C18/C19/C20), 125.4 (C2/C4/C10), 124.5 (C2/C4/C10), 124.0 (C3/C8/C9), 82.2 (C23), 59.2 (C15), 37.1 (C16), 27.9 (C24). HR-ESI-MS: m/z [M+H]$^+$ (calc.) = 462.1958, m/z [M+Na]$^+$ (calc.) = 484.1778, m/z [M+H]$^+$ = 462.1961, m/z [M+Na]$^+$ = 484.1780.

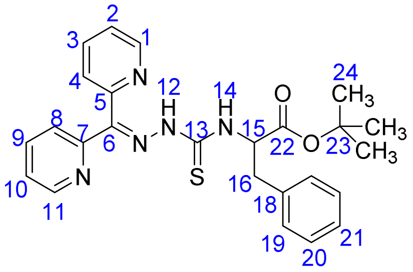

Di-2-pyridylketone-4-((*S*)-*tert*-butyl-4-methylpentanoate)-3-thiosemicarbazone from 0.2 g (0.8 mmol) 4-((*S*)-*tert*-butyl-4-methylpentonate)thiosemicarbazide, 0.14 g (0.8 mmol) di-pyridyl ketone. The product was obtained as brown oil. Yield: 0.34 g (0.8 mmol, quant.). $C_{22}H_{29}N_5O_2S$ (427.57 g/mol). ^{1}H NMR (499 MHz, DMSO-d_6) δ [ppm] = 13.42 (s, 1H), 11.94, (s, 1H), 8.84 (d, *J* = 4.1 Hz, 1H), 8.69 (d, *J* = 8.3 Hz, 1H), 8.60 (d, *J* = 3.9 Hz, 1H), 8.17 (d, *J* = 7.8 Hz, 1H), 8.00 (t, *J* = 7.5 Hz, 2H), 7.62 (m, 1H), 7.52 (m, 2H), 4.94 (m, 1H), 3.17 (m, 1H), 1.67 (m, 2H), 1.43 (s, 9H), 0.93 (t, *J* = 5.6 Hz, 6H).

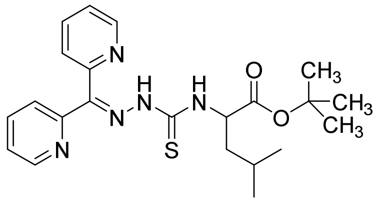

HR-ESI-MS: m/z $[M+H]^+$ (calc.) = 428.2115, m/z $[M+Na]^+$ (calc.) = 450.1934, m/z $[M+H]^+$ = 428.2116, m/z $[M+Na]^+$ = 450.1936.

***Tert*-butyl 2-{2-[bis(pyridin-2-yl)methylidene]hydrazinocarbamthioyl}-3-methylpentanoate** from 0.1 g (0.5 mmol) (*S*)-*tert*-butyl-2-(hydrazidecarbothioamino)-3-methylpentanoate, 0.14 g (0.5 mmol) di(2-pyridyl) ketone. The product was purified by column chromatography (*c*-hexane/EtOAc 1:2 v/v) and obtained as brown oil. Yield: 0.17 g (0.4 mmol, 80%). $C_{22}H_{29}N_5O_2S$ (427.57 g/mol). ^{1}H NMR (499 MHz, DMSO-d_6) δ [ppm] = 13.64 (s, 1H, H12), 8.85 (d, *J* = 4.3 Hz, 1H, H1), 8.62 (d, *J* = 4.6 Hz, 1H, H11), 8.38 (d, *J* = 8.2 Hz, 1H, H14), 8.00 (m, 3H, H3, H8, H9), 7.60 (m, 1H, H2), 7.50 (m, 2H, H4, H10), 4.83 (m, 1H, H15), 2.09 (m, 1H, H16), 1.53 (m, 1H), 1.44 (s, 9H, H22), 1.30 (m, 2H, H17), 0.33 (m, 6H, H18, H19). ^{13}C NMR (499 MHz, DMSO-d_6) δ [ppm] = 177.8 (C13), 170.4 (C20), 155.5 (C7), 151.6 (C5), 149.1 (C11), 148.7 (C1), 142.4 (C6), 138.2 (C3/C8/C9), 137.9 (C3/C8/C9), 127.6 (C4/C10), 125.4 (C2), 124.6 (C4/C10), 123.8 (C3/C8/C9), 81.8 (C21), 61.3 (C15), 37.5 (C16), 28.1 (C22), 26.1 (C17), 11.7 (C18/C19), 15.6 (C18/C19). HR-ESI-MS: m/z $[M+H]^+$ = 428.2116, m/z $[M+Na]^+$ = 450.1936.

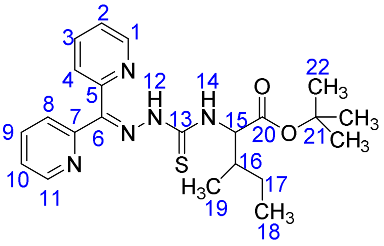

Di-*tert*-butyl 2-{[bis(pyridin-2-yl)methylideneaminocarbamthioyl]amino}butanedioate from 0.2 g (0.8 mmol) (*S*)-di-*tert*-butyl-2-(hydrazide-carbothioamino)butanedioate, 0.15 g (0.8 mmol) di(2-pyridyl ketone. The product was purified by column chromatography (*c*-hexane/EtOAc 1:2 v/v) and obtained as brown oil. Yield: 0.3 g (0.6 mmol, 75%). $C_{24}H_{31}N_5O_4S$ (485.60 g/mol). ^{1}H NMR (499 MHz, DMSO-d_6) δ [ppm] = 13.69 (s, 1H, H12), 8.86 (d, *J* = 4.2 Hz, 1H, H1), 8.79 (d, *J* = 8.5 Hz, 1H, H14), 8.62 (d, *J* = 4.7 Hz, 1H, H11), 7.99 (m, 3H, H3, H8, H9), 7.62 (m, 1H, H2), 7.51 (m, 2H, H4, H10), 5.18 (m, 1H, H15), 2.92 (d, *J* = 5.3 Hz, 2H, H16), 1.43 (s, 9H, H19/H22), 1.36 (s, 9H, H19/H22). ^{13}C NMR (499 MHz, DMSO-d_6) δ [ppm] = 178.2 (C13), 169.9 (C17/C20), 169.4 (C17/C20), 155.6 (C7), 151.5 (C5), 149.7 (C11), 148.7 (C1), 142.5 (C6), 138.2 (C3/C8/C9), 137.7 (C3/C8/C9), 127.6 (C4/C10), 125.3 (C2), 124.4 (C4/C10), 123.9 (C3/C8/C9), 82.3 (C18/C21), 81.2 (C18/C21), 53.8 (C15), 37.2 (C16), 28.0 (C19, C22). HR-ESI-MS: m/z $[M+H]^+$ (calc.) = 486.2170, m/z $[M+Na]^+$ (calc.) = 508.1989, m/z $[M+H]^+$ = 486.2173, m/z $[M+Na]^+$ = 508.1994.

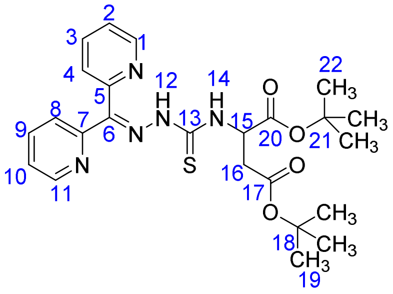

***Tert*-butyl 2-{2-[bis(pyridin-2-yl)methylidene]hydrazinocarbamthioyl}-3-(4-hydroxyphenyl)-propanoate** from 0.1 g (0.3 mmol) (*S*)-*tert*-butyl-2-(hydrazidecarbothioamino)-3-(4-hydroxyphenyl) propanoate, 0.07 g (0.3 mmol) di(2-pyridyl) ketone. The product was purified by column chromatography (*c*-hexane/EtOAc 1:3 v/v) and obtained as brown oil. Yield: 0.12 g (0.25 mmol, 82%). $C_{25}H_{27}N_5O_3S$ (477.58 g/mol). ^{1}H NMR (499 MHz, DMSO-d_6) δ [ppm] = 13.53 (s, 1H, H12), 9.30 (s, 1H, H21), 8.84 (d, *J* = 6.6 Hz, 1H, H1), 8.61 (d, *J* = 5.6 Hz, 1H, H11), 8.39 (d, *J* = 8.5 Hz, 1H, H14), 8.00 (m, 3H, H3, H8, H9), 7.59 (m, 1H, H2), 7.52 (m, 2H, H4, H10), 7.07 (d, *J* = 8.5 Hz, 2H, H18), 6.69 (d, *J* = 8.5, 2H, H19), 4.95 (m, 1H, H15), 3.10 (m, 2H, H16), 1.40 (s, 9H, H24). ^{13}C NMR (499 MHz, DMSO-d_6) δ [ppm] = 177.7 (C13), 169.1 (C22), 156.8 (C17), 155.6 (C7), 151.3 (C5), 148.9 (C11), 148.5 (C1), 142.0 (C6), 138.1 (C3/C8/C9), 137.7 (C3/C8/C9), 130.4 (C18) 127.7 (C4/C10), 126.7 (C20), 125.4 (C2), 124.4 (C4/C10), 124.0 (C3/C8/C9), 115.9 (C20), 81.9 (C23), 58.8 (C15), 36.2 (C16), 28.1 (C24). HR-ESI-MS: m/z $[M+H]^+$ (calc.) = 478.1907, m/z $[M+Na]^+$ (calc.) = 500.1727, m/z $[M+H]^+$ = 478.1911, m/z $[M+Na]^+$ = 500.1733.

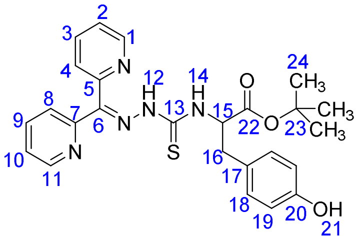

1.2.2 Synthesis of thiosemicarbazones derived from diamines

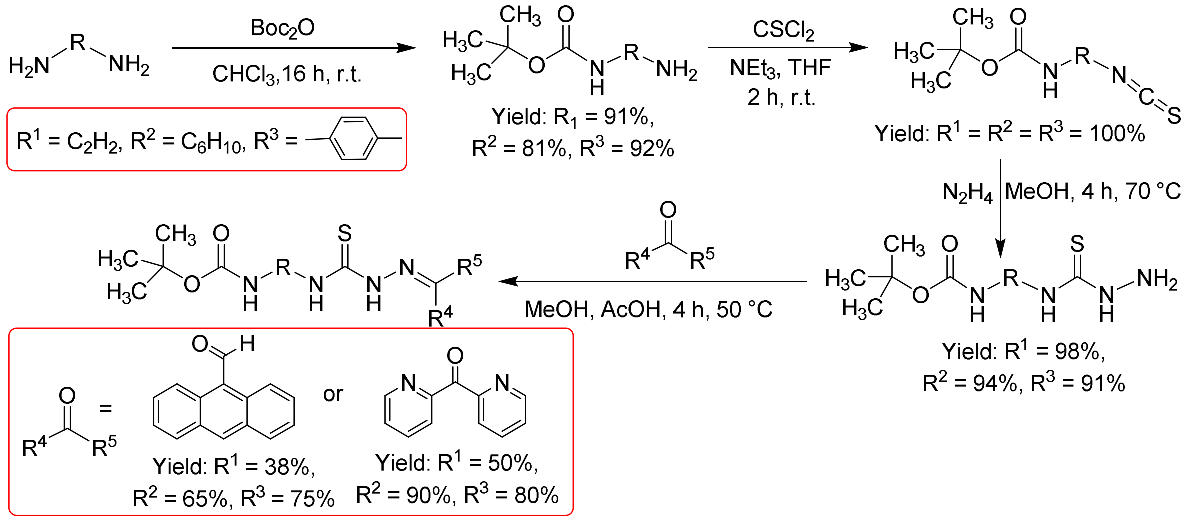

Scheme S2. Overview about the synthesis of TSCs derived from diamines.

1.2.2.1 Synthesis of mono-BOC-protected diamines

***Tert*-butyl *N*-(2-aminoethyl)carbamate**

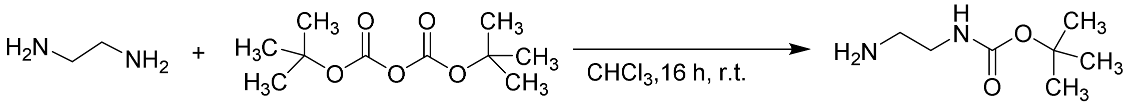

1,2-diaminoethane (28 mL, 419.3 mmol, 10 eq.) was dissolved in 400 mL $CHCl_3$ and a solution of di-*tert*-butyldicarbonate (9.14 g, 41.9 mmol, 1 eq.) in 200 mL $CHCl_3$ was added dropwise at 0 °C over a period of 3 h. The reaction mixture was stirred for further 16 h at room temperature. The solution was washed with 6 x 100 mL brine and 1 x 100 mL H_2O, dried over $MgSO_4$ and the solvent was removed under reduced pressure. The product was obtained as colorless oil. Yield: 6.09 g (38 mmol, 91%). $C_7H_{16}N_2O_2$ (160.22 g/mol). ^{1}H NMR (300 MHz, $CDCl_3$) δ [ppm] = 4.98 (s, 1H, H4), 3.13 (m, 2H, H2/H3), 2.77 (t, J = 5.8 Hz, 2H, H2/H3), 1.42 (s, 9H, H5), 1.28 (s, 2H, H1).

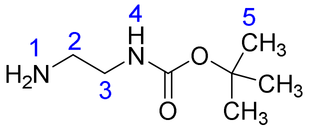

***Tert*-butyl *N*-(6-aminohexyl)carbamate**

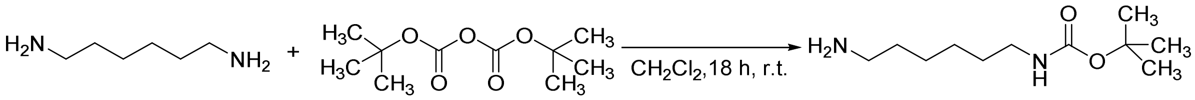

1,6-Hexadiamine (11.7 g, 101 mmol, 1 eq.) was dissolved in 100 mL CH_2Cl_2 and the solution was cooled to 0 °C. A solution of 4.4 g di-*tert*-butyl dicarbonate (20.2 mmol, 0.2 eq.) in 30 mL CH_2Cl_2 was added dropwise, which led to the formation of a colorless precipitate. The reaction mixture was stirred for 1 h at 0 °C and for further 18 h at room temperature. The precipitate was filtered off, washed with CH_2Cl_2 and the solvent was removed under reduced pressure. The crude product was dissolved in EtOAc and washed with 3 x 50 mL brine. The organic phase was separated, dried over $MgSO_4$ and the solvent was removed under reduced pressure, yielding the product as colorless oil. Yield: 3.5 g (16.4 mmol, 81%). $C_{11}H_{24}N_2O_2$ (216.33 g/mol). ^{1}H NMR (300 MHz, DMSO-d_6) δ [ppm] = 4.81 (s, 1H), 2.99 (m, 2H), 2.56 (m, 2H), 1.38 (m, 4H), 1.32 (s, 9H), 1.11 (s, 2H).

***Tert*-butyl (4-aminophenyl)carbamate**

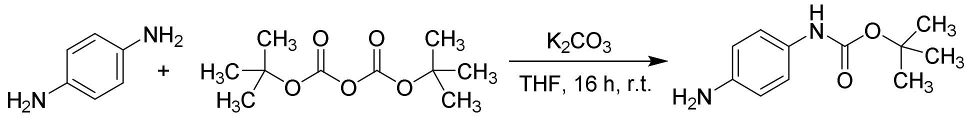

p-Aminoaniline (9.72 g, 90 mmol, 1 eq.) and K_2CO_3 (4.56 g, 33 mmol, 0.33 eq.) were dissolved in 100 mL THF and a solution of di-*tert*-butyl dicarbonate (6.54 g, 30 mmol, 1 eq.) in 30 mL THF was added dropwise over the course of 1.5 h. The reaction mixture was stirred at room temperature for 16 h. The solution was diluted with 200 mL demineralized H_2O and extracted with 4 x 100 mL CH_2Cl_2. The combined organic phase was washed with 2 x 100 mL brine, dried over Na_2SO_4, and the solvent was removed under reduced pressure. The crude product was purified by column chromatography (petroleum ether/EtOAc 2:1 v/v) yielding the product as yellow solid. Yield: 5.75 g (27.6 mmol, 92%). $C_{11}H_{16}N_2O_2$ (208.26 g/mol). ^1H NMR (499 MHz, DMSO-d_6) δ [ppm] = 8.76 (s, 1H, H6), 7.06 (m, 2H, H4), 6.46 (d, *J* = 7.6 Hz, 2H, H3), 4.71 (s, 2H, H1), 1.44 (s, 9H, H9). ^{13}C NMR (499 MHz, DMSO-d_6) δ [ppm] = 144.3 (C5), 129.6 (C2), 120.7 (C4), 114.4 (C2), 79.7 (C8), 28.6 (C9).

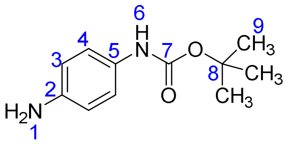

1.2.2.2 Synthesis of isothiocyanates – general description

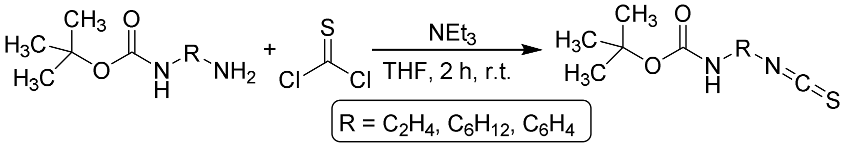

The reaction was performed under inert conditions. The *boc*-protected amine (1 eq.) and Et_3N (3 eq.) were dissolved in 200 mL THF and thiophosgene (85%, 1.4 eq.) was slowly added at 0 °C. The reaction mixture was stirred for 2 h at room temperature, which led to the formation of a brown suspension. The reaction was quenched by adding 200 mL H_2O and the product was extracted with 3 x 150 mL Et_2O. The organic phase was dried over Na_2SO_4, and the solvent was removed under reduced pressure, yielding the isothiocyanates.

***Tert*-butyl(2-isothiocyanatoethyl)carbamate** from 6 g (37.4 mmol) *tert*-butyl-*N*-(2-aminoethyl) carbamate, 4.6 mL (52.4 mmol) thiophosgene, 15.6 mL (112.3 mmol) triethylamine. The product was obtained as brown oil. Yield: 8.19 g (40.5 mmol, quant.). $C_8H_{14}N_2O_2S$ (202.27 g/mol). ^1H NMR (300 MHz, $CDCl_3$) δ [ppm] = 4.94 (s, 1H, H2), 3.63 (t, *J* = 5.5 Hz, 2H, H4), 3.36 (q, *J* = 5.7 Hz, 2H, H3), 1.44 (s, 9H, H1).

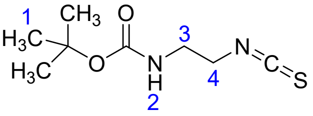

***Tert*-butyl(6-isothiocyanatohexyl)carbamate** from 3.55 g (16.4 mmol) *tert*-butyl-*N*-(6-aminohexyl) carbamate, 1.8 mL (23 mmol) thiophosgene, 6.8 mL (49 mmol) triethylamine. The product was obtained as brown oil. Yield: 4.26 g (16.4 mmol, quant.). $C_{12}H_{22}N_2O_2S$ (258.38 g/mol). ^1H NMR (300 MHz, $CDCl_3$) δ [ppm] = 4.64 (s, 1H, H7), 3.52 (t, *J* = 6.6 Hz, 2H, H1), 3.10 (q, *J* = 6.4 Hz, 2H, H6), 1.71 (m, 2H, H2), 1.50 (m, 2H, H5), 1.44 (s, 9H, H8), 1.35 (m, 4H, H3, H4).

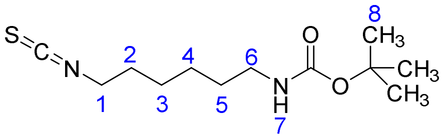

***Tert*-butyl(4-isothiocyanatophenyl)carbamate** from 3 g (14.4 mmol) *tert*-butyl(4-aminophenyl) carbamate, 1.4 mL (15.8 mmol) thiophosgene, 6 mL (43.2 mmol) triethylamine. The product was obtained as brown solid. Yield: 3.72 g (18.4 mmol, quant.). $C_{12}H_{14}N_2O_2S$ (250.32 g/mol). ^1H NMR (499 MHz, DMSO-d_6) δ [ppm] = 7.35 (d, *J* = 8.5 Hz, 2H, H4), 7.14 (m, 2H, H3), 6.69 (s, 1H, H6), 1.50 (s, 9H, H9). ^{13}C NMR (499 MHz, DMSO-d_6) δ [ppm] = 137.9 (C2), 126.3 (C3), 119.3 (C5), 119.2 (C4), 81.9 (C8), 28.2 (C9).

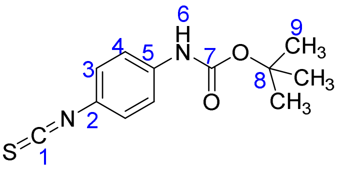

1.2.2.3 Synthesis of thiosemicarbazides – general description

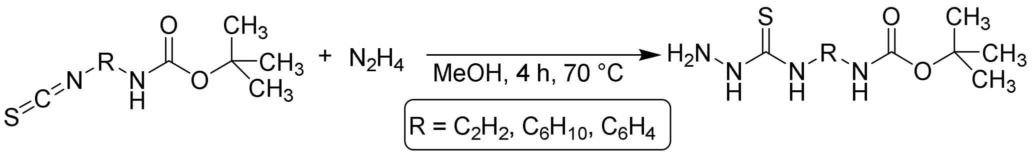

Hydrazine monohydrate (1.8 eq.) was dissolved in 50 mL MeOH and heated to 70 °C. The isothiocyanate (1 eq.) was dissolved in 100 mL MeOH and slowly added to the hydrazine solution over

the course of 1 h. The reaction mixture was stirred for further 3 h, then the solvent was removed under reduced pressure, yielding brown oil. The crude product was dissolved in 10 mL $CHCl_3$ and poured into boiling petrol ether. A precipitate formed, which was separated by decantation or filtration and the obtained product was dried under ambient conditions.

4-(2-*Tert*-butoxycarbonylamino-ethyl)thiosemicarbazide from 7.56 g (37.4 mmol) *tert*-butyl(2-isothiocyanatoethyl)carbamate, 3.3 mL (67.3 mmol) hydrazine hydrate. The product was obtained as brown solid. Yield: 8.03 g (36.8 mmol, 98%). $C_8H_{18}N_4O_2S$ (234.32 g/mol). ^{1}H NMR (300 MHz, DMSO-*d*$_6$) δ [ppm] = 8.67 (s, 1H, H2), 7.94 (s, 1H, H3), 6.83 (s, 1H, H6), 4.44 (s, 2H, H1), 3.48 (q, *J* = 5.9 Hz, 2H, H4/H5), 3.05 (q, *J* = 5.9 Hz, 2H, H4/H5), 1.37 (s, 9H, H7). ^{13}C NMR (300 MHz, DMSO-*d*$_6$) δ [ppm] = 181.8, 156.2, 78.2, 43.2, 40.4, 28.7. HR-ESI-MS: m/z $[M+Na]^+$ (calc.) = 257.1043, m/z $[M+Na]^+$ = 257.1044.

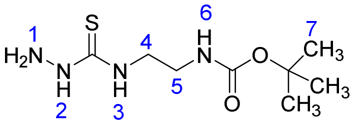

4-(6-*Tert*-butoxycarbonylamino-hexyl)thiosemicarbazide from 4.26 g (16.4 mmol) *tert*-butyl(6-isothiocyanatohexyl)carbamate, 2.2 mL (29.5 mmol) hydrazine hydrate. The product was obtained as brown solid. Yield: 4.46 g (15.4 mmol, 94%). $C_{12}H_{26}N_4O_2S$ (290.43 g/mol). ^{1}H NMR (300 MHz, DMSO-*d*$_6$) δ [ppm] = 8.51 (s, 1H, H2), 7.78 (s, 1H, H3), 6.74 (s, 1H, H10), 4.43 (s, 2H, H1), 2.86 (q, *J* = 6.1 Hz, 2H, H4), 3.39 (q, *J* = 6.1 Hz, 2H, H9), 1.47 (m, 2H, H5), 1.36 (s, 9H, H8, H11), 1.23 (m, 4H, H6, H7). ^{13}C NMR (300 MHz, DMSO-*d*$_6$) δ [ppm] = 181.6, 156.0, 77.7, 40.2, 43.3, 39.7, 29.9, 29.6, 28.7, 26.5. HR-ESI-MS: m/z $[M+H]^+$ (calc.) = 291.1849, m/z $[M+Na]^+$ (calc.) = 313.1669, m/z $[M+H]^+$ = 291.1851, m/z $[M+Na]^+$ = 313.1671.

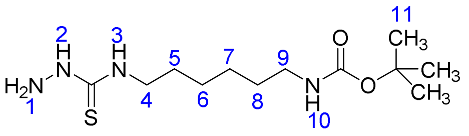

4-(4-*Tert*-butoxycarbonylamino-phenyl)thiosemicarbazide from 3.72 g (14.8 mmol) *tert*-butyl(4-isothiocyanatophenyl)carbamate, 1.1 mL (23.7 mmol) hydrazine hydrate. The product was obtained as brown solid. Yield: 3.78 g (13.4 mmol, 91%). $C_{12}H_{18}N_4O_2S$ (282.36 g/mol). ^{1}H NMR (499 MHz, DMSO-*d*$_6$) δ [ppm] = 9.53 (s, 1H, H2), 9.27 (s, 1H, H4), 9.01 (s, 1H, H9), 7.43 (d, *J* = 8.5 Hz, 2H, H7), 7.36 (d, *J* = 7.8 Hz, 2H, H6), 4.73 (s, 2H, H1), 1.47 (s, 9H, H12). ^{13}C NMR (499 MHz, DMSO-*d*$_6$) δ [ppm] = 153.3 (C10), 136.4 (C8), 134.0 (C5), 124.7 (C6), 118.3 (C7), 79.4 (C11), 28.6 (C12). HR-ESI-MS: m/z $[M+H]^+$ (calc.) = 283.1223, m/z $[M+Na]^+$ (calc.) = 305.1043, m/z $[M+H]^+$ = 283.1218, m/z $[M+Na]^+$ = 305.1038.

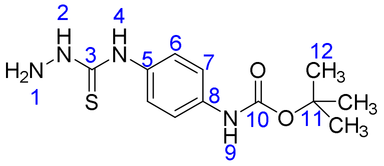

1.2.2.4 Synthesis of thiosemicarbazones – general description

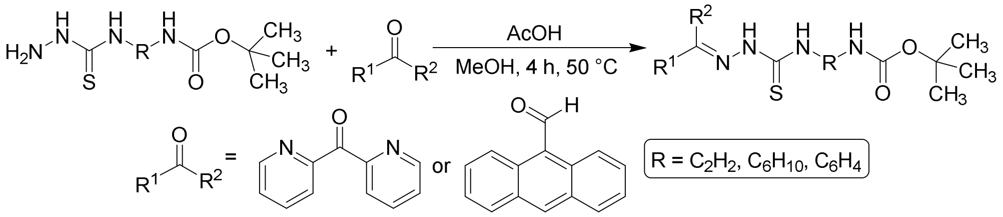

The thiosemicarbazide (1 eq.) and antracene-9-carbaldehyde or di(2-pyridyl) ketone (1 eq.) were dissolved in a minimum amount of MeOH. 0.1 mL glacial AcOH was added, and the reaction mixture was sonicated at 50 °C for 4 h. In case a precipitate was formed during the reaction, the product was filtered off, washed with cold MeOH and dried under ambient conditions. If the product did not precipitate, the solvent was removed under reduced pressure and the crude product was purified by column chromatography.

9-Anthraldehyde-4-(2-*tert*-butoxycarbonylamino-ethyl)-3-thiosemicarbazone form 0.2 g (0.8 mmol) 4-(2-*tert*-butoxycarbonylamino-ethyl)thiosemicarbazide, 0.18 g (0.8 mmol) antracene-9-carbaldehyde. The product precipitated as orange solid. Yield: 0.12 g (0.3 mmol, 38%). $C_{23}H_{26}N_4O_2S$ (422.55 g/mol). ^{1}H NMR (499 MHz, DMSO-*d*$_6$) δ [ppm] = 11.76 (s, 1H, H10), 9.29 (s, 1H, H9), 8.71 (s, 1H, H1), 8.52 (d, *J* = 8.4 Hz, 2H, H6), 8.35 (m, 1H, H12), 8.14 (d, *J* = 6.9 Hz, 2H, H3), 7.65 (t, *J* = 7.0 Hz, 2H, H5), 7.58 (t, *J* = 7.8 Hz, 2H, H4), 6.89 (m,

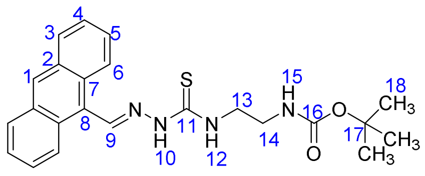

1H, H15), 3.65 (q, *J* = 5.7 Hz, 2H, H13), 3.18 (q, *J* = 5.1 Hz, 2H, H14), 1.28 (s, 9H, H18). ^{13}C NMR (499 MHz, DMSO-*d*$_6$) δ [ppm] = 176.7 (C11), 156.0 (C16), 142.1 (C9), 131.3 (C2), 130.1 (C7), 129.7 (C1), 129.3 (C3), 127.6 (C5), 126.0 (C4), 125.8 (C8), 124.2 (C6), 78.8 (C17), 43.9 (C13), 39.7 (C14), 28.5 (C18). HR-ESI-MS: m/z [M+H]$^+$ (calc.) = 423.1849, m/z [M+Na]$^+$ (calc.) = 445.1669, m/z [M+H]$^+$ = 423.1854, m/z [M+Na]$^+$ = 445.1673.

9-Anthraldehyde-4-(6-*tert*-butoxycarbonylamino-hexyl)-3-thiosemicarbazone from 0.3 g (10 mmol) 4-(6-*tert*-butoxycarbonylamino-hexyl) thiosemicarbazide, 0.21 g (10 mmol) antracene-9-carbaldehyde. The product precipitated as yellow solid. Yield: 0.31 g (6.5 mmol, 65%). $C_{27}H_{34}N_4O_2S$ (478.66 g/mol). ^{1}H NMR (499 MHz, DMSO-*d*$_6$) δ [ppm] = 11.66 (s, 1H, H10), 9.28 (s, 1H, H9), 8.70 (s, 1H, H1), 8.50 (d, *J* = 8.16 Hz, 2H, H6), 8.31 (m, 1H, H12), 8.14 (d, *J* = 8.3 Hz, 2H, H3), 7.62 (t, *J* = 6.7 Hz, 2H, H5), 7.57 (t, *J* = 7.7 Hz, 2H, H4), 6.76 (m, 1H, H19), 3.57 (q, *J* = 6.7 Hz, 2H, H13), 2.88 (q, *J* = 6.7 Hz, 2H, H18), 1.57 (m, 2H, H14), 1.36 (s, 11H, H17, H22), 1.27 (m, 4H, H15, H16). ^{13}C NMR (499 MHz, DMSO-*d*$_6$) δ [ppm] = 177.9 (C11), 155.6 (C20), 141.7 (C9), 131.3 (C2), 130.1 (C7), 129.6 (C1), 129.3 (C3), 127.6 (C5), 126.0 (C4), 125.9 (C8), 125.2 (C6), 77.8 (C21), 43.9 (C13), 40.3 (C18), 30.1 (C17), 29.1 (C14), 28.7 (C21), 26.6 (C15, C16). HR-ESI-MS: m/z [M+H]$^+$ (calc.) = 479.2480, m/z [M+Na]$^+$ (calc.) = 501.2301, m/z [M+H]$^+$ = 479.2480, m/z [M+Na]$^+$ = 501.2301.

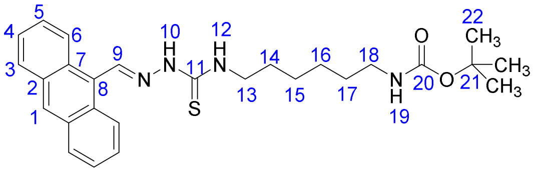

9-Anthraldehyde-4-(4-*tert*-butoxycarbonylamino-phenyl)-3-thiosemicarbazone from 0.3 g (1.1 mmol) 4-(4-*tert*-butoxycarbonylamino-phenyl) thiosemicarbazide, 0.22 g, (1.1 mmol) antracene-9-carbaldehyde. The product precipitated as yellow solid. Yield: 0.39 g (0.8 mmol, 75%). $C_{27}H_{26}N_4O_2S$ (470.59 g/mol). ^{1}H NMR (499 MHz, DMSO-*d*$_6$) δ [ppm] = 11.98 (s, 1H, H10), 9.89 (s, 1H, H12), 9.39 (s, 1H, H17), 9.34 (s, 1H, H9), 8.73 (s, 1H, H1), 8.58 (d, *J* = 9.0 Hz, 2H, H6), 8.15 (d, *J* = 9.0 Hz, 2H, H3), 7.66 (t, *J* = 8.0 Hz, 2H, H5), 7.59 (t, *J* = 7.2 Hz, 2H, H4), 7.41 (m, 4H, H14, H15), 1.48 (s, 9H, H20). ^{13}C NMR (499 MHz, DMSO-*d*$_6$) δ [ppm] = 176.6 (C11), 153.3 (C18), 142.5 (C9), 137.2 (C13/C16), 133.7 (C13/C16), 131.3 (C2), 130.2 (C7), 130.0 (C1), 129.2 (C3), 127.7 (C5), 126.4 (C14/C15), 126.1 (C4), 125.7 (C8), 125.2 (C6), 118.1 (C14/C15), 78.9 (C19), 28.3 (C20). HR-ESI-MS: m/z [M+H]$^+$ (calc.) = 471.1849, m/z [M+Na]$^+$ (calc.) = 493.1669, m/z [M+H]$^+$ = 471.1853, m/z [M+Na]$^+$ = 493.1674.

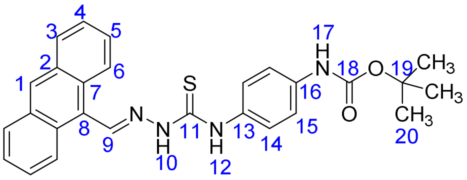

Di-2-pyridylketone-4-(2-*tert*-butoxycarbonylamino-ethyl)-3-thiosemicarbazone from 0.2 g (0.8 mmol) 4-(2-*tert*-butoxycarbonylamino-ethyl) thiosemicarbazide, 0.16 g (0.8 mmol) di-pyridyl ketone. The product was purified by column chromatography (c-hexane/EtOAc 1:3 v/v) and obtained as pale orange solid. Yield: 0.17 g (0.4 mmol, 50%). $C_{19}H_{24}N_6O_2S$ (400.50 g/mol). ^{1}H NMR (499 MHz, DMSO-*d*$_6$) δ [ppm] = 13.23 (s, 1H, H12), 8.91 (m, 1H, H14), 8.82 (d, *J* = 4.6 Hz, 1H, H1), 8.58 (d, *J* = 4.5 Hz, 1H, H11), 8.26 (d, *J* = 8.0 Hz, 1H, H8), 8.00–7.93 (m, 2H, H3, H9), 7.56 (m, 1H, H2), 7.51–7.46 (m, 2H, H4, H10), 7.04 (m, 1H, H17), 3.64 (m, 2H, H15), 3.21 (m, 2H, H16), 1.34 (s, 9H, H20). ^{13}C NMR (499 MHz, DMSO-*d*$_6$) δ [ppm] = 177.6 (C13), 155.6 (C18), 155.4 (C7), 151.7 (C5), 148.8 (C11), 148.6 (C1), 141.6 (C6), 138.0 (C3/C9), 137.4 (C3/C9), 127.6 (C4/C10), 125.1 (C2), 124.3 (C4/C10), 124.2 (C8), 79.0 (C19), 45.1 (C15), 39.4 (C16), 28.6 (C20). HR-ESI-MS: m/z [M+H]$^+$ (calc.) = 401.1754, m/z [M+Na]$^+$ (calc.) = 423.1574, m/z [M+H]$^+$ = 401.1758, m/z [M+Na]$^+$ = 423.1577.

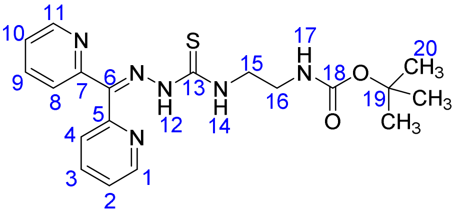

Di-2-pyridylketone-4-(6-*tert*-butoxycarbonylamino-hexyl)-3-thiosemicarbazone from 0.3 g (1 mmol) 4-(6-*tert*-butoxycarbonylamino-hexyl)thiosemicarbazide, 0.18 g (1 mmol) di-pyridyl ketone. The product was purified by column chromatography (CH_2Cl_2/MeOH 95:5 v/v) and obtained as orange oil. Yield: 0.4 g (0.9 mmol, 90%). $C_{23}H_{32}N_6O_2S$ (456.61 g/mol). ^{1}H NMR (499 MHz, DMSO-*d*$_6$) δ [ppm] =

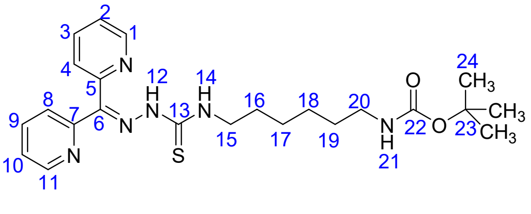

13.14 (s, 1H, H12), 8.88 (t, *J* = 5.8 Hz, 1H, H14), 8.82 (d, *J* = 7.9 Hz, 1H, H1), 8.57 (d, *J* = 4.1 Hz, 1H, H11), 8.23 (d, *J* = 7.9 Hz, 1H, H8), 7.98 (m, 2H, H3, H9), 7.57 (m, 1H, H2), 7.49 (m, 2H, H4, H10), 6.75 (m, 1H, H21), 3.59 (q, *J* = 6.5 Hz, 2H, H15), 2.90 (q, *J* = 6.2 Hz, 2H, H20), 1.60 (m, 2H, H16), 1.36 (s, 11H, H19, H24), 1.28 (m, 4H, H16, H17). ^{13}C NMR (499 MHz, DMSO-d_6) δ [ppm] = 177.5 (C13), 156.1 (C22), 155.6 (C7), 151.4 (C5), 148.8 (C11), 148.6 (C1), 141.5 (C6), 137.9 (C3/C9), 137.5 (C3/C9), 127.6 (C4/C10), 125.1 (C2), 124.3 (C4/C10), 124.2 (C8), 77.2 (C23), 44.3 (C15), 40.2 (C20), 29.9 (C19), 29.1 (C16), 28.7 (C24), 26.5 (C17, C18). HR-ESI-MS: m/z $[M+H]^+$ (calc.) = 457.2380, m/z $[M+Na]^+$ (calc.) = 479.2200, m/z $[M+H]^+$ = 457.2384, m/z $[M+Na]^+$ = 479.2201.

Di-2-pyridylketone-4-(4-*tert*-butoxycarbonylamino-phenyl)-3-thiosemicarbazone from 0.3 g (1 mmol) 4-(4-*tert*-butoxycarbonylamino-phenyl) thiosemicarbazide, 0.19 g (1 mmol) di-pyridyl ketone. The product was purified by column chromatography (*c*-hexane/EtOAc 1:3 v/v) and obtained as colorless solid. Yield: 0.38 g (0.8 mmol, 80%). $C_{23}H_{24}N_6O_2S$ (448.55 g/mol). ^{1}H NMR (499 MHz, DMSO-d_6) δ [ppm] = 13.45 (s, 1H, H12), 10.34 (s, 1H, H14), 9.40 (s, 1H, H19), 8.84 (m, 1H, H1), 8.58 (m, 1H, H11), 8.40 (m, 1H, H8), 8.01 (m, 1H, H3), 7.95 (m, 1H, H9), 7.60 (m, 1H, H2), 7.54 (m, 1H, H4), 7.47 (H10, H16/H17), 7.41 (H16/H17), 1.48 (s, 9H, H22). ^{13}C NMR (499 MHz, DMSO-d_6) δ [ppm] = 178.5 (C13), 155.4 (C7), 153.4 (C20), 151.4 (C5), 148.8 (C11), 148.6 (C1), 142.2 (C6), 138.0 (C3), 137.5 (C15/C18), 137.5 (C9), 133.4 (C15/C18), 127.7 (C4), 126.9 (C16/C17), 125.2 (C2), 124.6 (C8), 124.4 (C10), 118.2 (C16/C17), 78.6 (C21), 28.7 (C22). HR-ESI-MS: m/z $[M+H]^+$ (calc.) = 449.1754, m/z $[M+Na]^+$ (calc.) = 471.1574, m/z $[M+H]^+$ = 449.1757, m/z $[M+Na]^+$ = 471.1575.

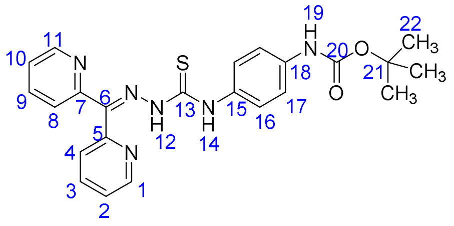

1.2.2.4 De-protection of the TSC-phenyl-Boc conjugate. Synthesis of **(4-aminophenylamino)[bis(pyridin-2-yl)methylideneamino]carbothioamide**. The thiosemicarbazone (0.1 g, 0.22 mmol, 1 eq.) was dissolved in 50 mL MeOH and a solution of 0.42 mL HCl conc. (5.1 mol, 23 eq.) in 20 mL MeOH was slowly added at 0 °C. The reaction mixture was stirred for 16 h at room temperature. The solvent was removed under reduced pressure and the residue was re-dissolved in 40 mL EtOAc. The organic phase was washed with 3 x 40 mL sat. $NaHCO_3$ solution and dried over $MgSO_4$. After that, the solvent was removed under reduced pressure yielding the product as a yellow solid. Yield: 0.07 g, (0.2 mmol, 93%). $C_{18}H_{16}N_6S$ (348.43 g/mol). ^{1}H NMR (499 MHz, DMSO-d_6) δ [ppm] = 13.38 (s, 1H), 11.06 (s, 1H), 8.82 (d, *J* = 4.5 Hz, 1H), 8.29 (d, J = 5.0 Hz, 1H), 8.25 (m, 2H), 8.14 (m, 1H), 7.77 (m, 4H), 7.40 (d, *J* = 8.7 Hz, 2H), 5.29 (s, 2H).

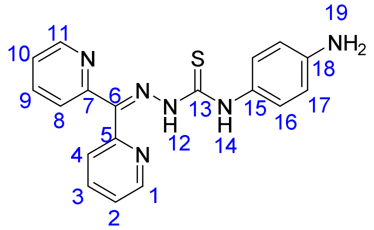

1.2.3 Thiosemicarbazones derived from acetobromo-α-*D*-glucose

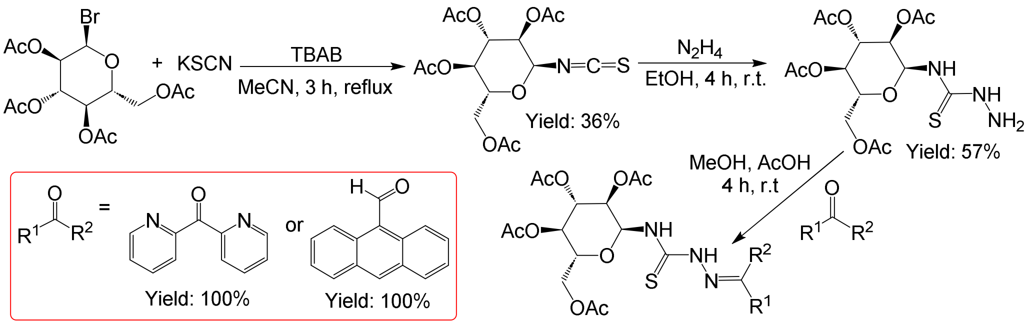

Scheme S3. Overview about the synthesis of TSCs derived from glucose.

1.2.3.1 Synthesis of β-*D*-Glucopyranosyl isothiocyanate

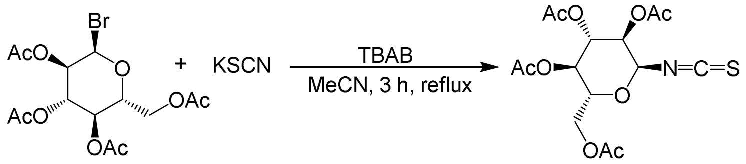

KSCN (0.4 g, 4 mmol, 2 eq.) and TBAB (0.64 g, 2 mmol, 1 eq.) were dissolved in 200 mL dry MeCN and 3 g molecular sieves (4 Å) were added. The reaction mixture was stirred for 3 h. Then, acetobromo-*α*-*D*-glucose (0.85 g, 2 mmol, 1 eq.) was added, and the reaction was refluxed for further 3 h. The reaction mixture was filtrated, and the solvent was removed under reduced pressure. The crude product was purified by column chromatography (*c*-hexane/EtOAc 3:2). The product was obtained as colorless solid. Yield: 0.28 g (0.7 mmol, 36%). $C_{15}H_{19}NO_9S$ (389.38 g/mol). ^1H NMR (499 MHz, DMSO-*d*$_6$) δ [ppm] = 5.26–5.02 (m, 4H), 4.29–4.13 (m, 2H), 3.75 (m, 1H), 2.12 (s, 6H), 2.03 (d, *J* = 3.9 Hz, 6H).

1.2.3.2 Synthesis of *N*-(2,3,4,6-Tetra-*O*-acetyl-β-*D*-glucopyranosyl)hydrazinecarbothioamide

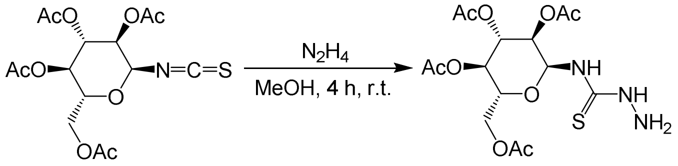

Hydrazine monohydrate (0.035 mL, 0.7 mmol) was dissolved in 20 mL EtOH. β-*D*-glucopyranosyl isothiocyanate (0.28 g, 0.7 mmol) was dissolved in 20 mL EtOH and slowly added to the hydrazine solution over the course of 1 h. The reaction mixture was stirred for further 3 h. The formed precipitate was filtered off and washed with cold EtOH. The product was obtained as colorless solid. Yield: 0.16 g (0.4 mmol, 57%). $C_{15}H_{23}N_3O_9S$ (421.42 g/mol). ^1H NMR (499 MHz, DMSO-*d*$_6$) δ [ppm] = 9.27 (s, 1H), 8.18 (s, 1H), 5.82 (m, 1H), 5.37 (t, *J* = 9.5, 1H), 5.09 (t, *J* = 9.4 Hz, 1H), 4.92 (t, *J* = 9.8 Hz, 1H), 4.61 (s, 2H), 4.20-4.11 (m, 1H), 4.02–3.93 (m, 2H), 2.03–1.92 (m, 12H).

1.2.3.3 Synthesis of thiosemicarbazones – general description

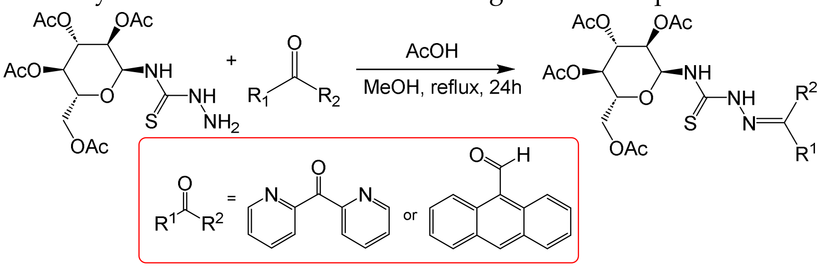

9-anthracene aldehyde or di(2-pyridyl) ketone (1 eq.) and *N*-(2,3,4,6-tetra-*O*-acetyl-β-*D*-glucopyranosyl)hydrazinecarbothioamide (1 eq.) were dissolved in 10 mL MeOH and 0.1 mL glacial acetic acid were added. The reaction mixture was refluxed for 24 h. The solvent was removed under reduced pressure, yielding the product in quantitative yields.

9-Anthraldehyde-4-(2,3,4,6-tetra-*O*-acetyl-β-*D*-glucopyranosyl)-3-thiosemicarbazone from 0.07 g (0.17 mmol) *N*-(2,3,4,6-tetra-*O*-acetyl-β-*D*-gluco-pyranosyl) hydrazine carbothioamide, 0.035 g (0.17 mmol) 9-antracene aldehyde. The product was obtained as colorless solid. Yield: 0.105 g (0.17 mmol, quant.). $C_{30}H_{31}N_3O_9S$ (609.65 g/mol). ^1H NMR (499 MHz, DMSO-*d*$_6$) δ [ppm] = 12.16 (s, 1H, H10), 9.38 (s, 1H), 8.75 (s, 1H, H9), 8.75 (s, 1H, H1), 8.54 (t, *J* = 9.1 Hz, 3H, H6, H12), 8.17 (d, *J* = 8.3 Hz, 2H, H3), 7.69 (m, 2H, H5), 7.60 (m, 2H, H4), 5.98 (m, 1H, H13), 5.45 (t, *J* = 9.5 Hz, 1H, H15), 5.26 (t, *J* = 9.3 Hz, 1H, H14), 4.92 (t, *J* = 9.7 Hz, 1H, H16), 4.18 (m, 1H, H18), 4.10 (m, 1H, H17), 3.99 (m, 1H, H18), 2.04 (s, 3H, H26), 2.00 (s, 3H, H22), 1.98 (s, 3H, H24), 1.97 (s, H25). ^{13}C NMR (499 MHz, DMSO-*d*$_6$) δ [ppm] = 179.3 (C11), 170.5 (C19), 170.1 (C25), 170.0 (C23), 169.8 (C21), 144.0 (C9), 131.3 (C2), 130.3 (C1), 130.2 (C7), 129.4 (C3), 128.2 (C5), 126.1 (C4), 124.9 (C8), 81.7 (C13), 73.0 (C15), 72.6 (C17), 71.1 (C14), 68.3 (C16), 62.1 (C18), 21.0

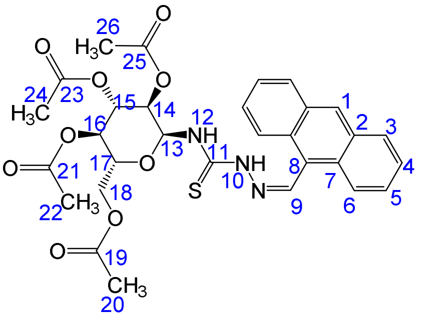

(C20/C22/C24/C26), 20.9 (C20/C22/C24/C26), 20.9 (C20/C22/C24/C26), 20.8 (C20/C22/C24/C26). HR-ESI-MS: m/z $[M+H]^+$ (calc.) = 610.1854, m/z $[M+Na]^+$ (calc.) = 632.1673, m/z $[M+H]^+$ = 610.1860, m/z $[M+Na]^+$ = 632.1680.

Di-2-pyridylketone-4-(2,3,4,6-tetra-*O*-acetyl-β-*D*-glucopyranosyl)-3-thiosemicarbazone from 0.07 g (0.17 mmol) *N*-(2,3,4,6-tetra-*O*-acetyl-β-*D*-gluco-pyranosyl) hydrazine carbothioamide, 0.031 g (0.17 mmol) di(2-pyridyl) ketone. The product was obtained as colorless solid. Yield: 0.1 g (0.17 mmol, quant.). $C_{26}H_{29}N_5O_9S$ (587.60 g/mol). ^1H NMR 499 MHz, DMSO-*d*$_6$) δ [ppm] = 13.61 (s, 1H, H12), 9.14 (d, *J* = 9.0, 1H, H14), 8.84 (d, *J* = 4.4 Hz, 1H, H1), 8.60 (d, *J* = 4.2 Hz, 1H, H11), 8.27 (d, *J* = 7.8 Hz, 1H, H8), 7.99 (m, 2H, H3, H9), 7.61 (m, 1H, H2), 7.53 (m, 2H, H4, H10), 5.98 (t, *J* = 9.1 Hz, 1H, H15), 5.47 (t, *J* = 9.5 Hz, 1H, H17), 5.38 (t, *J* = 8.9 Hz, 1H, H16), 4.98 (t, *J* = 9.7 Hz, 1H, H18), 4.24 (m, 1H, H20), 4.14 (m, 1H, H19), 4.01 (d, *J* = 11.3 Hz, 1H, H20), 2.01 (s, 3H, H22/H24/H26/H28), 1.98 (s, 3H, H22/H24/H26/H28), 1.95 (s, 3H, H22/H24/H26/H28), 1.92 (s, 3H, H22/H24/H26/H28). ^{13}C NMR (499 MHz, DMSO-*d*$_6$) δ [ppm] = 179.4 (C13), 170.5 (C21), 170.1 (C23), 170.0 (C25), 169.8 (C27), 155.2 (C7), 151.2 (C5), 148.9 (C11), 148.7 (C1), 143.1 (C6), 138.1 (C3/C9), 137.5 (C3/C9), 127.9 (C4/C10), 125.5 (C2), 124.6 (C4/C10), 124.6 (C8), 82.2 (C21), 73.0 (C17), 72.7 (C19), 71.3 (C16), 68.3 (C18), 62.1 (C20), 21.0 (C22/C24/C26/C28), 20.9 (C22/C24/C26/C28), 20.9 (C22/C24/C26/C28), 20.8 (C22/C24/C26/C28). HR-ESI-MS: m/z $[M+H]^+$ (calc.) = 588.1759, m/z $[M+Na]^+$ (calc.) = 610.1578, m/z $[M+H]^+$ = 588.1762, m/z $[M+Na]^+$ = 610.1582.

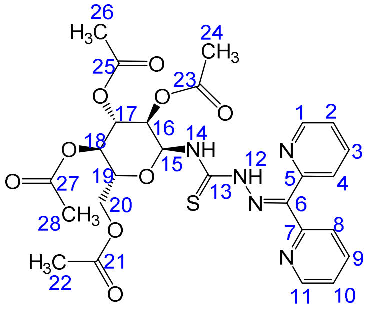

1.2.4 Syntheses of thiosemicarbazones derived from *trans*-4-hydroxycyclohexanol

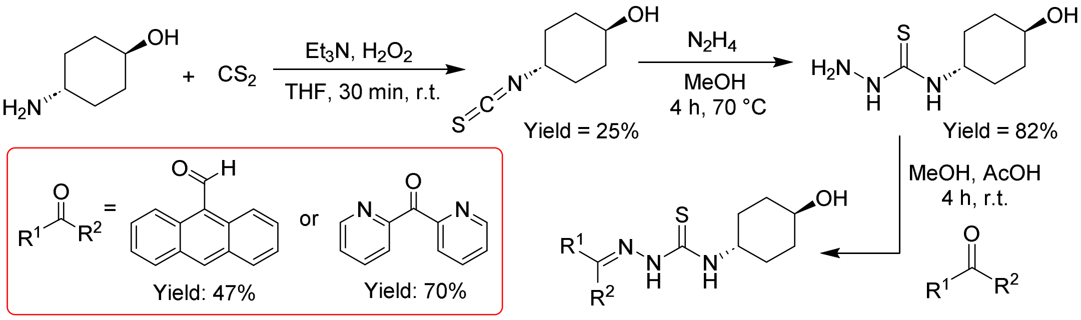

Scheme S4. Overview about the synthesis of TSCs derived from *trans*-4-hydroxycyclohexanol.

1.2.4.1 Synthesis of *trans*-4-hydroxycyclohexyl isothiocyanate

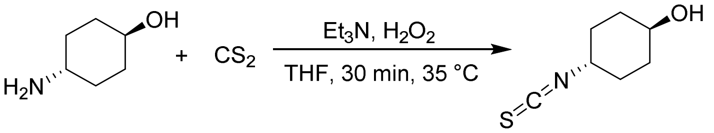

The reaction was performed under inert conditions. 4 g *trans*-4-hydroxycyclohexanol (34.7 mmol, 1 eq.) was dissolved in 35 mL THF and 10.5 mL CS_2 (173.5 mmol, 5 eq.) and 0.5 mL Et_3N (3.5 mmol, 0.1 eq.) were added dropwise. The mixture was stirred for 30 min at room temperature. Then, the reaction was cooled to 0 °C and 9.9 mL conc. H_2O_2 (97.2 mmol, 2.8 eq.) was slowly added. The solution was subsequently acidified with conc. HCl and concentrated under reduced pressure to 15 mL. The suspension was filtered off, and the filtrate was extracted with 3 x 50 mL EtOAc. The combined organic phase was dried over $MgSO_4$ and the solvent was removed under reduced pressure. The crude product was extracted with $CHCl_3$ and insoluble residues were filtered off. The product was obtained as yellow oil. Yield: 1.38 g (8.8 mmol, 25%). $C_7H_{11}NOS$ (157.23 g/mol). ^1H NMR (499 MHz, $CDCl_3$) δ [ppm] = 3.78 (m, 1H, H5), 3.67 (m, 1H, H2), 2.10 (m, 2H, H3), 1.96 (m, 3H, H4), 1.62 (m, 2H, H3), 1.41 (m, 2H, H4). ^{13}C NMR (499 MHz, DMSO-*d*$_6$) δ [ppm] = 130.3 (C1), 67.7 (C5), 54.6 (C2), 31.5 (C4), 29.7 (C3).

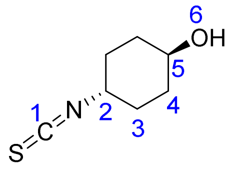

1.2.4.2 Synthesis of 4-(*trans*-4-hydroxycyclohexyl)-thiosemicarbazide

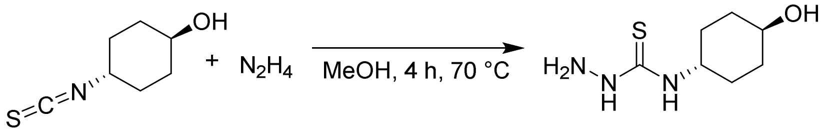

0.40 mL hydrazine monohydrate (8.7 mmol, 1 eq.) was dissolved in 20 mL MeOH and heated to 70 °C. 1.38 g *trans*-4-hydroxycyclohexyl isothiocyanate (8.7 mmol, 1 eq.) was dissolved in 50 mL MeOH and slowly added to the hydrazine solution over the course of 1 h. The reaction mixture was stirred for further 3 h, then the solvent was removed under reduced pressure, yielding a brown oil. The crude product was dissolved in 10 mL $CHCl_3$ and poured into boiling petrol ether. The formed precipitate was filtered off and dried under ambient conditions. The product was obtained as colorless solid. Yield: 1.35 g (7.1 mmol, 82%). $C_7H_{15}N_3OS$ (189.28 g/mol). ^{1}H NMR (499 MHz, DMSO-*d*$_6$) δ [ppm] = 8.55 (s, 1H, H2), 7.43 (d, *J* = 8.8 Hz, 1H, H4), 4.52 (s, 1H, H9), 4.43 (s, 2H, H1), 3.99 (m, 1H, H5), 3.37 (m, 2H, H8), 1.82 (m, 4H, H7/H6), 1.28 (m, 2H, H6), 1.17 (m, 2H, H7). ^{13}C NMR (499 MHz, DMSO-*d*$_6$) δ [ppm] = 180.6 (C3), 68.5 (C8), 51.7 (C5), 34.6 (C7), 30.6 (C6). HR-ESI-MS: m/z [M+H]$^+$ (calc.) = 190.1009, m/z [M+H]$^+$ = 190.1011.

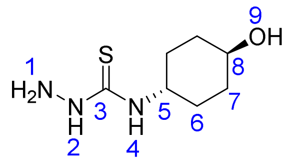

1.2.4.3 Synthesis of thiosemicarbazones – general description

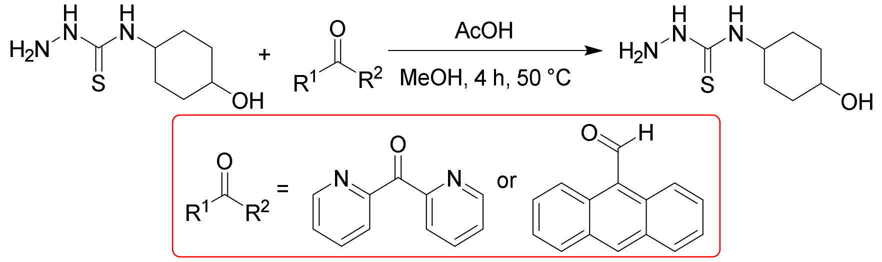

4-(*Trans*-4-hydroxycyclohexyl)-thiosemicarbazide (1 eq.) and antracene-9-carbaldehyde or di(2-pyridyl) ketone (1 eq.) were dissolved in a minimum amount MeOH. 0.1 mL glacial AcOH was added, and the reaction mixture was sonicated at 50 °C for 4 h. The solvent was removed under reduced pressure and the crude product was purified by column chromatography.

9-Anthraldehyde-4-(*trans*-4-hydroxycyclohexyl)-3-thiosemicarbazone from 0.3 g (1.6 mmol) 4-(*trans*-4-hydroxycyclohexyl)thiosemicarbazide, (0.32 g, 1.6 mmol) antracene-9-carbaldehyde. The product was purified by column chromatography (*c*-hexane/EtOAc 1:2 v/v) and obtained as yellow solid. Yield: 0.28 g (0.7 mmol, 47%). $C_{22}H_{23}N_3OS$ (377.51 g/mol). ^{1}H NMR (499 MHz, DMSO-*d*$_6$) δ [ppm] = 11.72 (s, 1H, H10), 9.28 (s, 1H, H9), 8.70 (s, 1H, H1), 8.47 (d, *J* = 9.1 Hz, 2H, H6), 8.14 (d, *J* = 8.3 Hz, 2H, H3), 7.83 (d, *J* = 8.3 Hz, 1H, H12), 7.62 (m, 2H, H5), 7.52 (m, 2H, H4), 4.55 (s, 1H, H17), 4.16 (m, 1H, H13), 3.37 (m, 1H, H16), 1.91 (m, 2H, H14), 1.80 (m, 2H, H15), 1.40 (m, 2H, H14), 1.25 (m, 2H, H15). ^{13}C NMR (499 MHz, DMSO-*d*$_6$) δ [ppm] = 176.6 (C11), 141.9 (C9), 131.3 (C2), 130.0 (C7), 129.7 (C1), 129.4 (C3), 127.6 (C4/C5), 125.9 (C4/C5), 125.9 (C8), 124.9 (C6), 68.4 (C16), 52.5 (C13), 34.4 (C15), 29.9 (C14). ESI-MS: m/z [M+H]$^+$ (calc.) = 378.1635, m/z [M+Na]$^+$ (calc.) = 400.1454, m/z [M+H]$^+$ = 378.1637, m/z [M+Na]$^+$ = 400.1457.

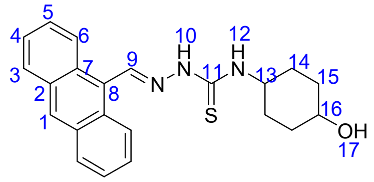

Di-2-pyridylketone-4-(*trans*-4-hydroxycyclohexyl)-3-thiosemicarbazone from 0.3 g (1.6 mmol) 4-(*trans*-4-hydroxycyclohexyl)thiosemicarbazide, 0.29 g (1.6 mmol) di(2-pyridyl) ketone. The product was purified by column chromatography (CH_2Cl_2/MeOH 95:5 v/v) and obtained as pale orange solid. Yield: 0.4 g (1.1 mmol, 70%). $C_{18}H_{21}N_5O_2S$ (355.46 g/mol). ^{1}H NMR (499 MHz, DMSO-*d*$_6$) δ [ppm] = 13.20 (s, 1H, H13), 8.81 (d, *J* = 4.3 Hz, 1H, H1), 8.56 (d, *J* = 5.1 Hz, 1H, H11), 8.33 (d, *J* = 9.4 Hz, 1H, H14), 8.17 (d, *J* = 8.3 Hz, 1H, H8), 7.96 (m, 2H, H3, H9), 7.58 (m, 1H, H2), 7.47 (m, 2H, H4, H10), 4.57 (s, 1H, H19),

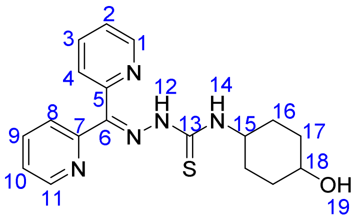

4.17 (d, J = 4.4 Hz, 1H, H15), 3.39 (m, 1H, H18), 1.87 (m, 4H, H16, H17), 1.52 (m, 2H, H16), 1.23 (m, 2H, H17). ^{13}C NMR (499 MHz, DMSO-d_6) δ [ppm] = 176.8 (C13), 155.5 (C7), 151.6 (C5), 148.8 (C11), 148.6 (C1), 141.8 (C6), 138.0 (C3/C9), 137.6 (C3/C9), 127.5 (C4/C10), 125.2 (C2), 124.3 (C4/C10, C8), 68.6 (C18), 53.4 (C15), 34.6 (C17), 29.9 (C16). HR-ESI-MS: m/z $[M+H]^+$ (calc.) = 356.1540, m/z $[M+Na]^+$ (calc.) = 378.1359, m/z $[M+H]^+$ = 356.1543, m/z $[M+Na]^+$ = 378.1361.

1.2.5 Thiosemicarbazones derived from dopamine

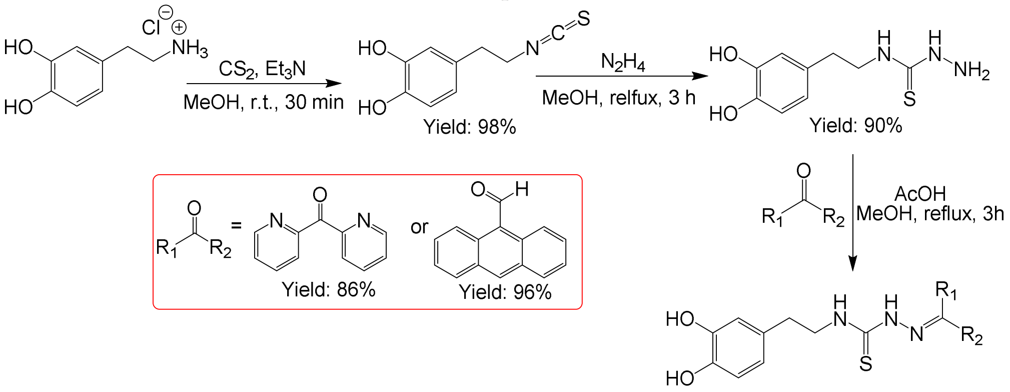

Scheme S5. Overview about the synthesis of TSCs derived from dopamine.

1.2.5.1 Synthesis of 4-(2-isothiocyanatoethyl)benzene-1,2-diol

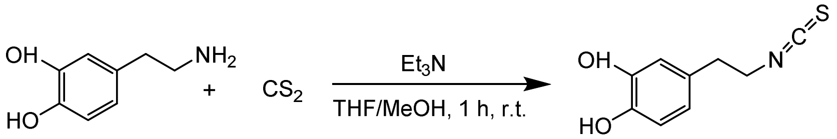

The reaction was performed under inert conditions. Dopamine hydrochloride (2 g, 10.5 mmol, 1 eq.) was suspended 26 mL THF and Et_3N (1.62 mL, 11.6 mmol, 1.1 eq.) was added under stirring. 21 mL of MeOH were slowly added to form a clear solution. Then, CS_2 (3.17 mL, 52.7 mmol, 5 eq.) was added, and the mixture was stirred for 1 h at room temperature. The reaction was cooled to 0 °C and H_2O_2 (30% in H_2O, 0.94 mL) was added dropwise. The mixture was acidified (pH = 2) using conc. HCl and the solution was concentrated under reduced pressure. The formed precipitate was filtered off and the filtrate was diluted with 15 mL H_2O. The filtrate was extracted with 3 x 50 mL EtOAc. The combined organic phase was dried over $MgSO_4$ and the solvent was removed under reduced pressure. The product was obtained as colorless solid. Yield: 1.13 g (5.8 mmol, 55%). $C_9H_9NO_2S$ (195.24 g/mol). ^{1}H NMR (499 MHz, DMSO-d_6) δ [ppm] = 6.82 (d, J = 8.1 Hz, 1H, H2), 6.74 (d, J = 2.0 Hz, 1H, H5), 6.65 (dd, J = 8.1 Hz, 2.0 Hz, 1H, H1), 5.74–4.65 (s, 2H, H10, H11), 3.66 (t, J = 6.8 Hz, 2H, H8), 2.86 (t, J = 6.8 Hz, 2H, H7), ^{13}C NMR (499 MHz, DMSO-d_6) δ [ppm] = 143.7 (C3), 142.6 (C4), 130.1 (C6), 121.5 (C1), 116.0 (C5), 115.7 (C2), 46.6 (C8), 35.8 (C7).

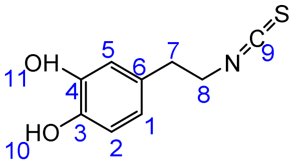

1.2.5.2 Synthesis of 4-[2-(hydrazidecarbothioamino)ethyl]benzene-1,2-diol

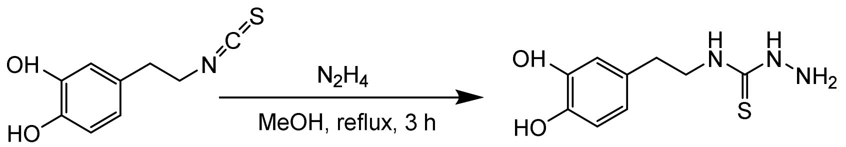

Hydrazine monohydrate (0.28 mL, 5.8 mmol) was dissolved in 50 mL MeOH and heated to 70 °C. 1.13 g 4-(2-isothiocyanatoethyl)benzene-1,2-diol (5.8 mmol) was dissolved in 50 mL MeOH and slowly added to the hydrazine solution over the course of 1 h. The reaction mixture was stirred for further 3 h and the solvent was removed under reduced pressure. The product was obtained as colorless solid. Yield: 1.18 g (5.2 mmol, 90%). $C_9H_{13}N_3O_2S$ (227.28 g/mol). ^{1}H NMR (499 MHz, DMSO-d_6) δ [ppm] = 8.59 (s, 1H, H11), 7.81 (s, 1H, H9), 6.66–6.59 (m, 2H, H2, H5), 6.47 (dd,

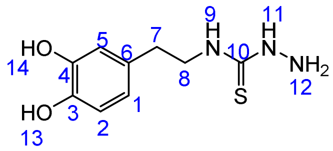

J = 8.20, 2.0 Hz, 1H, H1), 4.41 (s, 2H, H12), 3.58 (m, 3H, H8), 2.62 (m, 2H, H7). ^{13}C NMR (499 MHz, DMSO-*d*6) δ [ppm] = 145.6 (C4), 144.0 (C3), 130.6 (C6), 119.7 (C1), 116.4 (C2/C5), 116.0 (C2/C5), 45.2 (C8), 35.0 (C7). HR-ESI-MS: m/z [M+H]+ (calc.) = 228.0801, m/z [M+H]+ = 228.0800.

1.2.5.3 Synthesis of thiosemicarbazones

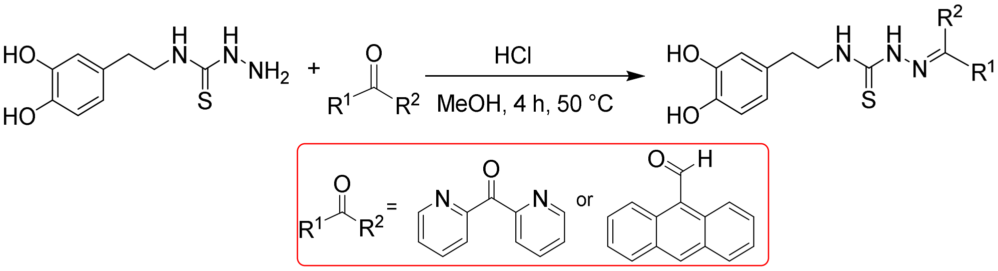

9-anthracene aldehyde or di(2-pyridyl) ketone (1 eq.) and 4-[2-(hydrazidecarbothioamino)ethyl] benzene-1,2-diol (1 eq.) were dissolved in a minimum amount of MeOH and a few droplets of conc. HCl were added. The reaction mixture was heated under reflux for 72 h. The solvent was removed under reduced pressure and the crude product was purified by column chromatography.

[Bis(pyridin-2-yl)methylideneamino][2-(3,4-dihydroxyphenyl)ethylamino] carbothioamide from 0.1 g (0.44 mmol) 4-[2-(hydrazidecarbothioamino)ethyl] benzene-1,2-diol, 0.08 g (0.44 mmol) dipyridyl ketone. The product was purified by column chromatography (*c*-hexane/EtOAc 1:2 v/v) and obtained as colorless solid. Yield: 0.16 g (0.4 mmol, 90%). $C_{20}H_{19}N_5O_2S$ (393.47 g/mol). ^{1}H NMR (499 MHz, DMSO-*d*6) δ [ppm] = 12.81 (s, 1H), 9.66 (t, *J* = 4.7 Hz, 1H), 8.91 (d, *J* = 4.5 Hz, 1H), 8.81 (d, *J* = 4.6 Hz, 1H), 8.34 (t, *J* = 8.6 Hz, 1H), 8.18–8.06 (m, 2H), 7.83 (m, 1H), 7.76–7.68 (m, 2H), 6.65 (m, 2H), 6.51 (dd, *J* = 8.0, 1.9 Hz, 1 H), 3.70 (m, 2H), 2.78 (m, 2H). HR-ESI-MS: m/z [M+H]+ (calc.) = 394.1332, m/z, [M+Na]+ (calc.) = 416.1152 m/z [M+H]+ = 394.1331 m/z, [M+Na]+ = 416.1151.

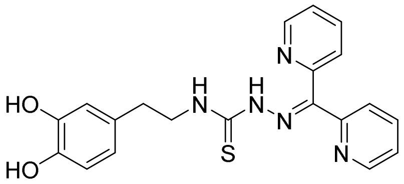

[(1*E*)-(anthracen-9-yl)methylideneamino][2-(3,4 dihydroxyphenyl)ethylamino] carbothioamide from 0.1 g (0.44 mmol) 4-[2-(hydrazidecarbothioamino)ethyl] benzene-1,2-diol, 0.09 g (0.44 mmol) dipyridyl ketone. The product was purified by column chromatography (*c*-hexane/EtOAc 6:1 v/v) and obtained as yellow solid. Yield: 0.16 g (0.38 mmol, 87%). $C_{24}H_{21}N_3O_2S$ (415.51 g/mol). ^{1}H NMR (499 MHz, DMSO-*d*6) δ [ppm] = 11.71 (s, 1H), 9.29 (s, 1H), 8.71 (s, 1H), 8.47 (d, *J* = 8.7 Hz, 2H), 8.27 (t, *J* = 5.7 Hz, 1H), 8.15 (d, *J* = 8.9 Hz, 2H), 7.61 (m, 4H), 6.65 (m, 2H), 6.50 (dd, *J* = 8.0, 2.0 Hz, 1H), 3.71 (q, *J* = 6.3 Hz, 2H), 2.73 (t, *J* = 7.5 Hz, 2H). HR-ESI-MS: m/z [M+H]+ (calc.) = 416.1427, m/z [M+Na]+ (calc.) = 438.1247, m/z [M+H]+ = 416.1433, m/z [M+Na]+ = 438.1251.

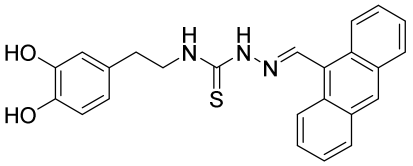

1.2.6 Synthesis of thiosemicarbazones derived from amino thiols

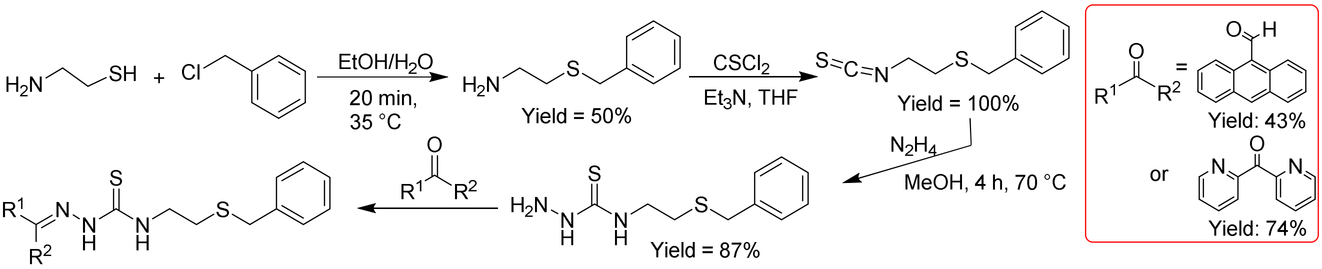

Scheme S6. Overview about the synthesis of TSCs derived from amino thiols.

1.2.6.1 Synthesis of 2-benzylsulfanyl ethylamine

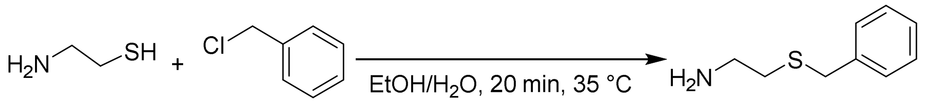

LiOH (0.49 g, 20.4 mmol, 2 eq.) was dissolved in a mixture of 10 mL demineralized H_2O and 30 mL EtOH and the resulting solution was transferred to a flask containing cysteamine hydrochloride (1.14 g, 10 mmol, 1 eq.). Then, 1.15 mL benzyl chloride (10 mmol, 1 eq.) was added dropwise to the solution and the reaction mixture was stirred for 20 min at 35 °C. Then, the EtOH was removed under reduced pressure and 40 mL demineralized H_2O was subsequently added. The aqueous solution was extracted with CH_2Cl_2 (3 x 60 mL), dried over anhydrous Na_2SO_4 and the solvent was removed under reduced pressure. The product was obtained as yellowish oil. Yield: 0.84 g (5 mmol, 50%). $C_9H_{13}NS$ (167.27 g/mol). ^{1}H NMR (499 MHz, $CDCl_3$) δ [ppm] = 7.23 (m, 4H, H6, H7) 7.14 (m, 1H, H8), 3.62 (s, 2H, H4), 2.73 (t, *J* = 6.5 Hz, 2H, H2), 2.43 (t, *J* = 6.3 Hz, 2H, H3), 1.40 (s, 2H, H1). ^{13}C NMR (499 MHz, DMSO-*d*$_6$) δ [ppm] = 138.1 (C5), 128.6 (C6, C7), 127.1 (C8), 41.0 (C2), 35.9 (C4), 35.4 (C3).

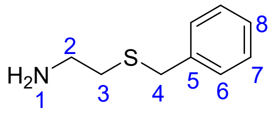

1.2.6.2 Synthesis of 2-benzylsulfanylethyl isothiocyanate

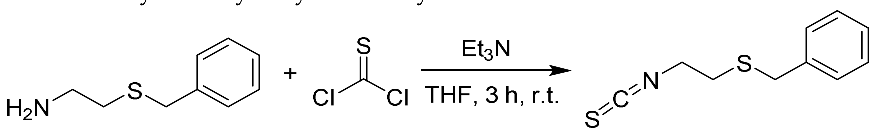

The reaction was performed under inert conditions. The protected cysteamine (0.8 g, 4.8 mmol, 1 eq.) and 2 mL Et_3N (14.3 mmol, 3 eq.) were dissolved in 30 mL THF. 0.46 mL thiophosgene (4.8 mmol, 1 eq.) was slowly added at 0 °C and the reaction mixture was stirred for 3 h at room temperature, which led to the formation of a brown suspension. The reaction was quenched by adding 100 mL demineralized H_2O and the product was extracted with 3 x 100 mL Et_2O. The organic phase was dried over Na_2SO_4 and the solvent was removed under reduced pressure, yielding the product as brown oil. Yield: 1 g (4.8 mmol, quant.). $C_{10}H_{11}NS_2$ (209.33 g/mol). ^{1}H NMR (499 MHz, $CDCl_3$) δ [ppm] = 7.33 (m, 4H, H6, H7), 7.28 (m, 1H, H8), 3.77 (s, 2H, H4), 3.54 (t, *J* = 7.5 Hz, 2H, H2), 2.69 (t, *J* = 7.5 Hz, 2H, H3). ^{13}C NMR (499 MHz, DMSO-*d*$_6$) δ [ppm] = 137.1 (C5), 132.6 (C1), 128.7 (C6, C7), 127.5 (C8), 44.5 (C2), 36.4 (C4), 30.8 (C3).

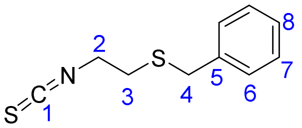

1.2.6.3 Synthesis of 4-(2-benzylsulfanylethyl)thiosemicarbazide

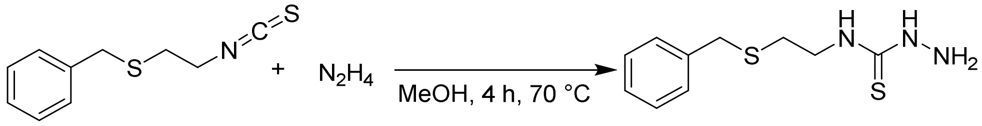

Hydrazine monohydrate (65% aqueous solution, 0.23 mL, 4.8 mmol, 1 eq.) was dissolved in 20 mL MeOH and heated to 70 °C. Then, the isothiocyanate (1 g, 4.8 mmol, 1 eq.) was dissolved in 50 mL MeOH and slowly added to the hydrazine solution over 1 h. The reaction mixture was stirred for further 3 h, then, the solvent was removed under reduced pressure, yielding a brown oil. The crude product was dissolved in 10 mL $CHCl_3$ and poured into boiling petrol ether. The oily precipitate was separated by decantation and dried under ambient conditions. The product was obtained as brown oil. Yield: 1.01 g (4.2 mmol, 87%). $C_{10}H_{15}N_3S_2$ (241.37 g/mol). ^{1}H NMR (499 MHz, DMSO-*d*$_6$) δ [ppm] = 8.69 (s, 1H, H2), 8.04 (s, 1H, H4), 7.35 (m, 2H, H9), 7.30 (m, 2H, H10), 7.23 (m, 1H, H11), 4.47 (s, 2H, H1), 3.77 (s, 2H, H7), 3.64 (q, *J* = 8.1 Hz, 2H, H5), 2.57 (t, *J* = 7.3 Hz, 2H, H6). ^{13}C NMR (499 MHz, DMSO-*d*$_6$) δ [ppm] = 181.4 (C3), 139.2 (C8), 129.4 (C9), 128.8 (C10), 127.2 (C11), 42.7 (C5), 35.2 (C7), 30.6 (C6). HR-ESI-MS: m/z $[M+H]^+$ (calc.) = 242.0780, m/z $[M+H]^+$ = 242.0781.

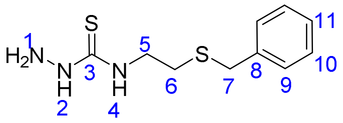

1.2.6.4 Synthesis of thiosemicarbazones – general description

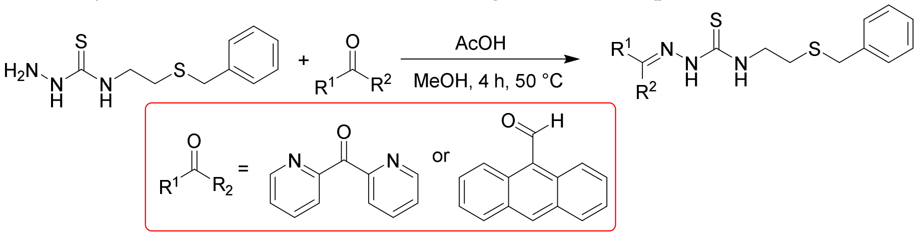

4-(2-Benzylsulfanylethyl)thiosemicarbazide (1 eq.) and antracene-9-carbaldehyde or di(2-pyridyl) ketone (1 eq.) were dissolved in a minimum amount of MeOH. Then, 0.1 mL glacial acetic acid were added, and the reaction mixture was sonicated at 50 °C for 4 h. Then, the solvent was removed under reduced pressure and the crude product was purified by column chromatography.

9-Anthraldehyde-4-(2-benzylsulfanylethyl)-3-thiosemicarbazone from 0.3 g (1.2 mmol) 4-(2-benzylsulfanylethyl)-thiosemicarbazide, 0.25 g (1.2 mmol) antracene-9-carbaldehyde. The product was purified by column chromatography (*c*-hexane/EtOAc 4:1 v/v) and obtained as yellow solid. Yield: 0.22 g (0.5 mmol, 43%). $C_{25}H_{23}N_3S_2$ (429.60 g/mol). ^{1}H NMR (499 MHz, DMSO-*d*$_6$) δ [ppm] = 11.80 (s, 1H, H10), 9.31 (s, 1H, H9), 8.71 (s, 1H, H1), 8.51 (m, 3H, H6, H12), 8.14 (d, *J* = 8.4 Hz, 2H, H3), 7.59 (m, 4H, H4, H5), 7.34 (d, *J* = 6.9 Hz, 2H, H17), 7.27 (m, 2H, H18), 7.21 (m, 1H, H19), 3.80 (m, 4H, H13, H15), 2.68 (t, *J* = 7.2 Hz, 2H, H14). ^{13}C NMR (499 MHz, DMSO-*d*$_6$) δ [ppm] = 177.6 (C11), 142.3 (C9), 139.1 (C16), 131.3 (C2), 130.1 (C7), 129.8 (C1), 129.4 (C3), 129.4 (C17), 128.8 (C18), 127.7 (C4/C5), 127.2 (C19), 126.1 (C4/C5), 125.8 (C8), 125.2 (C6), 43.3 (C13), 35.2 (C15), 30.3 (C14). HR-ESI-MS: m/z [M+H]$^+$ (calc.) = 430.1406, m/z [M+Na]$^+$ (calc.) = 452.1226, m/z [M+H]$^+$ = 430.1410, m/z [M+Na]$^+$ = 452.1229.

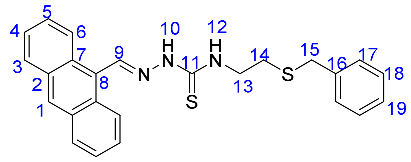

Di-2-pyridylketone-4-(2-benzylsulfanylethyl)-3-thiosemicarbazone from 0.3 g (1.2 mmol) 4-(2-benzylsulfanylethyl)thiosemicarbazide, 0.23 g (1.2 mmol) di(2-pyridyl) ketone. The product was purified by column chromatography (*c*-hexane/EtOAc 1:3 v/v) and obtained as colorless solid. Yield: 0.36 g (0.9 mmol, 74%). $C_{21}H_{21}N_5S_2$ (407.55 g/mol). ^{1}H NMR (499 MHz, DMSO-*d*$_6$) δ [ppm] = 13.32 (s, 1H, H12), 9.00 (t, *J* = 5.9 Hz, 1H, H14), 8.84 (d, *J* = 4.4 Hz, 1H, H1), 8.59 (d, *J* = 4.4 Hz, 1H, H11), 8.20 (d, *J* = 8.0 Hz, 1H, H8), 7.97 (m, 2H, H3, H9), 7.58 (m, 1H, H2), 7.49 (m, 2H, H4, H10), 7.36 (d, *J* = 7.3 Hz, 2H, H19), 7.30 (t, *J* = 6.5 Hz, 2H, H20), 7.23 (m, 1H, H21), 3.82 (m, 4H, H15, H17), 2.70 (t, *J* = 7.2 Hz, 2H, H16). ^{13}C NMR (499 MHz, DMSO-*d*$_6$) δ [ppm] = 177.9 (C13), 155.5 (C7), 151.5 (C5), 148.9 (C11), 148.6 (C1), 141.9 (C6), 139.1 (C18), 138.0 (C3/C9), 137.5 (C3/C9), 129.4 (C19), 128.8 (C20), 127.6 (C4/C10), 127.3 (C21), 125.2 (C2), 124.4 (C4/C10), 124.3 (C8), 43.9 (C15), 35.4 (C17), 30.0 (C16). HR-ESI-MS: m/z [M+H]$^+$ (calc.) = 408.1311, m/z [M+Na]$^+$ (calc.) = 430.1131, m/z [M+H]$^+$ = 408.1314, m/z [M+Na]$^+$ = 430.1133.

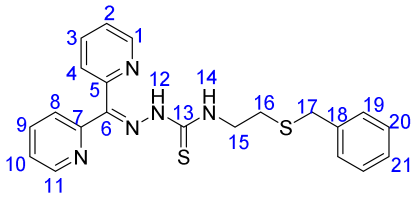

1.2.7 Syntheses of thiosemicarbazones with alkyne function

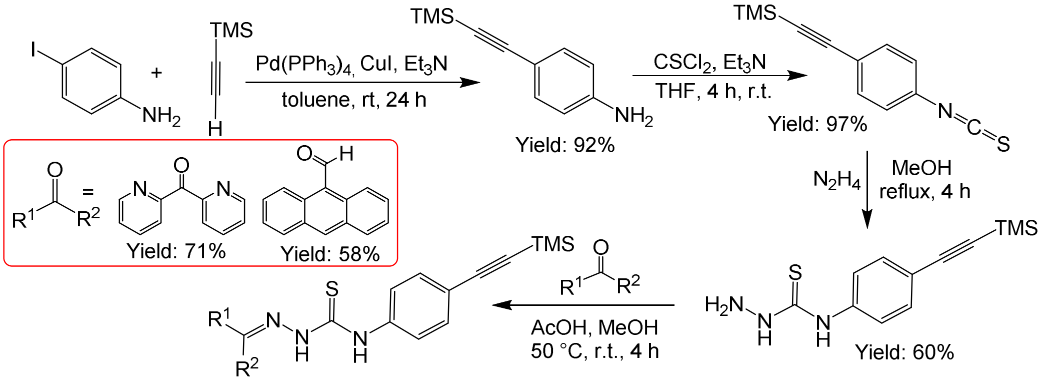

Scheme S7. Overview about the synthesis of TSCs with alkyne function.

1.2.7.1 Synthesis of 4-[(trimethylsilyl)ethynyl]aniline

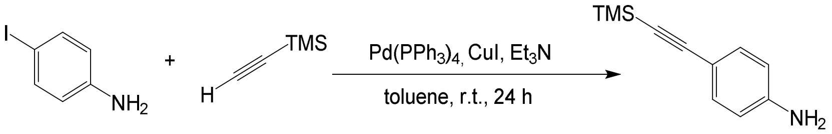

The reaction was performed under inert conditions. [Pd(PPh_3)$_4$] (0.28 g, 0.2 mmol, 0.03 eq.), CuI (0.14 g, 0.7 mmol, 0.09 eq.), 4-iodobenzene amine (1.75 g, 8 mmol, 1 eq.), trimethylsilyl acetylene (1.2 mL, 8.6 mmol, 1.08 eq.) and Et_3N (1.4 mL, 10 mmol, 1.25 eq.) were dissolved in 20 mL toluene and the reaction mixture was stirred for 24 h at room temperature. Then, the solvent was removed under reduced pressure and the product was purified by column chromatography (*c*-hexane/EtOAc 8:1 → 5:1 → 3:1 → 2:1 → EtOAc v/v). The product was obtained as yellow solid. Yield: 1.39 g (7.3 mmol, 92%). $C_{11}H_{15}NSi$ (189.33 g/mol). ^{1}H NMR (499 MHz, DMSO-*d*$_6$) δ [ppm] = 7.28 (d, *J* = 7.9 Hz, 2H), 6.61 (d, *J* = 8.4 Hz, 2H), 3.80 (s, 2H), 0.24 (s, 9H).

1.2.7.2 Synthesis of 4-[(trimethylsilyl)ethynyl] phenyl isothiocyanate

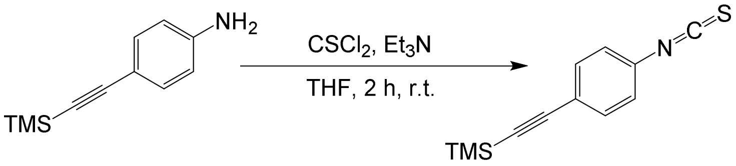

The reaction was performed under inert conditions. 1 g 4-[(trimethylsilyl)ethynyl]aniline (5.3 mmol, 1 eq.) and 2.2 mL Et_3N (15.8 mmol, 3 eq.) were dissolved in 50 mL THF and 0.44 mL thiophosgene (5.8 mmol, 1.1 eq.) was slowly added at 0 °C. The reaction mixture was stirred for 2 h at room temperature, which led to the formation of a brown suspension. The reaction was quenched by adding 100 mL demineralized H_2O and the product was extracted with 3 x 100 mL Et_2O. The organic phase was dried over Na_2SO_4, and the solvent was removed under reduced pressure. The product was obtained as brown oil. Yield: 1.2 g (5.2 mmol, 98%). $C_{12}H_{13}NSSi$ (231.39 g/mol). ^{1}H NMR (499 MHz, DMSO-*d*$_6$) δ [ppm] = 7.43 (d, *J* = 8.4 Hz, 2H), 7.16 (d, *J* = 8.4 Hz, 2H), 0.26 (s, 9H).

1.2.7.3 Synthesis of *N*-hydrazidecarbothio[4-(trimethylsilyl)ethynylphenyl]amine

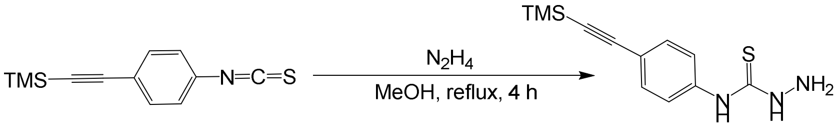

0.26 mL hydrazine monohydrate (5.3 mmol, 1 eq.) was dissolved in 50 mL MeOH and heated to 70 °C. 1.2 g 4-[(trimethylsilyl)ethynyl] phenyl isothiocyanate (5.3 mmol, 1 eq.) was dissolved in 50 mL MeOH and slowly added to the hydrazine solution over the course of 1 h. The reaction mixture was stirred for further 3 h followed by the removal of the solvent under reduced pressure, yielding a brown oil. The crude product was purified by column chromatography (*c*-hexane/EtOAc 3:1 → EtOAc v/v) and obtained as pale-yellow solid. Yield: 0.81 g (3.1 mmol, 60%). $C_{12}H_{17}N_3SSi$ (263.43 g/mol). ^{1}H NMR (499 MHz, DMSO-*d*$_6$) δ [ppm] = 9.26 (s, 1H, H10), 7.83–7.68 (m, 3H, H6, H7), 7.37 (d, *J* = 8.0 Hz, 2H, H5), 0.22 (s, 9H, H1). ^{13}C NMR (499 MHz, DMSO-*d*$_6$) δ [ppm] = 179.2 (C9), 140.5 (C4), 131.9 (C5), 122.83 (C6), 106.0 (C3), 93.6 (C2), 0.5 (C1). HR-ESI-MS: m/z [M+H]$^+$ (calc.) = 264.0985, m/z [M+H]$^+$ = 264.0986.

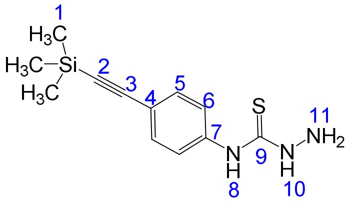

1.2.7.4 Synthesis of thiosemicarbazones – general description

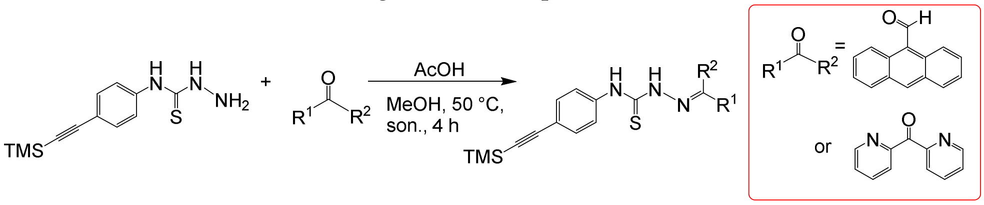

9-anthracene aldehyde or di(2-pyridyl) ketone (1 eq.) and *N*-hydrazidecarbothio[4-(trimethylsilyl) ethynylphenyl]amine (1 eq.) were dissolved in 50 mL MeOH and 0.1 mL glacial acetic acid were added. The reaction mixture was sonicated at 50 °C for 4 h. The solvent was removed under reduced pressure and the crude product was purified by column chromatography.

[(1*E*)-(anthracen-9-yl)methylideneamino][4-(trimethylsilyl)ethynylphenylamino] carbothioamide from 0.2 g (0.8 mmol) *N*-hydrazidecarbothio[4-(trimethylsilyl)ethynylphenyl]amine, 0.16 g (0.8 mmol) antracene-9-carbaldehyde. The formed precipitate was filtered off and washed with cold MeOH. The product was obtained as yellow solid. Yield: 0.2 g (0.4 mmol, 58%). $C_{27}H_{25}N_3SSi$ (451.66 g/mol). ^{1}H NMR (499 MHz, DMSO-*d*$_6$) δ [ppm] = 12.16 (s, 1H, H10), 10.13 (s, 1H, H12), 9.45 (s, 1H, H9), 8.72 (s, 1H, H1), 8.57 (d, *J* = 8.8 Hz, 2H, H6), 8.16 (d, *J* = 8.3 Hz, 2H, H3), 7.71 (d, *J* = 8.5 Hz, 2H, H14), 7.65 (t, *J* = 8.0 Hz, 2H, H4/H5), 7.58 (t, *J* = 8.0 Hz, 2H, H4/H5), 7.44 (d, *J* = 8.5 Hz, 2H, H15), 0.23 (s, 9H, H19). ^{13}C NMR (499 MHz, DMSO-*d*$_6$) δ [ppm] = 176.0 (C11), 143.4 (C9), 140.2 (C16), 131.9 (C15), 131.3 (C2), 130.2 (C7), 130.0 (C1), 129.4 (C3), 127.8 (C4/C5), 126.1 (C4/C5), 125.6 (C8), 125.3 (C6), 125.0 (C14), 118.8 (C13), 105.7 (C17), 94.2 (C18), 0.4 (C19). HR-ESI-MS: m/z [M+H]$^+$ (calc.) = 452.1611, m/z [M+Na]$^+$ (calc.) = 474.1431, m/z [M+H]$^+$ = 452.1615, m/z [M+Na]$^+$ = 474.1433.

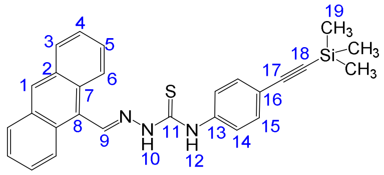

[Bis(pyridin-2-yl)methylideneamino][4-(trimethylsilyl)ethynylphenylamino] carbothioamide from 0.2 g (0.8 mmol) *N*-hydrazidecarbothio[4-(trimethylsilyl)ethynylphenyl]amine, 0.14 g (0.8 mmol) dipyridyl ketone. The product was purified by column chromatography (*c*-hexane/EtOAc 4:1 v/v) and obtained as brownish solid. Yield: 0.23 g (0.5 mmol, 71%). $C_{23}H_{23}N_5SSi$ (429.62 g/mol). ^{1}H NMR (499 MHz, DMSO-*d*$_6$) δ [ppm] = 13.70 (s, 1H, H12), 10.51 (s, 1H, H14), 8.86 (d, *J* = 4.2 Hz, 1H, H1), 8.60 (d, *J* = 4.2 Hz, 1H, H11), 8.37 (d, *J* = 8.0 Hz, 1H, C8), 8.02 (m, 2H, H3, H9), 7.68 (m, 2H, H16), 7.62 (m, 1H, H2), 7.56 (m, 2H, H4, H10), 7.52 (m, 2H, H17), 0.24 (s, 1H, H21). ^{13}C NMR (499 MHz, DMSO-*d*$_6$) δ [ppm] = 176.6 (C13), 155.3 (C7), 151.4 (C5), 148.8 (C11), 148.6 (C1), 142.6 (C6), 139.8 (C18), 138.1 (C3/C9), 137.6 (C3/C9), 132.1 (C4/C10, C17), 125.7 (C2), 125.4 (C16), 124.7 (C8), 124.6 (C4), 119.4 (C15), 105.5 (C19), 94.5 (C20), 0.4 (C21 HR-ESI-MS: m/z [M+H]$^+$ (calc.) = 430.1516, m/z [M+Na]$^+$ (calc.) = 452.1336, m/z [M+H]$^+$ = 430.1520, m/z [M+Na]$^+$ = 452.1339.

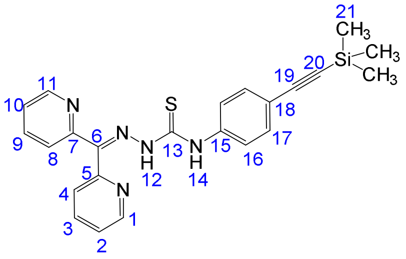

1.2.8 Syntheses of thiosemicarbazones with azide function

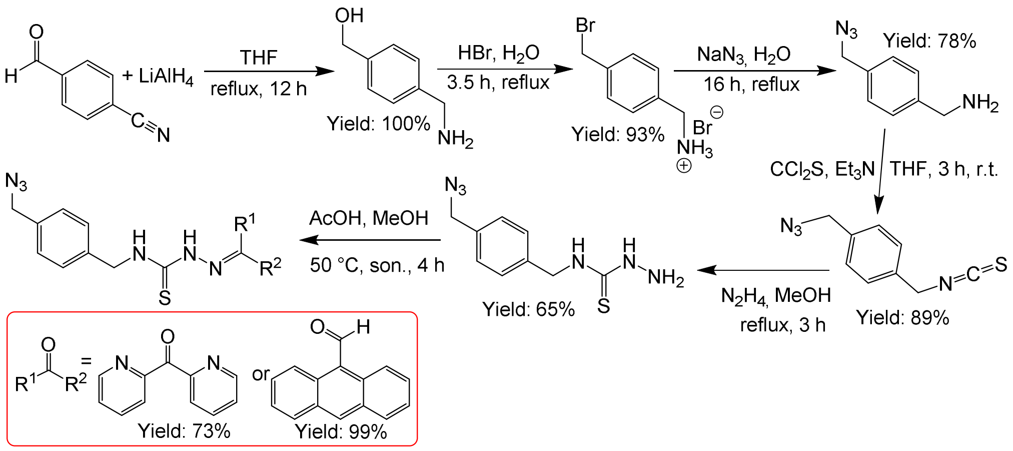

Scheme S8. Overview about the synthesis of TSCs with azide function.

1.2.8.1 Synthesis of 4-(aminomethyl)benzyl alcohol

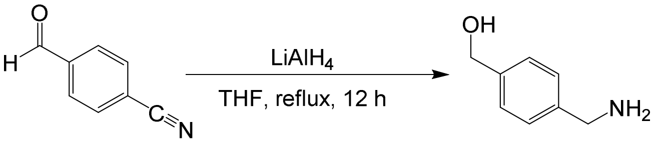

The reaction was performed under inert conditions. 4-cyanobenzaldehyde (5.11 g, 39.1 mmol, 1 eq.) was dissolved in 40 mL THF and added to a solution of 5.93 g $LiAlH_4$ (156 mmol, 4 eq.) in 120 mL THF at 0 °C. The reaction mixture was refluxed for 12 h. Then, the reaction was quenched with 20 mL H_2O and the mixture was filtered over Celite® and washed with 150 mL EtOAc. The solvent was removed under reduced pressure yielding the product as colorless solid. Yield: 5.4 g (39 mmol, quant.). $C_8H_{11}NO$ (137.18 g/mol). ^1H NMR (499 MHz, $CDCl_3$) δ [ppm] = 7.54 (s, 2H), 6.85 (s, 2H), 3.62 (m, 2H), 3.02 (m, 2H), 2.81 (m, 2H).

1.2.8.2 Synthesis of 4-(bromomethyl)benzylamine hydrobromide

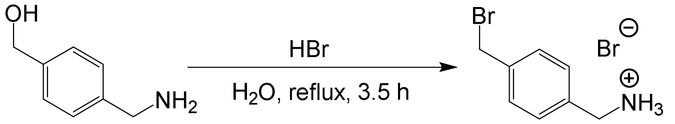

4-(Aminomethyl)benzyl alcohol (5.4 g, 39 mmol, 1 eq.) was dissolved in 60 mL H_2O and 95 mL HBr (46% aqueous solution). The reaction mixture was refluxed for 3.5 h. Then, the solvent was removed under reduced pressure, and the crude product was washed with 50 mL Et_2O and dried under ambient conditions. The product was obtained as colorless solid. Yield: 10.15 g (36.1 mmol, 93%). $C_8H_{10}BrN{\cdot}HBr$ (280.99 g/mol). ^1H NMR (499 MHz, DMSO-d_6) δ [ppm] = 8.27 (s, 3H), 7.48 (s, 4H), 4.72 (s, 2H), 4.02 (m, 2H).

1.2.8.3 Synthesis of 4-(azidomethyl)benzylamine

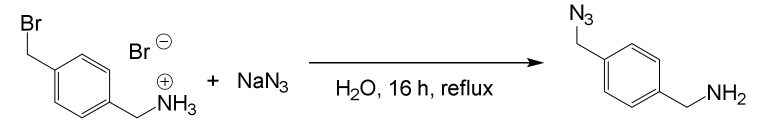

4-(Bromomethyl)benzylamine hydrobromide (5 g, 17.8 mmol, 1 eq.) was suspended in 50 mL H_2O and a solution of NaN_3 (3.47 g, 53.4 mmol, 3 eq.) in 70 mL H_2O was added. The reaction mixture was refluxed for 16 h. Then, half of the volume was removed under reduced pressure and 20 mL Et_2O was added. The mixture was cooled to 0 °C, 5 g KOH was added, and the mixture was stirred for 20 min. The organic phase was separated, and the aqueous phase was extracted with 3 x 50 mL Et_2O. The combined organic phase was dried over $NaSO_4$, and the solvent was removed under reduced pressure. The product was obtained as colorless solid. Yield: 2.26 g (13.9 mmol, 78%). $C_8H_{10}N_4$ (162.20 g/mol). ^1H NMR (499 MHz, DMSO-d_6) δ [ppm] = 7.29 (m, 4H), 4.30 (m, 2H), 3.86 (m, 2H), 1.59 (m, 2H).

1.2.8.4 Synthesis of 1-(azidomethyl)-4-(isothiocyanatomethyl)benzene

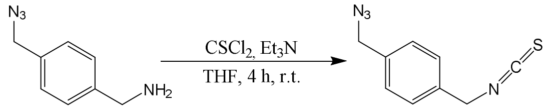

The reaction was performed under inert conditions. 4-(azidomethyl)benzylamine (1 g, 6.2 mmol, 1 eq.) and Et_3N (2.6 mL, 18.6 mmol, 3 eq.) were dissolved in 50 mL THF and thiophosgene (0.6 mL, 6.8 mmol, 1.1 eq., 85 %) was slowly added at 0 °C. The reaction mixture was stirred for 2 h at room temperature, which led to the formation of a brown suspension. The reaction was quenched by adding 100 mL demineralized H_2O and the product was extracted with 3 x 100 mL Et_2O. The organic phase was dried over Na_2SO_4 and the solvent was removed under reduced pressure. The product was obtained as brownish solid and used without further purification. Yield: 1.13 g (5.5 mmol, 89%). $C_9H_8N_4S$ (204.25 g/mol). ^1H NMR (499 MHz, DMSO-d_6) δ [ppm] = 7.35 (s, 4H), 4.73 (s, 2H), 4.36 (s, 2H).

1.2.8.5 Synthesis of 4-{[*p*-(azidomethyl)phenyl]methyl}thiosemicarbazide

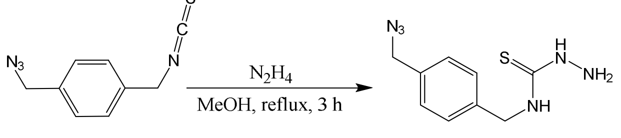

Hydrazine monohydrate (0.27 mL, 5.5 mmol) was dissolved in 50 mL MeOH and heated to 70 °C. 1-(azidomethyl)-4-(isothiocyanatomethyl)benzene (1.13 g, 5.5 mmol) was dissolved in 50 mL MeOH and slowly added to the hydrazine solution over the course of 1 h. The reaction mixture was stirred for further 3 h and the solvent was removed under reduced pressure. The crude product was purified by column chromatography (*c*-hexane/EtOAc 1:3 v/v). The product was obtained as brownish solid. Yield: 0.84 g (3.6 mmol, 65%). $C_9H_{12}N_6S$ (236.30 g/mol). ^{1}H NMR (499 MHz, DMSO-*d*$_6$) δ [ppm] = 8.75 (s, 1H, H9), 8.33 (s, 1H, H7), 7.32 (m, 4H, H3, H4), 4.72 (d, *J* = 6.0 Hz, 2H, H6), 4.51 (s, 2H, H10), 4.41 (s, 1H, H1).^{13}C NMR (499 MHz, DMSO-d$_6$) δ [ppm] = 182.0 (C8), 140.3 (C5), 134.3 (C2), 128.8 (C3/C4), 128.1 (C3/C4), 53.9 (C1), 46.3 (C6). HR-ESI-MS: m/z [M+H]$^+$ (calc.) = 237.0917, m/z [M+H]$^+$ = 237.0918.

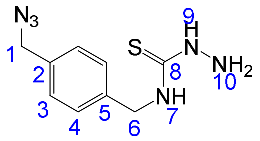

1.2.8.6 Synthesis of thiosemicarbazones – general description

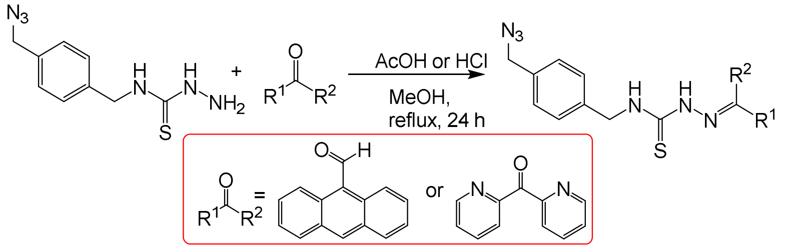

9-Anthracene aldehyde or di(2-pyridyl) ketone (1 eq.) and 4-{[*p*-(azidomethyl)phenyl]methyl} thiosemicarbazide (1 eq.) were dissolved in a minimum amount of MeOH and a few droplets of glacial acetic acid or conc. HCl were added. The reaction mixture was refluxed for 24 h. If the product precipitates during the reaction, the solid was filtered off and washed with cold MeOH. Otherwise, the solvent was removed under reduced pressure and the compound was purified by column chromatography.

[(2*E*)-2-[(Anthracen-9-yl)methylidene]hydrazino]-[4-(azidomethyl)phenyl]ethanethioamide from 0.2 g (0.8 mmol) 4-{[*p*-(azidomethyl)phenyl]methyl} thiosemicarbazide, 0.17 g (0.8 mmol) antracene-9-carbaldehyde., conc. HCl. The product was washed with cold EtOH and obtained as yellow solid. Yield: 0.34 g (0.8 mmol, 100%). $C_{24}H_{20}N_6S$ (424.53 g/mol). ^{1}H NMR (499 MHz, DMSO-*d*$_6$) δ [ppm] = 11.83 (s, 1H, H10), 9.32 (s, 1H, H9), 8.92 (m, 1H, H12), 8.70 (s, 1H, H1), 8.52 (d, *J* = 8.1 Hz, 2H, H6), 8.14 (d, *J* = 7.5 Hz, H3), 7.59 (m, 4H, H4, H5), 7.37 (m, 4H, H15, H16), 4.85 (d, *J* = 5.5 Hz, 2H, H13), 4.40 (s, 2H, H18). ^{13}C NMR (499 MHz, DMSO-*d*$_6$) δ [ppm] = 178.1 (C11), 142.3 (C9), 139.9 (C14), 134.7 (C17), 131.3 (C2), 130.0 (C7), 129.7 (C1), 129.3 (C3), 128.9 (C15/C16), 128.0 (C15/C16), 127.6 (C4/C5), 126.0 (C4/C5), 125.9 (C8), 125.3 (C6), 53.9 (C18), 47.0 (C13). HR-ESI-MS: m/z [M+H]$^+$ (calc.) = 425.1543, m/z [M+Na]$^+$ (calc.) = 447.1362, m/z [M+H]$^+$ = 425.1543, m/z [M+Na]$^+$ = 447.1364.

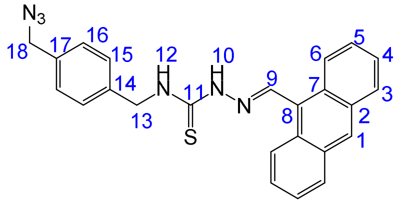

{2-[Bis(pyridin-2-yl)methylidene]hydrazino}-[4-(azidomethyl)phenyl]ethanethioamide from 0.2 g (0.8 mmol) 4-{[*p*-(azidomethyl)phenyl]methyl} thiosemicarbazide, (0.15 g, 0.8 mmol) dipyridyl ketone, glacial AcOH. The product was purified by column chromatography (*c*-hexane/EtOAc 1:3 v/v) and obtained as colorless solid. Yield: 0.25 g (0.6 mmol, 73%). $C_{20}H_{18}N_8S$ (402.48 g/mol). ^{1}H NMR (499 MHz, DMSO-*d*$_6$) δ [ppm] = 13.32 (s, 1H, H12), 9.46 (t, *J* = 6.2 Hz, 1H, H14), 8.85 (d, *J* = 4.9 Hz, 1H, H1), 8.58 (d, *J* = 4.8 Hz, 1H, H11), 8.26 (d, *J* = 7.9 Hz, 1H, H8), 7.97 (dtd, *J* = 15.6, 7.8, 1.8 Hz, 2H, H3, H9), 7.58 (m, 1H, H2), 7.52 (m, 1H, H4), 7.47 (H10), 7.35 (m, 4H, H17, H18), 4.90 (d, *J* = 6.2 Hz, 2H, H15), 4.41 (s, 2H, H20), ^{13}C NMR (499 MHz, DMSO-*d*$_6$) δ [ppm] = 178.6 (C13), 155.5 (C7), 151.5 (C5), 148.8 (C11), 148.6 (C1), 142.0 (C6), 139.4 (C16), 138.0 (C3/C9), 137.5 (C3/C9), 134.5 (C19), 128.9 (C17/C18), 128.00 (C17/C18), 127.7 (C4), 125.2 (C2), 124.4 (C10), 124.4 (C8), 53.9 (C20), 47.3 (C15). HR-ESI-MS: m/z [M+H]$^+$ (calc.) = 403.1448, m/z [M+Na]$^+$ (calc.) = 425.1267, m/z [M+H]$^+$ = 403.1451, m/z [M+Na]$^+$ = 425.1269.

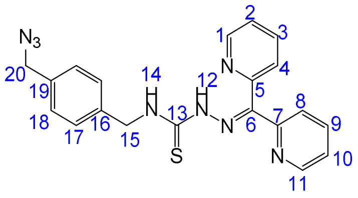

1.2.9 Synthesis of thiosemicarbazones with phosphonate group

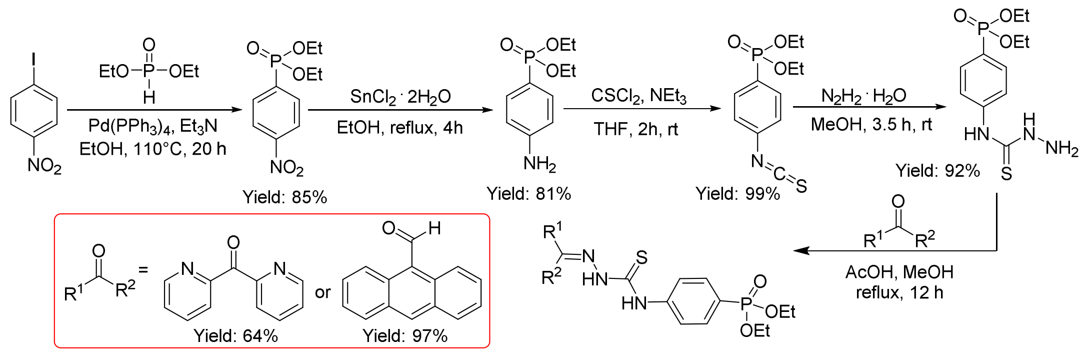

Scheme S9. Overview about the synthesis of TSCs with phosphonate group.

1.2.9.1 Synthesis of diethyl(4-nitrophenyl)phosphonate

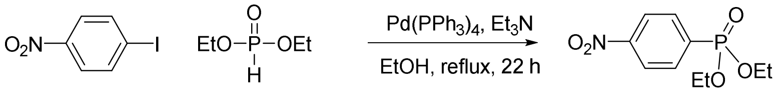

The reaction was performed under inert conditions. 1-Iodo-nitrobenzene (3.59 g, 14.4 mmol, 1 eq.) was dissolved in 70 mL EtOH and diethylphosphite (2.04 mL, 15.9 mmol, 1.1 eq.), Et_3N (8.04 mL, 57.7 mmol, 4 eq.) and [$Pd(PPh_3)_4$] (0.5 g, 0.43 mmol, 0.03 eq.) were added. The mixture was stirred at room temperature for 10 min and refluxed for 22 h. The solvent was removed under reduced pressure and the crude product was purified by column chromatography (*c*-hexane/EtOAc 1:1 v/v → EtOAc), yielding the product as brownish oil. Yield: 3.19 g (12.3 mmol, 85%). $C_{10}H_{14}NO_5P$ (259.20 g/mol). ^1H NMR (499 MHz, DMSO-d_6) δ [ppm] = 8.35 (dd, J = 8.8, 3.2 Hz, H6, 2H, H3), 7.99 (dd, J = 12.5, 8.8 Hz, H5, 2H, H2), 4.16–3.98 (m, H2, 4H, H5), 1.25 (t, J = 7.0 Hz, H1, 6H, H6). ^{13}C NMR (499 MHz, DMSO-d_6) δ [ppm] = 149.9 (C1), 136.2 (C4), 132.9 (C2), 123.6 (C3), 62.4 (C5), 16.2 (C6).

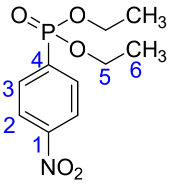

1.2.9.2 Synthesis of diethyl(4-aminophenyl)phosphonate

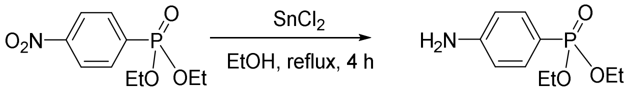

A solution of diethyl(4-nitrophenyl)phosphonate (0.5 g, 1.9 mmol, 1 eq.) and $SnCl_2$ x 2 H_2O (1.74 g, 7.7 mmol, 4 eq.) in 20 mL EtOH was heated under reflux for 4 h. The solvent was removed under reduced pressure and 20 mL 2 M NaOH solution was added to the residue. The precipitate was filtered off and washed with EtOAc. Then, the aqueous layer was extracted with 3 x 50 mL EtOAc and the combined organic layers were washed with 2 x 50 mL brine. The organic phase was dried over Na_2SO_4 and the solvent was removed under reduced pressure leaving the product as pale-yellow solid. Yield: 0.42 g (1.8 mmol, 94%). $C_{10}H_{16}NO_3P$ (229.22 g/mol). ^1H NMR (499 MHz, DMSO-d_6) δ [ppm] = 7.32 (dd, J = 12.5, 8.5 Hz, 2H, H3), 6.60 (dd, J = 8.5, 3.8 Hz, 2H, H4), 5.80 (s, 2H, H1), 4.03–3.77 (m, 4H, H6), 1.19 (t, J = 7.0 Hz, 6H, H7). ^{13}C NMR (499 MHz, DMSO-d_6) δ [ppm] = 153.0 (C2), 133.4 (C3), 113.4 (C4), 113.3 (C5), 61.3 (C6), 16.6 (C7).

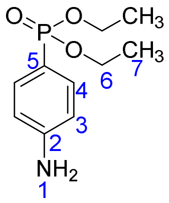

1.2.9.3 Synthesis of diethyl (4-isothiocyanatophenyl)phosphonate

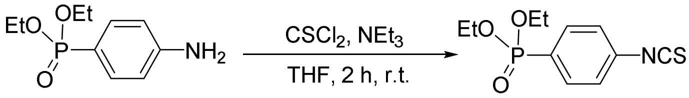

The reaction was performed under inert conditions. A solution of diethyl(4-aminophenyl)phosphonate (1 g, 4.4 mmol, 1 eq.) and Et_3N (1.82 mL, 13.1 mmol, 3 eq.) in 50 mL THF was cooled to 0 °C and thiophosgene (0.39 mL, 4.4 mmol, 1 eq., 85%) was added slowly. The reaction was stirred for 2 h at room temperature, followed by quenching with the addition of 100 mL H_2O. The aqueous phase was extracted with 3 x 100 mL Et_2O and the combined organic layers were dried over Na_2SO_4. The solvent was removed under reduced pressure and the crude product was purified by column chromatography (EtOAc), yielding the product as dark reddish oil. Yield: 1.18 g (4.4 mmol, 99%). $C_{11}H_{14}NO_3PS$ (271.27

g/mol). ^{1}H NMR (300 MHz, DMSO-d_6) δ [ppm] = 7.76 (dd, *J* = 12.8, 8.4 Hz, 2H), 7.57 (dd, *J* = 8.4, 3.4 Hz, 2H), 4.18–3.88 (m, 4H), 1.23 (t, *J* = 7.0 Hz, 6H).

1.2.9.4 Synthesis of diethyl [4-(hydrazidecarbothioamino)phenyl] phosphonate

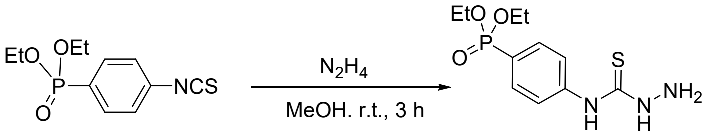

To a stirred solution of 0.29 mL hydrazine hydrate (1.1 mmol, 1 eq.) in 50 mL MeOH a solution of 0.3 g diethyl(4-isothiocyanatephenyl)phosphonate (1.1 mmol, 1 eq.) in 20 mL MeOH was added over the course of 1.5 h. The reaction was stirred for further 2 h at room temperature. The solvent was removed under reduced pressure yielding the product as yellowish oil, which was used without further purification. Yield: 0.22 g (1.1 mmol, quant.). $C_{11}H_{18}N_3O_3PS$ (303.32 g/mol). ^{1}H NMR (300 MHz, DMSO-d_6) δ [ppm] = 7.93 (m, H6, 2H), 7.60 (dd, *J* = 12.7, 8.6 Hz, H5, 2H), 4.15–3.82 (m, H2, 4H), 1.29 (t, *J* = 7.1 Hz, H1, 6H).

1.2.9.5 Synthesis of thiosemicarbazones – general description

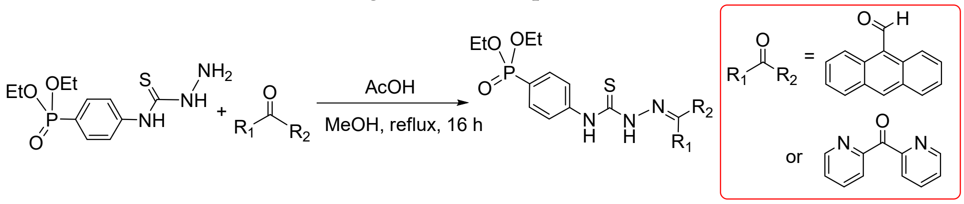

To a stirred solution of diethyl(4-thiosemicarbazidephenyl)phosphonate (1 eq.) in 50 mL MeOH was added di-2-pyridylketone or 9-anthracene aldehyde (1 eq.) and a few droplets of glacial acetic acid. The reaction mixture was refluxed for 16 h. If the product precipitates during the reaction, the solid was filtered off and washed with cold MeOH. Otherwise, the solvent was removed under reduced pressure and the compound was purified by column chromatography.

[(2*E*)-2-[(Anthracen-9-yl)methylidene]hydrazino]-4-(diethoxyphosphoryl)benzene-1-carbothioamide from 0.22 g (0.7 mmol) diethyl [4-(hydrazidecarbothioamino)phenyl] phosphonate, 0.15 g (0.73 mmol) 9-anthraaldehyde. The product was filtered off, washed with cold MeOH and obtained as yellow solid. Yield: 0.35 g (0.7 mmol, 97%). $C_{26}H_{26}N_3O_3PS$ (491.55 g/mol). ^{1}H NMR (499 MHz, DMSO-d_6) δ [ppm] = 12.21 (s, 1H, H10), 10.23 (s, 1H, H12), 9.43 (s, 1H, H9), 8.74 (s, 1H, H1), 8.58 (dd, *J* = 8.7, 1.2 Hz, 2H, H6), 8.19 (m, 2H, H3), 7.87 (dd, *J* = 8.6, 3.7 Hz, 2H, H14/H15), 7.77–7.34 (m, 6H, H14/H15, H4/H5), 4.15–3.76 (m, 4H, H17), 1.23 (t, *J* = 7.0 Hz, 6H, H18). ^{13}C NMR (499 MHz, DMSO-d_6) δ [ppm] = 176.2 (C11), 143.6 (C9), 143.4 (C16), 131.9 (C14/C15), 131.3 (C2), 130.2 (C7), 130.1 (C1), 129.5 (C3), 127.8 (C4/C5), 126.1 (C4/C5), 125.5 (C7), 125.3 (C6), 124.9 (C14/C15), 123.8 (C13), 62.1 (C17), 16.6 (C18). HR-ESI-MS: m/z [M+H]$^+$ (calc.) = 492.1505, m/z [M+Na]$^+$ (calc.) = 514.1325, m/z [M+H]$^+$ = 492.1509, m/z [M+Na]$^+$ = 514.1333.

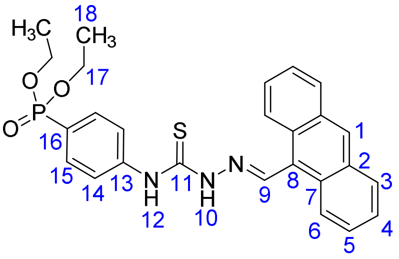

{2-[Bis(pyridin-2-yl)methylidene]hydrazino}-4-(diethoxyphosphoryl)benzene-1-carbothioamide from 0.22 g (0.7 mmol) diethyl [4-(hydrazidecarbothioamino)phenyl] phosphonate, 0.14 g (0.7 mmol) dipyridyl ketone. The product was purified by column chromatography (CH_2Cl_2/MeOH, 95:5) and obtained as yellow solid. Yield: 0.22 g (0.5 mmol, 64%). $C_{22}H_{24}N_5O_3PS$ (469.50 g/mol). ^1H NMR (499 MHz, DMSO-*d*$_6$) δ [ppm] = 13.76 (s, H10, 1H, H12), 10.58 (s, H8, 1H, H14), 8.85 (d, *J* = 4.0 Hz, 1H, H1), 8.60 (d, *J* = 4.5 Hz, 1H, H11), 8.34 (d, *J* = 7.9 Hz, 2H, H8), 8.00 (dtd, *J* = 14.6, 7.8, 1.8 Hz, 2H, H3, H9), 7.85 (dd, *J* = 8.4, 3.6 Hz, 2H, H16/H17), 7.72 (dd, *J* = 12.8, 8.4 Hz,2H, H16/H17), 7.61 (m, 1H, H2), 7.55 (m, 1H, H4), 7.51 (m, H10), 4.17–3.87 (m, 4H, H19), 1.24 (t, *J* = 7.0 Hz, 6H, H20). ^{13}C NMR (499 MHz, DMSO-*d*$_6$) δ [ppm] = 176.9 (C13), 155.2 (C7), 151.4 (C5), 148.8 (C11), 148.6 (C1), 143.0 (C15/C18), 142.8 (C6), 138.2 (C3/C9), 137.7 (C3/C9), 131.9 (C4, C16/C17), 126.5 (C2), 126.1 (C15/C18), 125.6 (C16/C17), 124.7 (C10), 124.6 (C8), 62.2 (C19), 16.6 (C20). HR-ESI-MS: m/z $[M+H]^+$ (calc.) = 470.1410, m/z $[M+Na]^+$ (calc.) = 492.1230, m/z $[M+H]^+$ = 470.1413 , m/z $[M+Na]^+$ = 492.1232.

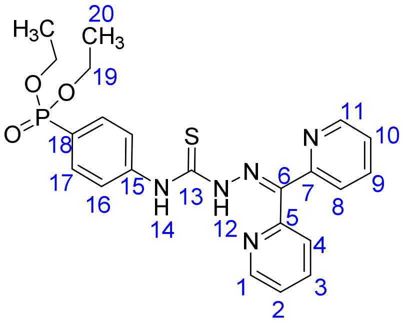

1.2.9.6 Deprotection of the TSC-phosphonate conjugates – general description

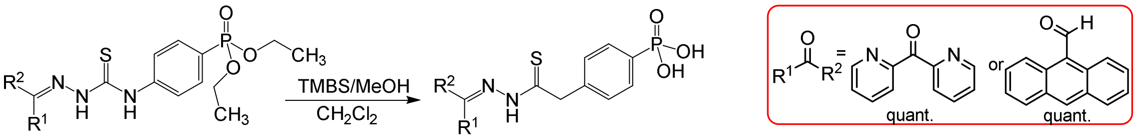

The reaction was performed under inert conditions. To a solution of the thiosemicarbazone (1 eq.) in 30 mL dry CH_2Cl_2 was added trimethylsilanbromide (TMBS) (7 eq.). The reaction mixture was stirred at room temperature for 24 h. After that, the solvent was removed under reduced pressure and the residue was re-dissolved in MeOH. After stirring for further 3 h at room temperature, the solvent was removed under reduced pressure and the product was dried under high vacuum for 10 h at 50 °C.

(4-{[bis(pyridin-2-yl)methylideneaminocarbamthioyl]amino}phenyl)phosphonic acid from 0.1 g (0.21 mmol) {2-[bis(pyridin-2-yl)methylidene]hydrazino}-4-(diethoxyphosphoryl)benzene-1-carbothio-amide, 0.19 mL (1.5 mmol) TMBS. The product was obtained as an orange solid. Yield: 0.08 g (0.21 mmol, quant.). $C_{18}H_{16}N_5O_3PS$ (413.39 g/mol). ^1H NMR (499 MHz, DMSO-*d*$_6$) δ [ppm] = 13.46 (s, 1H), 10.66 (s, 1H), 8.93 (d, *J* = 4.3 Hz, 1H), 8.74 (d, *J* = 5.1 Hz, 1H), 8.36 (d, *J* = 8.0 Hz, 2H), 8.17 (m, 2H), 7.71 (m, 7H).

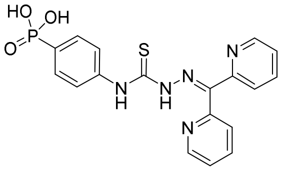

[(2*Z*)-2-[(anthracen-9-yl)methylidene]hydrazino]-4-phosphonobenzene-1-carbothioamide from 0.06 g (0.12 mmol) [(2*E*)-2-[(anthracen-9-yl)methylidene]hydrazino]-4-(diethoxyphosphoryl)benzene-1-carbothioamide, 0.11 mL (0.85 mmol) TMBS. The product was obtained as a yellow solid. Yield: 0.05 g (0.12 mmol, quant.). $C_{22}H_{18}N_3O_3PS$ (435.55 g/mol). ^1H NMR (499 MHz, DMSO-*d*$_6$) δ [ppm] = 12.15 (s, 1H), 10.14 (s, 1H), 8.75 (s, 1H), 8.57 (d, *J* = 8.7 Hz, 2H), 8.17 (d, *J* = 8.0 Hz, 2H), 7.62 (m, 7H).

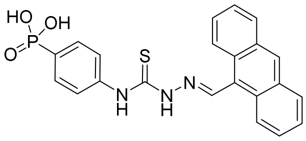

1.2.9.7 Anchoring the TSC-phosphonic acid conjugates on TiO_2 NPs – general description

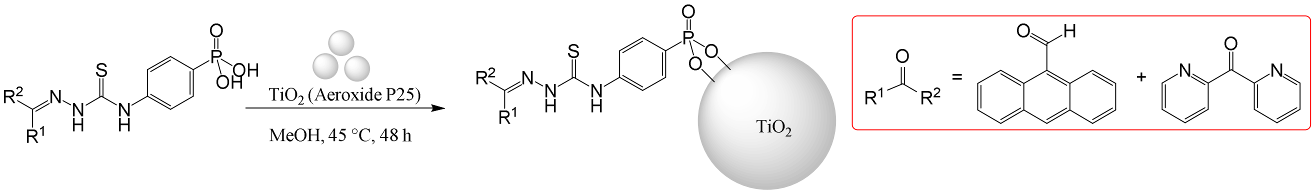

10 mg TiO_2 NP (Aeroxid P25®) were dispersed in 2 mL MeOH using ultrasound. After 15 min, a solution of the thiosemicarbazone in 3 mL MeOH was added and the mixture was stirred for 48 h at 45 °C. The particles were separated by centrifugation (5 min, 12000 rpm) and washed 10 times with MeOH. The functionalized particles were dried in a drying oven (45 °C) for 5 h.

2. Supplementary Figures

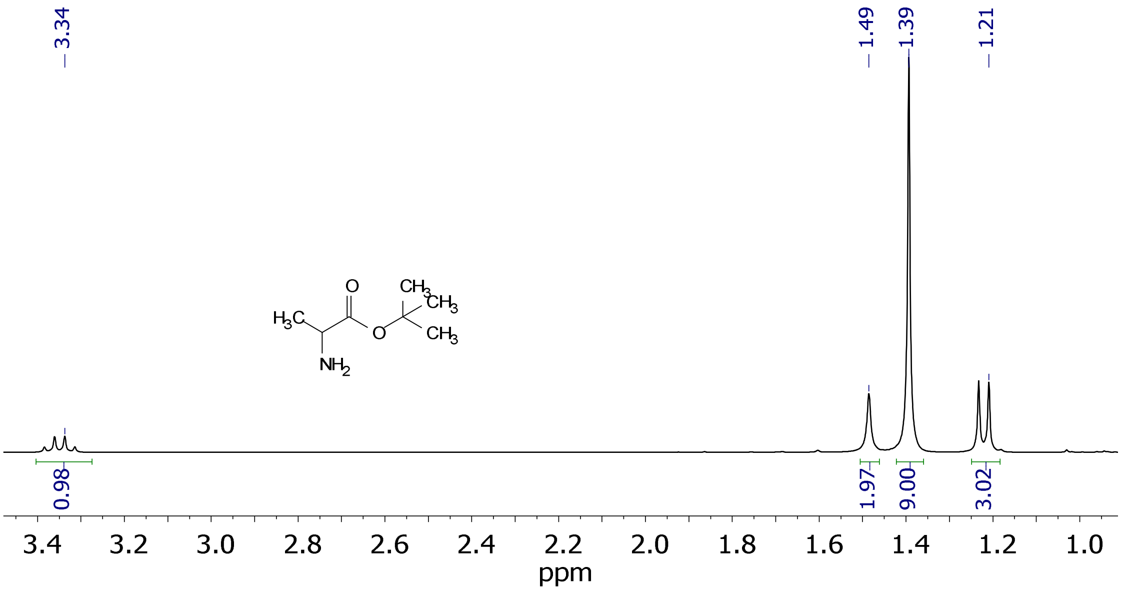

Figure S1. ^{1}H NMR spectrum of *L*-alanine *tert*-butyl ester in $CDCl_3$ at 499 MHz.

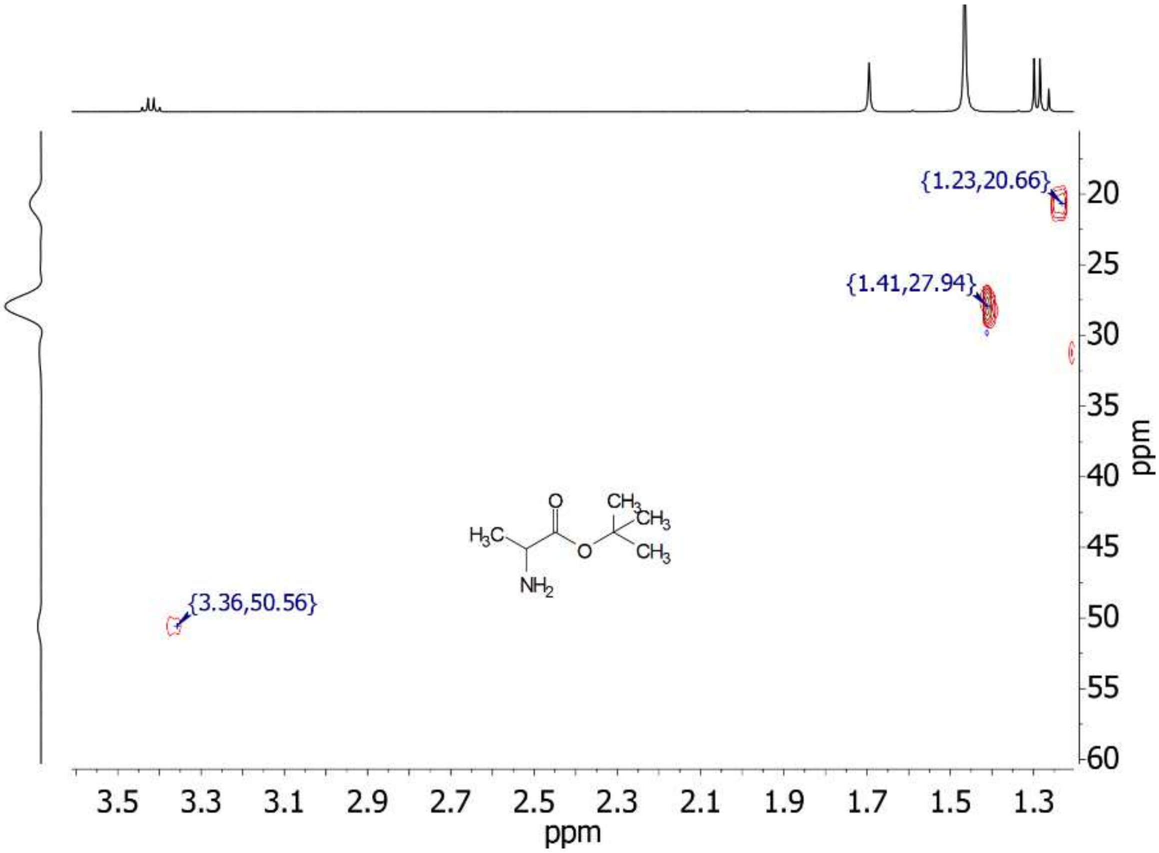

Figure S2. ^{1}H,^{13}C HMQC/HSQC NMR spectrum of *L*-alanine *tert*-butyl ester in $CDCl_3$ at 499 MHz.

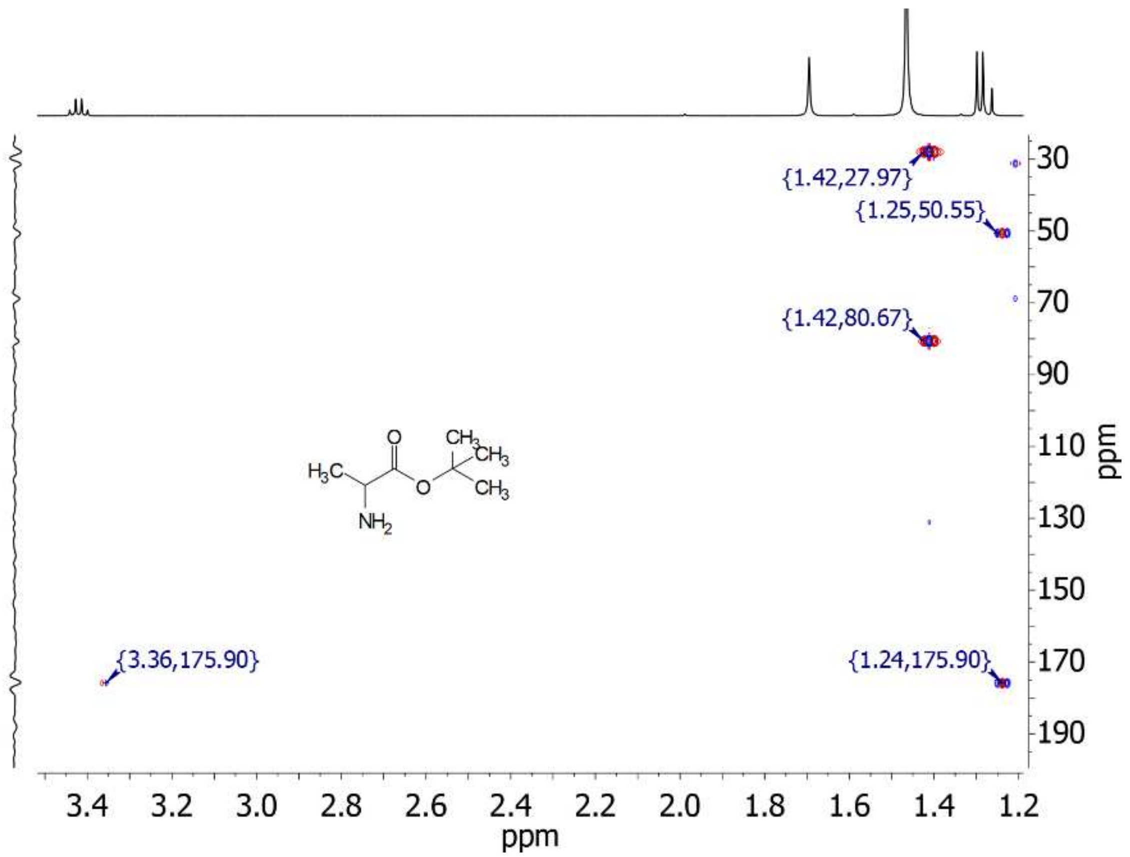

Figure S3. $^{1}H,^{13}C$ HMBC NMR spectrum of *L*-alanine *tert*-butyl ester in $CDCl_3$ at 499 MHz.

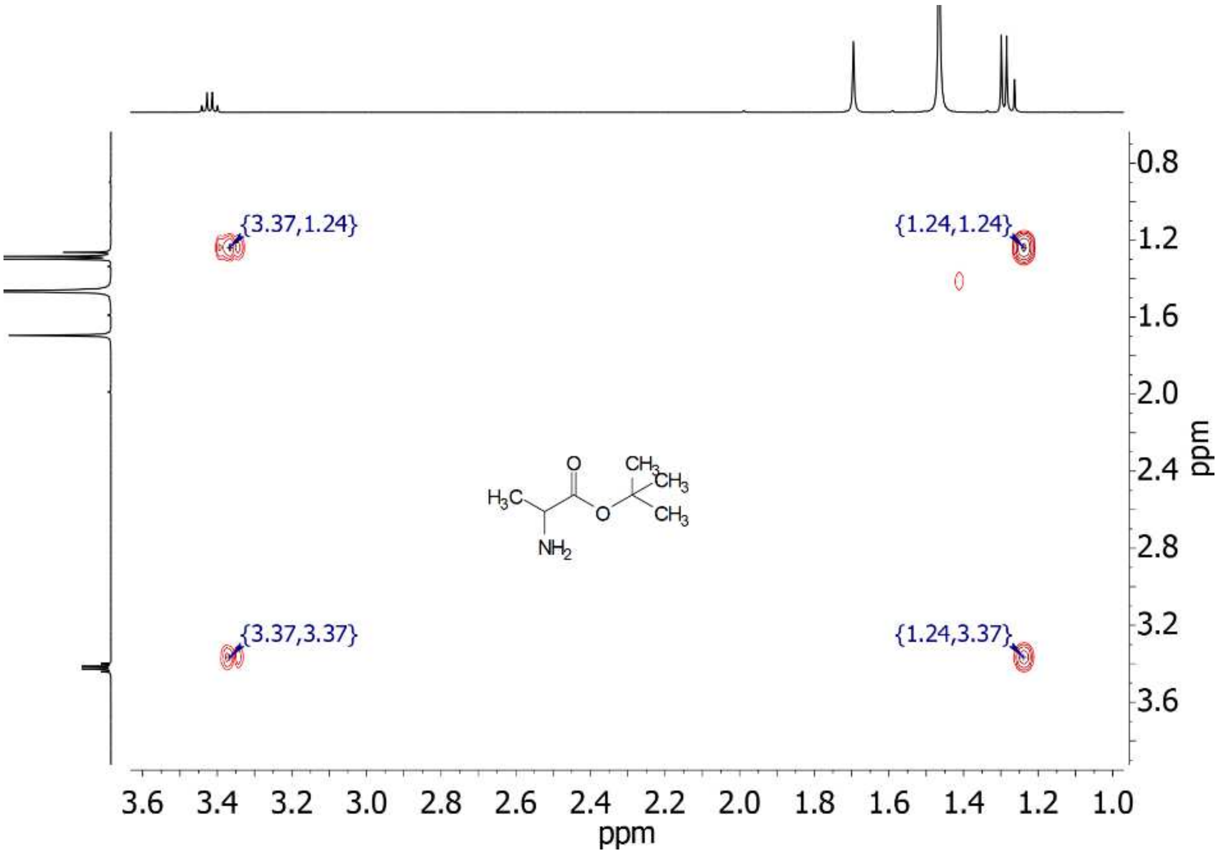

Figure S4. $^{1}H,^{1}H$ COSY NMR spectrum of *L*-alanine *tert*-butyl ester spectrum in $CDCl_3$ at 499 MHz.

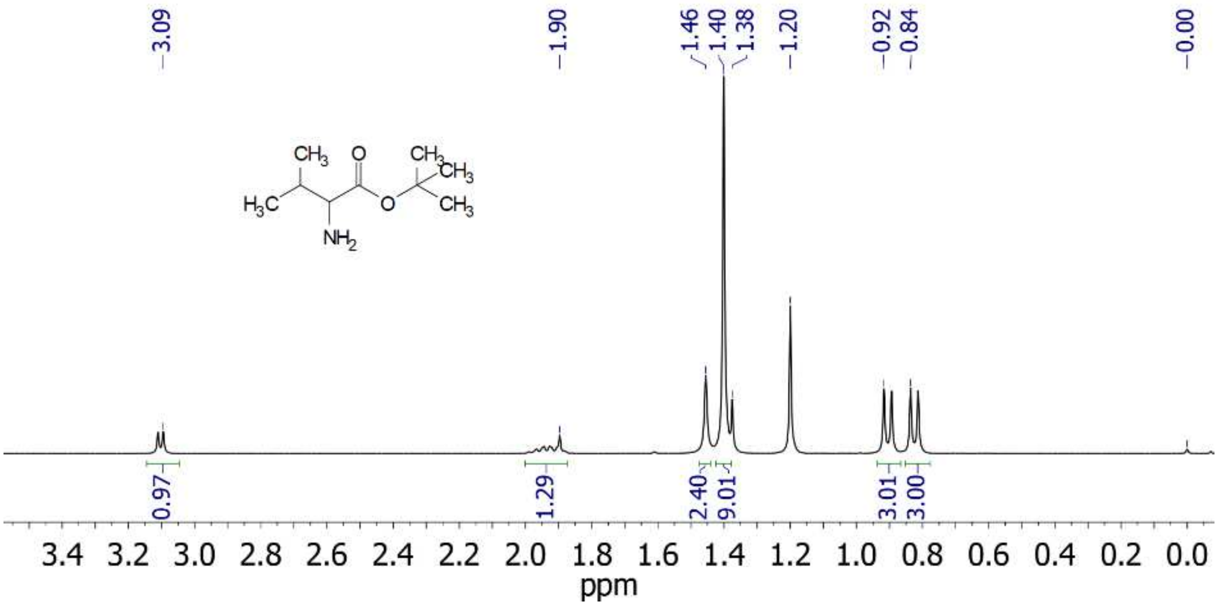

Figure S5. ^{1}H NMR spectrum of *L*-valine *tert*-butyl ester in $CDCl_3$ at 300 MHz.

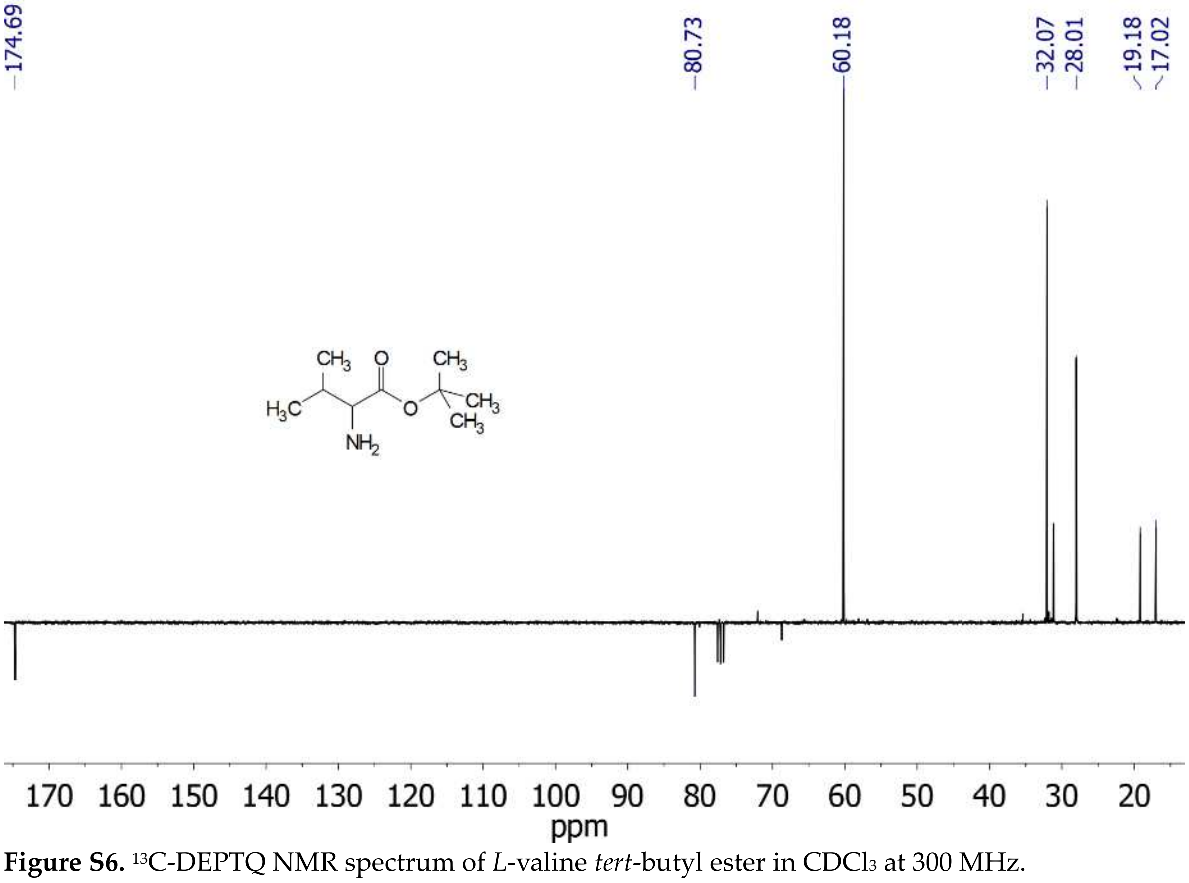

Figure S6. ^{13}C-DEPTQ NMR spectrum of *L*-valine *tert*-butyl ester in $CDCl_3$ at 300 MHz.

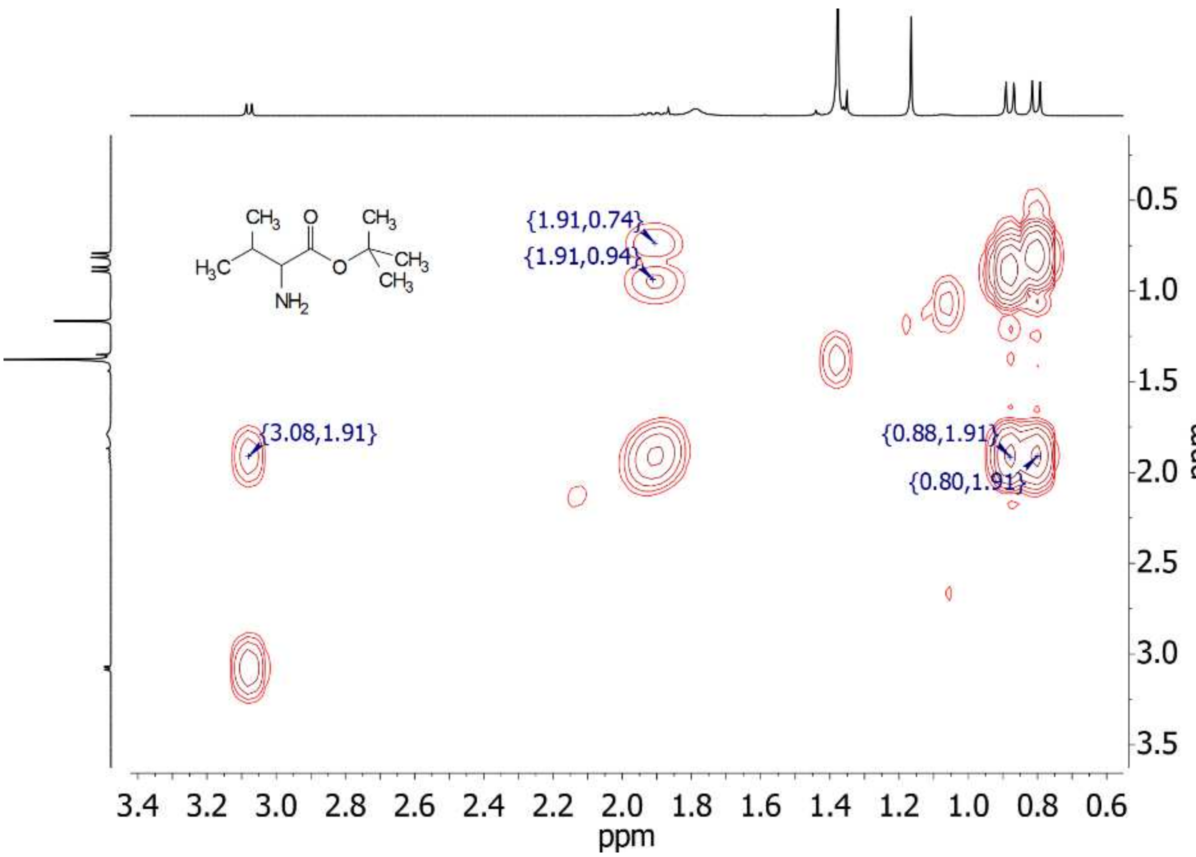

Figure S7. ^{1}H,^{1}H COSY NMR spectrum of *L*-valine *tert*-butyl ester in $CDCl_3$ at 300 MHz.

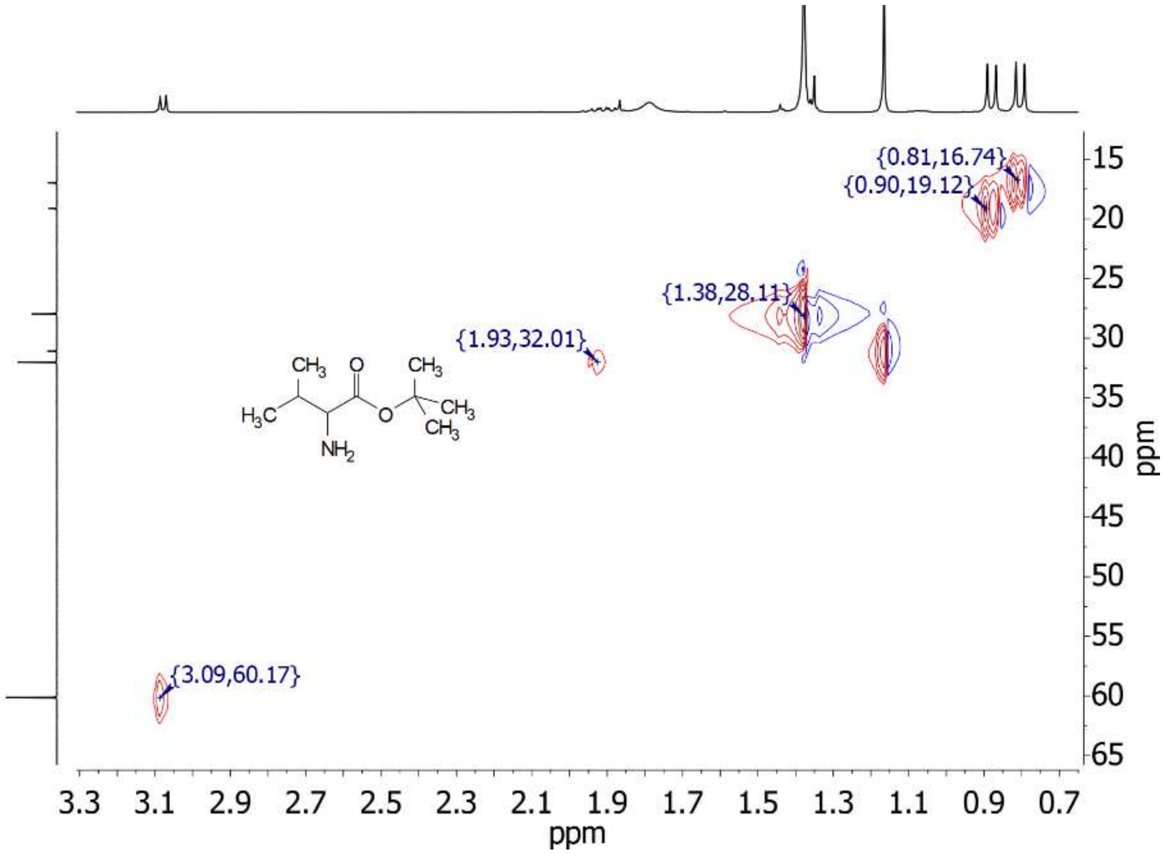

Figure S8. ^{1}H ^{13}C HMQC/HSQC NMR spectrum of *L*-valine *tert*-butyl ester in $CDCl_3$ at 300 MHz.

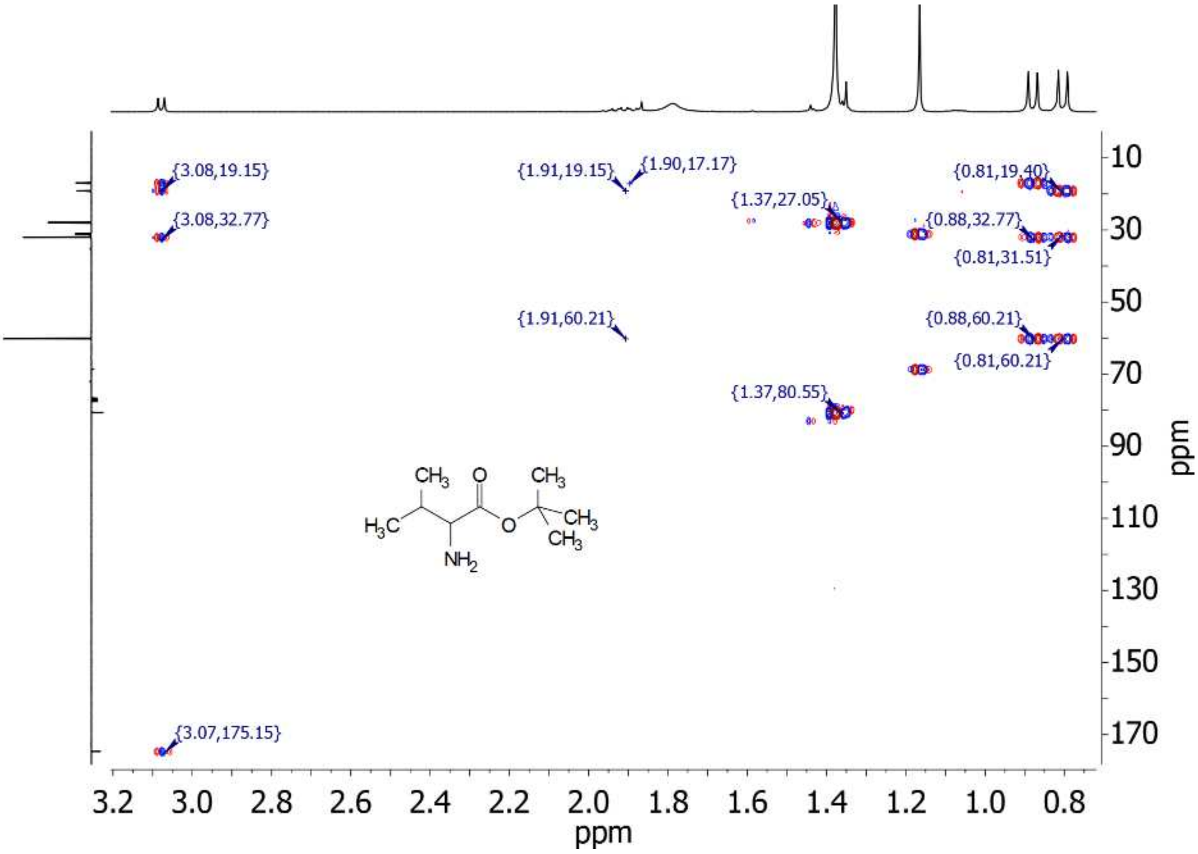

Figure S9. $^{1}H,^{13}C$ HMBC NMR spectrum of *L*-valine *tert*-butyl ester in $CDCl_3$ at 300 MHz.

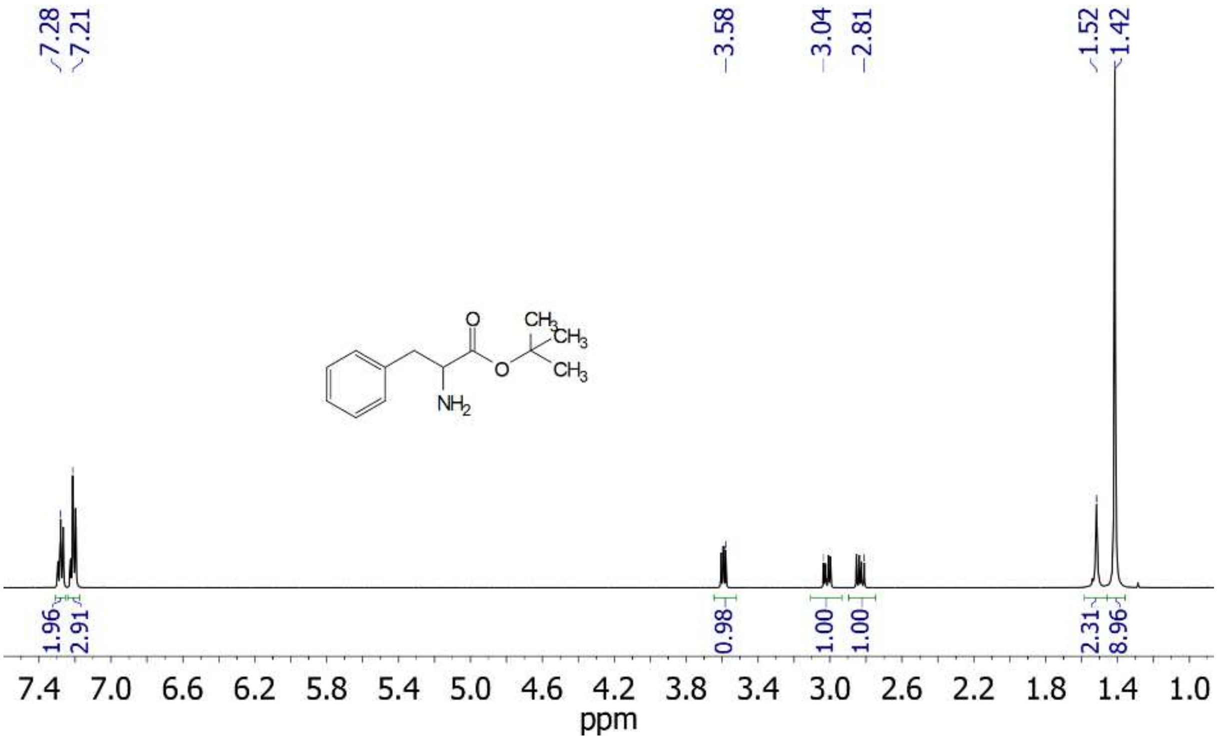

Figure S10. ^{1}H NMR spectrum of *L*-phenylalanine *tert*-butyl ester in $CDCl_3$ at 499 MHz.

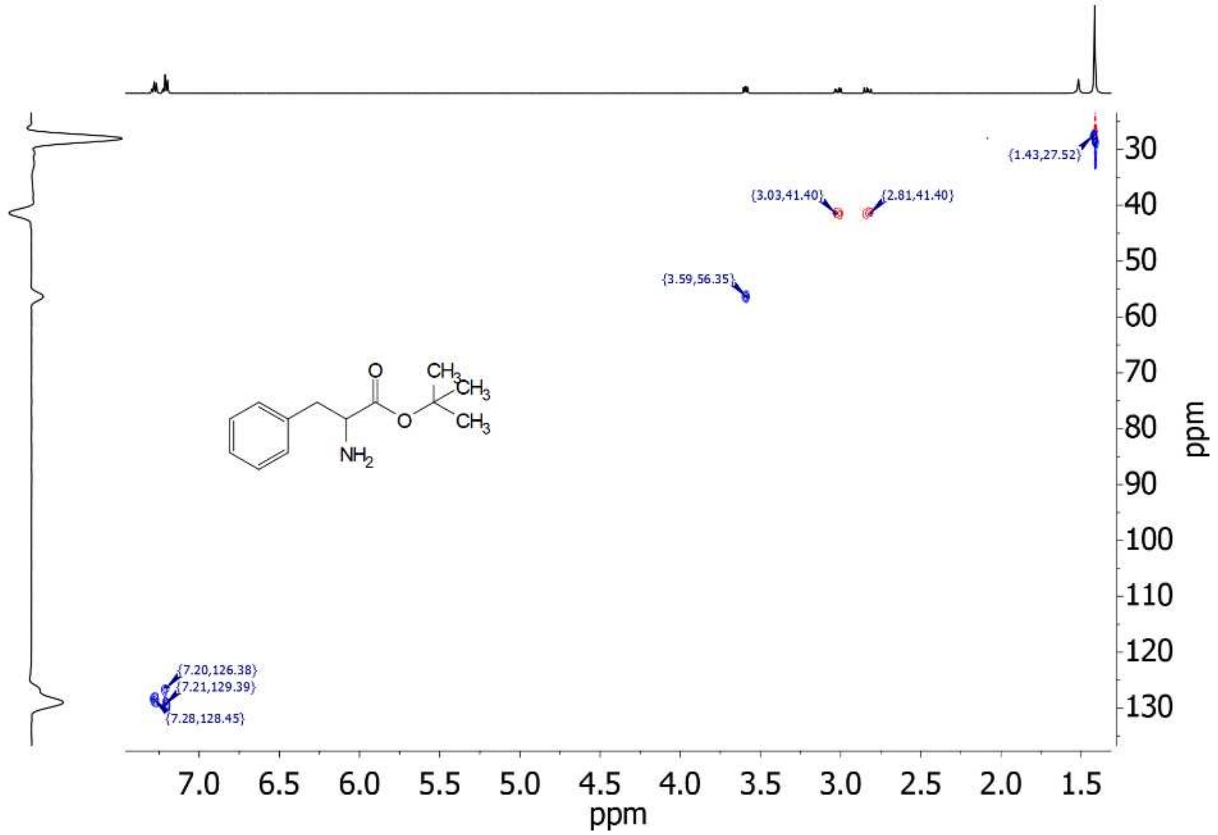

Figure S11. ^{1}H,^{13}C HMQC/HSQC NMR spectrum of *L*-phenylalanine *tert*-butyl ester in $CDCl_3$ at 499 MHz.

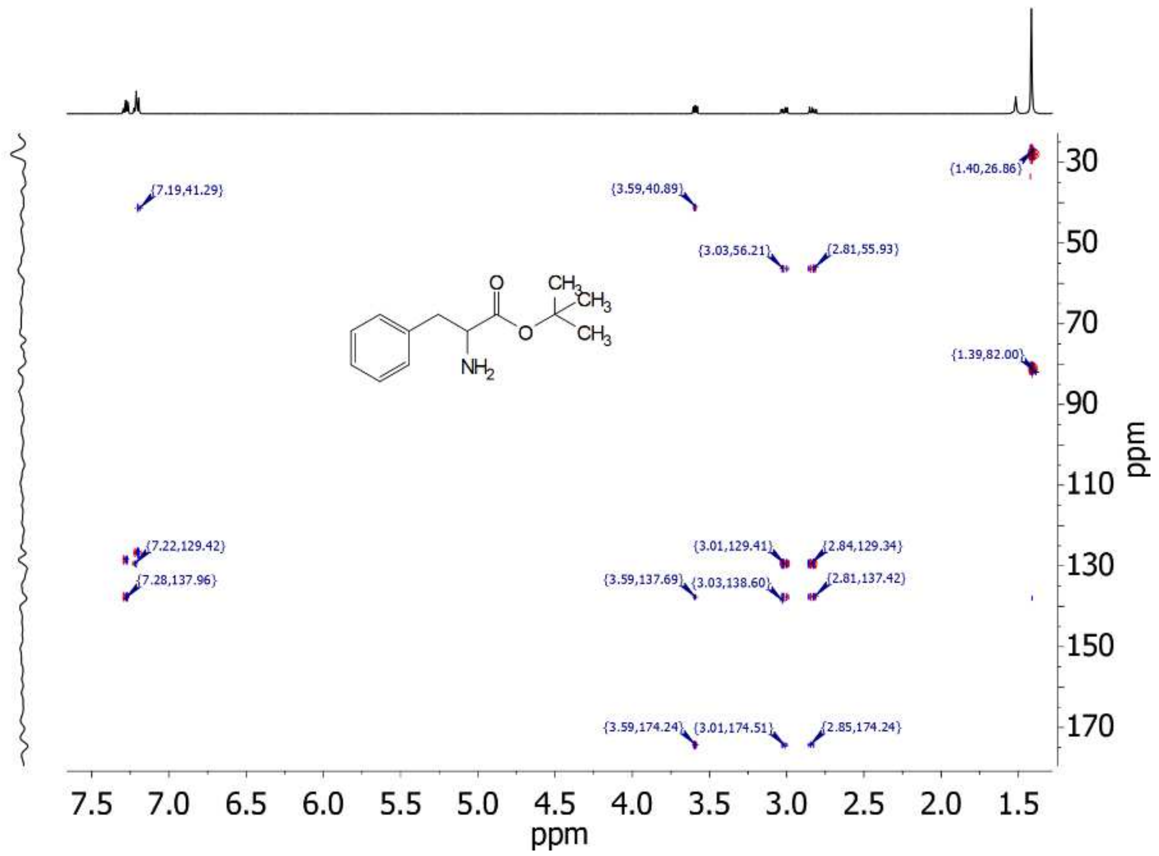

Figure S12. ^{1}H,^{13}C HMBC NMR spectrum of *L*-phenylalanine *tert*-butyl ester in $CDCl_3$ at 499 MHz.

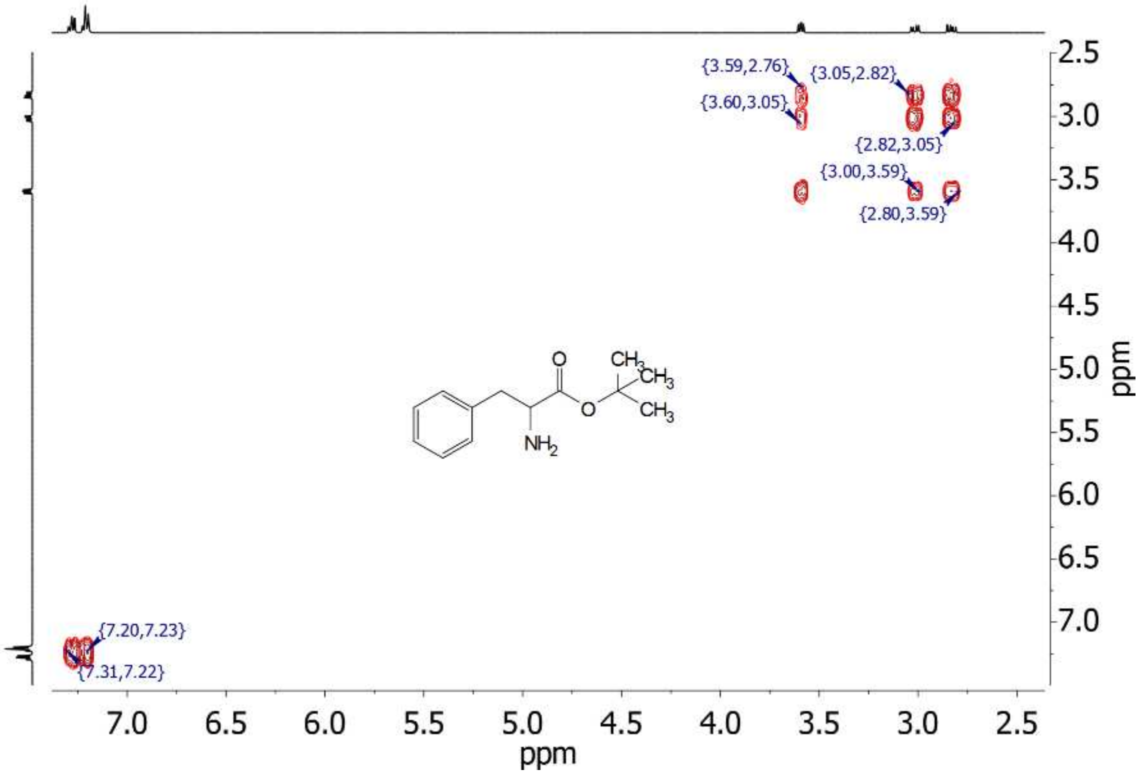

Figure S13. $^{1}H,^{1}H$ COSY NMR spectrum of *L*-phenylalanine *tert*-butyl ester in $CDCl_3$ at 499 MHz.

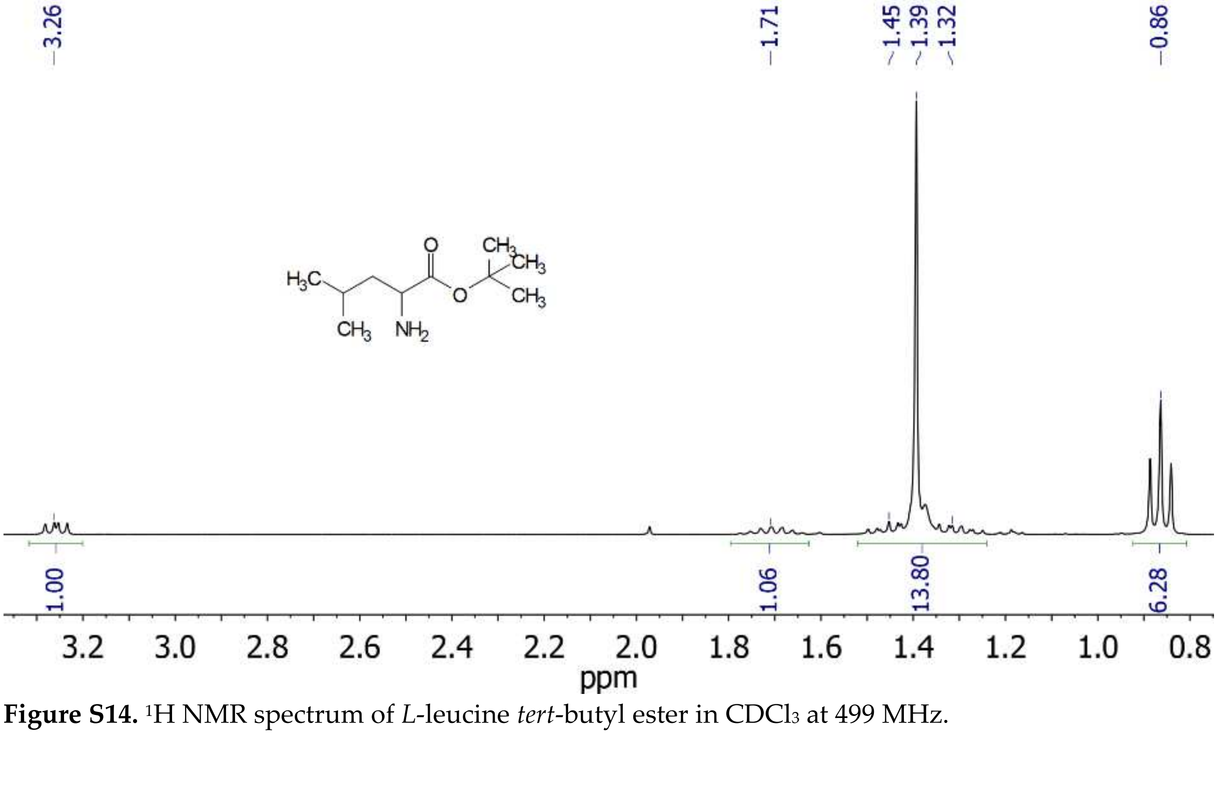

Figure S14. ^{1}H NMR spectrum of *L*-leucine *tert*-butyl ester in $CDCl_3$ at 499 MHz.

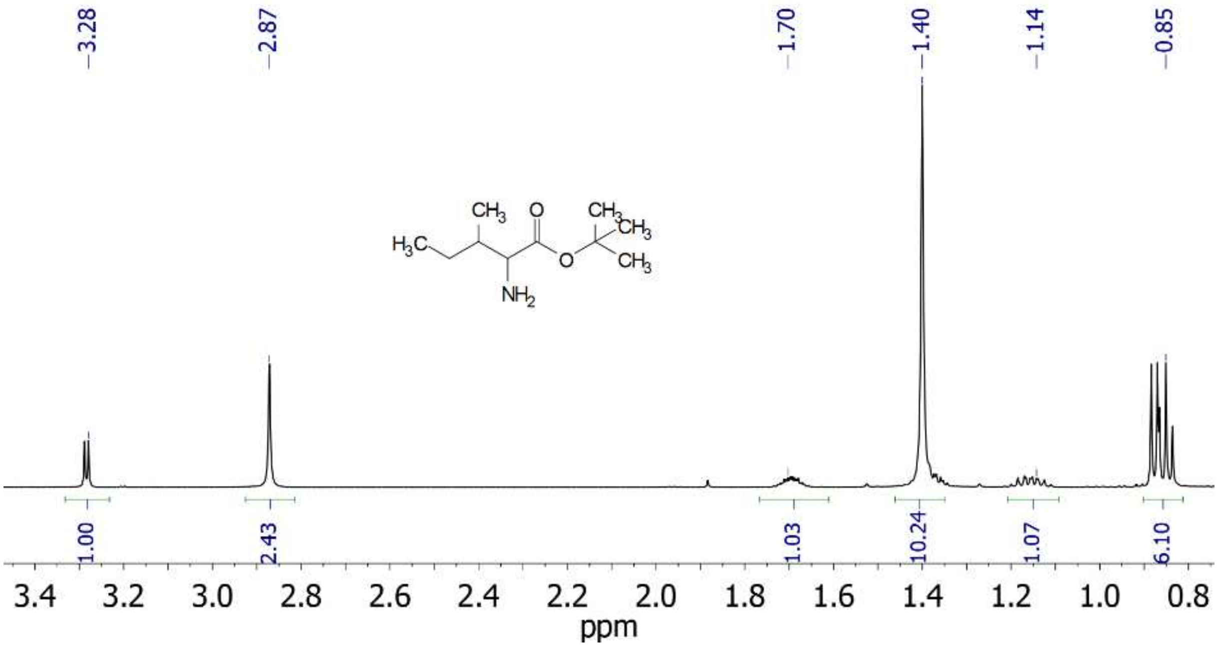

Figure S15. ^{1}H NMR spectrum of *L*-isoleucine *tert*-butyl ester in $CDCl_3$ at 499 MHz.

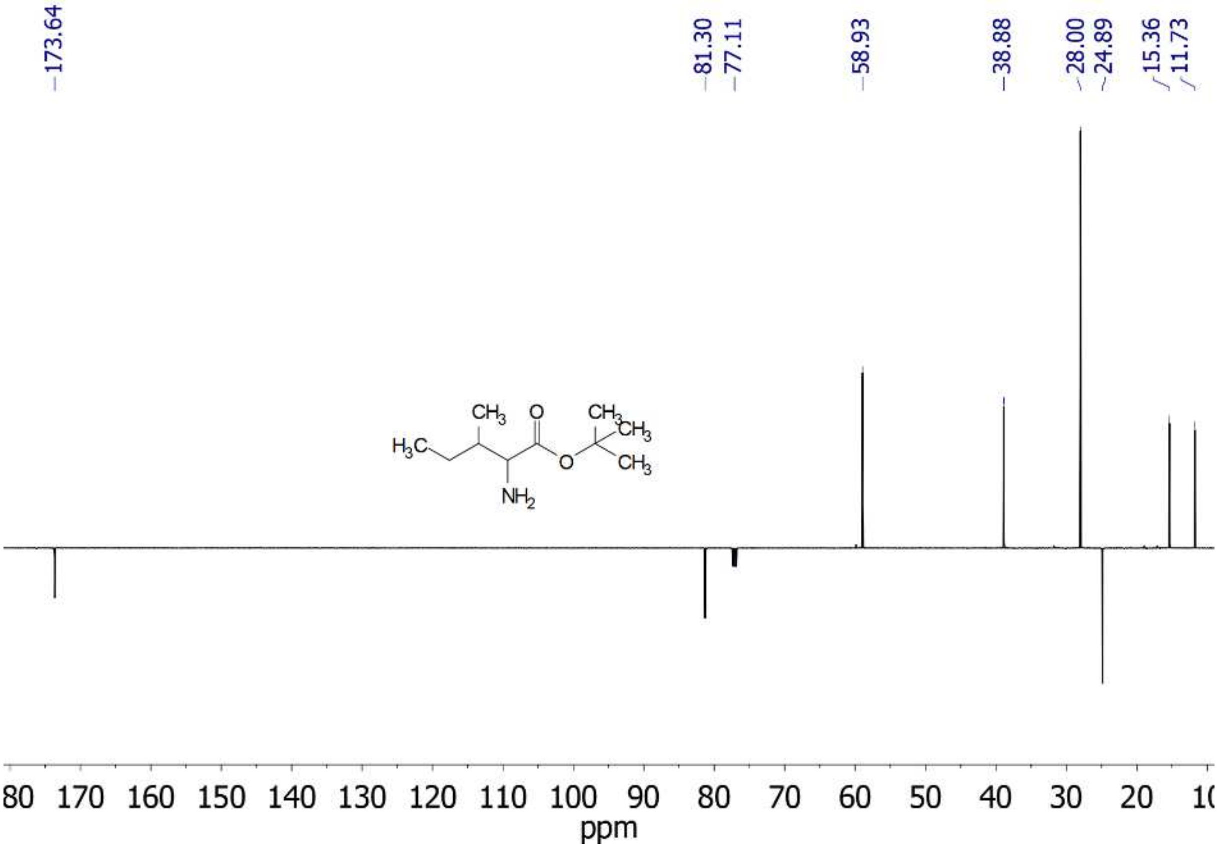

Figure S16. ^{13}C APT NMR spectrum of *L*-isoleucine *tert*-butyl ester in $CDCl_3$ at 499 MHz.

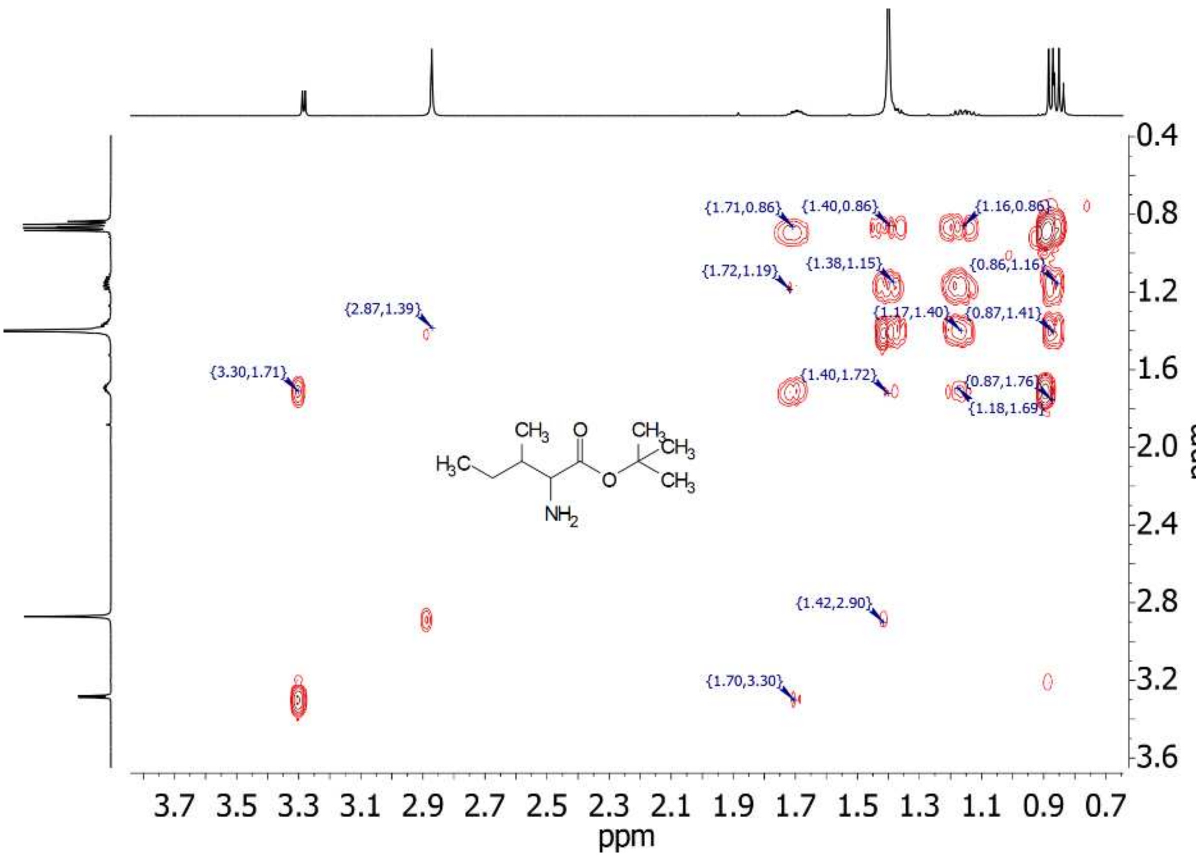

Figure S17. $^1H,^1H$ COSY NMR spectrum of *L*-isoleucine *tert*-butyl ester in $CDCl_3$ at 499 MHz.

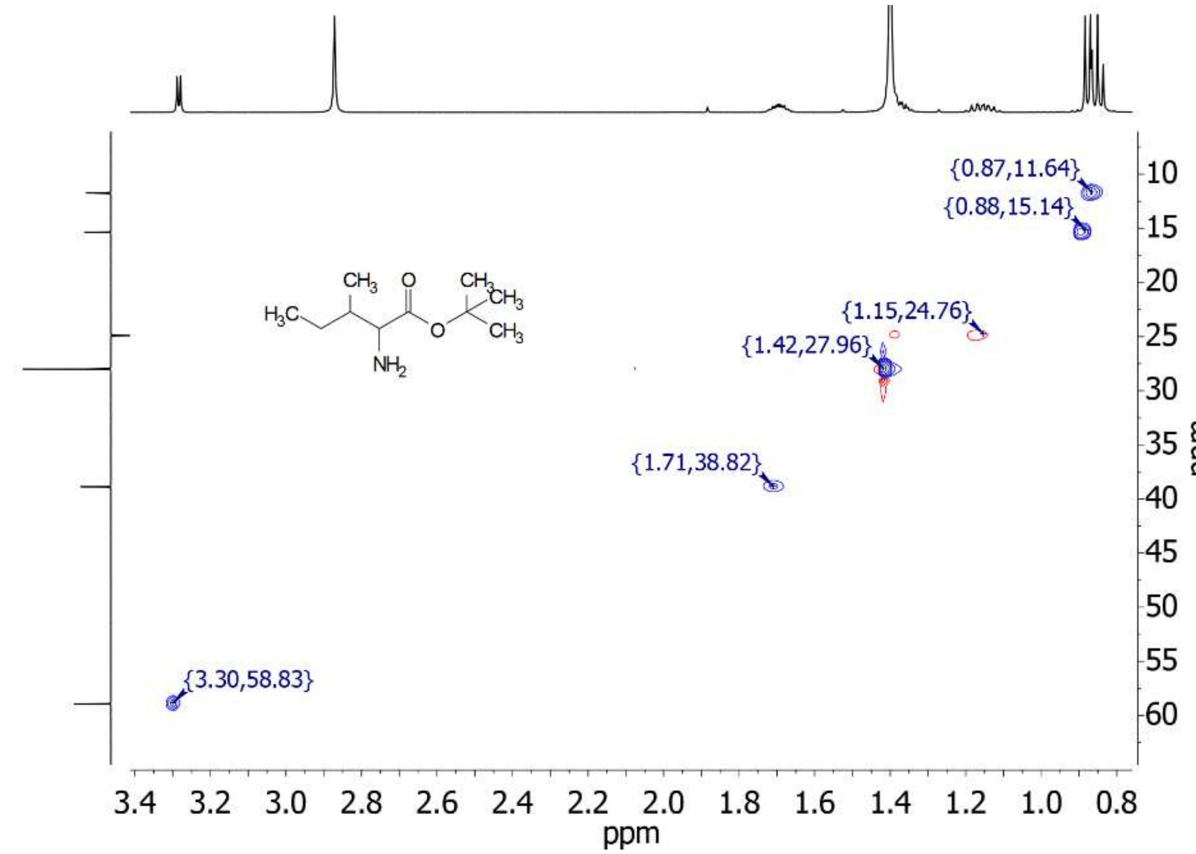

Figure S18. $^1H,^{13}C$ HMQC/HSQC NMR spectrum of *L*-isoleucine *tert*-butyl ester in $CDCl_3$ at 499 MHz.

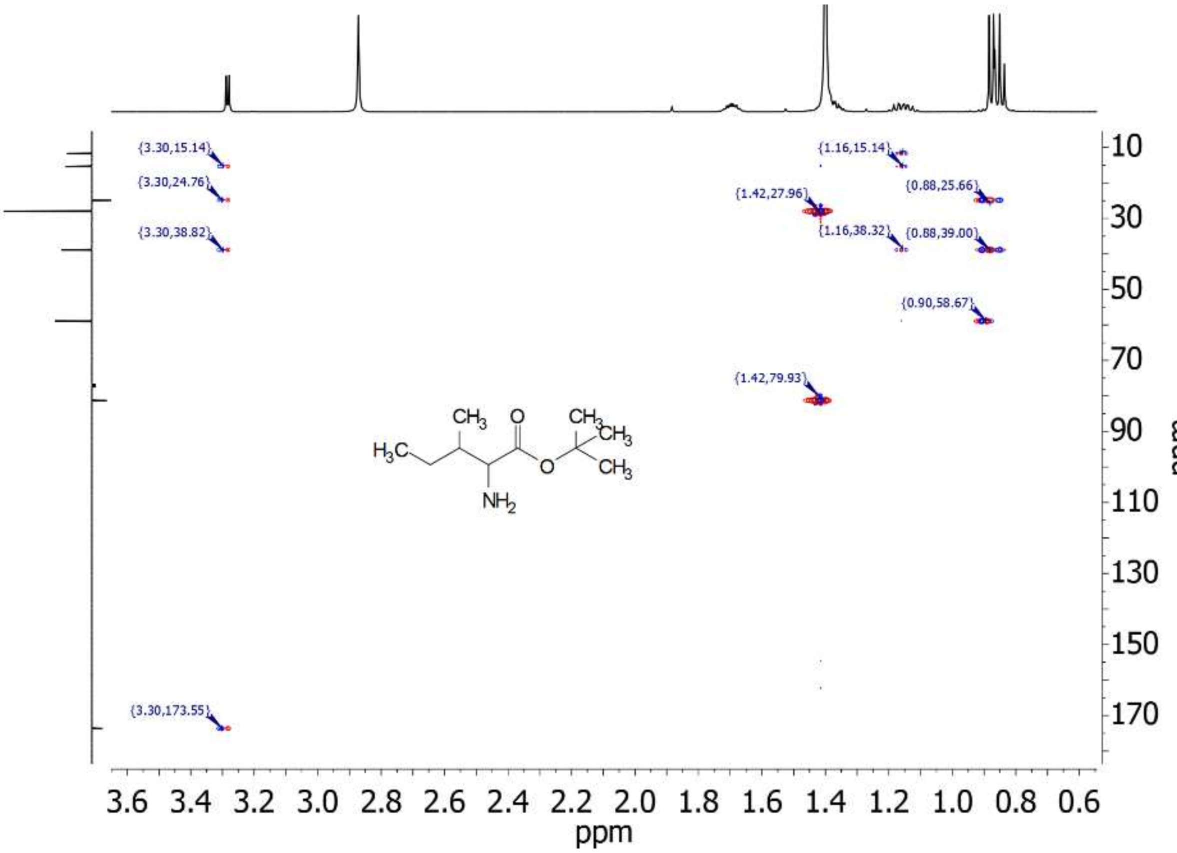

Figure S19. ^{1}H,^{13}C HMBC NMR spectrum of *L*-isoleucine *tert*-butyl ester in $CDCl_3$ at 499 MHz.

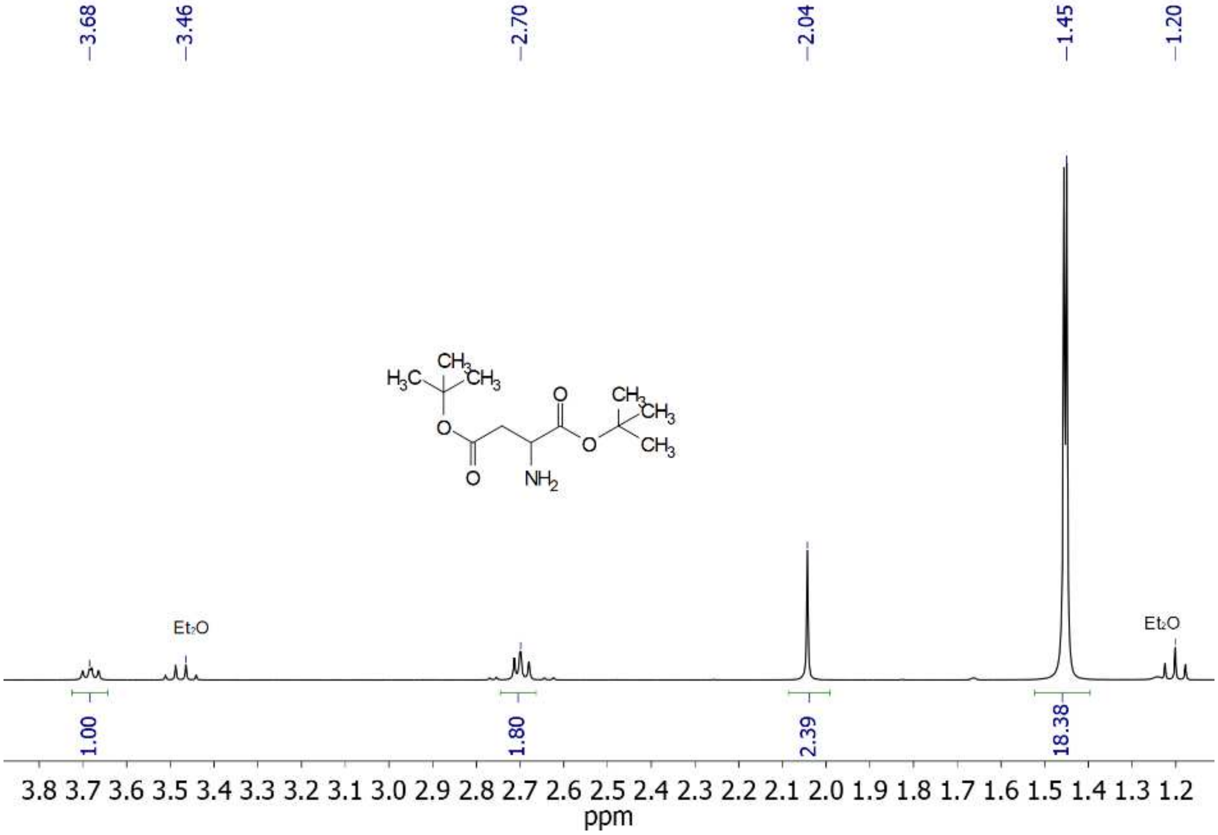

Figure S20. ^{1}H NMR spectrum of *L*-aspartic acid *tert*-butyl ester in $CDCl_3$ at 499 MHz.

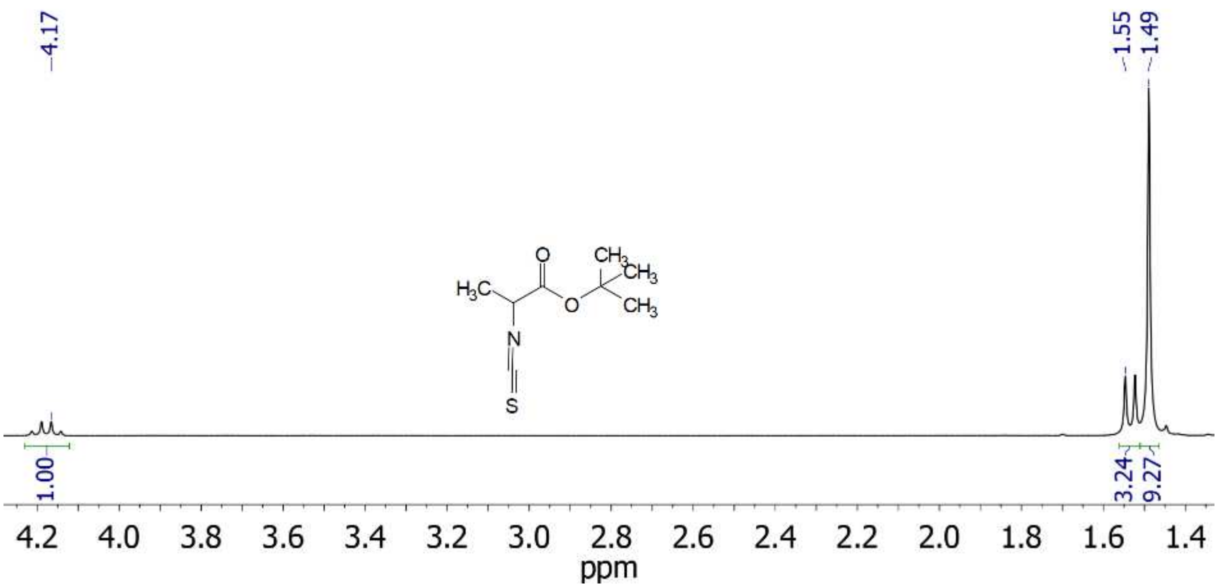

Figure S21. ^{1}H NMR spectrum of (*S*)-*tert*-butyl 2-isothiocyanatopropanoate in $CDCl_3$ at 499 MHz.

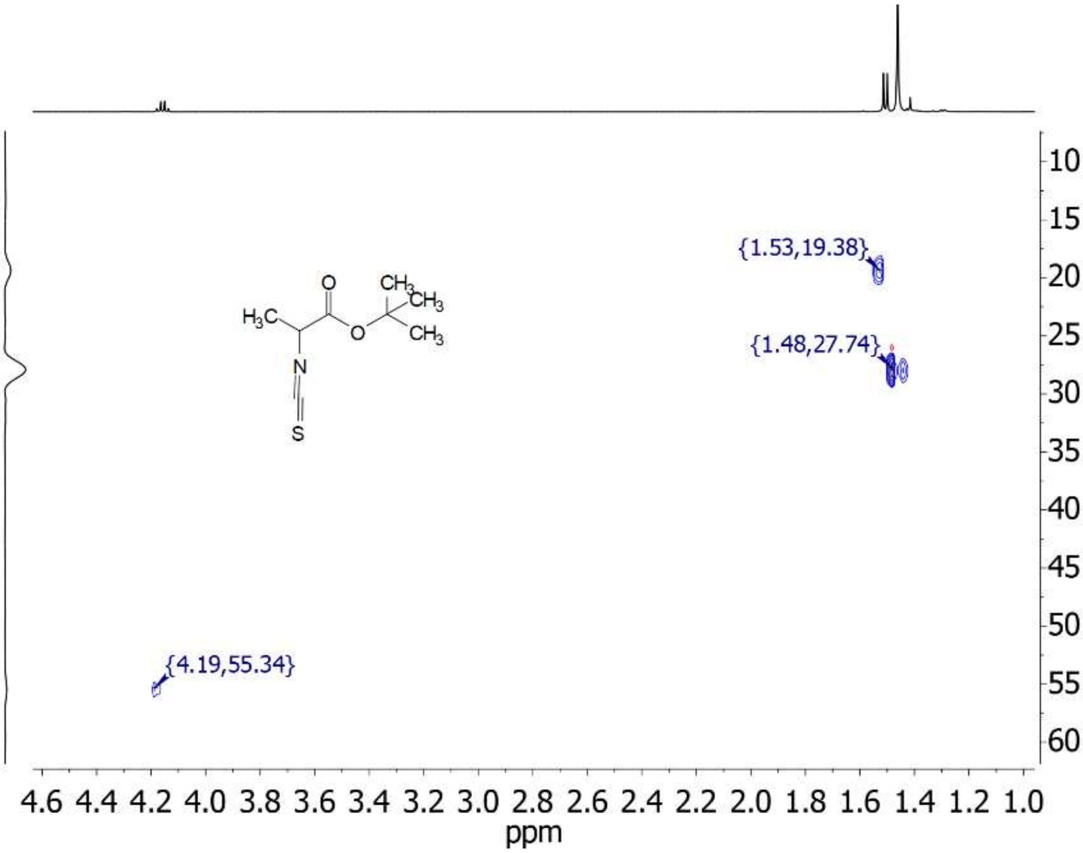

Figure S22. ^{1}H,^{13}C HMQC/HSQC NMR spectrum of (*S*)-*tert*-butyl 2-isothiocyanatopropanoate in $CDCl_3$ at 499 MHz.

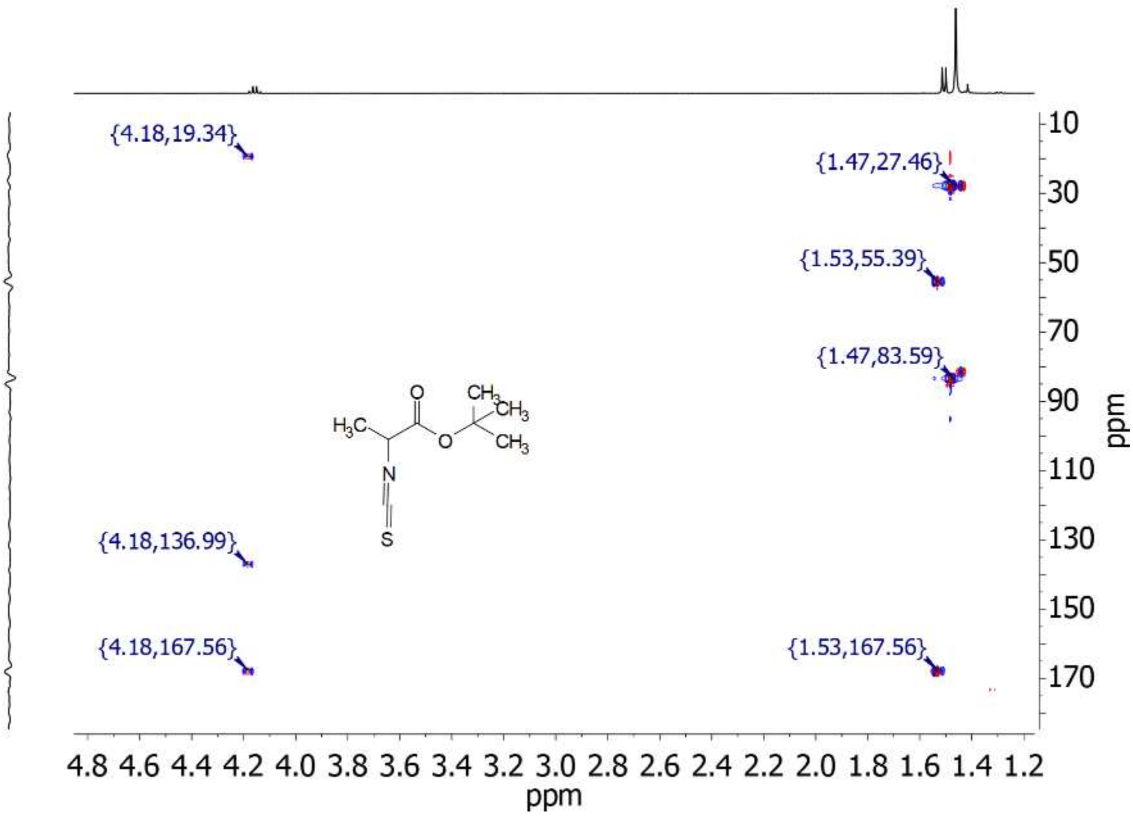

Figure S23. $^{1}H,^{13}C$-HMBC NMR spectrum of (*S*)-*tert*-butyl 2-isothiocyanatopropanoate in $CDCl_3$ at 499 MHz.

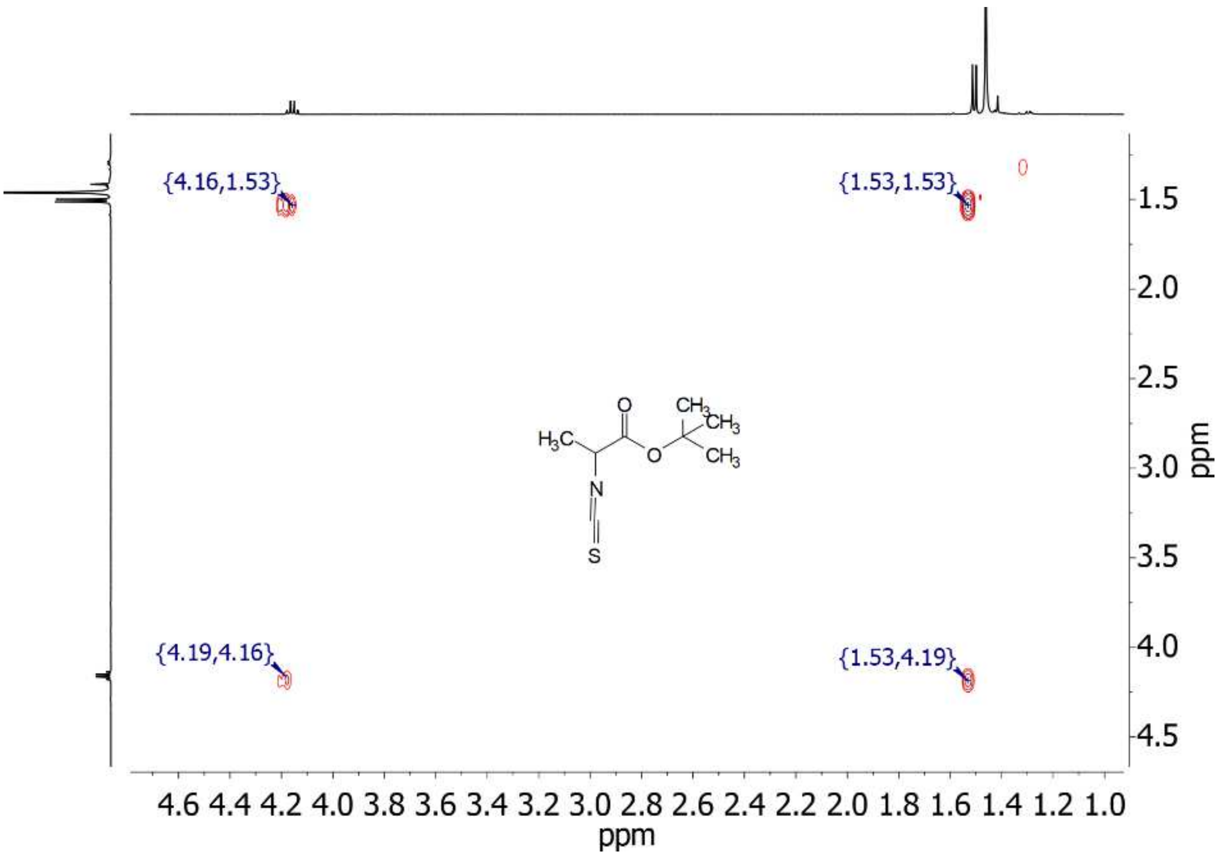

Figure S24. $^{1}H,^{1}H$ COSY NMR spectrum of (*S*)-*tert*-butyl 2-isothiocyanatopropanoate in $CDCl_3$ at 499 MHz.

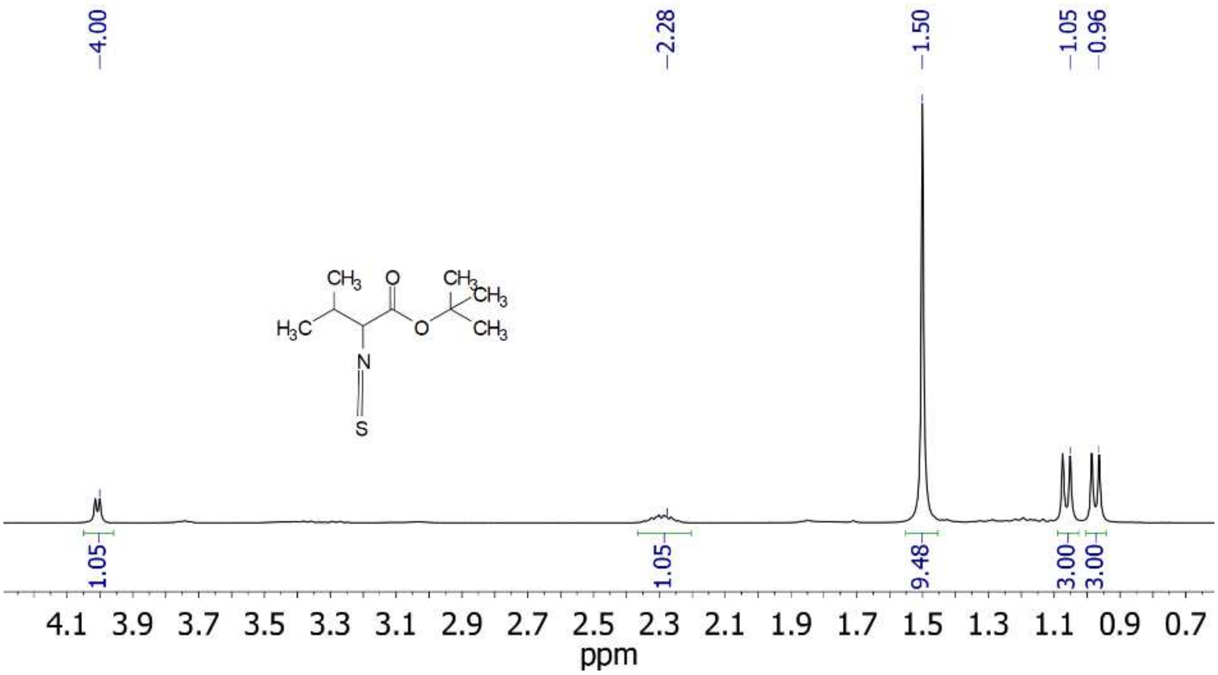

Figure S25. ^{1}H NMR spectrum of (*S*)-*tert*-butyl 2-isothiocyanato-3-metylbutanoate in $CDCl_3$ at 499 MHz.

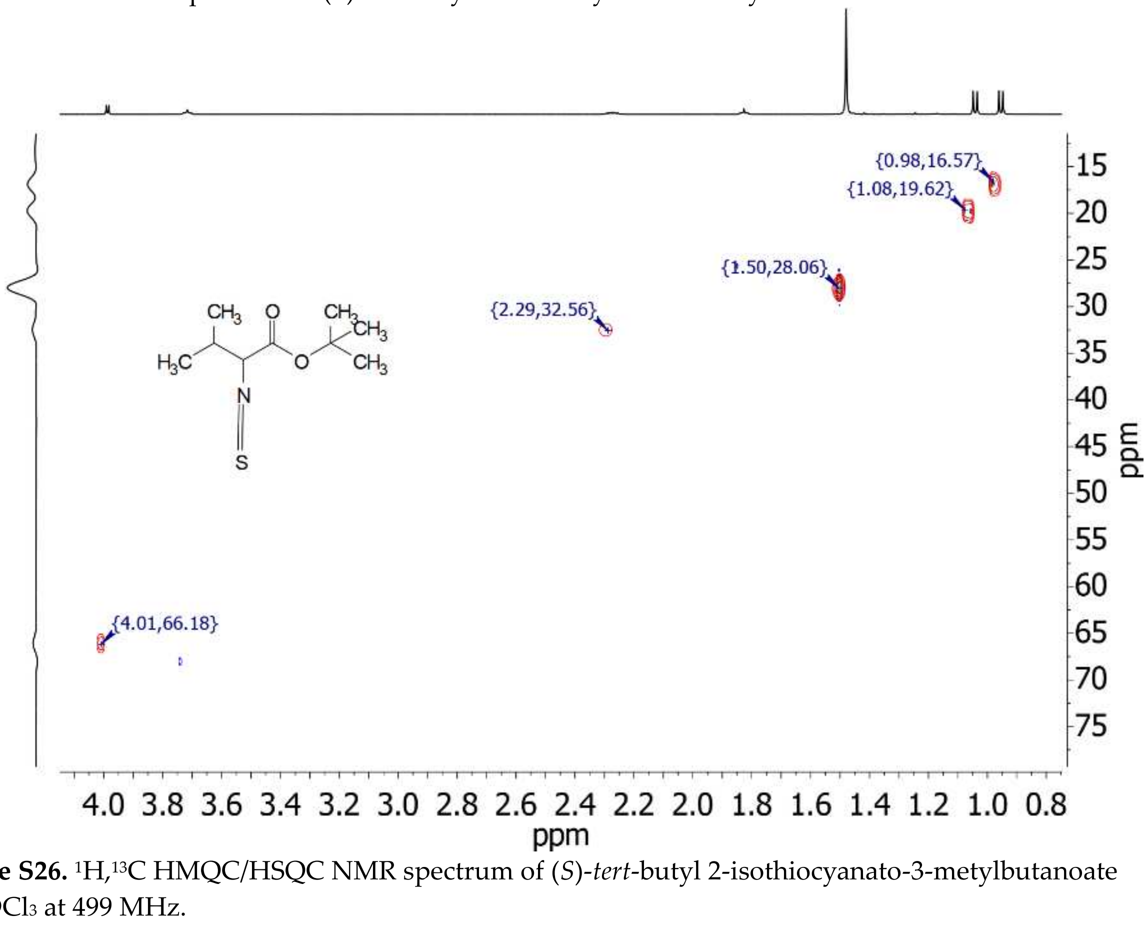

Figure S26. ^{1}H,^{13}C HMQC/HSQC NMR spectrum of (*S*)-*tert*-butyl 2-isothiocyanato-3-metylbutanoate in $CDCl_3$ at 499 MHz.

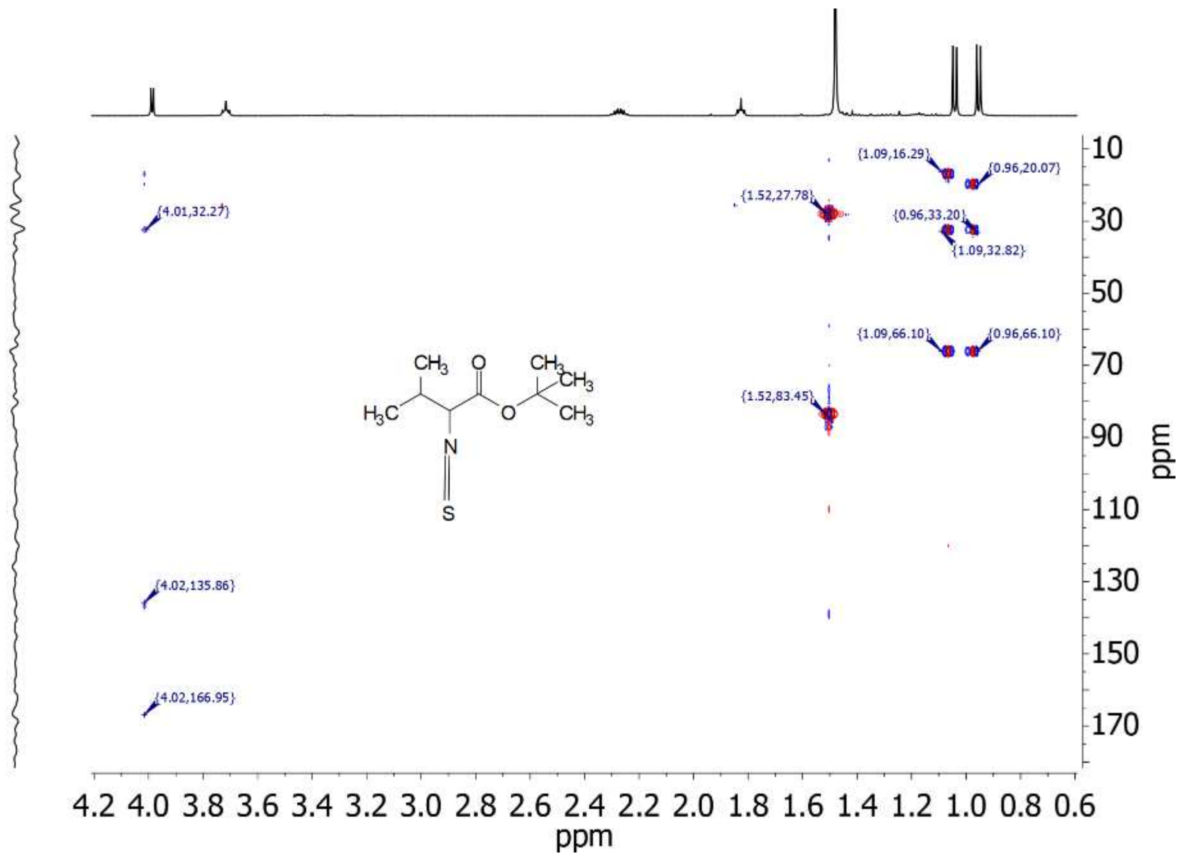

Figure S27. $^{1}H,^{13}C$ HMBC NMR spectrum of (*S*)-*tert*-butyl 2-isothiocyanato-3-metylbutanoate in $CDCl_3$ at 499 MHz.

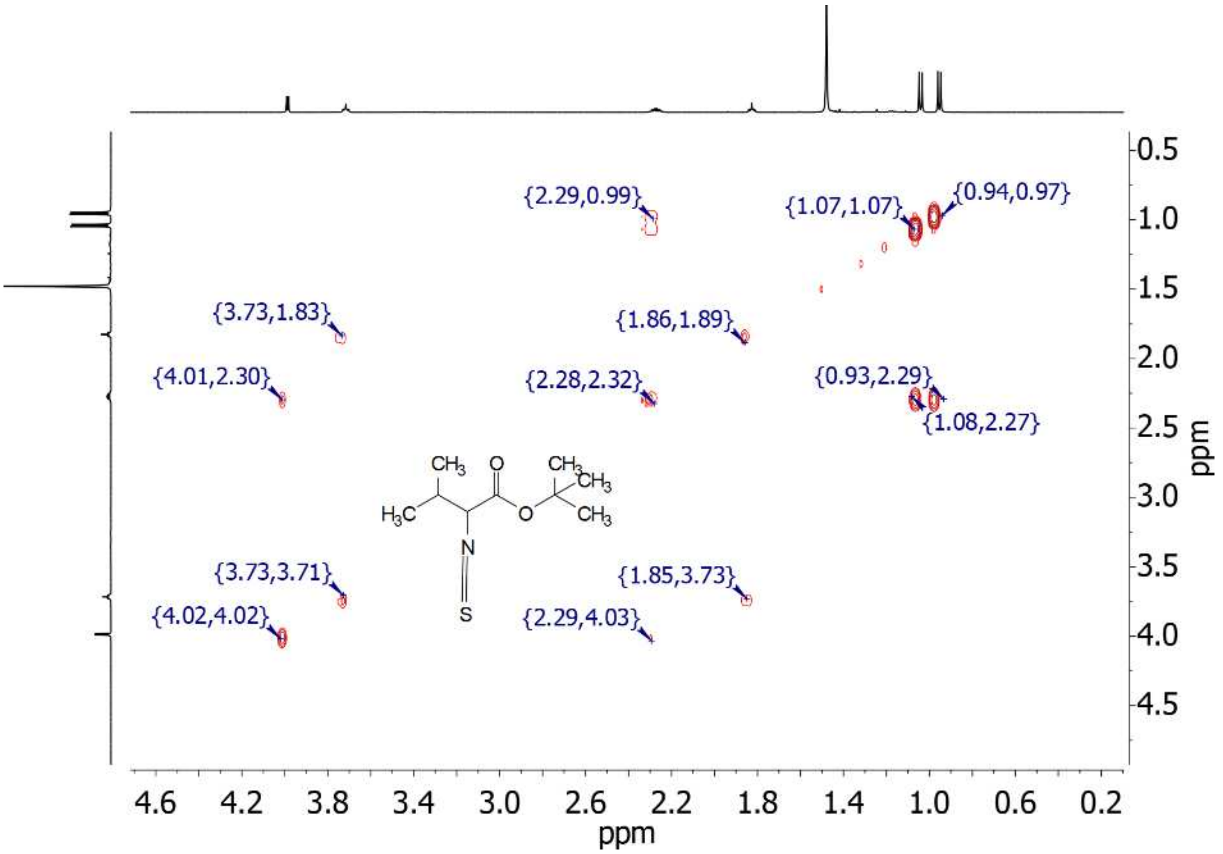

Figure S28. $^{1}H,^{1}H$ COSY-NMR spectrum of (*S*)-*tert*-butyl 2-isothiocyanato-3-metylbutanoate in $CDCl_3$ at 499 MHz.

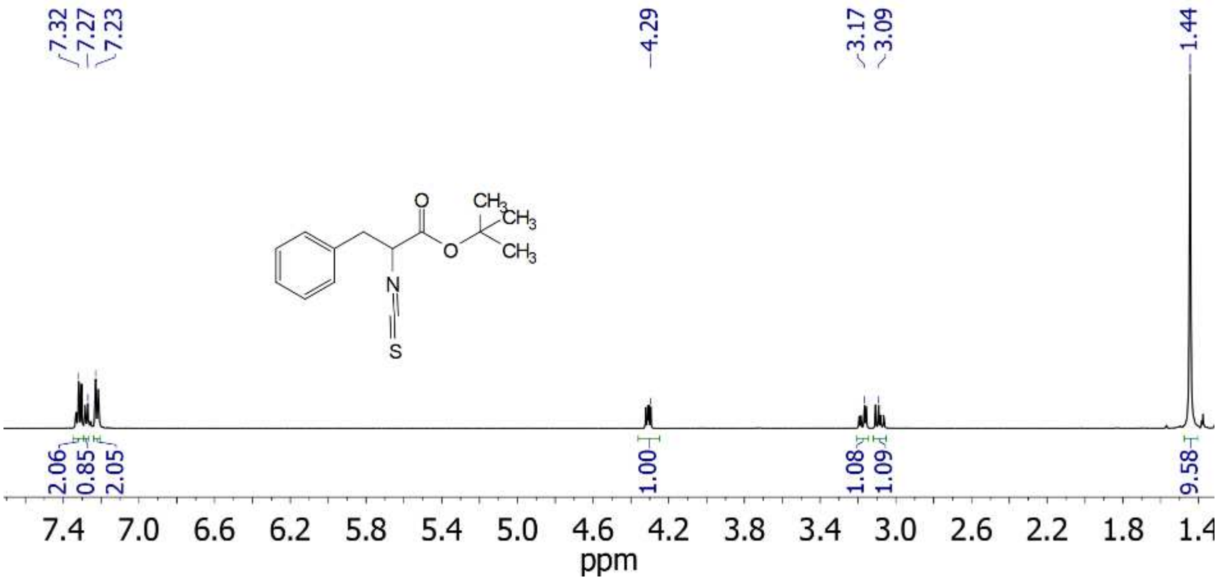

Figure S29. ^{1}H NMR spectrum of (*S*)-*tert*-butyl 2-isothiocyanato-3-phenylpropanoate in $CDCl_3$ at 499 MHz.

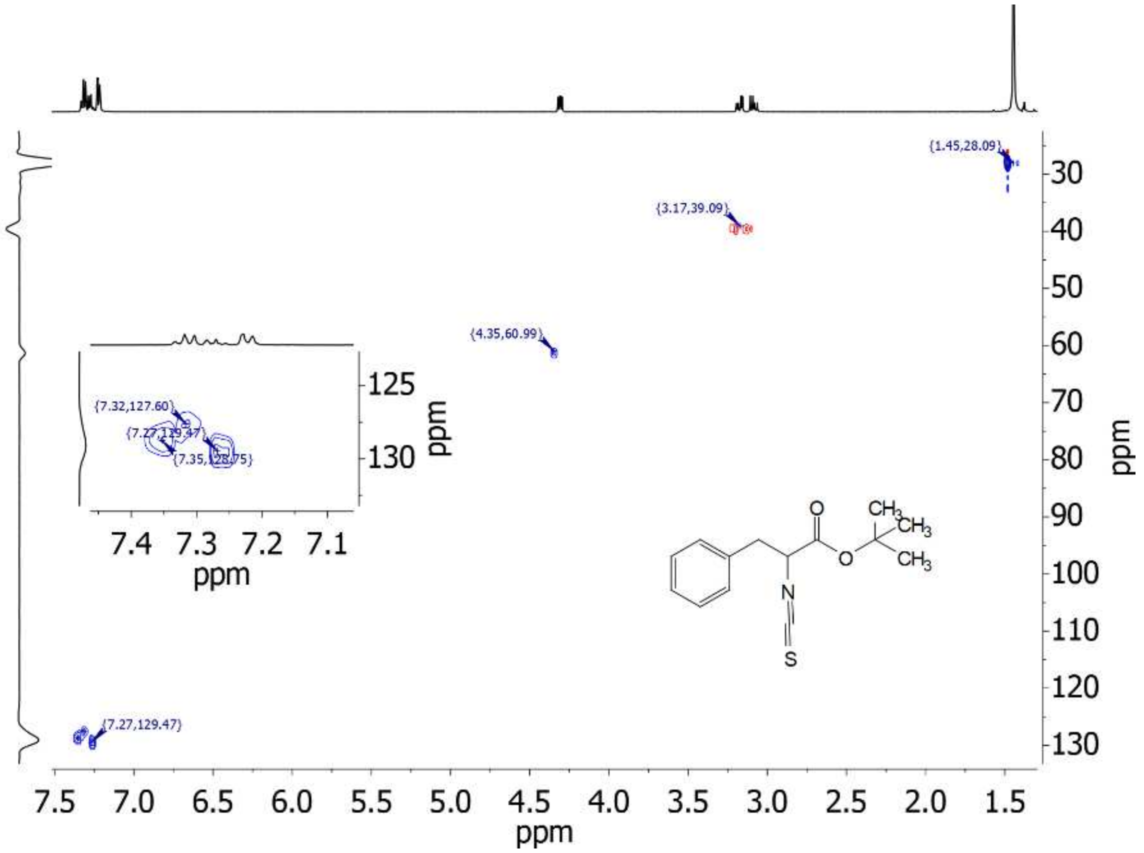

Figure S30. ^{1}H,^{13}C HMQC/HSQC NMR spectrum of (*S*)-*tert*-butyl 2-isothiocyanato-3-phenyl propanoate in $CDCl_3$ at 499 MHz.

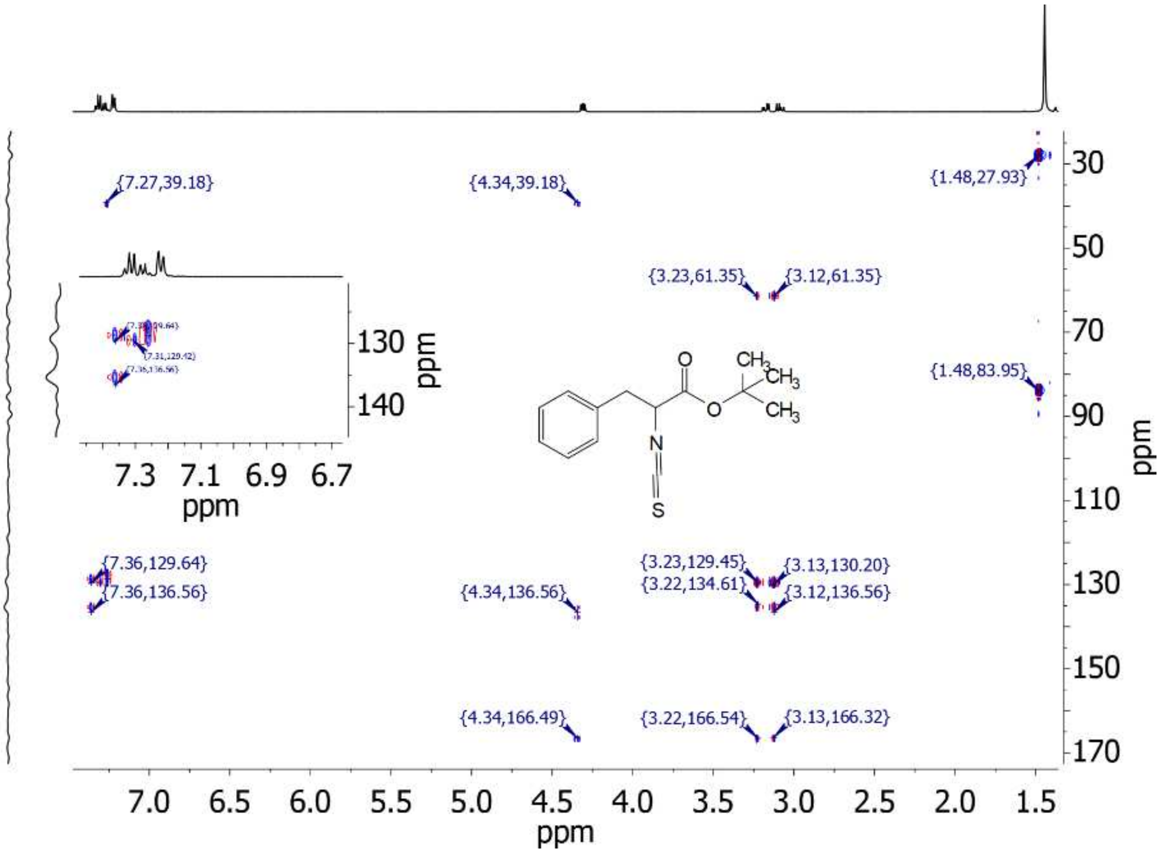

Figure S31. $^{1}H,^{13}C$ HMBC NMR spectrum of (*S*)-*tert*-butyl 2-isothiocyanato-3-phenylpropanoate in $CDCl_3$ at 499 MHz.

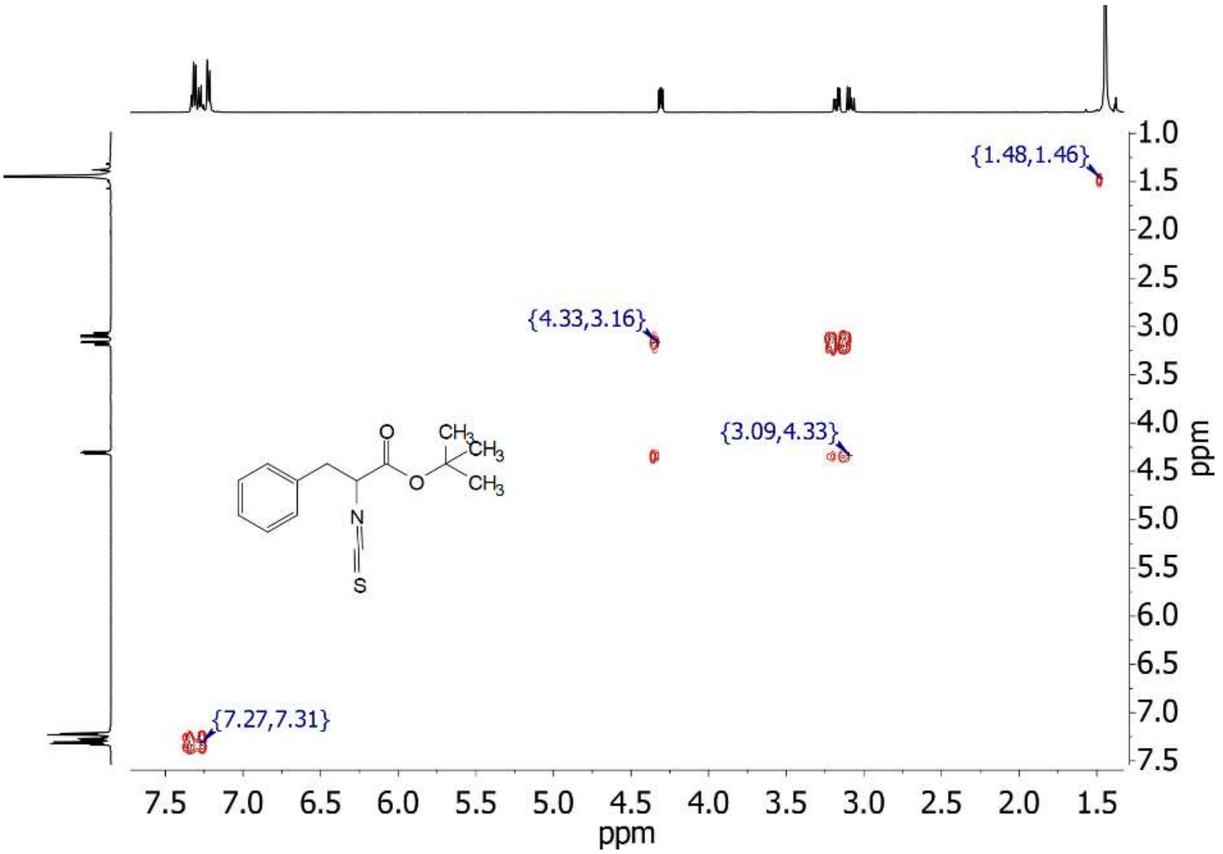

Figur*e* S32. $^{1}H,^{1}H$ COSY NMR spectrum of (*S*)-*tert*-butyl 2-isothiocyanato-3-phenylpropanoate in $CDCl_3$ at 499 MHz.

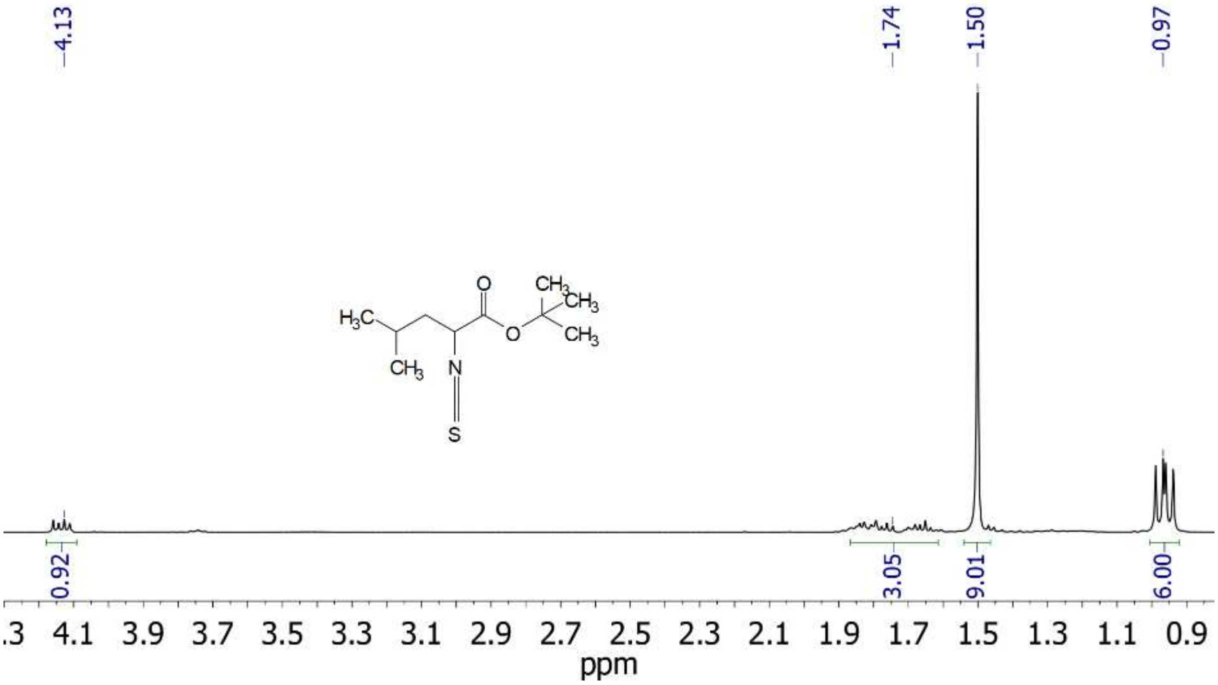

Figure S33. ^{1}H NMR spectrum of (*S*)-*tert*-butyl 2-isothiocyanato-4-methylpentanoate in $CDCl_3$ at 300 MHz.

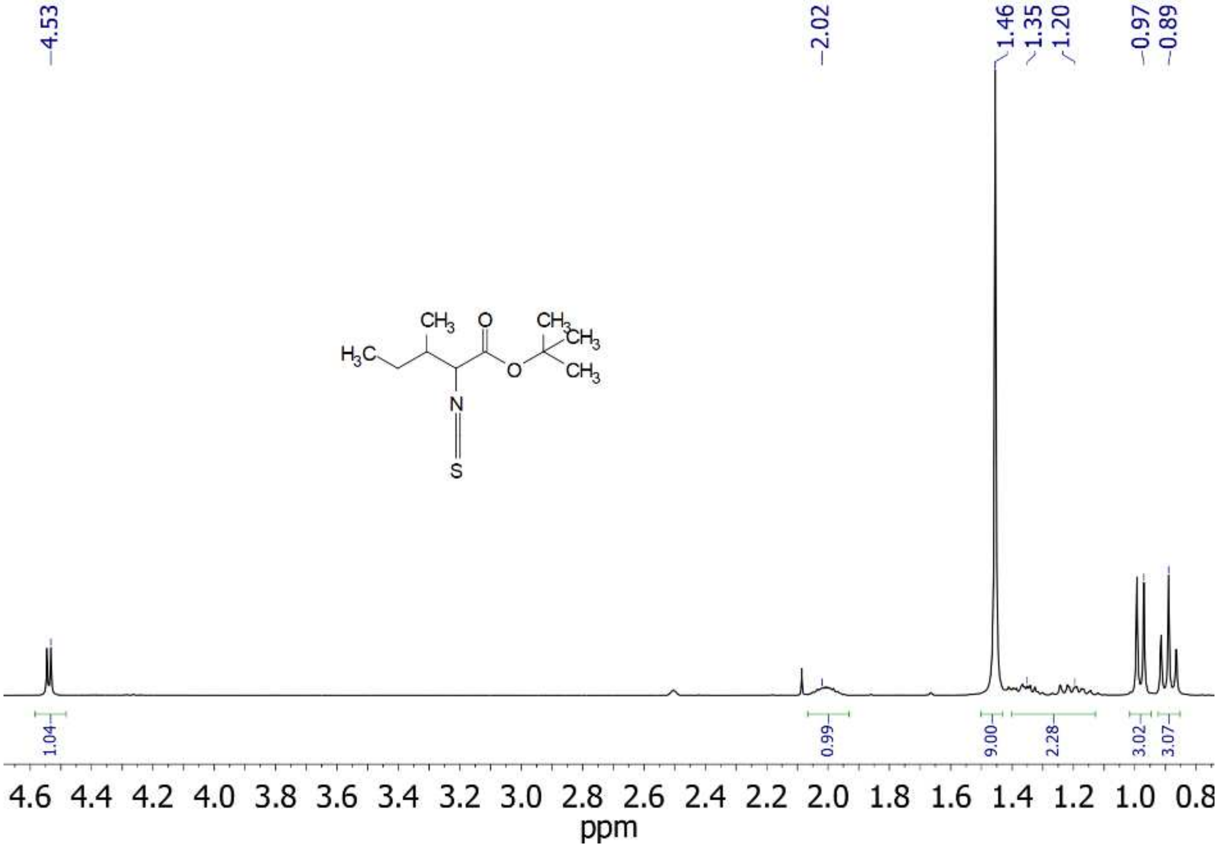

Figure S34. ^{1}H NMR spectrum of (*S*)-*tert*-butyl 2-isothiocyanato-3-methylpentanoate in $CDCl_3$ at 499 MHz.

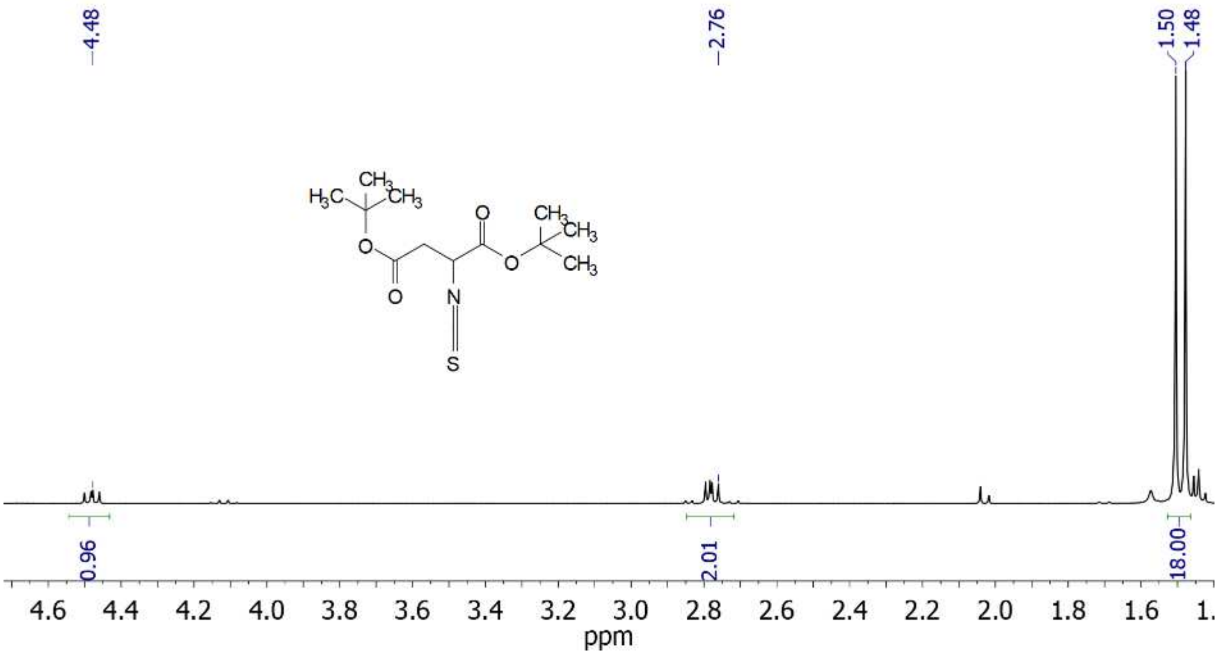

Figure S35. ^{1}H NMR spectrum of (*S*)-di-*tert*-butyl 2-isothiocyanatobutanedioate in $CDCl_3$ at 300 MHz.

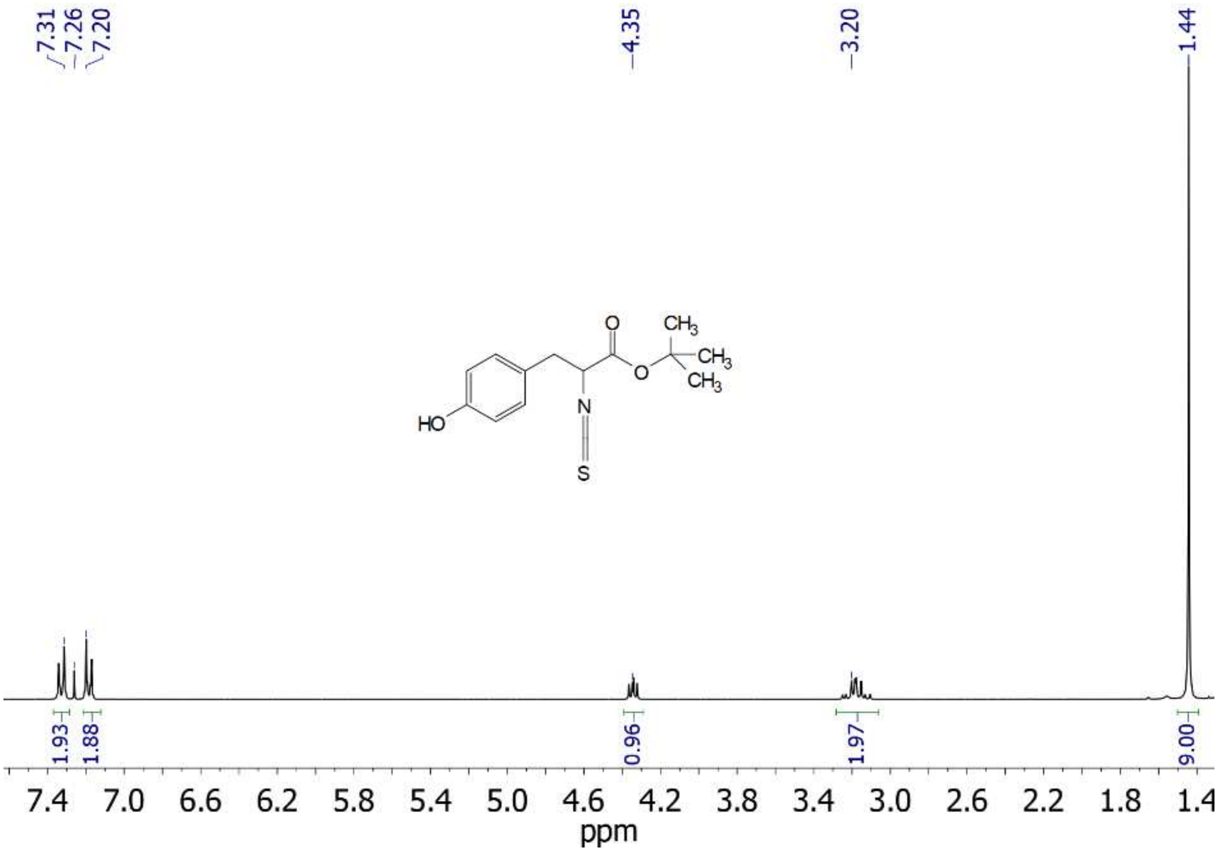

Figure S36. ^{1}H NMR spectrum of *tert*-butyl 3-(4-hydroxyphenyl)-2-isothiocyanatopropanoate in $CDCl_3$ at 499 MHz.

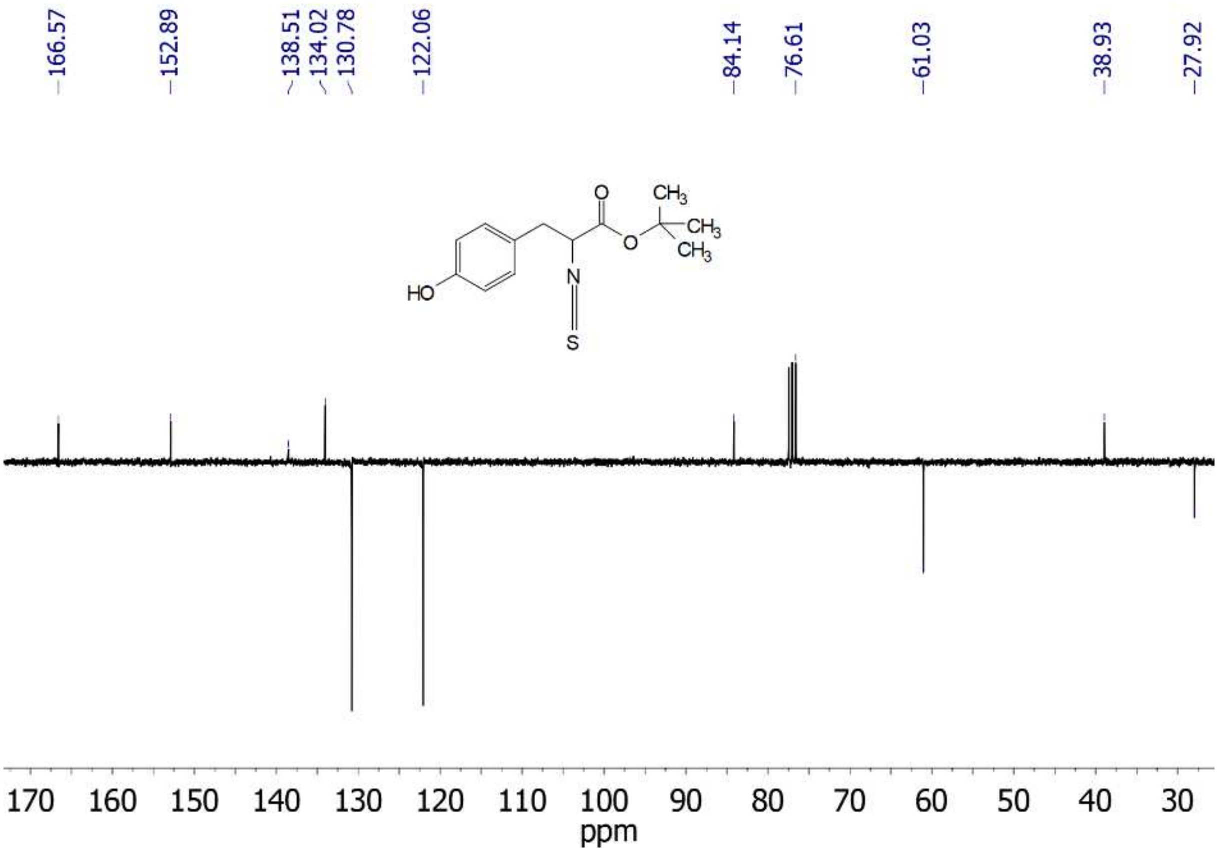

Figure S37. ^{13}C DEPTQ NMR spectrum of *tert*-butyl 3-(4-hydroxyphenyl)-2-isothiocyanatopropanoate in $CDCl_3$ at 499 MHz.

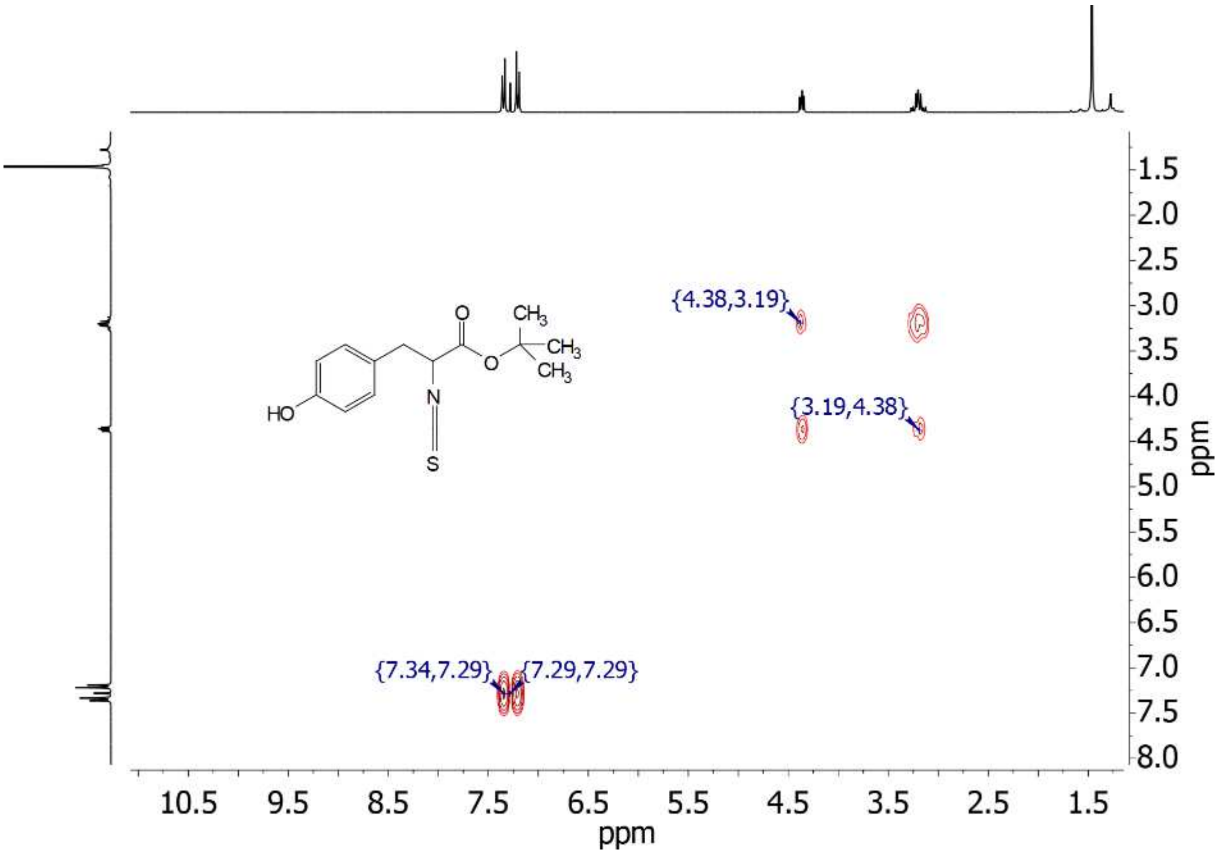

Figure S38. $^{1}H,^{1}H$ COSY NMR spectrum of *tert*-butyl 3-(4-hydroxyphenyl)-2-isothiocyanato-propanoate in $CDCl_3$ at 499 MHz.

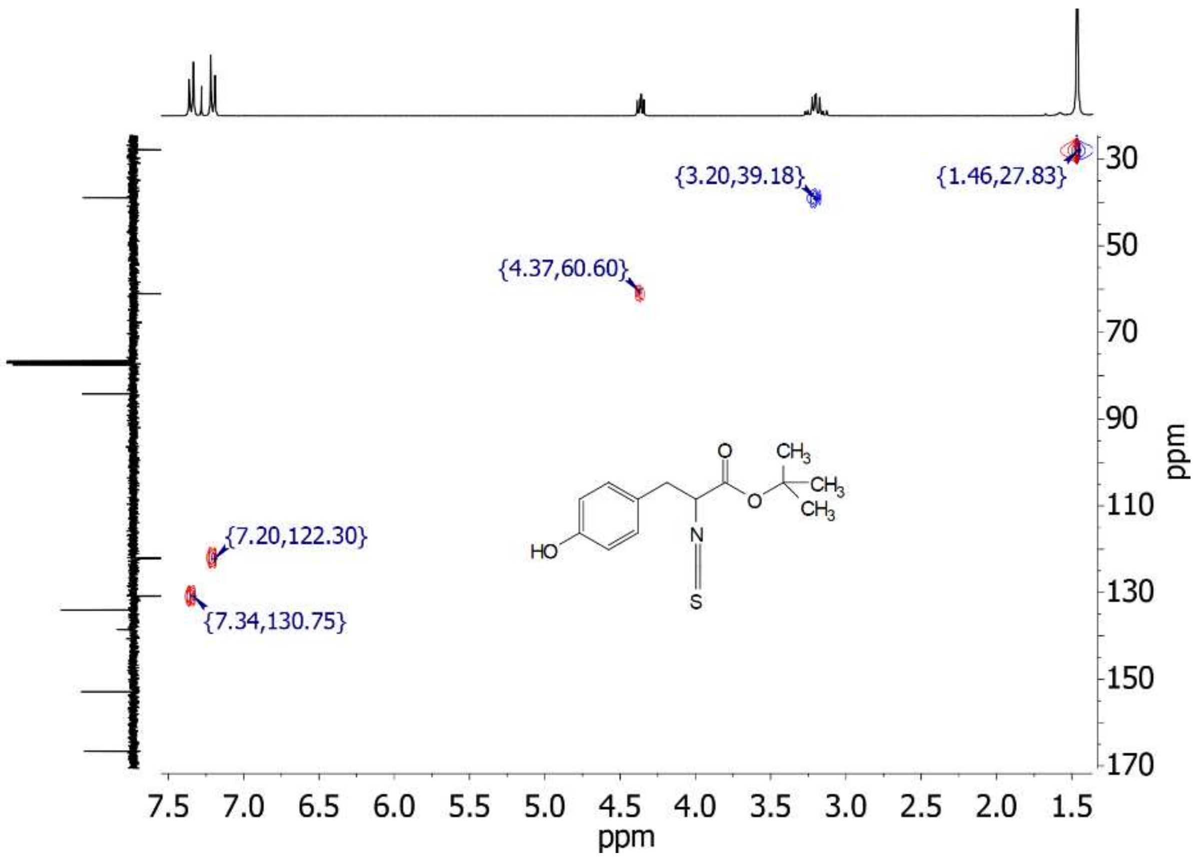

Figure S39. $^{1}H,^{13}C$ HMQC/HSQC NMR spectrum of *tert*-butyl 3-(4-hydroxyphenyl)-2-isothiocyanato-propanoatein $CDCl_3$ at 499 MHz.

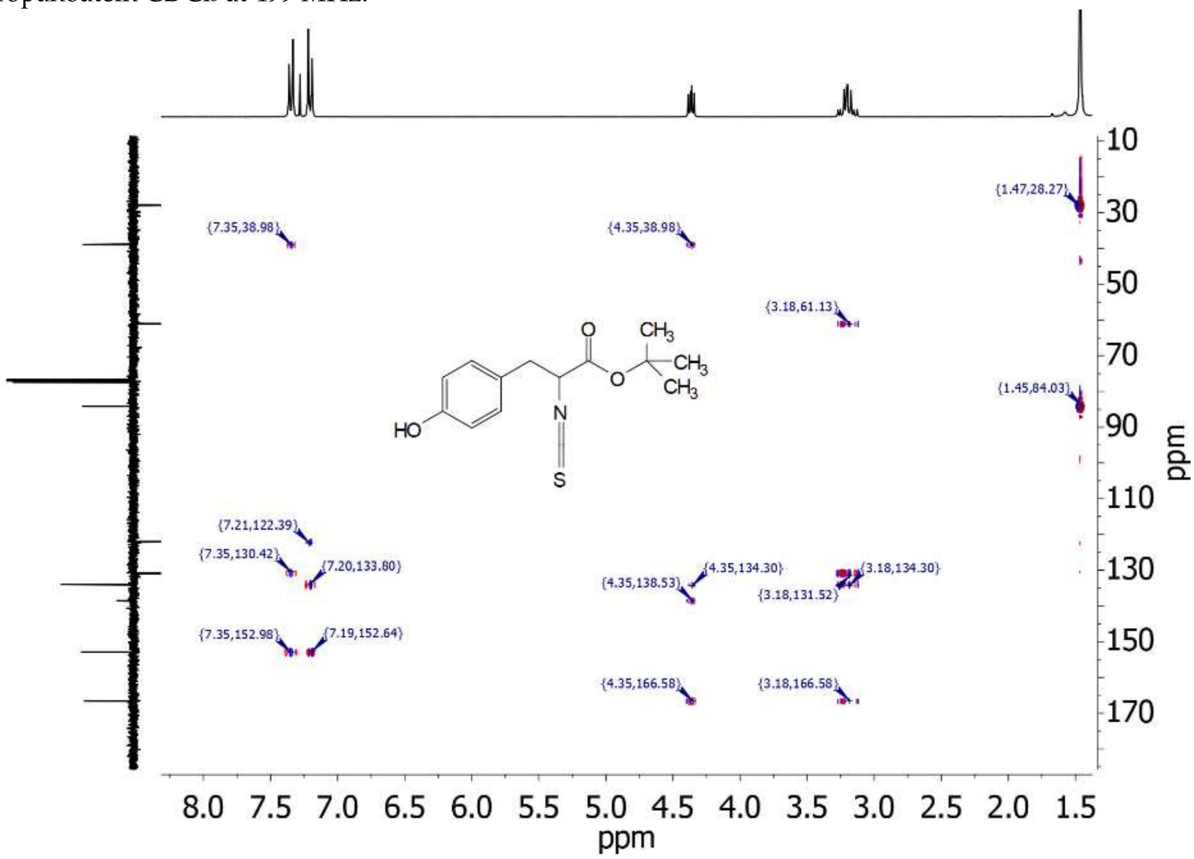

Figure S40. $^{1}H,^{13}C$ HMBC NMR spectrum of *tert*-butyl 3-(4-hydroxyphenyl)-2-isothiocyanato-propanoate in $CDCl_3$ at 499 MHz.

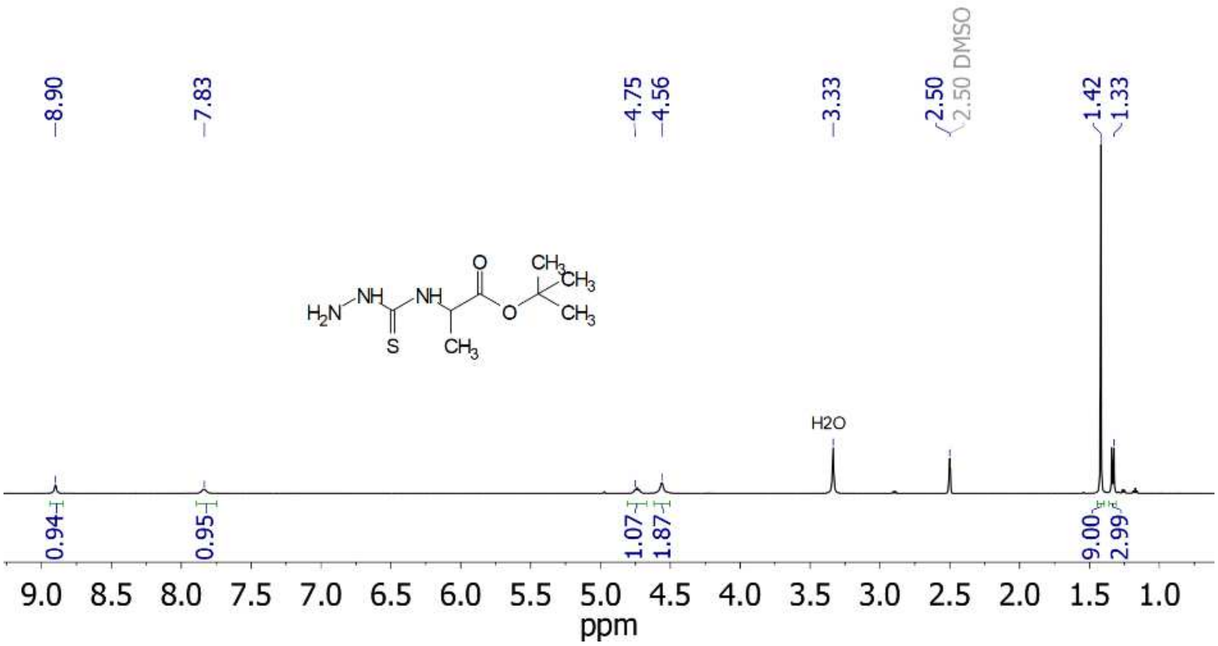

Figure S41. ^{1}H NMR spectrum of 4-((*S*)-*tert*-butylpropanoate)thiosemicarbazide in DMSO-*d*$_6$ at 499 MHz.

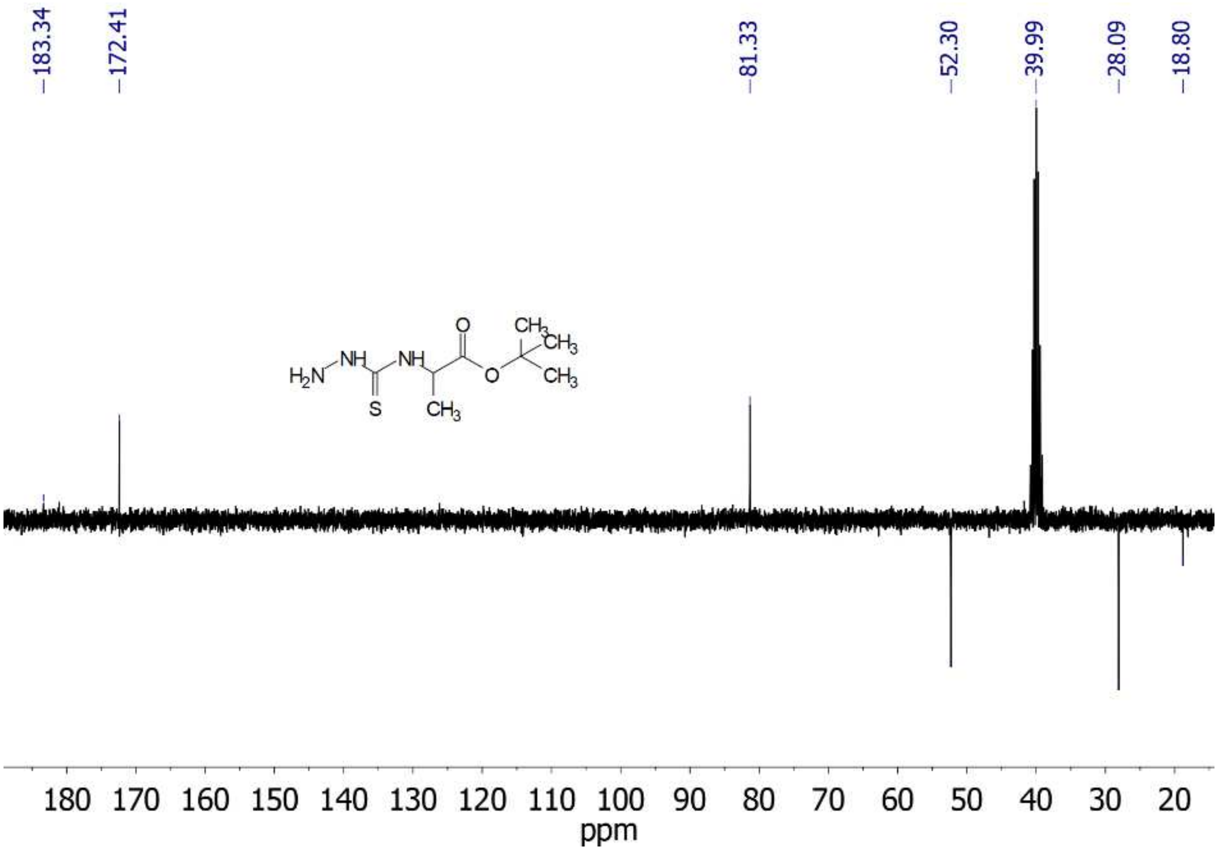

Figure S42. ^{13}C DEPTQ NMR spectrum of 4-((*S*)-*tert*-butylpropanoate)thiosemicarbazide in DMSO-*d*$_6$ at 300 MHz.

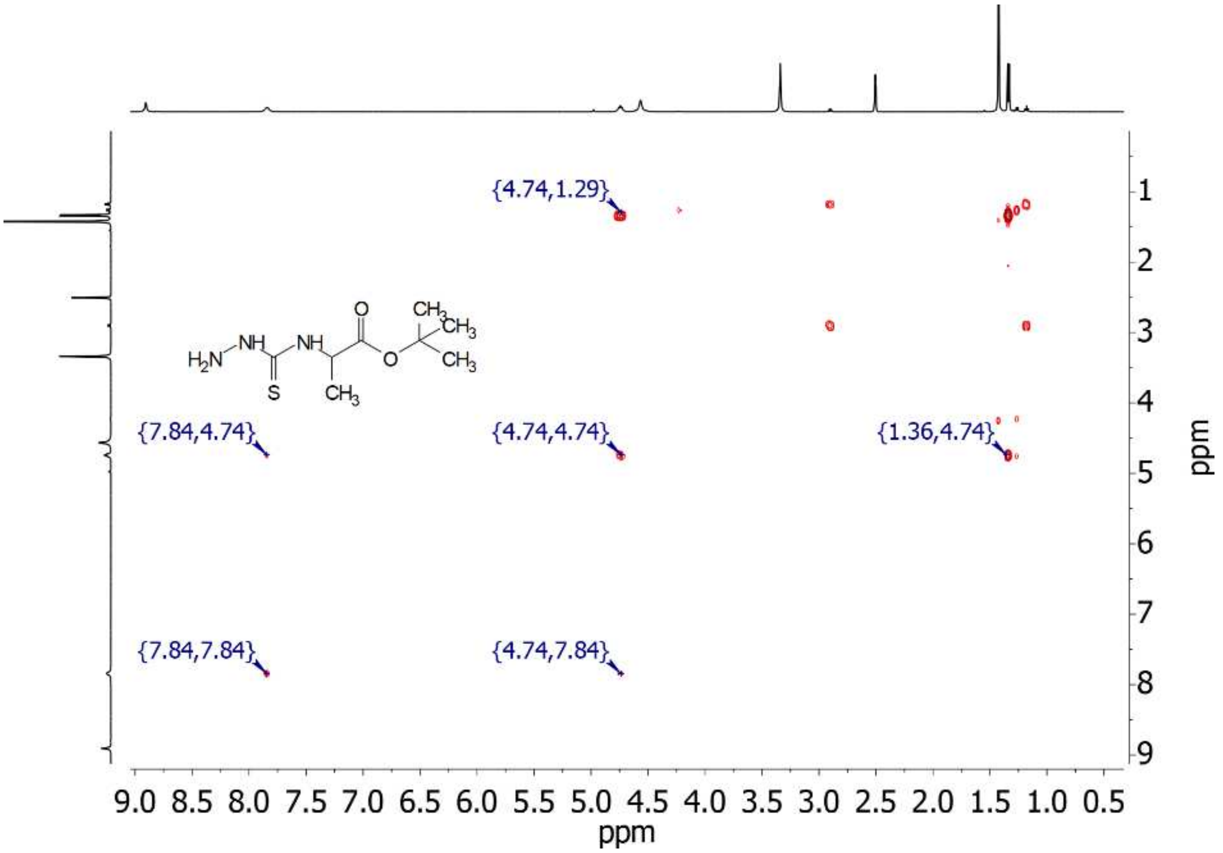

Figure S43. $^{1}H,^{1}H$ COSY NMR spectrum of 4-((*S*)-*tert*-butylpropanoate)thiosemicarbazide in DMSO-d_6 at 499 MHz.

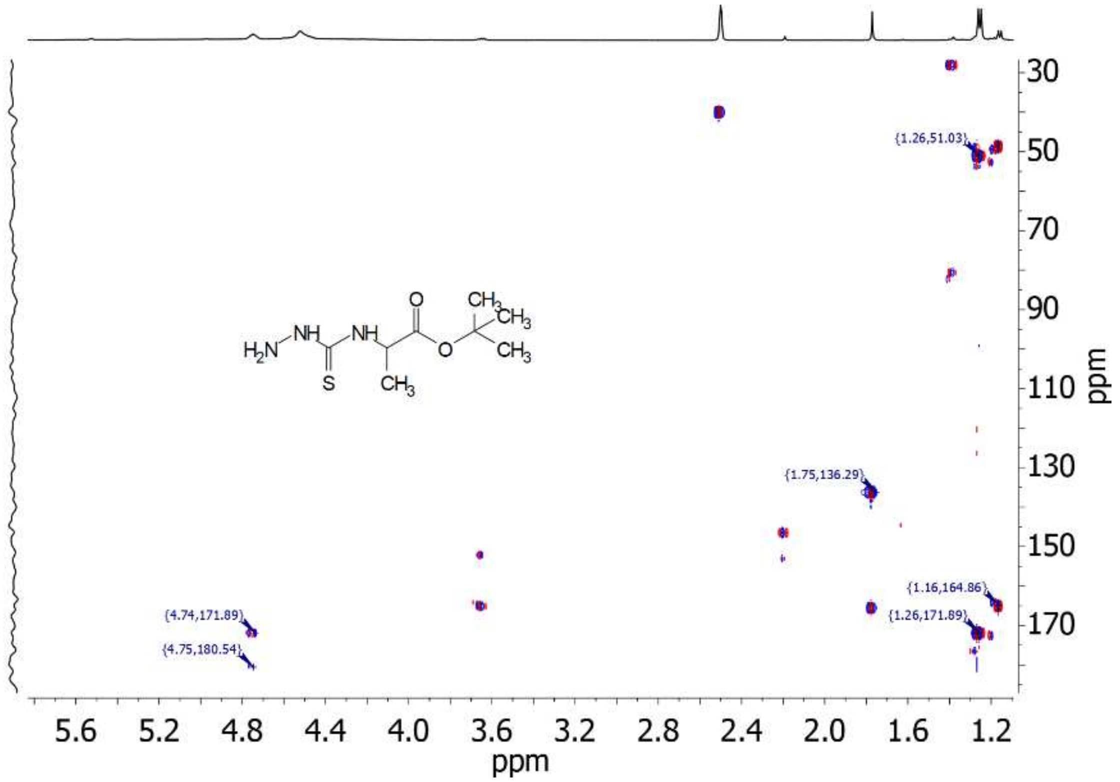

Figure S44. $^{1}H,^{13}C$ HMBC NMR spectrum of 4-((*S*)-*tert*-butylpropanoate)thiosemicarbazide in DMSO-d_6 at 499 MHz.

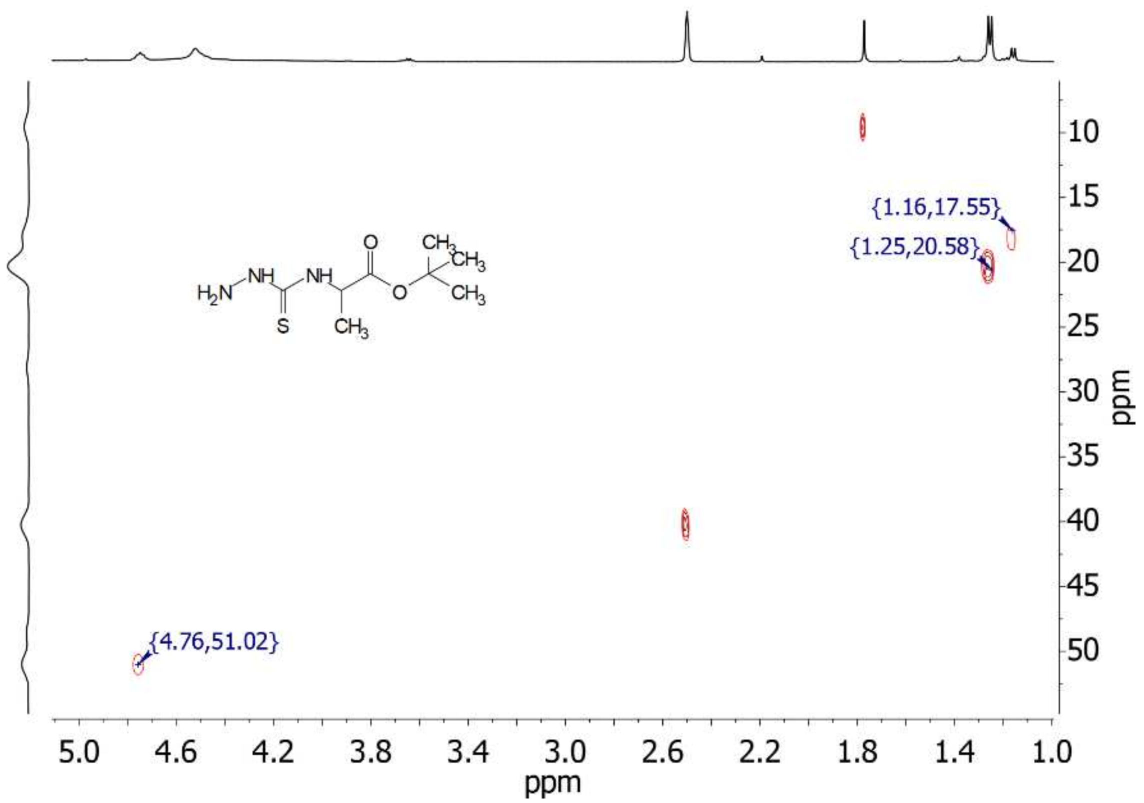

Figure S45. $^{1}H,^{13}C$ HMQC/HSQC NMR spectrum of 4-((*S*)-*tert*-butylpropanoate)thiosemicarbazide in DMSO-d_6 at 499 MHz.

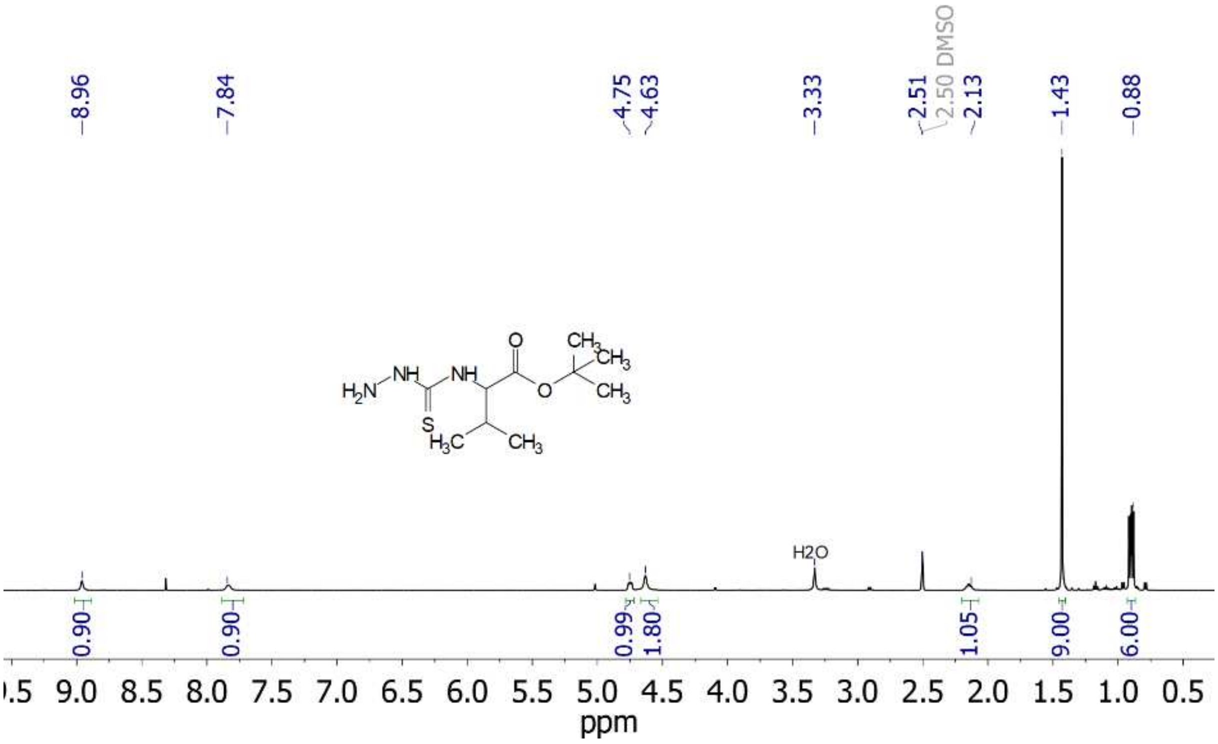

Figure S46. ^{1}H NMR spectrum of 4-((*S*)-*tert*-butyl-3-metylbutanoate)thiosemicarbazide in DMSO-d_6 at 499 MHz.

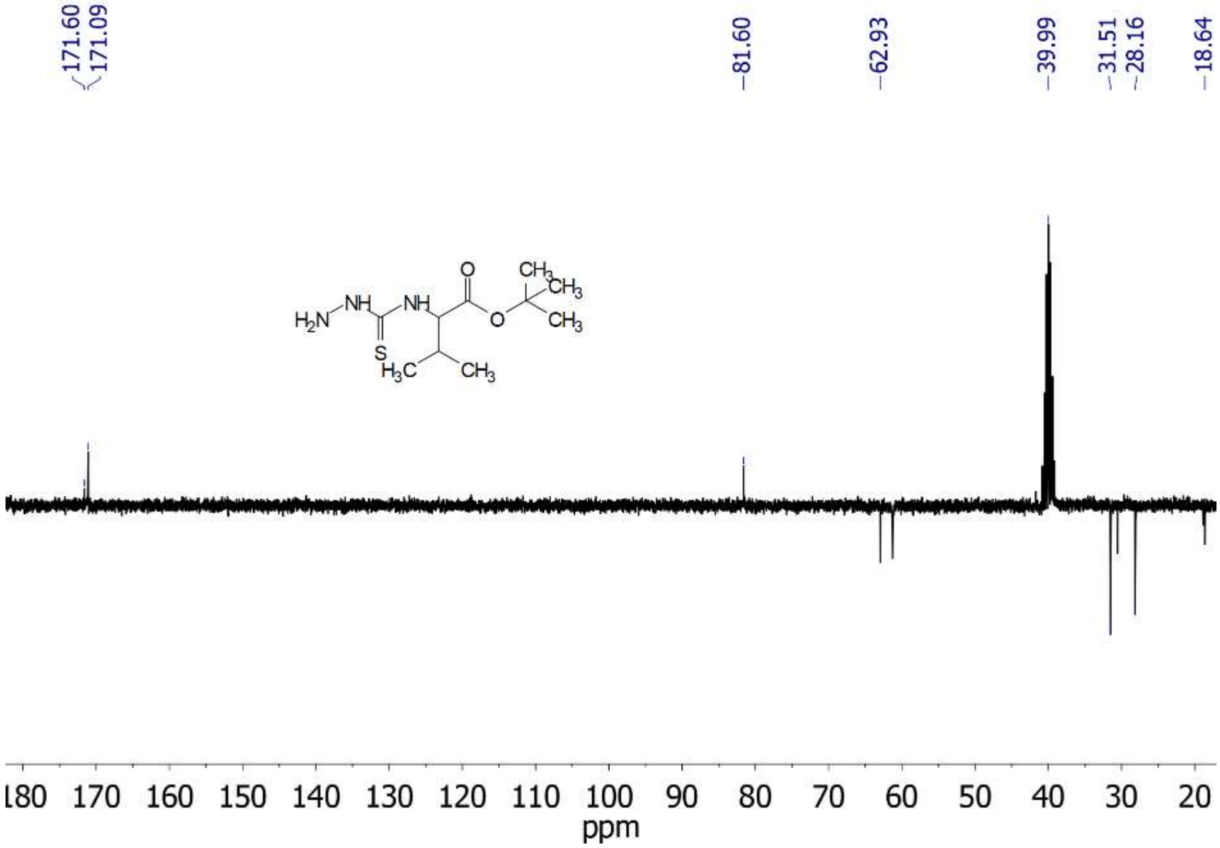

Figure S47. ^{13}C DEPTQ NMR spectrum of 4-((*S*)-*tert*-butyl-3-metylbutanoate)thiosemicarbazide in DMSO-d_6 at 300 MHz.

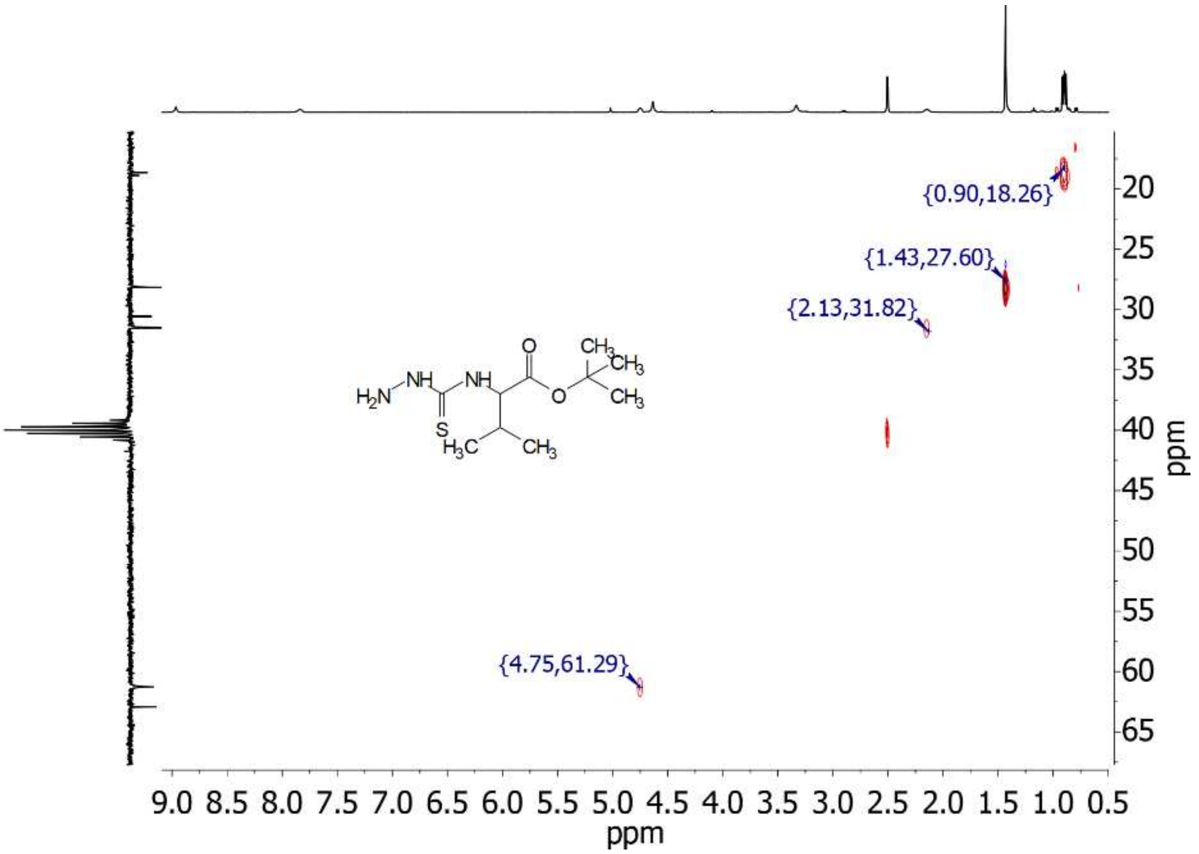

Figure S48. ^{1}H,^{13}C HMQC/HSQC NMR spectrum of 4-((*S*)-*tert*-butyl-3-metylbutanoate)thiosemicarbazide DMSO-d_6 at 499 MHz.

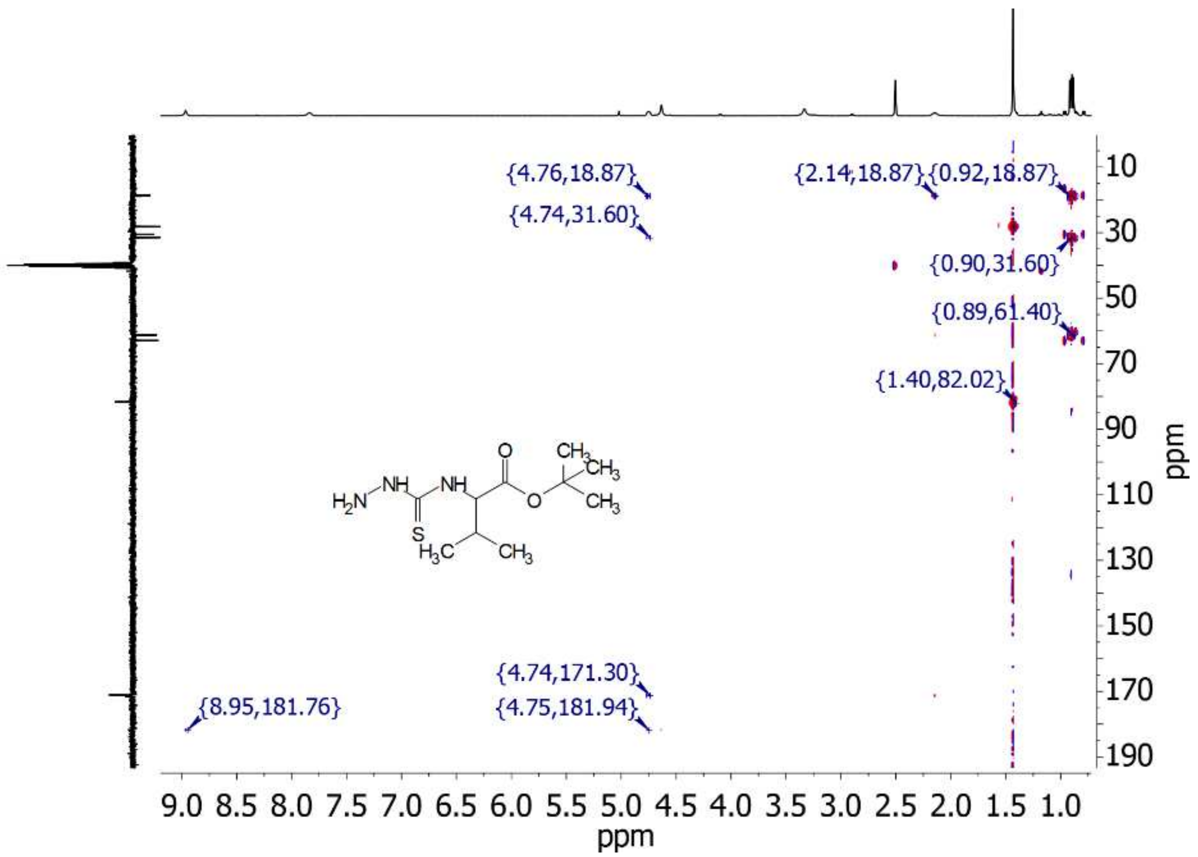

Figure S49. ^{1}H,^{13}C HMBC NMR spectrum of 4-((*S*)-*tert*-butyl-3-metylbutanoate)thiosemicarbazide in DMSO-d_6 at 499 MHz.

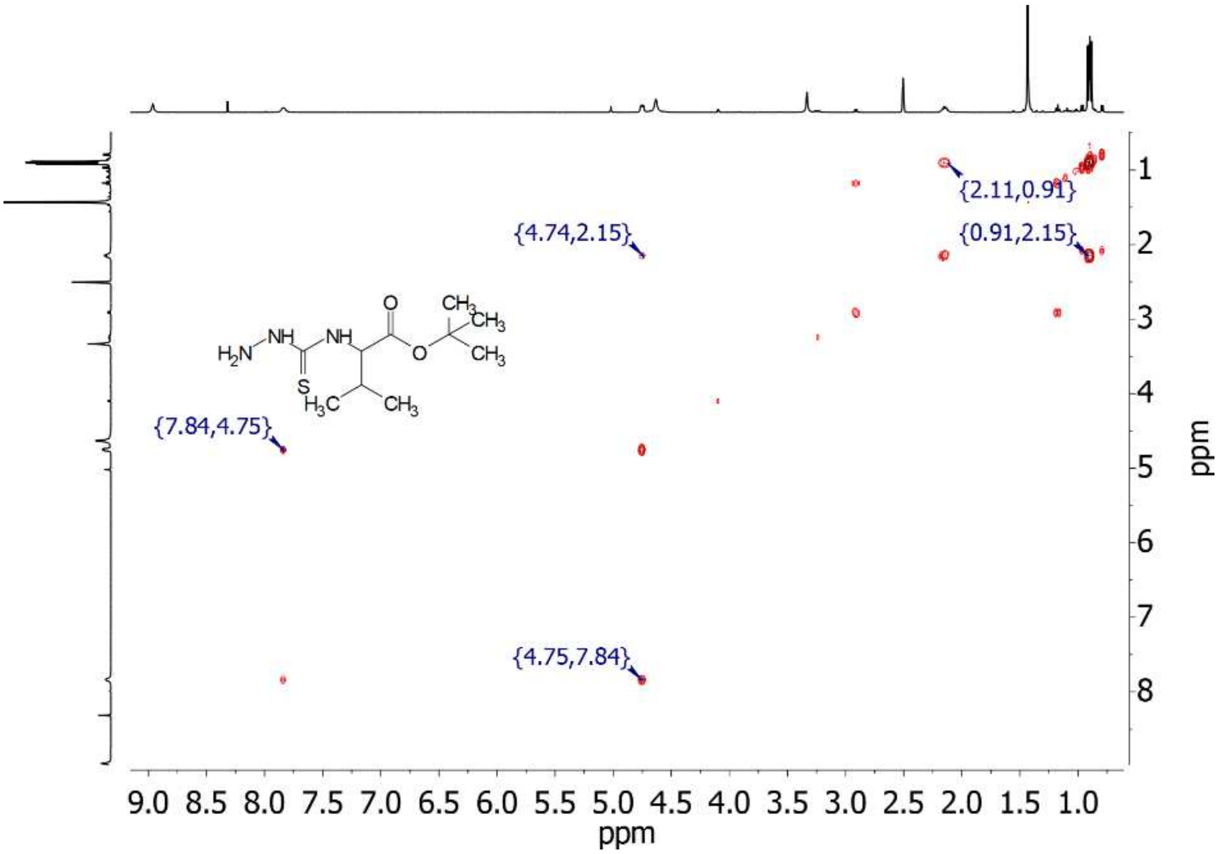

Figure S50. ^{1}H,^{1}H COSY NMR spectrum of 4-((*S*)-*tert*-butyl-3-metylbutanoate)thiosemicarbazide in DMSO-d_6 at 499 MHz.

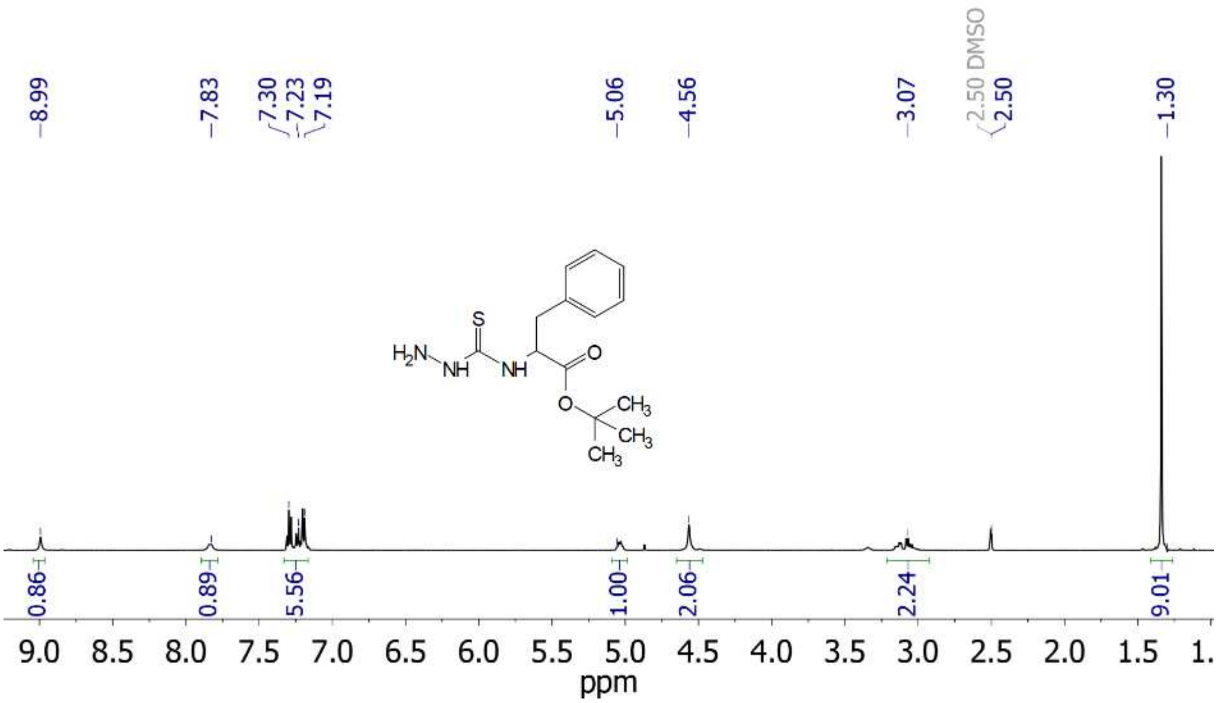

Figure S51. ^{1}H NMR spectrum of 4-((*S*)-*tert*-butyl-3-phenylpropanoate)thiosemicarbazide in DMSO-d_6 at 300 MHz.

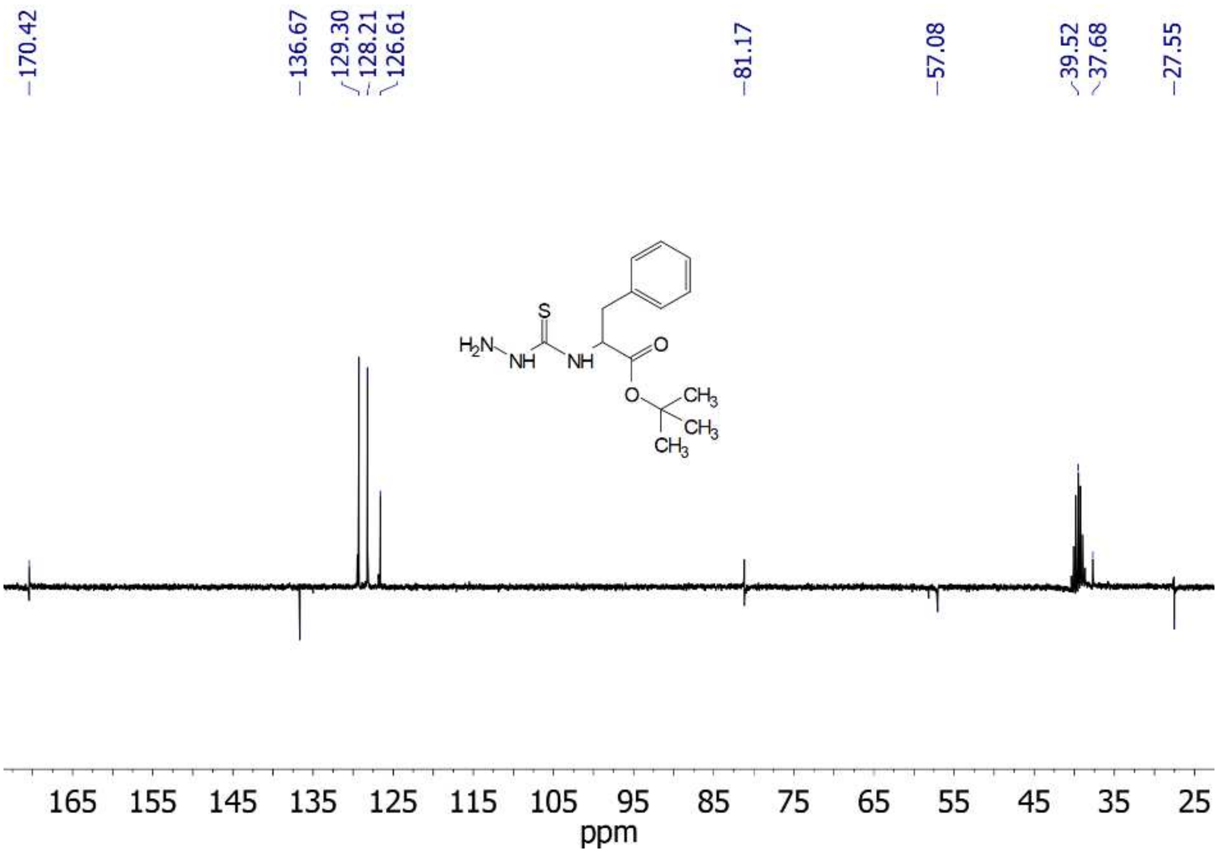

Figure S52. ^{13}C DEPTQ NMR spectrum of 4-((*S*)-*tert*-butyl-3-phenylpropanoate)thiosemicarbazide in DMSO-d_6 at 499 MHz.

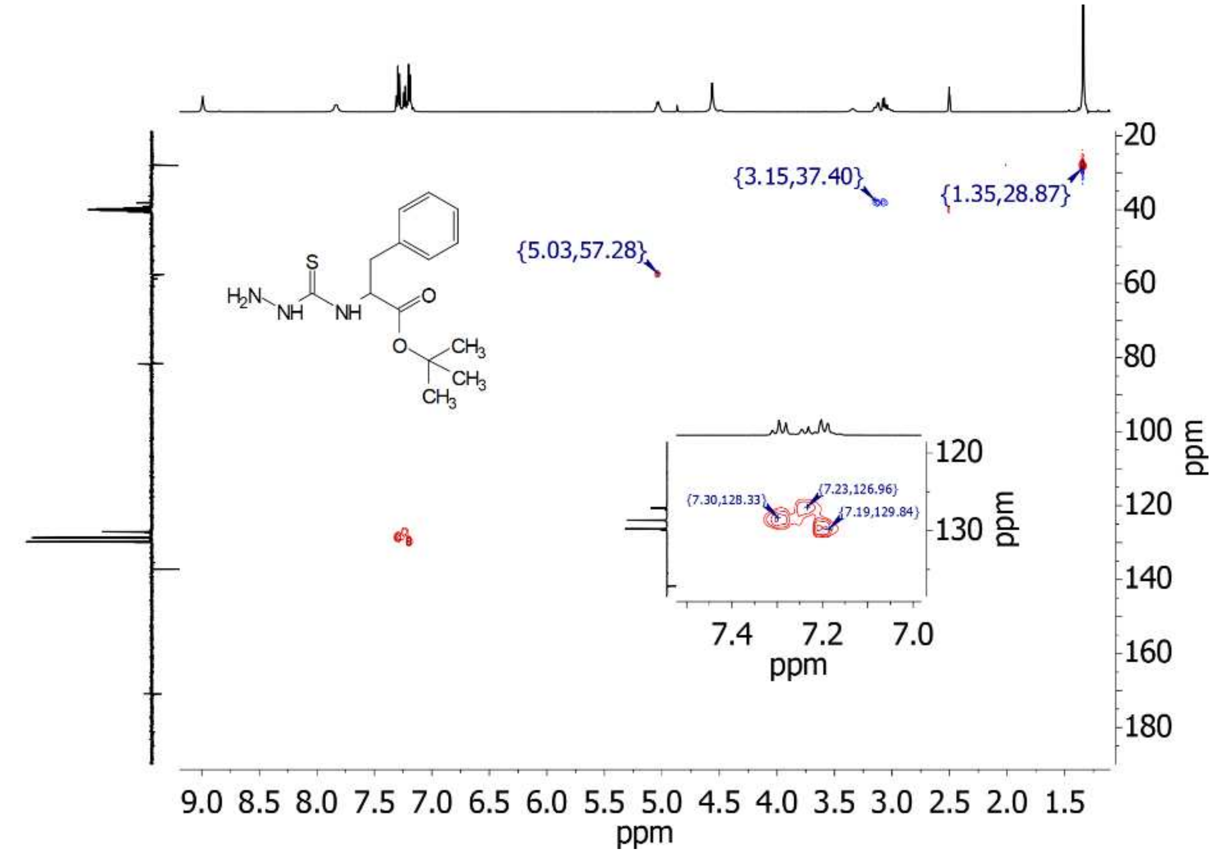

Figure S53. ^{1}H,^{13}C HMQC/HSQC NMR spectrum of 4-((*S*)-*tert*-butyl-3-phenylpropanoate)thiosemicarbazide in DMSO-d_6 at 499 MHz.

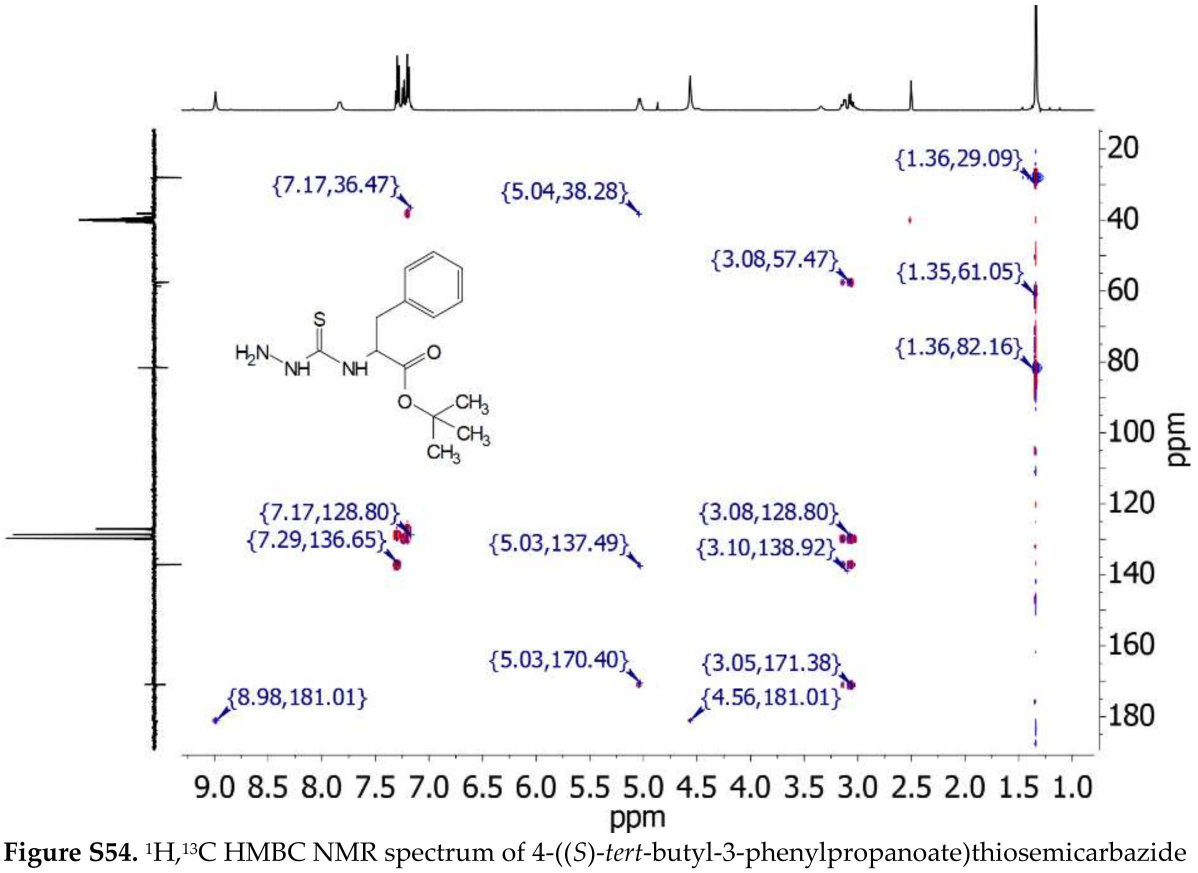

Figure S54. ^{1}H,^{13}C HMBC NMR spectrum of 4-((*S*)-*tert*-butyl-3-phenylpropanoate)thiosemicarbazide DMSO-d_6 at 499 MHz.

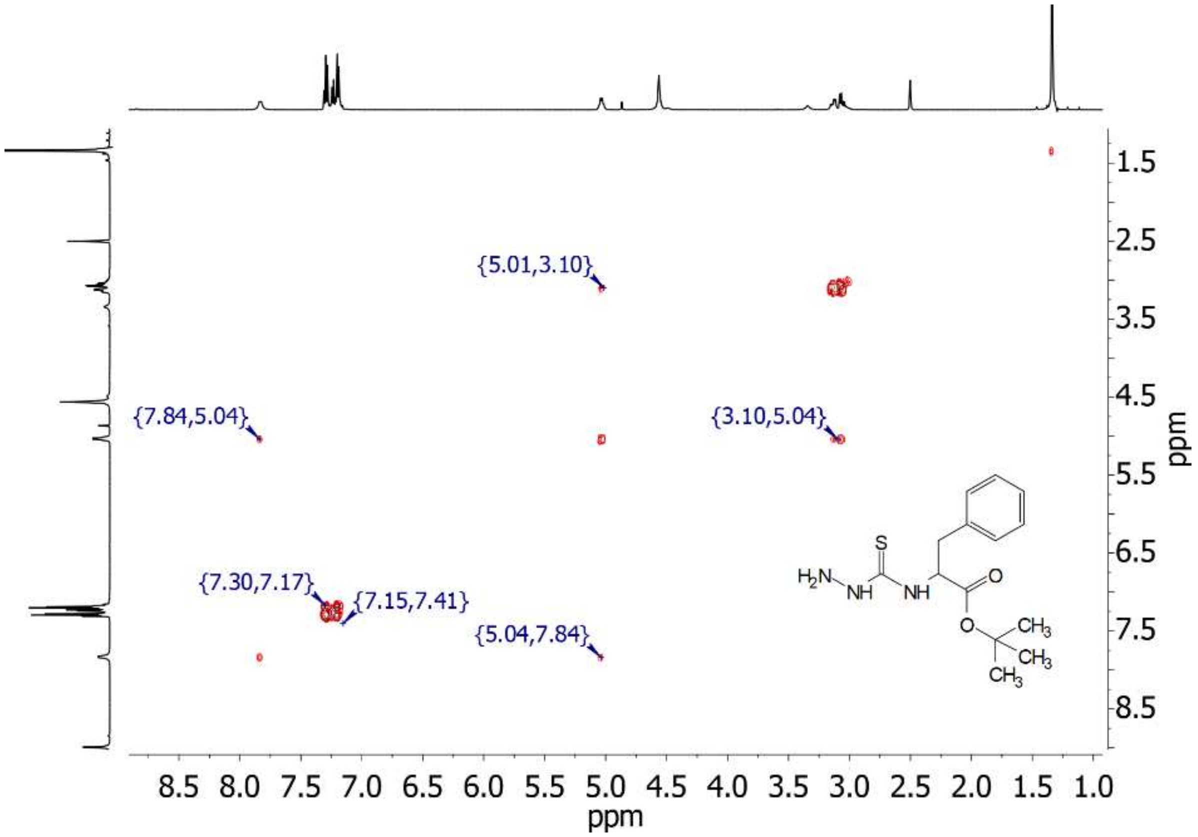

Figure S55. $^{1}H,^{1}H$ COSY-NMR spectrum of 4-((*S*)-*tert*-butyl-3-phenylpropanoate)thiosemicarbazide in DMSO-d_6 at 499 MHz.

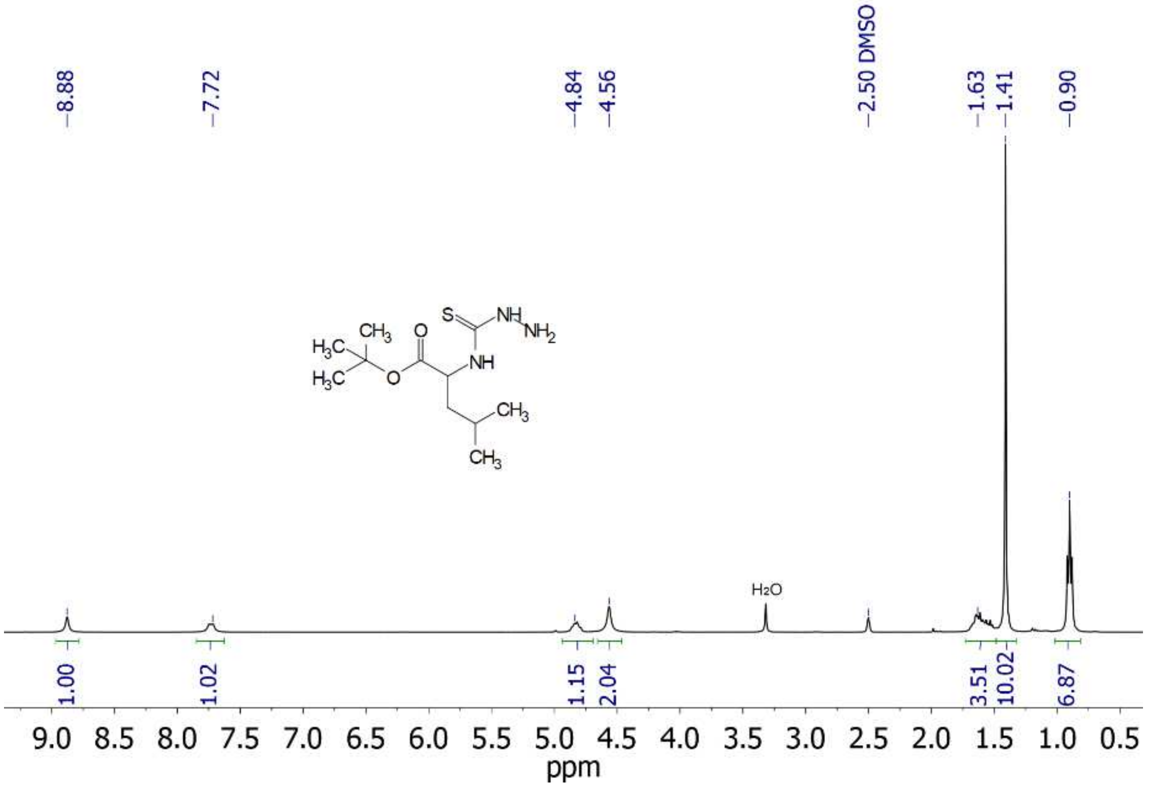

Figure S56. ^{1}H NMR spectrum of 4-((*S*)-*tert*-butyl-4-methylpentonate)thiosemicarbazide in DMSO-d_6 at 499 MHz.

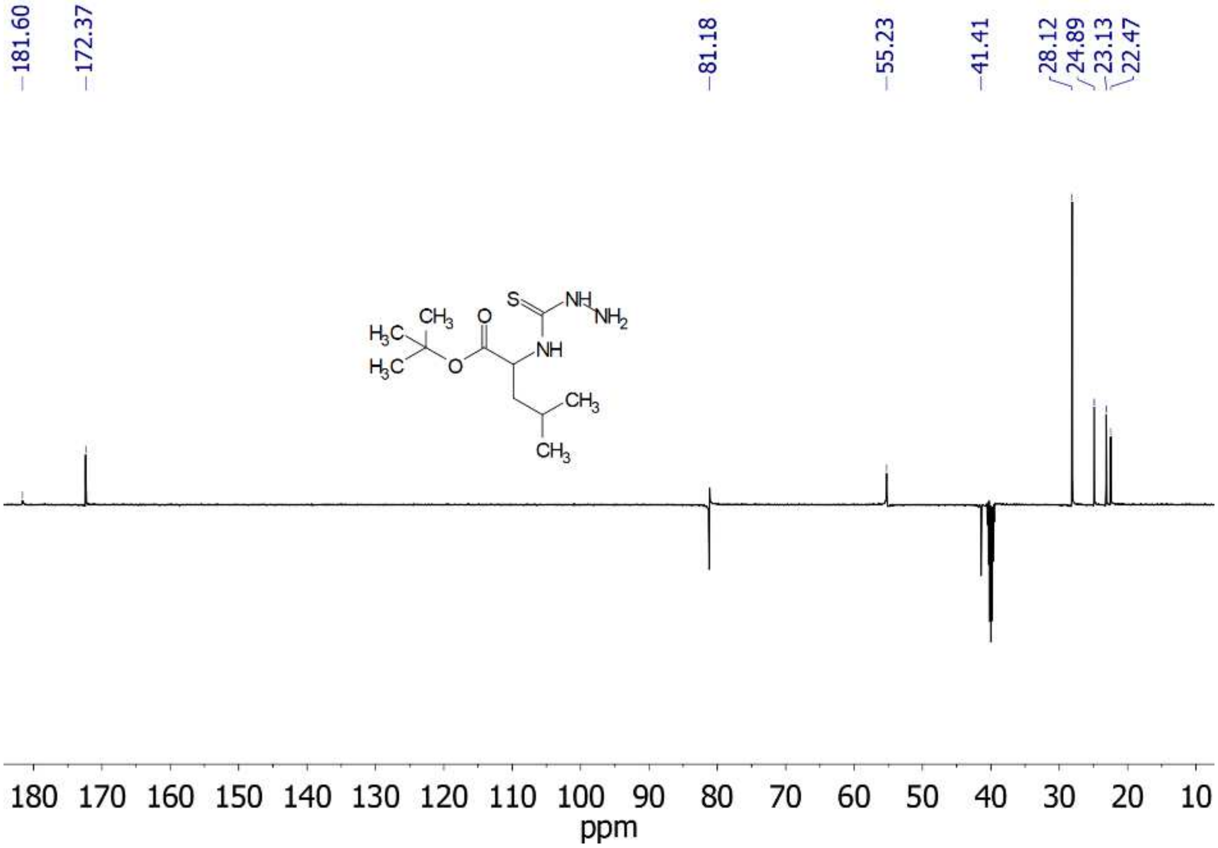

Figure S57. ^{13}C APT NMR spectrum of 4-((*S*)-*tert*-butyl-4-methylpentonate)thiosemicarbazide in DMSO-d_6 at 499 MHz.

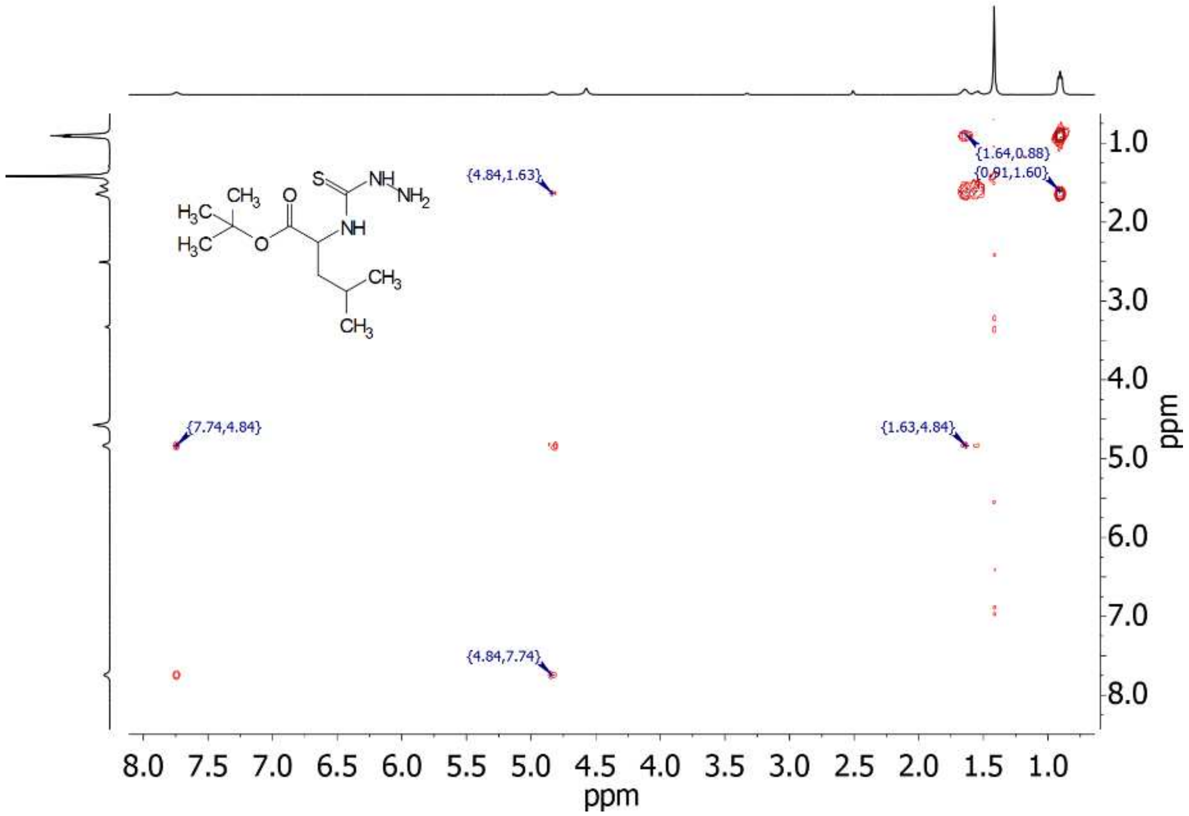

Figure S58. $^{1}H,^{1}H$ COSY NMR spectrum of 4-((*S*)-*tert*-butyl-4-methylpentonate)thiosemicarbazide in DMSO-d_6 at 499 MHz.

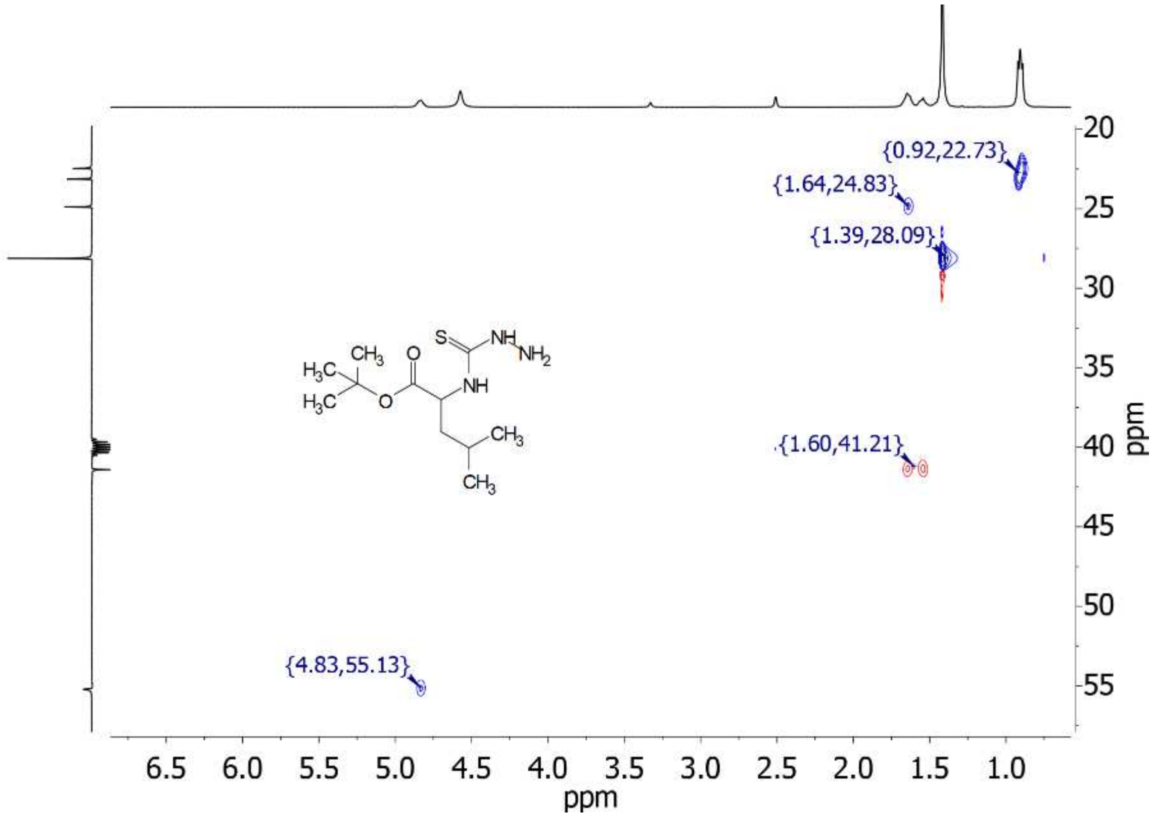

Figure S59. $^{1}H,^{13}C$ HMQC/HSQC NMR spectrum of 4-((*S*)-*tert*-butyl-4-methylpentonate)thiosemicarbazide in DMSO-d_6 at 499 MHz.

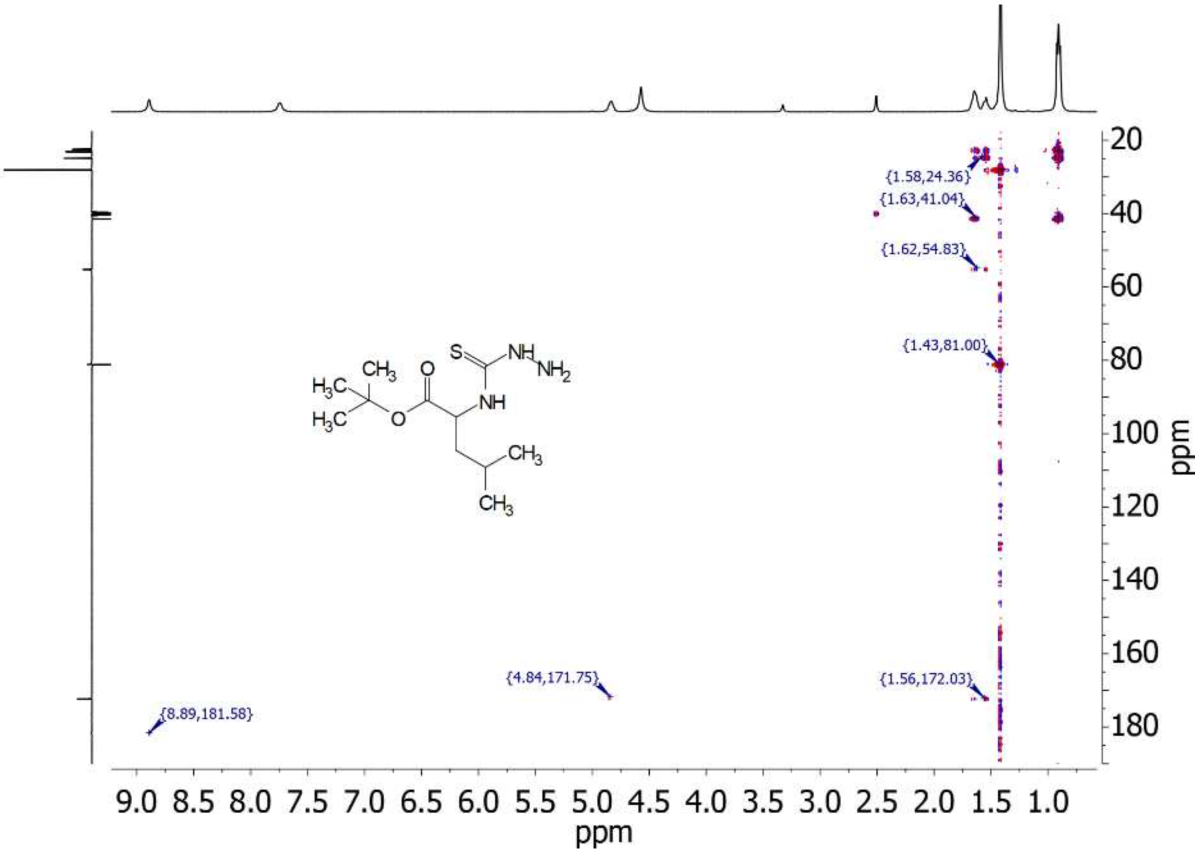

Figure S60. $^{1}H,^{13}C$ HMBC NMR spectrum of 4-((*S*)-*tert*-butyl-4-methylpentonate)thiosemicarbazide in DMSO-d_6 at 499 MHz.

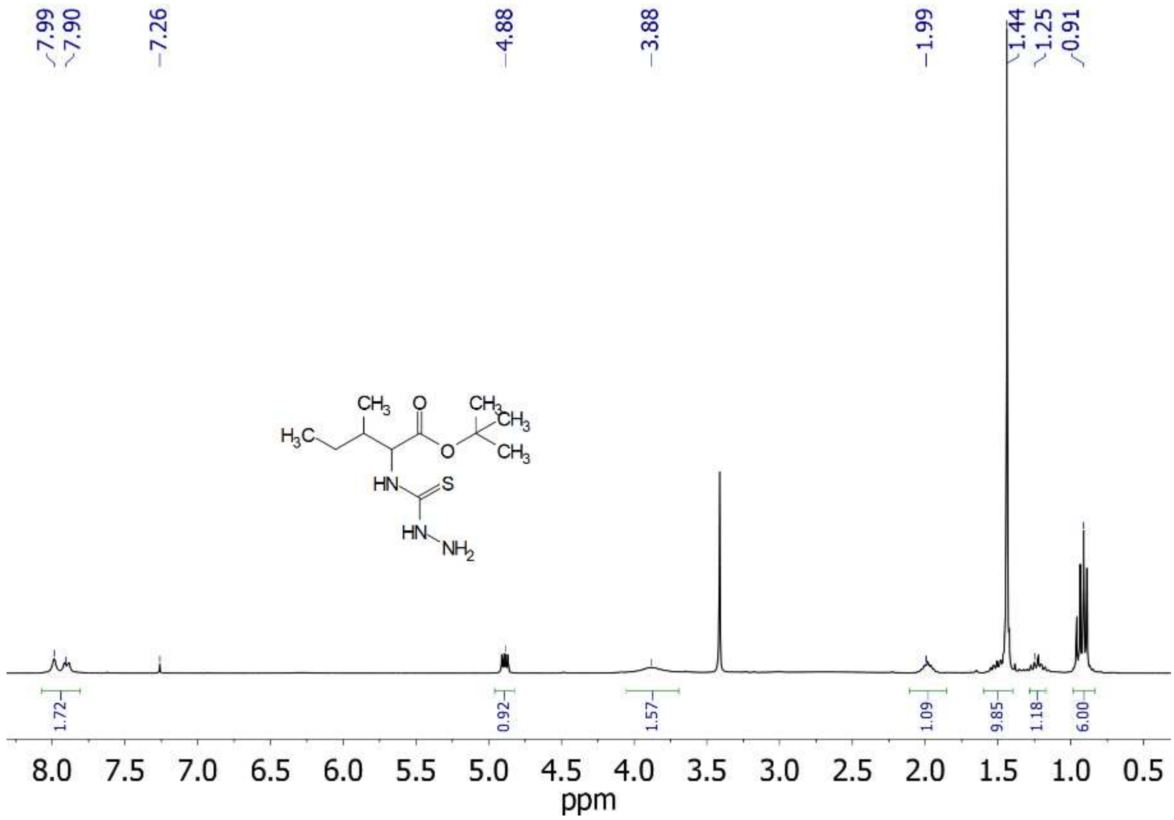

Figure S61. ^{1}H NMR spectrum of (*S*)-*tert*-butyl 2-(hydrazidecarbothioamino)-3-methylpentanoate in DMSO-d_6 at 300 MHz.

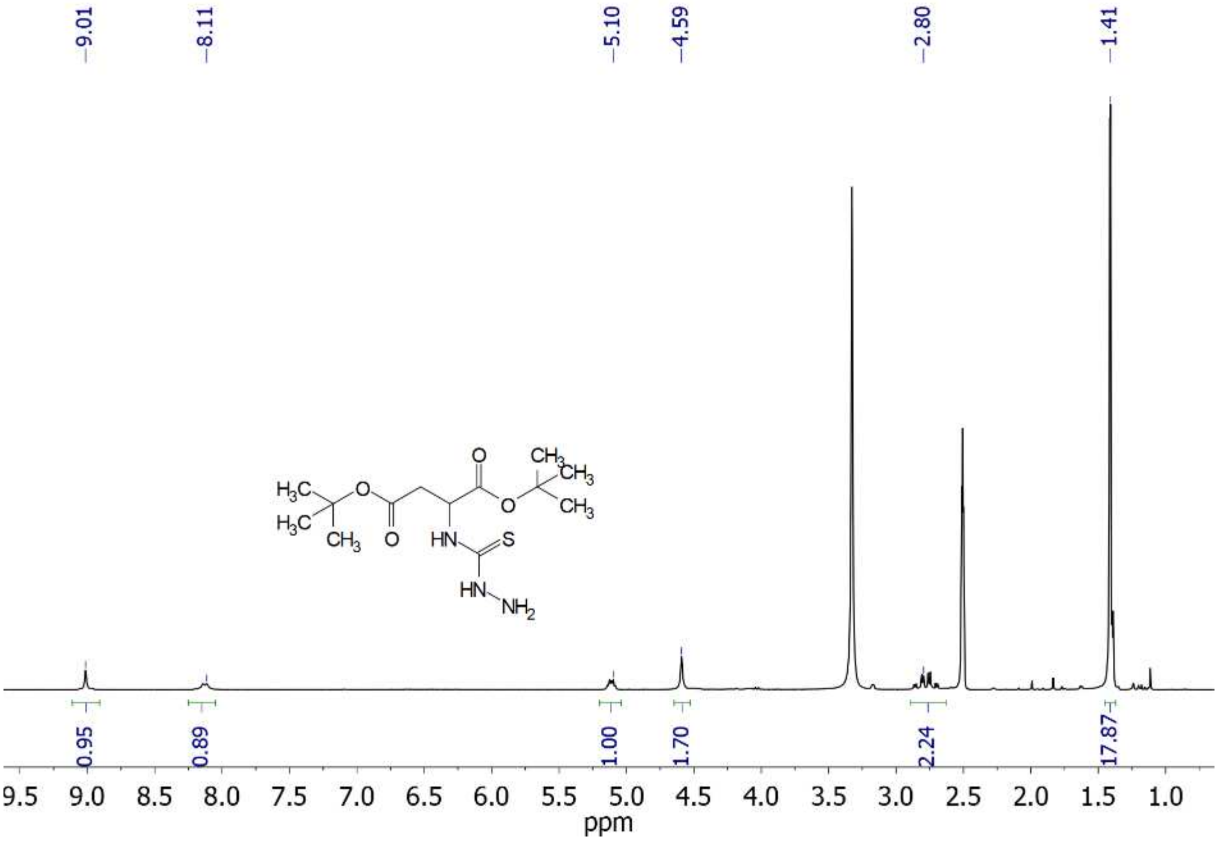

Figure S62. ^{1}H NMR spectrum of (*S*)-Di-*tert*-butyl 2-(hydrazidecarbothioamino)butanedioate in DMSO-d_6 at 300 MHz.

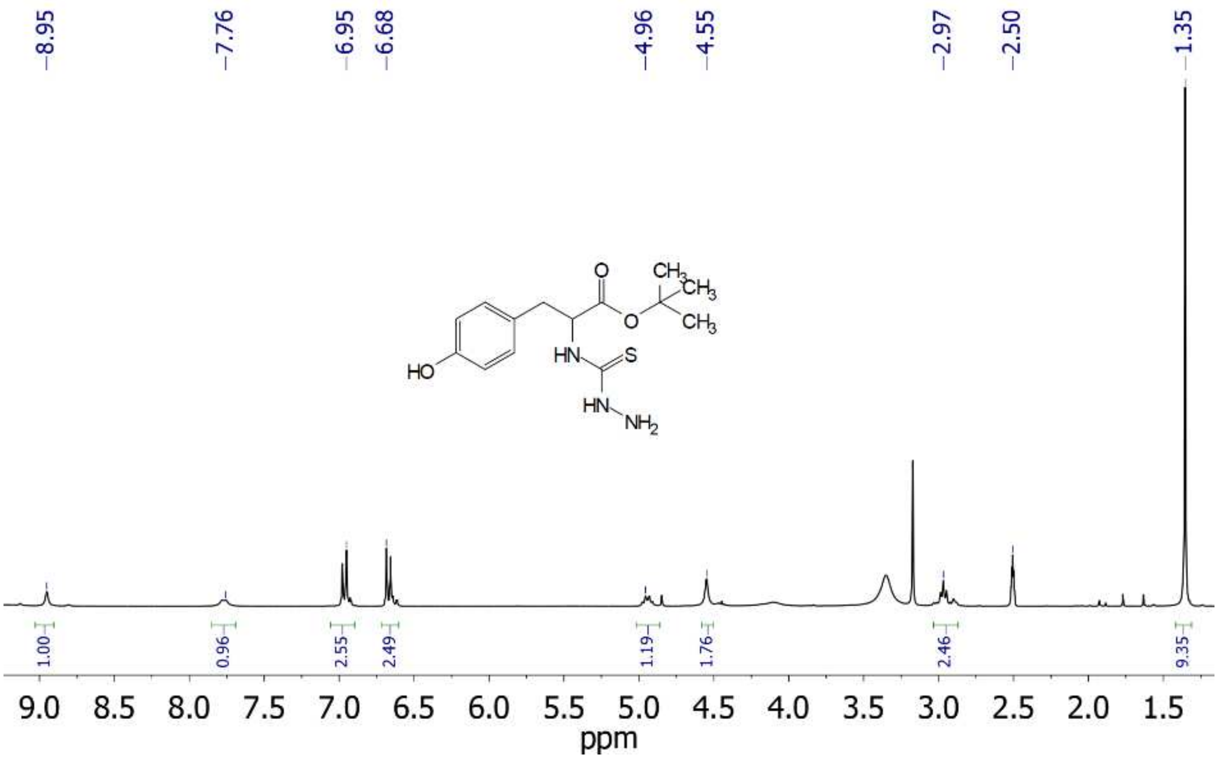

Figure S63. ^{1}H NMR spectrum of (*S*)-*tert*-butyl-2-(hydrazidecarbothioamino)-3-(4-hydroxyphenyl) propanoate in DMSO-d_6 at 300 MHz.

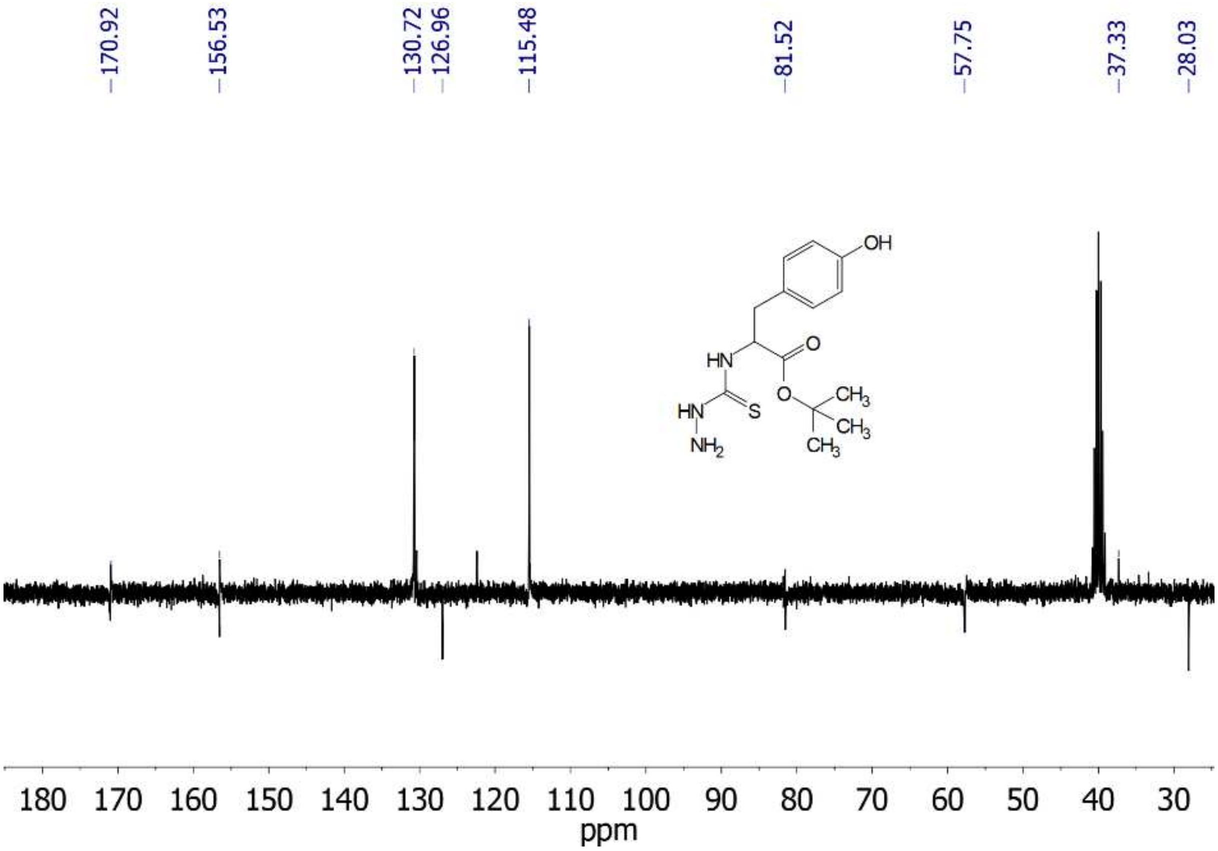

Figure S64. ^{13}C DEPTQ NMR spectrum of (*S*)-*tert*-butyl-2-(hydrazidecarbothioamino)-3-(4-hydroxyphenyl)propanoate in DMSO-d_6 at 300 MHz.

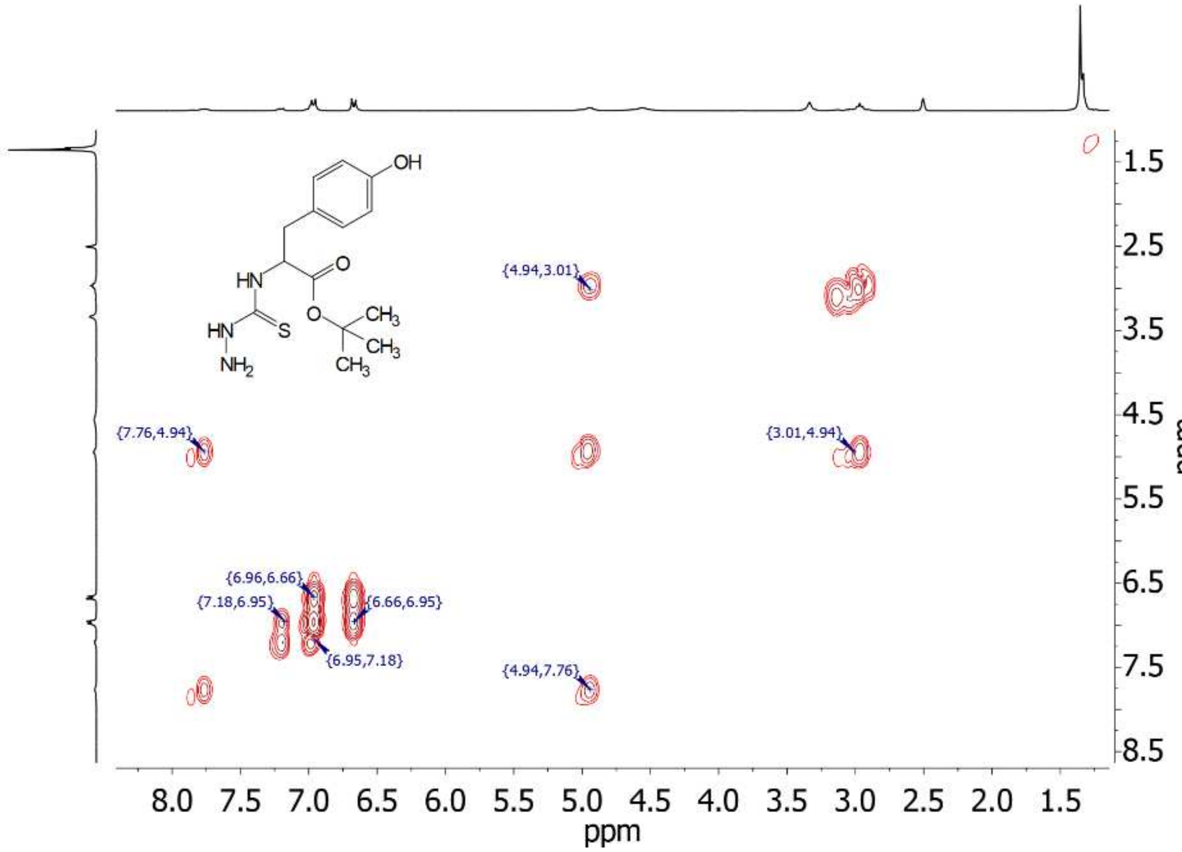

Figure S65. $^{1}H,^{1}H$ COSY NMR spectrum of (*S*)-*tert*-butyl-2-(hydrazidecarbothioamino)-3-(4-hydroxyphenyl)propanoate in DMSO-d_6 at 300 MHz.

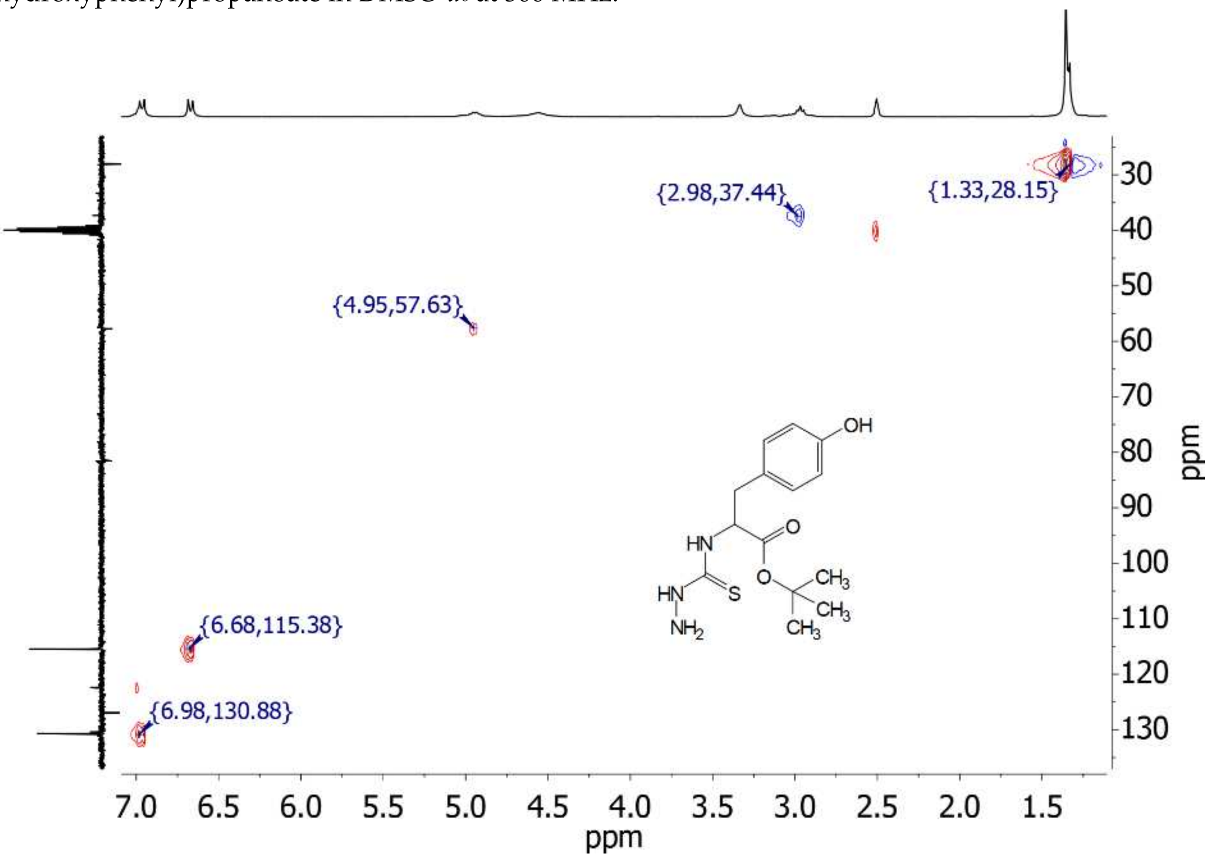

Figure S66. $^{1}H,^{13}C$ HMQC/HSQC NMR spectrum of (*S*)-*tert*-butyl-2-(hydrazidecarbothioamino)-3-(4-hydroxyphenyl)propanoate in DMSO-d_6 at 300 MHz.

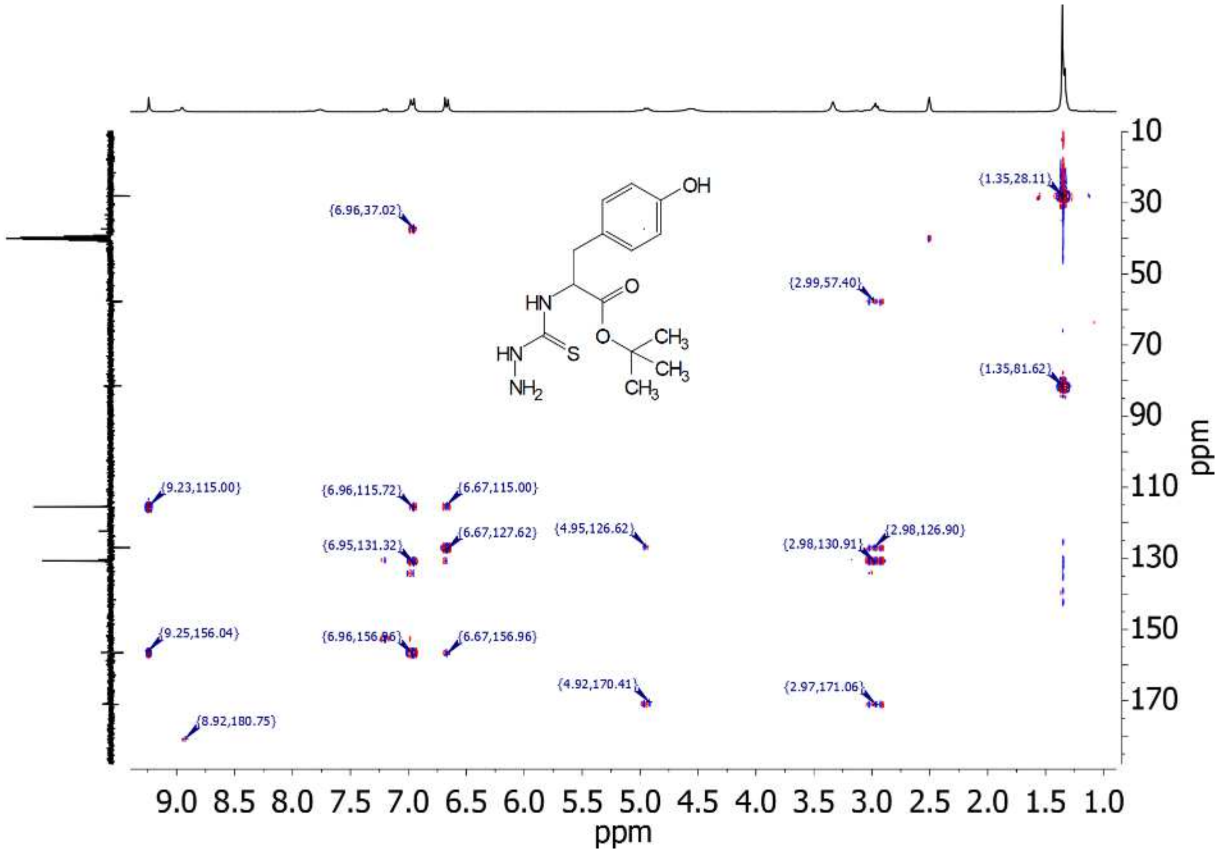

Figure S67. $^{1}H,^{13}C$ HMBC NMR spectrum of (*S*)-*tert*-butyl-2-(hydrazidecarbothioamino)-3-(4-hydroxyphenyl)propanoate in DMSO-d_6 at 300 MHz.

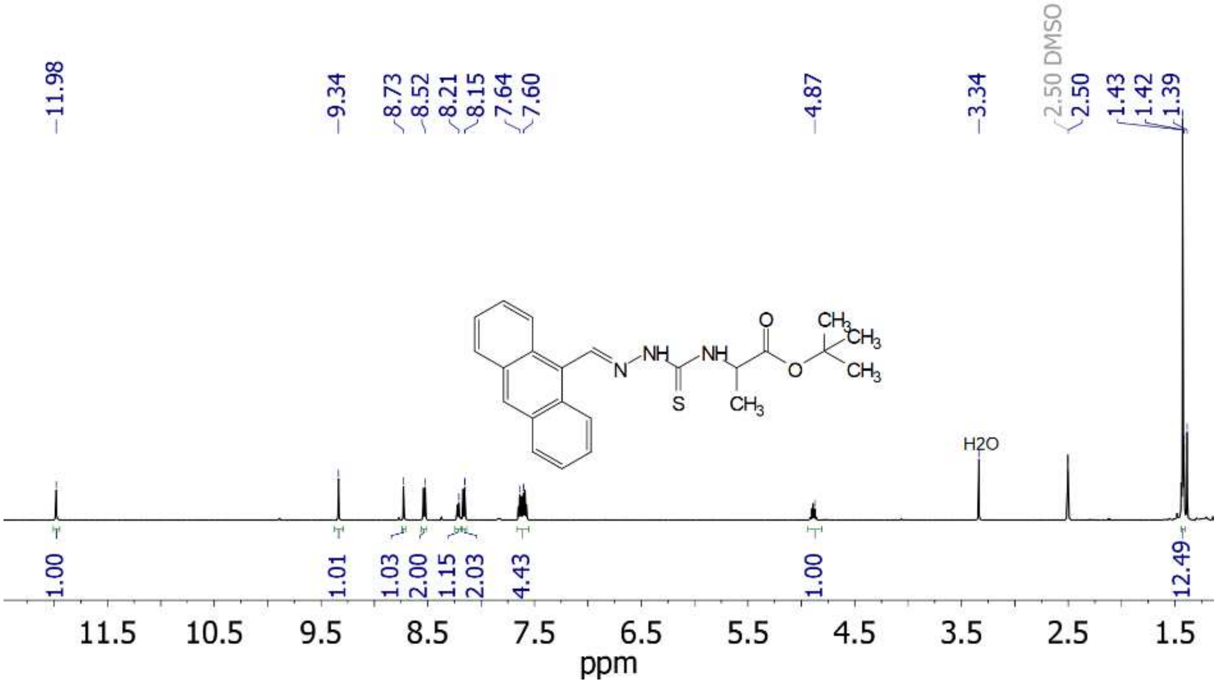

Figure S68. ^{1}H NMR spectrum of 9-anthraldehyde-4-((*S*)-*tert*-butylpropanoate)-3-thiosemicarbazone DMSO-d_6 at 499 MHz.

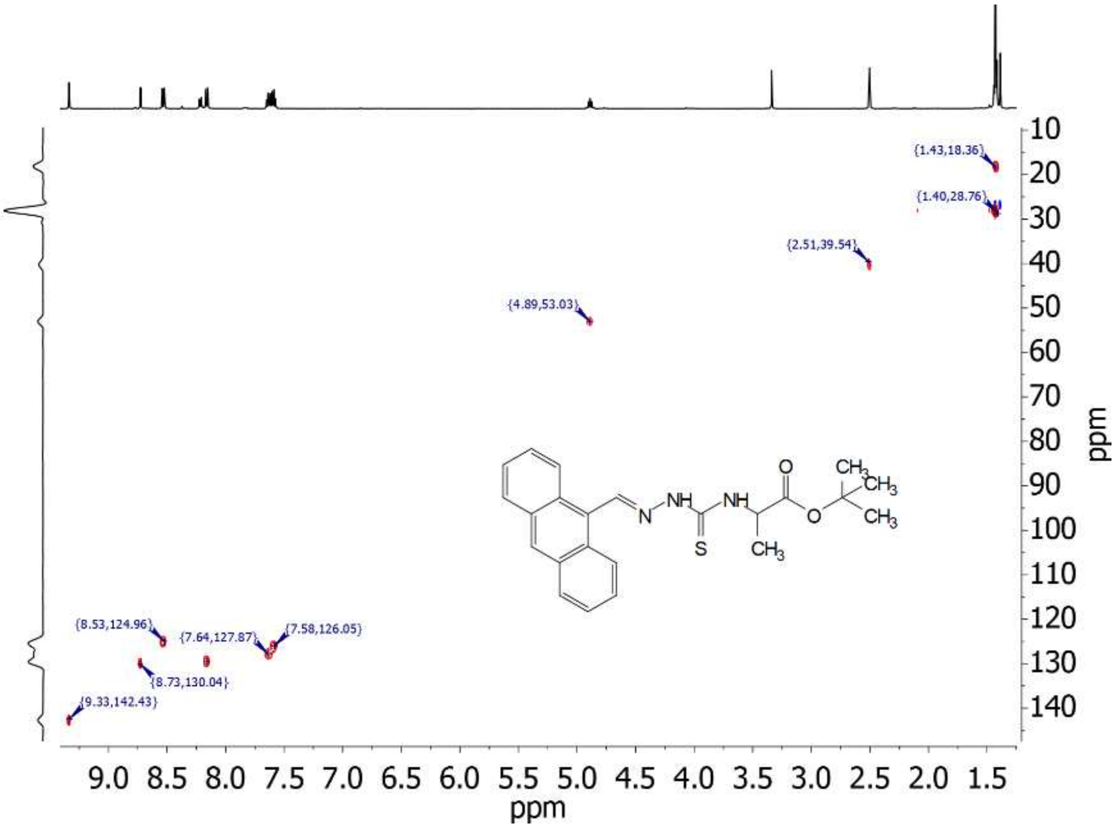

Figure S69. ^{1}H,^{13}C HMQC/HSQC NMR of 9-anthraldehyde-4-((*S*)-*tert*-butylpropanoate)-3-thiosemicarbazone spectrum in DMSO-d_6 at 499 MHz.

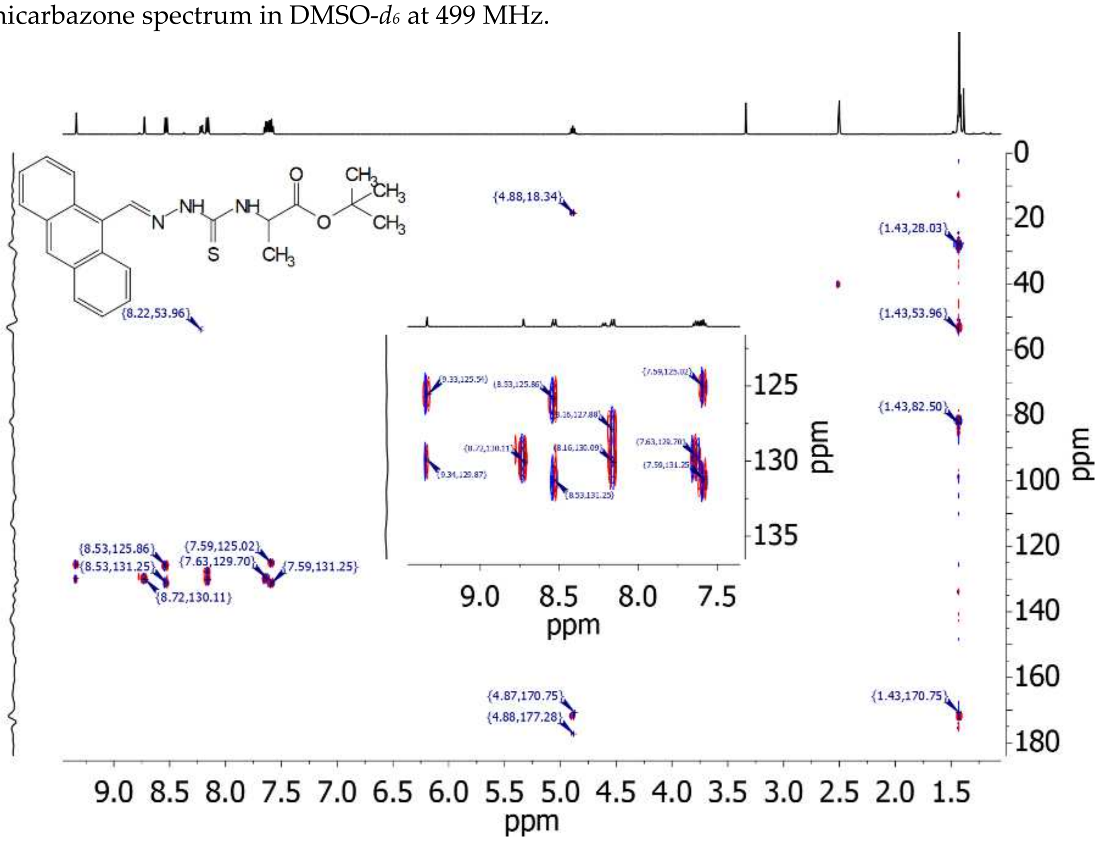

Figure S70. ^{1}H,^{13}C HMBC NMR spectrum of 9-anthraldehyde-4-((*S*)-*tert*-butylpropanoate)-3-thiosemicarbazone DMSO-d_6 at 499 MHz.

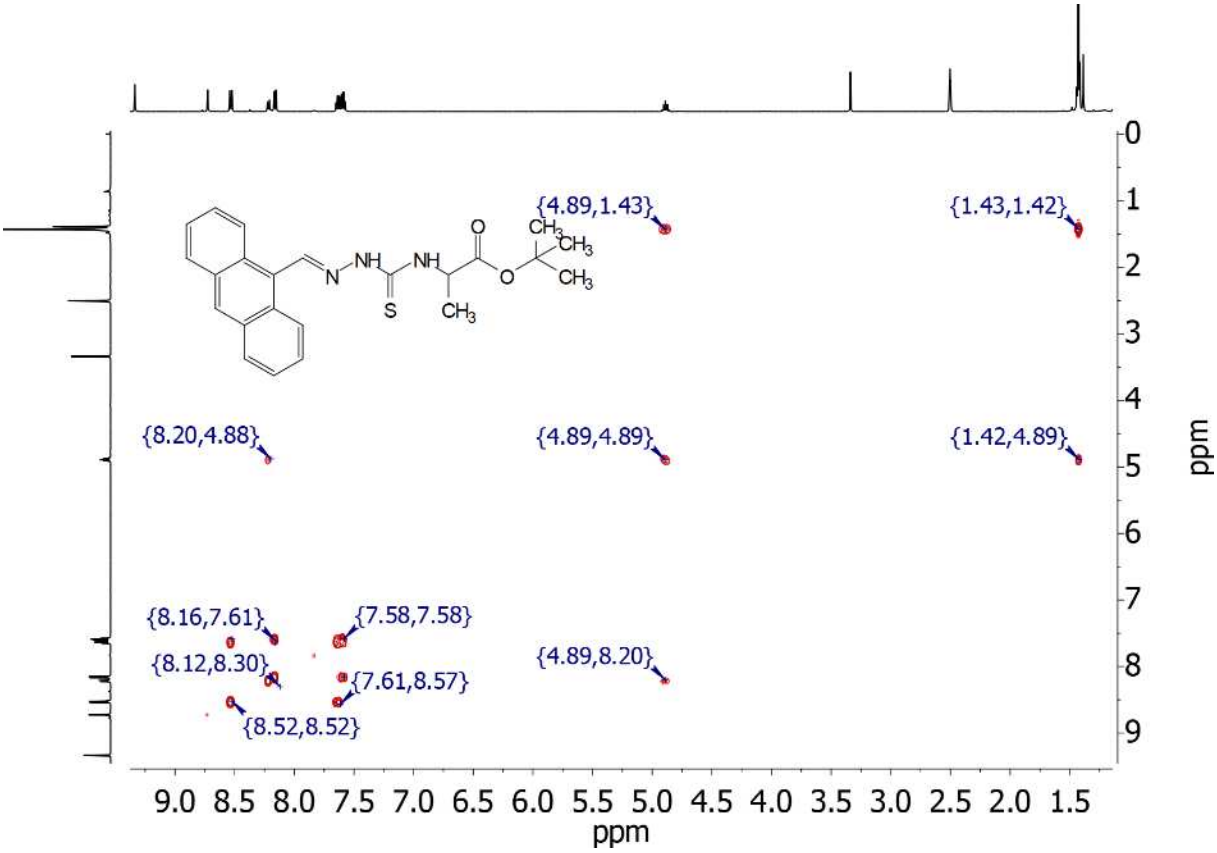

Figure S71. $^{1}H,^{1}H$ COSY NMR spectrum of 9-anthraldehyde-4-((*S*)-*tert*-butylpropanoate)-3-thiosemicarbazone in DMSO-d_6 at 499 MHz.

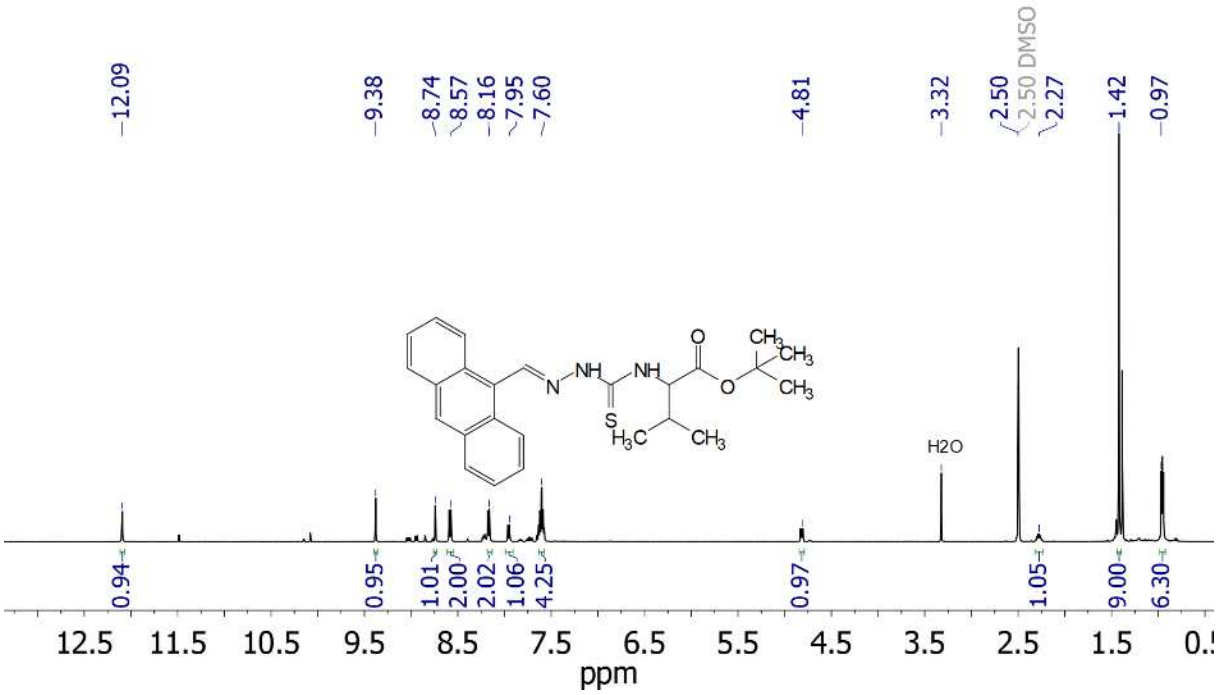

Figure S72. ^{1}H NMR spectrum of 9-anthraldehyde-4-((*S*)-*tert*-butyl 3-metylbutanoate)-3-thiosemicarbazone in DMSO-d_6 at 499 MHz.

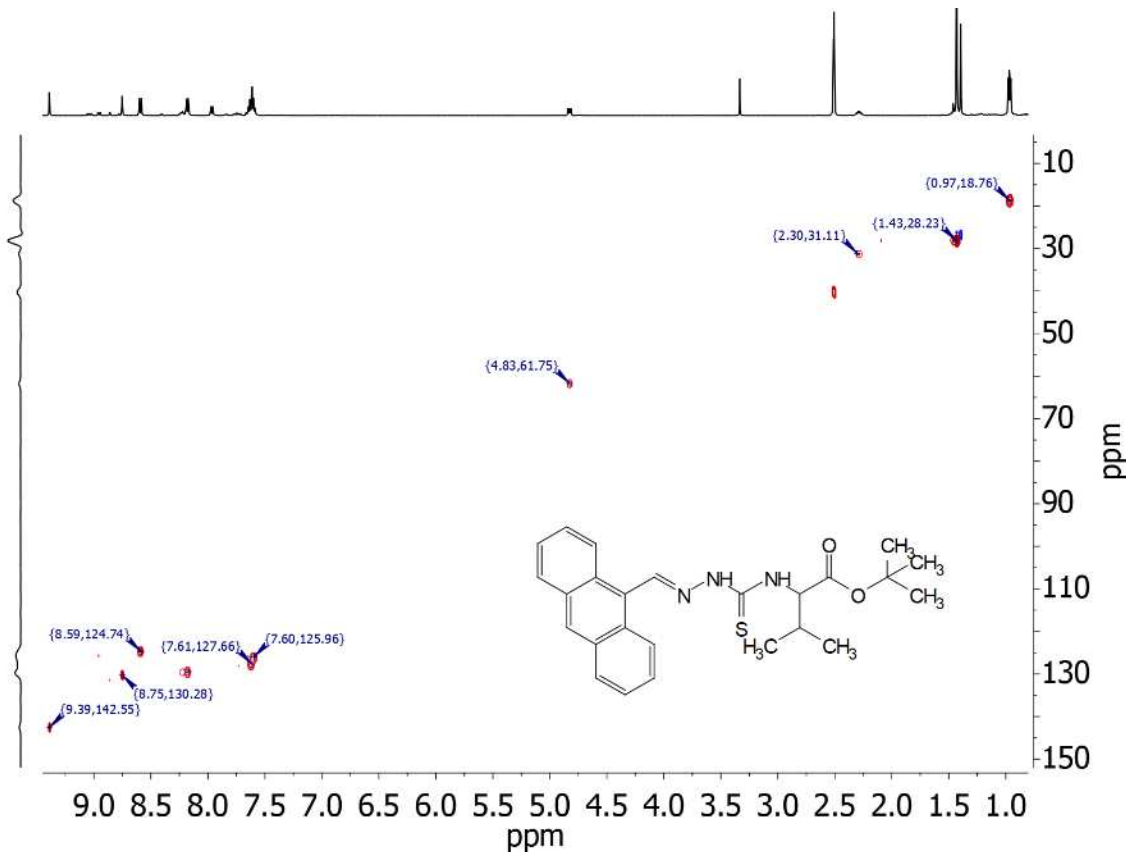

Figure S73. $^{1}H,^{13}C$ HMQC/HSQC NMR spectrum of 9-anthraldehyde-4-((*S*)-*tert*-butyl-3-metyl-butanoate)-3-thiosemicarbazone in DMSO-d_6 at 499 MHz.

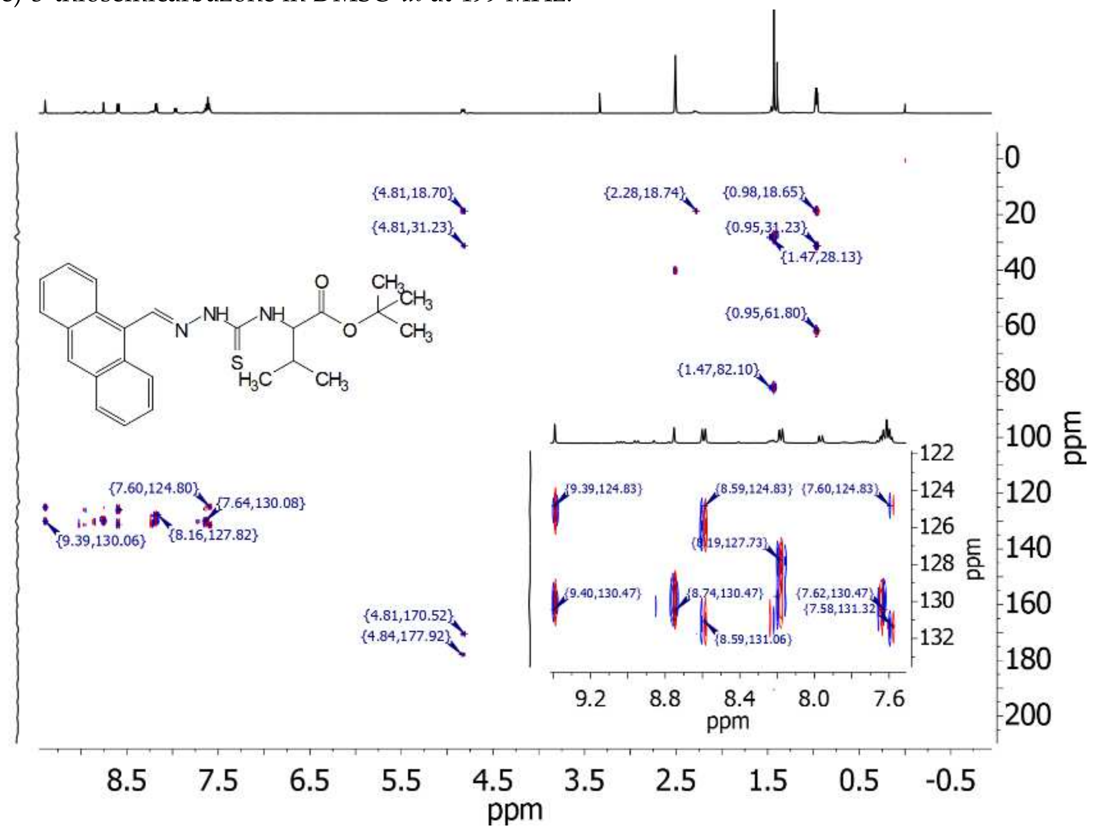

Figure S74. $^{1}H,^{13}C$ HMBC NMR spectrum of 9-anthraldehyde-4-((*S*)-*tert*-butyl 3-metylbutanoate)-3-thiosemicarbazone in DMSO-d_6 at 499 MHz.

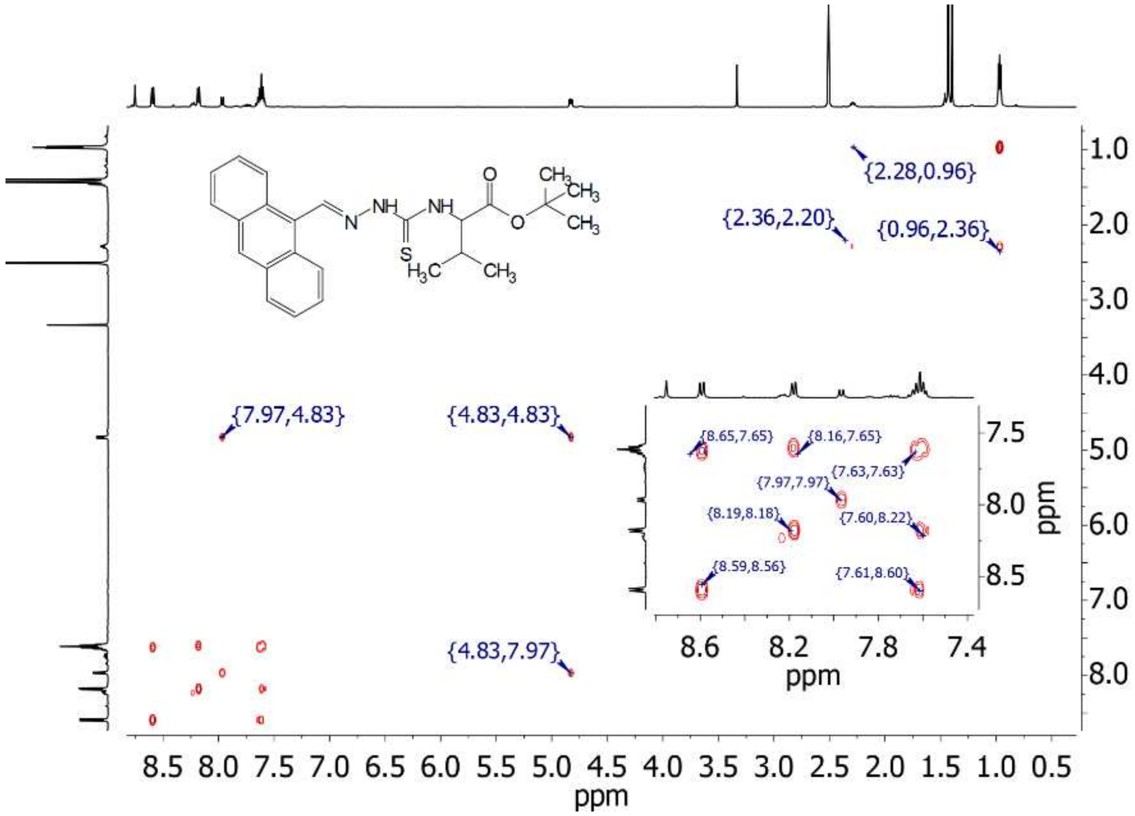

Figure S75. $^{1}H,^{1}H$ COSY NMR spectrum of 9-anthraldehyde-4-((*S*)-*tert*-butyl 3-metylbutanoate)-3-thiosemicarbazone in DMSO-d_6 at 499 MHz.

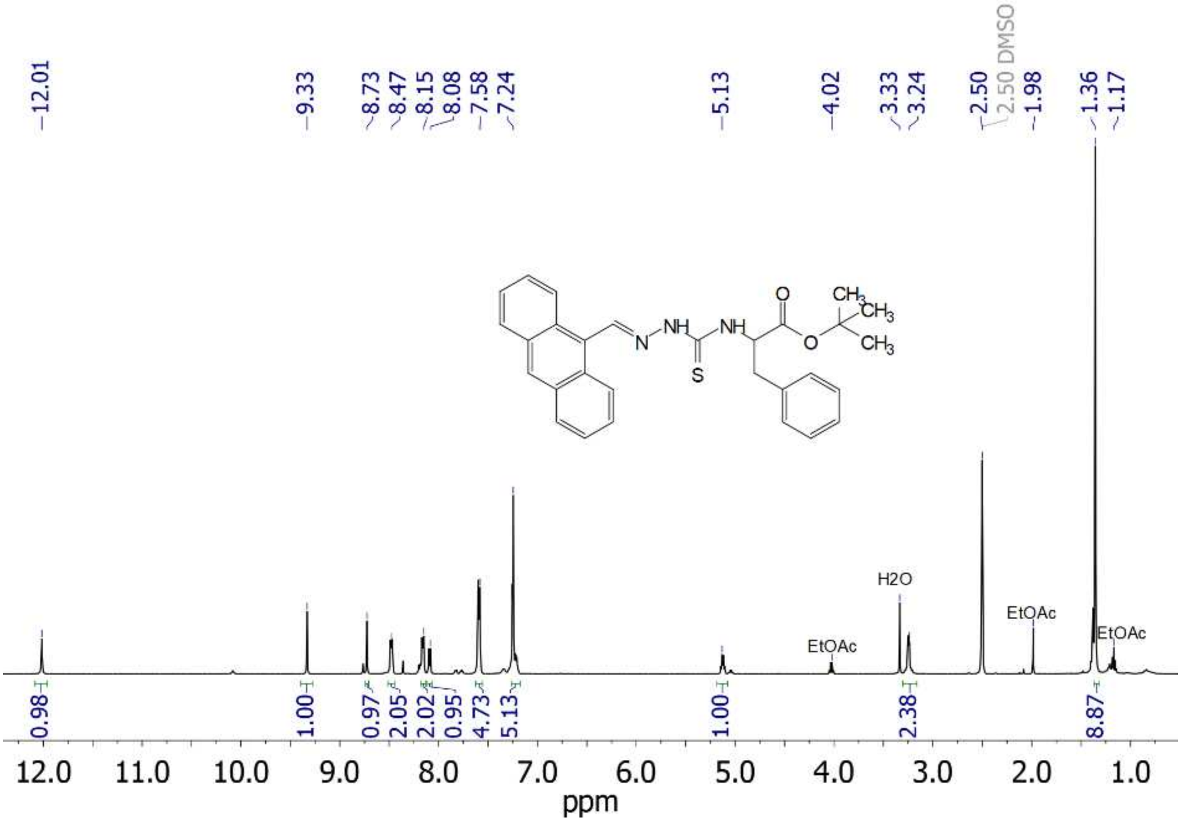

Figure S76. ^{1}H NMR spectrum of 9-anthraldehyde-4-((*S*)-*tert*-butyl 3-metylbutanoate)-3-thiosemicarbazone in DMSO-d_6 at 499 MHz.

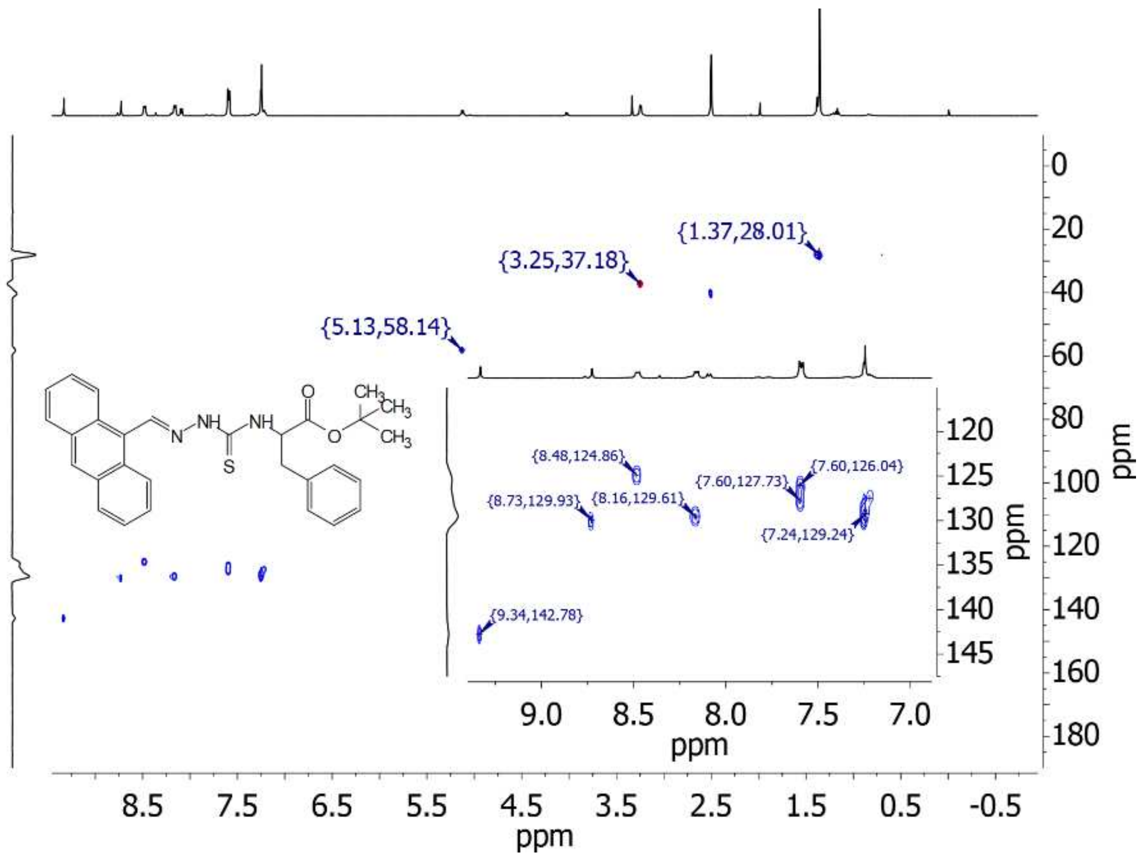

Figure S77. ^{1}H,^{13}C HMQC/HSQC NMR spectrum of 9-anthraldehyde-4-((*S*)-*tert*-butyl 3-metylbutanoate)-3-thiosemicarbazone in DMSO-d_6 at 499 MHz.

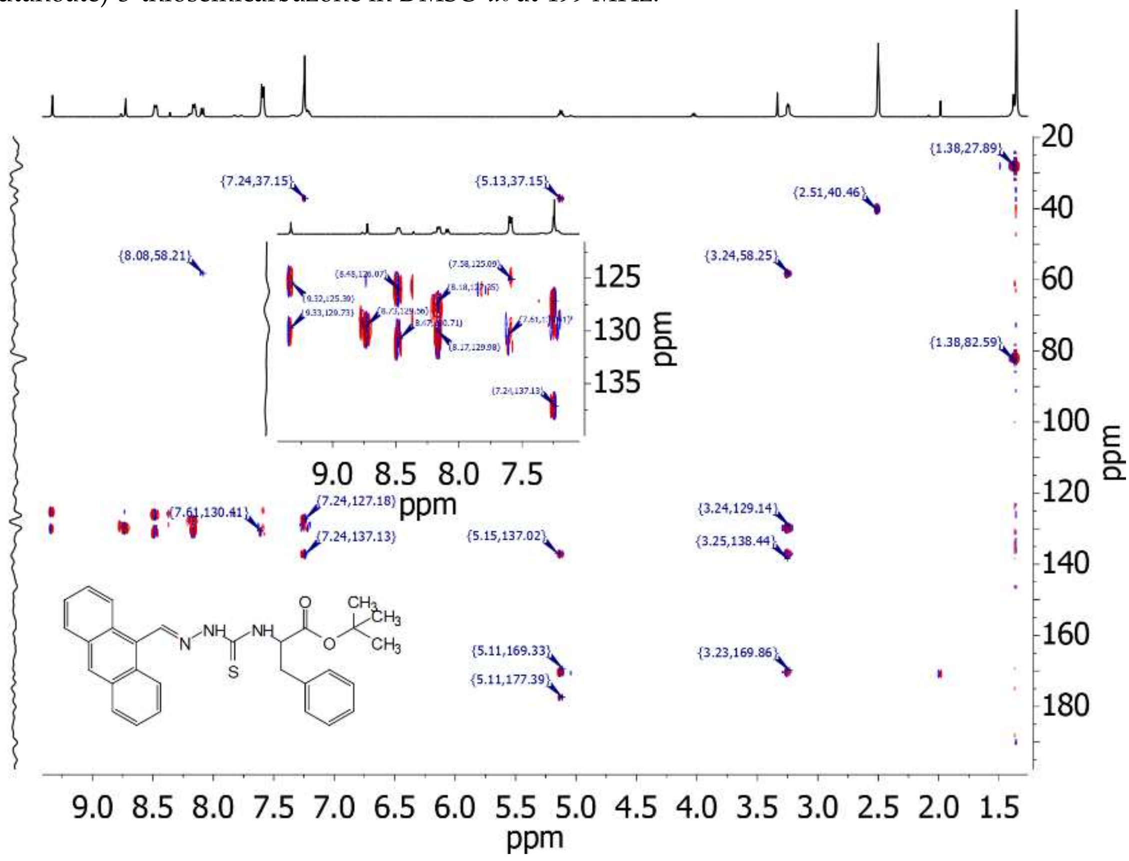

Figure S78. ^{1}H,^{13}C HMBC NMR spectrum of 9-anthraldehyde-4-((*S*)-*tert*-butyl 3-metylbutanoate)-3-thiosemicarbazone in DMSO-d_6 at 499 MHz.

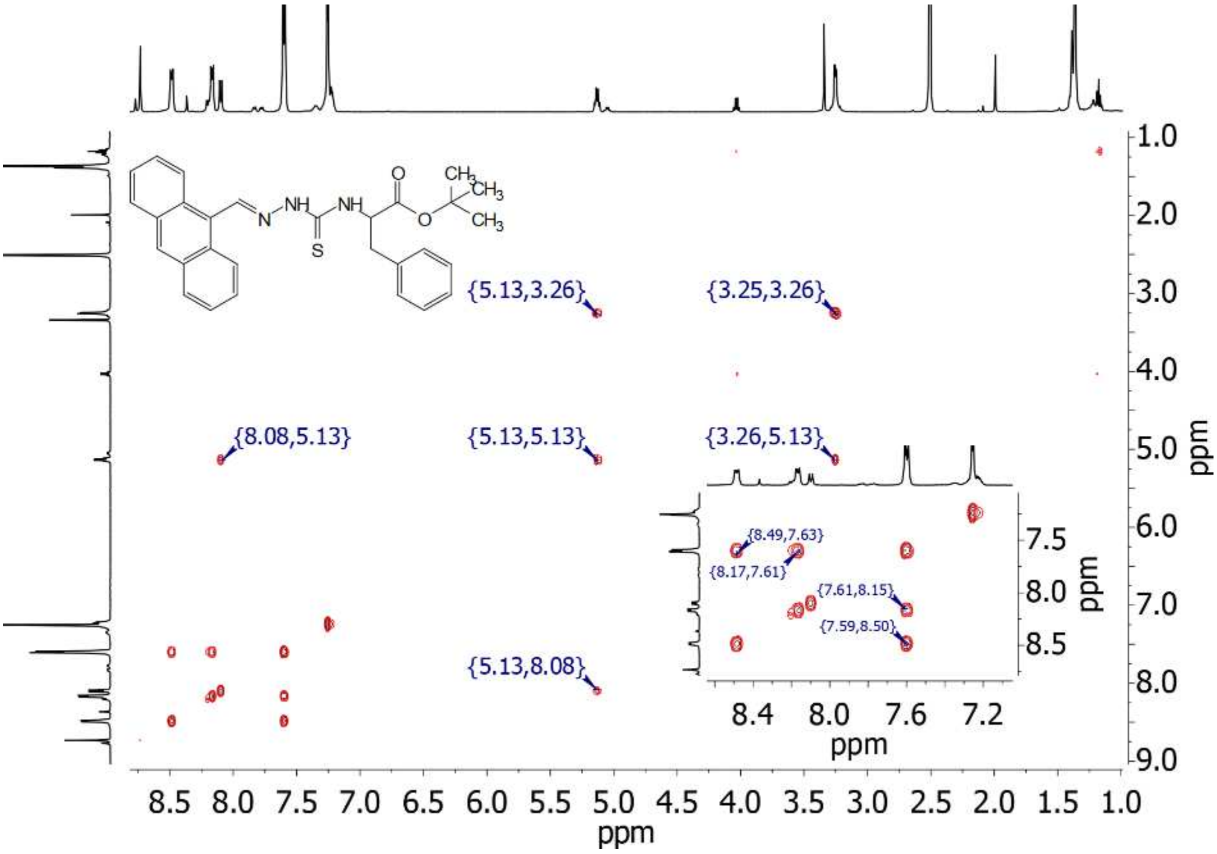

Figure S79. $^{1}H,^{1}H$ COSY NMR spectrum of 9-anthraldehyde-4-((*S*)-*tert*-butyl 3-metylbutanoate)-3-thiosemicarbazone DMSO-*d*₆ at 499 MHz.

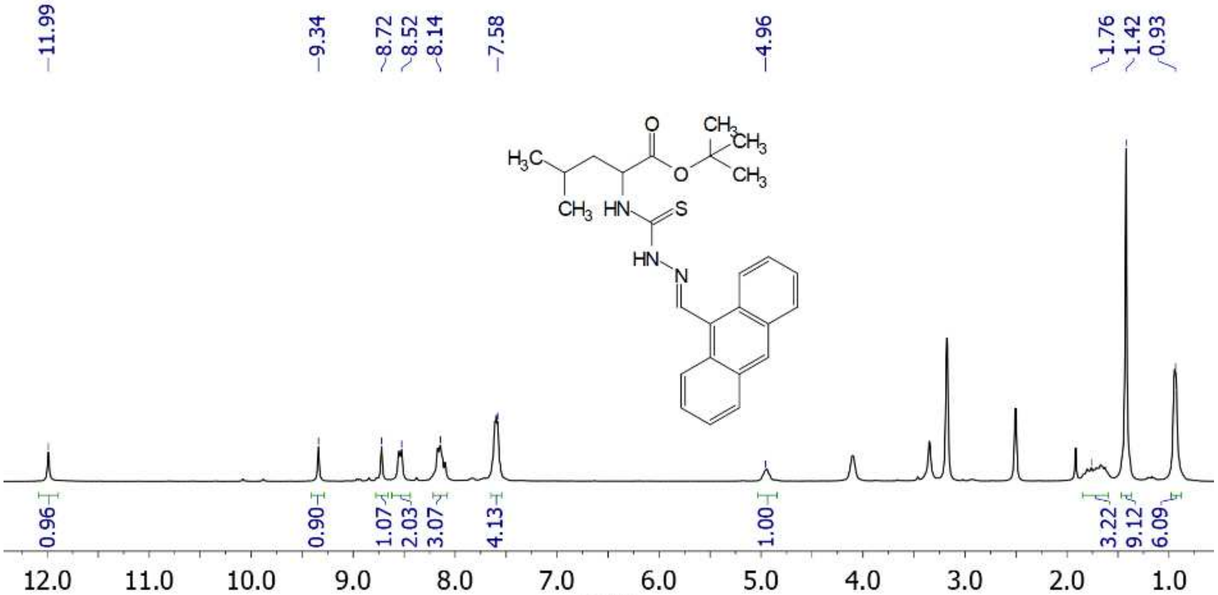

Figure S80. ^{1}H NMR spectrum of 9-anthraldehyde-4-((*S*)-*tert*-butyl 4-methylpentonate)-3-thiosemicarbazone in DMSO-d_6 at 300 MHz.

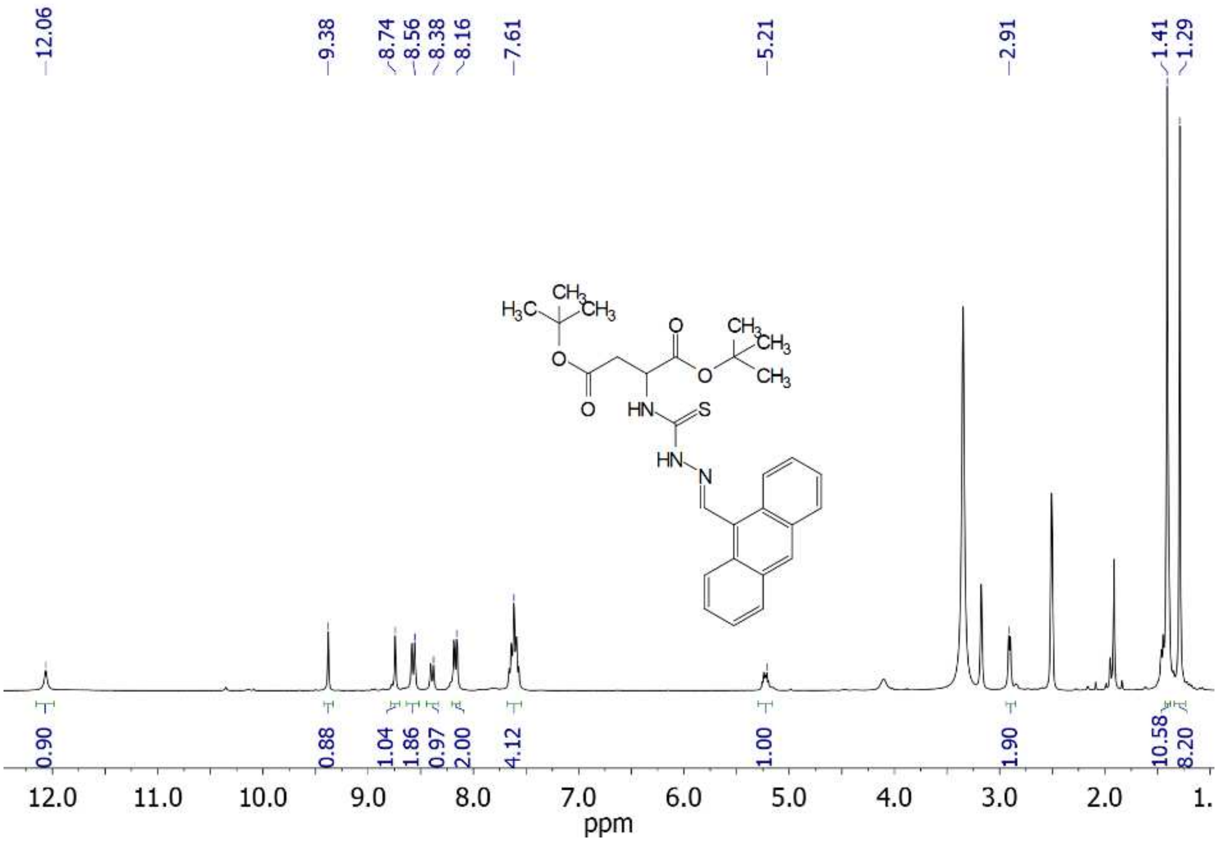

Figure S81. ^{1}H NMR spectrum of di-*tert*-butyl 2-{[(1*E*)-(anthracen-9-yl)methylideneamino-carbamthioyl]amino} butanedioate in DMSO-d_6 at 300 MHz.

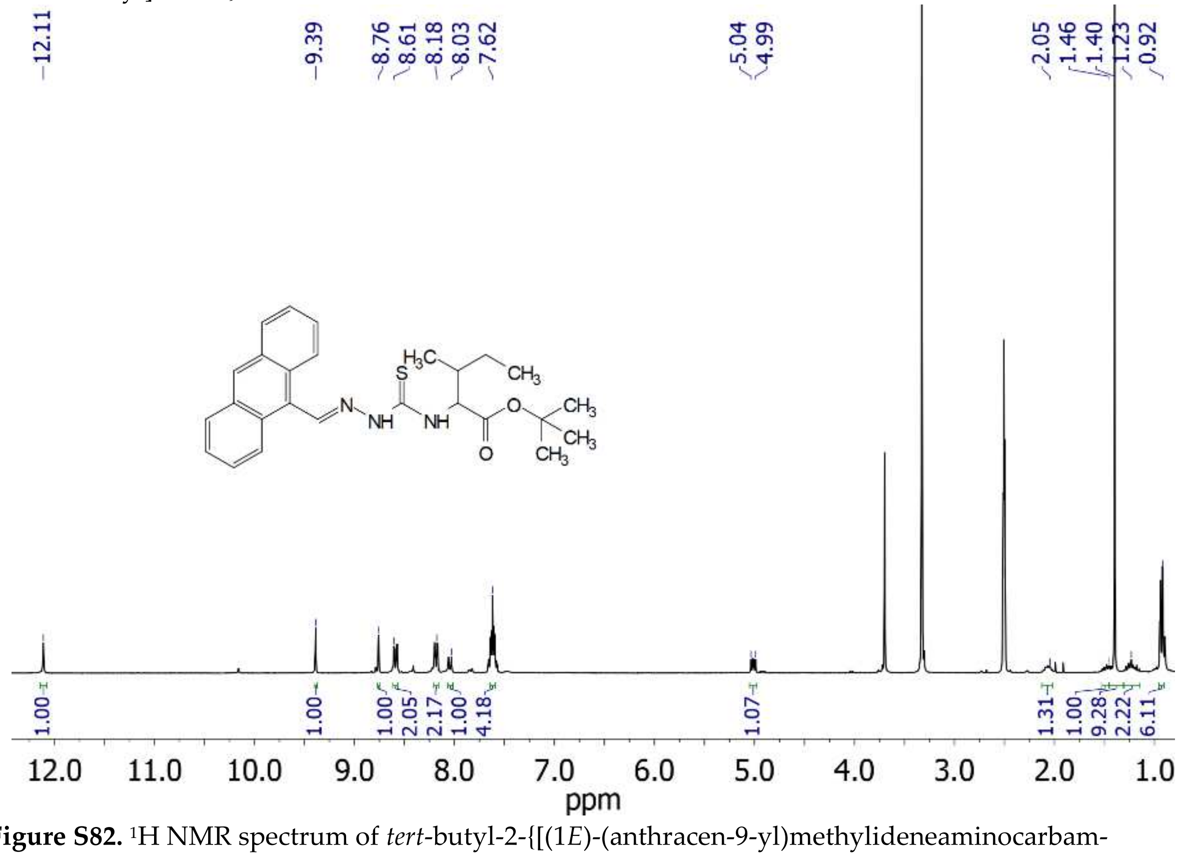

Figure S82. ^{1}H NMR spectrum of *tert*-butyl-2-{[(1*E*)-(anthracen-9-yl)methylideneaminocarbam-thioyl]amino}-3-methylpentanoate in DMSO-d_6 at 300 MHz.

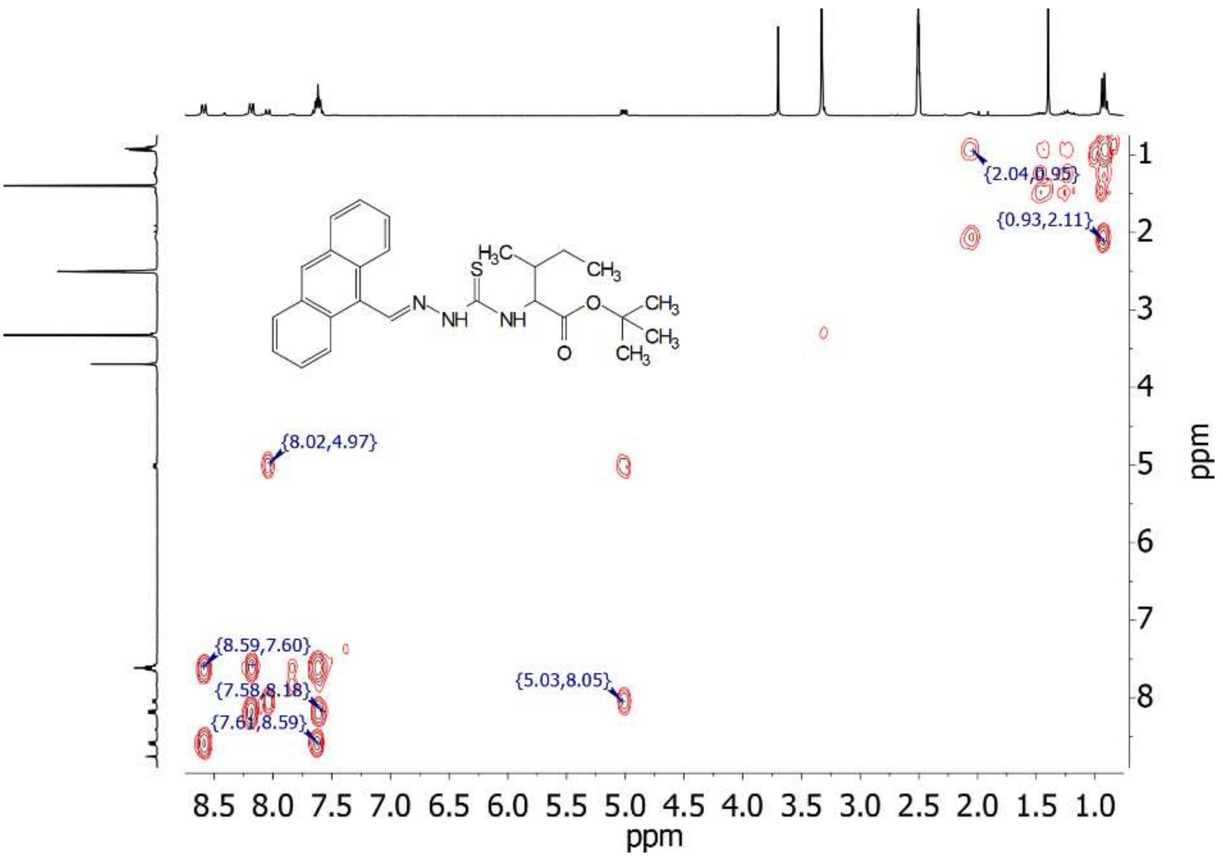

Figure S83. $^1H,^1H$ COSY NMR spectrum of *tert*-butyl-2-{[(1*E*)-(anthracen-9-yl)methylideneamino-carbamthioyl]amino}-3-methylpentanoate in DMSO-d_6 at 300 MHz.

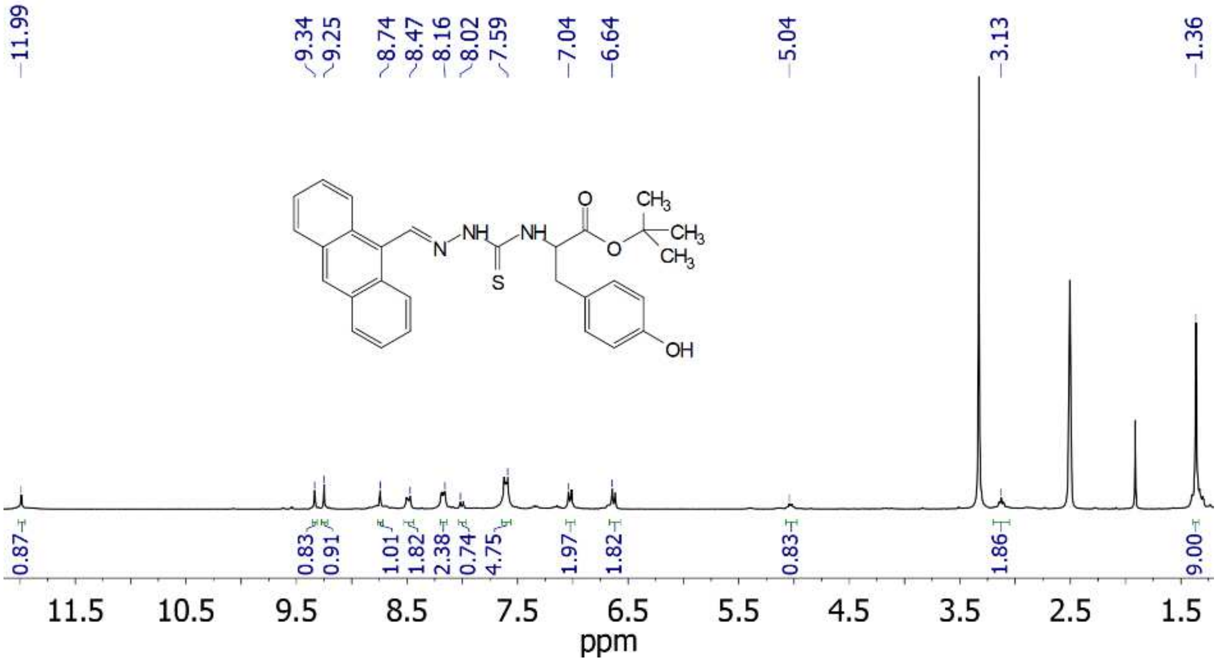

Figure S84. ^1H NMR spectrum of *tert*-butyl-2-[(2*E*)-2-[(anthracen-9-yl)methylidene]hydrazine-carbamthioyl]-3-(4-hydroxyphenyl) propanoate in DMSO-d_6 at 300 MHz.

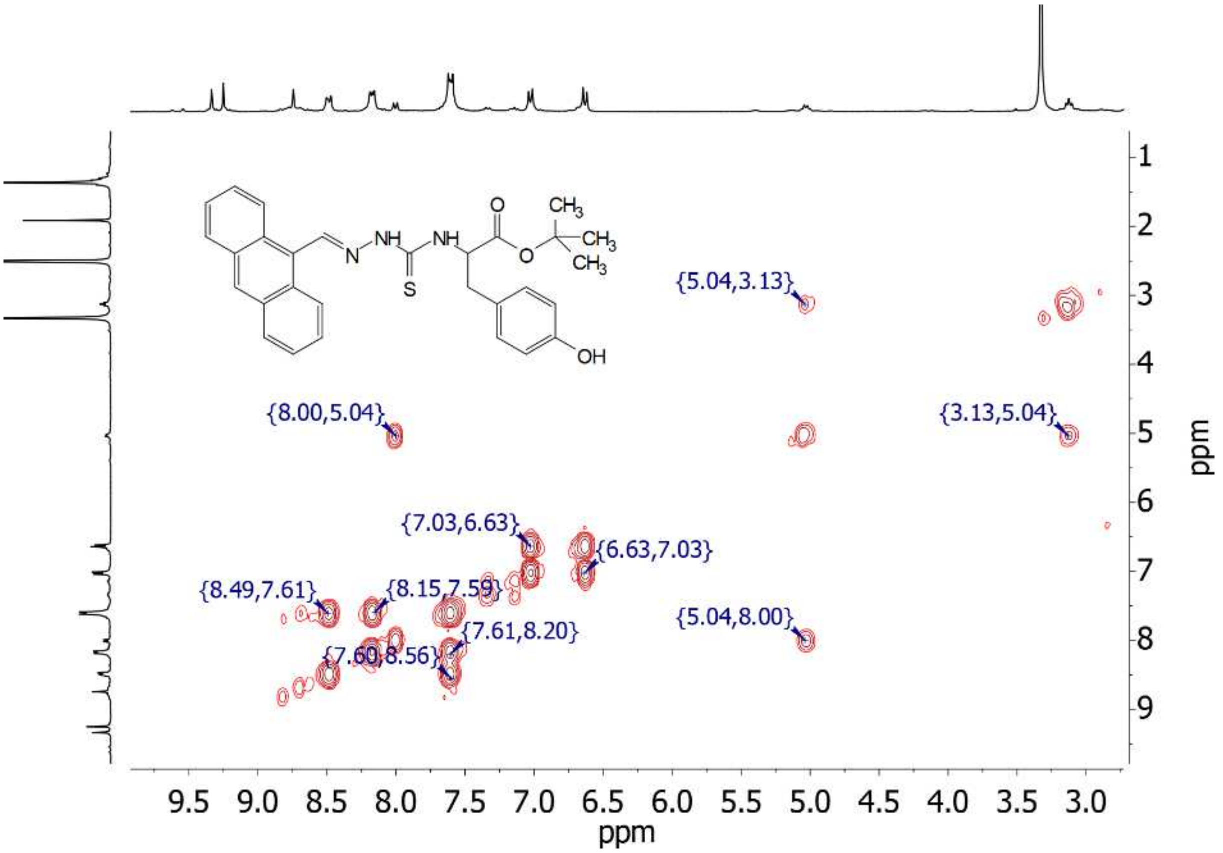

Figure S85. $^{1}H,^{1}H$ COSY NMR spectrum of *tert*-butyl-2-[(2*E*)-2-[(anthracen-9-yl)methylidene]-hydrazinecarbamthioyl]-3-(4-hydroxyphenyl) propanoate in DMSO-d_6 at 300 MHz.

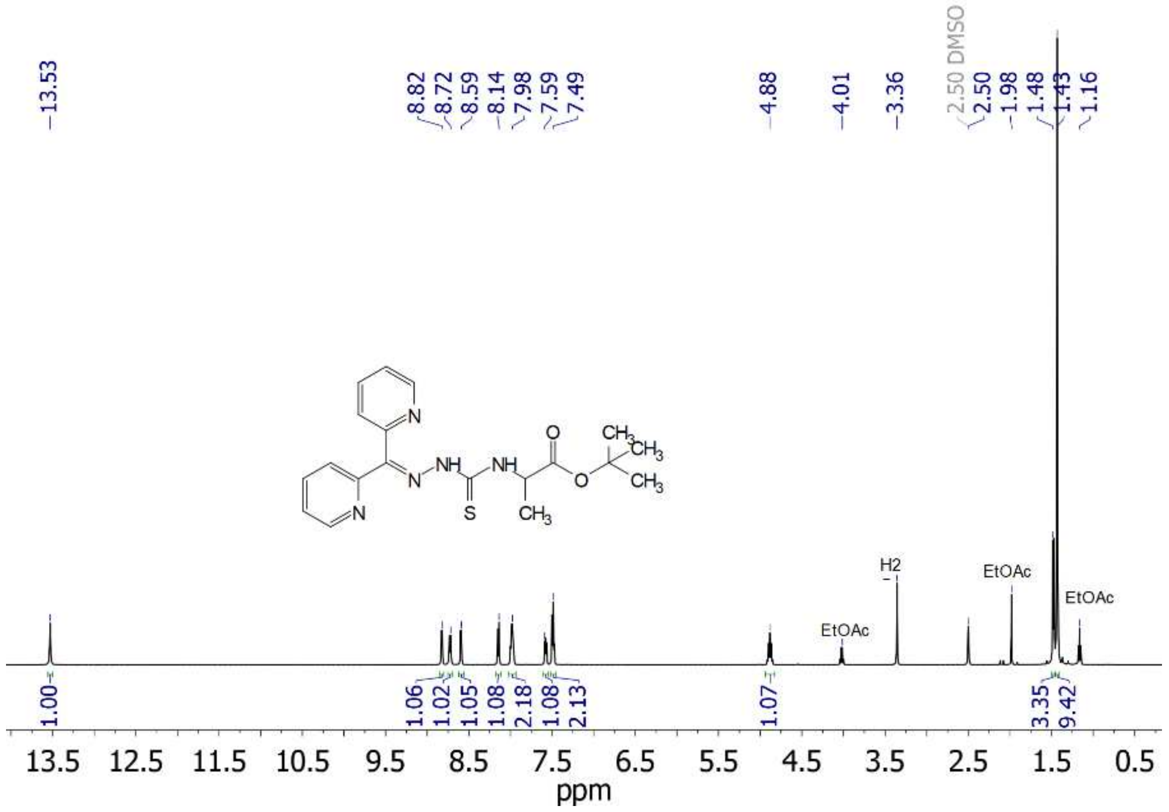

Figure S86. ^{1}H NMR spectrum of di-2-pyridylketone-4-((*S*)-*tert*-butyl propanoate)-3-thiosemicarbazone in DMSO-d_6 at 499 MHz.

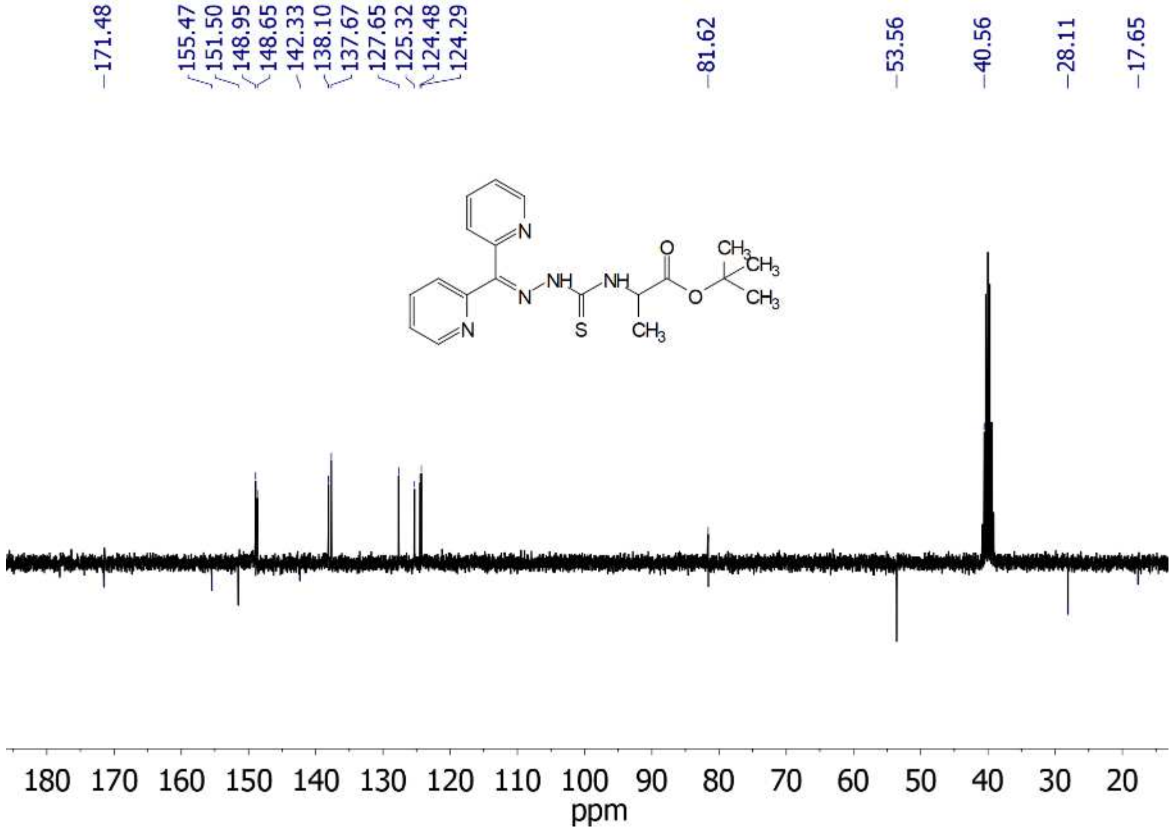

Figure S87. ^{13}C DEPTQ NMR spectrum of di-2-pyridylketone-4-((*S*)-*tert*-butyl propanoate)-3-thiosemicarbazone in DMSO-d_6 at 300 MHz.

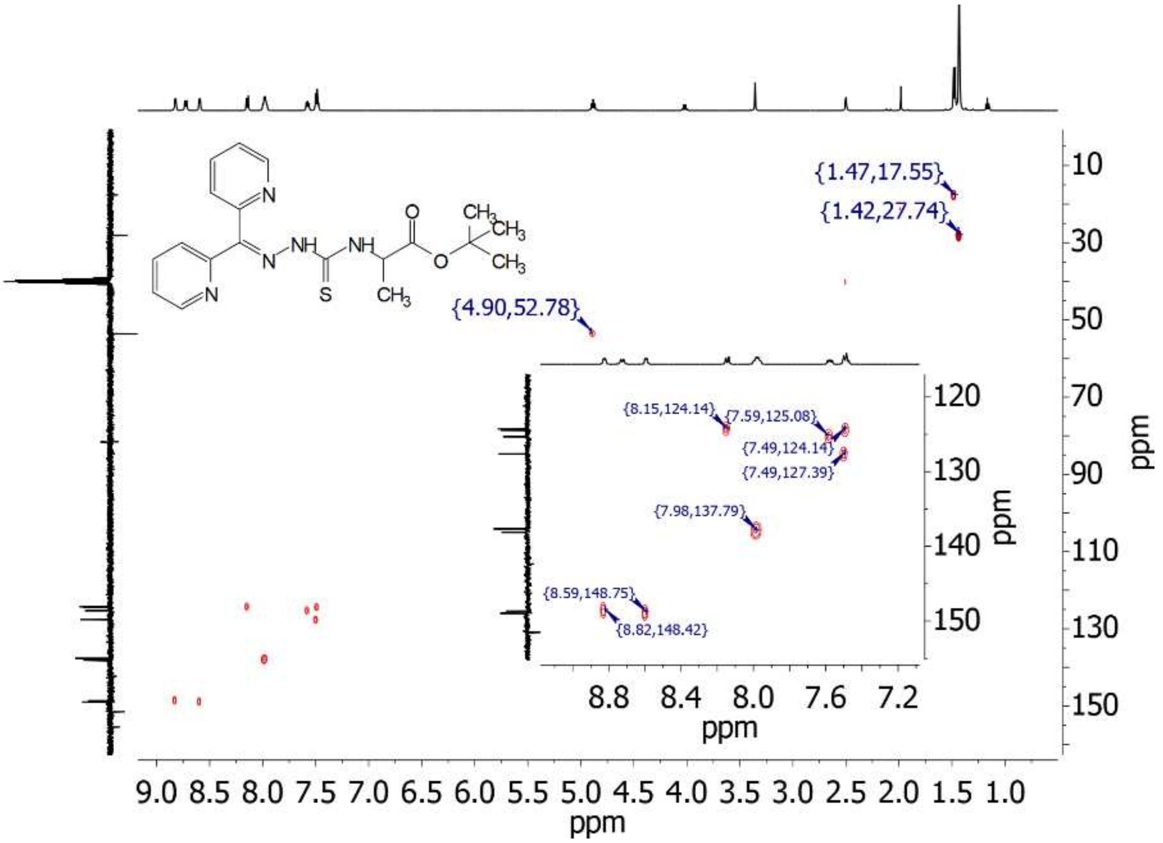

Figure S88. ^{1}H,^{13}C HMQC/HSQC NMR spectrum of di-2-pyridylketone-4-((*S*)-*tert*-butyl propanoate)-3-thiosemicarbazone in DMSO-d_6 at 499 MHz.

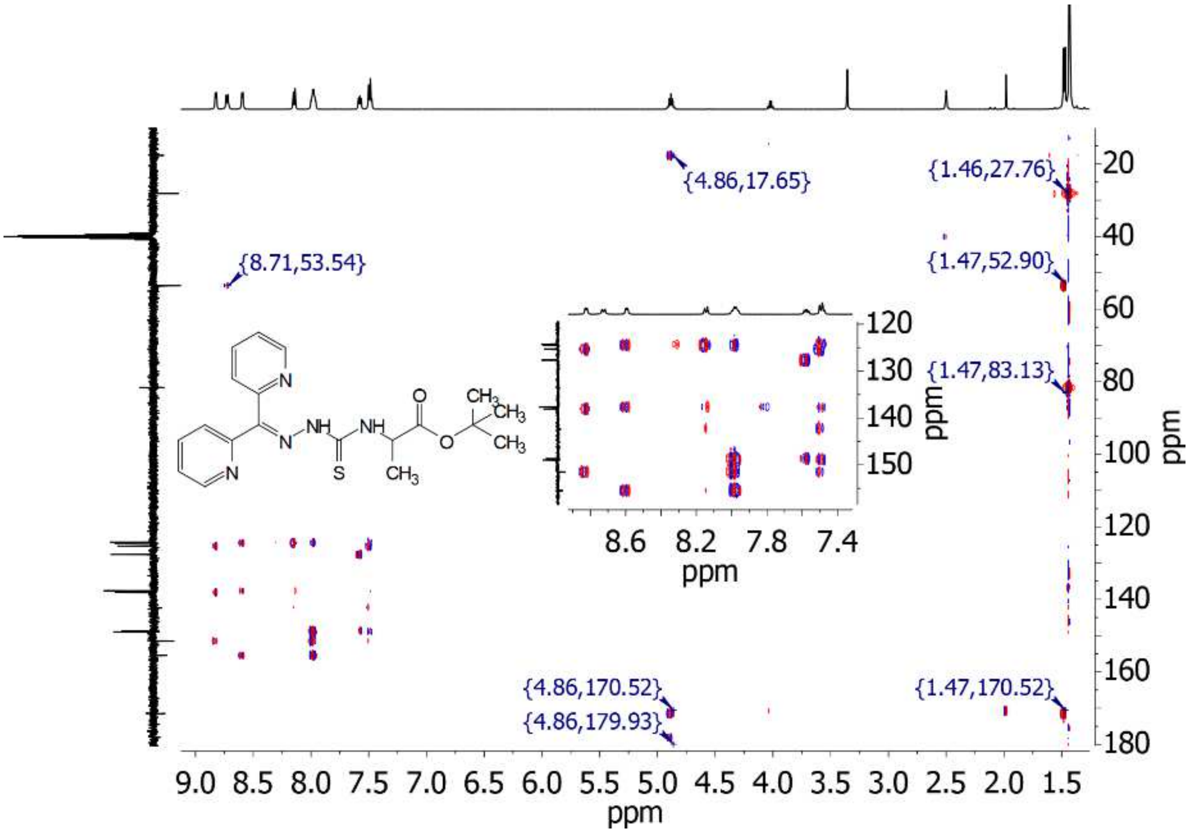

Figure S89. $^{1}H,^{13}C$ HMBC NMR spectrum of di-2-pyridylketone-4-((*S*)-*tert*-butyl propanoate)-3-thiosemicarbazone in DMSO-d_6 at 499 MHz.

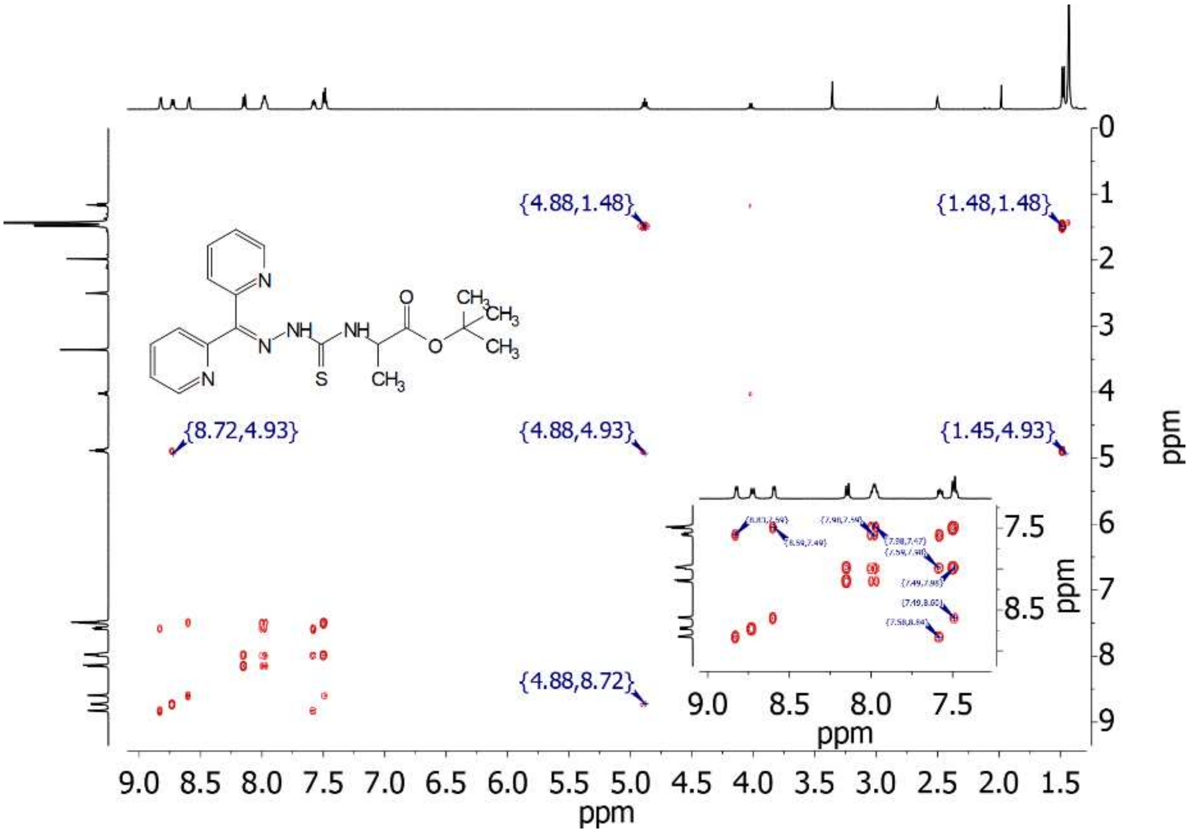

Figure S90. $^{1}H,^{1}H$ COSY NMR spectrum of di-2-pyridylketone-4-((*S*)-*tert*-butyl propanoate)-3-thiosemicarbazone in DMSO-d_6 at 499 MHz.

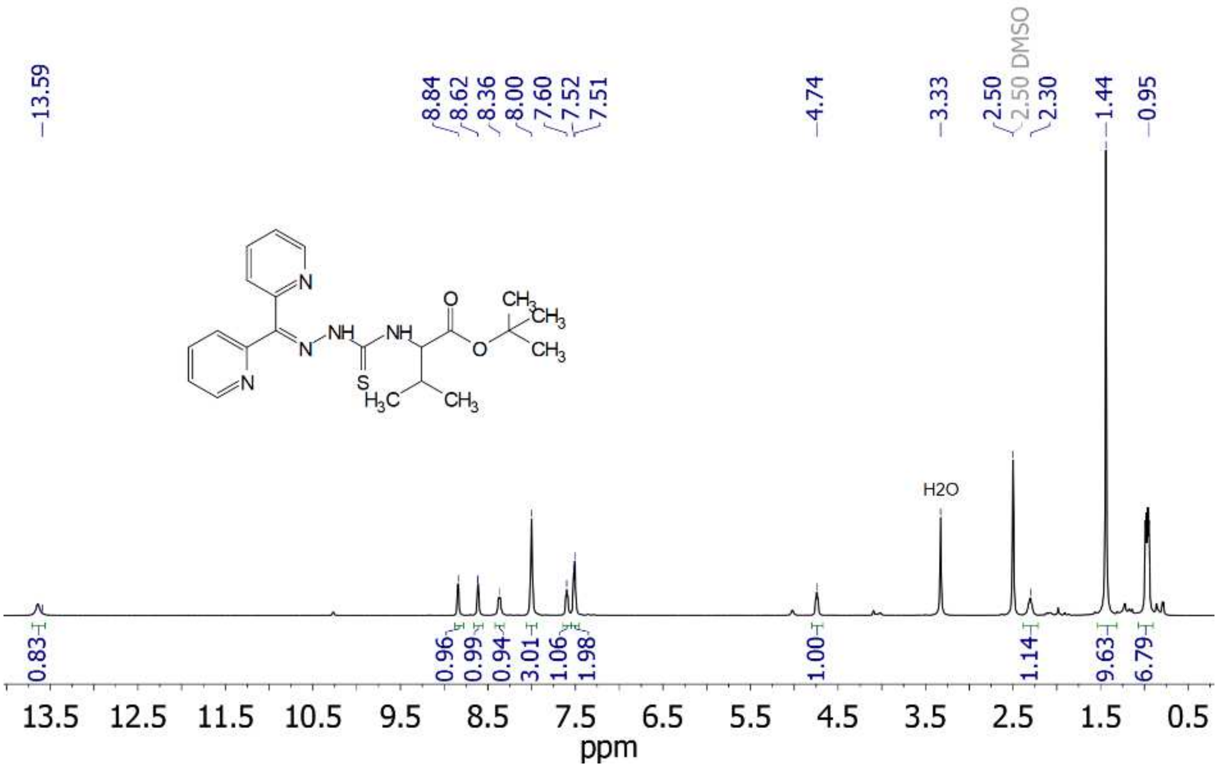

Figure S91. ^{1}H NMR spectrum of di-2-pyridylketone-4-((*S*)-*tert*-butyl 3-metylbutanoate)-3-thiosemicarbazone DMSO-d_6 at 499 MHz.

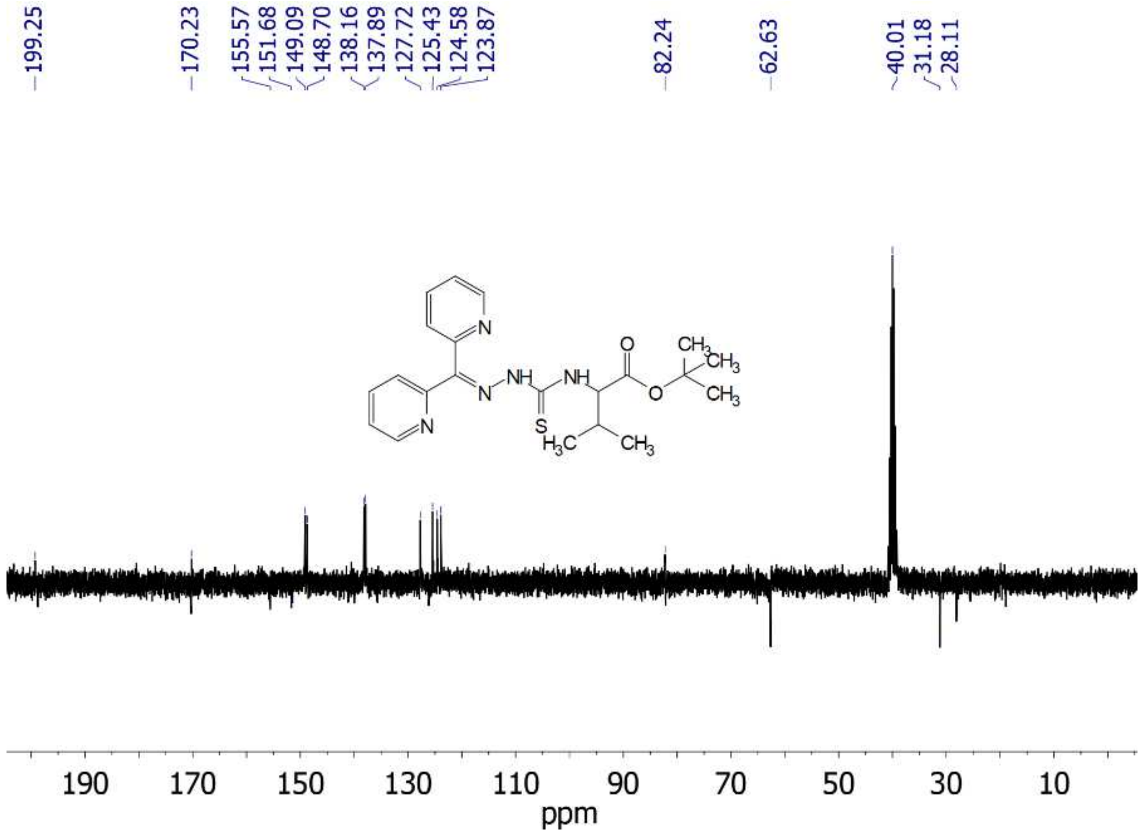

Figure S92. ^{13}C DEPTQ NMR spectrum of di-2-pyridylketone-4-((*S*)-*tert*-butyl 3-metylbutanoate)-3-thiosemicarbazone in DMSO-d_6 at 300 MHz.

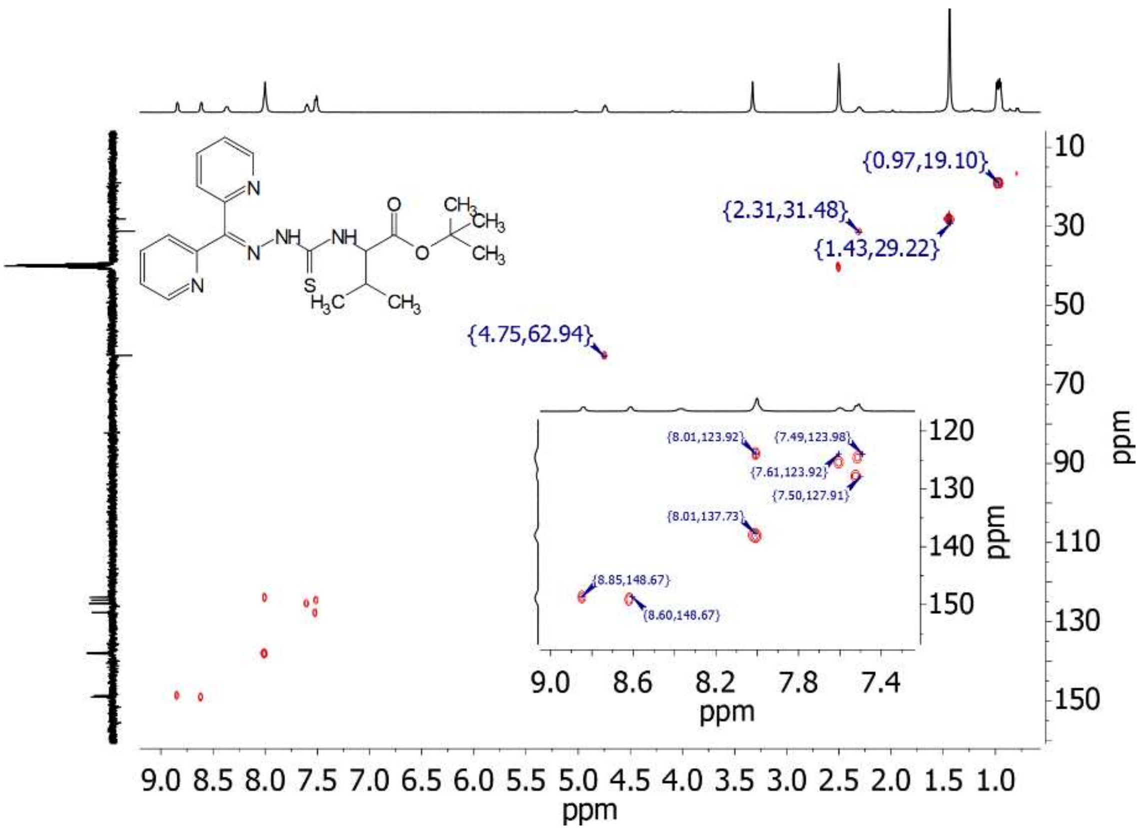

Figure S93. $^{1}H,^{13}C$ HMQC/HSQC NMR spectrum of di-2-pyridylketone-4-((*S*)-*tert*-butyl 3-metyl-butanoate)-3-thiosemicarbazone in DMSO-d_6 at 499 MHz.

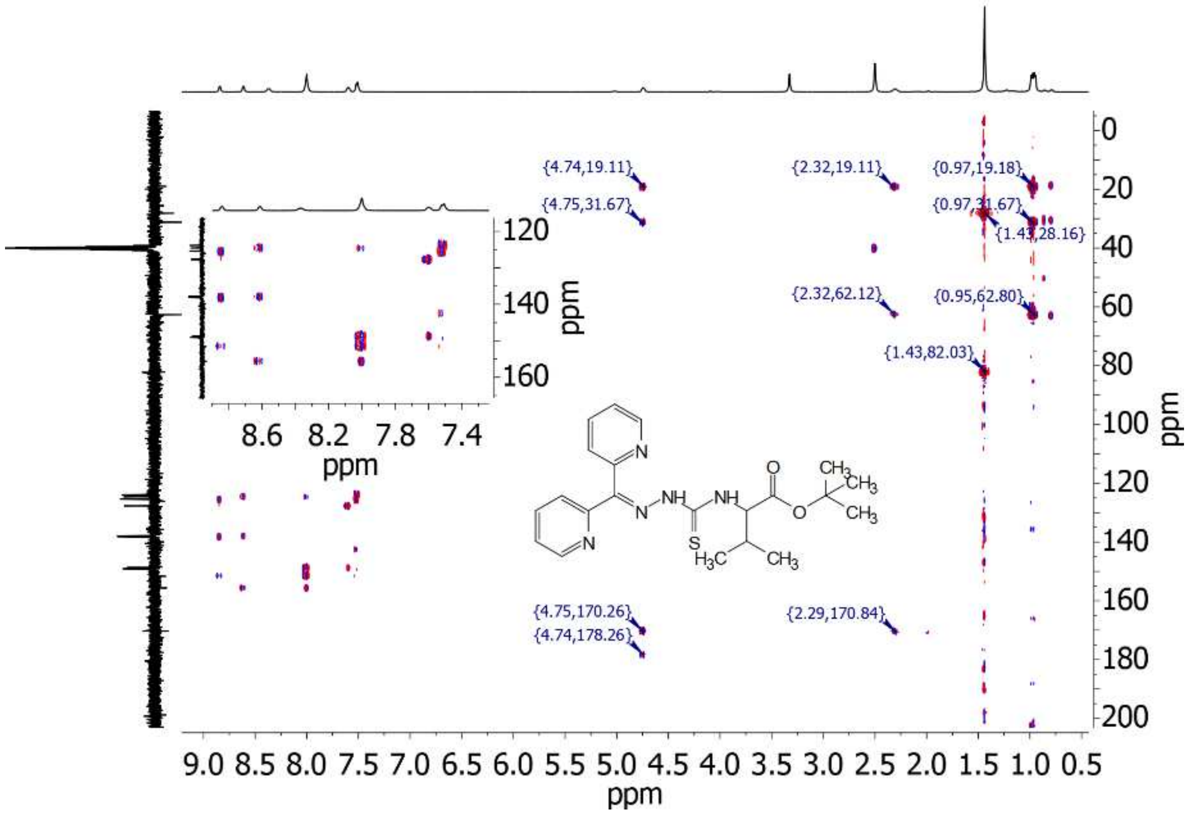

Figure S94. $^{1}H,^{13}C$ HMBC NMR spectrum of di-2-pyridylketone-4-((*S*)-*tert*-butyl 3-metylbutanoate)-3-thiosemicarbazone in DMSO-d_6 at 499 MHz.

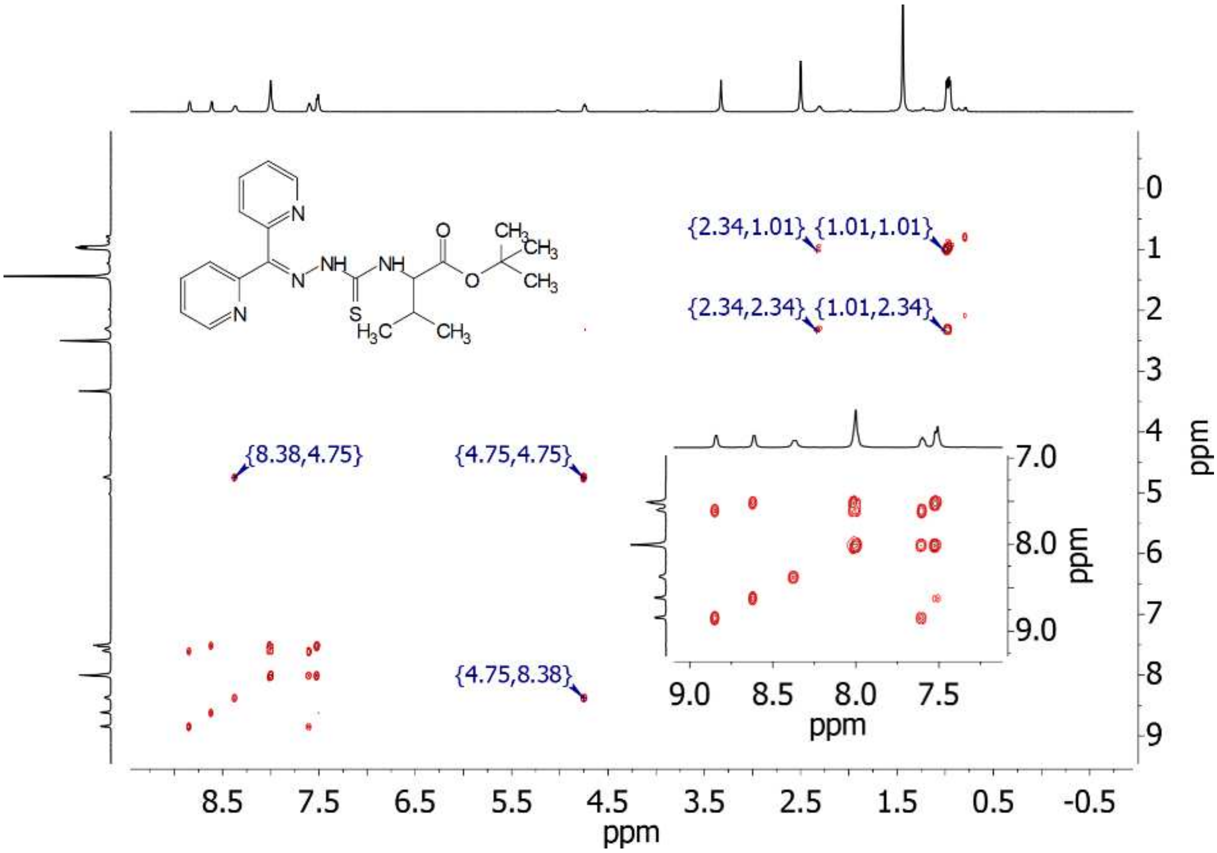

Figure S95. $^1H,^1H$ COSY NMR spectrum of di-2-pyridylketone-4-((*S*)-*tert*-butyl 3-metylbutanoate)-3-thiosemicarbazone in DMSO-d_6 at 499 MHz.

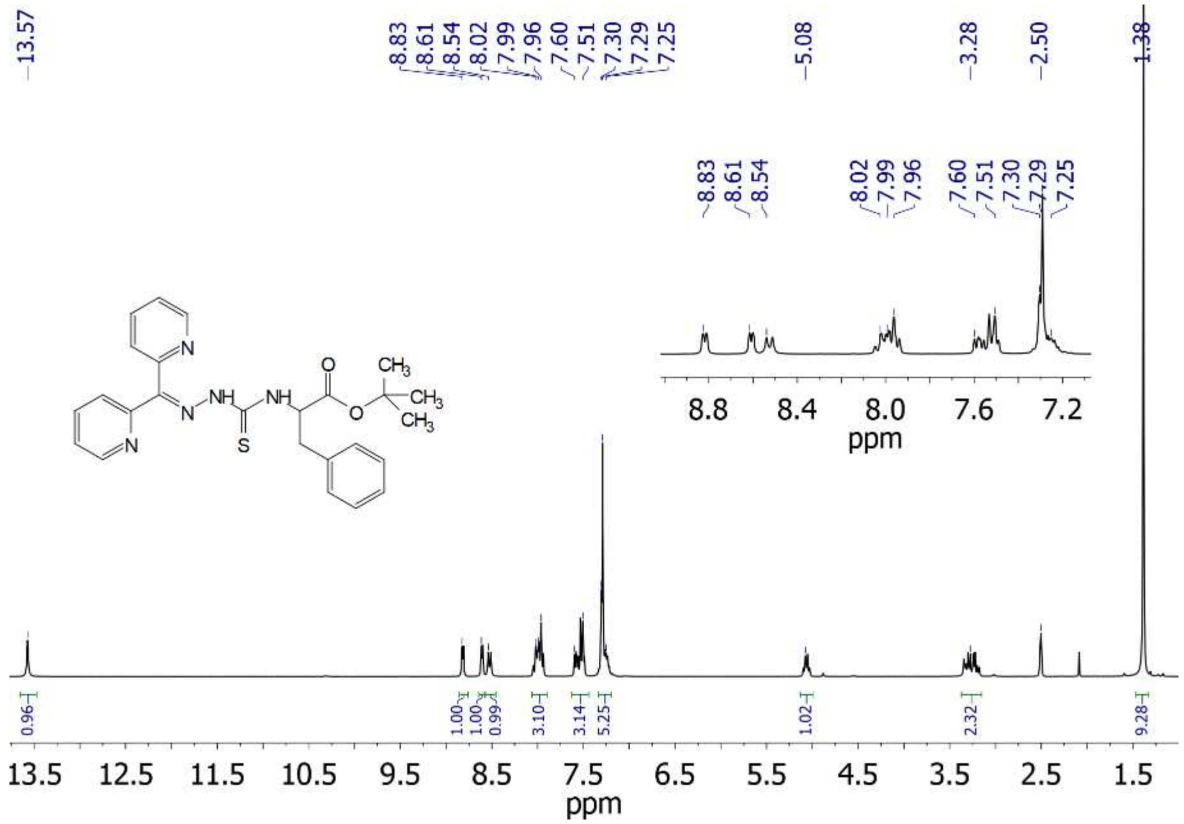

Figure S96. ^1H NMR spectrum of di-2-pyridylketone-4-((*S*)-*tert*-butyl 3-phenylbutanoate)-3-thiosemicarbazone in DMSO-d_6 at 499 MHz.

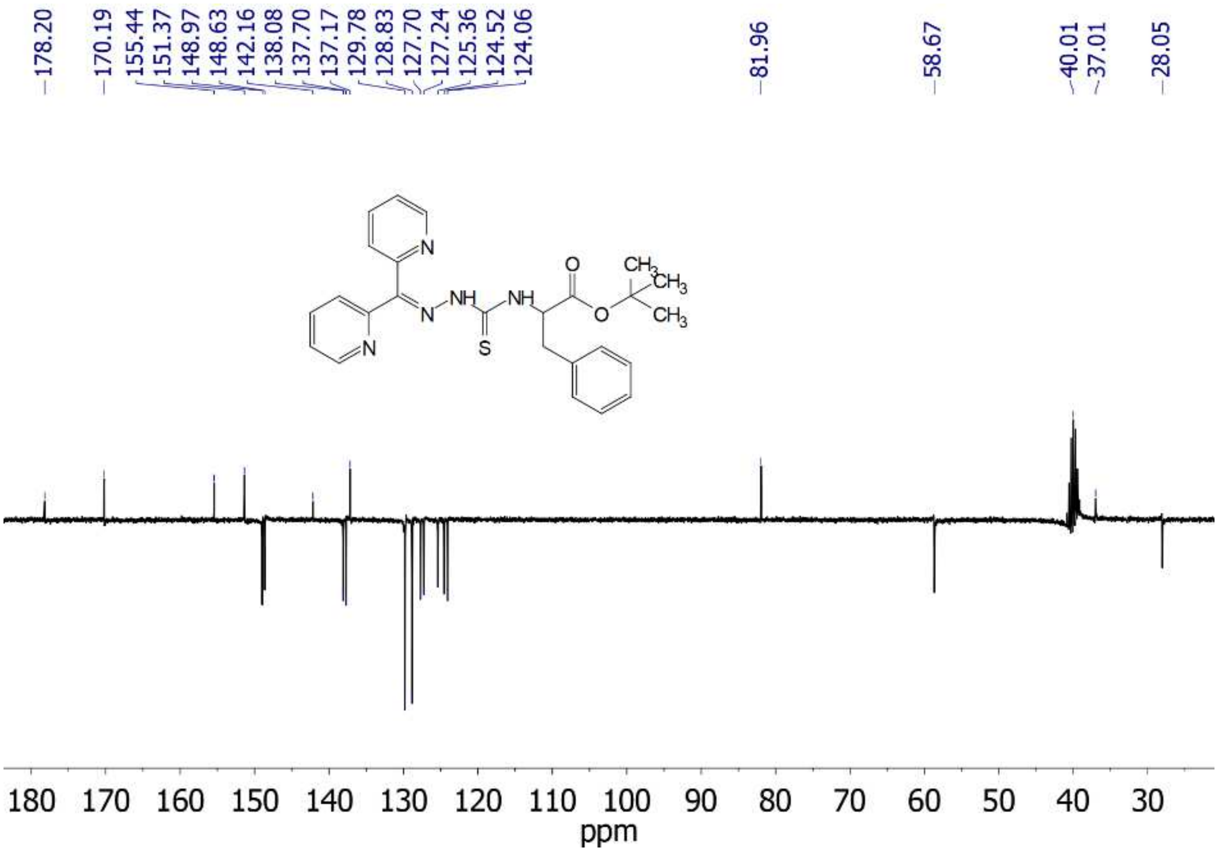

Figure S97. ^{13}C DEPTQ NMR spectrum of di-2-pyridylketone-4-((*S*)-*tert*-butyl 3-phenylbutanoate)-3-thiosemicarbazone in DMSO-d_6 at 300 MHz.

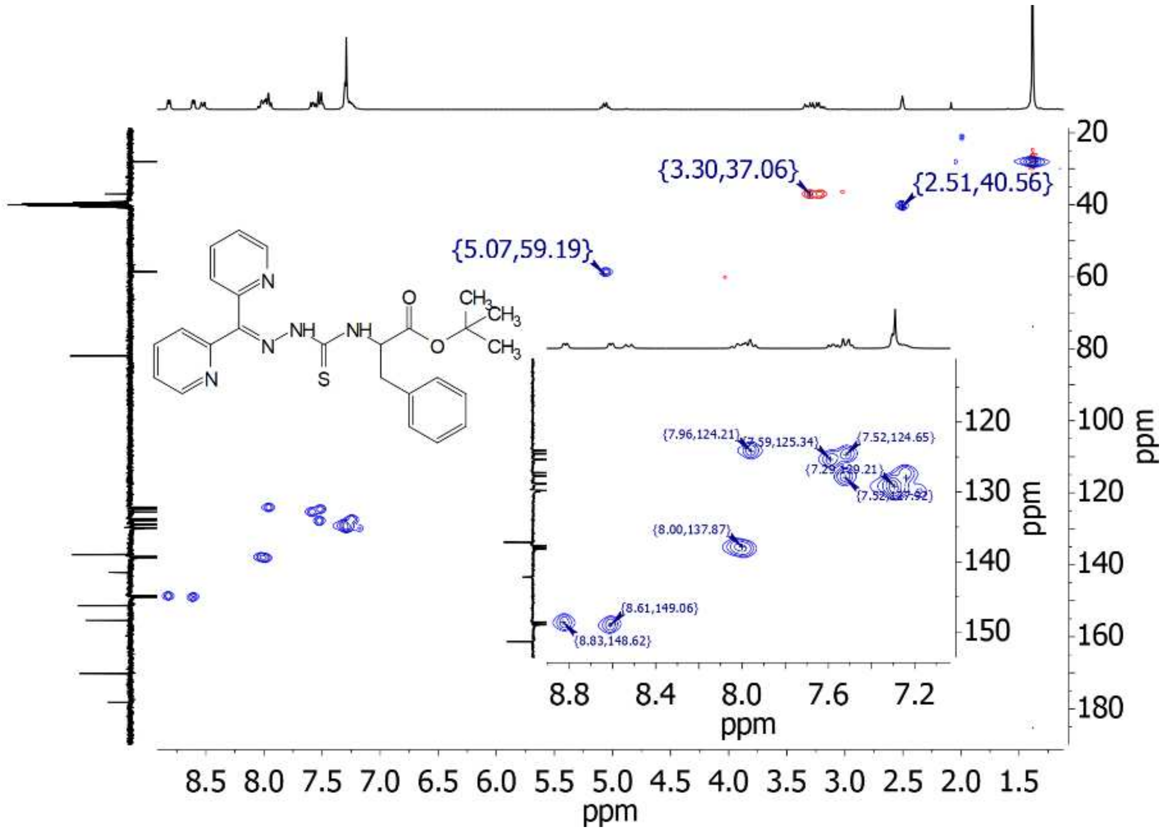

Figure S98. ^{1}H,^{13}C HMQC/HSQC NMR spectrum of di-2-pyridylketone-4-((*S*)-*tert*-butyl 3-phenylbutanoate)-3-thiosemicarbazone in DMSO-d_6 at 499 MHz.

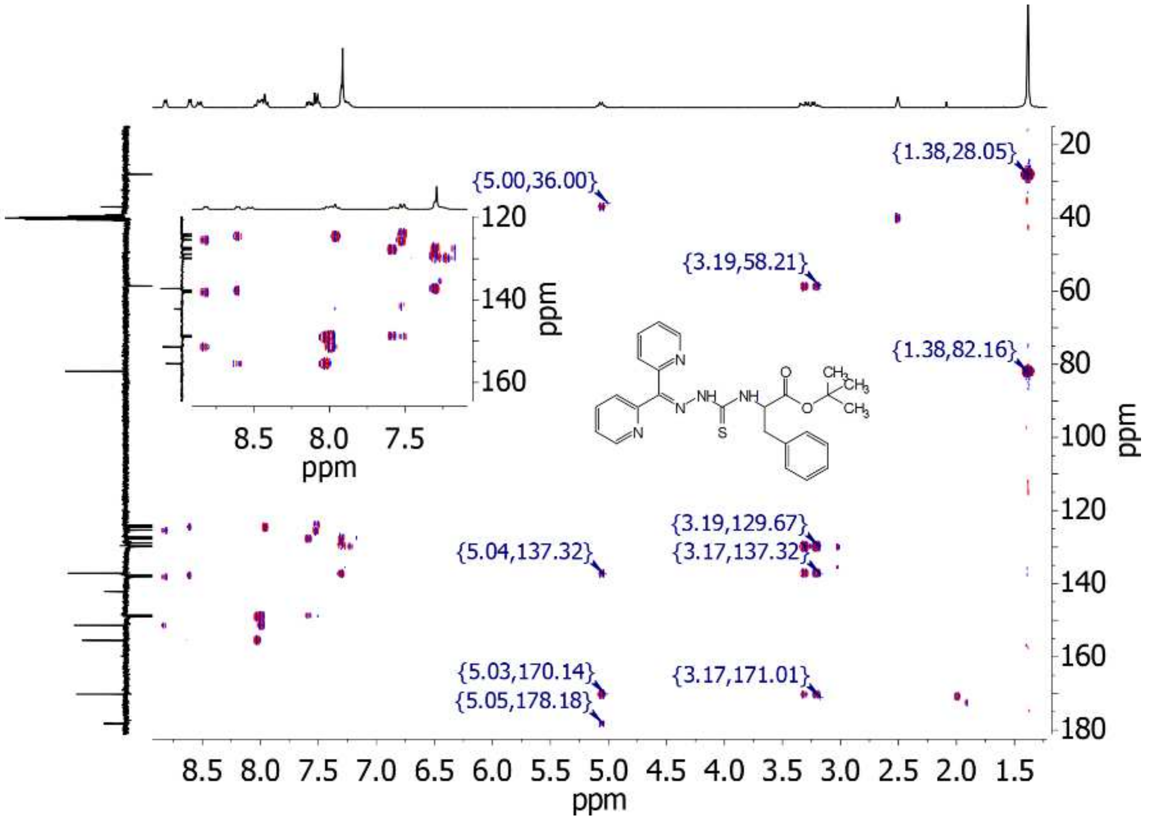

Figure S99. $^{1}H,^{13}C$ HMBC NMR spectrum of di-2-pyridylketone-4-((*S*)-*tert*-butyl 3-phenylbutanoate)-3-thiosemicarbazonein DMSO-d_6 at 499 MHz.

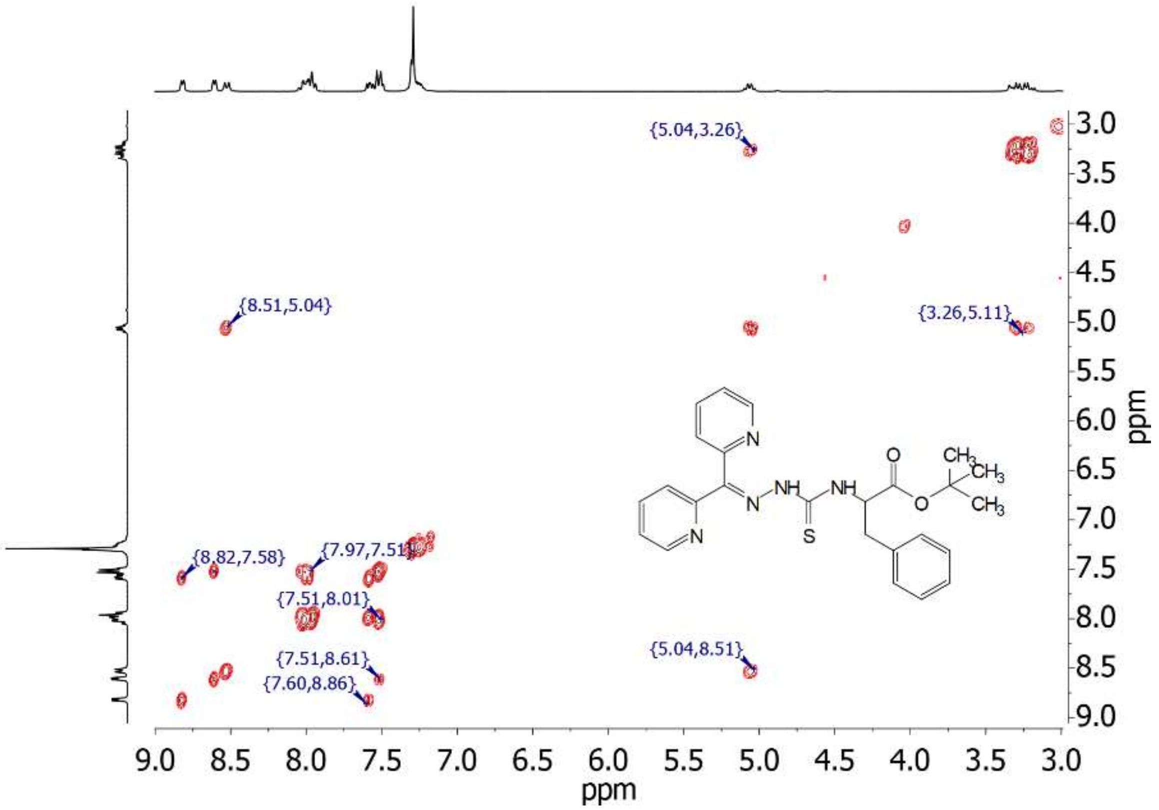

Figure S100. $^{1}H,^{1}H$ COSY NMR spectrum of di-2-pyridylketone-4-((*S*)-*tert*-butyl 3-phenylbutanoate)-3-thiosemicarbazone in DMSO-d_6 at 499 MHz.

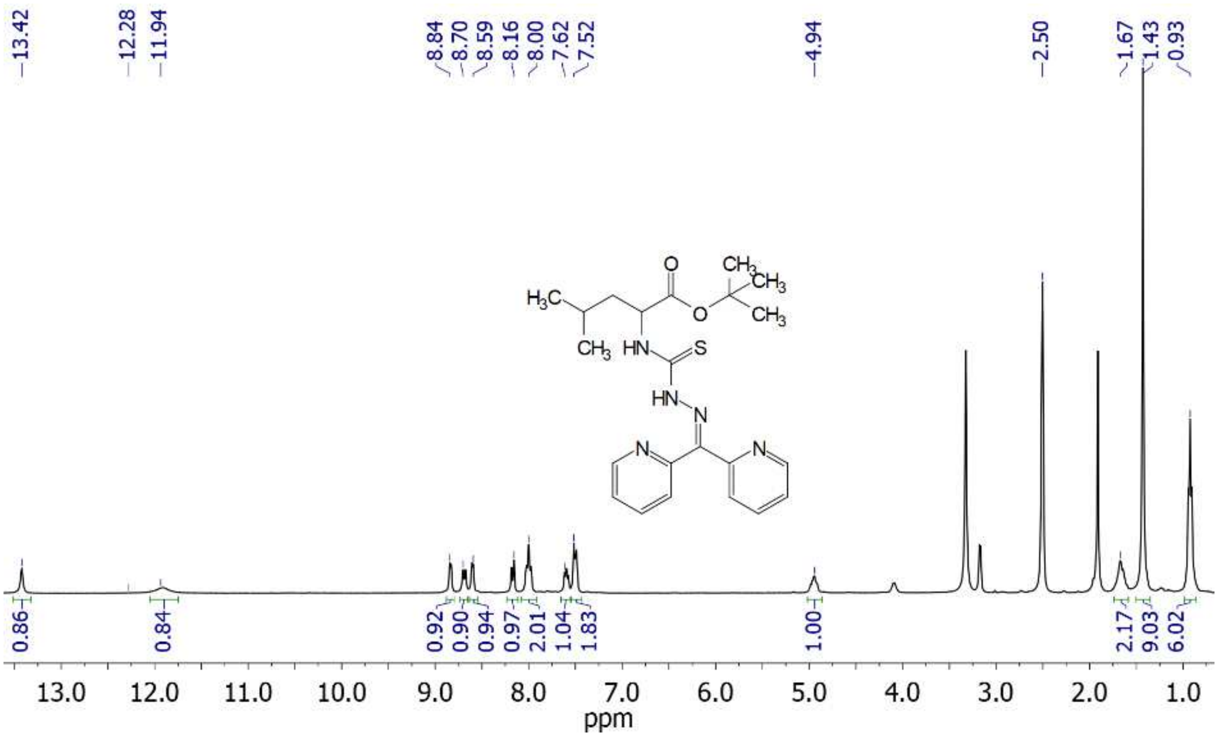

Figure S101. ^{1}H NMR spectrum of di-2-pyridylketone-4-((*S*)-*tert*-butyl-4-methylpentanoate)-3-thiosemicarbazone in DMSO-d_6 at 300 MHz.

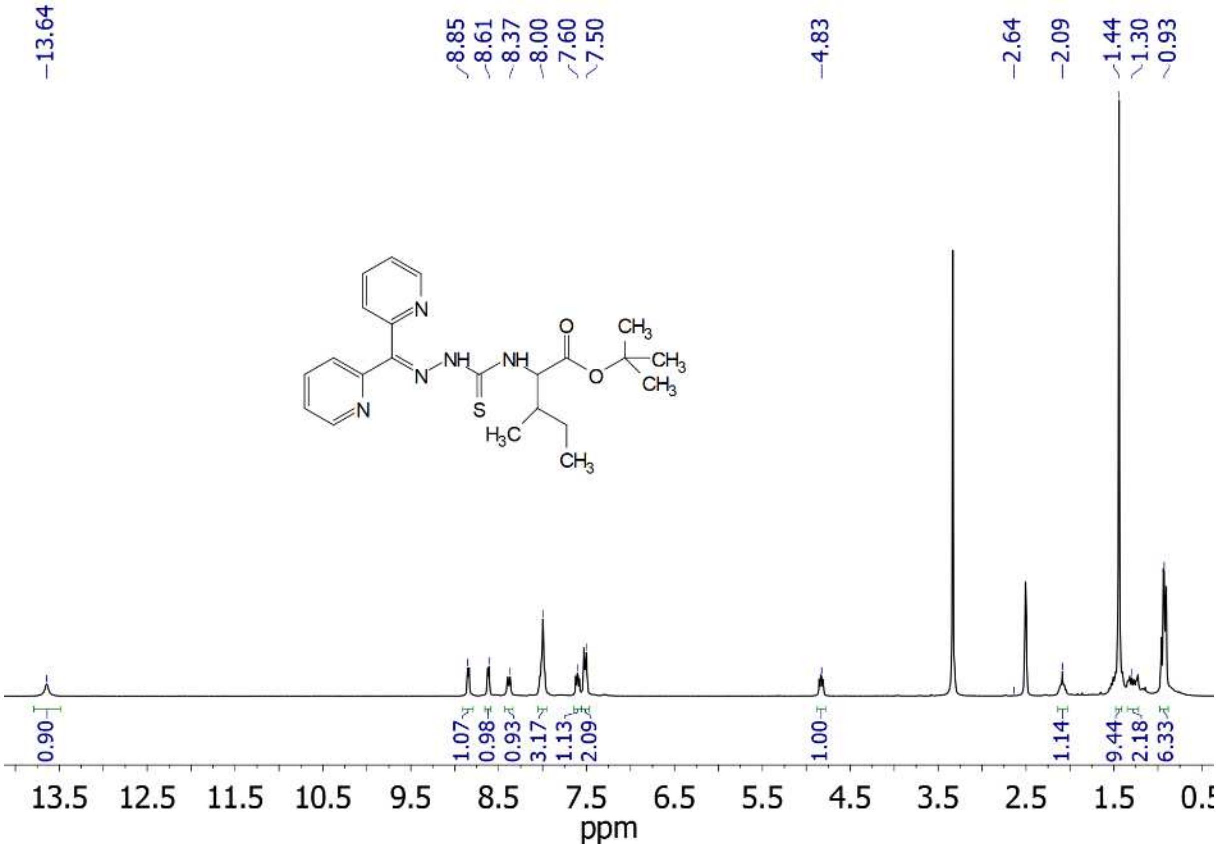

Figure S102. ^{1}H NMR spectrum of *tert*-butyl 2-{2-[bis(pyridin-2-yl)methylidene]hydrazine-carbamthioyl}-3-methylpentanoate in DMSO-d_6 at 300 MHz.

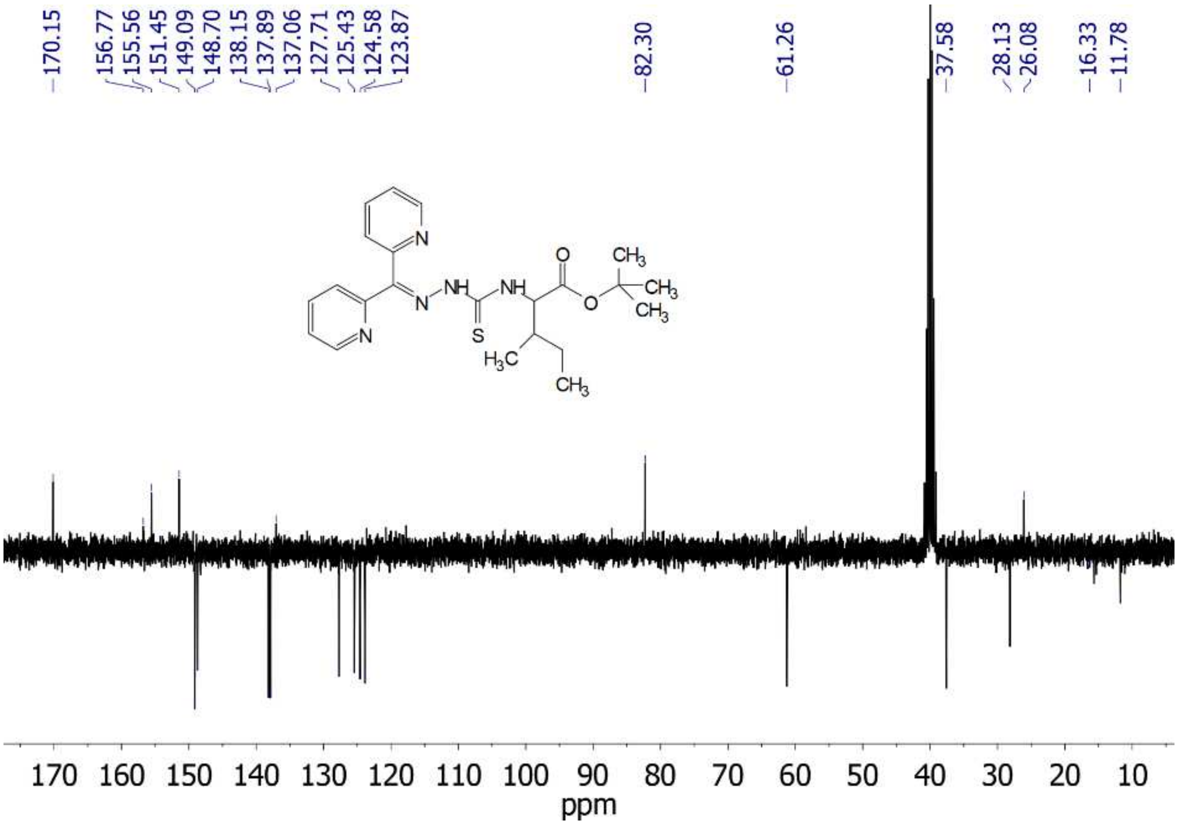

Figure S103. ^{13}C DEPTQ NMR spectrum of *tert*-butyl 2-{2-[bis(pyridin-2-yl)methylidene]hydrazine-carbamthioyl}-3-methylpentanoate in DMSO-d_6 at 300 MHz.

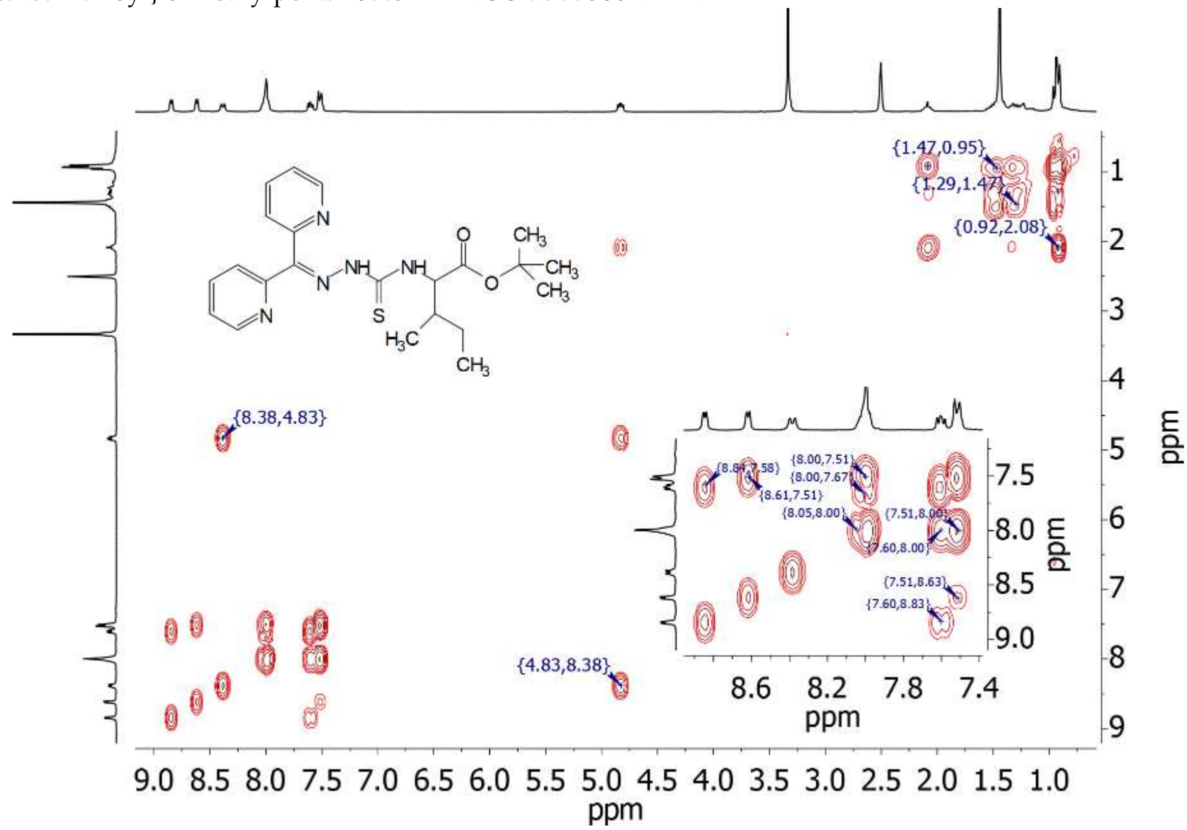

Figure S104. $^1H,^1H$ COSY NMR spectrum of *tert*-butyl 2-{2-[bis(pyridin-2-yl)methylidene]hydrazine-carbamthioyl}-3-methylpentanoate in DMSO-d_6 at 300 MHz.

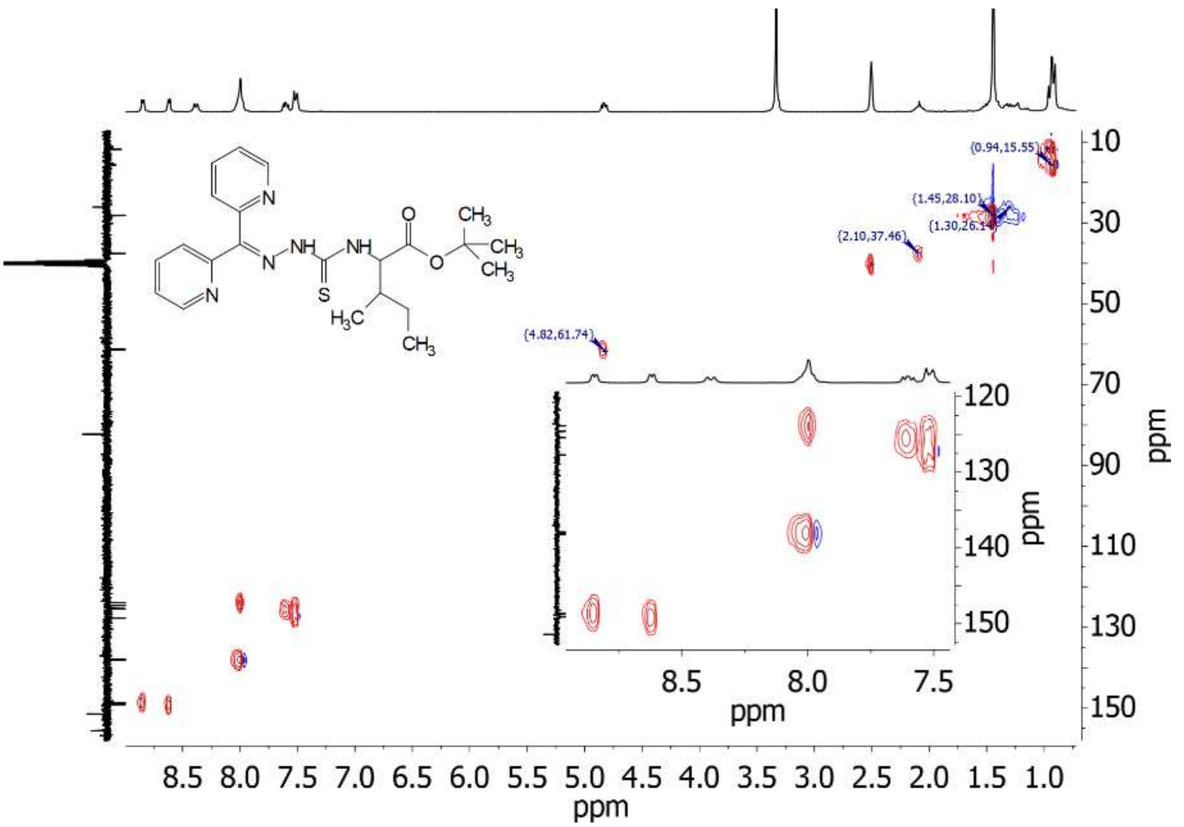

Figure S105. $^{1}H,^{13}C$ HMQC/HSQC spectrum of *tert*-butyl 2-{2-[bis(pyridin-2-yl)methylidene]-hydrazinecarbamthioyl}-3-methylpentanoate in DMSO-d_6 at 300 MHz.

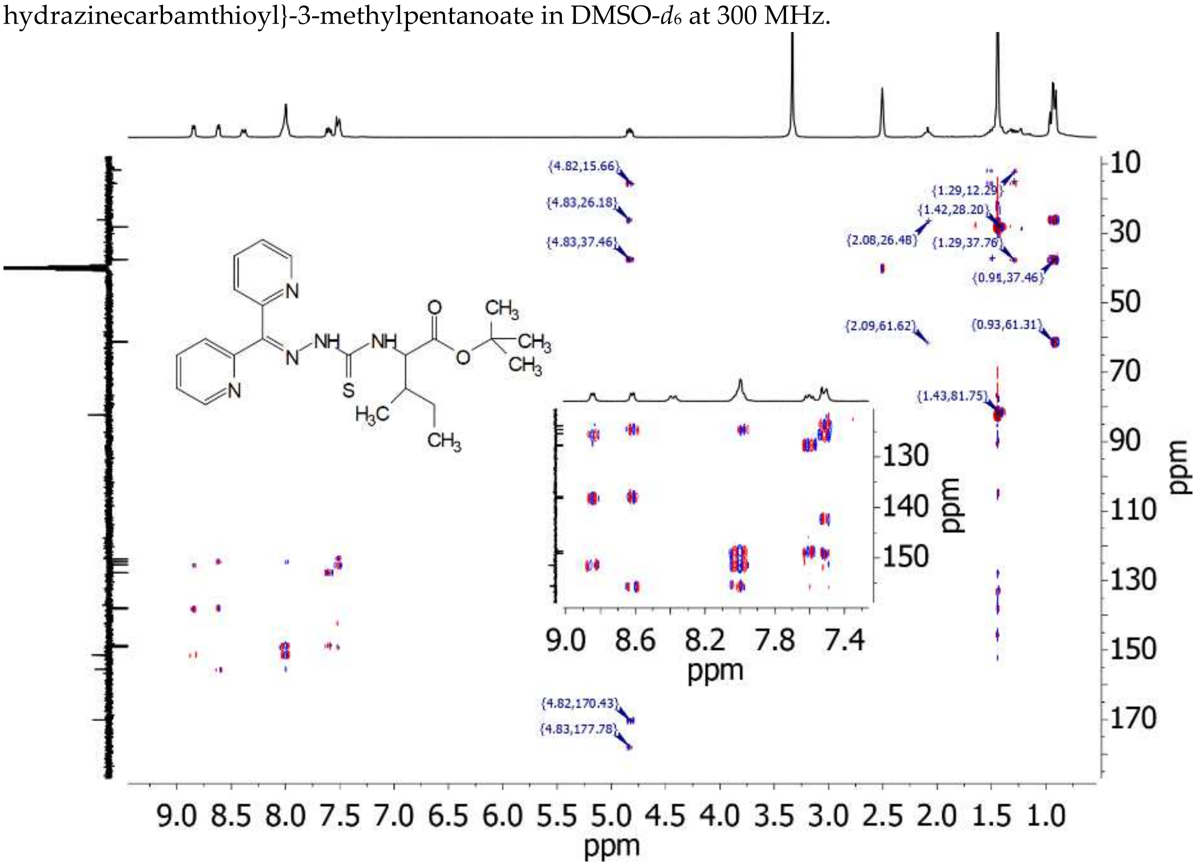

Figure S106. $^{1}H,^{13}C$ HMBC NMR spectrum of *tert*-butyl 2-{2-[bis(pyridin-2-yl)methylidene]hydrazine-carbamthioyl}-3-methylpentanoate in DMSO-d_6 at 300 MHz.

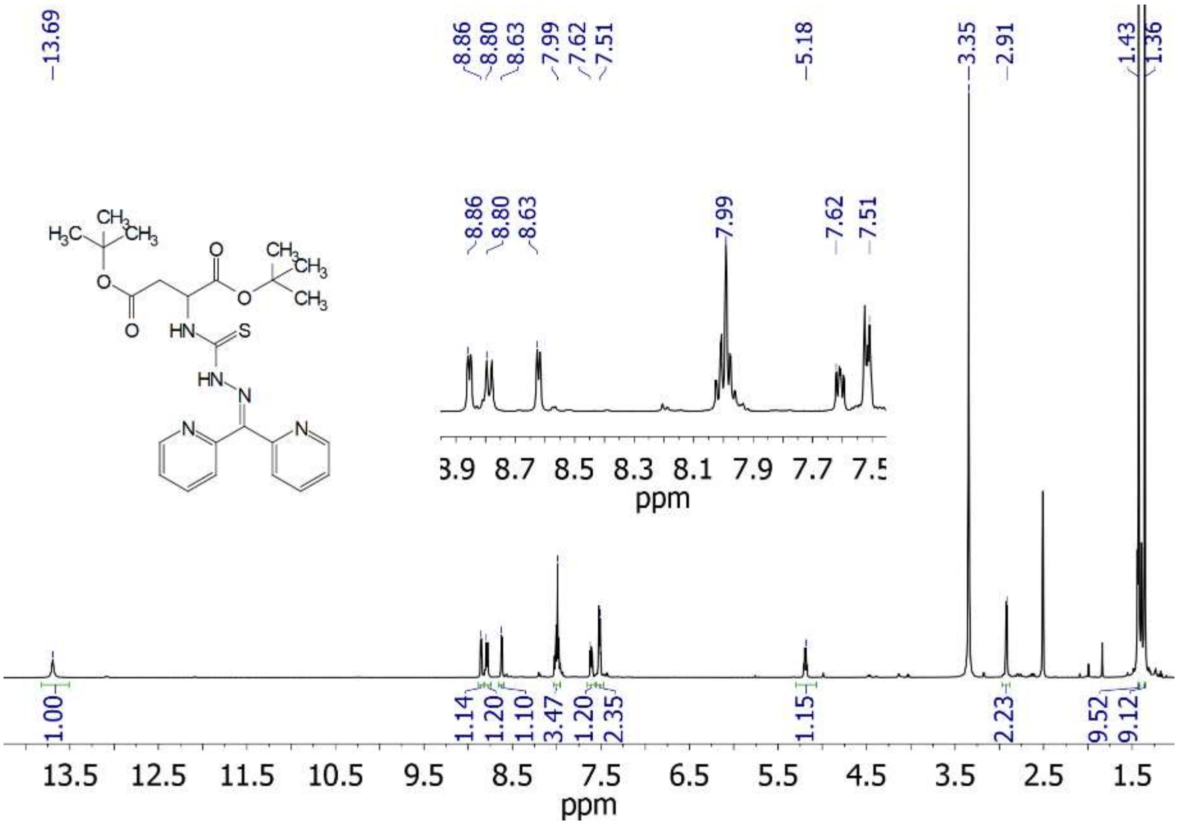

Figure S107. ^{1}H NMR spectrum of di-*tert*-butyl 2-{[bis(pyridin-2-yl)methylideneamino-carbamthioyl]-amino}butanedioate in DMSO-d_6 at 499 MHz.

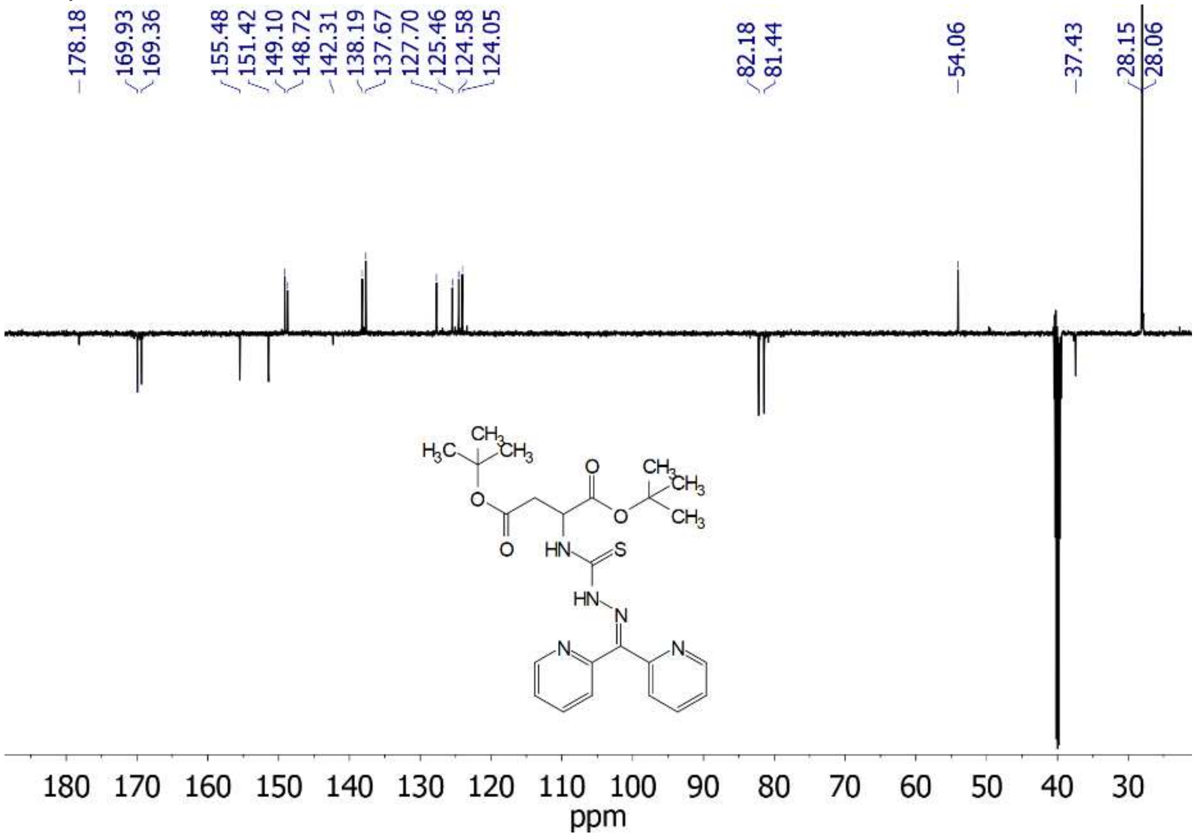

Figure S108. ^{13}C APT NMR spectrum of di-*tert*-butyl 2-{[bis(pyridin-2-yl)methylideneaminocarbam-thioyl]amino}butanedioate in DMSO-d_6 at 499 MHz.

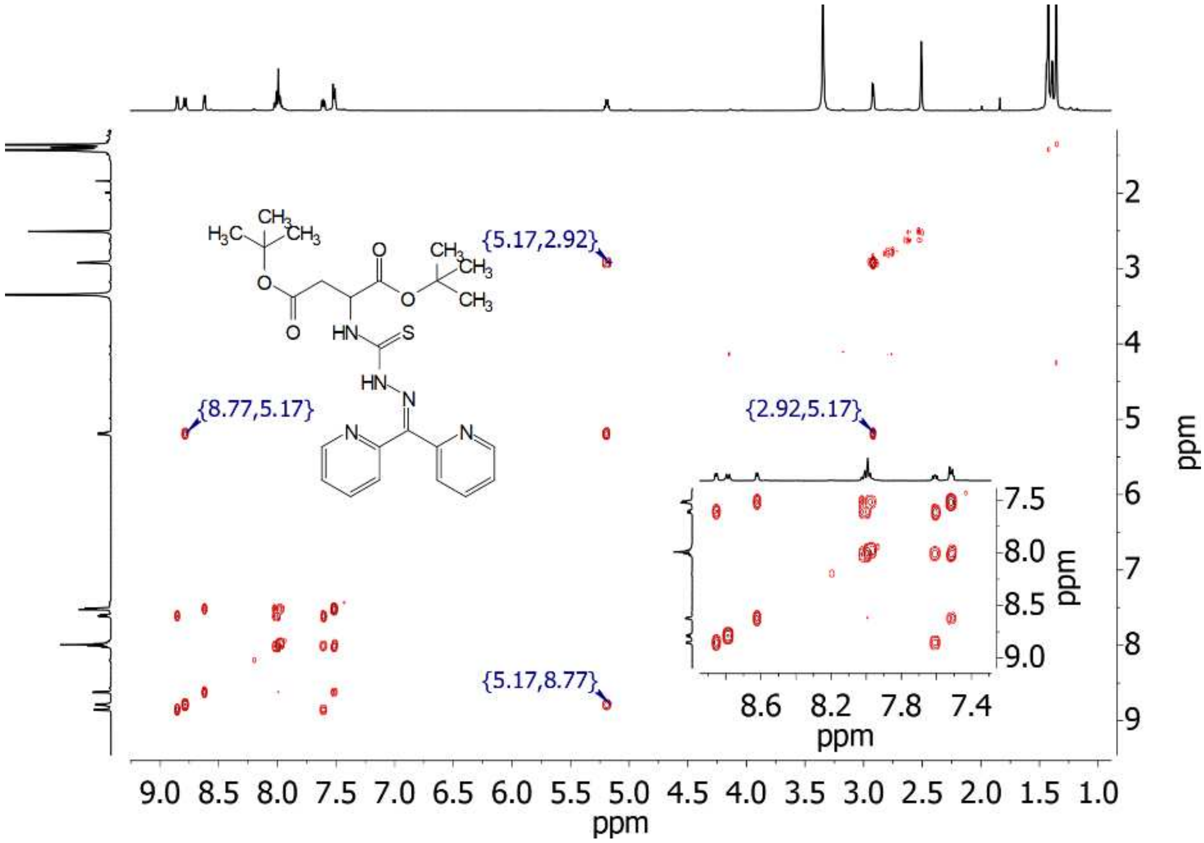

Figure S109. $^{1}H,^{1}H$ COSY NMR spectrum of di-*tert*-butyl 2-{[bis(pyridin-2-yl)methylideneamino-carbamthioyl]amino}butanedioate in DMSO-d_6 at 499 MHz.

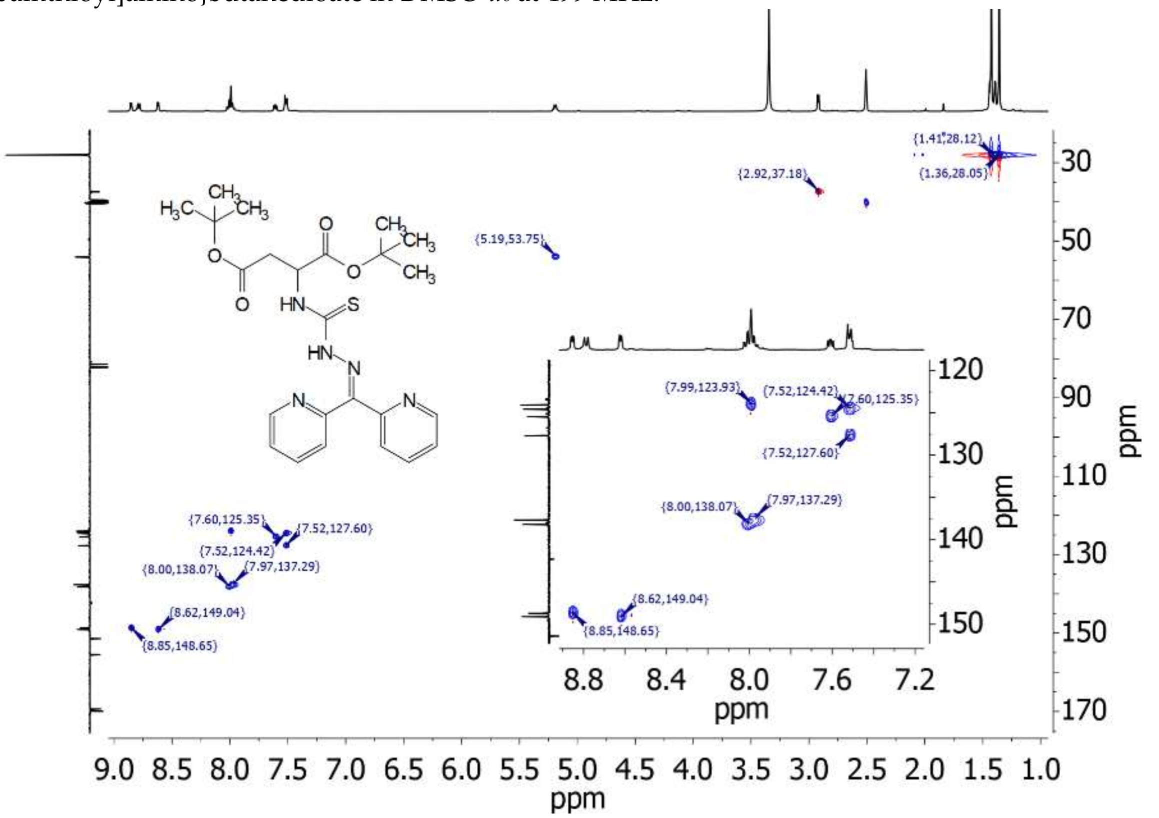

Figure S110. $^{1}H,^{13}C$ HMQC/HSQC NMR spectrum of di-*tert*-butyl 2-{[bis(pyridin-2-yl)methylidene-aminocarbamthioyl]amino}butanedioate in DMSO-d_6 at 499 MHz.

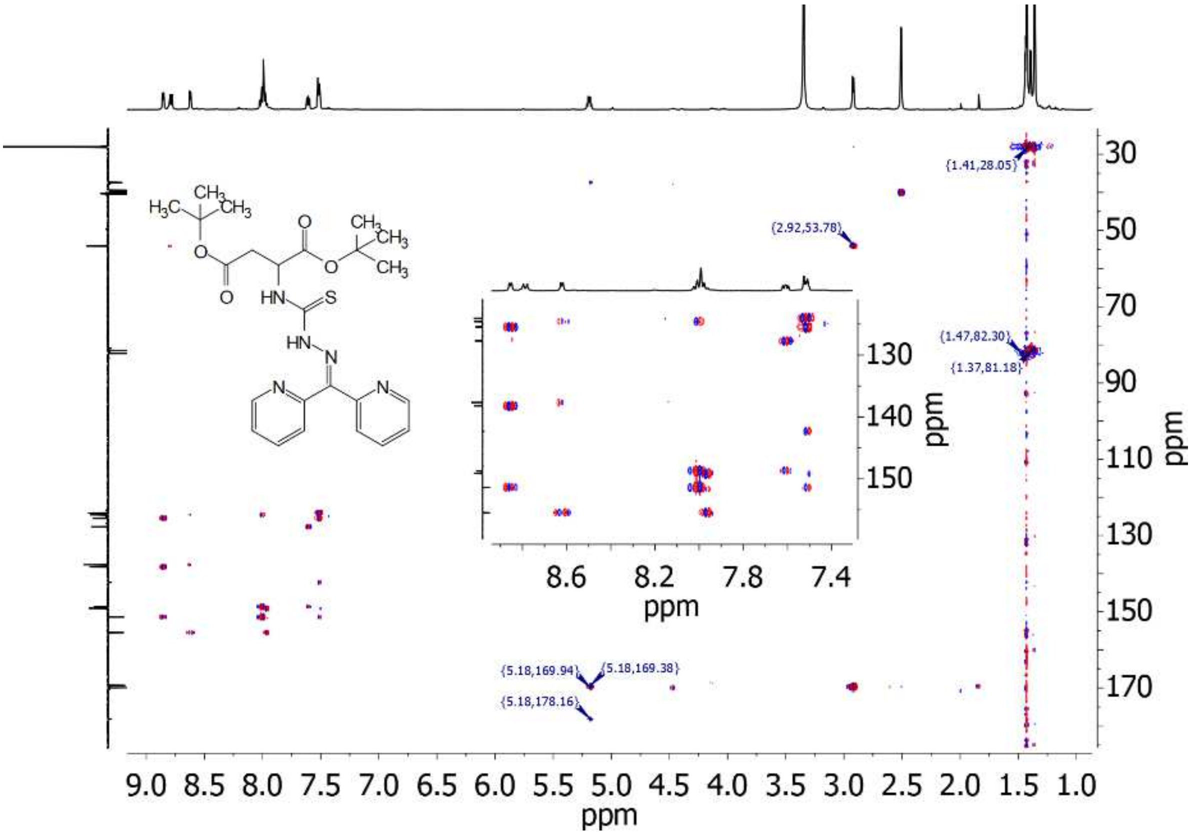

Figure S111. $^{1}H,^{13}C$ HMBC NMR spectrum of di-*tert*-butyl 2-{[bis(pyridin-2-yl)methylideneamino-carbamthioyl]amino}butanedioate in DMSO-d_6 at 499 MHz.

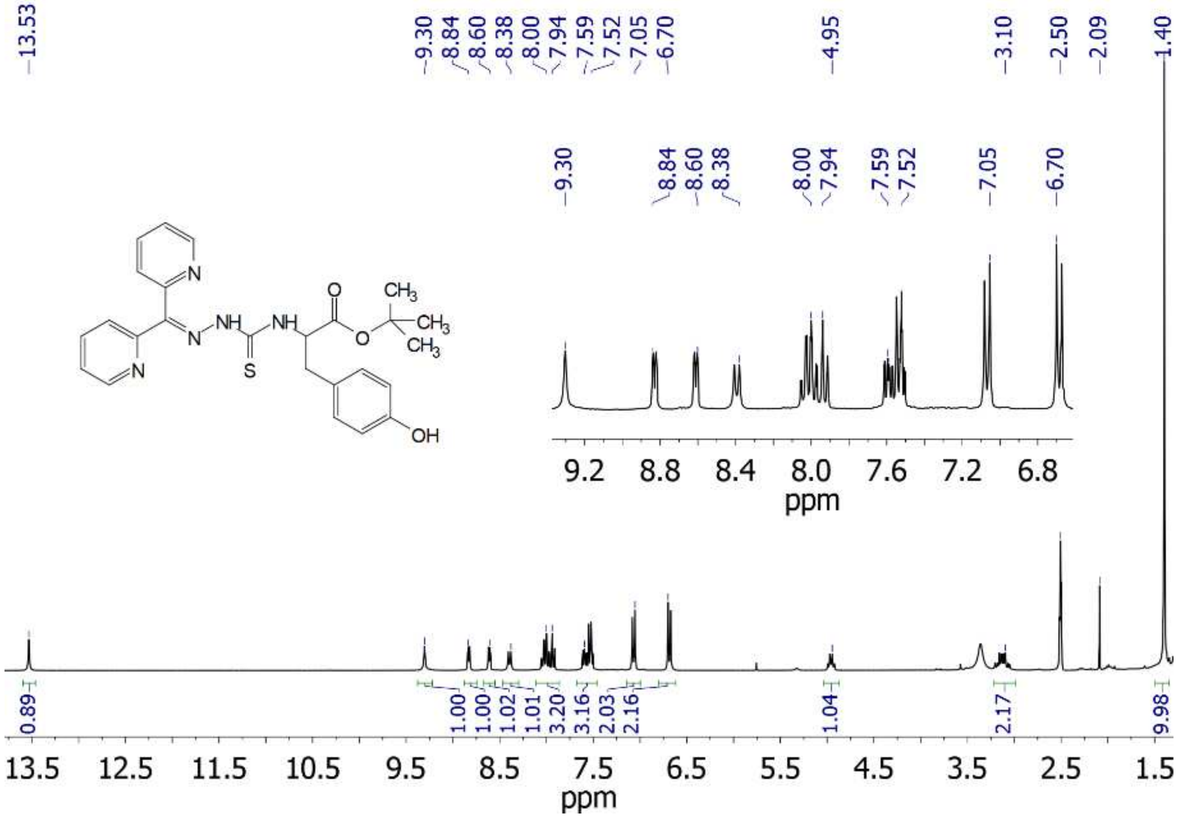

Figure S112. ^{1}H NMR spectrum of t*ert*-butyl-2-{2-[bis(pyridin-2-yl)methylidene]hydrazine-carbamthioyl}-3-(4-hydroxyphenyl)-propanoate in DMSO-d_6 at 300 MHz.

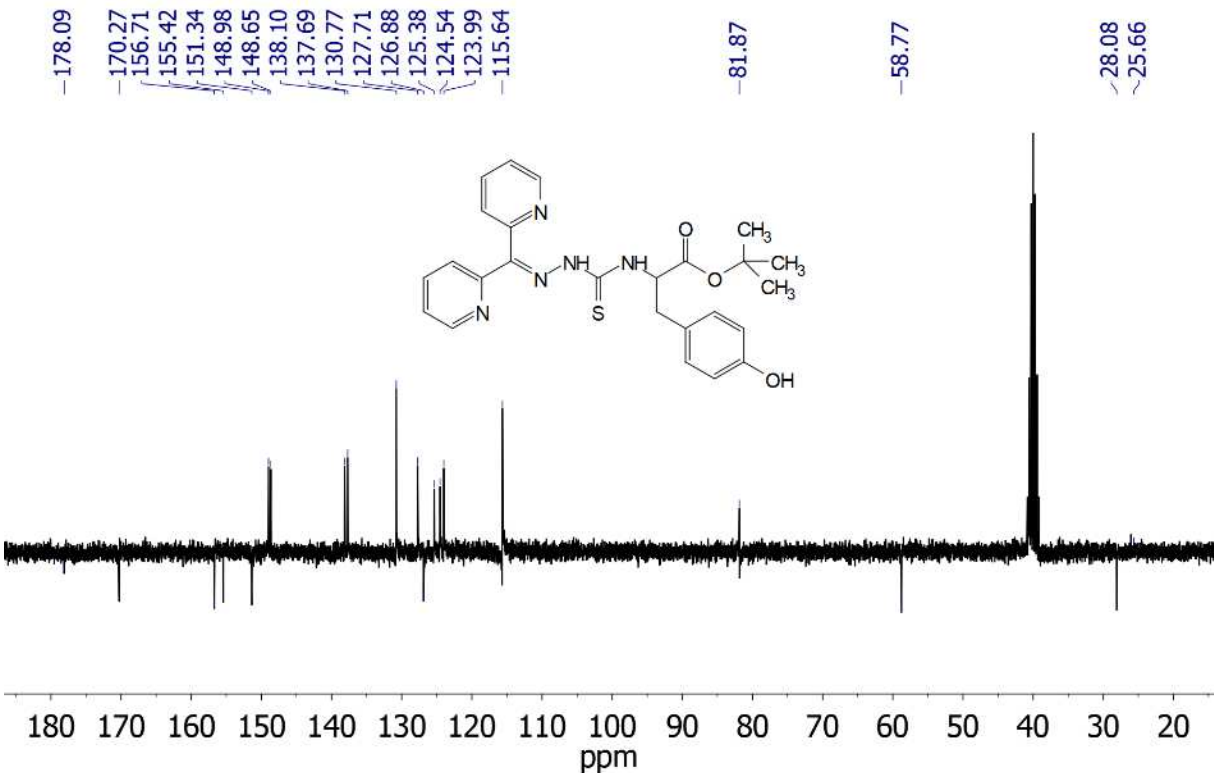

Figure S113. ^{13}C DEPTQ NMR spectrum of *tert*-butyl-2-{2-[bis(pyridin-2-yl)methylidene]hydrazine-carbamthioyl}-3-(4-hydroxyphenyl)-propanoate in DMSO-d_6 at 300 MHz.

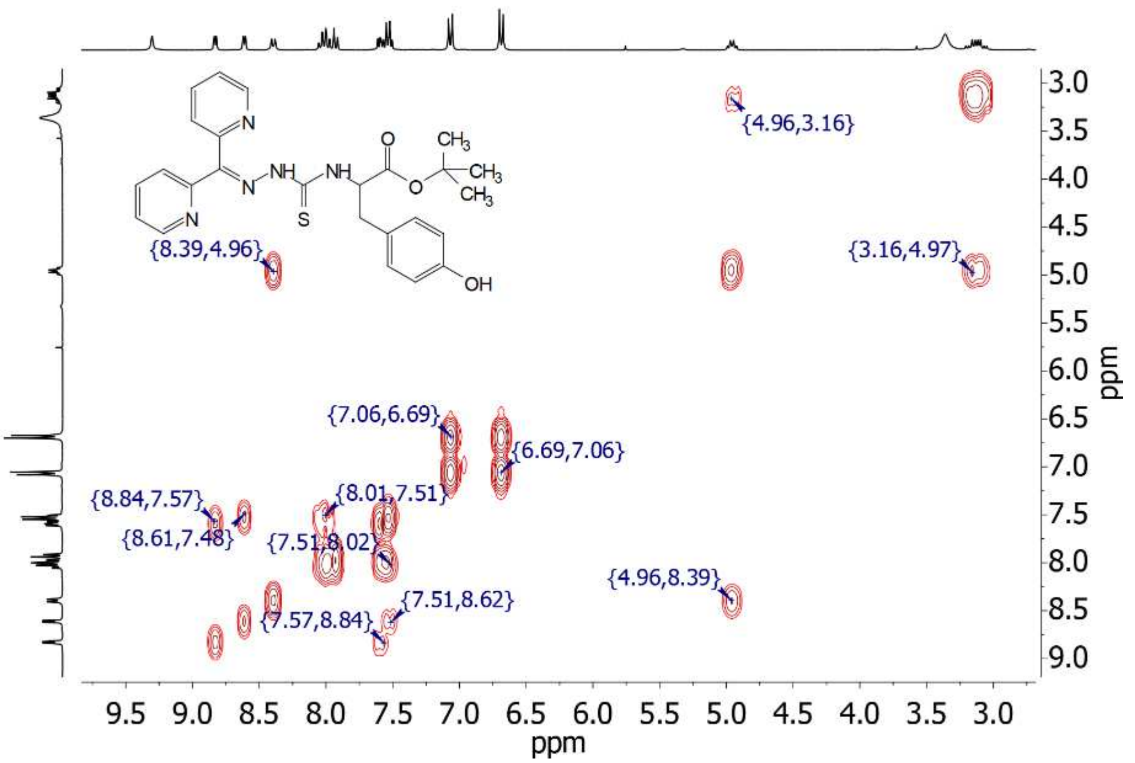

Figure S114. $^{1}H,^{1}H$ COSY NMR spectrum of *tert*-butyl-2-{2-[bis(pyridin-2-yl)methylidene]hydrazine-carbamthioyl}-3-(4-hydroxyphenyl)-propanoate in DMSO-d_6 at 300 MHz.

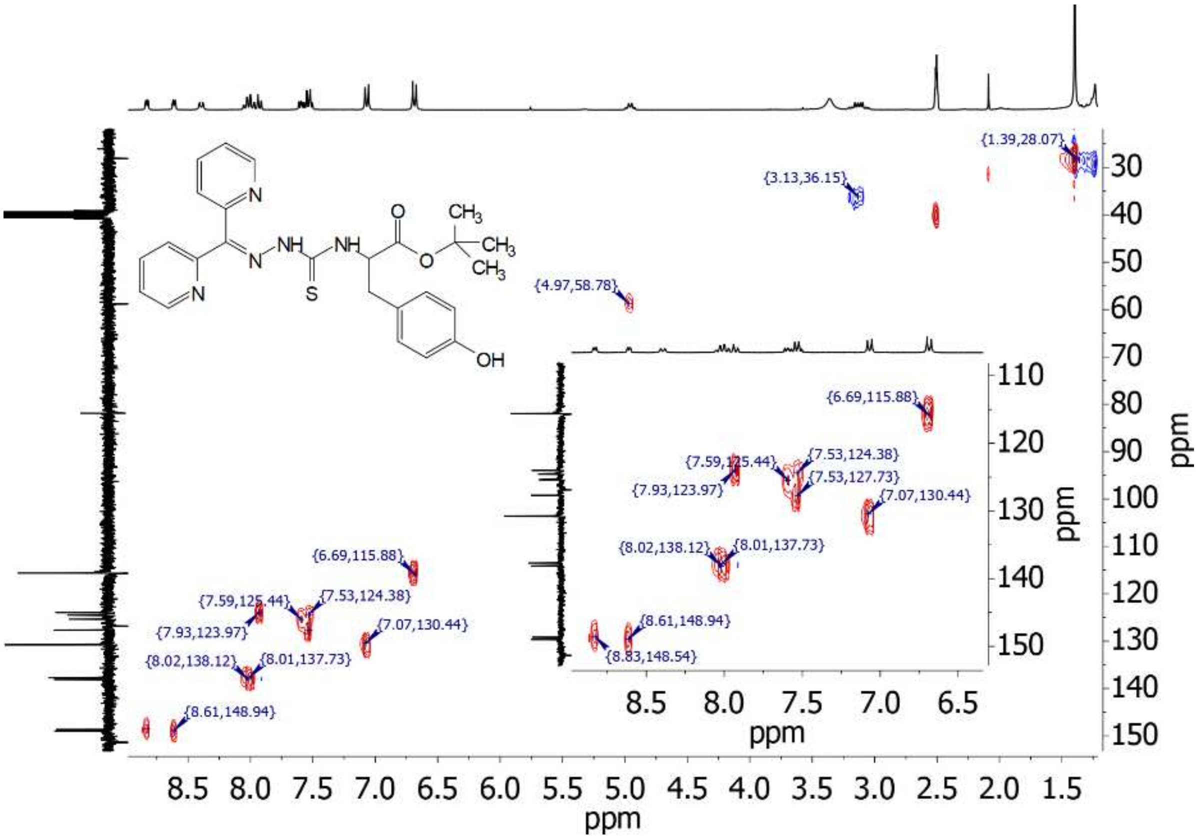

Figure S115. $^{1}H,^{13}C$ HMQC/HSQC NMR spectrum of t*ert*-butyl-2-{2-[bis(pyridin-2-yl)methylidene]-hydrazinecarbamthioyl}-3-(4-hydroxyphenyl)-propanoate in DMSO-d_6 at 300 MHz.

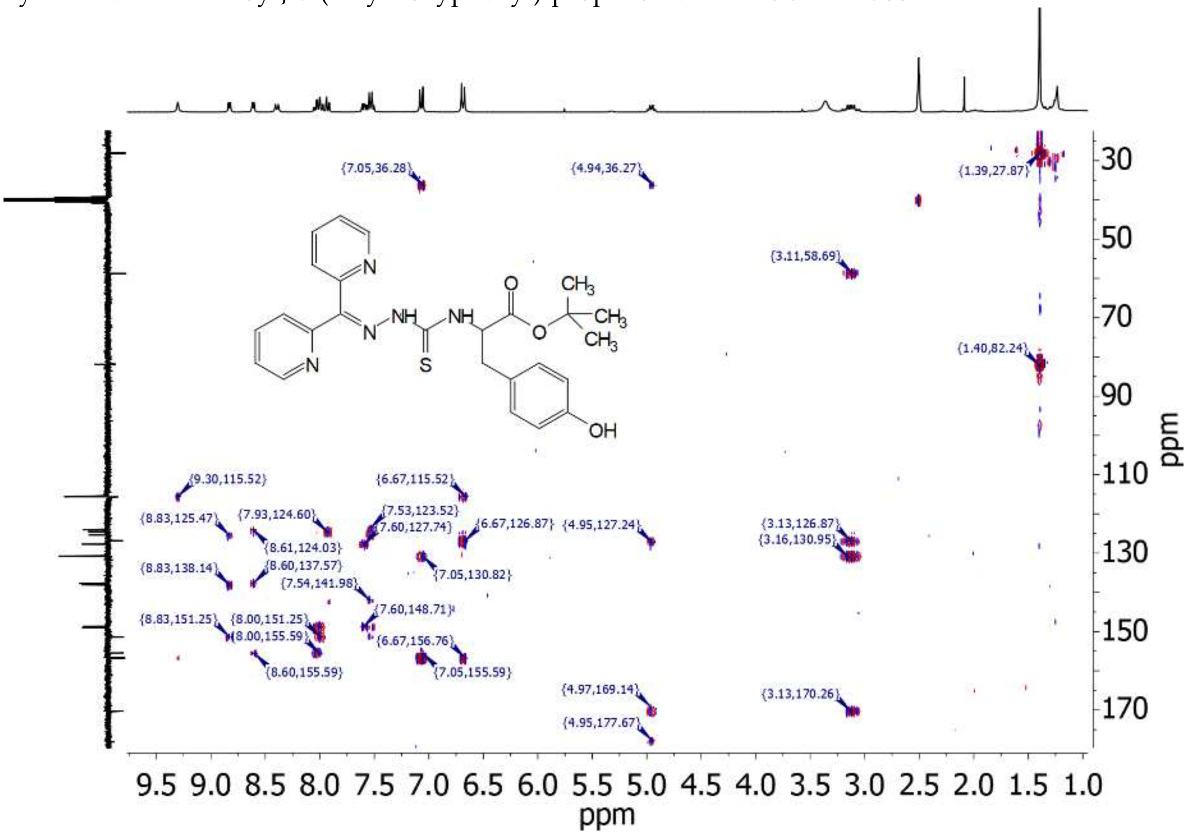

Figure S116. $^{1}H,^{13}C$ HMBC NMR spectrum of t*ert*-butyl-2-{2-[bis(pyridin-2-yl)methylidene]hydrazine-carbamthioyl}-3-(4-hydroxyphenyl)-propanoate in DMSO-d_6 at 300 MHz.

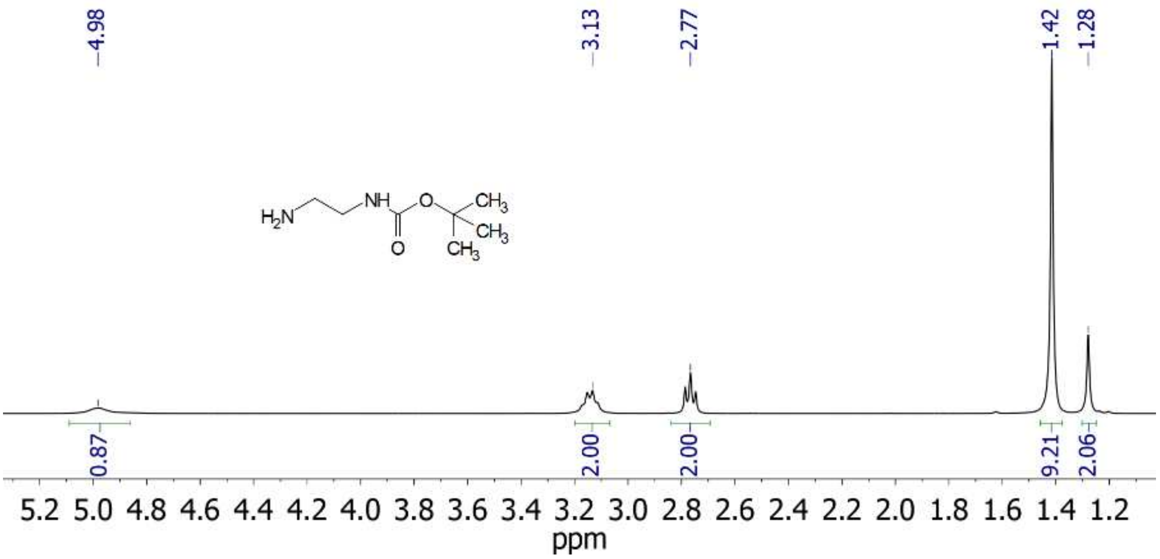

Figure S117. ^{1}H NMR spectrum of *tert*-butyl *N*-(2-aminoethyl)carbamate in $CDCl_3$ at 300 MHz.

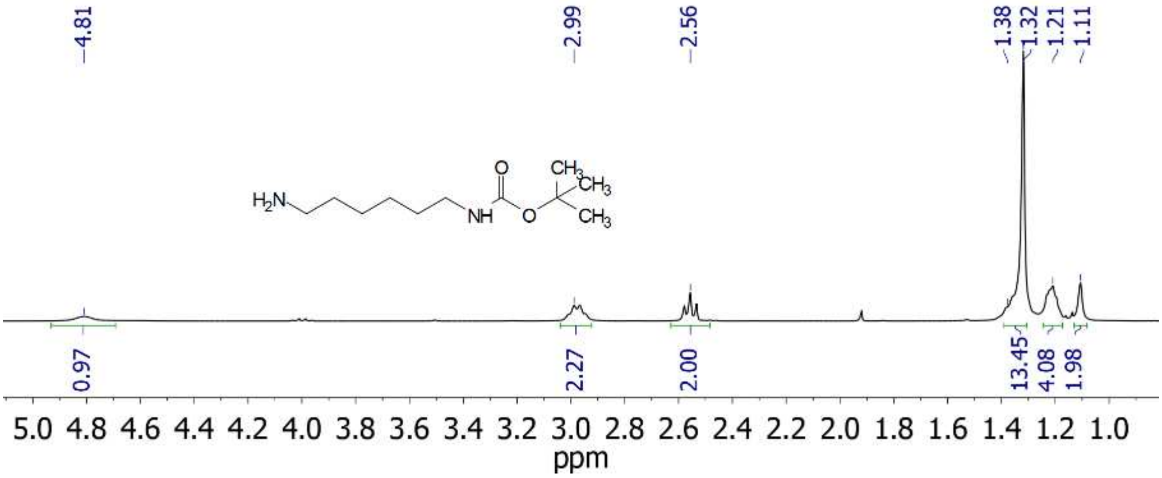

Figure S118. ^{1}H NMR spectrum of t*ert*-butyl *N*-(6-aminohexyl)carbamate in $CDCl_3$ at 300 MHz.

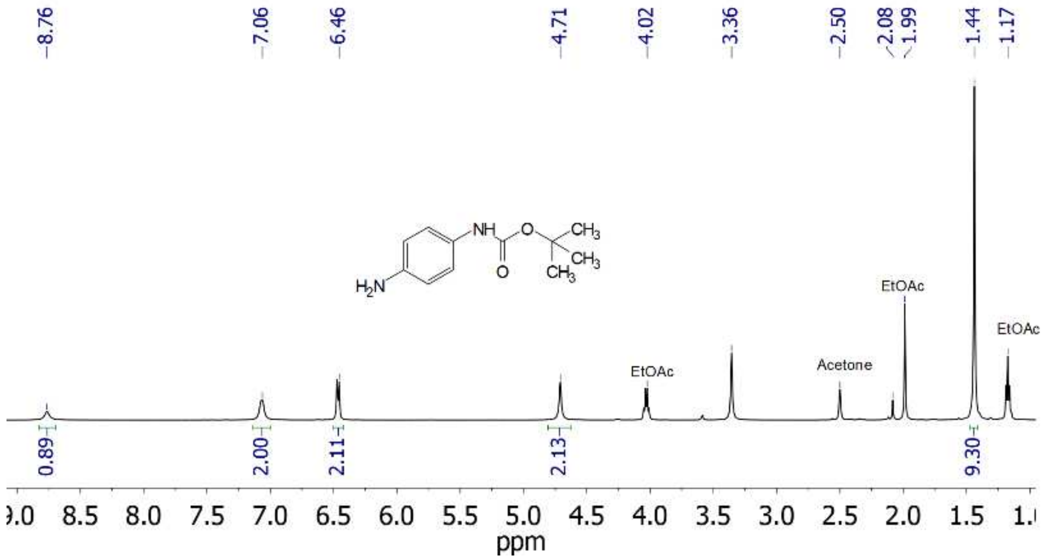

Figure S119. ^{1}H NMR spectrum of *tert*-butyl (4-aminophenyl)carbamate in DMSO-d_6 at 499 MHz.

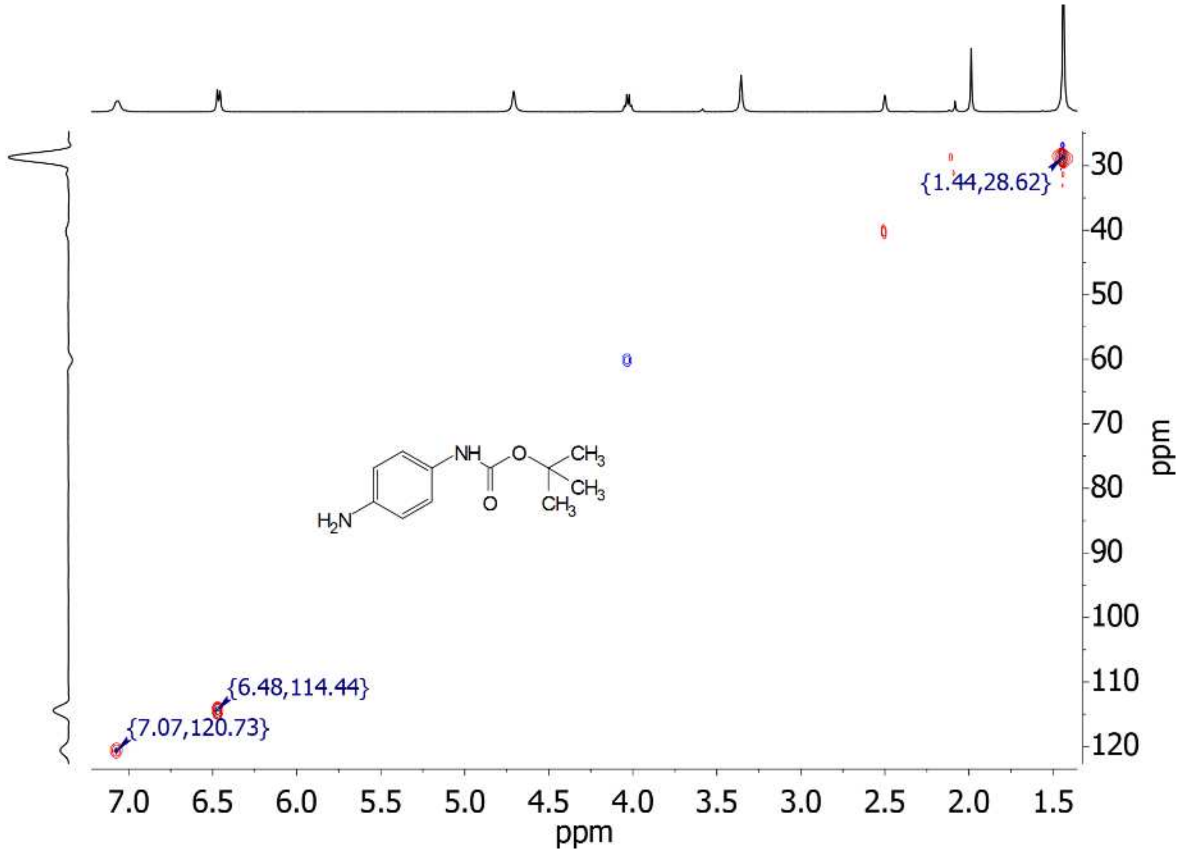

Figure S120. ^{1}H,^{13}C HMQC/HSQC NMR spectrum of *tert*-butyl (4-aminophenyl)carbamate in DMSO-d_6 at 499 MHz.

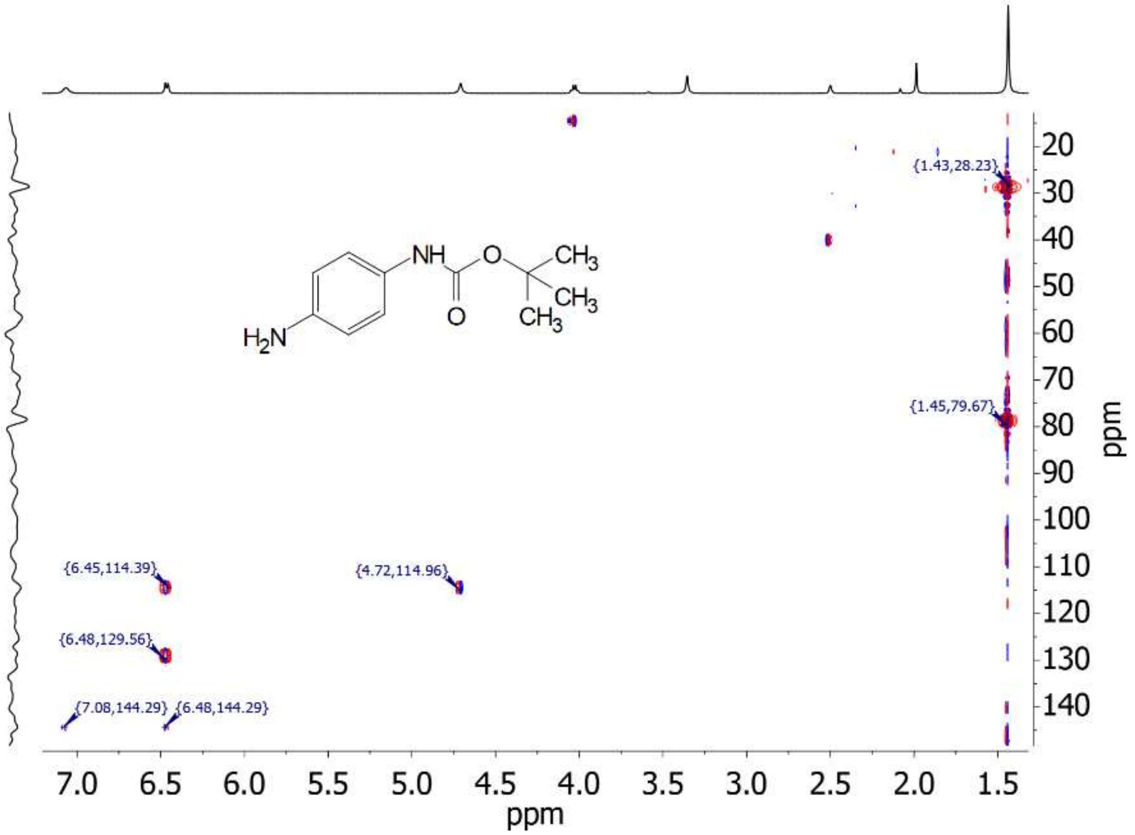

Figure S121. ^{1}H,^{13}C HMBC NMR spectrum of *tert*-butyl (4-aminophenyl)carbamate in DMSO-d_6 at 499 MHz.

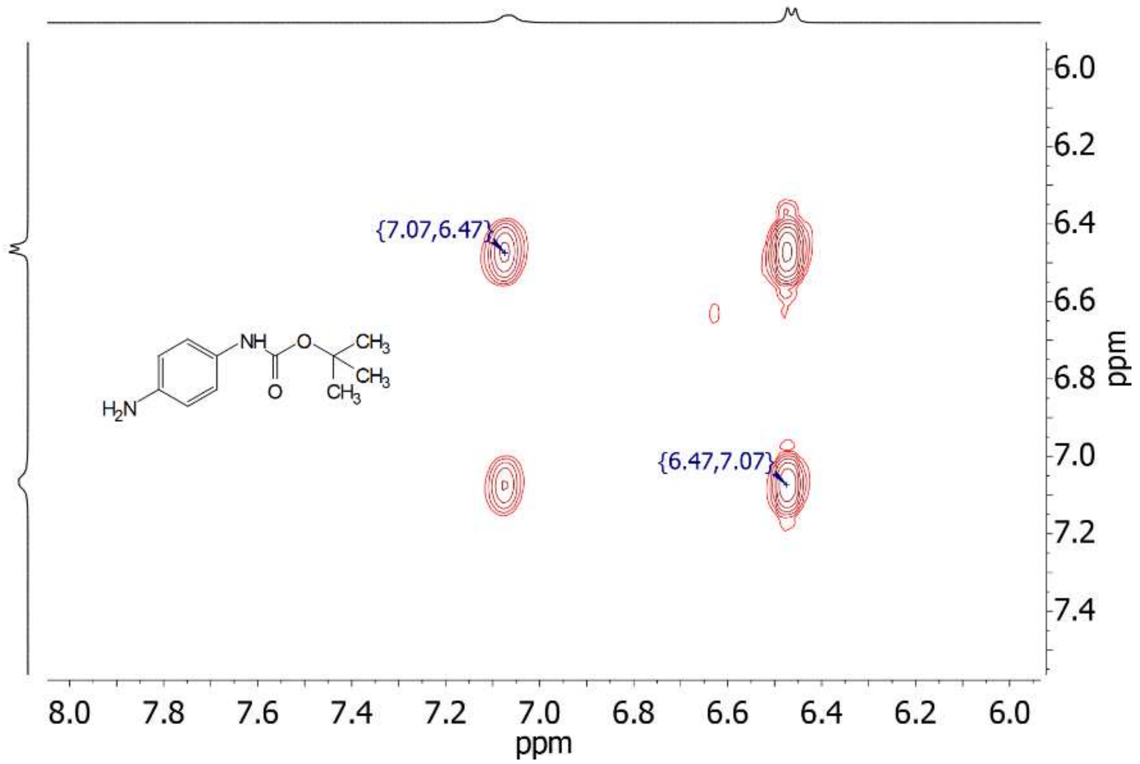

Figure S122. $^{1}H,^{1}H$ COSY NMR spectrum of *tert*-butyl (4-aminophenyl)carbamate in DMSO-d_6 at 499 MHz.

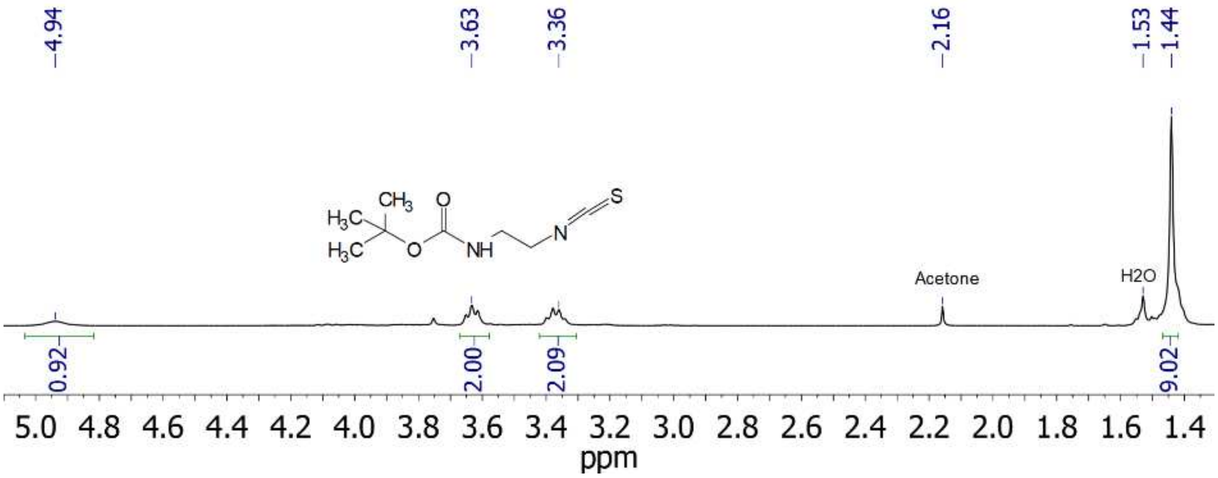

Figure S123. ^{1}H NMR spectrum of *ter*t-butyl(2-isothiocyanatoethyl)carbamate in $CDCl_3$ at 300 MHz.

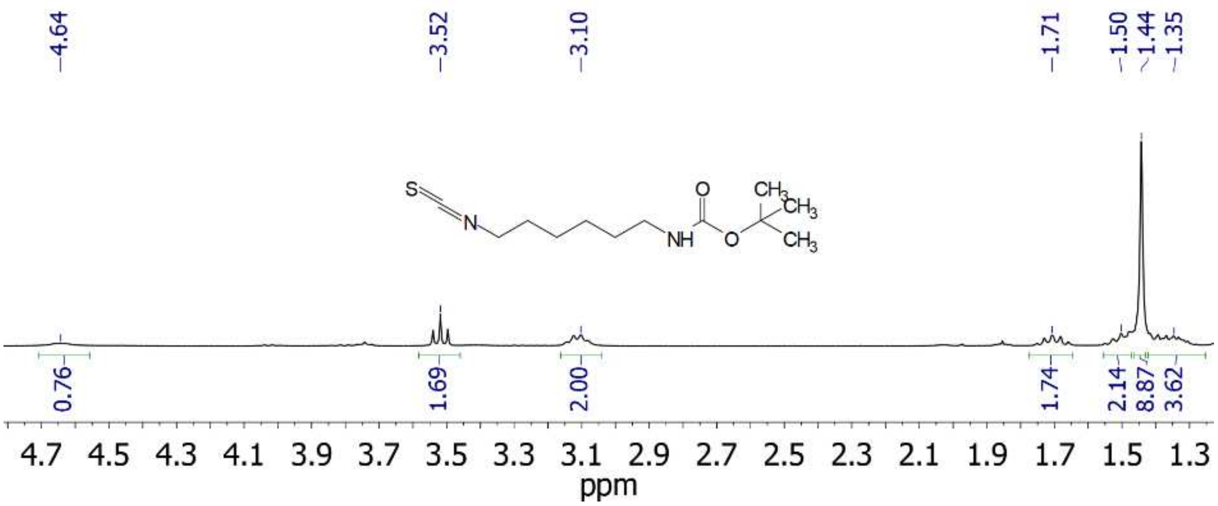

Figure S124. ^{1}H NMR spectrum of *tert*-butyl(6-isothiocyanatohexyl)carbamate in $CDCl_3$ at 300 MHz.

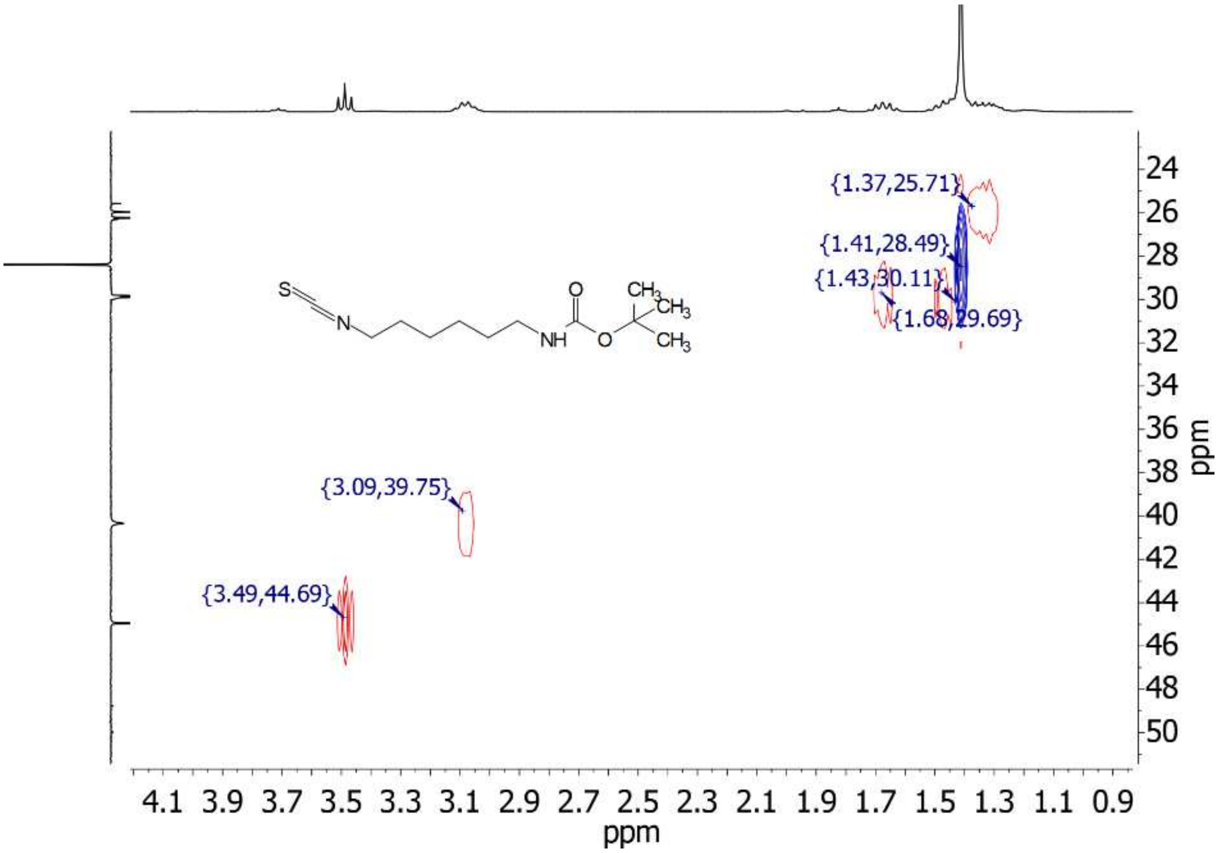

Figure S125. $^{1}H,^{13}C$ HSQC NMR spectrum of *tert*-butyl(6-isothiocyanatohexyl)carbamate in $CDCl_3$ at 300 MHz.

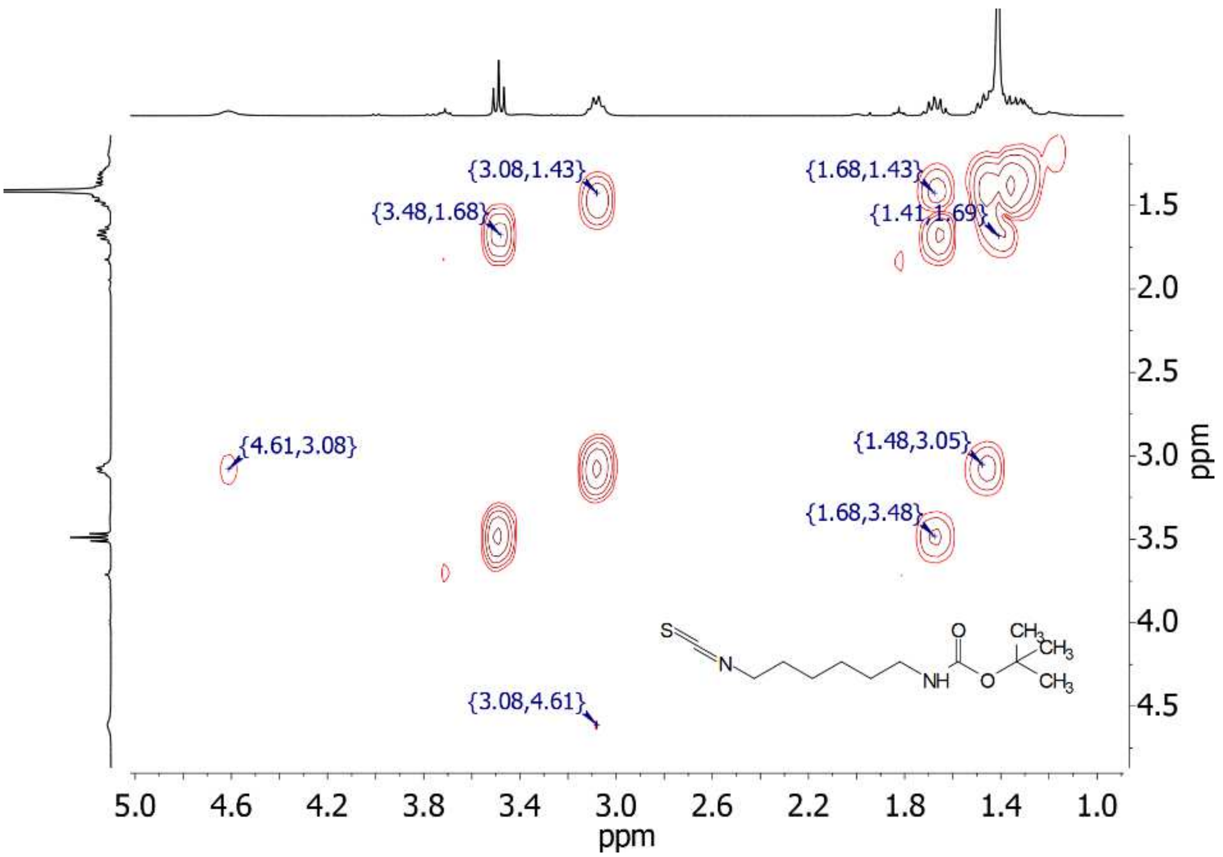

Figure S126. $^1H,^1H$ COSY NMR spectrum of *tert*-butyl(6-isothiocyanatohexyl)carbamate in $CDCl_3$ at 300 MHz.

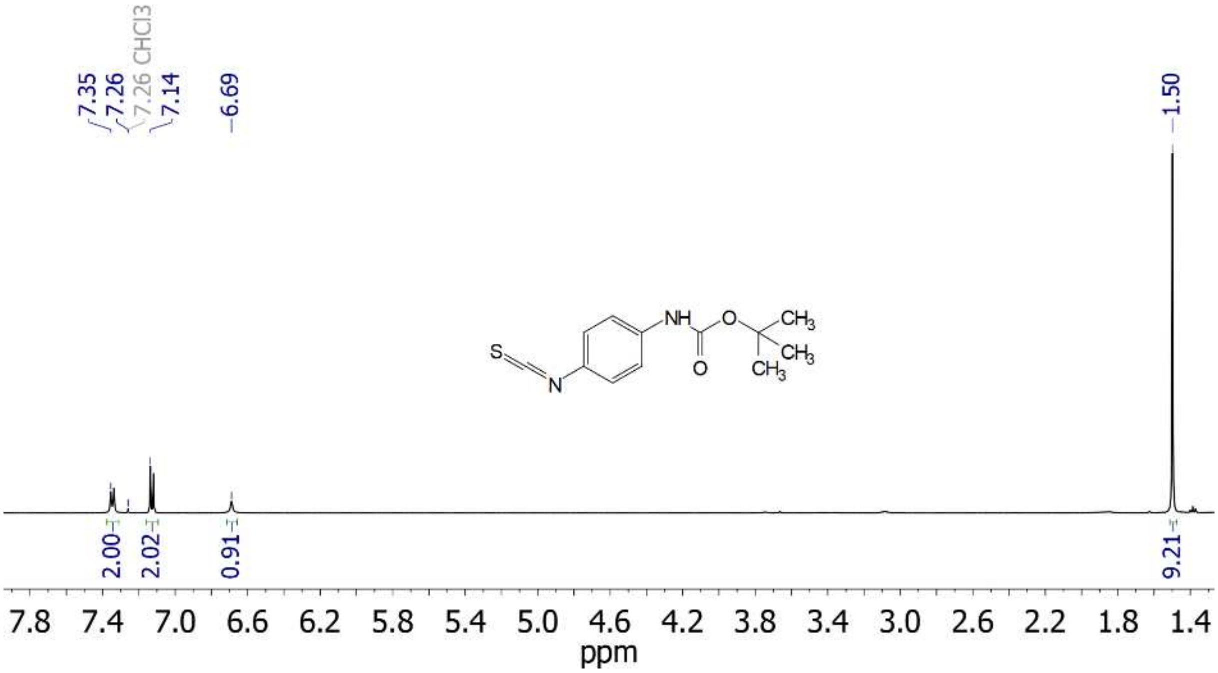

Figure S127. ^1H NMR spectrum of *tert*-butyl(4-isothiocyanatophenyl)carbamate in $CDCl_3$ at 499 MHz.

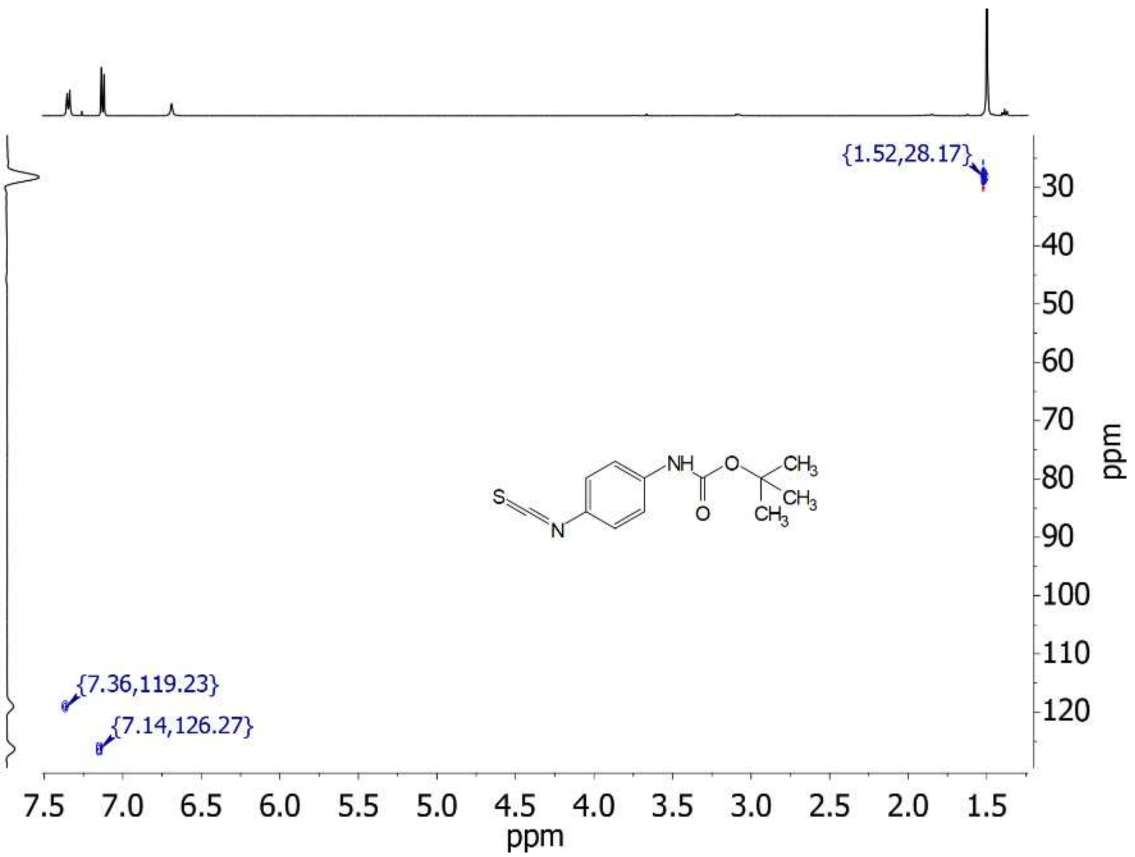

Figure S128. ^{1}H,^{13}C HMQC/HSQC NMR spectrum of *tert*-butyl(4-isothiocyanatophenyl)carbamate in $CDCl_3$ at 499 MHz.

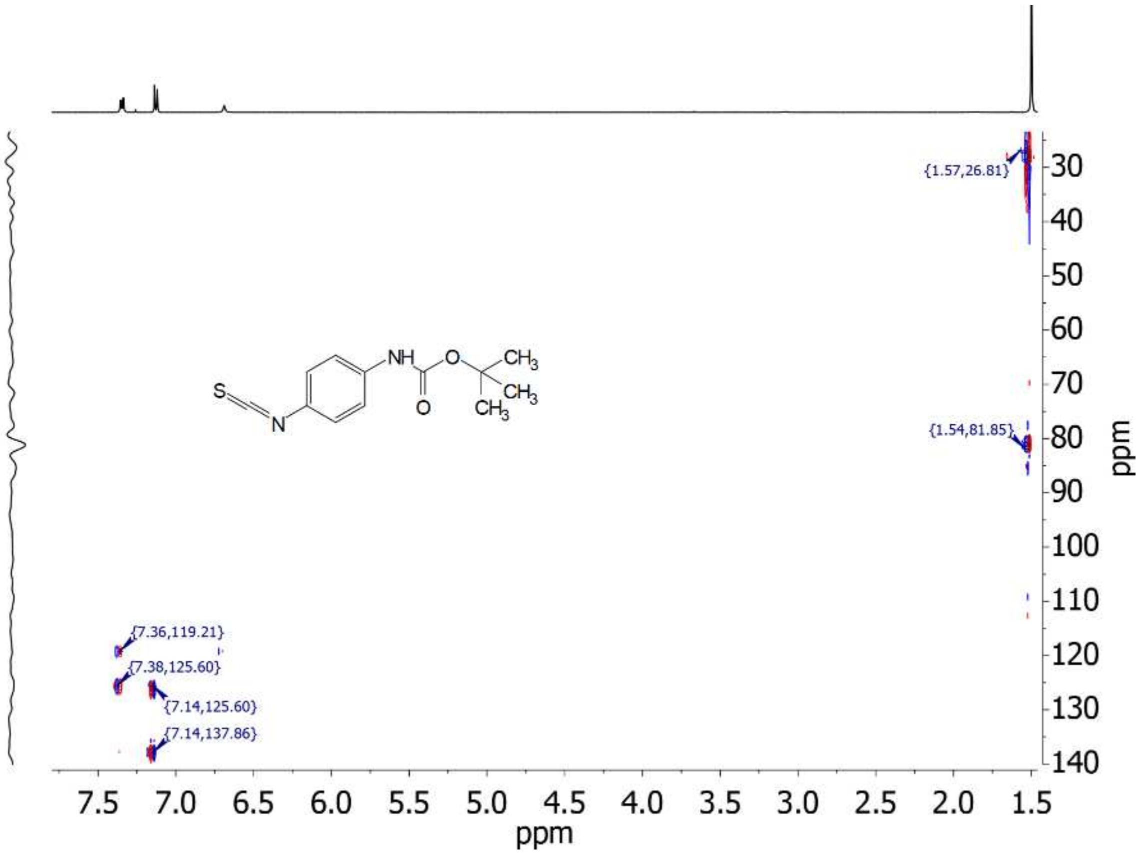

Figure S129. ^{1}H,^{13}C HMBC NMR spectrum of *tert*-butyl(4-isothiocyanatophenyl)carbamate in $CDCl_3$ at 499 MHz.

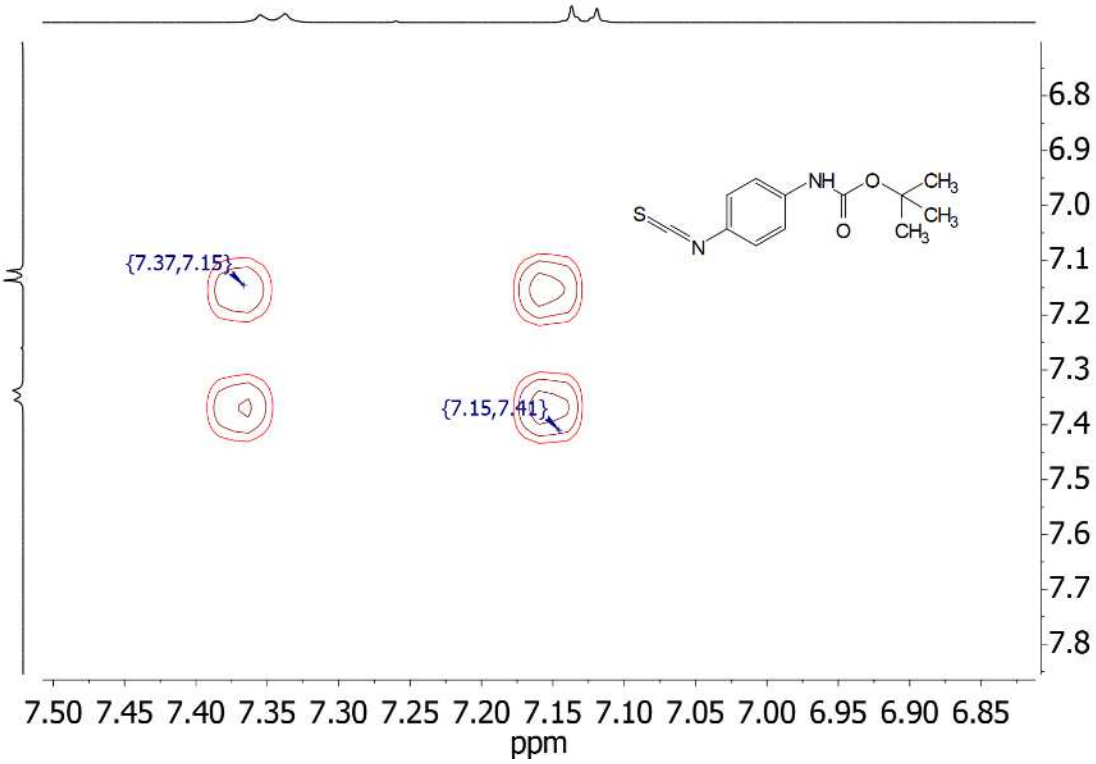

Figure S130. $^1H,^1H$ COSY NMR spectrum of *tert*-butyl(4-isothiocyanatophenyl)carbamate in $CDCl_3$ at 499 MHz.

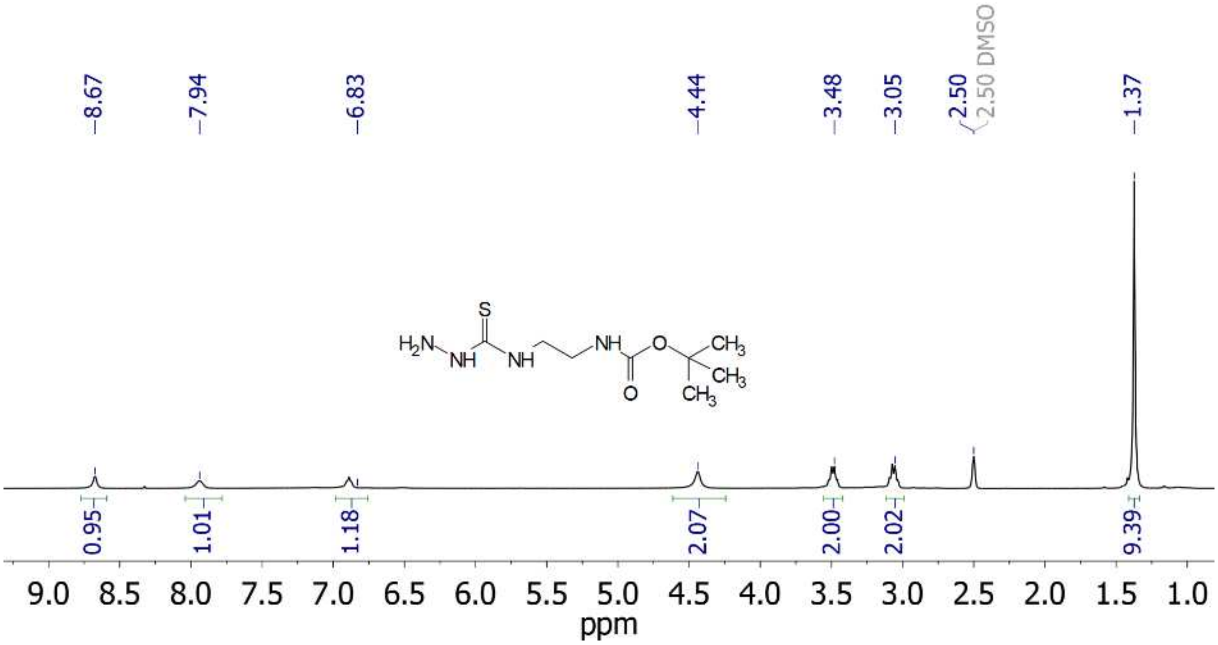

Figure S131. ^1H NMR spectrum of 4-(2-*tert*-butoxycarbonylamino-ethyl)thiosemicarbazide in DMSO-d_6 at 300 MHz.

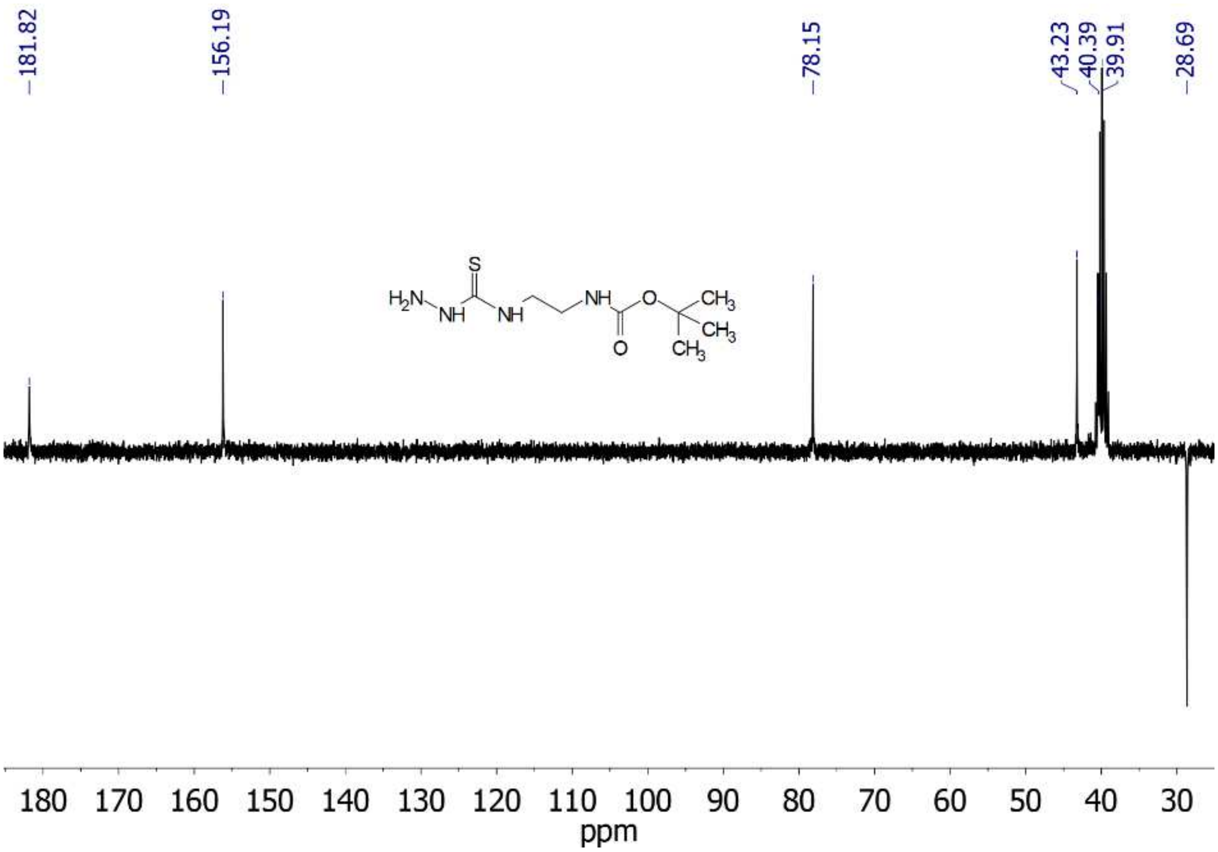

Figure S132. ^{13}C DEPTQ NMR spectrum of 4-(2-*tert*-butoxycarbonylamino-ethyl)thiosemicarbazide in DMSO-d_6 at 300 MHz.

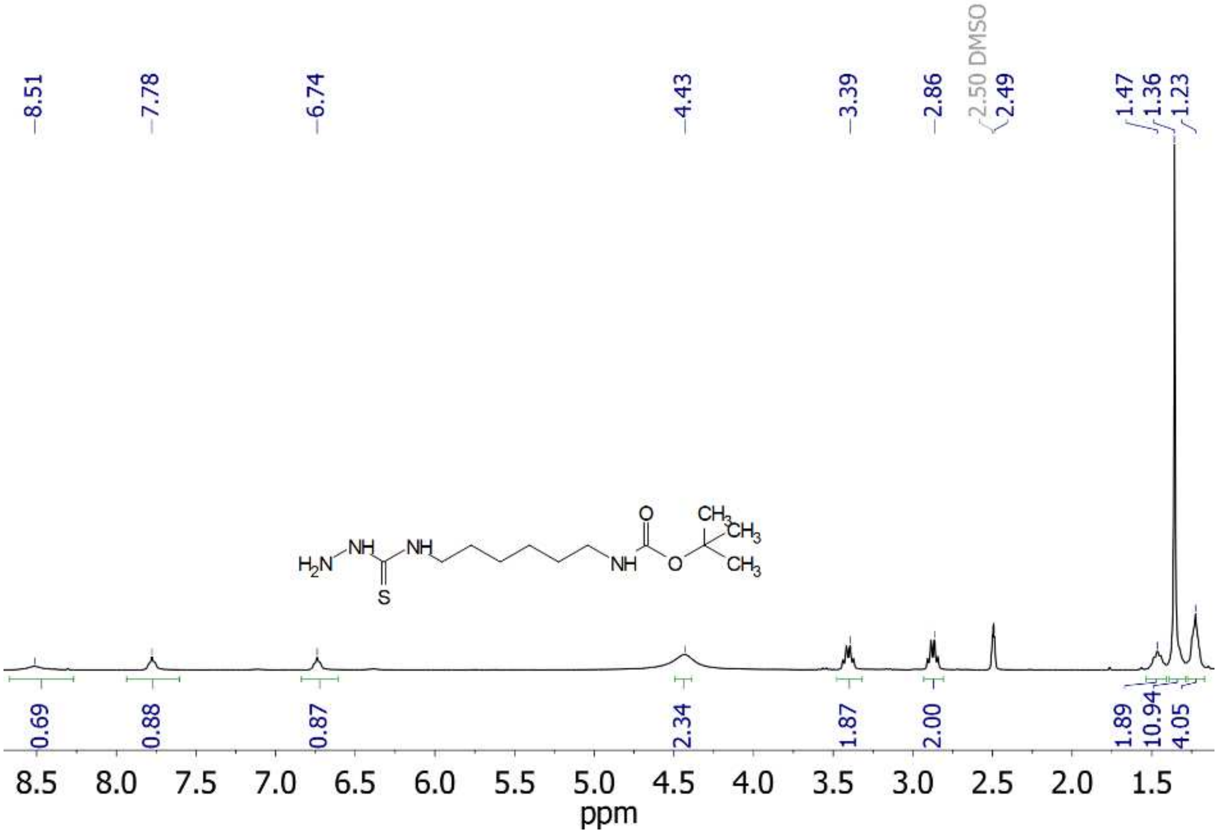

Figure S133. ^{1}H NMR spectrum of 4-(6-*tert*-butoxycarbonylamino-hexyl)thiosemicarbazide in DMSO-d_6 at 300 MHz.

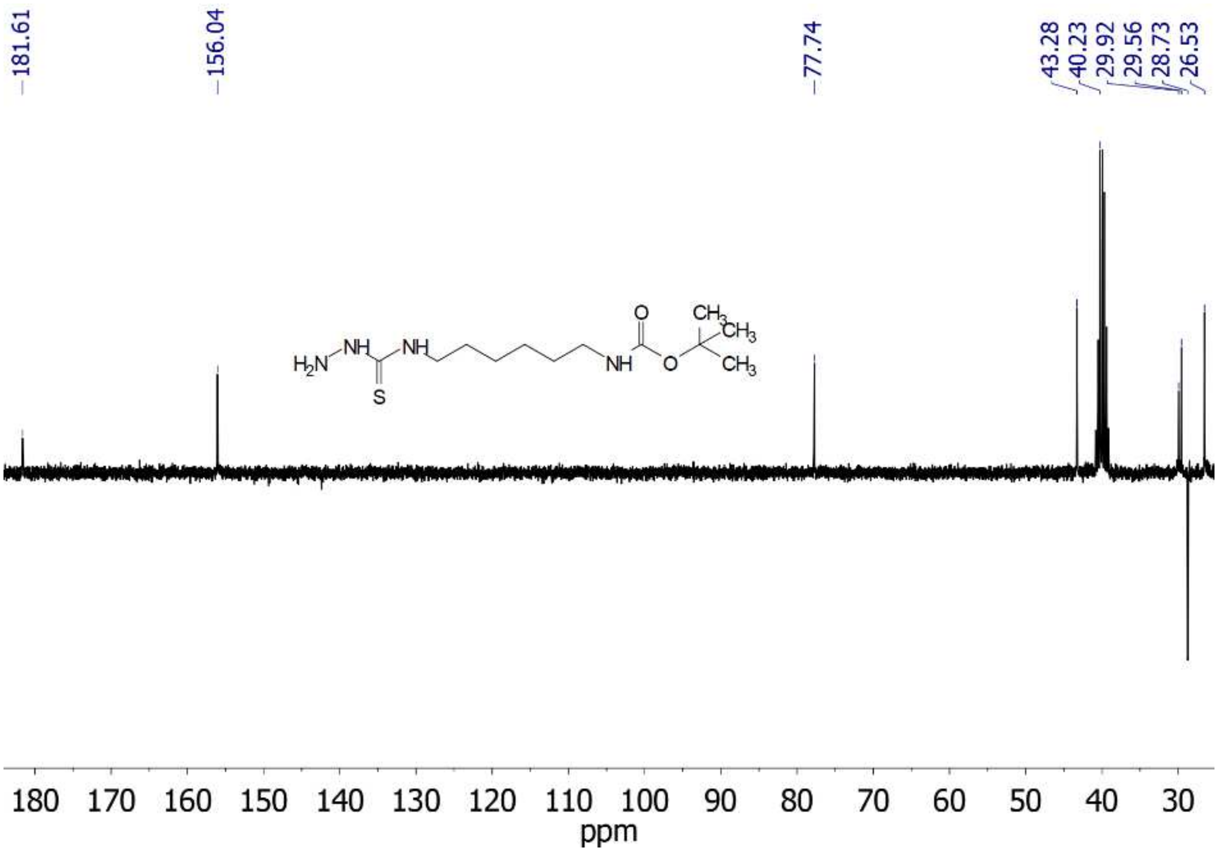

Figure S134. ^{13}C DEPTQ NMR spectrum of 4-(6-*tert*-butoxycarbonylamino-hexyl)thiosemicarbazide in DMSO-d_6 at 300 MHz.

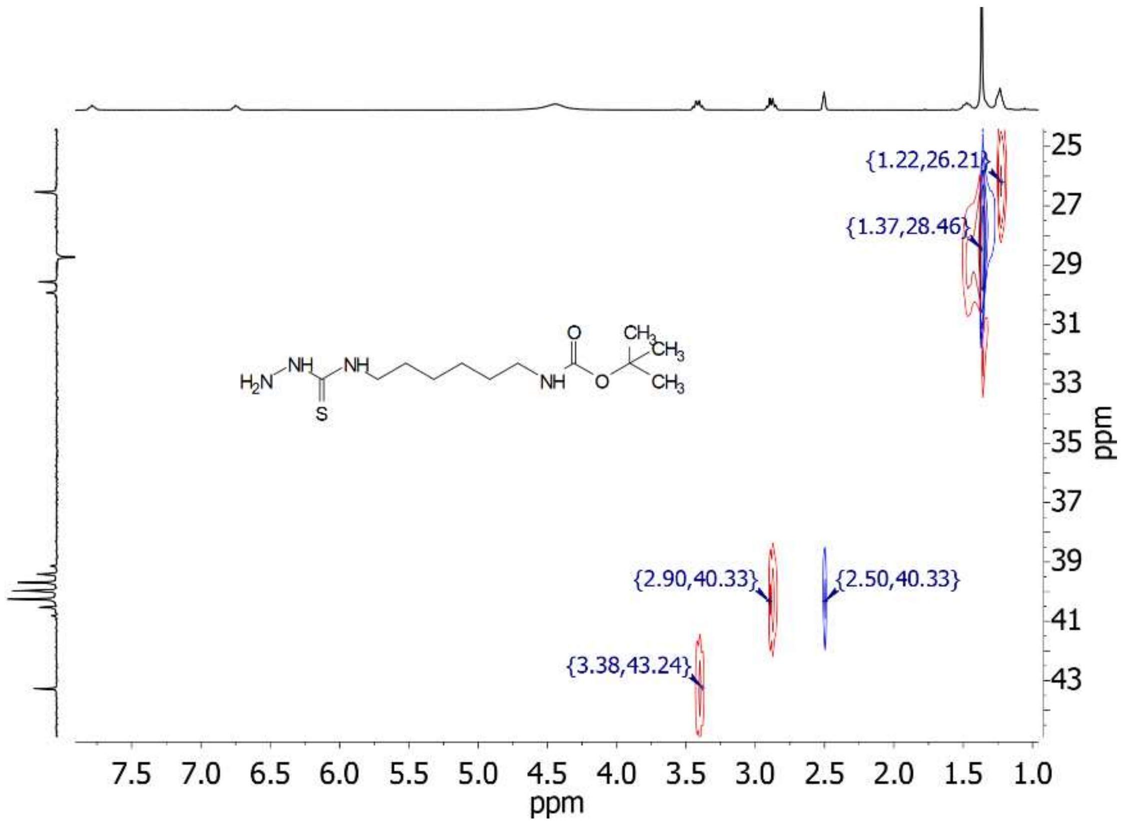

Figure S135. $^{1}H,^{13}C$ HSQC NMR spectrum of 4-(6-*tert*-butoxycarbonylamino-hexyl)thiosemicarbazide in DMSO-d_6 at 300 MHz.

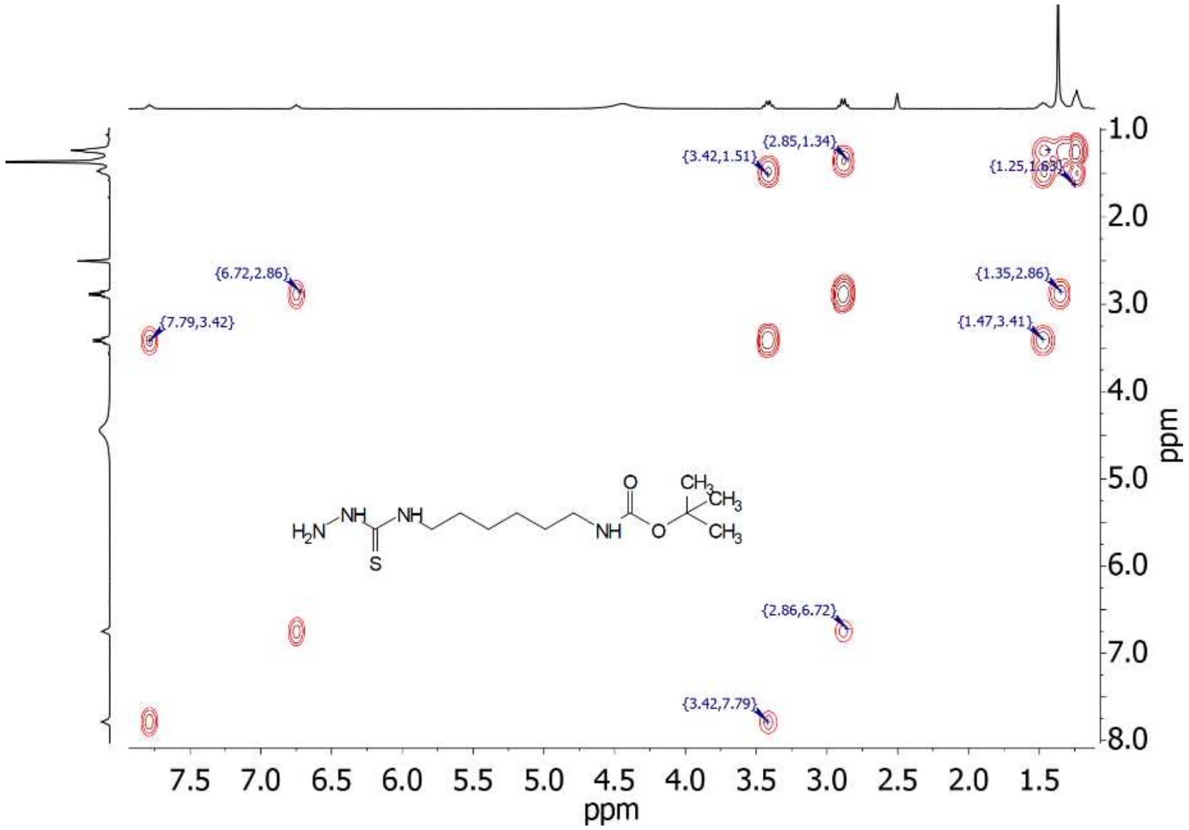

Figure S136. ^{1}H,^{1}H COSY NMR spectrum of 4-(6-*tert*-butoxycarbonylamino-hexyl)thiosemicarbazide in DMSO-d_6 at 300 MHz.

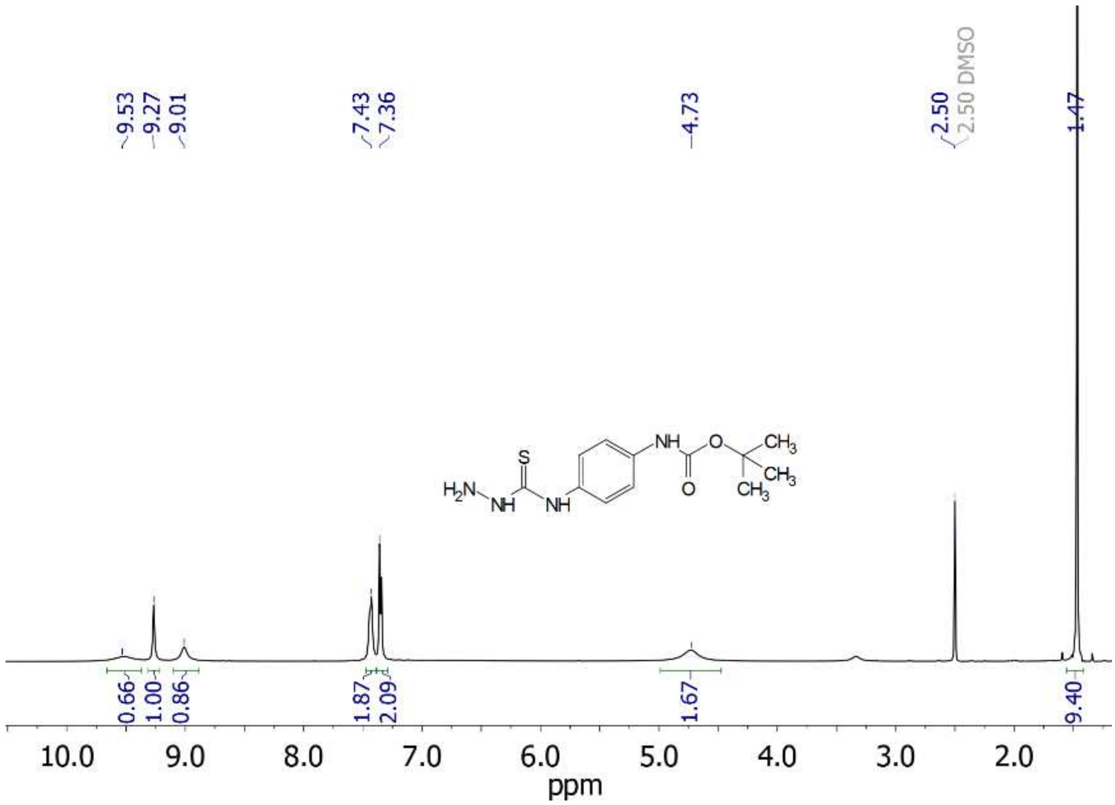

Figure S137. ^{1}H NMR spectrum of 4-(4-*tert*-butoxycarbonylamino-phenyl)thiosemicarbazide in DMSO-d_6 at 499 MHz.

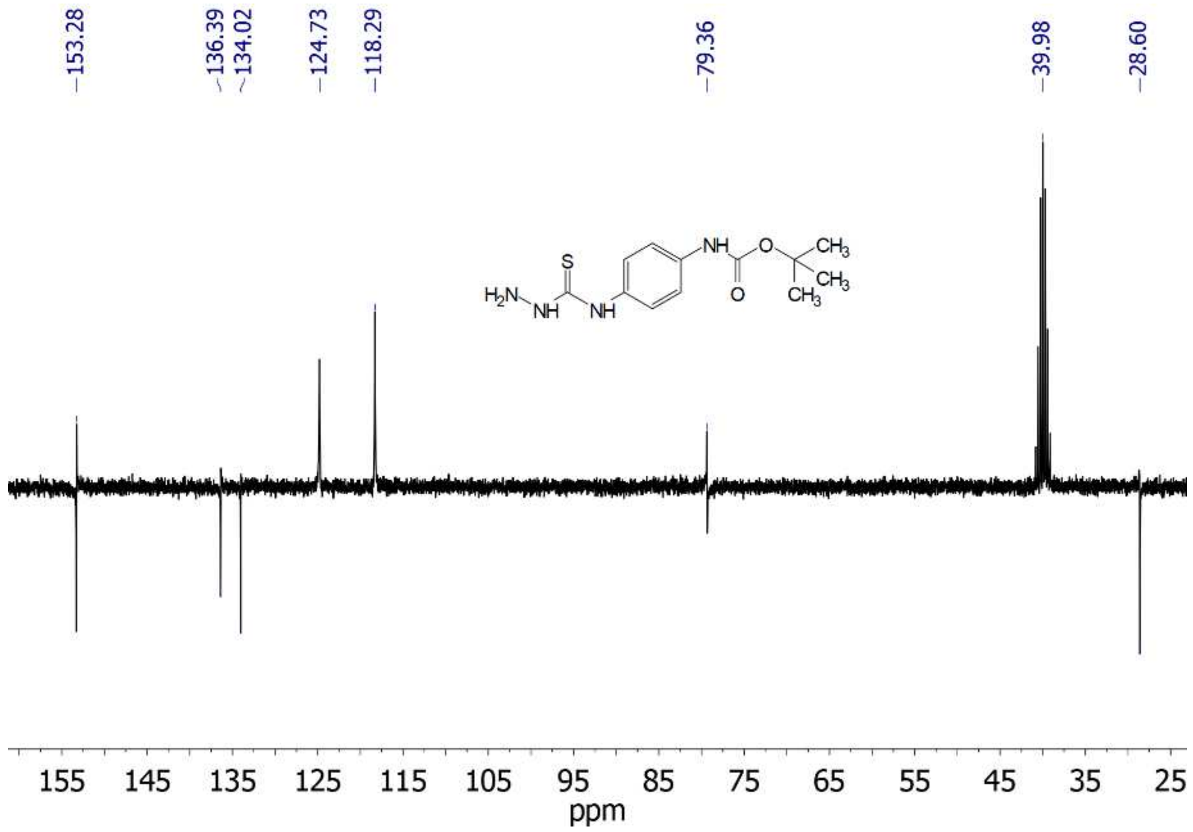

Figure S138. ^{13}C DEPTQ NMR spectrum of 4-(4-*tert*-butoxycarbonylamino-phenyl)thiosemicarbazide in DMSO-d_6 at 300 MHz.

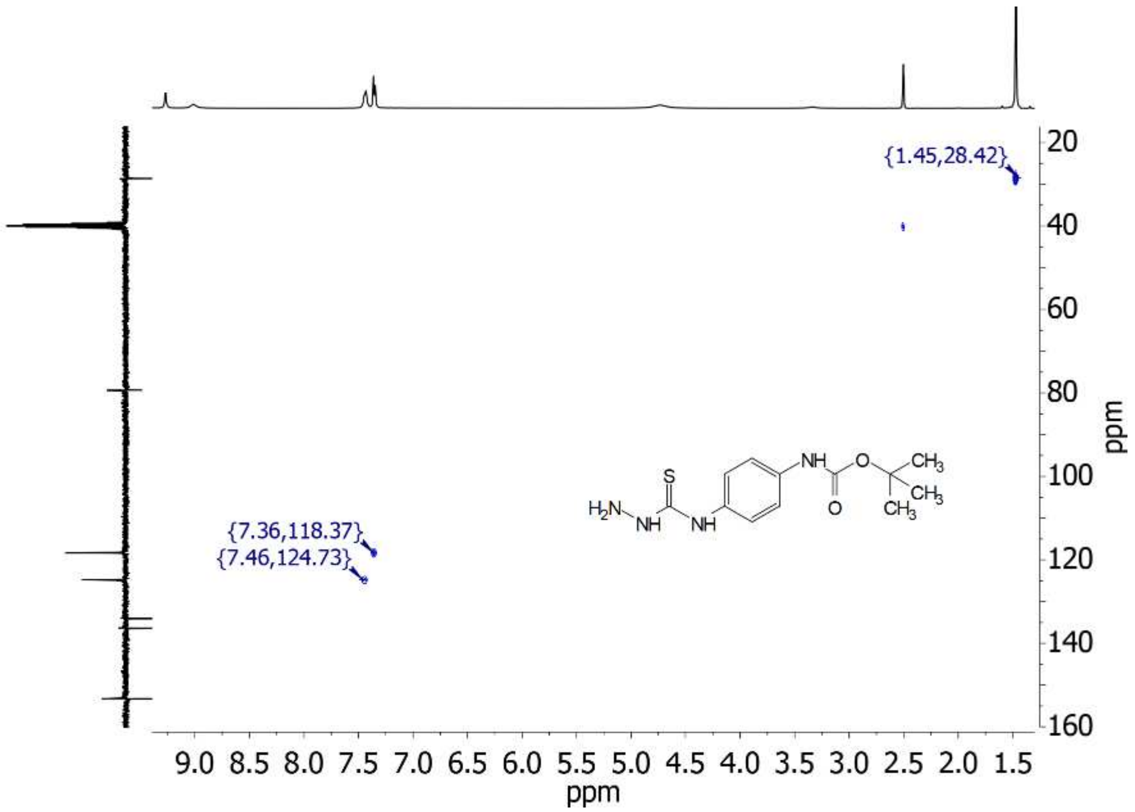

Figure S139. ^{1}H,^{13}C HMQC/HSQC NMR spectrum of 4-(4-*tert*-butoxycarbonylamino-phenyl) thiosemicarbazide in DMSO-d_6 at 499 MHz.

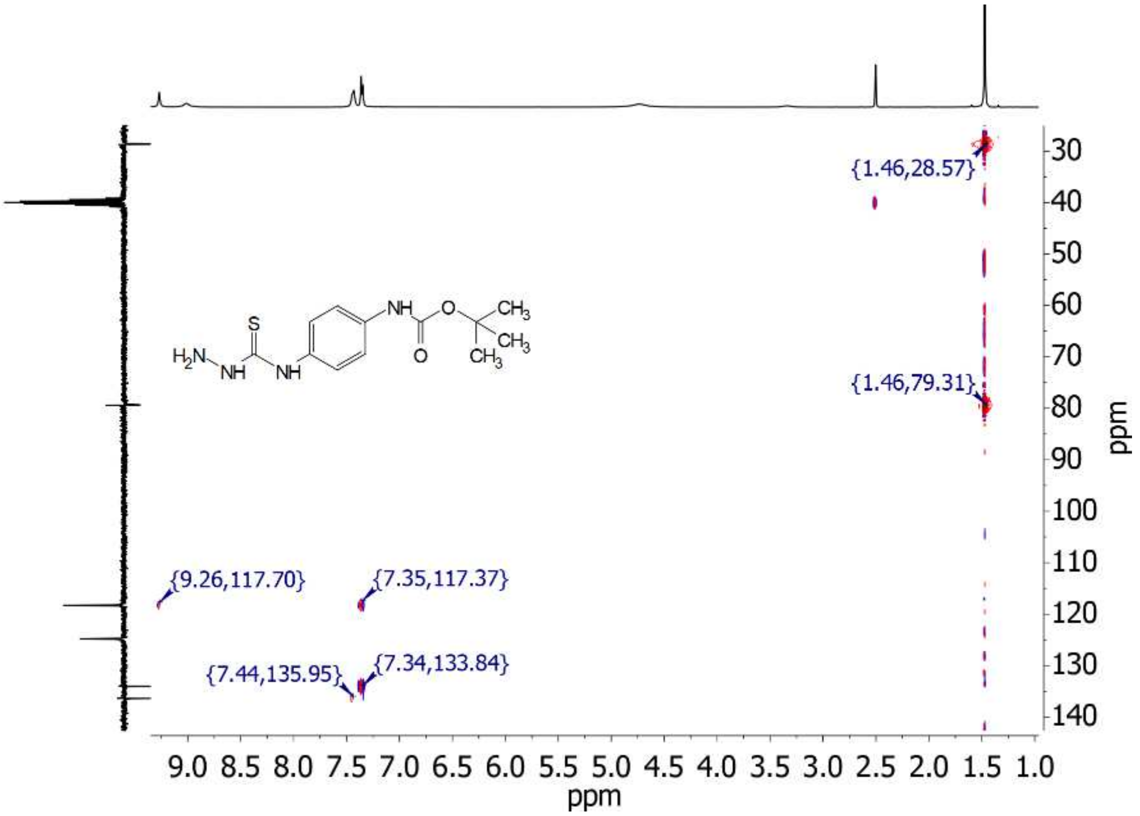

Figure S140. $^1H,^{13}C$ HMBC NMR spectrum of 4-(4-*tert*-butoxycarbonylamino-phenyl)thiosemicarbazide in DMSO-d_6 at 499 MHz.

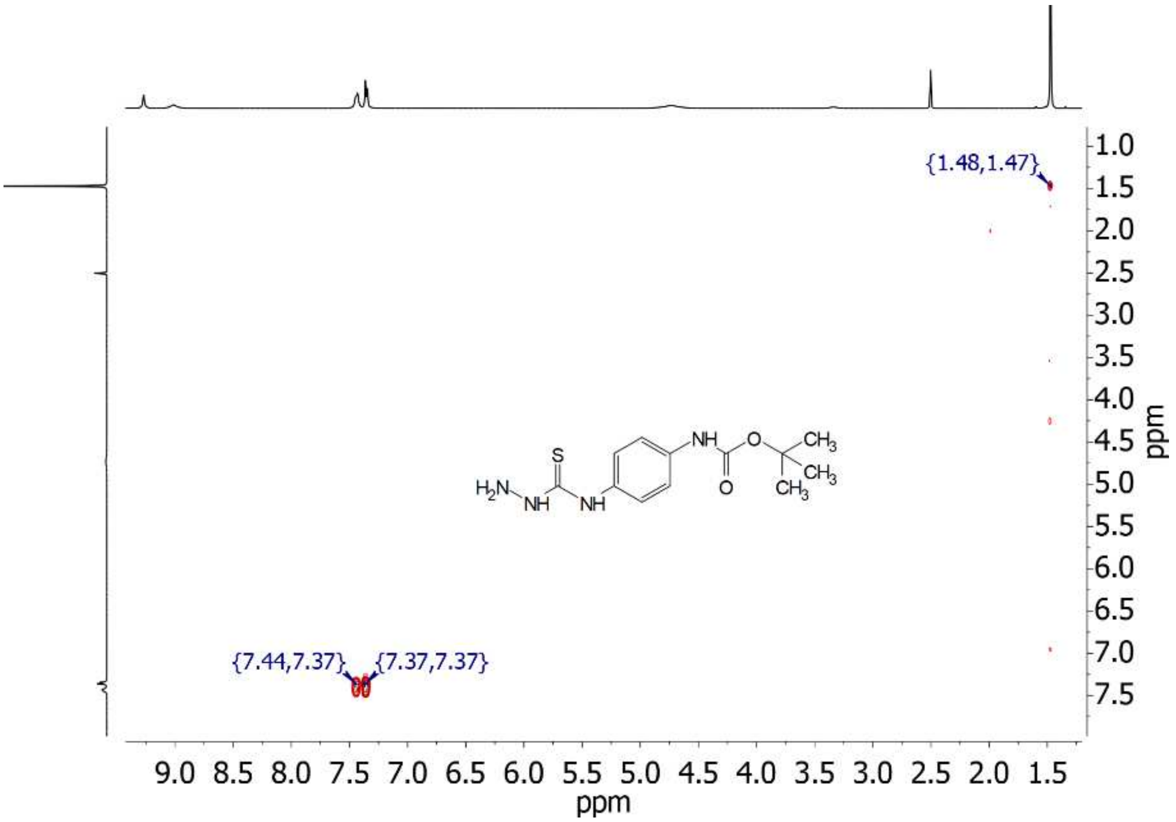

Figure S141. $^1H,^1H$ COSY NMR spectrum of 4-(4-*tert*-butoxycarbonylamino-phenyl)thiosemicarbazide in DMSO-d_6 at 499 MHz.

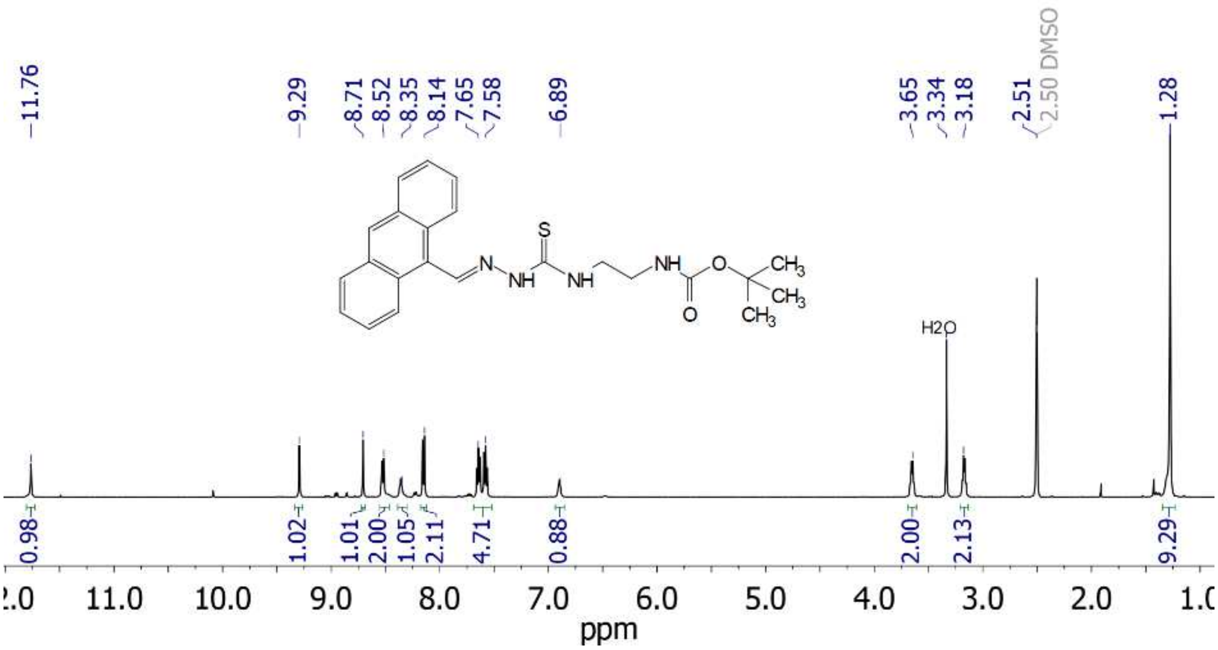

Figure S142. ^{1}H NMR spectrum of 9-anthraldehyde-4-(2-*tert*-butoxycarbonylamino-ethyl)-3-thiosemicarbazone in DMSO-d_6 at 499 MHz.

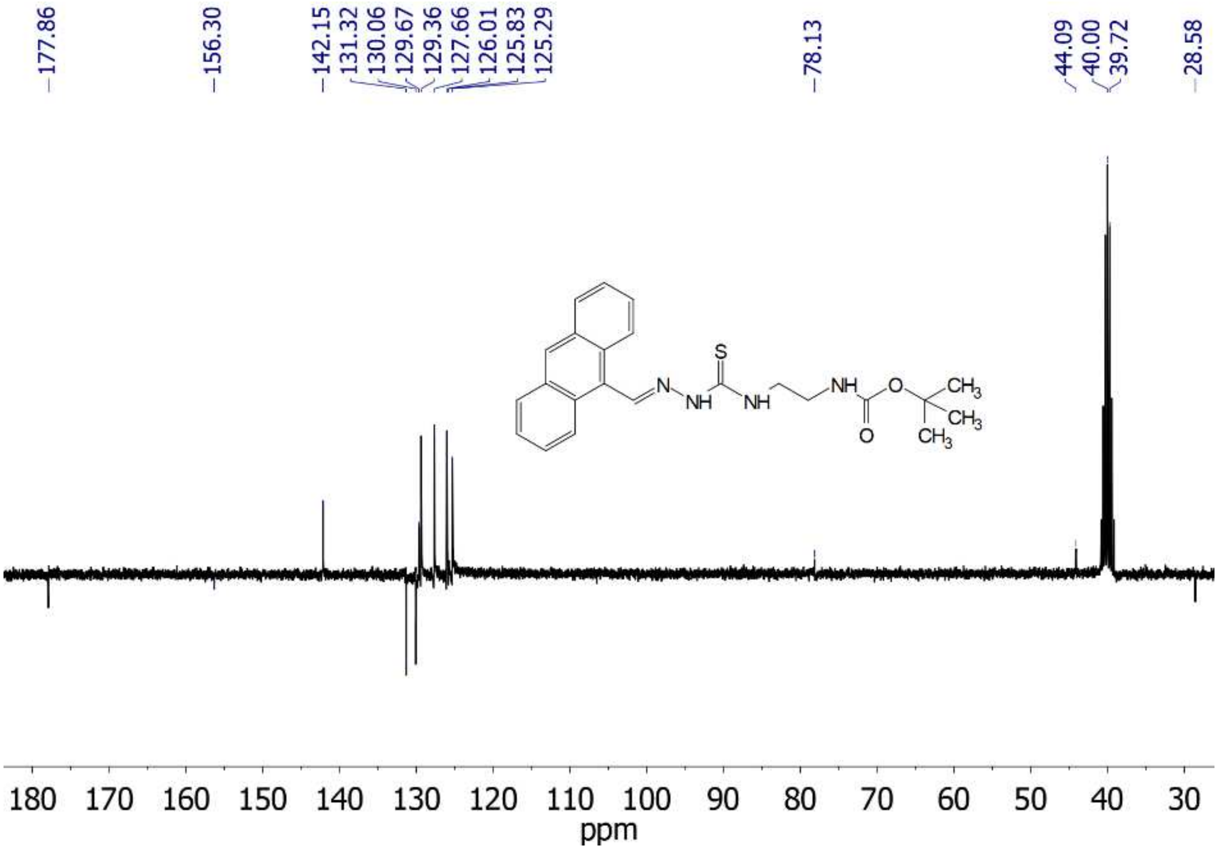

Figure S143. ^{13}C DEPTQ NMR spectrum of 9-anthraldehyde-4-(2-*tert*-butoxycarbonylamino-ethyl)-3-thiosemicarbazone in DMSO-d_6 at 300 MHz.

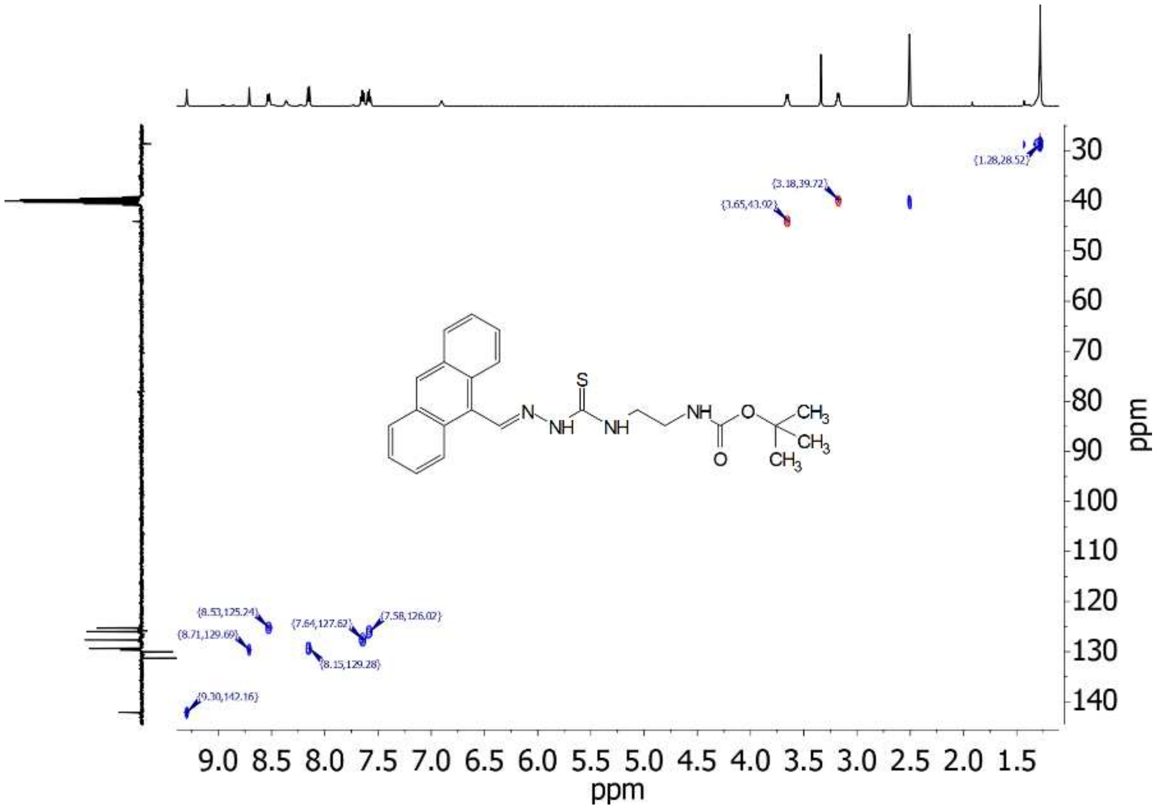

Figure S144. $^{1}H,^{13}C$ HMQC/HSQC NMR spectrum of 9-anthraldehyde-4-(2-*tert*-butoxycarbonylamino-ethyl)-3-thiosemicarbazone in DMSO-d_6 at 499 MHz.

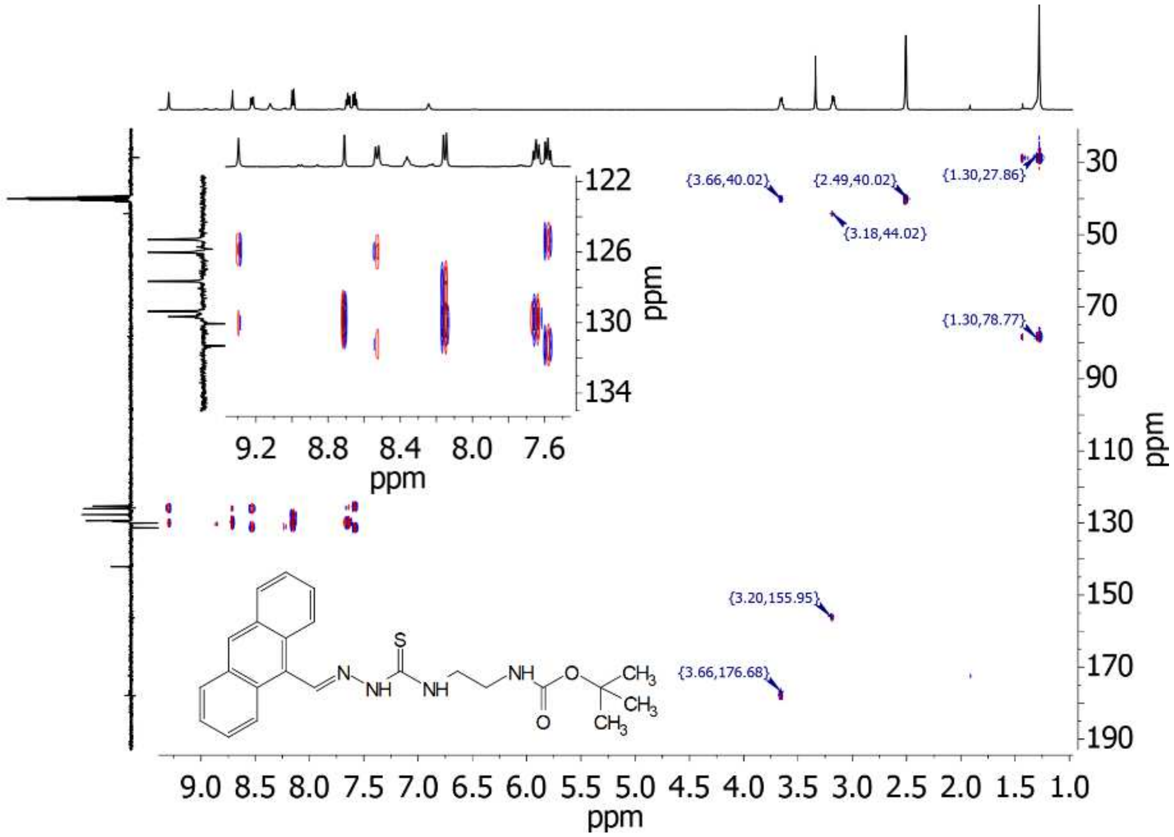

Figure S145. $^{1}H,^{13}C$ HMBC NMR spectrum of 9-anthraldehyde-4-(2-*tert*-butoxycarbonylamino-ethyl)-3-thiosemicarbazone in DMSO-d_6 at 499 MHz.

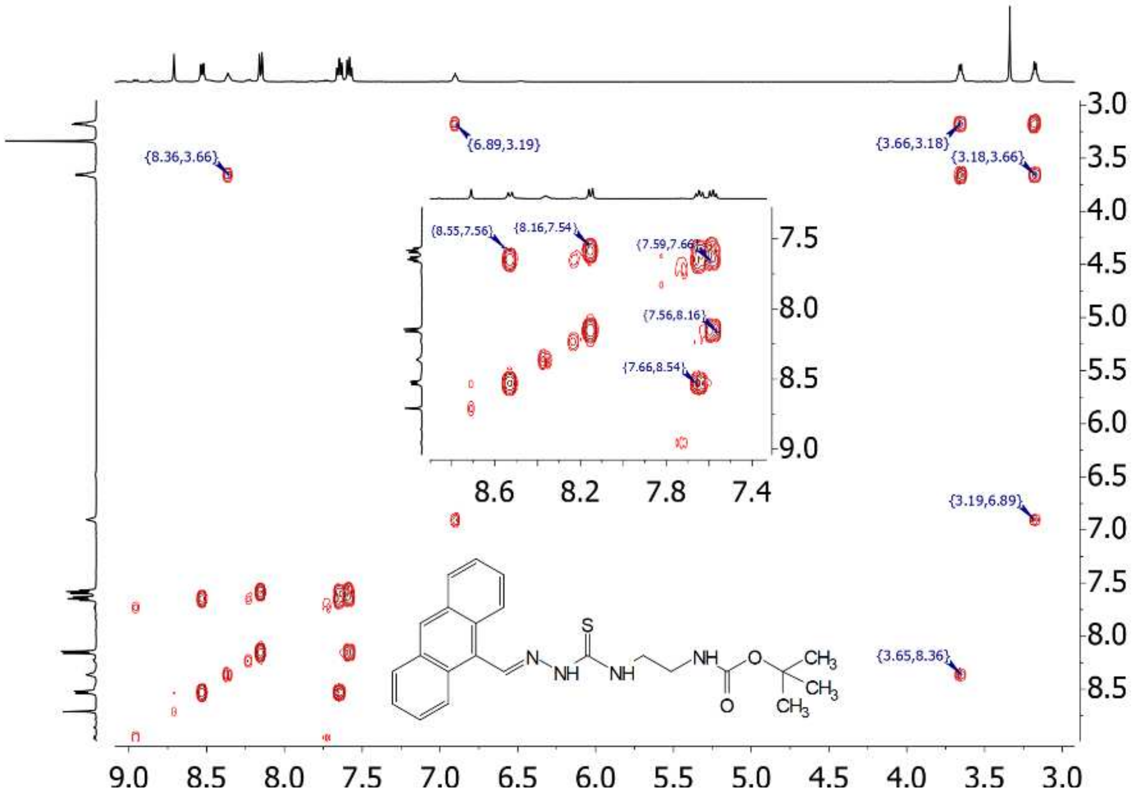

Figure S146. ^{1}H,^{1}H COSY NMR spectrum of 9-anthraldehyde-4-(2-*tert*-butoxycarbonylamino-ethyl)-3-thiosemicarbazone in DMSO-d_6 at 499 MHz.

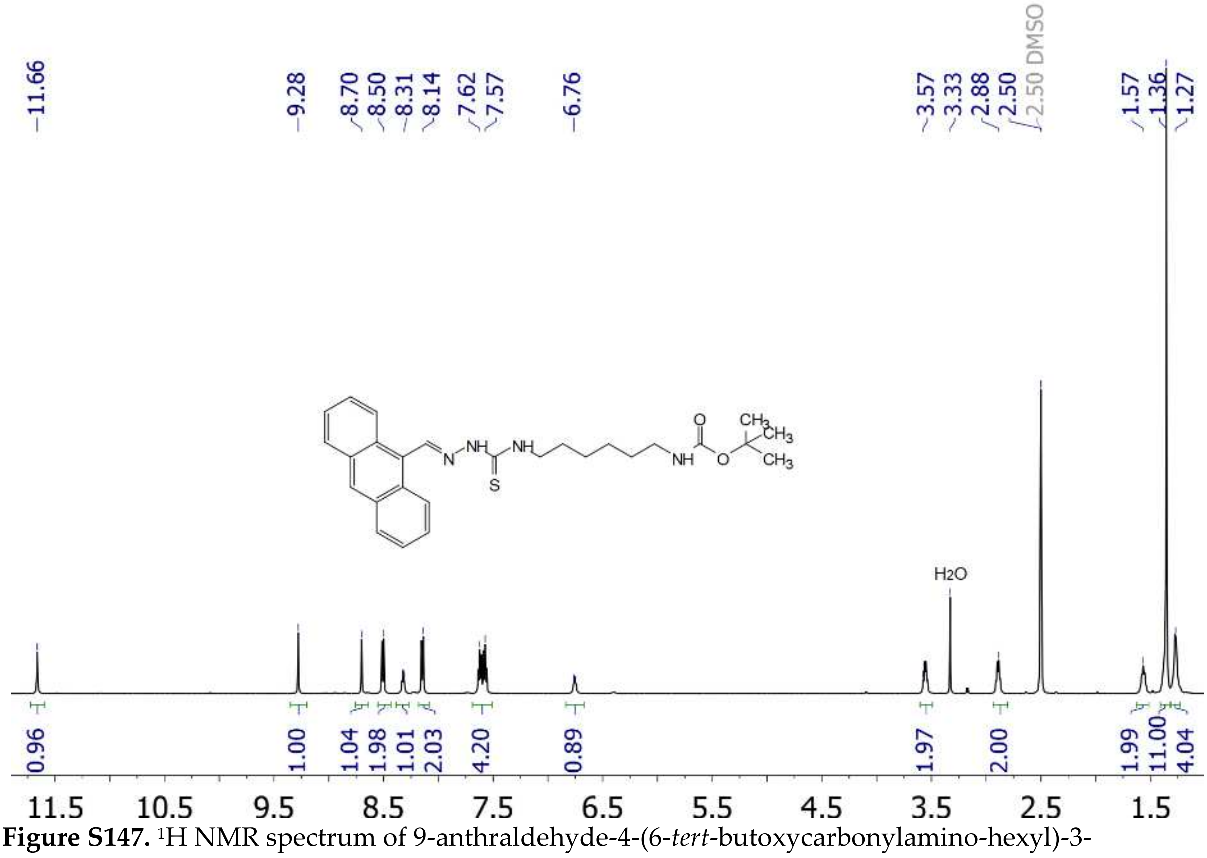

Figure S147. ^{1}H NMR spectrum of 9-anthraldehyde-4-(6-*tert*-butoxycarbonylamino-hexyl)-3-thiosemicarbazone DMSO-d_6 at 499 MHz.

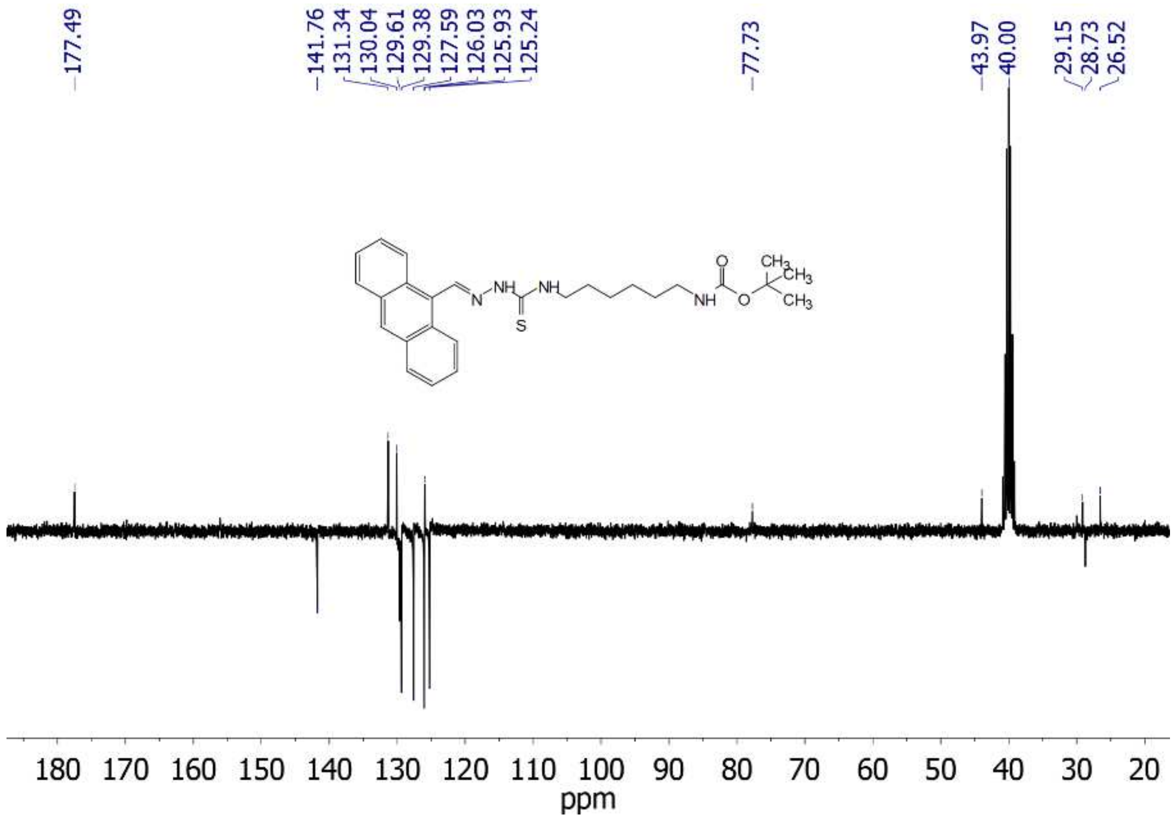

Figure S148. ^{13}C DEPTQ NMR spectrum of 9-anthraldehyde-4-(6-*tert*-butoxycarbonylamino-hexyl)-3-thiosemicarbazone in DMSO-d_6 at 300 MHz.

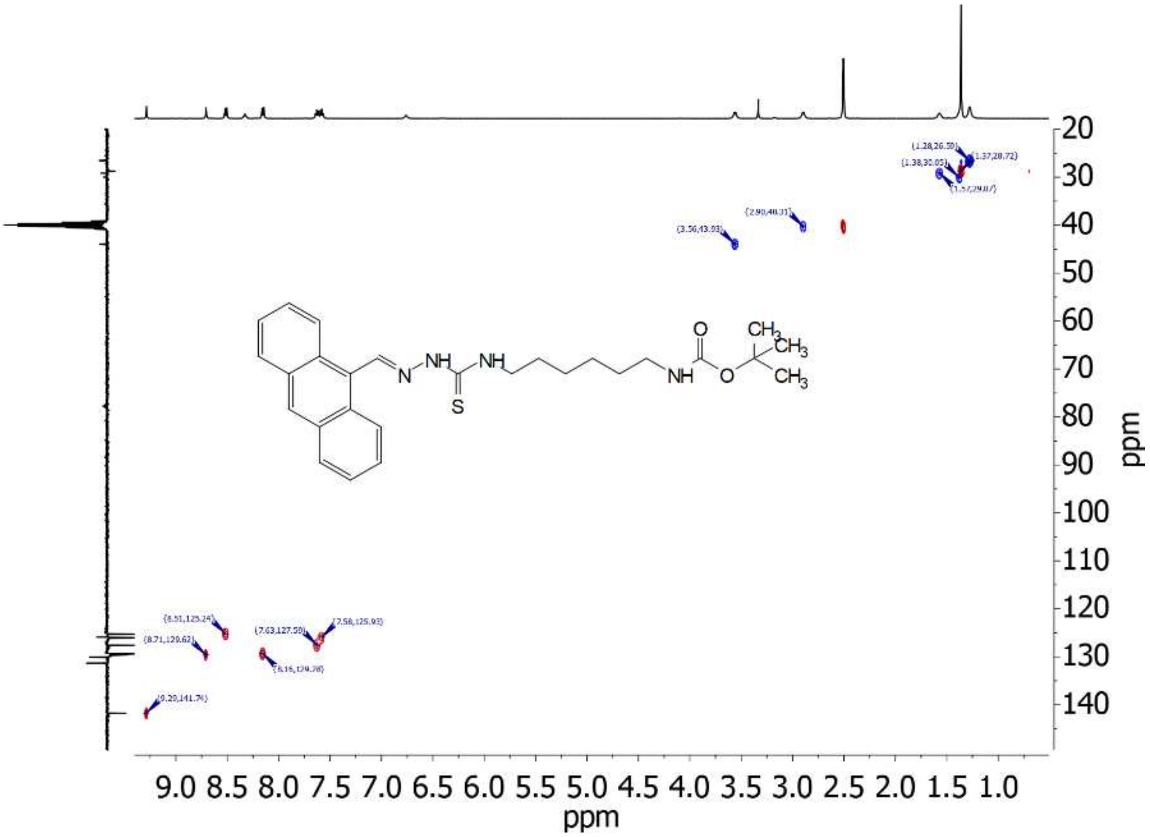

Figure S149. $^{1}H,^{13}C$ HMQC/HSQC NMR spectrum of 9-anthraldehyde-4-(6-*tert*-butoxycarbonylamino-hexyl)-3-thiosemicarbazone in DMSO-d_6 at 499 MHz.

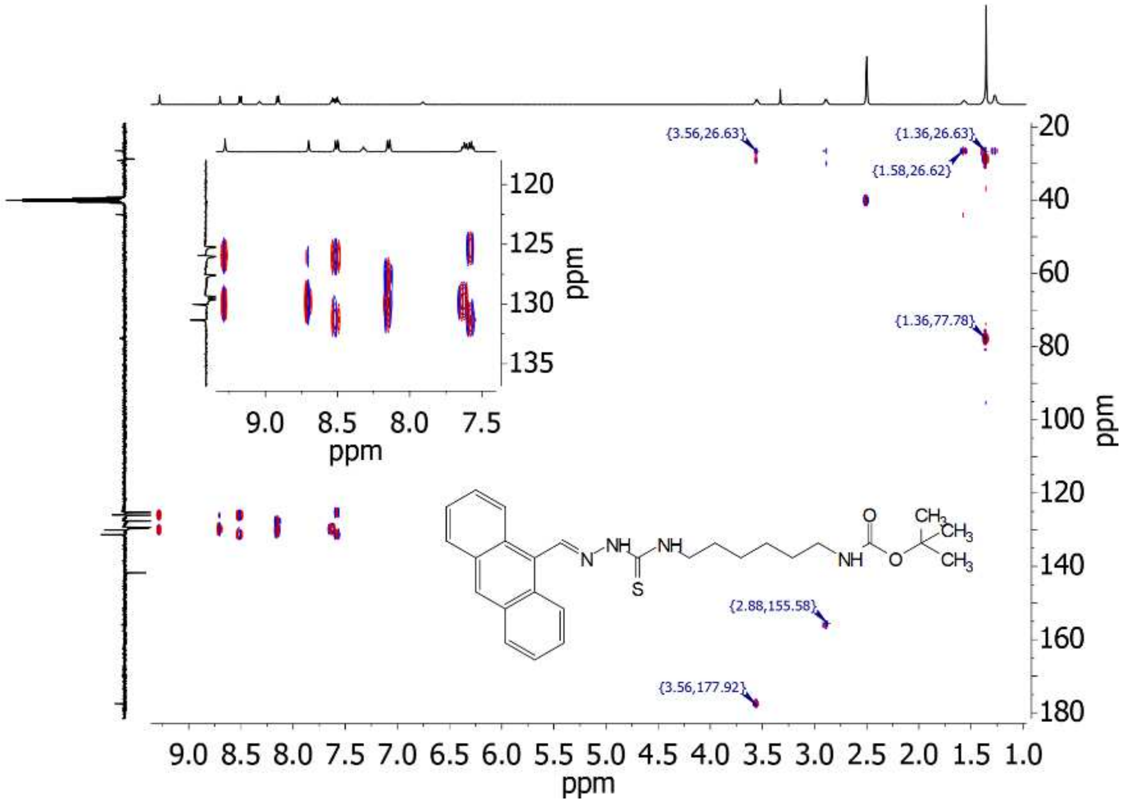

Figure S150. $^{1}H,^{13}C$ HMBC NMR spectrum of 9-anthraldehyde-4-(6-*tert*-butoxycarbonylamino-hexyl)-3-thiosemicarbazone in DMSO-d_6 at 499 MHz.

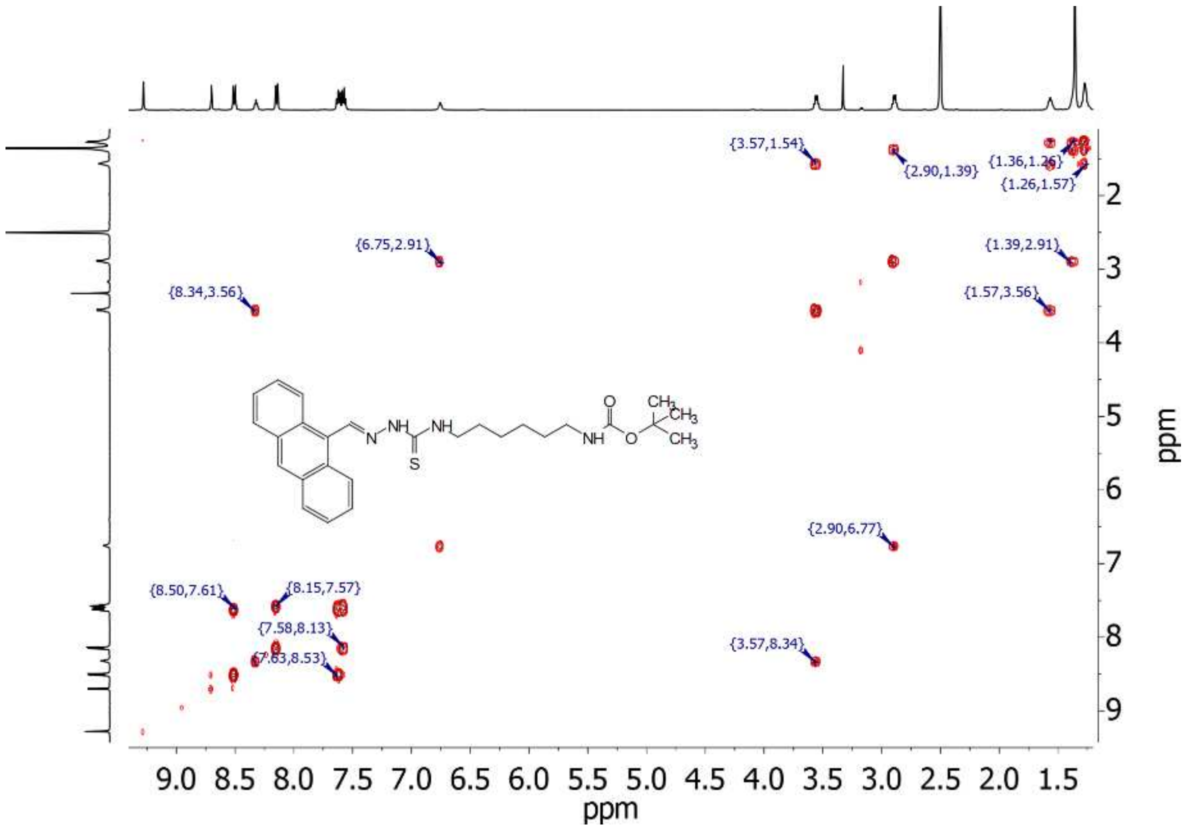

Figure S151. $^{1}H,^{1}H$ COSY NMR spectrum of 9-anthraldehyde-4-(6-*tert*-butoxycarbonylamino-hexyl)-3-thiosemicarbazone in DMSO-d_6 at 499 MHz.

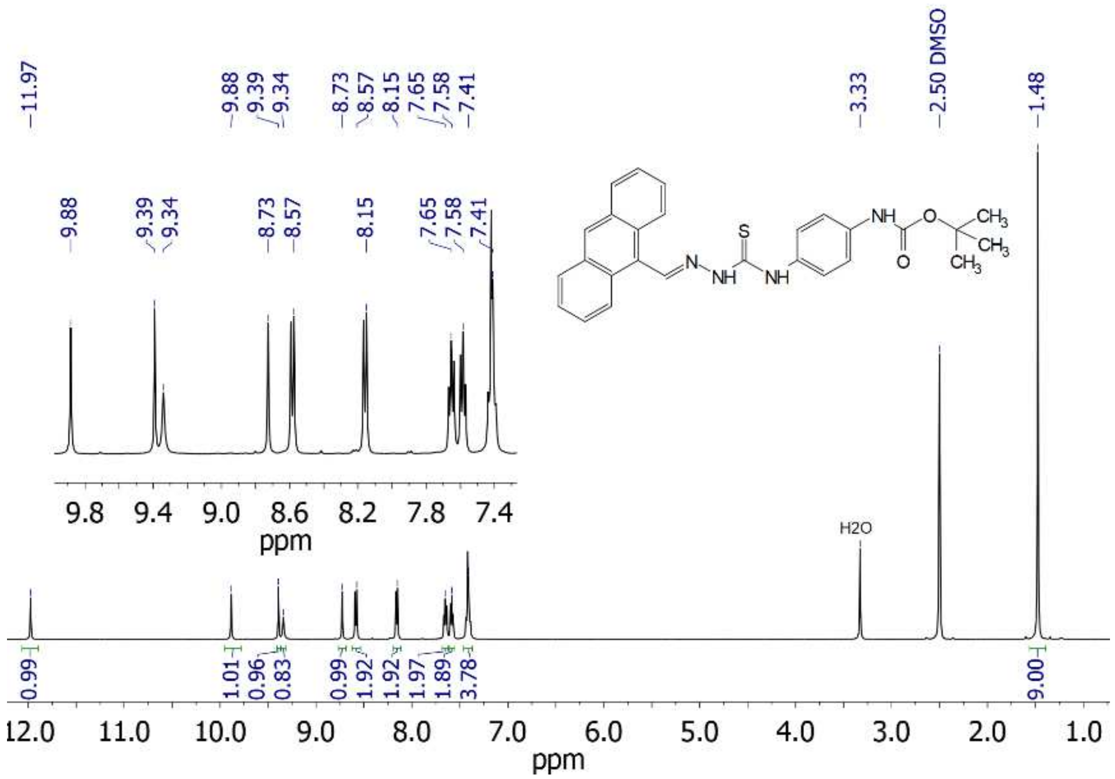

Figure S152. ^{1}H NMR spectrum of 9-anthraldehyde-4-(4-*tert*-butoxycarbonylamino-phenyl)-3-thiosemicarbazone in DMSO-d_6 at 499 MHz.

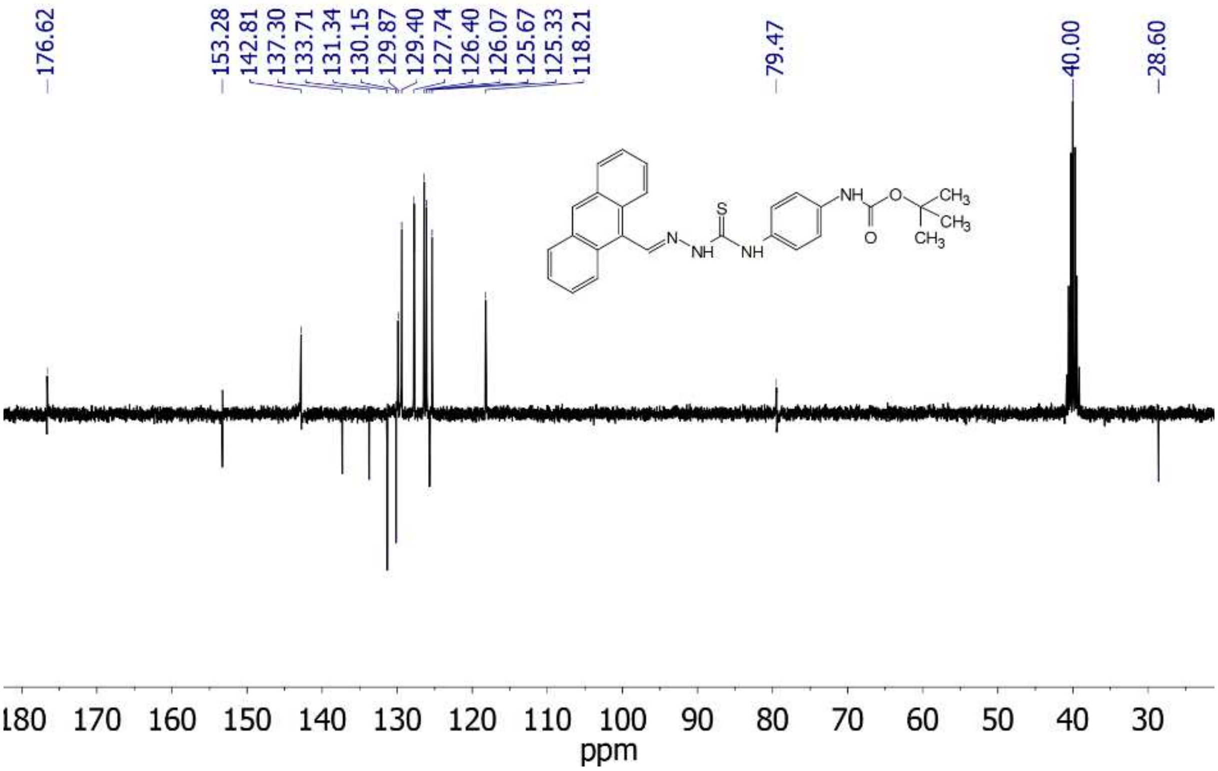

Figure S153. ^{13}C DEPTQ NMR spectrum of 9-anthraldehyde-4-(4-*tert*-butoxycarbonylamino-phenyl)-3-thiosemicarbazone in DMSO-d_6 at 300 MHz.

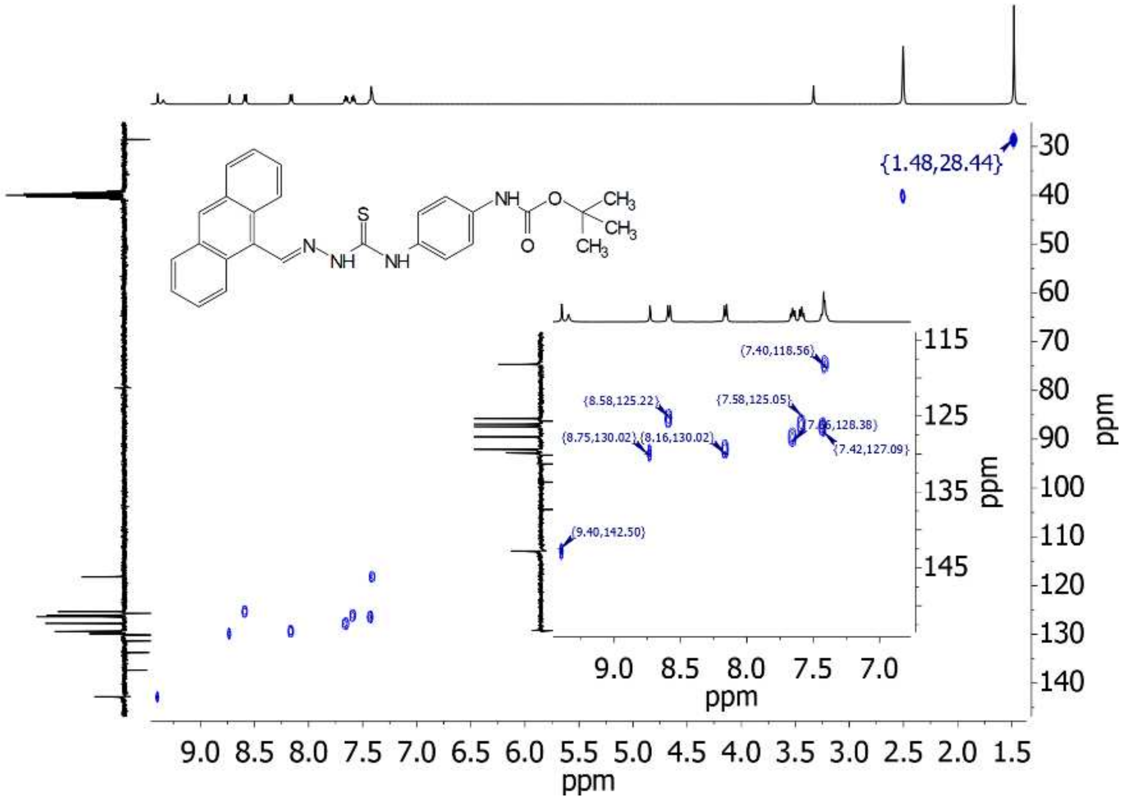

Figure S154. ^{1}H,^{13}C HMQC/HSQC NMR spectrum of 9-anthraldehyde-4-(4-*tert*-butoxycarbonylamino-phenyl)-3-thiosemicarbazone in DMSO-d_6 at 499 MHz.

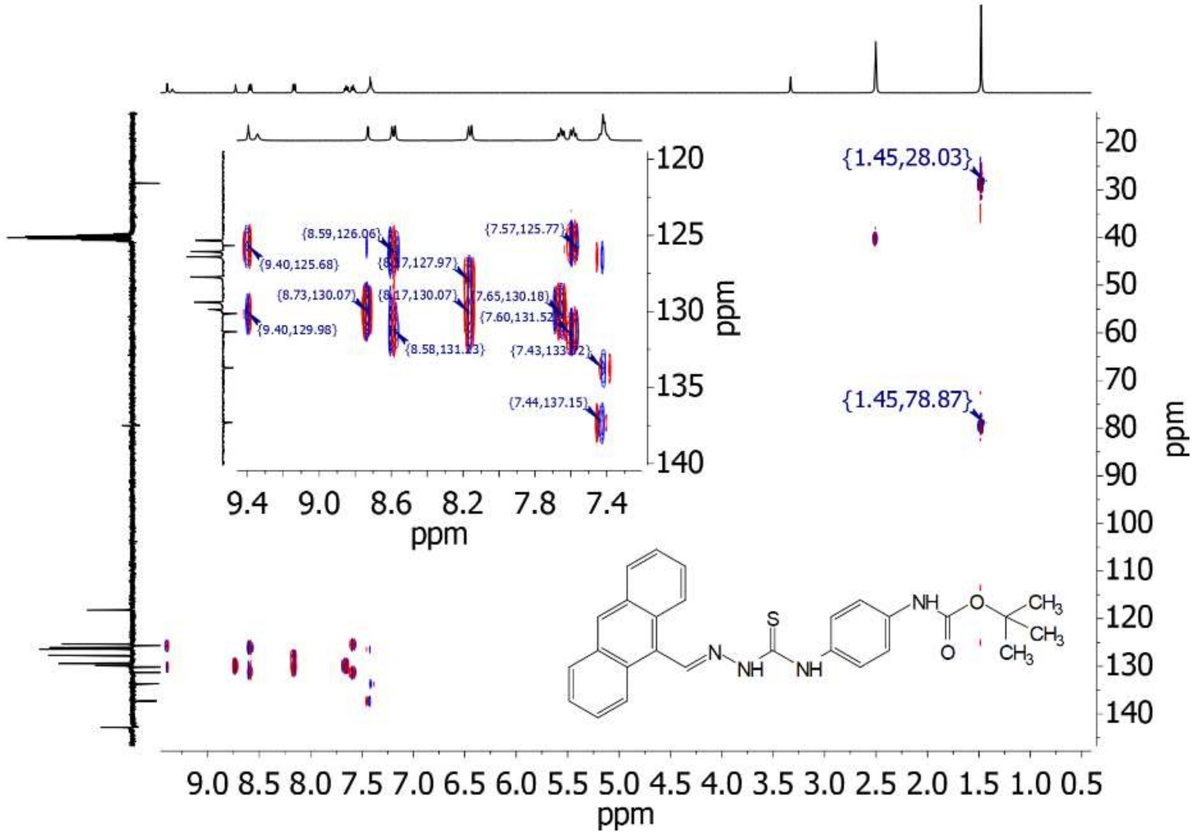

Figure S155. ^{1}H,^{13}C HMBC NMR spectrum of 9-anthraldehyde-4-(4-*tert*-butoxycarbonylamino-phenyl)-3-thiosemicarbazone in DMSO-d_6 at 499 MHz.

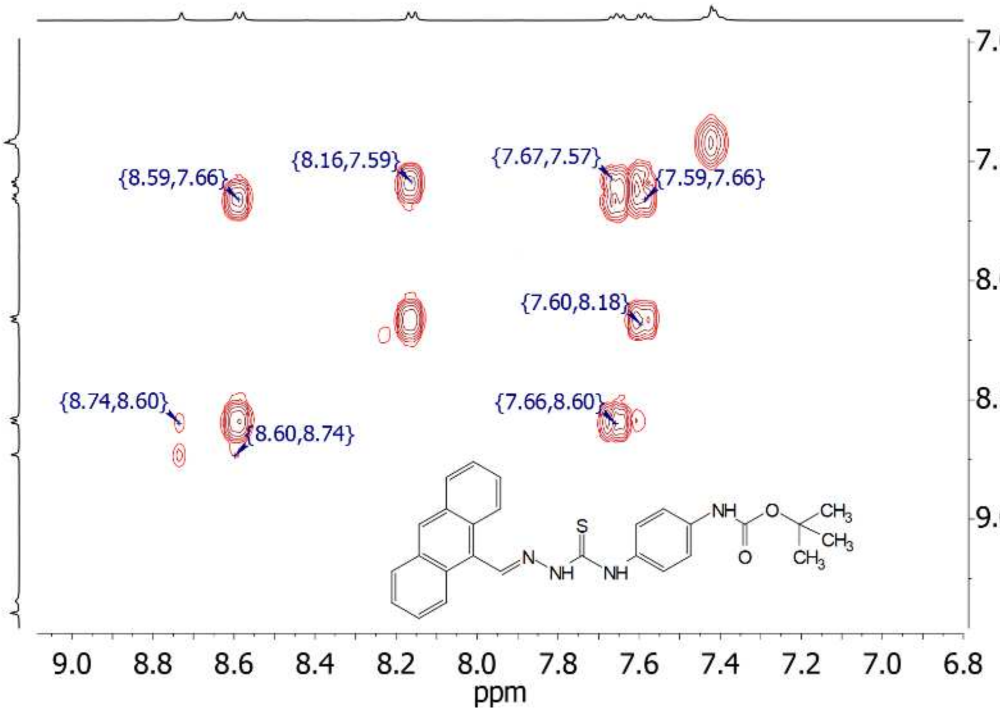

Figure S156. $^{1}H,^{1}H$ COSY NMR spectrum of 9-anthraldehyde-4-(4-*tert*-butoxycarbonylamino-phenyl)-3-thiosemicarbazone in DMSO-d_6 at 499 MHz.

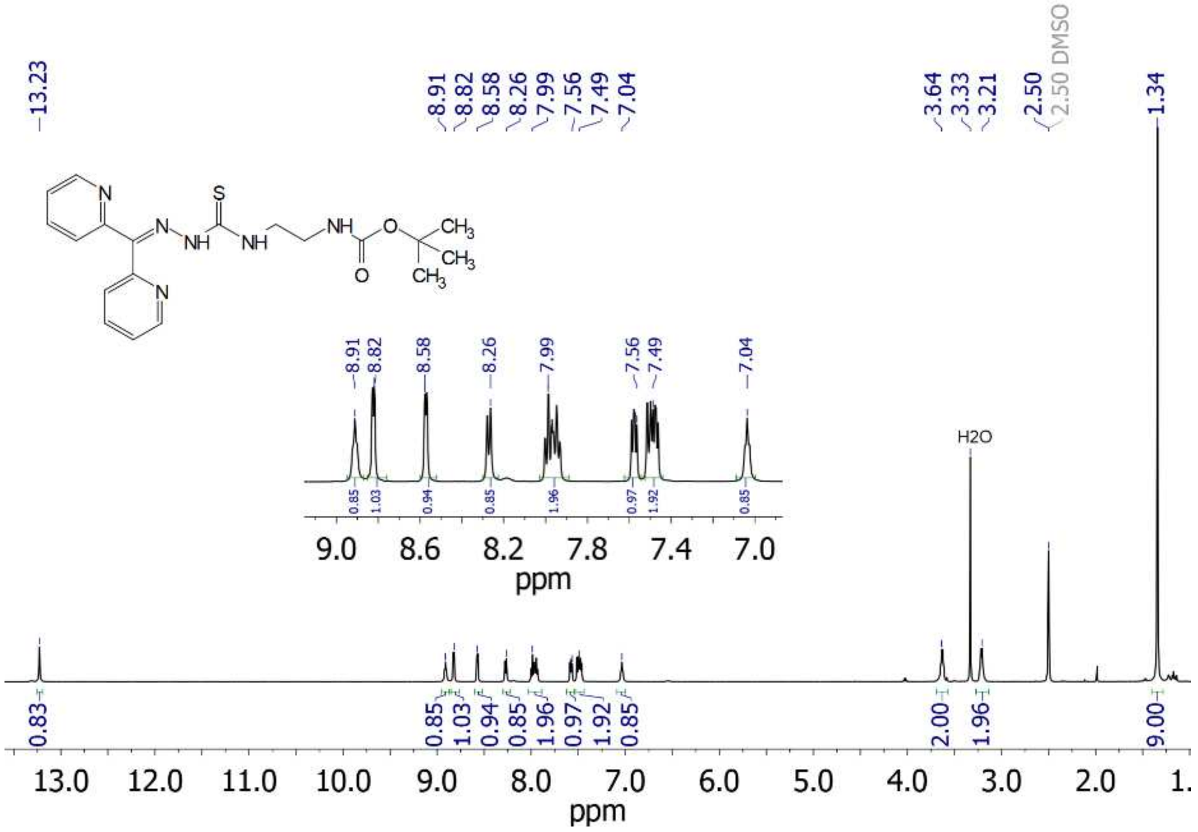

Figure S157. ^{1}H NMR spectrum of di-2-pyridylketone-4-(2-*tert*-butoxycarbonylamino-ethyl)-3-thiosemicarbazone in DMSO-d_6 at 499 MHz.

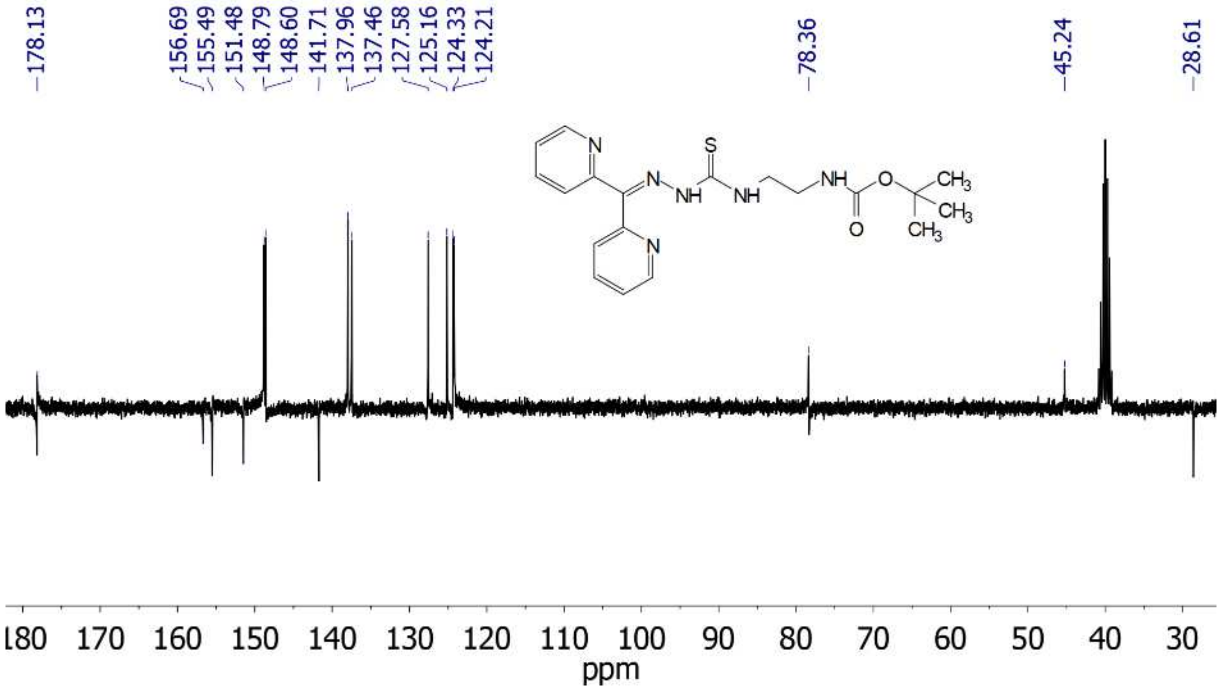

Figure S158. ^{13}C DEPTQ NMR spectrum of di-2-pyridylketone-4-(2-*tert*-butoxycarbonylamino-ethyl)-3-thiosemicarbazone in DMSO-d_6 at 300 MHz.

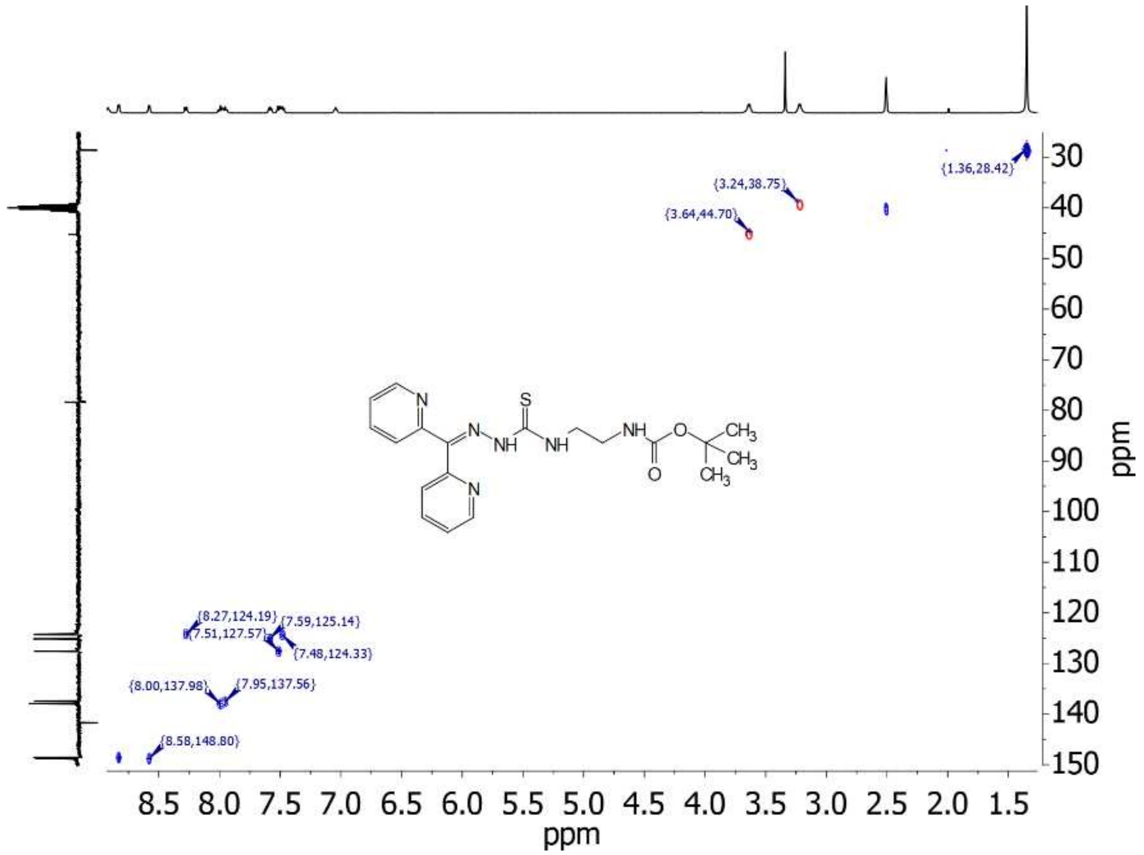

Figure S159. ^{1}H,^{13}C HMQC/HSQC NMR spectrum of di-2-pyridylketone-4-(2-*tert*-butoxycarbonyl-amino-ethyl)-3-thiosemicarbazone in DMSO-d_6 at 499 MHz.

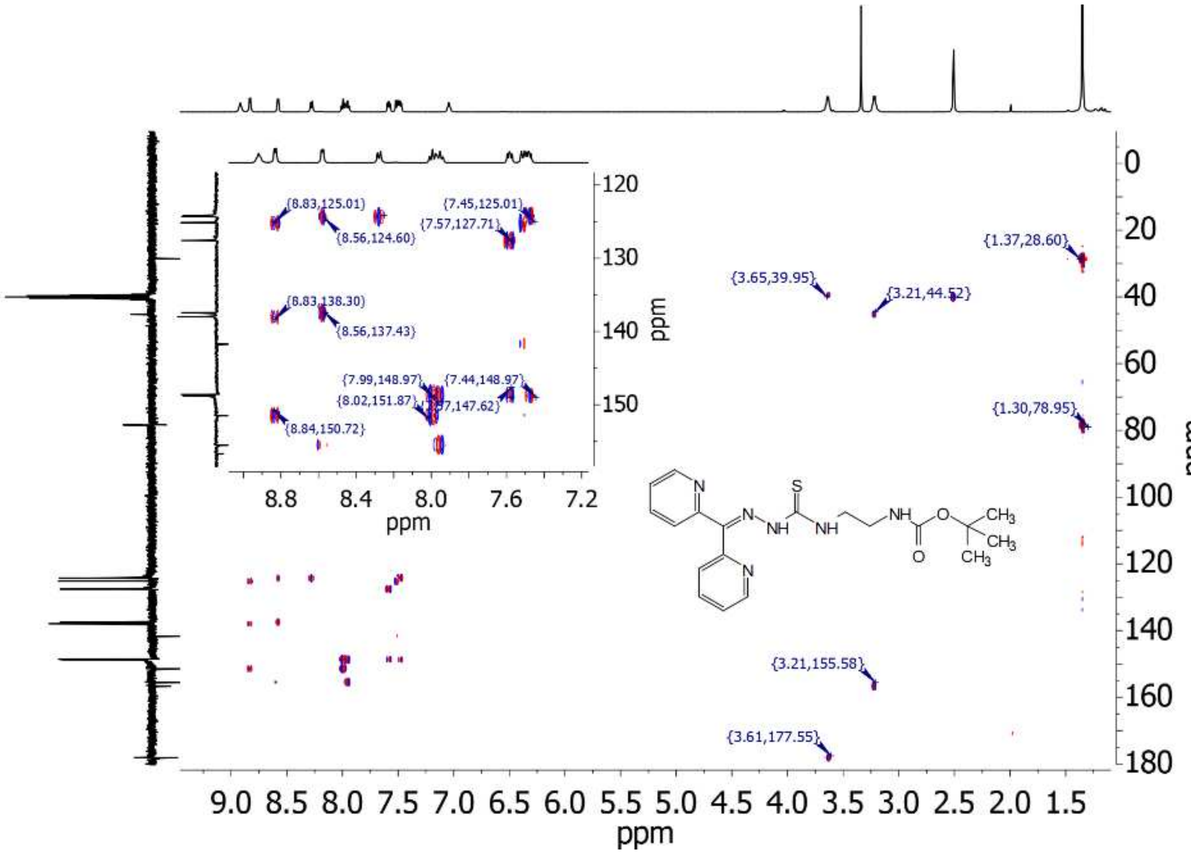

Figure S160. ^{1}H,^{13}C HMBC NMR spectrum of di-2-pyridylketone-4-(2-*tert*-butoxycarbonylamino-ethyl)-3-thiosemicarbazone in DMSO-d_6 at 499 MHz.

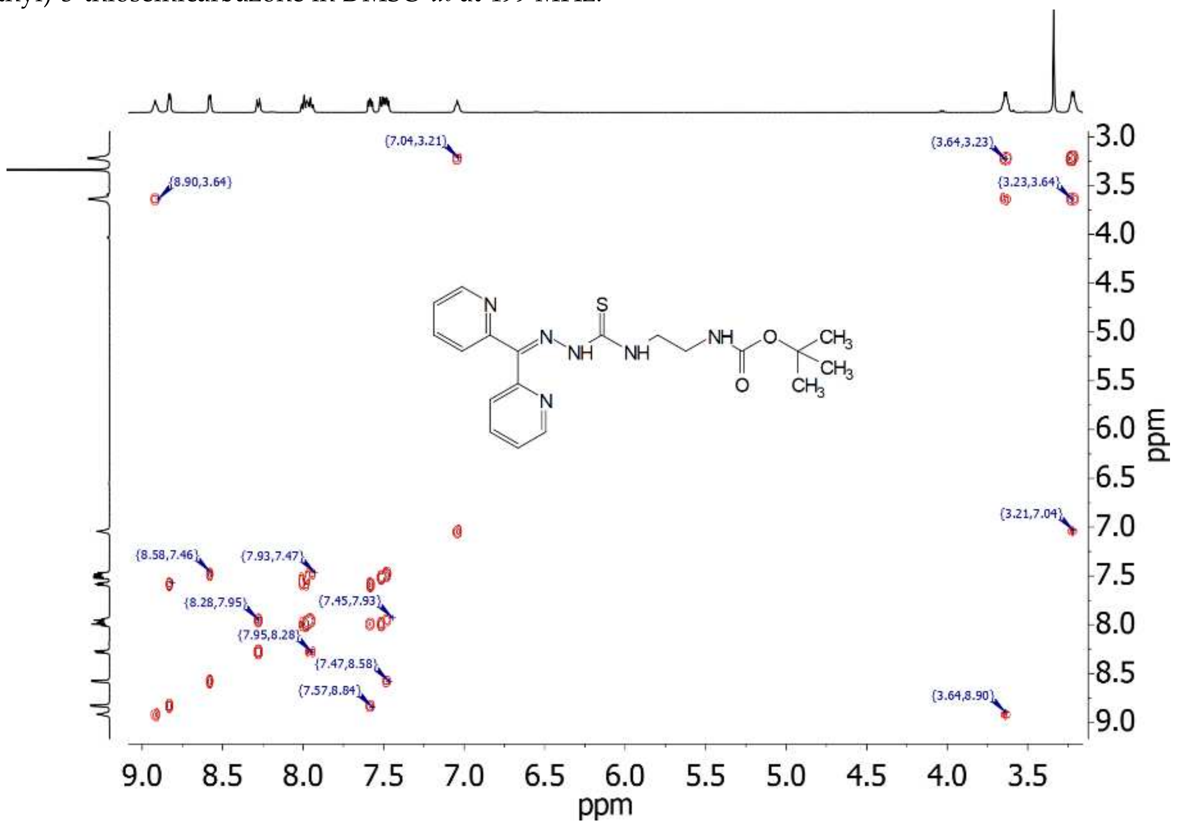

Figure S161. ^{1}H,^{1}H COSY NMR spectrum of di-2-pyridylketone-4-(2-*tert*-butoxycarbonylamino-ethyl)-3-thiosemicarbazone in DMSO-d_6 at 499 MHz.

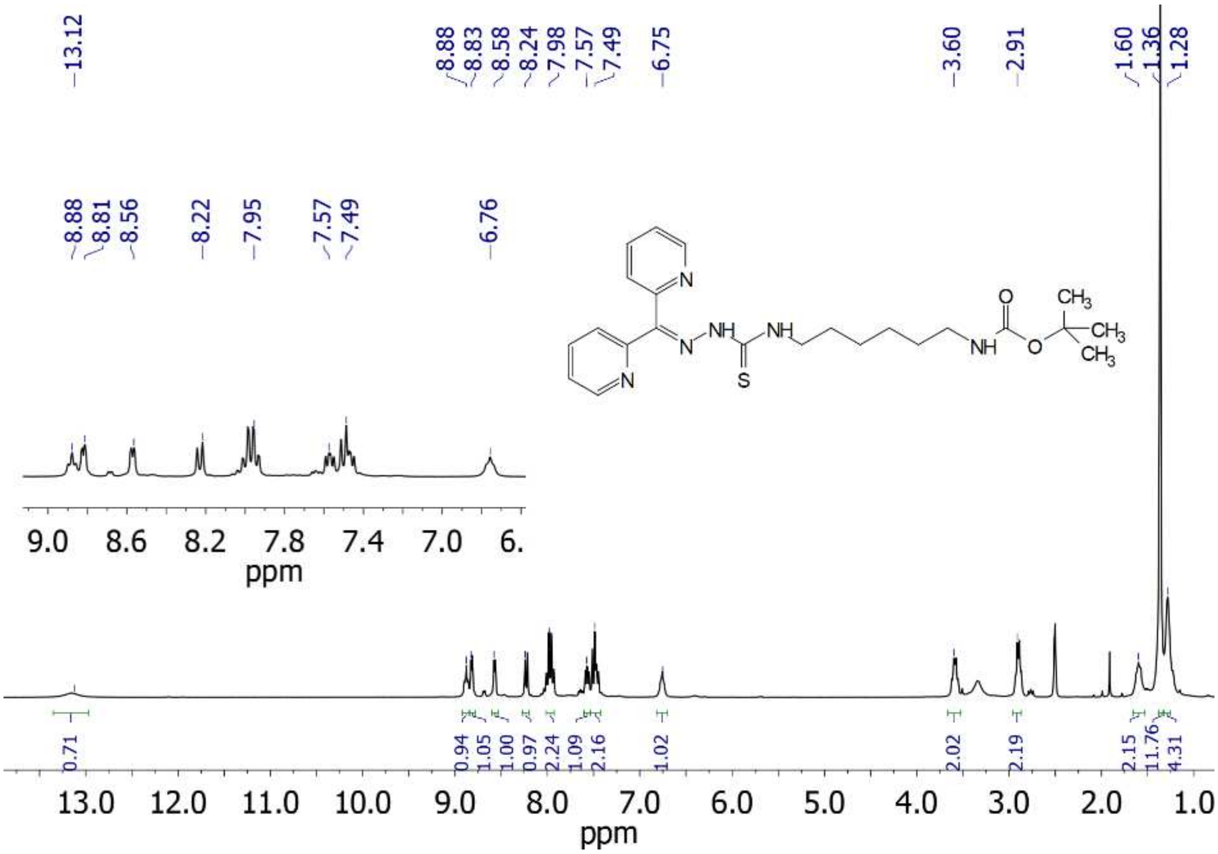

Figure S162. ^{1}H NMR spectrum of di-2-pyridylketone-4-(6-*tert*-butoxycarbonylamino-hexyl)-3-thiosemicarbazone in DMSO-d_6 at 499 MHz.

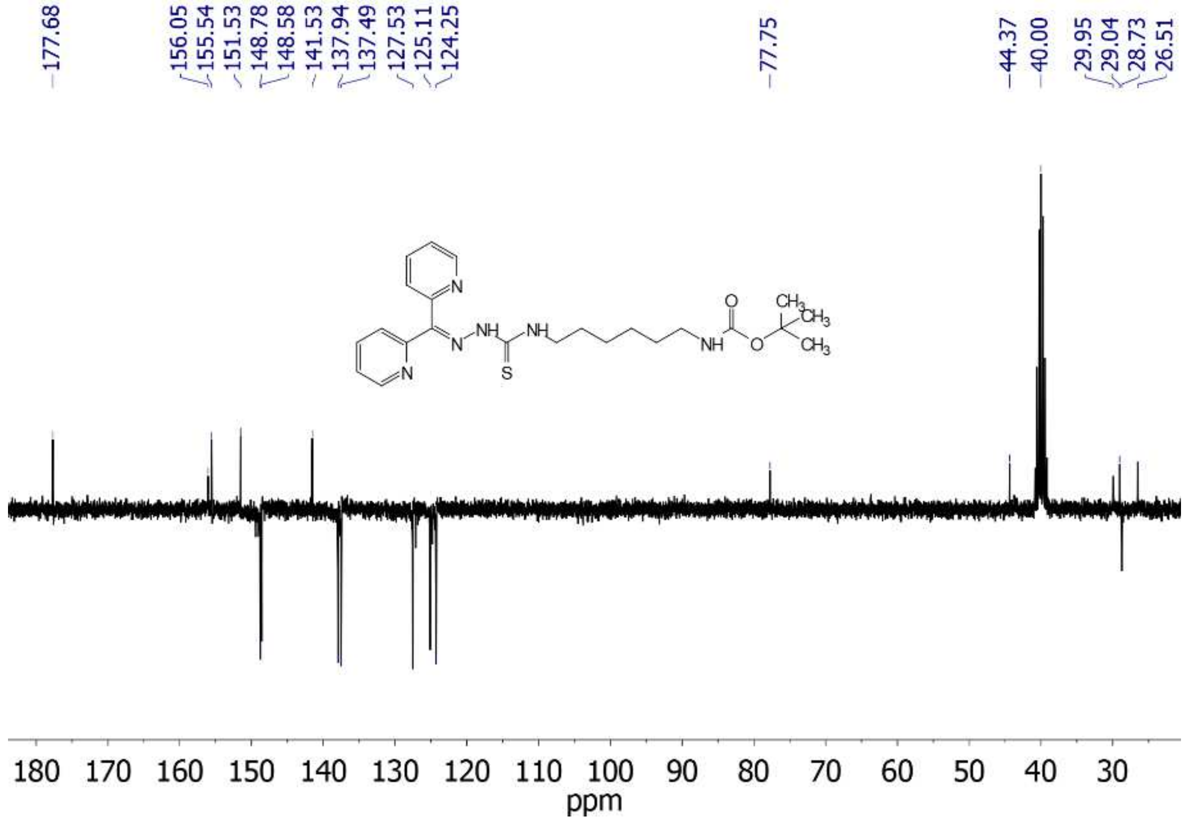

Figure S163. ^{13}C DEPTQ NMR spectrum of di-2-pyridylketone-4-(6-*tert*-butoxycarbonylamino-hexyl)-3-thiosemicarbazone in DMSO-d_6 at 300 MHz.

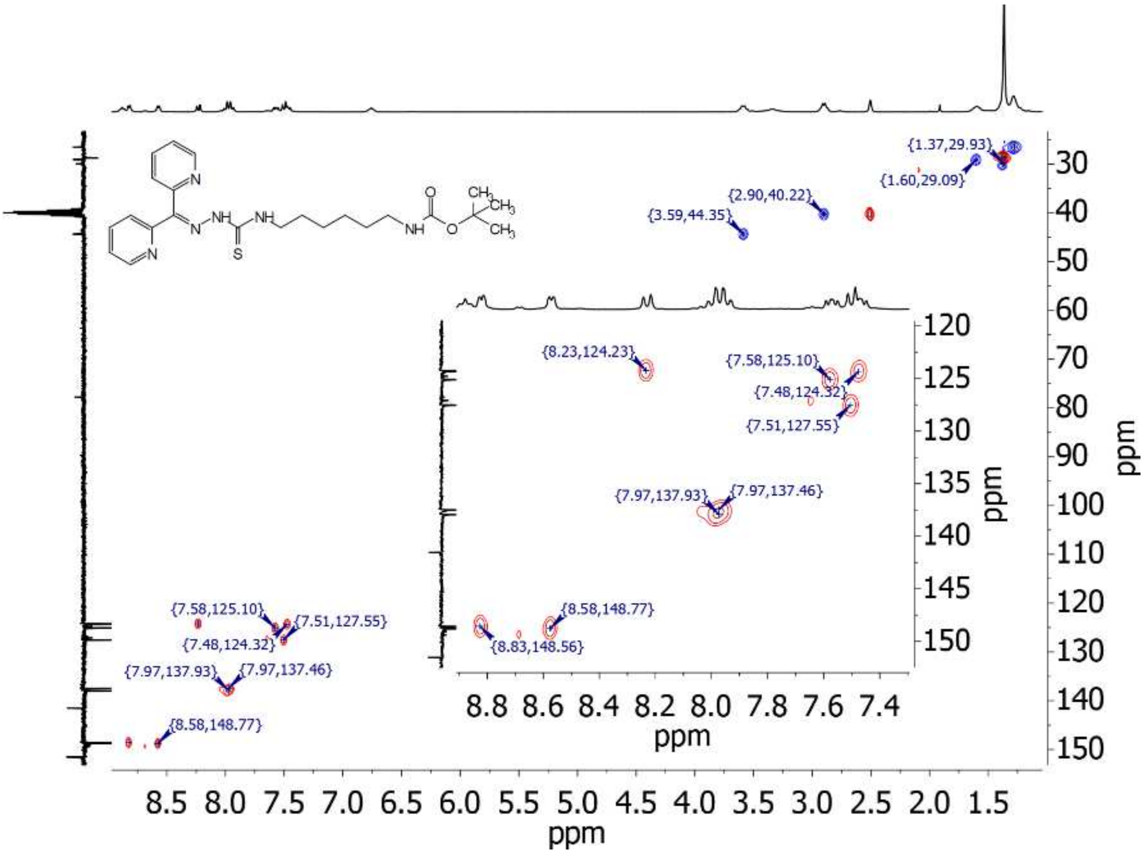

Figure S164. $^{1}H,^{13}C$ HMQC/HSQC NMR spectrum of di-2-pyridylketone-4-(6-*tert*-butoxycarbonyl-amino-hexyl)-3-thiosemicarbazone in DMSO-d_6 at 499 MHz.

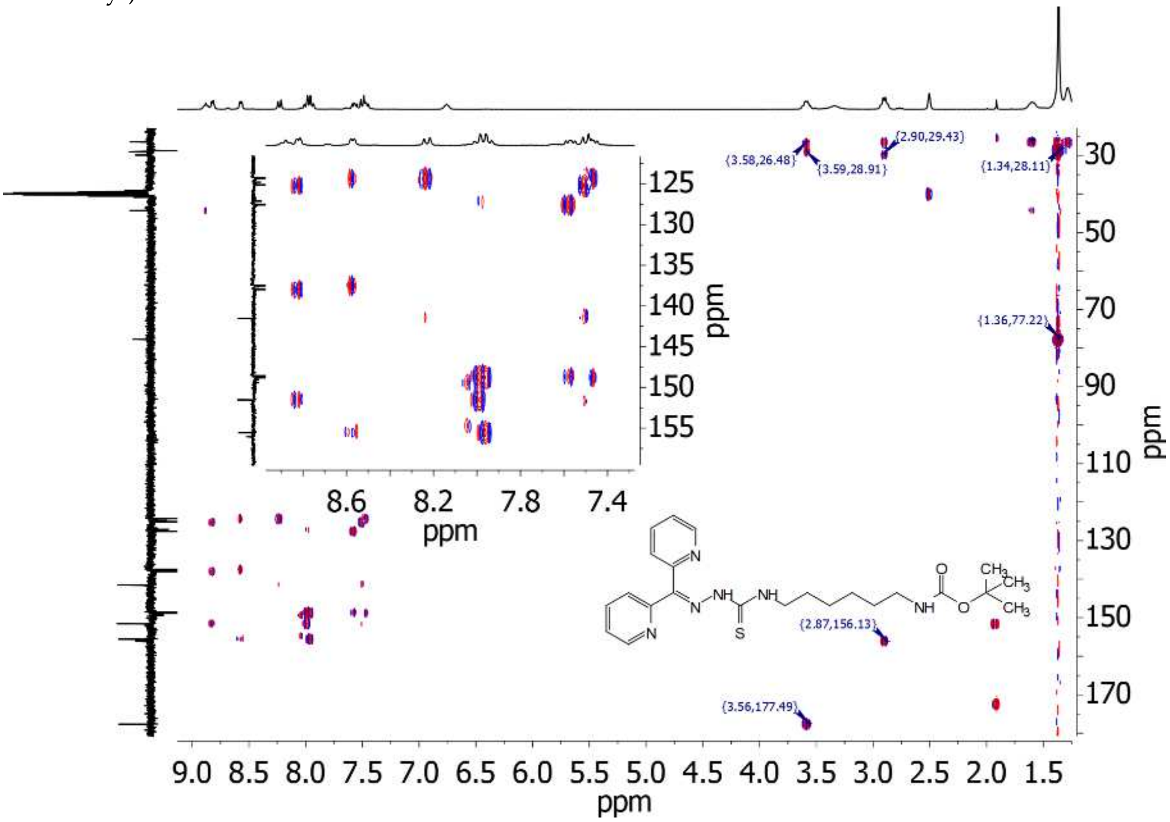

Figure S165. $^{1}H,^{13}C$ HMBC NMR spectrum of di-2-pyridylketone-4-(6-*tert*-butoxycarbonylamino-hexyl)-3-thiosemicarbazone in DMSO-d_6 at 499 MHz.

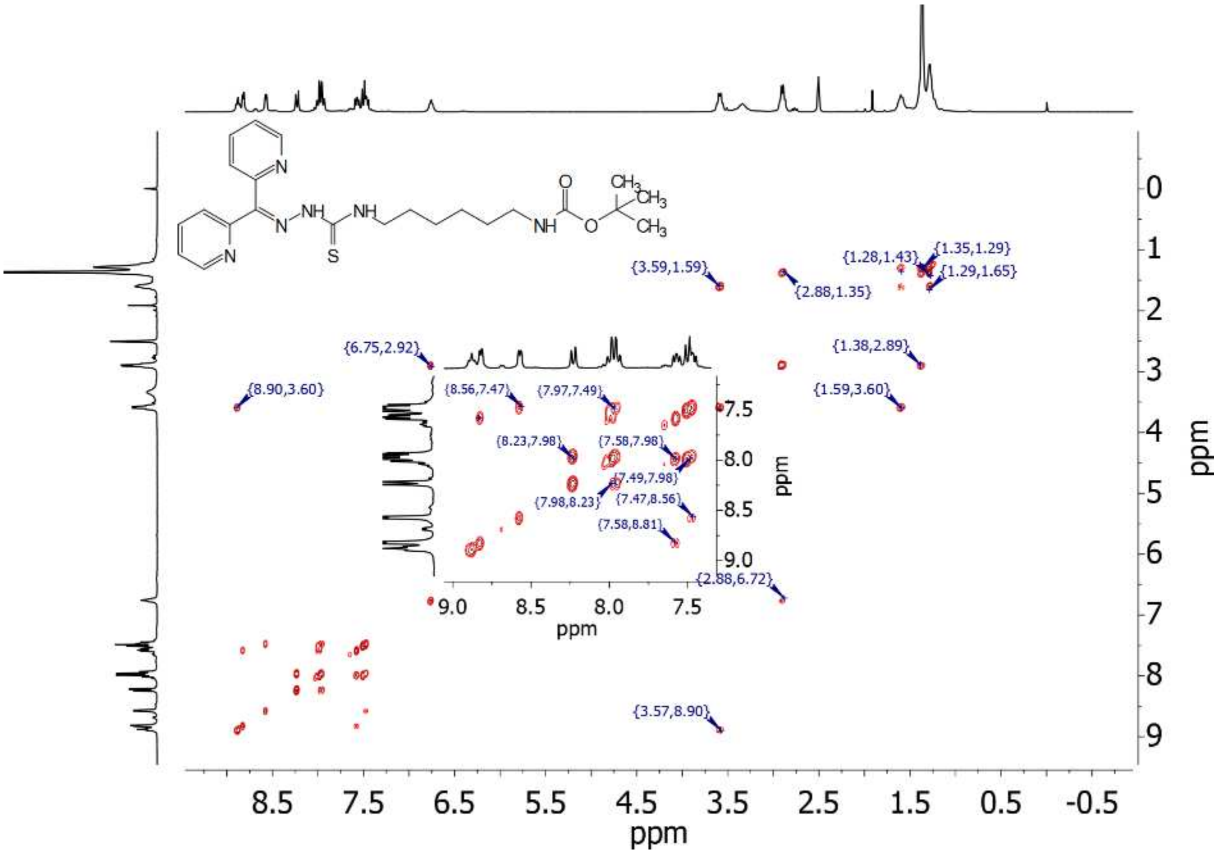

Figure S166. ^{1}H,^{1}H COSY NMR spectrum of di-2-pyridylketone-4-(6-*tert*-butoxycarbonylamino-hexyl)-3-thiosemicarbazone in DMSO-d_6 at 499 MHz.

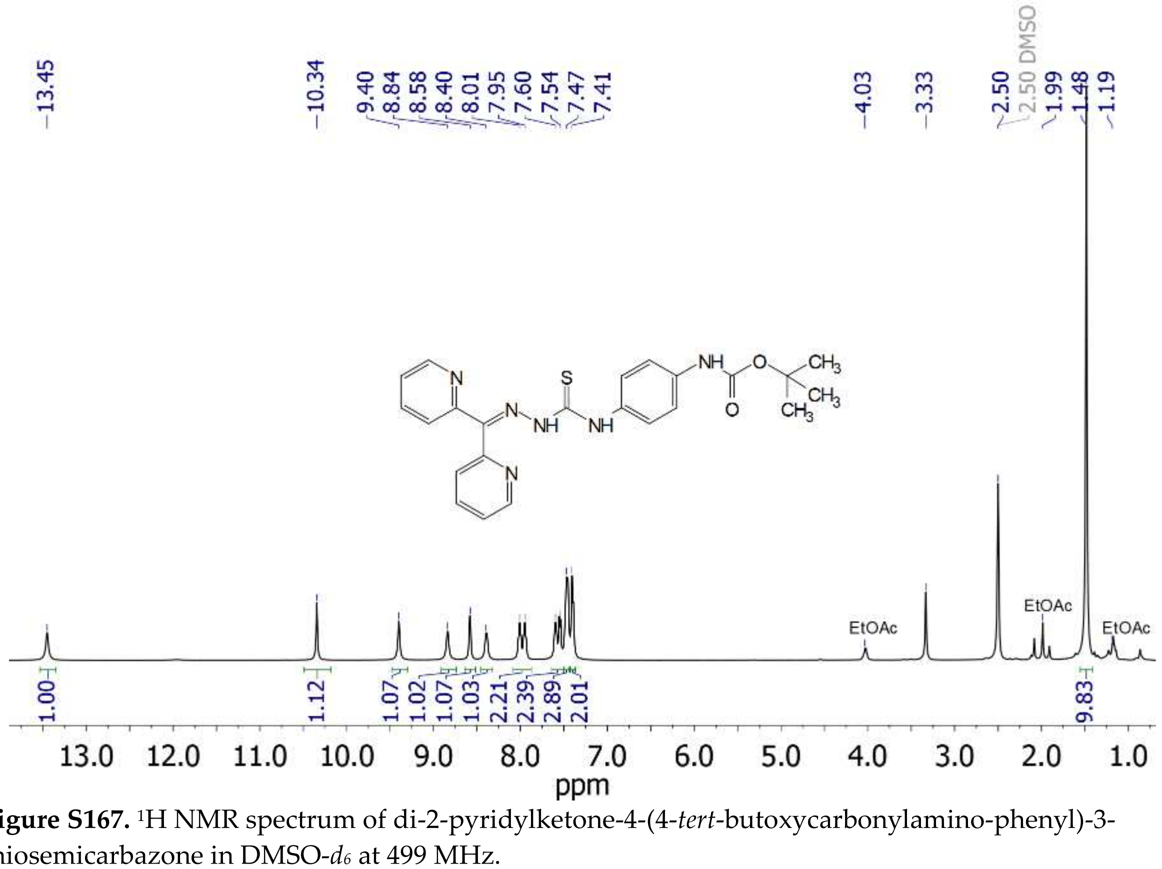

Figure S167. ^{1}H NMR spectrum of di-2-pyridylketone-4-(4-*tert*-butoxycarbonylamino-phenyl)-3-thiosemicarbazone in DMSO-d_6 at 499 MHz.

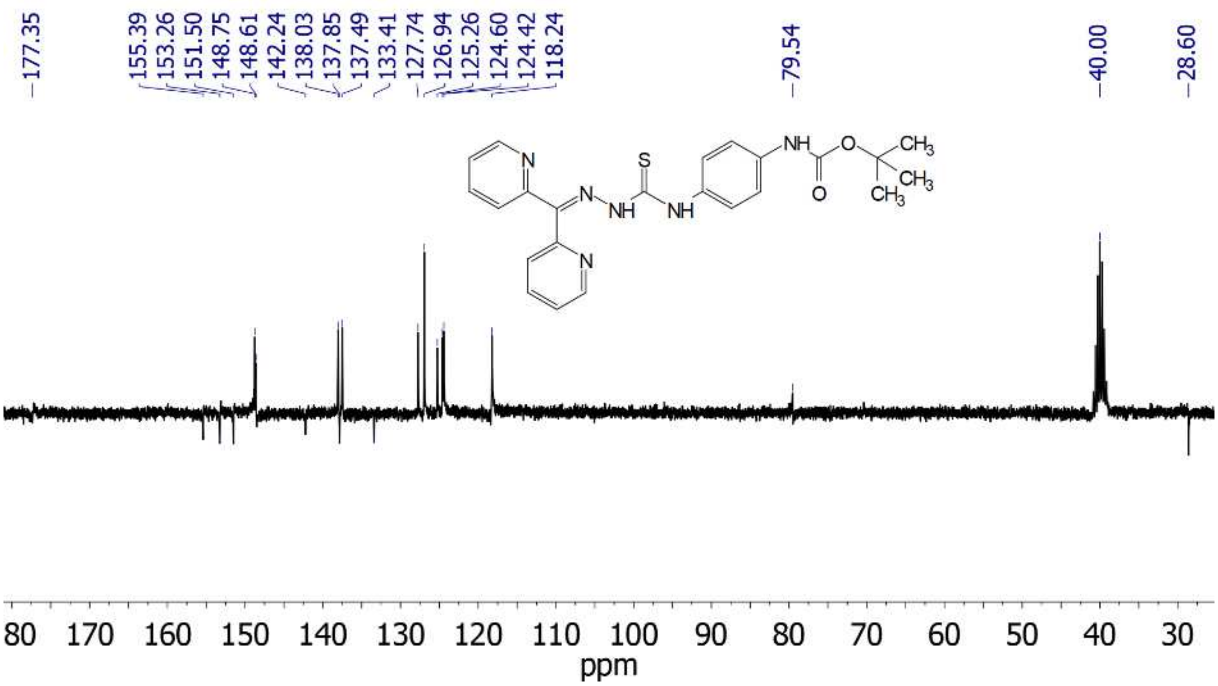

Figure S168. ^{13}C DEPTQ NMR spectrum of di-2-pyridylketone-4-(4-*tert*-butoxycarbonylamino-phenyl)-3-thiosemicarbazone in DMSO-d_6 at 300 MHz.

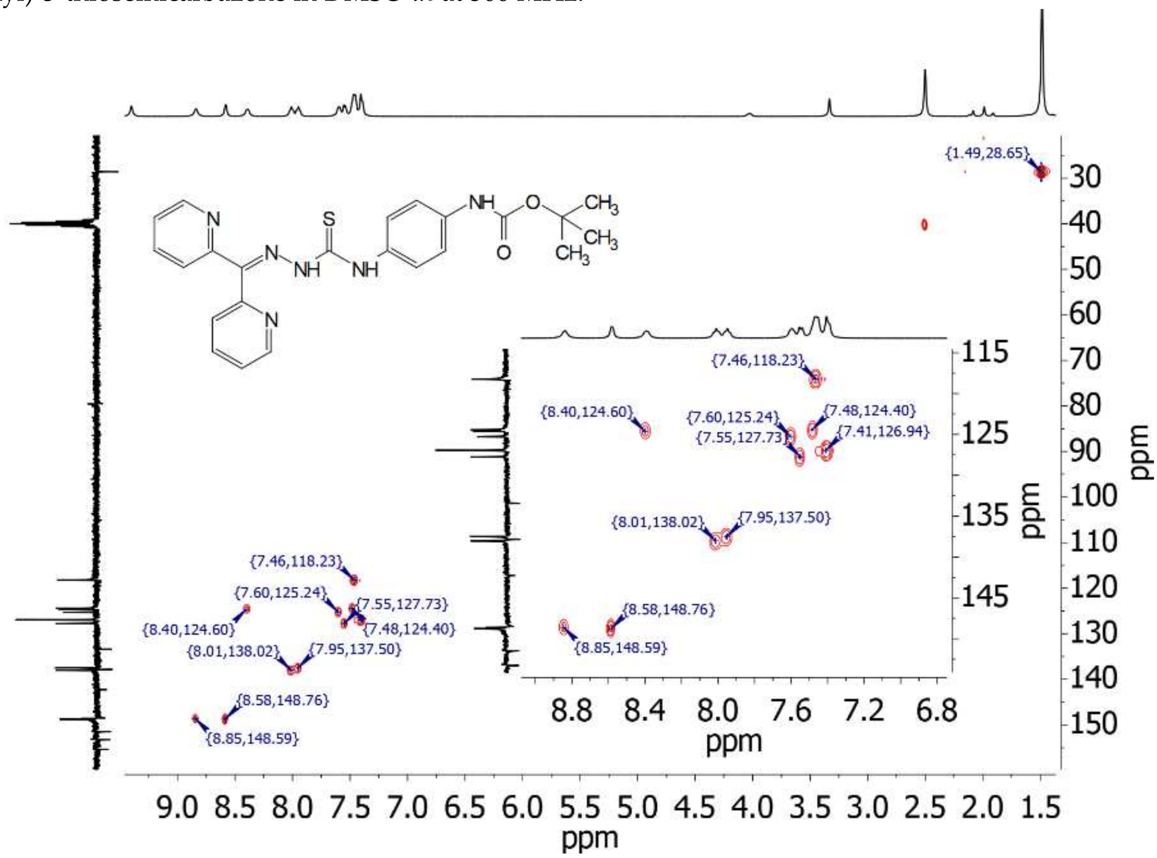

Figure S169. ^{1}H,^{13}C HMQC/HSQC NMR spectrum of di-2-pyridylketone-4-(4-*tert*-butoxycarbonyl-amino-phenyl)-3-thiosemicarbazone in DMSO-d_6 at 499 MHz.

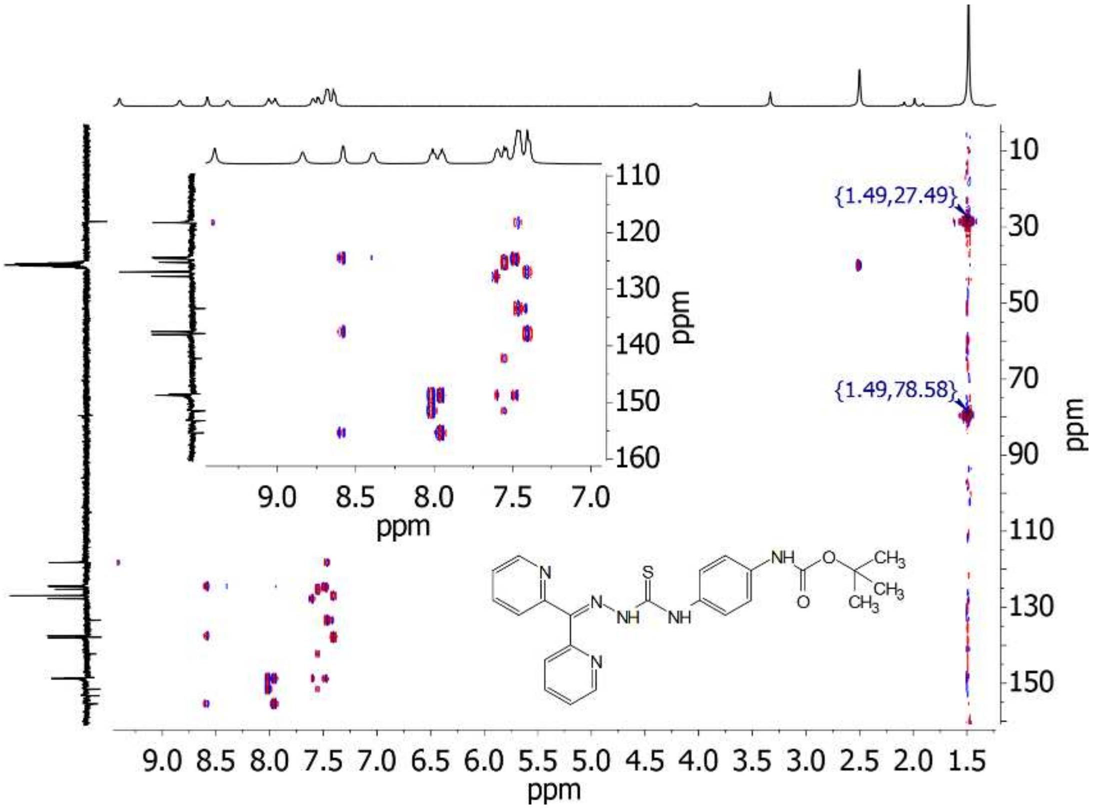

Figure S170. $^{1}H,^{13}C$ HMBC NMR spectrum of di-2-pyridylketone-4-(4-*tert*-butoxycarbonylamino-phenyl)-3-thiosemicarbazone in DMSO-d_6 at 499 MHz.

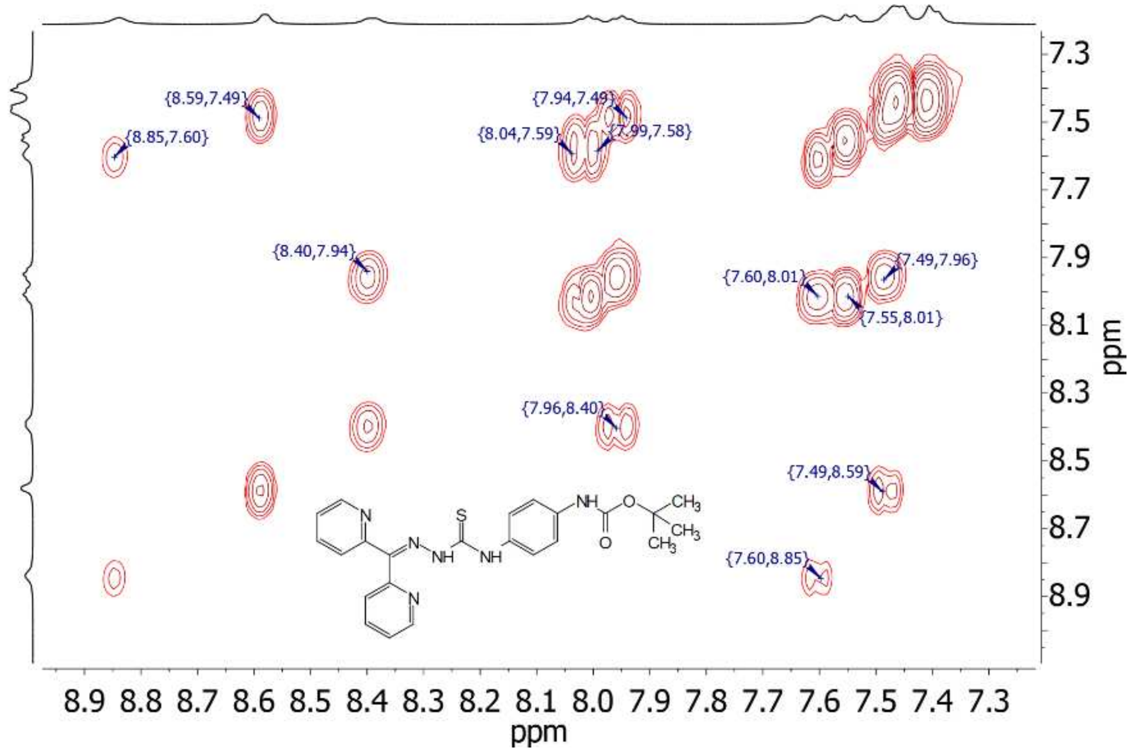

Figure S171. $^{1}H,^{1}H$ COSY NMR spectrum of di-2-pyridylketone-4-(4-*tert*-butoxycarbonylamino-phenyl)-3-thiosemicarbazone DMSO-d_6 at 499 MHz.

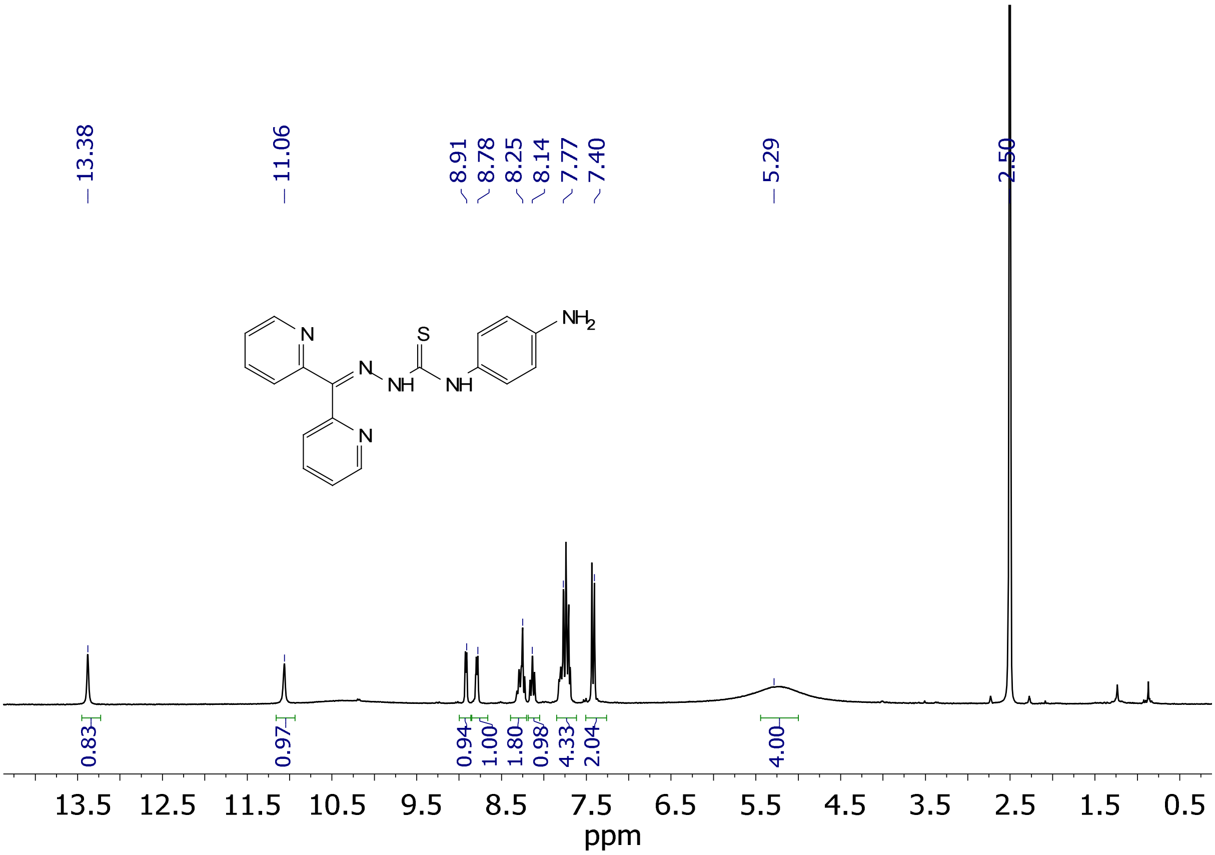

Figure S172. ^{1}H NMR spectrum of (4-aminophenylamino)[bis(pyridin-2-yl)methylideneamino] carbothioamide in DMSO-d_6 at 300 MHz.

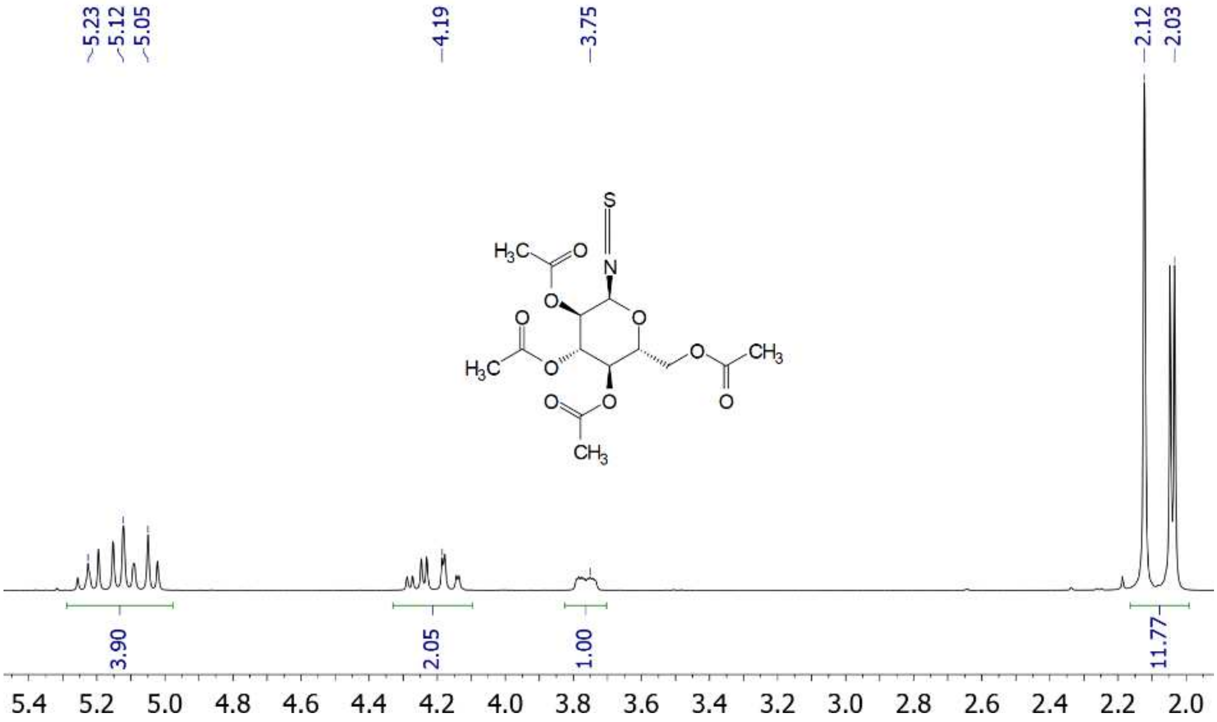

Figure S173. ^{1}H NMR spectrum of β-D-Glucopyranosyl isothiocyanate in $CDCl_3$ at 300 MHz.

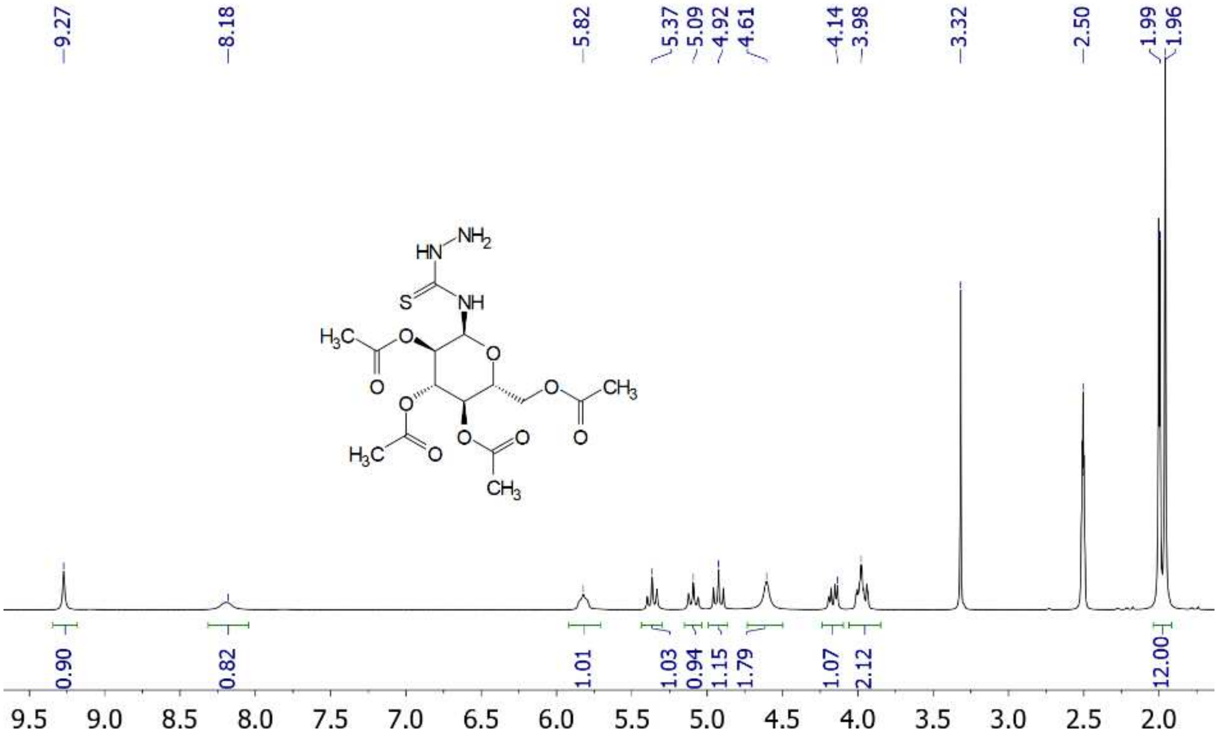

Figure S174. ^{1}H NMR spectrum of *N*-(2,3,4,6-Tetra-*O*-acetyl-β-D-glucopyranosyl)hydrazine-carbothioamide in $CDCl_3$ at 300 MHz.

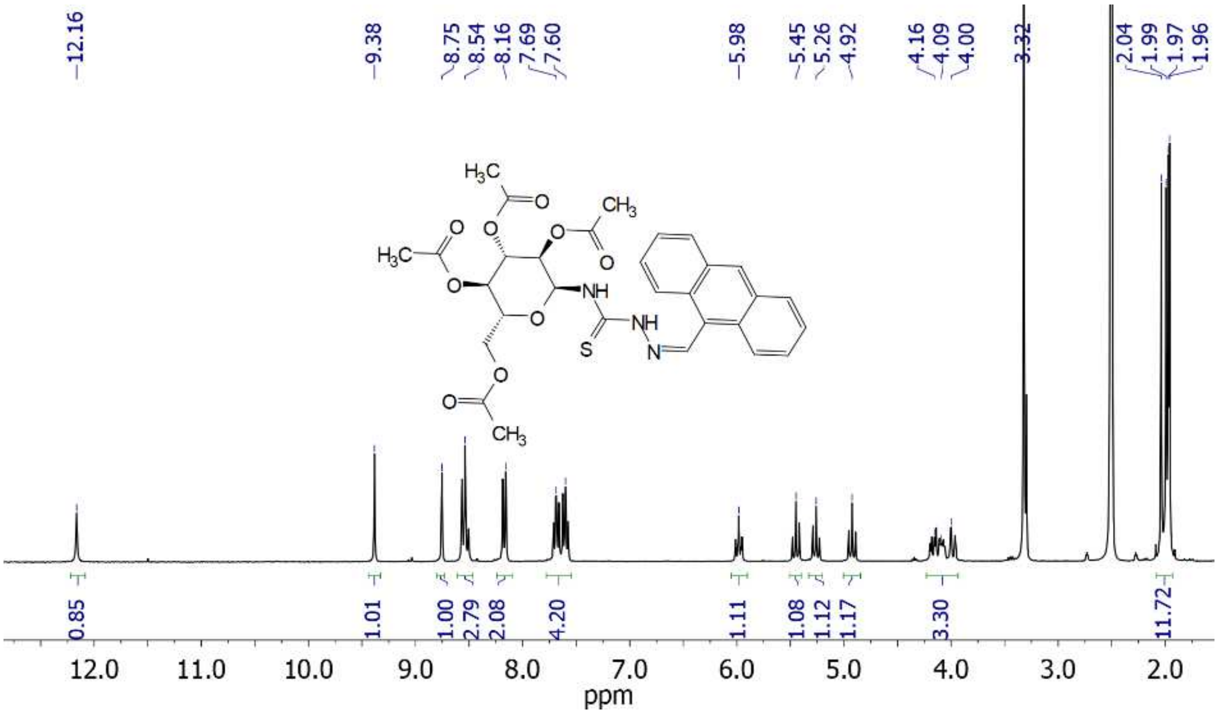

Figure S175. ^{1}H NMR spectrum of 9-anthraldehyde-4-(2,3,4,6-Tetra-*O*-acetyl-β-D-glucopyranosyl)-3-thiosemicarbazone in DMSO-*d*$_6$ at 499 MHz.

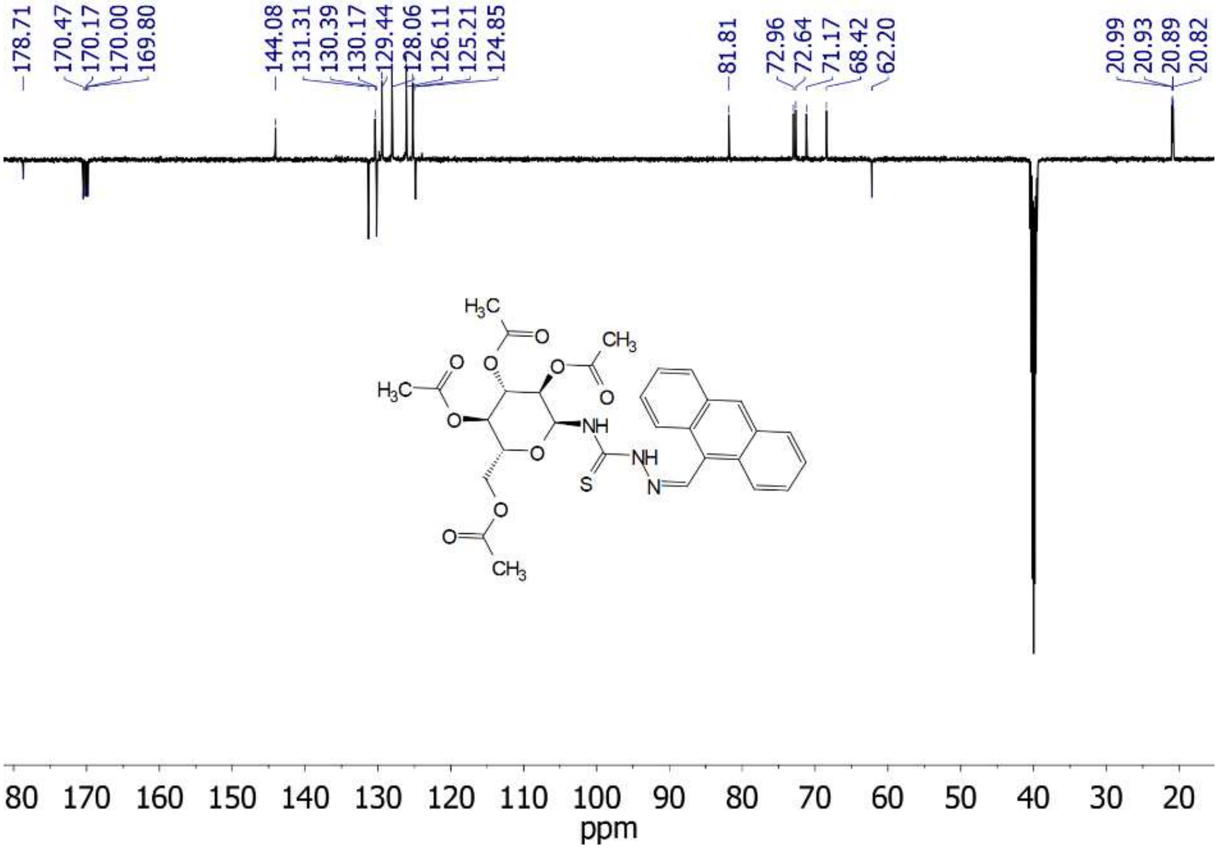

Figure S176. ^{13}C APT NMR spectrum of 9-anthraldehyde-4-(2,3,4,6-Tetra-*O*-acetyl-β-D-glucopyranosyl)-3-thiosemicarbazone in DMSO-d_6 at 499 MHz.

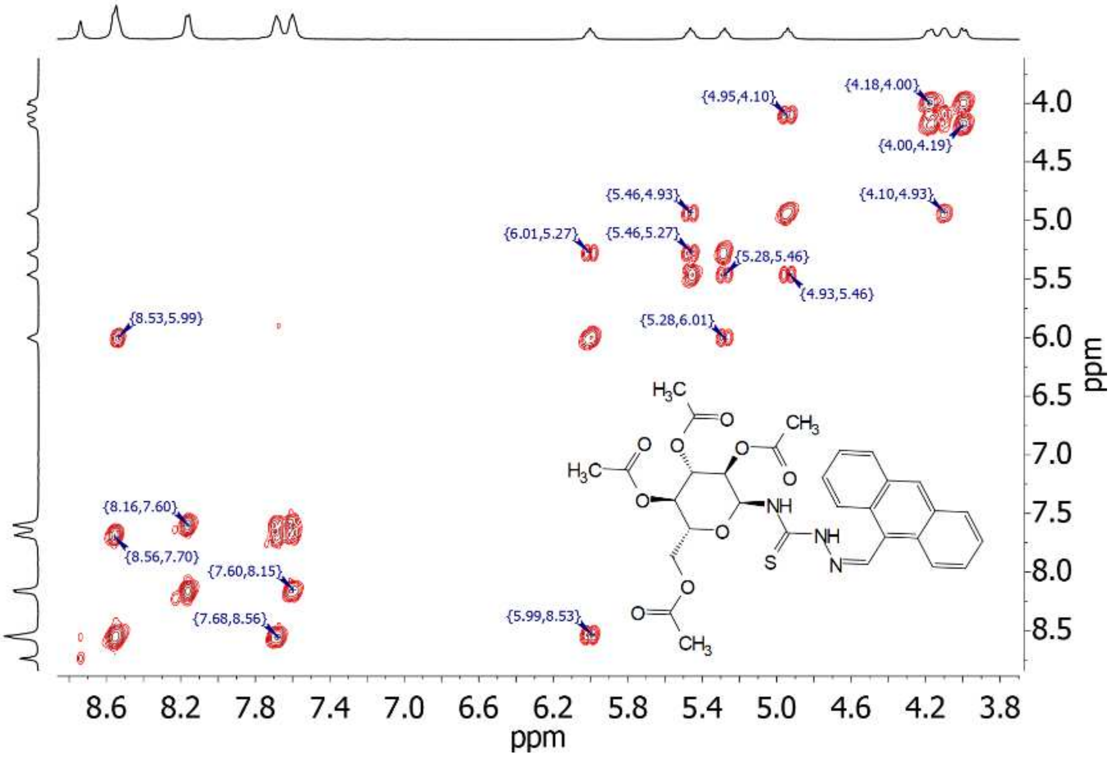

Figure S177. $^{1}H,^{1}H$ COSY NMR spectrum of 9-anthraldehyde-4-(2,3,4,6-Tetra-*O*-acetyl-β-D-glucopyranosyl)-3-thiosemicarbazone in DMSO-d_6 at 499 MHz.

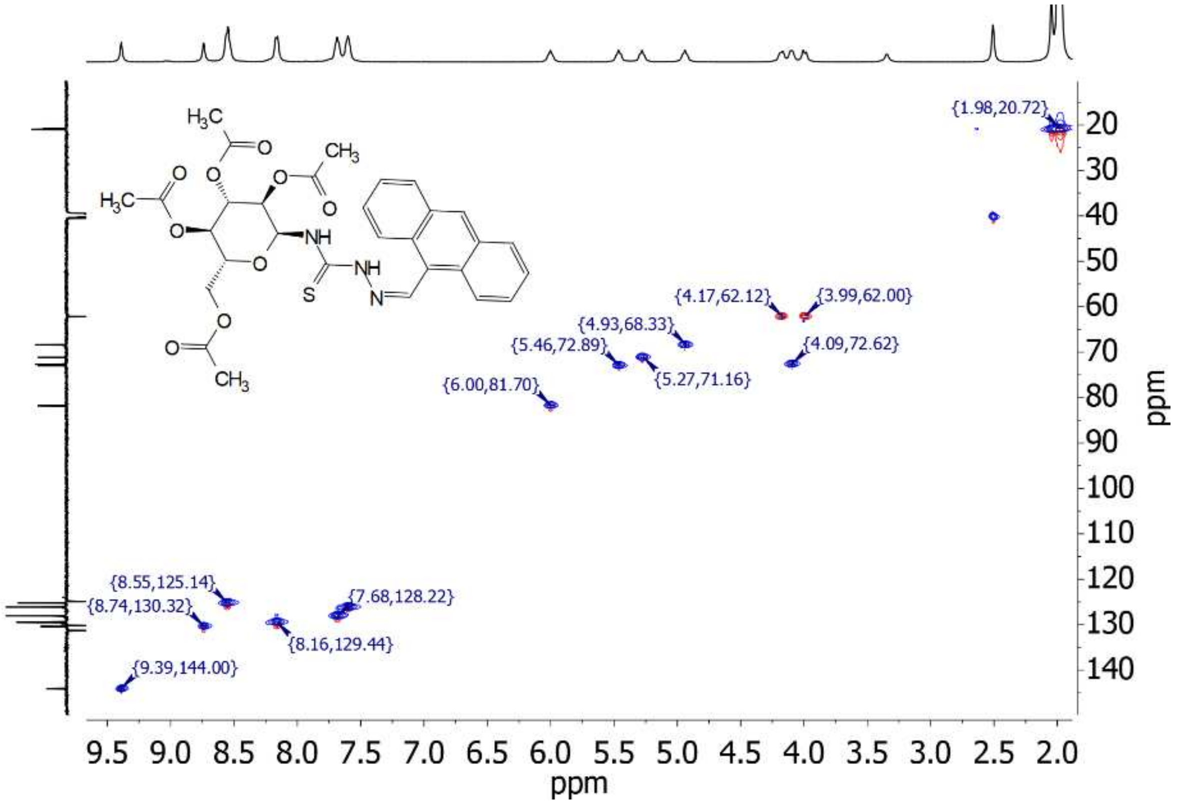

Figure S178. ^{1}H,^{13}C HMQC/HSQC NMR spectrum of 9-anthraldehyde-4-(2,3,4,6-Tetra-*O*-acetyl-β-D-glucopyranosyl)-3-thiosemicarbazone in DMSO-d_6 at 499 MHz.

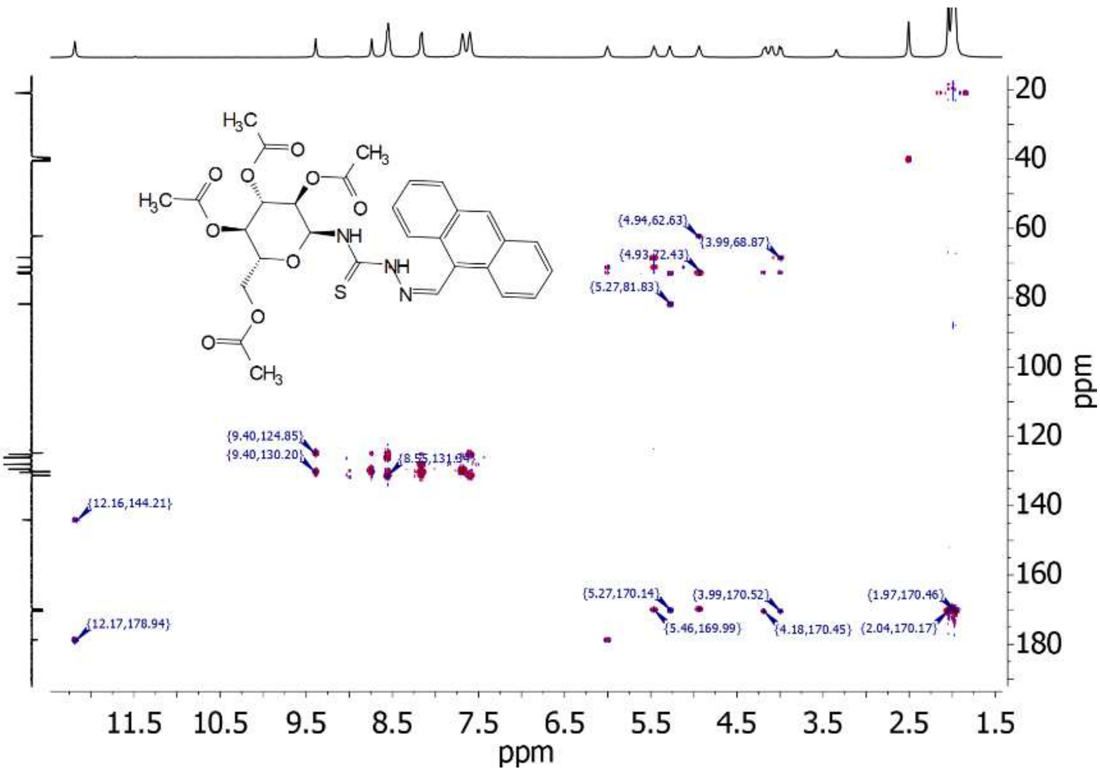

Figure S179. ^{1}H,^{13}C HMBC NMR spectrum of 9-anthraldehyde-4-(2,3,4,6-Tetra-*O*-acetyl-β-D-glucopyranosyl)-3-thiosemicarbazone in DMSO-d_6 at 499 MHz.

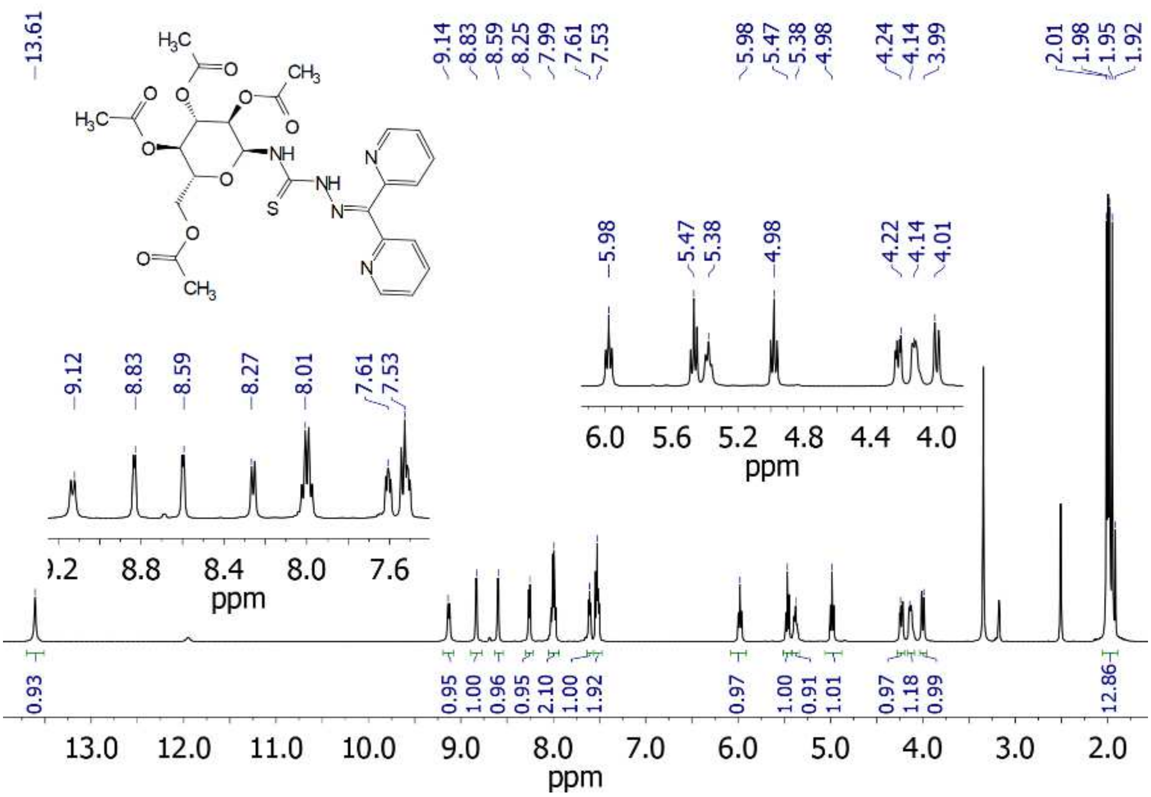

Figure S180. ^{1}H NMR spectrum of di-2-pyridylketone-4-(2,3,4,6-Tetra-*O*-acetyl-β-D-glucopyranosyl)-3-thiosemicarbazone in DMSO-d_6 at 499 MHz.

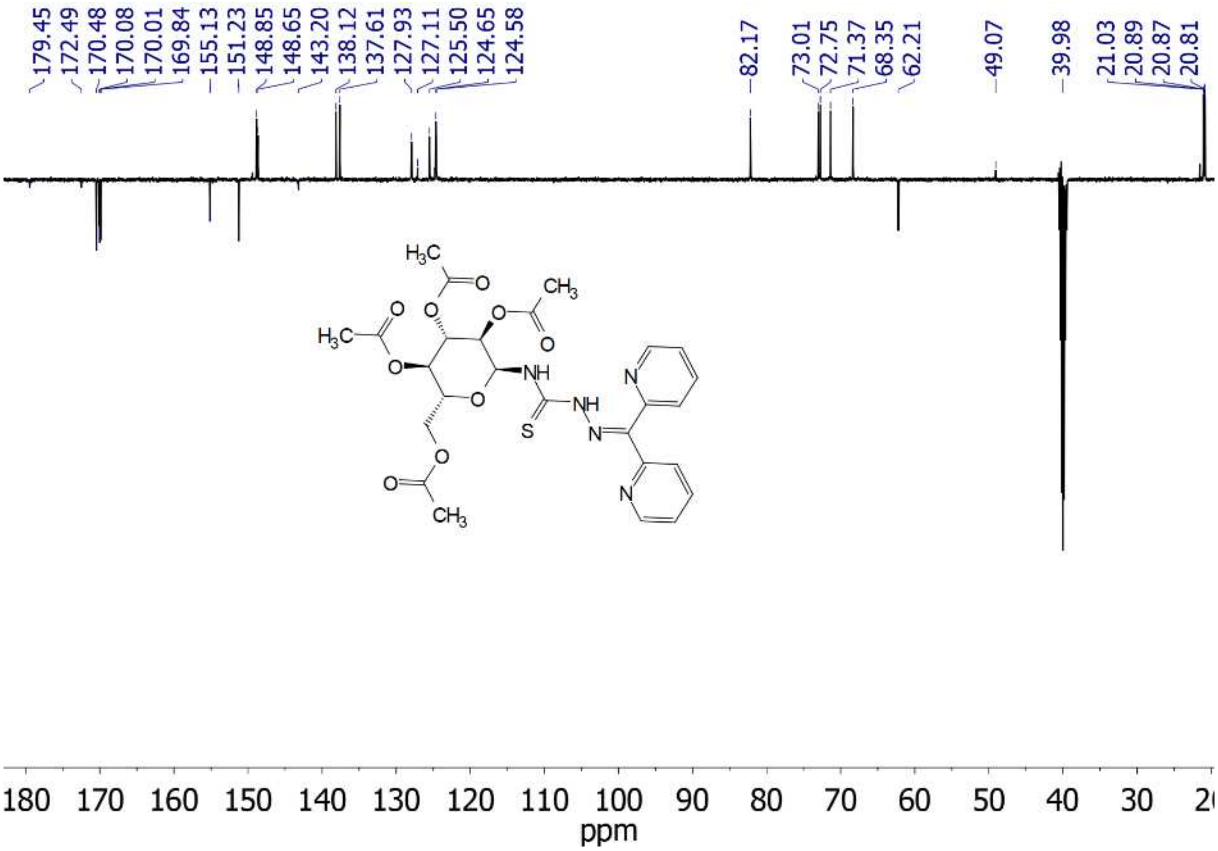

Figure S181. ^{13}C APT NMR spectrum of di-2-pyridylketone-4-(2,3,4,6-Tetra-*O*-acetyl-β-D-glucopyranosyl)-3-thiosemicarbazone in DMSO-d_6 at 499 MHz.

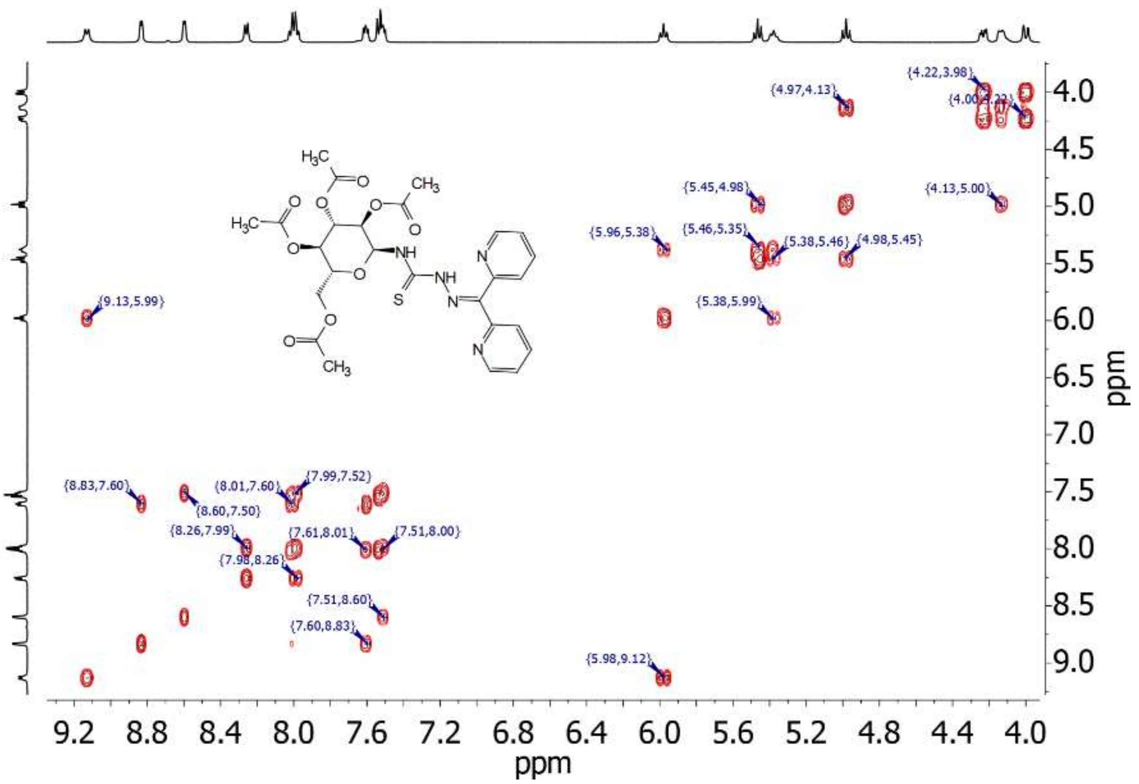

Figure S182. $^{1}H,^{1}H$ COSY NMR spectrum of di-2-pyridylketone-4-(2,3,4,6-Tetra-*O*-acetyl-β-D-glucopyranosyl)-3-thiosemicarbazone in DMSO-d_6 at 499 MHz.

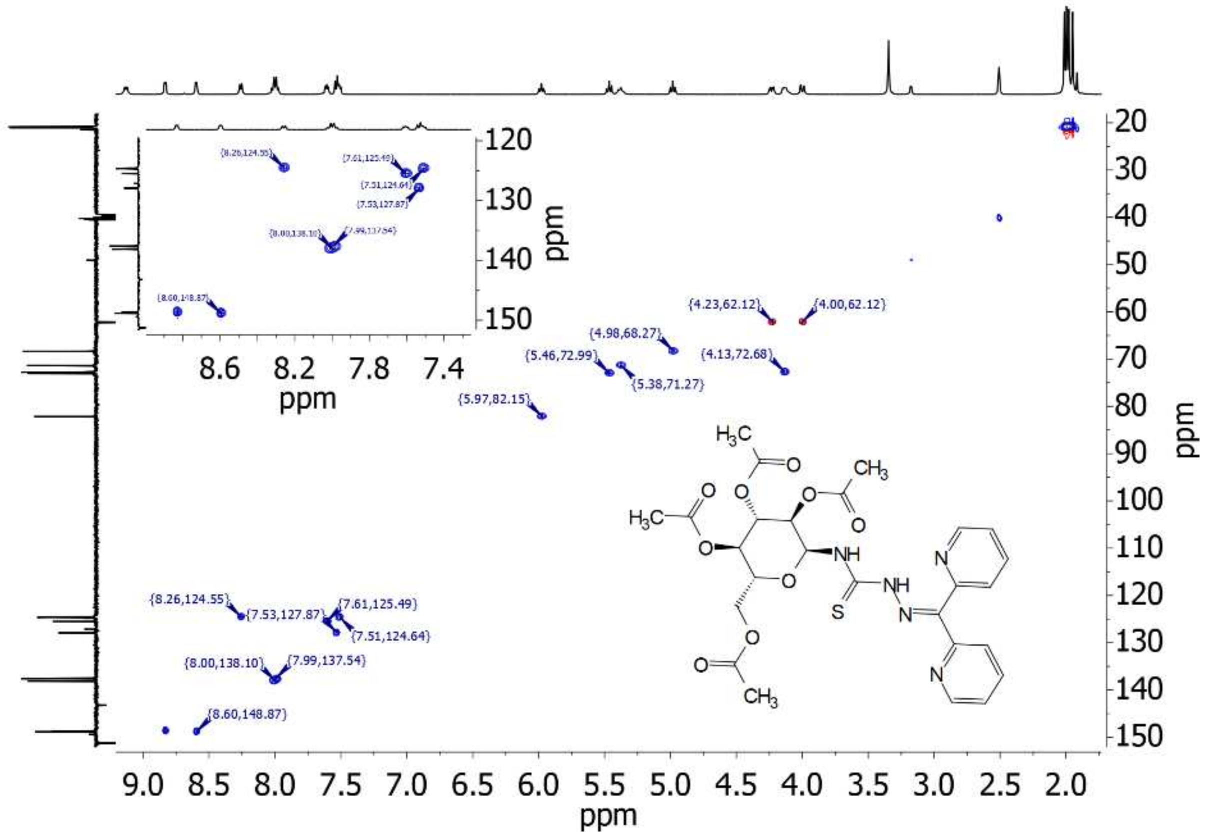

Figure S183. $^{1}H,^{13}C$ HMQC/HSQC NMR spectrum of di-2-pyridylketone-4-(2,3,4,6-Tetra-*O*-acetyl-β-D-glucopyranosyl)-3-thiosemicarbazone in DMSO-d_6 at 499 MHz.

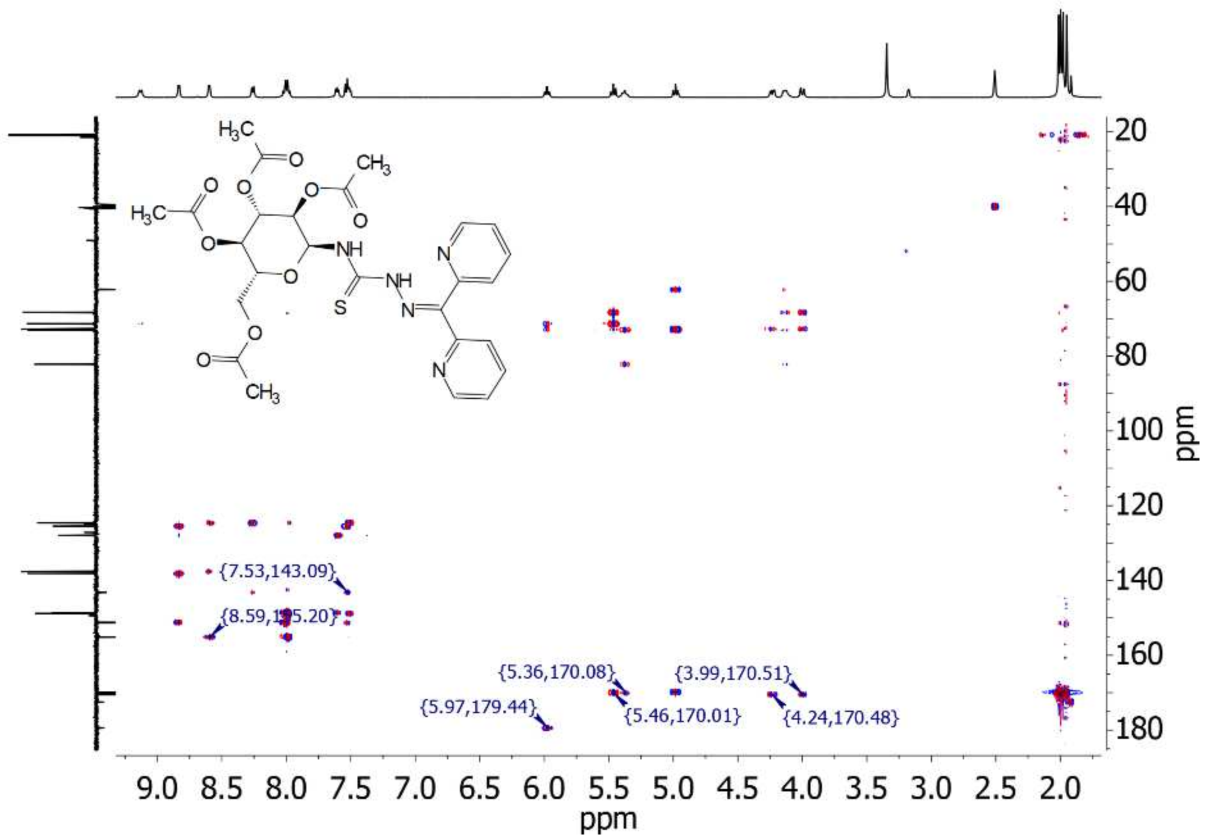

Figure S184. $^{1}H,^{13}C$ HMBC NMR spectrum of di-2-pyridylketone-4-(2,3,4,6-Tetra-*O*-acetyl-β-D-glucopyranosyl)-3-thiosemicarbazone in DMSO-d_6 at 499 MHz.

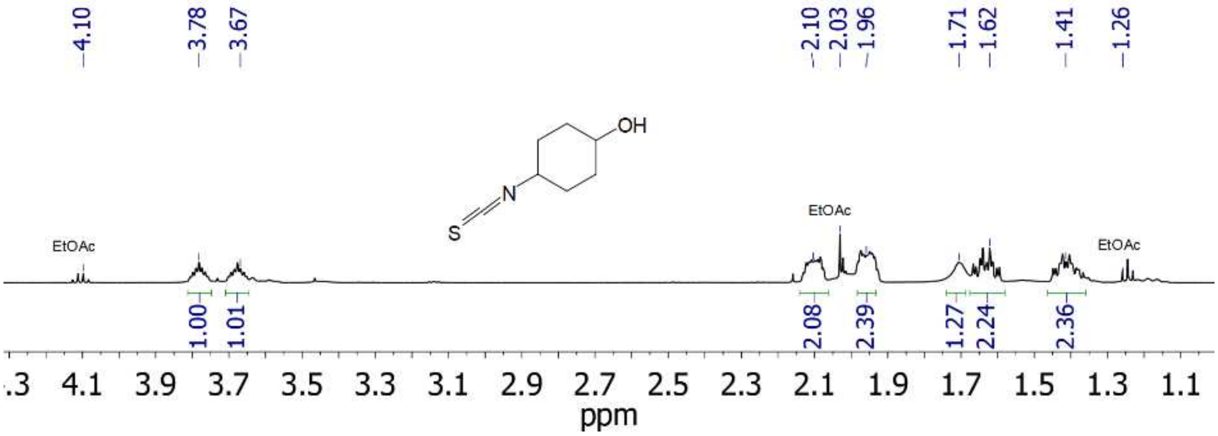

Figure S185. ^{1}H NMR spectrum of <u>*trans*-4-hydroxycyclohexyl isothiocyanate</u> in $CDCl_3$ at 499 MHz.

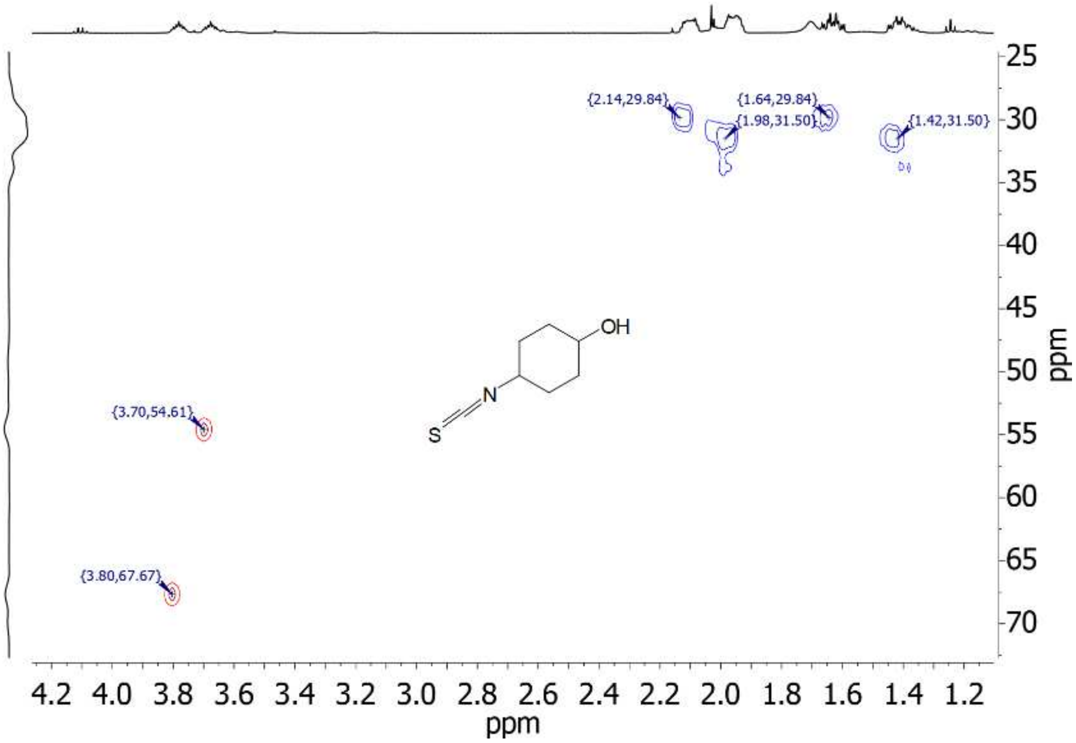

Figure S186. $^{1}H,^{13}C$ HMQC/HSQC NMR spectrum of <u>*trans*-4-hydroxycyclohexyl isothiocyanate</u> in $CDCl_3$ at 499 MHz.

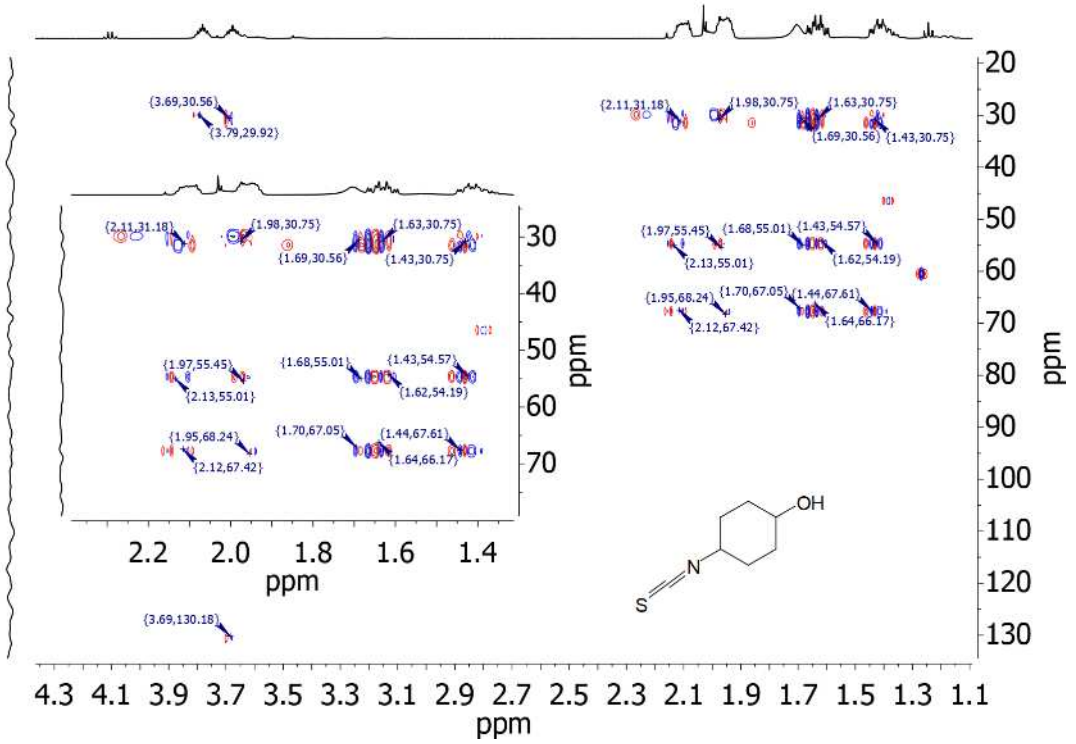

Figure S187. $^{1}H,^{13}C$ HMBC NMR spectrum of <u>*trans*-4-hydroxycyclohexyl isothiocyanate</u> in $CDCl_3$ at 499 MHz.

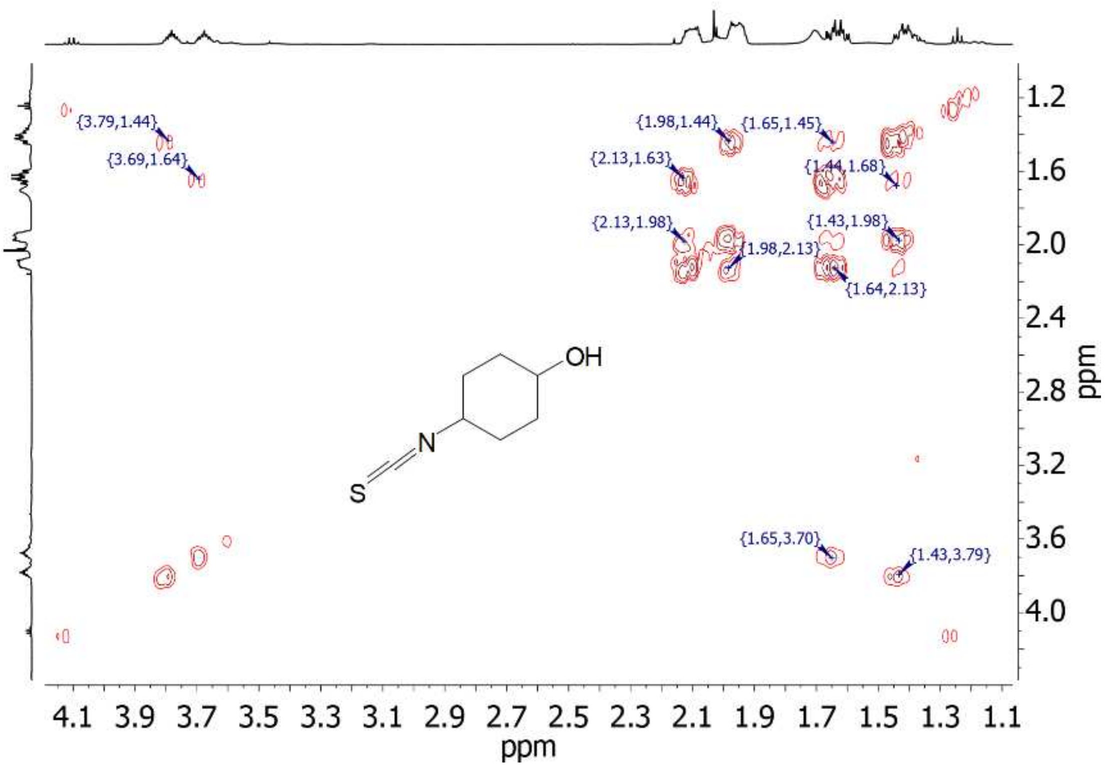

Figure S188. $^{1}H,^{1}H$ COSY NMR spectrum of <u>*trans*-4-hydroxycyclohexyl isothiocyanate</u> in $CDCl_3$ at 499 MHz.

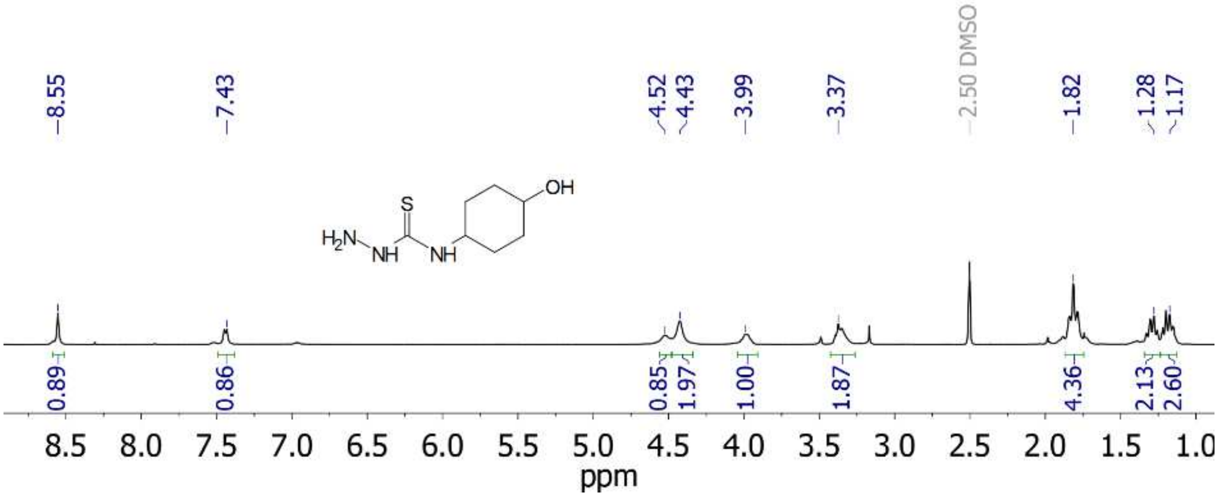

Figure S189. ^{1}H NMR spectrum of 4-(trans-4-hydroxycyclohexyl)-thiosemicarbazide in DMSO-d_6 at 499 MHz.

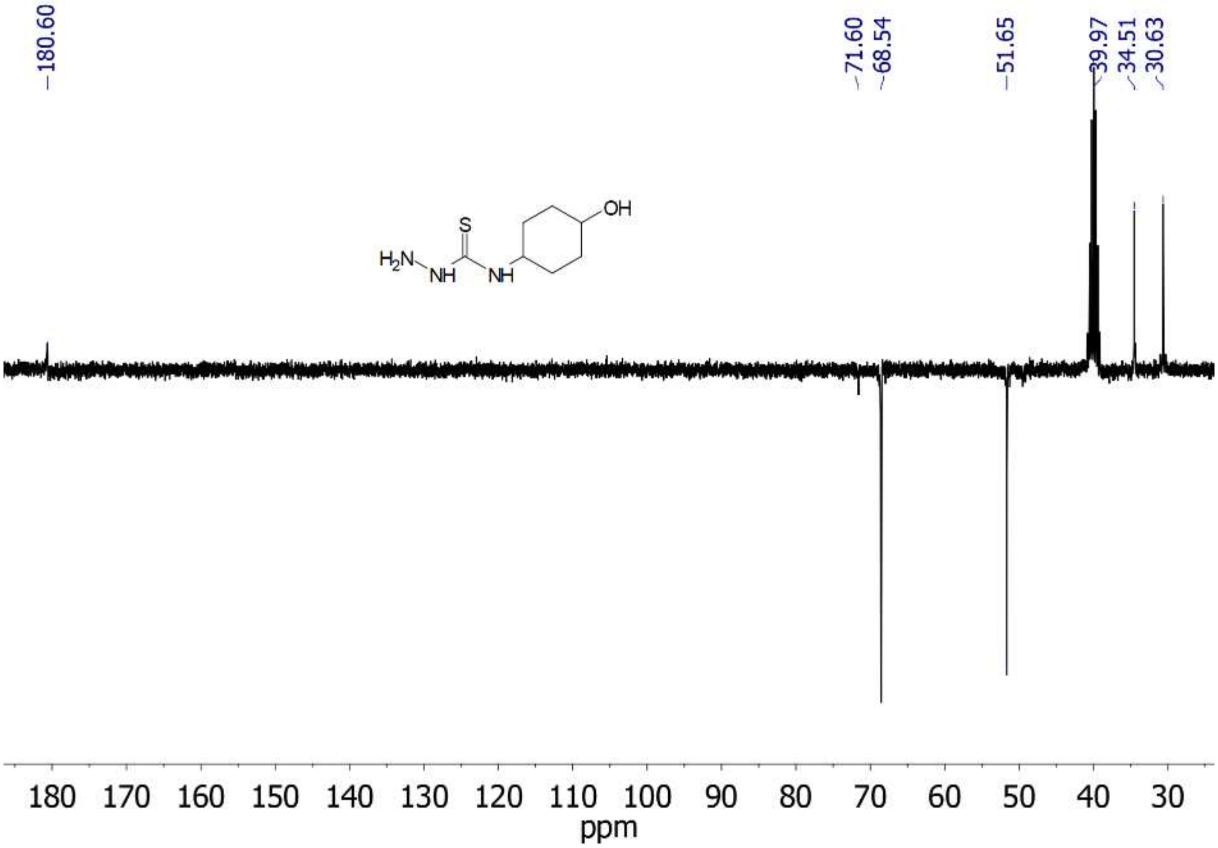

Figure S190. ^{13}C DEPTQ NMR spectrum of 4-(trans-4-hydroxycyclohexyl)-thiosemicarbazide in DMSO-d_6 at 300 MHz.

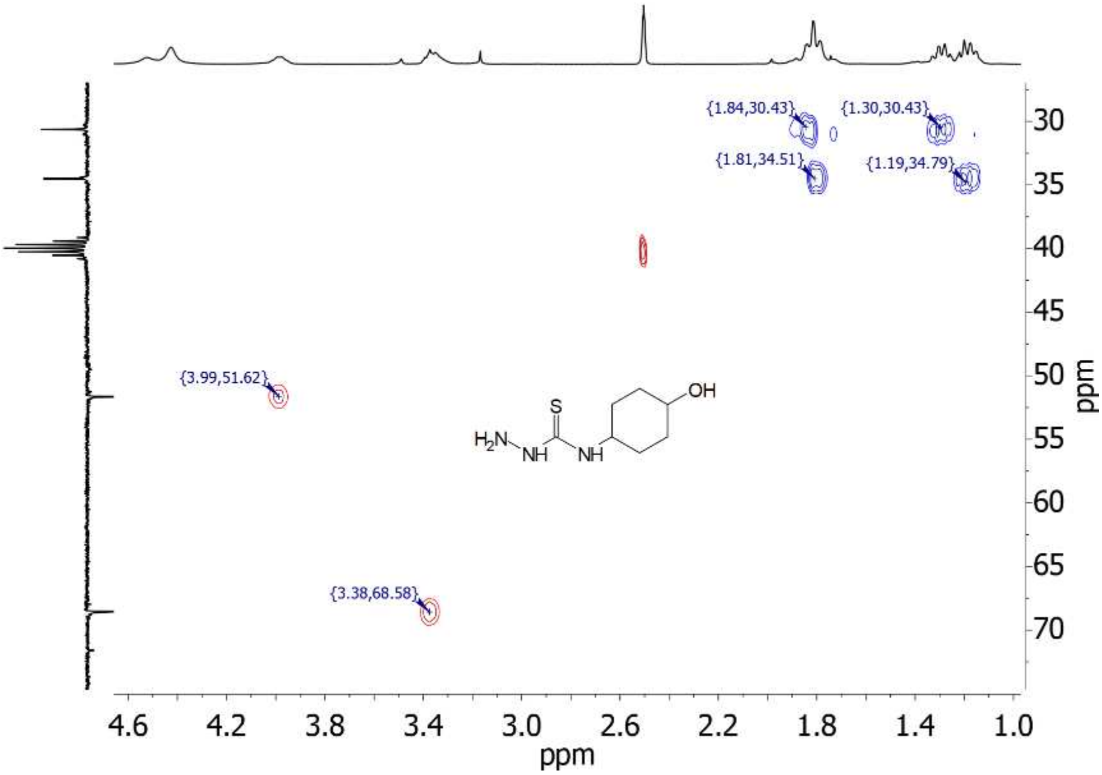

Figure S191. ^{1}H,^{13}C HMQC/HSQC NMR spectrum of 4-(trans-4-hydroxycyclohexyl)-thiosemicarbazide in DMSO-d_6 at 499 MHz.

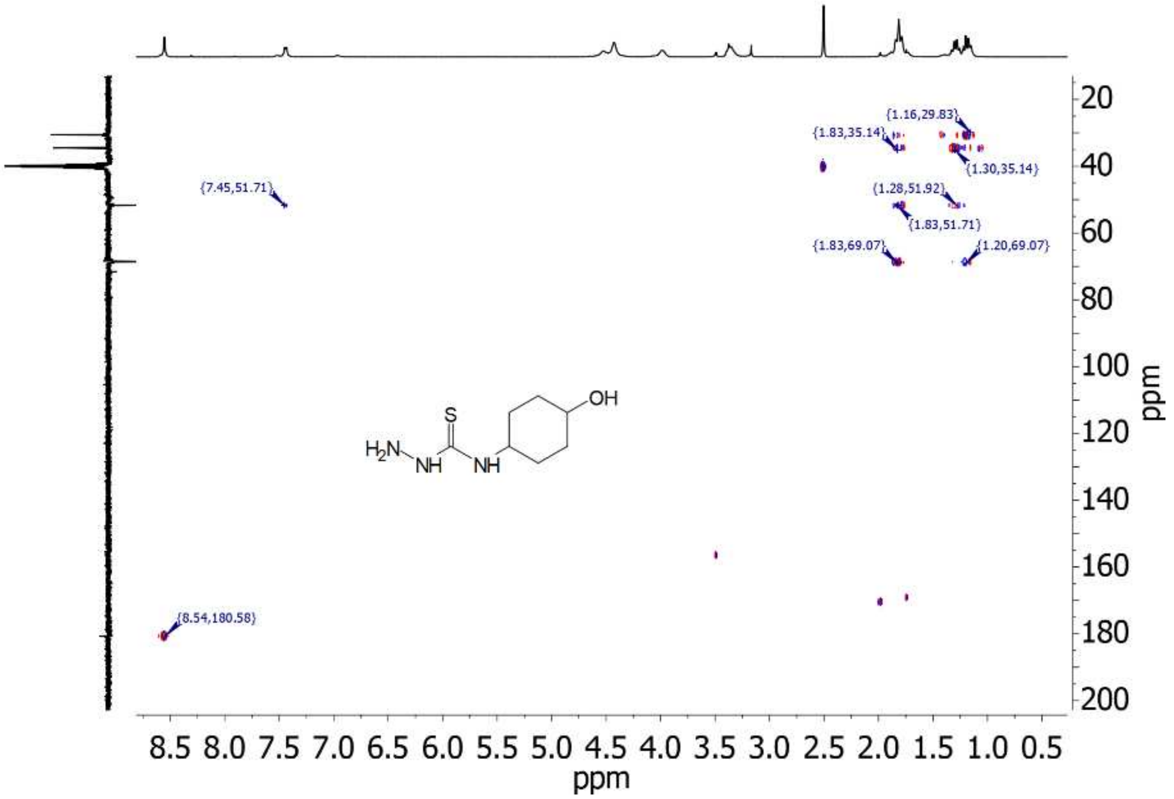

Figure S192. ^1H,^{13}C HMBC NMR spectrum of 4-(trans-4-hydroxycyclohexyl)thiosemicarbazide in DMSO-d_6 at 499 MHz.

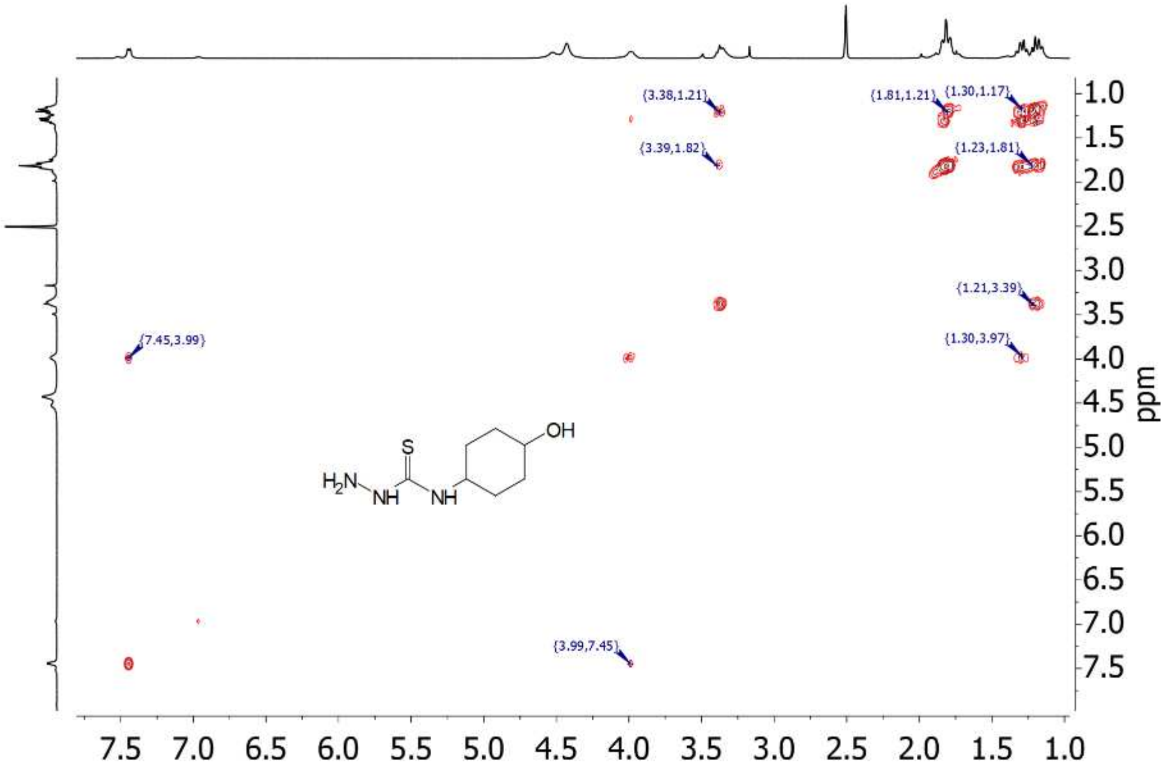

Figure S193. ^1H,^1H COSY NMR spectrum of 4-(trans-4-hydroxycyclohexyl)-thiosemicarbazide in DMSO-d_6 at 499 MHz.

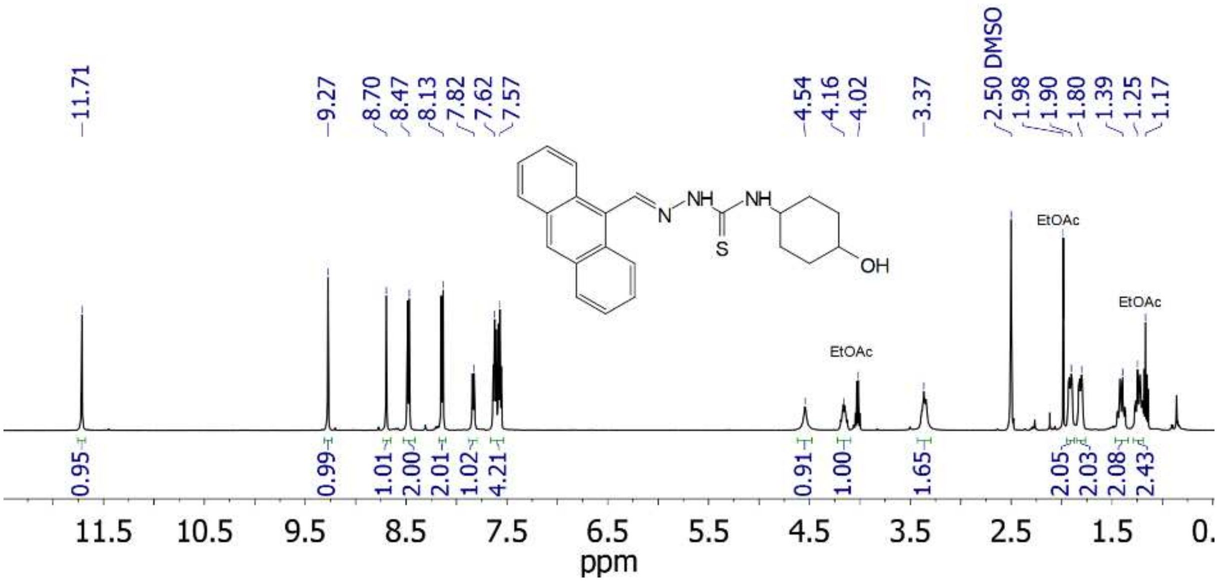

Figure S194. ^{1}H NMR spectrum of 9-anthraldehyde-4-(*trans*-4-hydroxycyclohexyl)-3-thiosemicarbazone in DMSO-d_6 at 499 MHz.

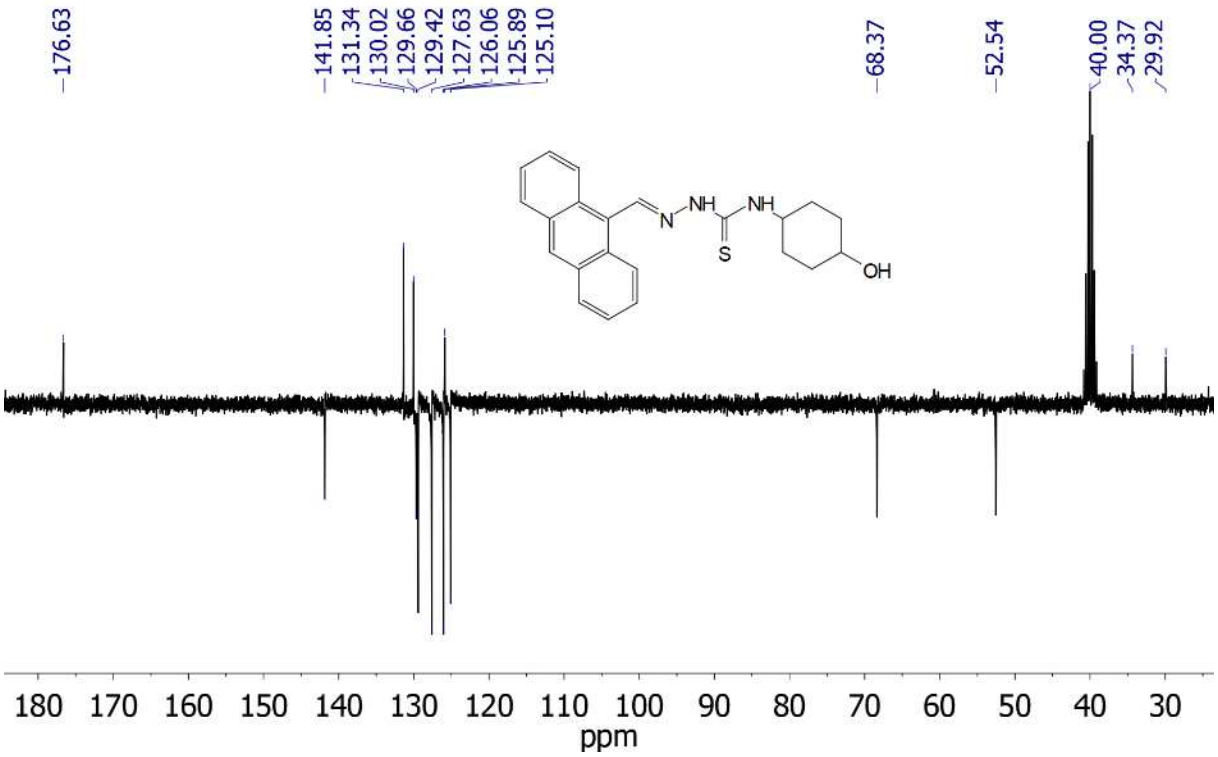

Figure S195. ^{13}C DEPTQ NMR spectrum of 9-anthraldehyde-4-(*trans*-4-hydroxycyclohexyl)-3-thiosemicarbazone in DMSO-d_6 at 300 MHz.

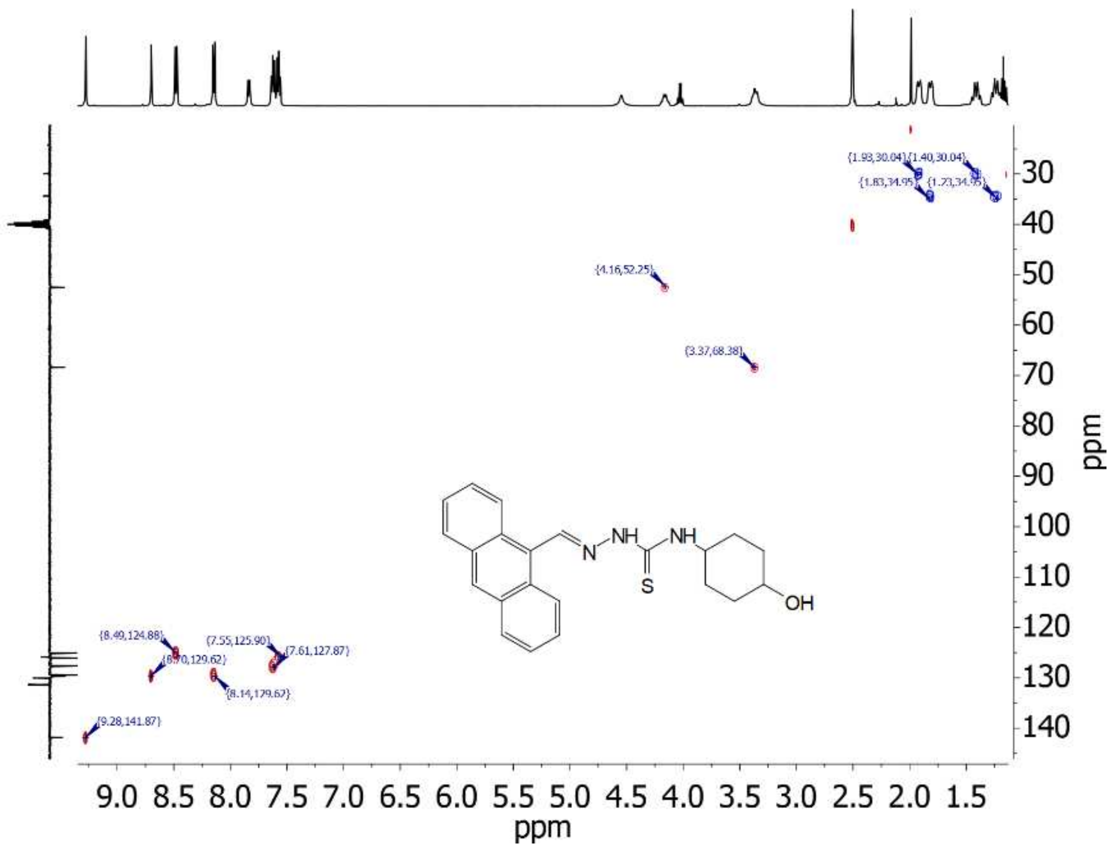

Figure S196. $^1H,^{13}C$ HMQC/HSQC NMR spectrum of 9-anthraldehyde-4-(*trans*-4-hydroxycyclohexyl)-3-thiosemicarbazone in DMSO-d_6 at 499 MHz.

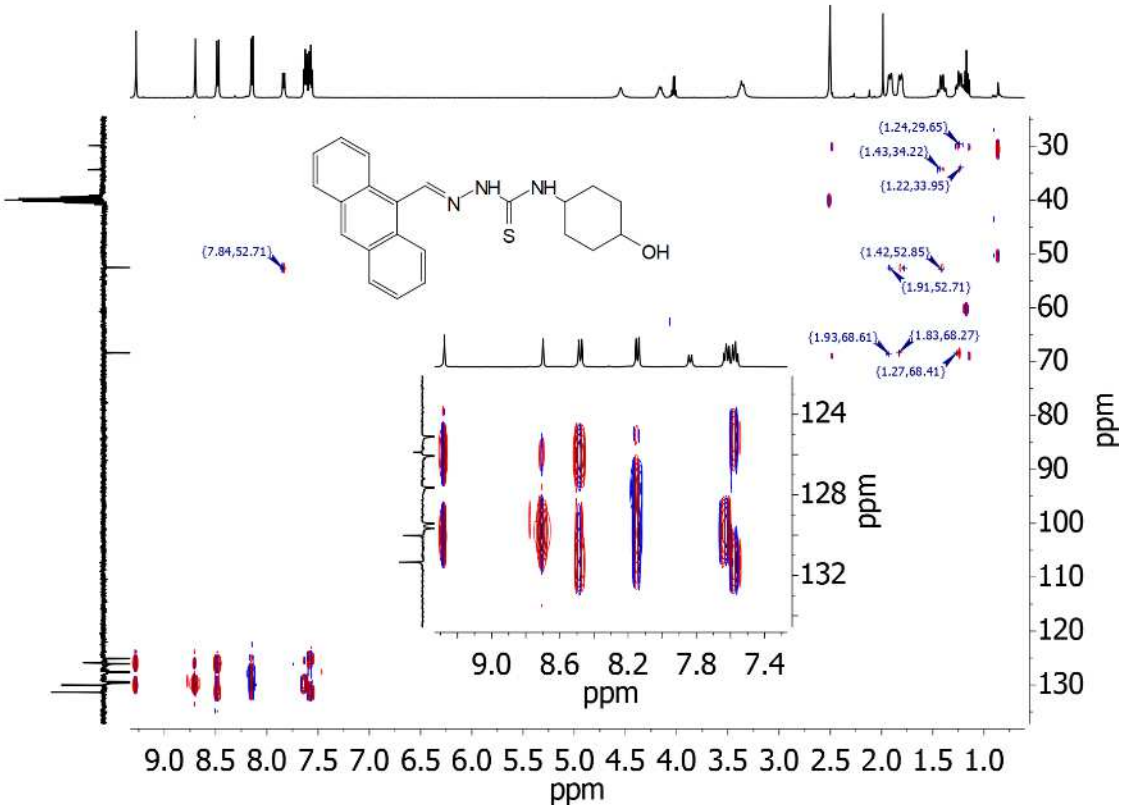

Figure S197. $^1H,^{13}C$ HMBC NMR spectrum of 9-anthraldehyde-4-(*trans*-4-hydroxycyclohexyl)-3-thiosemicarbazone in DMSO-d_6 at 499 MHz.

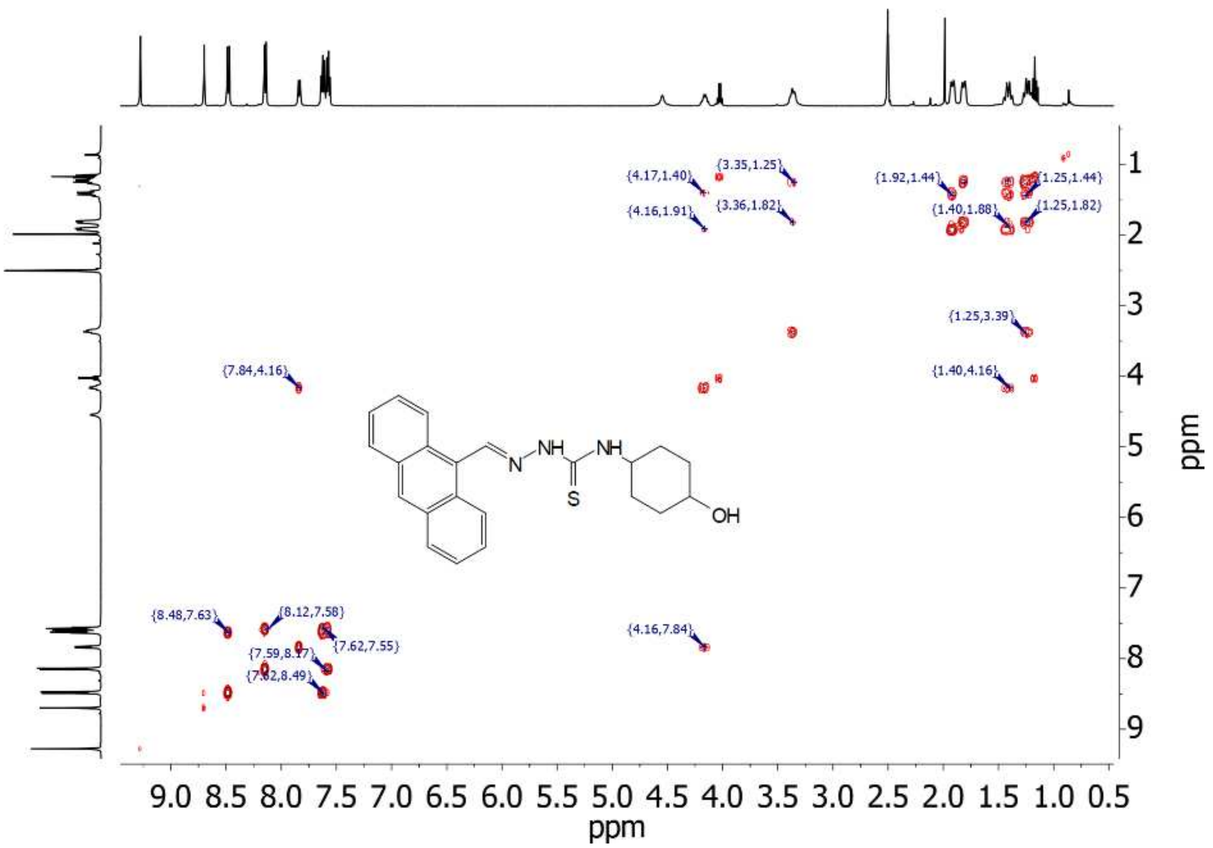

Figure S198. ^{1}H,^{1}H COSY NMR spectrum of 9-anthraldehyde-4-(*trans*-4-hydroxycyclohexyl)-3-thiosemicarbazone in DMSO-*d*$_6$ at 499 MHz.

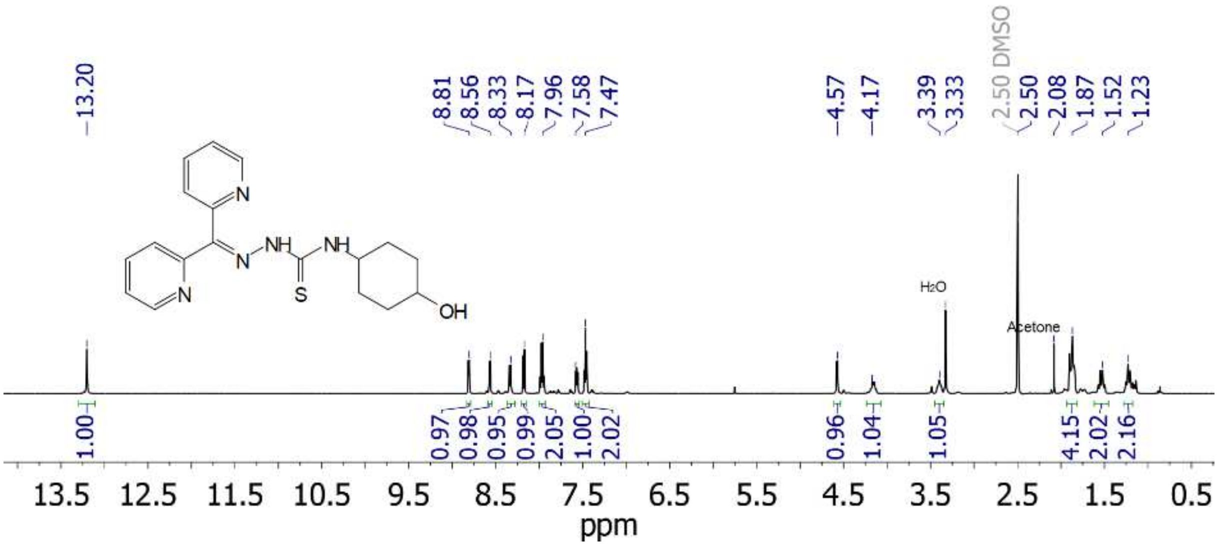

Figure S199. ^{1}H NMR spectrum of di-2-pyridylketone-4-(*trans*-4-hydroxycyclohexyl)-3-thiosemicarbazone DMSO-*d*$_6$ at 499 MHz.

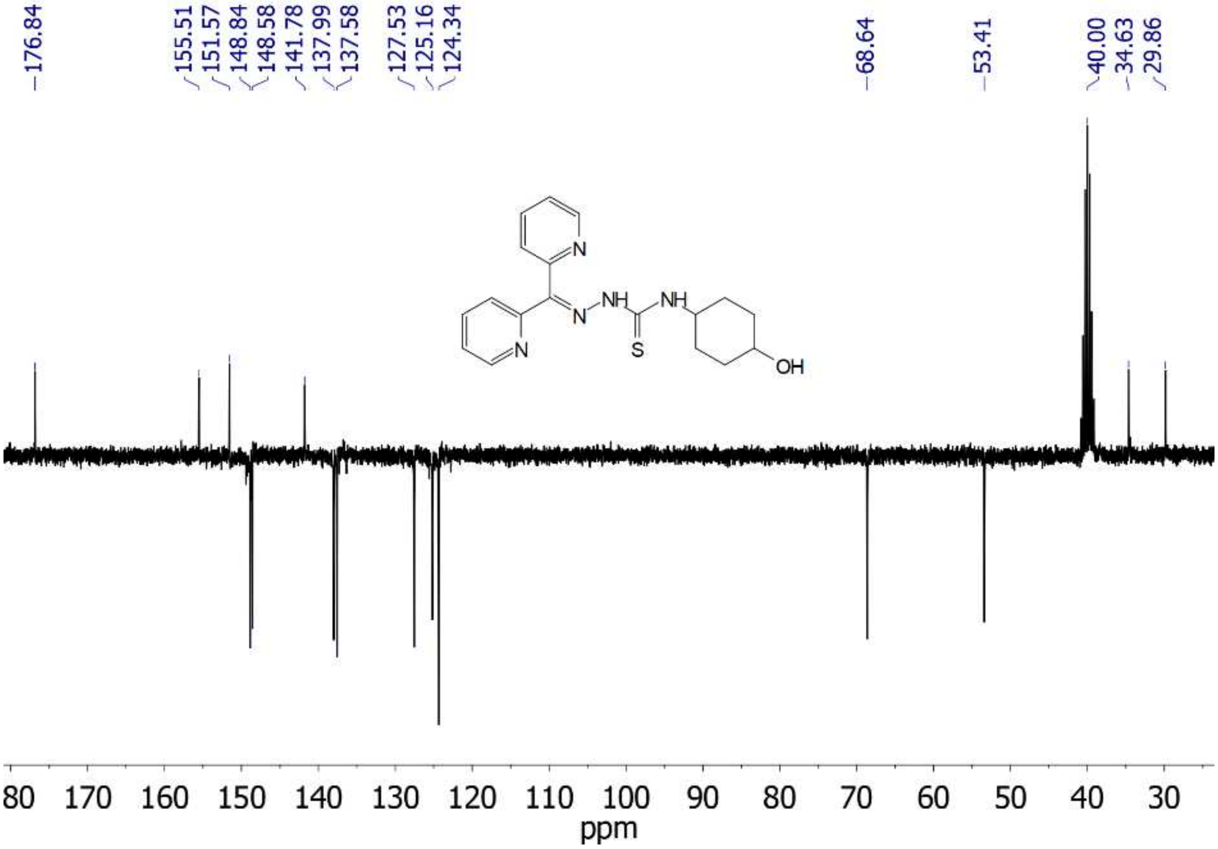

Figure S200. ^{13}C DEPTQ NMR spectrum of di-2-pyridylketone-4-(*trans*-4-hydroxycyclohexyl)-3-thiosemicarbazone in DMSO-d_6 at 300 MHz.

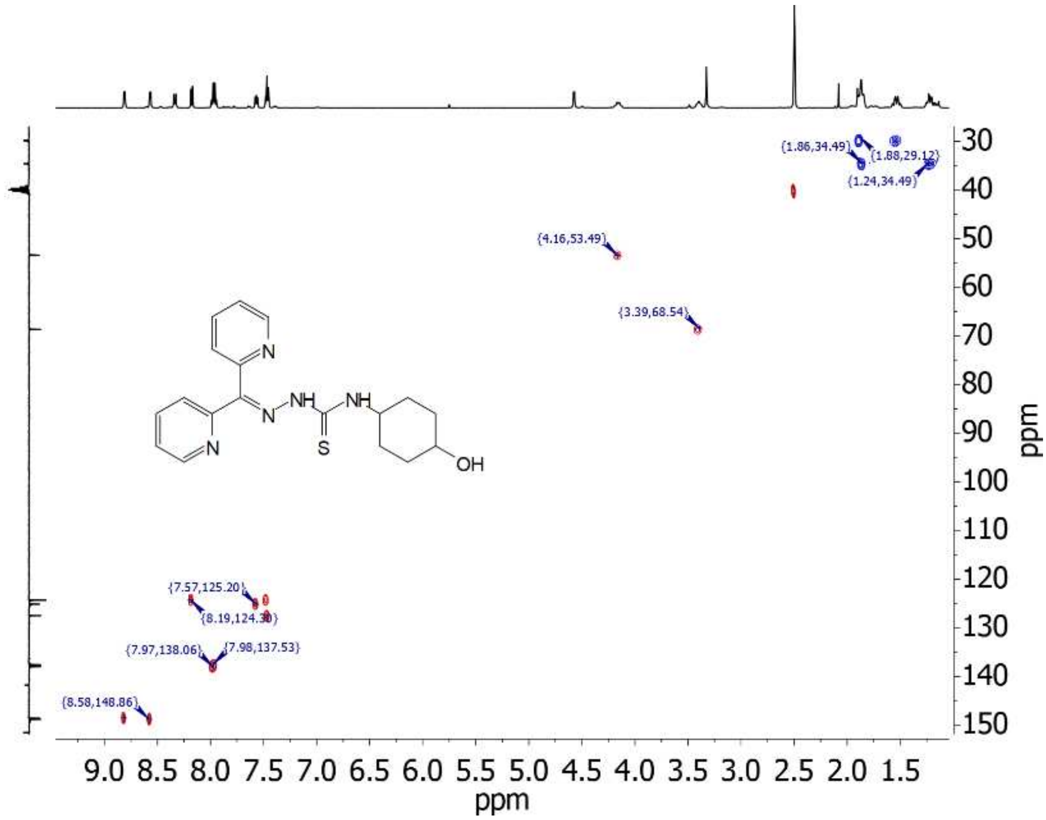

Figure S201. ^{1}H,^{13}C HMQC/HSQC NMR spectrum of di-2-pyridylketone-4-(*trans*-4-hydroxy-cyclohexyl)-3-thiosemicarbazone in DMSO-d_6 at 499 MHz.

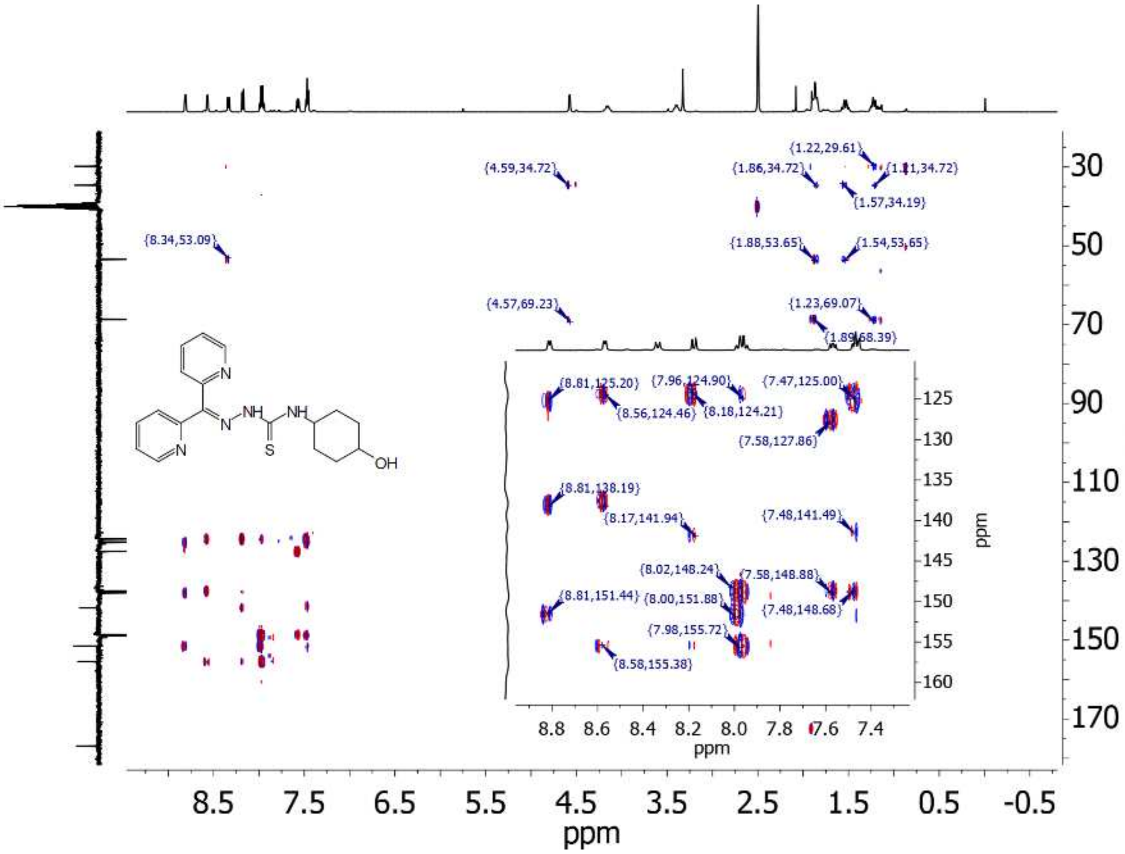

Figure S202. $^{1}H,^{13}C$ HMBC NMR spectrum of di-2-pyridylketone-4-(*trans*-4-hydroxycyclohexyl)-3-thiosemicarbazone in DMSO-d_6 at 499 MHz.

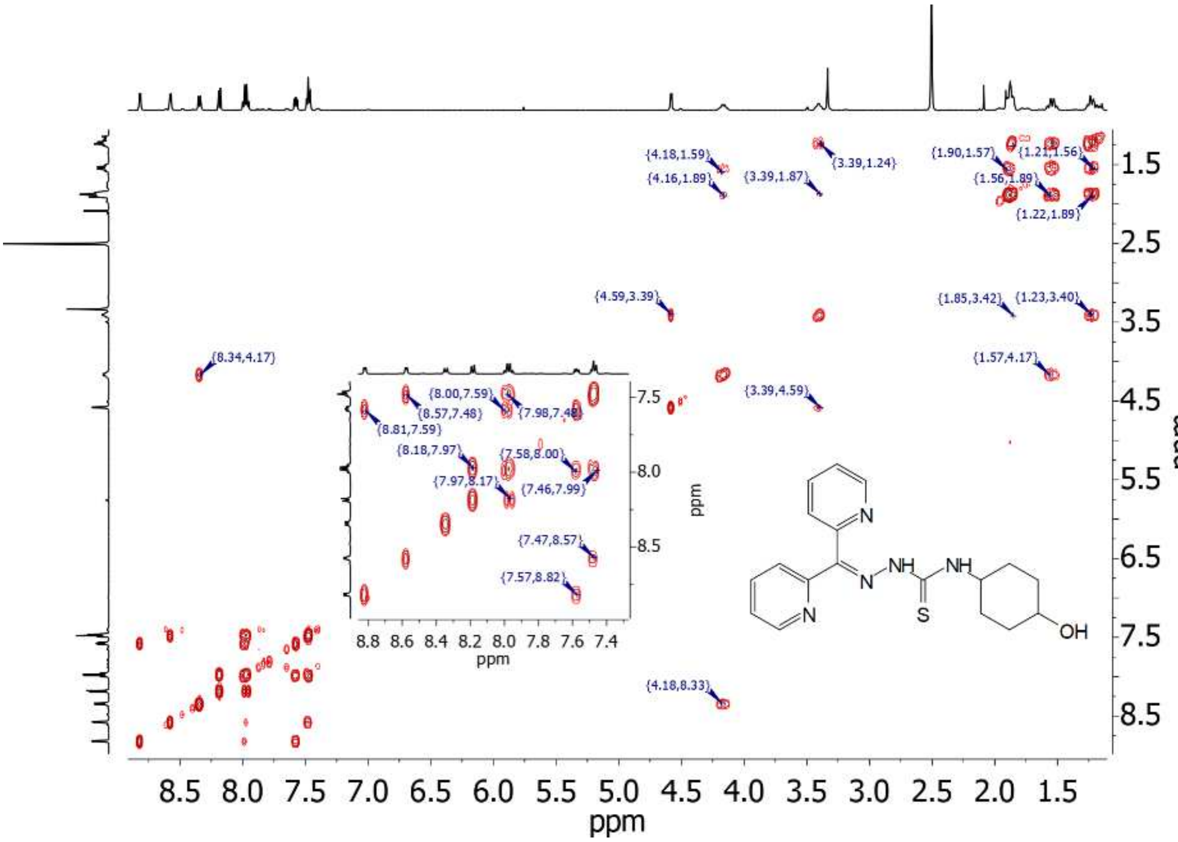

Figure S203. $^{1}H,^{1}H$ COSY NMR spectrum of di-2-pyridylketone-4-(*trans*-4-hydroxycyclohexyl)-3-thiosemicarbazone in DMSO-d_6 at 499 MHz.

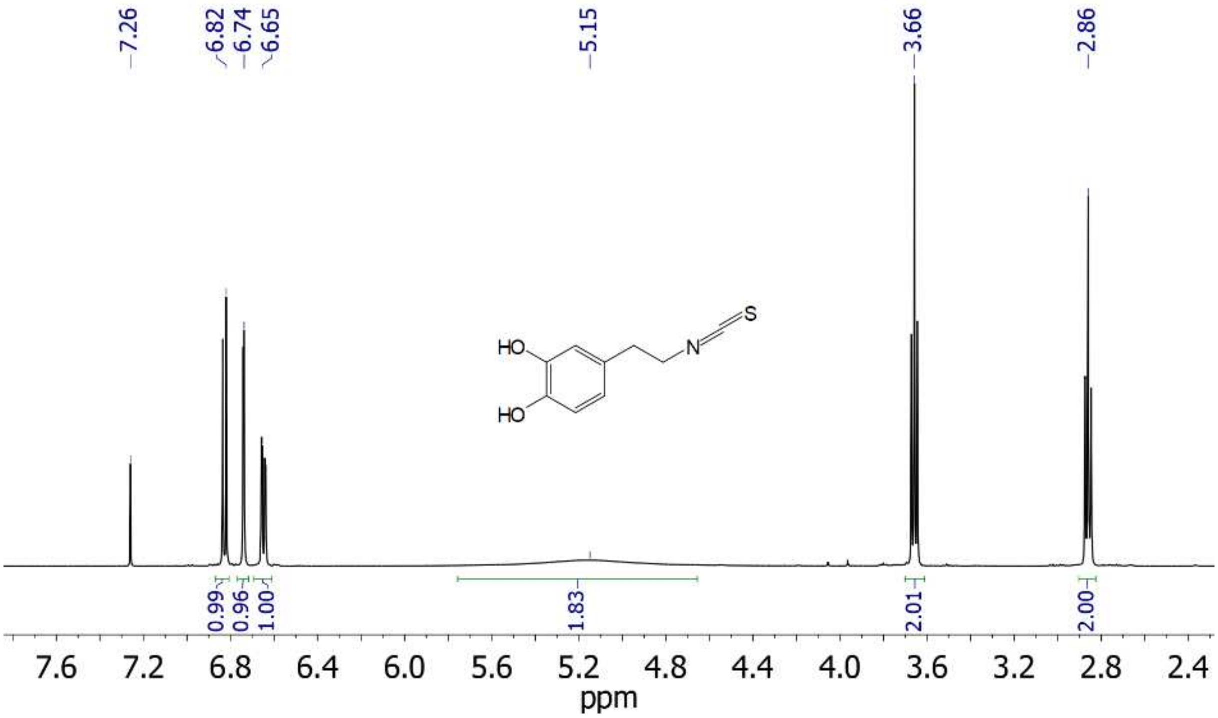

Figure S204. ^{1}H NMR spectrum of 4-(2-isothiocyanatoethyl)benzene-1,2-diol in $CDCl_3$ at 499 MHz.

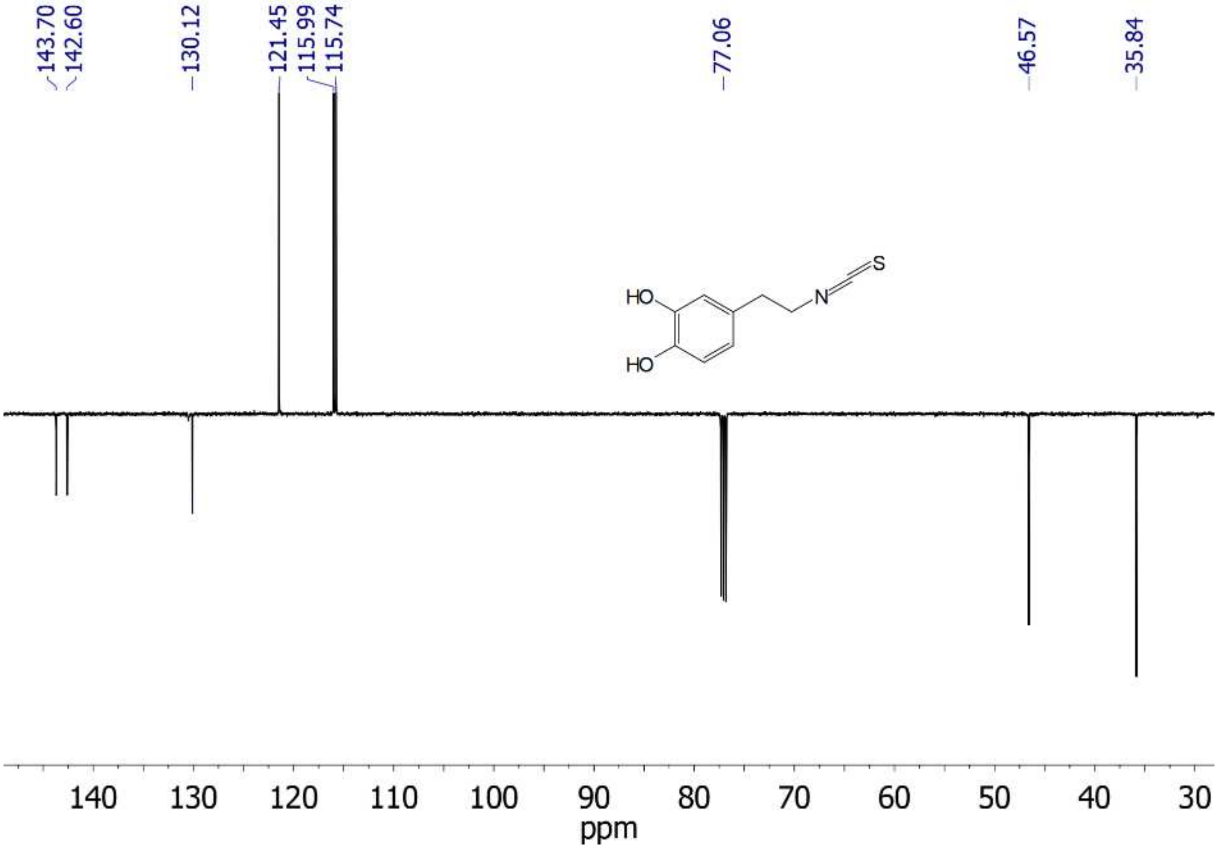

Figure S205. ^{13}C APT NMR spectrum of 4-(2-isothiocyanatoethyl)benzene-1,2-diol in $CDCl_3$ at 499 MHz.

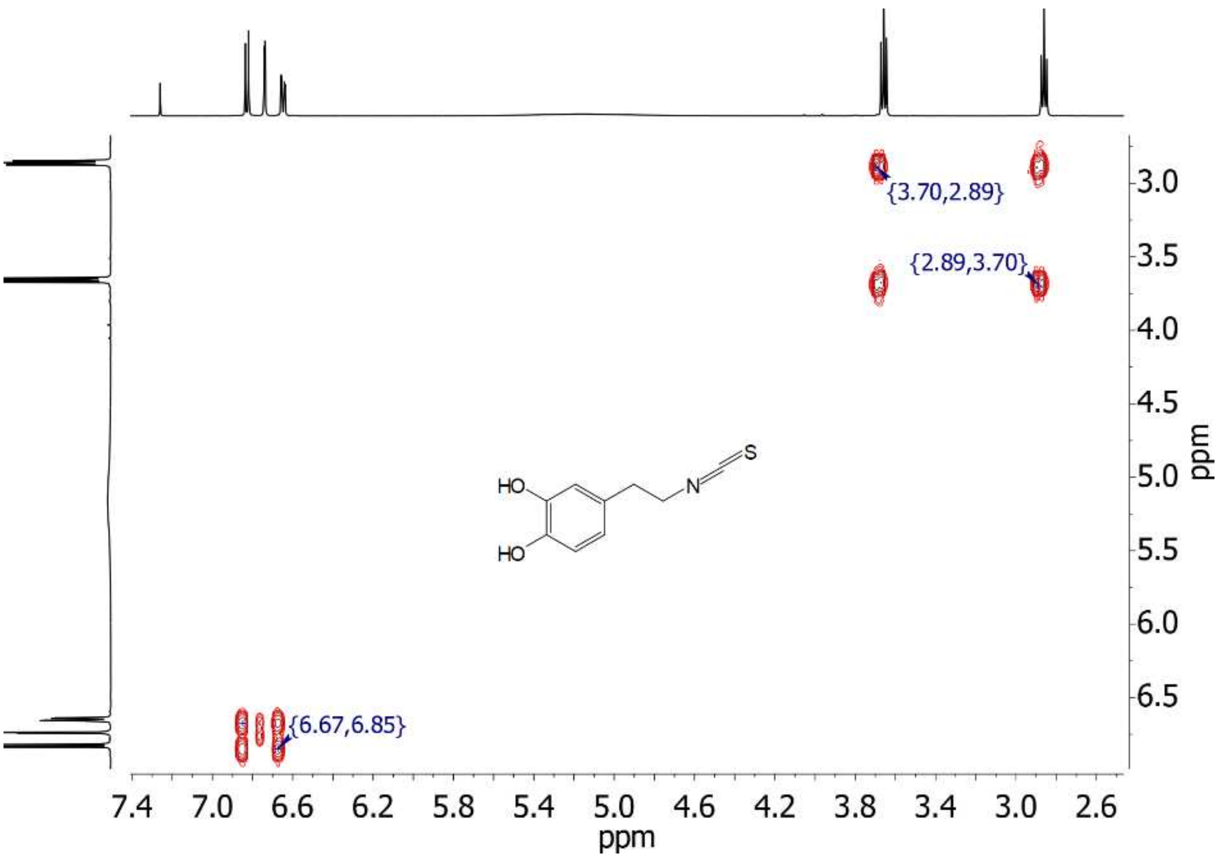

Figure S206. $^{1}H,^{1}H$ COSY NMR spectrum of 4-(2-isothiocyanatoethyl)benzene-1,2-diol in $CDCl_3$ at 499 MHz.

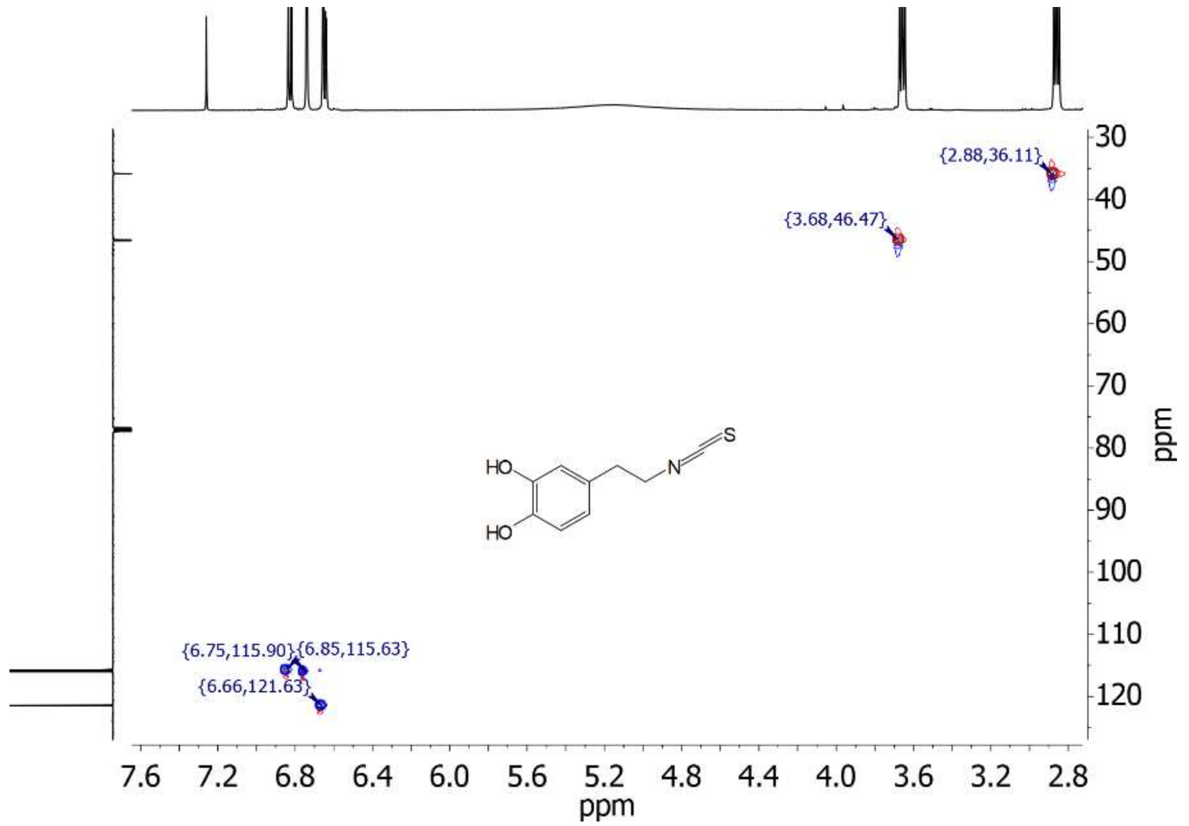

Figure S207. $^{1}H,^{13}C$ HMQC/HSQC NMR spectrum of 4-(2-isothiocyanatoethyl)benzene-1,2-diol in $CDCl_3$ at 499 MHz.

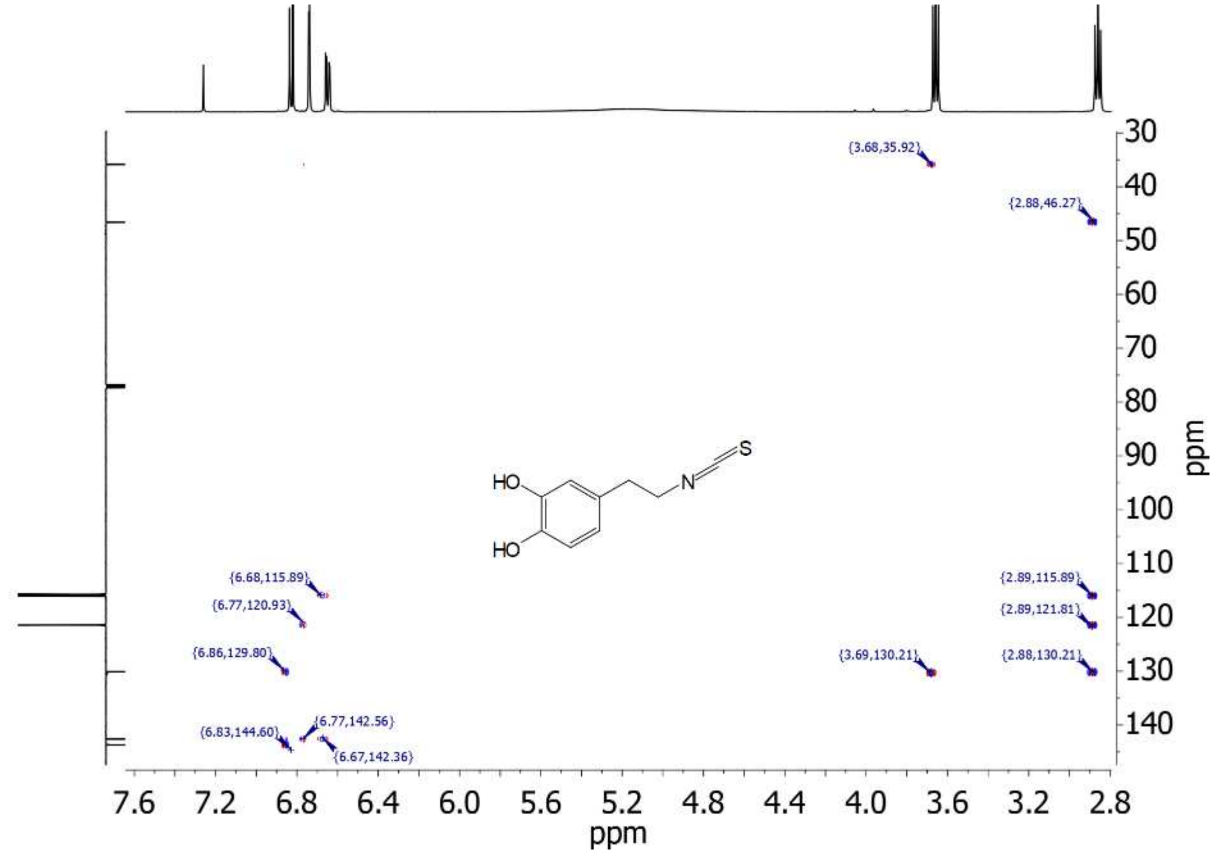

Figure S208. ^{1}H,^{13}C HMBC NMR spectrum of 4-(2-isothiocyanatoethyl)benzene-1,2-diol in $CDCl_3$ at 499 MHz.

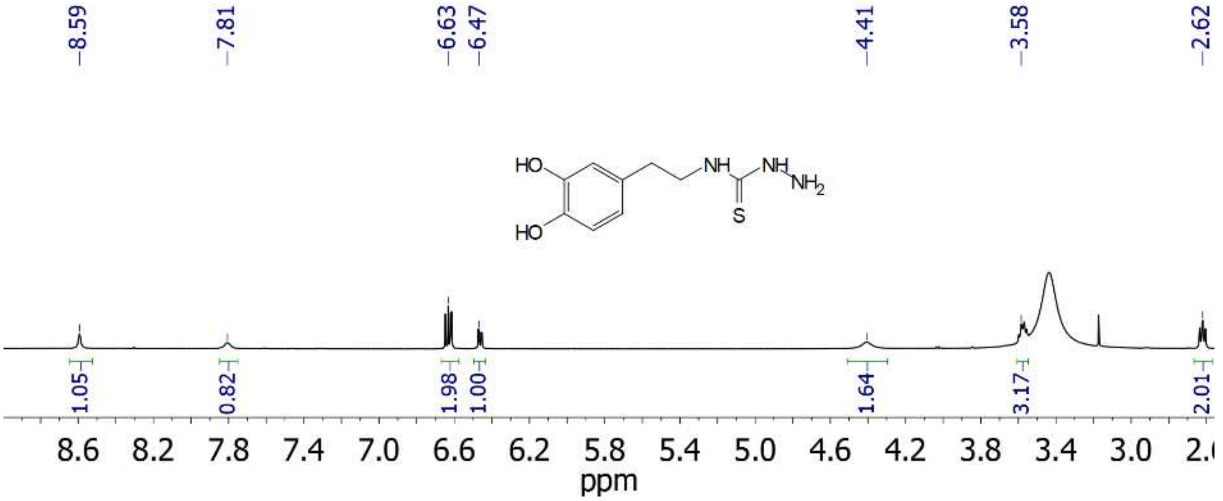

Figure S209. ^{1}H NMR spectrum of 4-[2-(hydrazidecarbothioamino)ethyl]benzene-1,2-diol in DMSO-d_6 at 499 MHz.

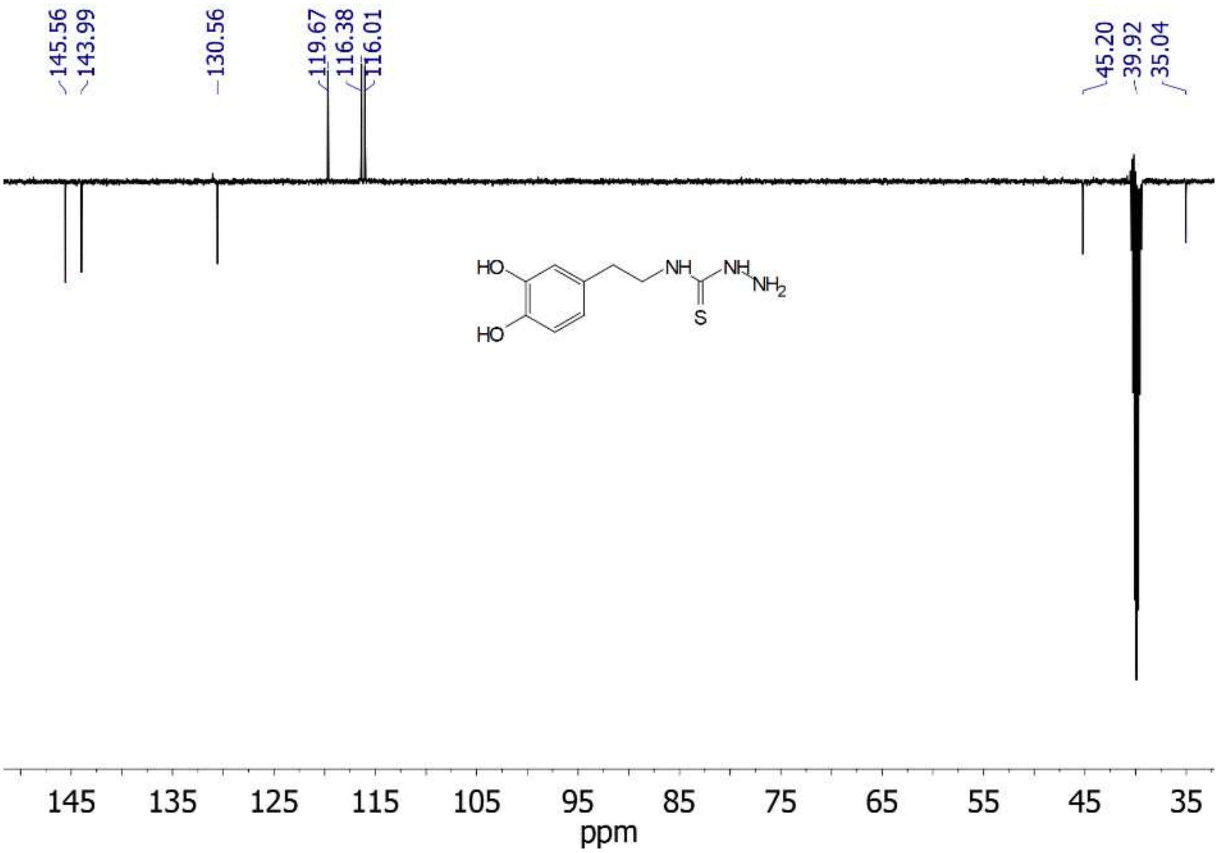

Figure S210. ^{13}C APT NMR spectrum of 4-[2-(hydrazidecarbothioamino)ethyl]benzene-1,2-diol in DMSO-d_6 at 499 MHz.

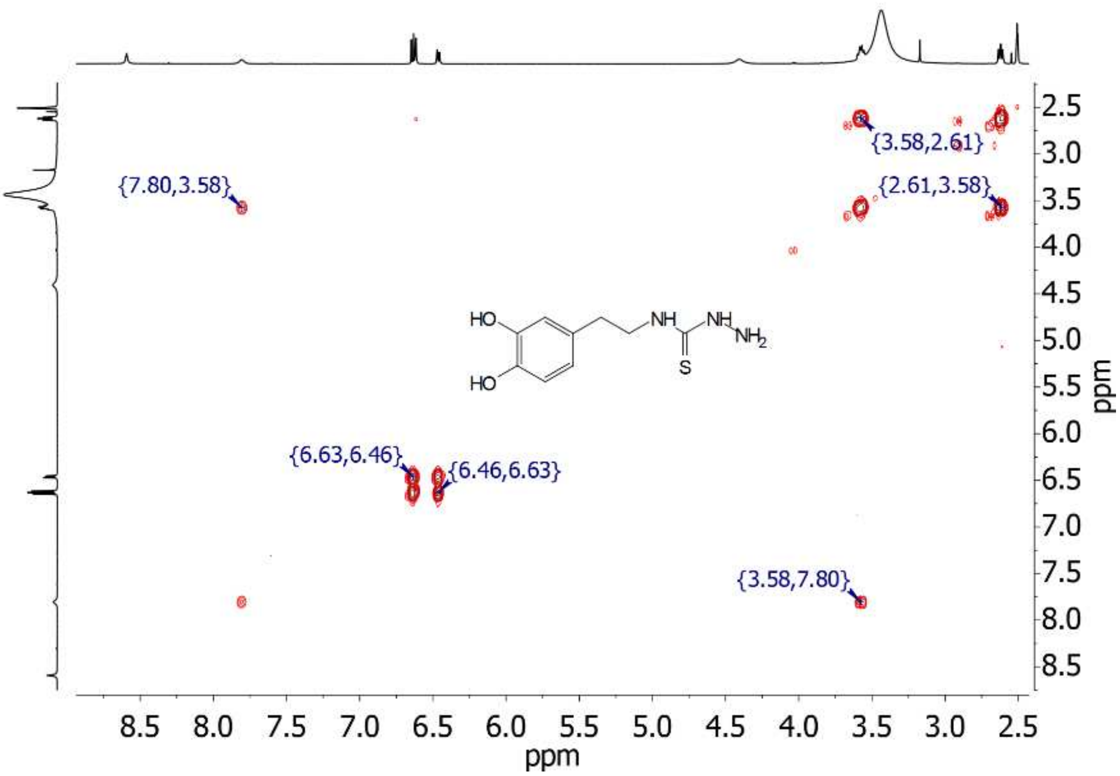

Figure S211. ^{1}H,^{1}H COSY NMR spectrum of 4-[2-(hydrazidecarbothioamino)ethyl]benzene-1,2-diol in DMSO-d_6 at 499 MHz.

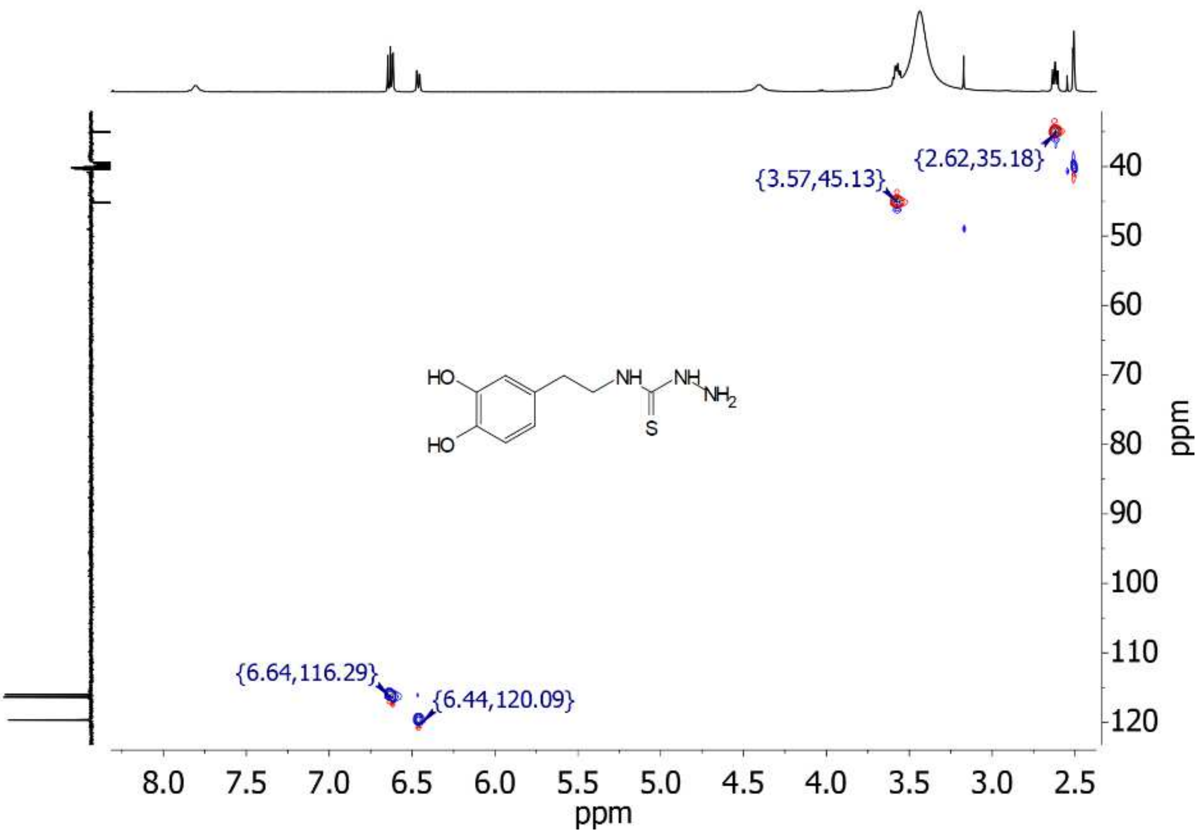

Figure S212. ^{1}H,^{13}C HMQC/HSQC NMR spectrum of 4-[2-(hydrazidecarbothioamino)ethyl]benzene-1,2-diol in DMSO-d_6 at 499 MHz.

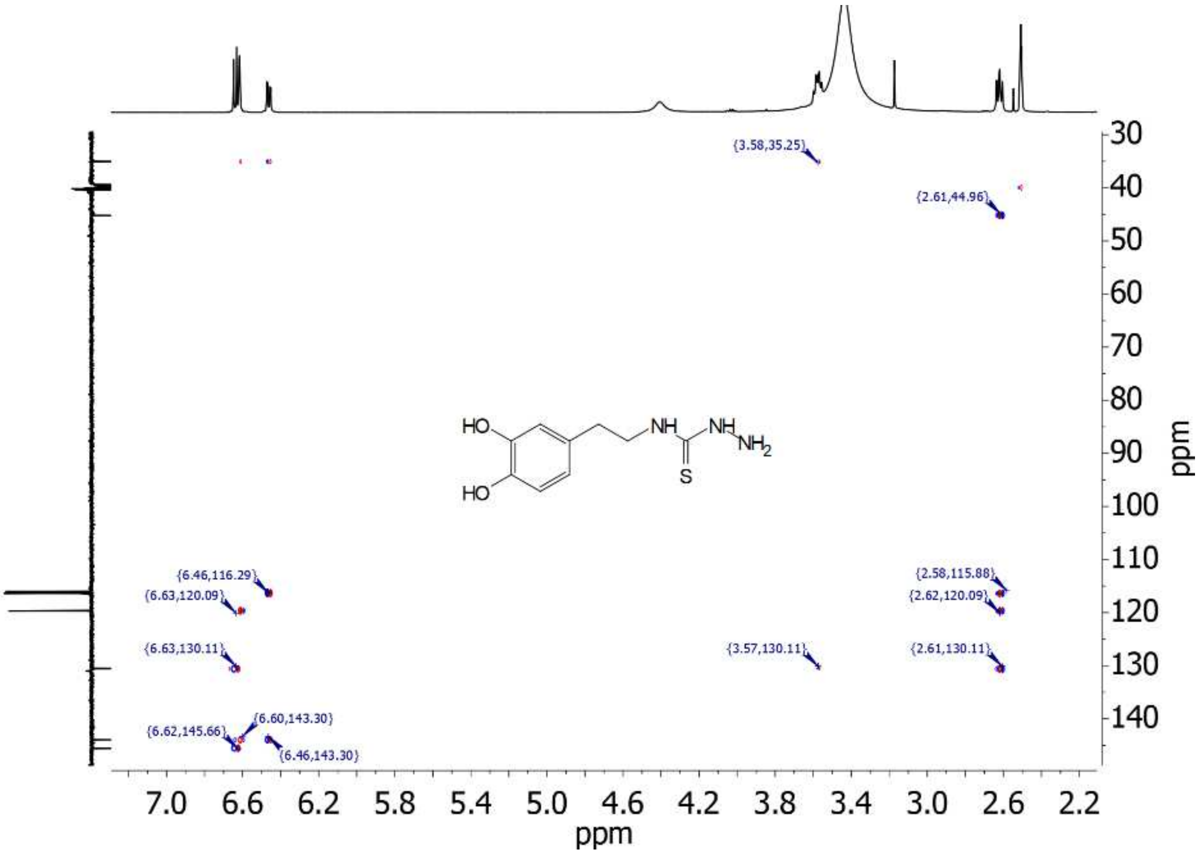

Figure S213. ^{1}H,^{13}C HMBC NMR spectrum of 4-[2-(hydrazidecarbothioamino)ethyl]benzene-1,2-diol in DMSO-d_6 at 499 MHz.

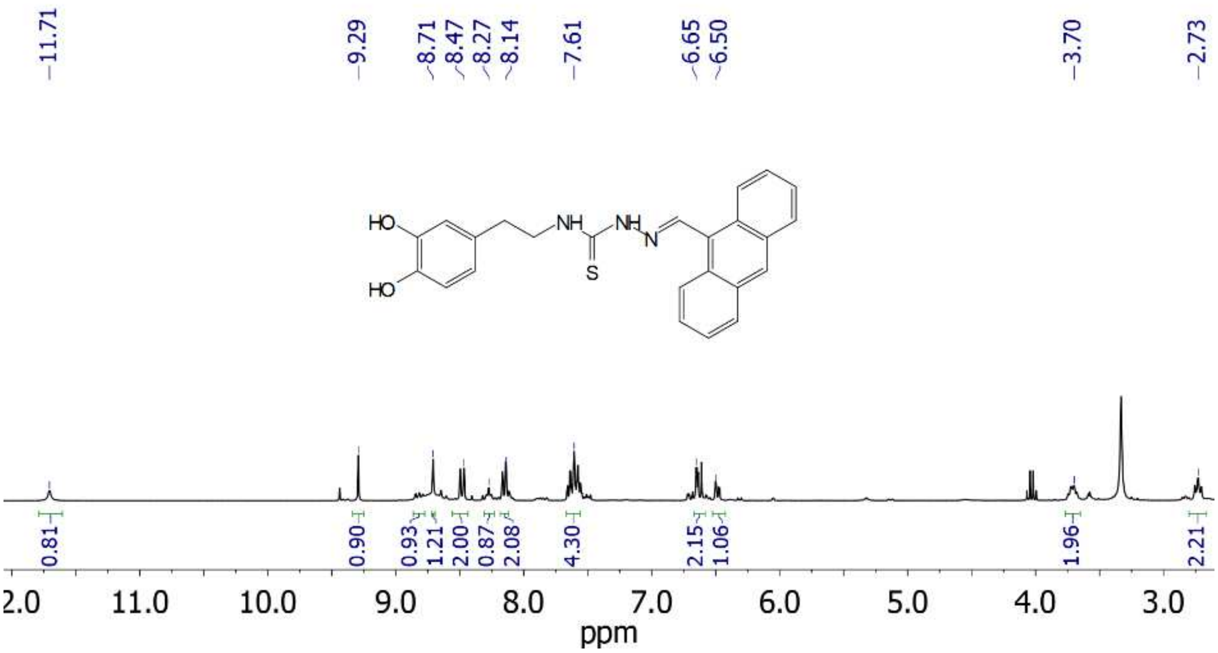

Figure S214.^{1}H NMR spectrum of [(1*E*)-(anthracen-9-yl)methylideneamino][2-(3,4 dihydroxyphenyl) ethylamino] carbothioamide in DMSO-d_6 at 300 MHz.

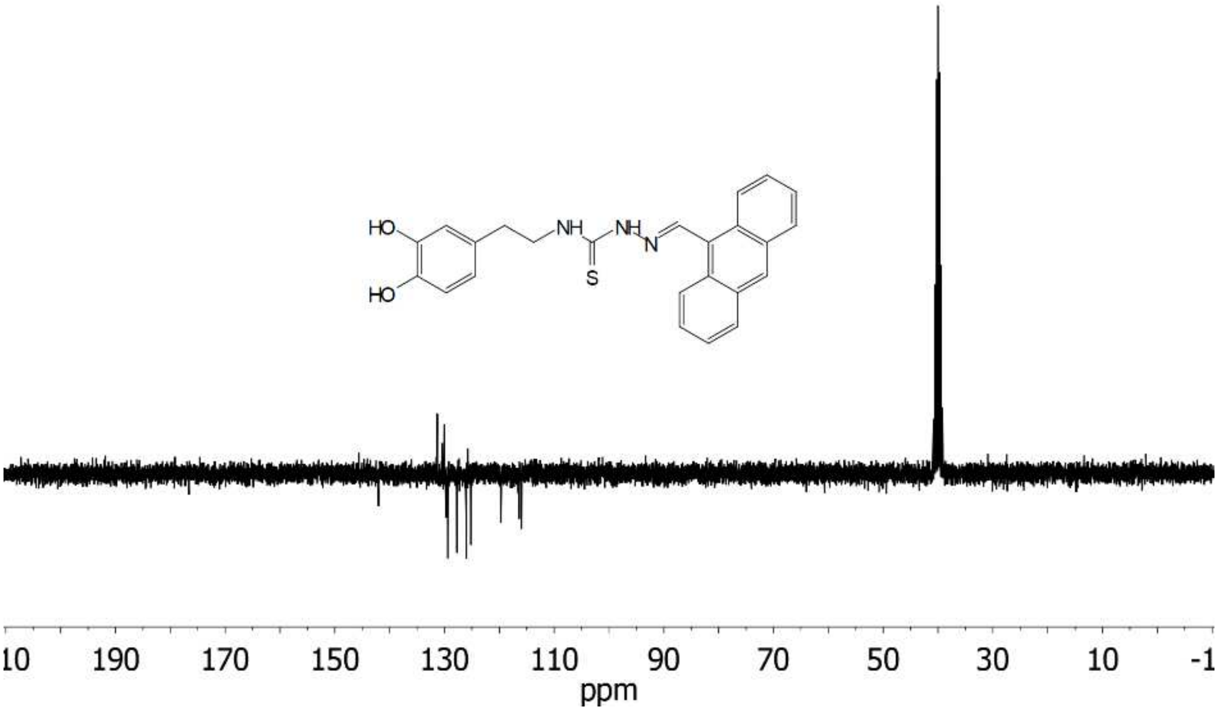

Figure S215. ^{13}C DEPTQ NMR spectrum of [(1*E*)-(anthracen-9-yl)methylideneamino][2-(3,4 dihydroxyphenyl)ethylamino] carbothioamide in DMSO-d_6 at 300 MHz.

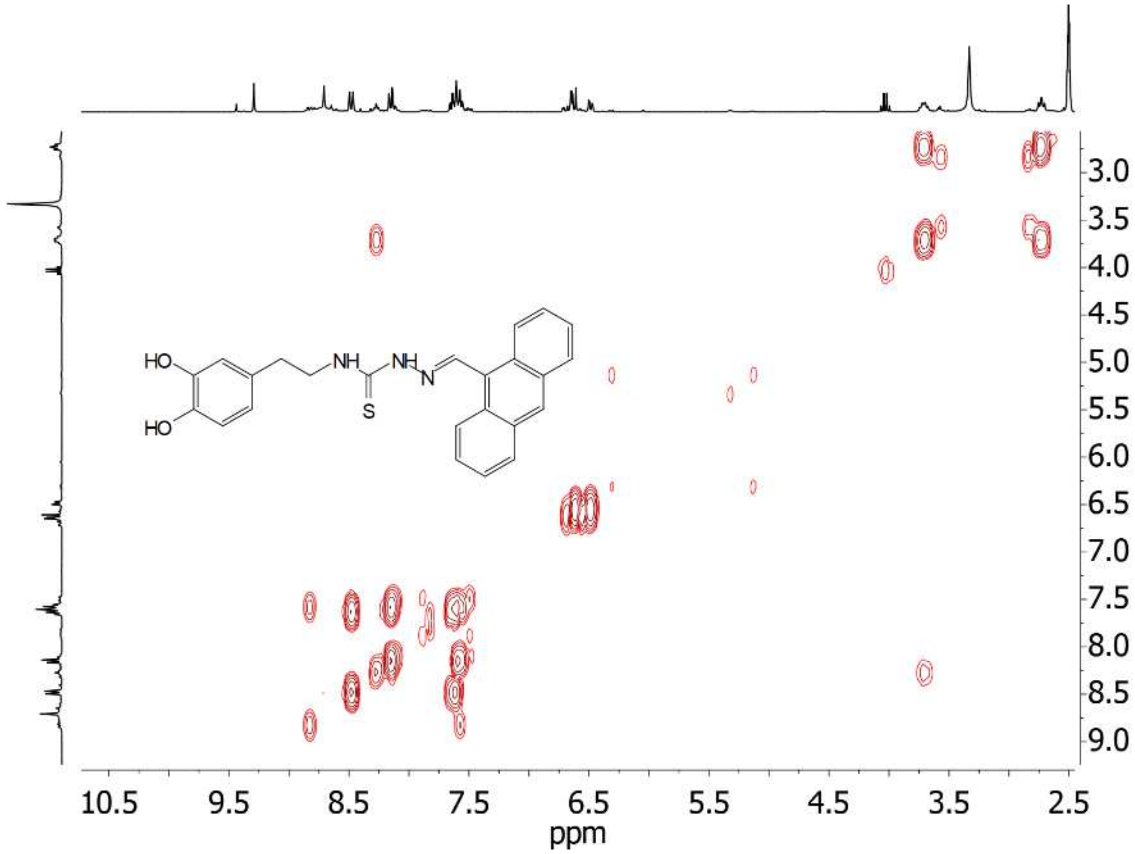

Figure S216. $^{1}H,^{1}H$ COSY NMR spectrum of [(1*E*)-(anthracen-9-yl)methylideneamino][2-(3,4 dihydroxyphenyl)ethylamino] carbothioamide in DMSO-d_6 at 300 MHz.

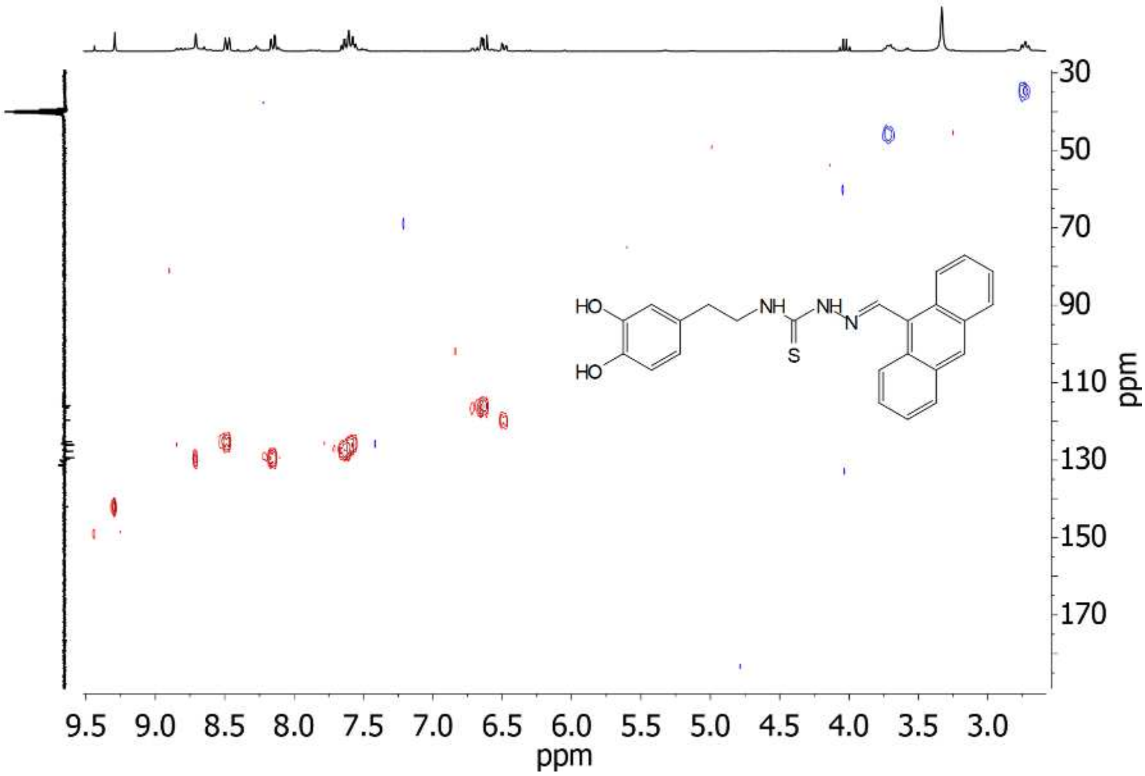

Figure S217. $^{1}H,^{13}C$ HMQC/HSQC NMR spectrum of [(1*E*)-(anthracen-9-yl)methylideneamino][2-(3,4 dihydroxyphenyl)ethylamino] carbothioamide in DMSO-d_6 at 300 MHz.

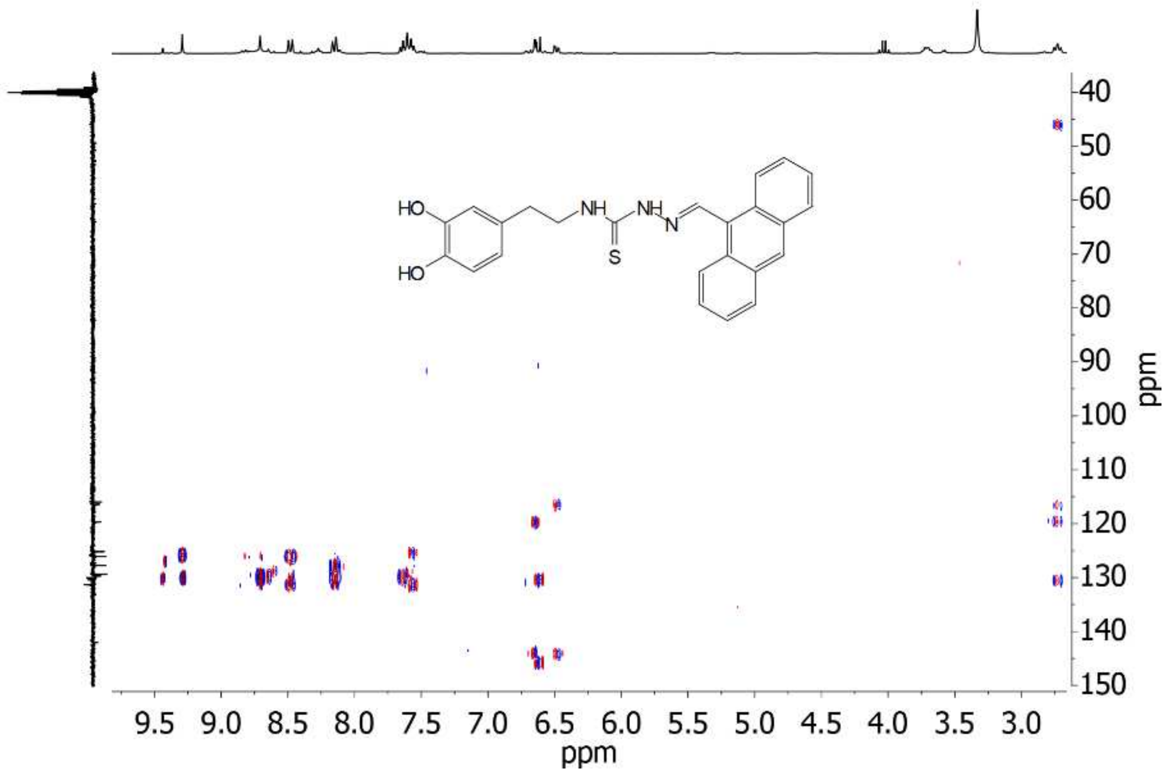

Figure S218. ^{1}H,^{13}C HMBC NMR spectrum of [(1*E*)-(anthracen-9-yl)methylideneamino][2-(3,4 dihydroxyphenyl)ethylamino] carbothioamide in DMSO-*d*$_6$ at 300 MHz.

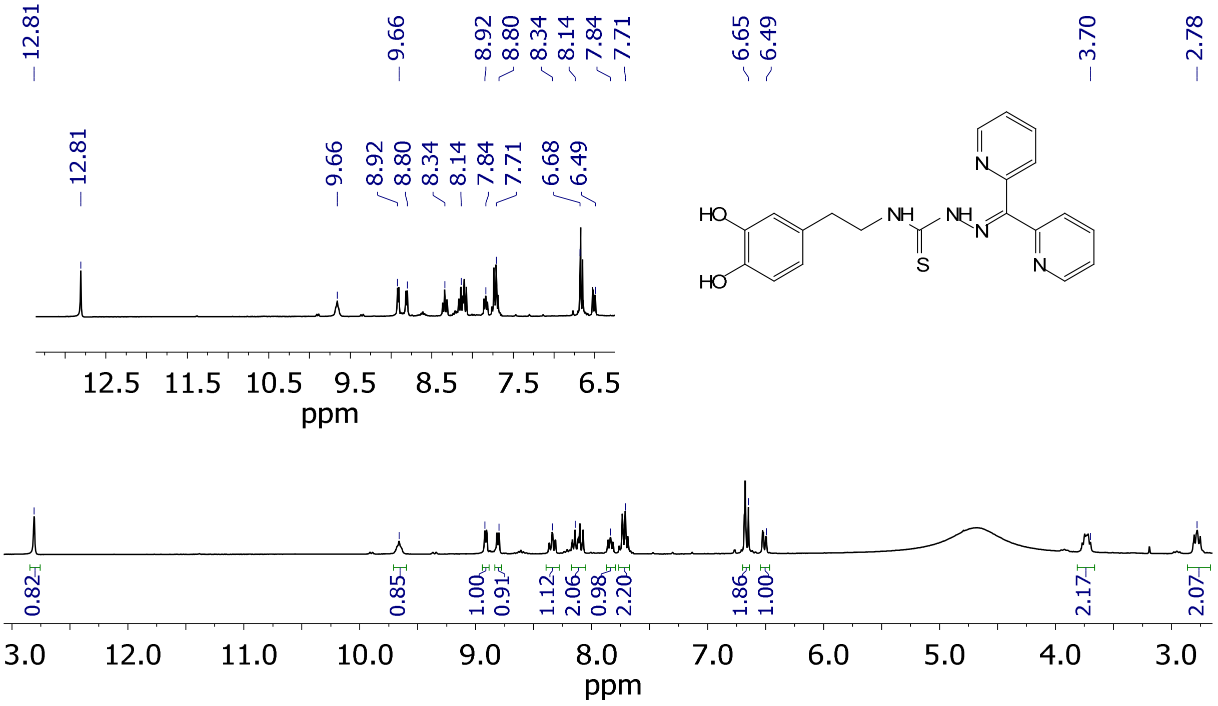

Figure S219.^{1}H NMR spectrum of [Bis(pyridin-2-yl)methylideneamino][2-(3,4dihydroxyphenyl) ethylamino]carbothioamide in DMSO-*d*$_6$ at 300 MHz.

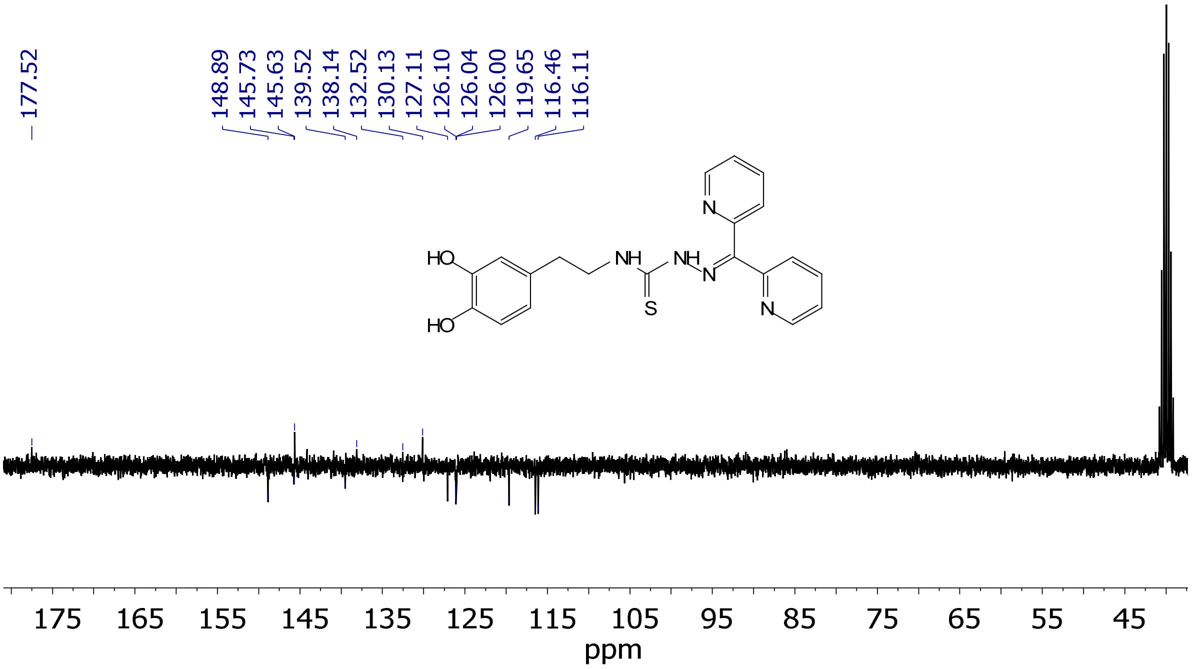

Figure S220. ^{13}C NMR spectrum of [Bis(pyridin-2-yl)methylideneamino][2-(3,4 dihydroxyphenyl) ethylamino] carbothioamide in DMSO-d_6 at 300 MHz.

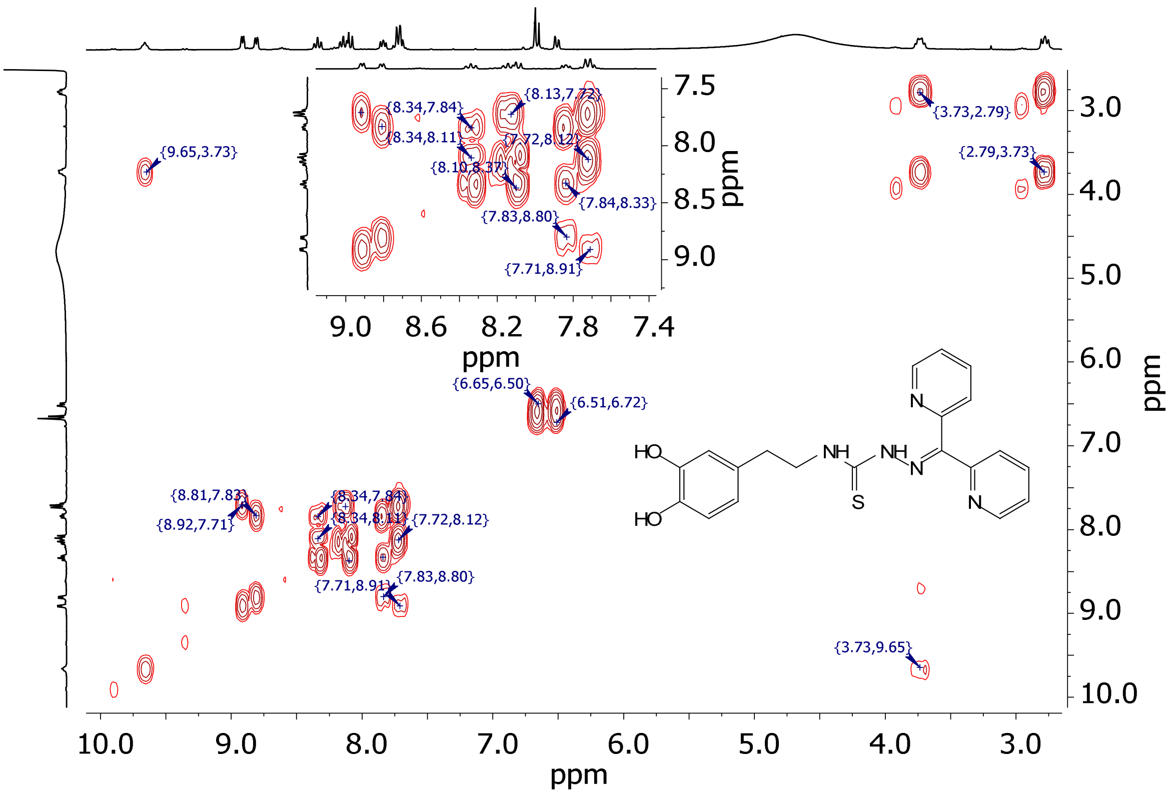

Figure S221. $^1H,^1H$ COSY NMR spectrum of [Bis(pyridin-2-yl)methylideneamino][2-(3,4-dihydroxy phenyl)ethylamino] carbothioamide in DMSO-d_6 at 300 MHz.

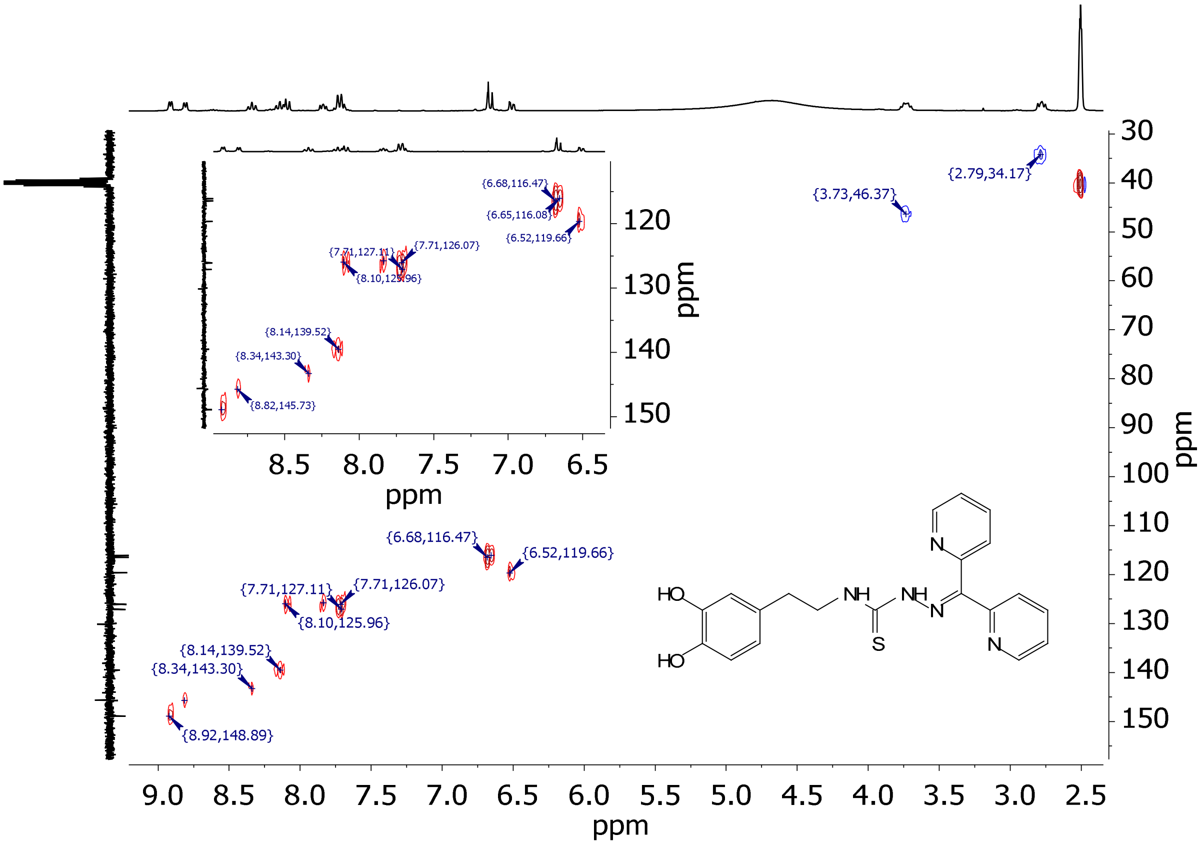

Figure S222. $^{1}H,^{13}C$ HMQC/HSQC NMR spectrum of [Bis(pyridin-2-yl)methylideneamino][2-(3,4 dihydroxyphenyl)ethylamino] carbothioamide in DMSO-d_6 at 300 MHz.

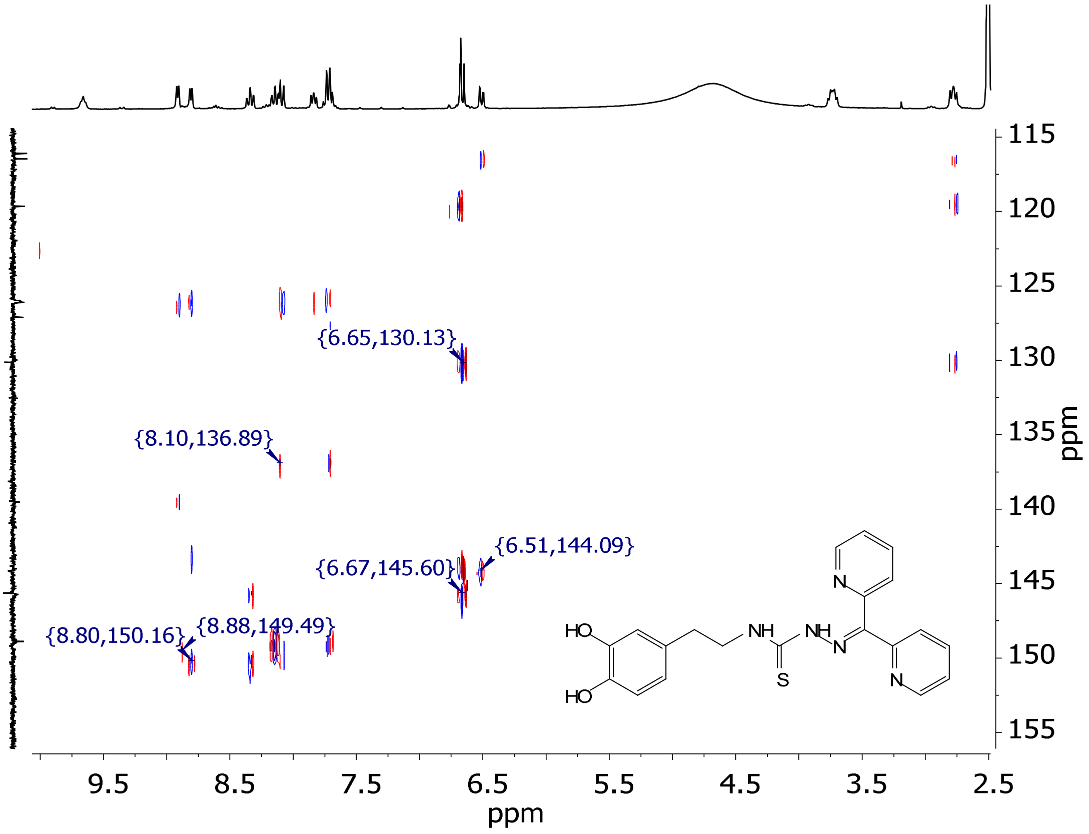

Figure S223. $^{1}H,^{13}C$ HMBC NMR spectrum of [bis(pyridin-2-yl)methylideneamino][2-(3,4 dihydroxyphenyl)ethylamino] carbothioamide in DMSO-d_6 at 300 MHz.

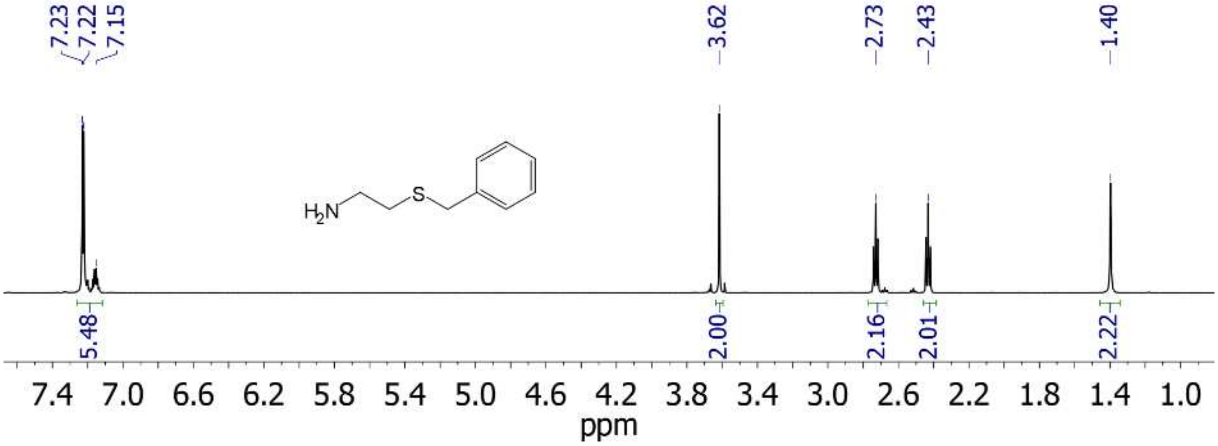

Figure S224. ^{1}H NMR spectrum of 2-benzylsulfanyl ethylamine in $CDCl_3$ at 499 MHz.

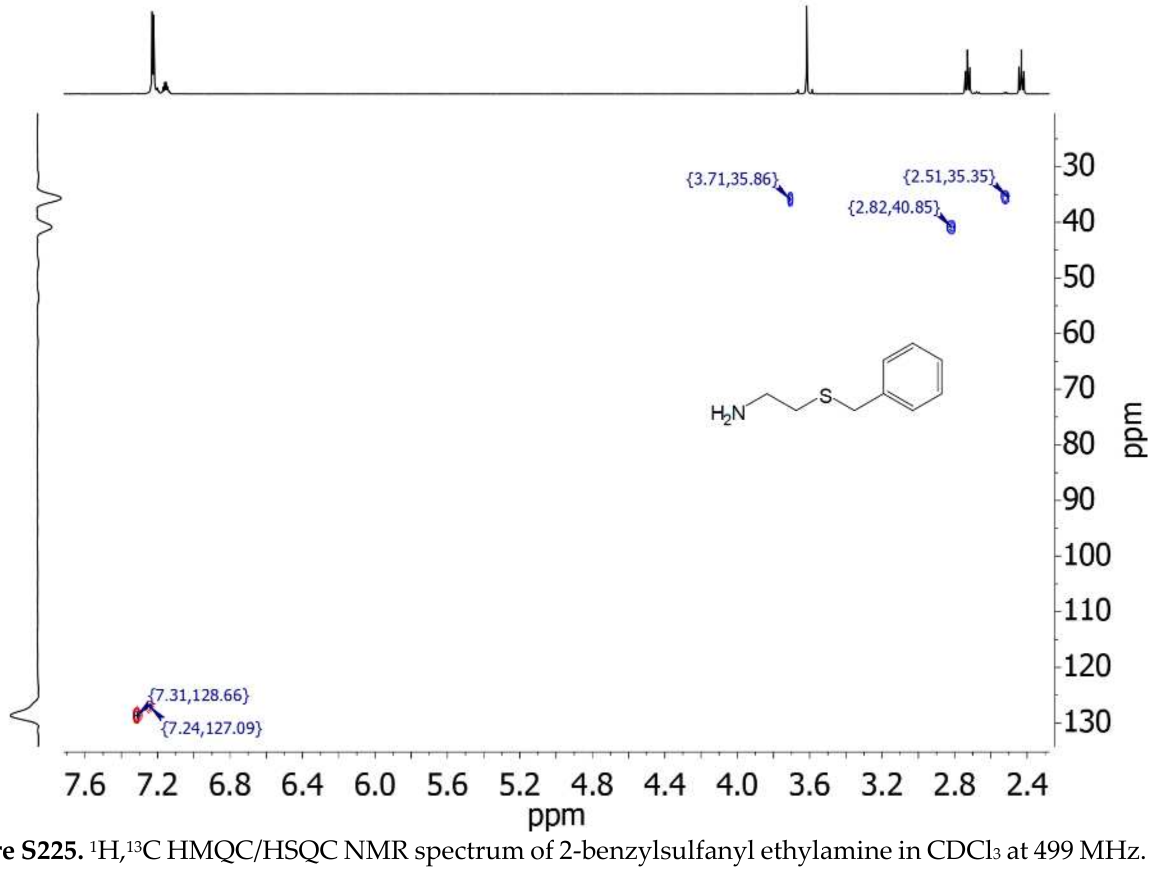

Figure S225. $^{1}H,^{13}C$ HMQC/HSQC NMR spectrum of 2-benzylsulfanyl ethylamine in $CDCl_3$ at 499 MHz.

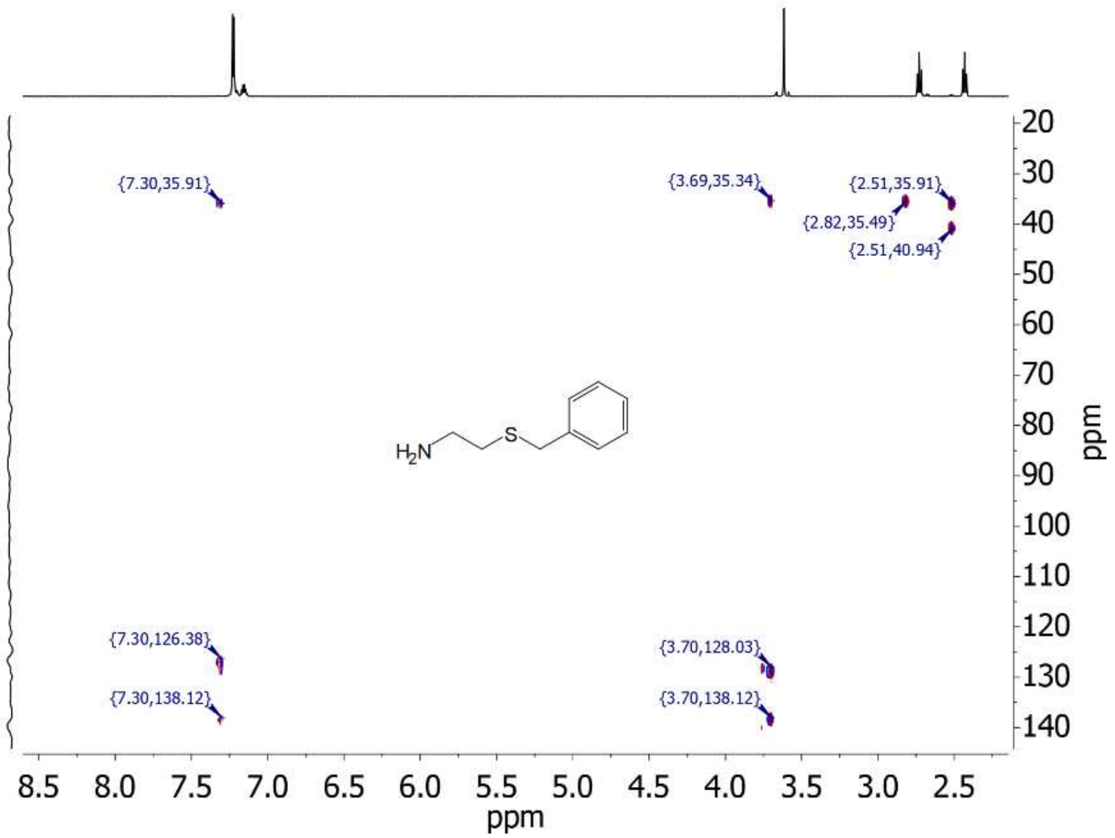

Figure S226. $^{1}H,^{13}C$ HMBC NMR spectrum of 2-benzylsulfanyl ethylamine in $CDCl_3$ at 499 MHz.

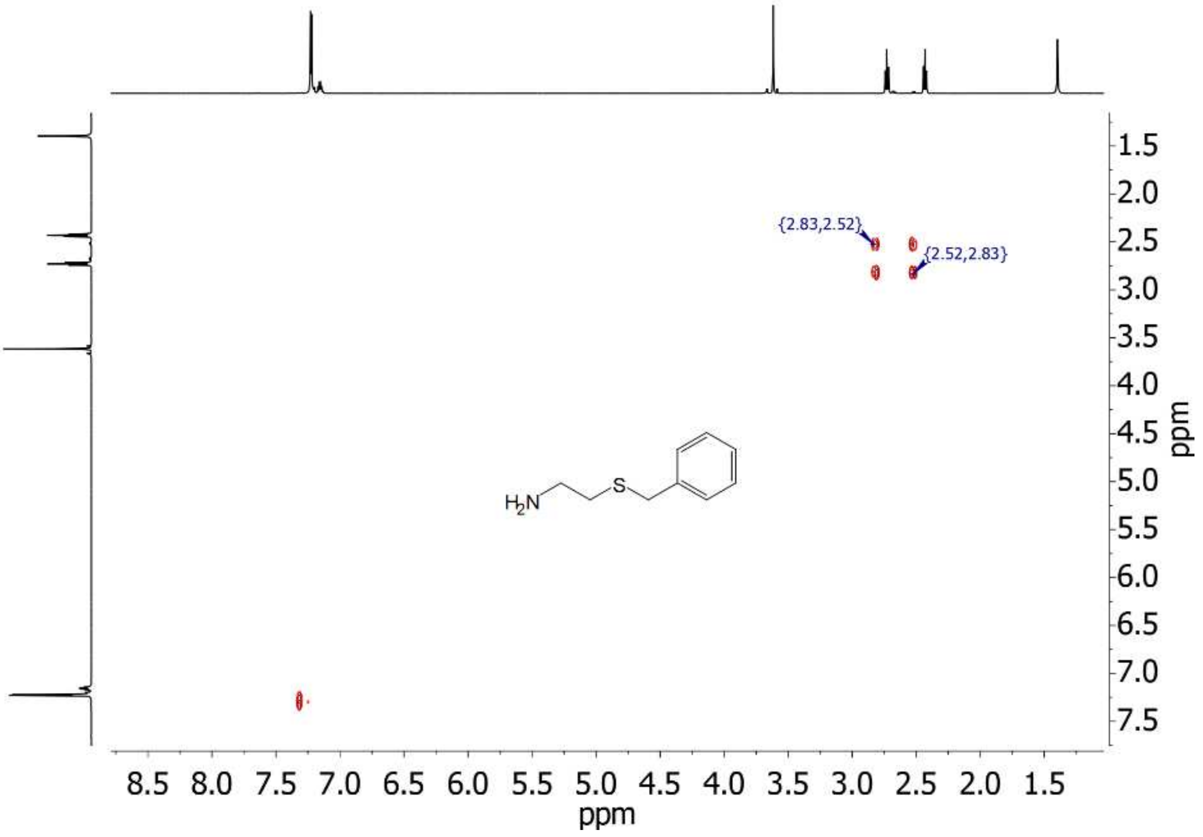

Figure S227. $^{1}H,^{1}H$ COSY NMR spectrum of 2-benzylsulfanyl ethylamine in DMSO-d_6 at 499 MHz.

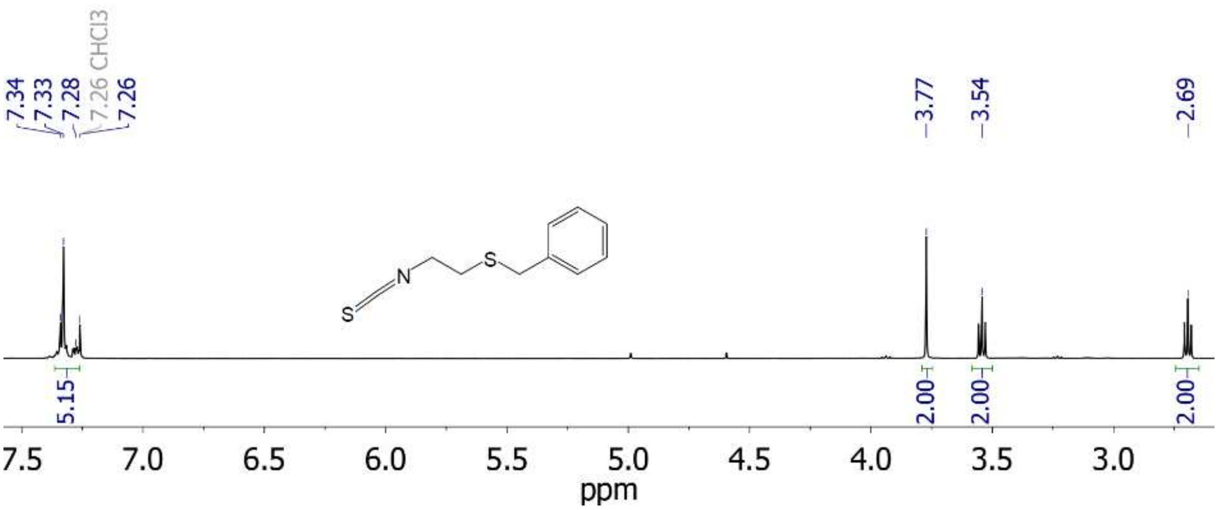

Figure S228. ^{1}H NMR spectrum of 2-benzylsulfanylethyl isothiocyanate in $CDCl_3$ at 499 MHz.

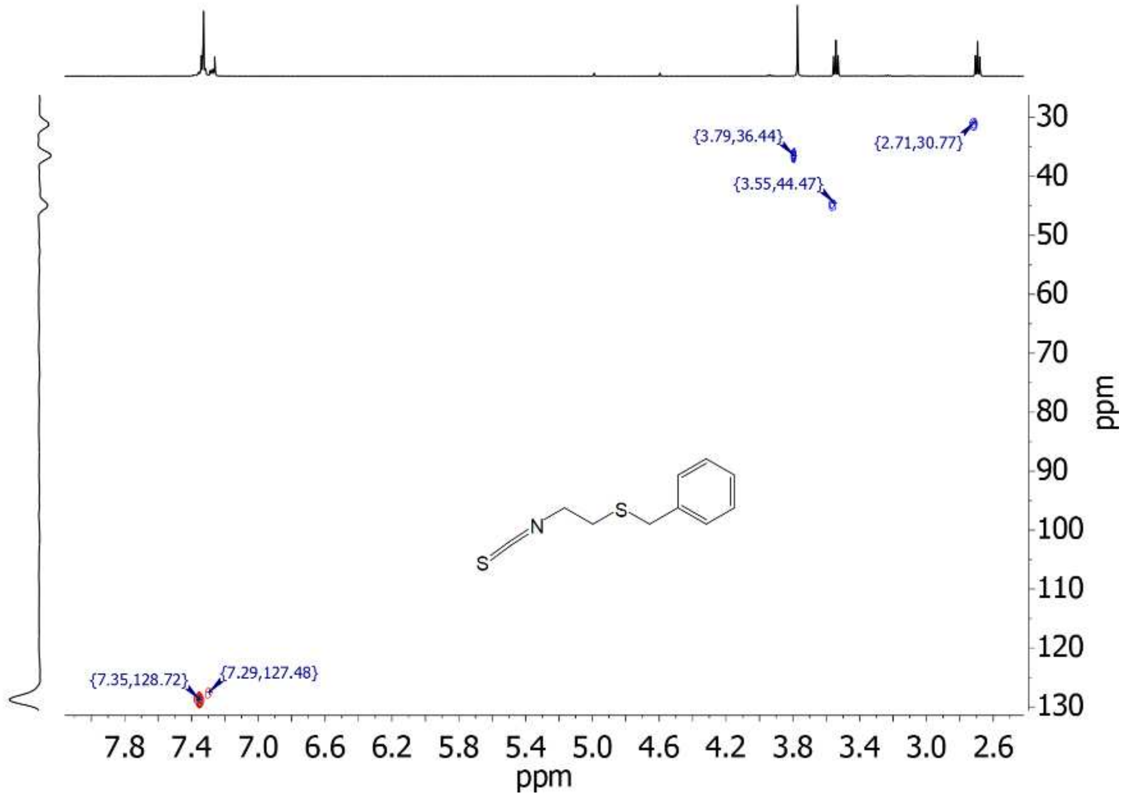

Figure S229. ^{1}H,^{13}C HMQC/HSQC NMR spectrum of 2-benzylsulfanylethyl isothiocyanate in $CDCl_3$ at 499 MHz.

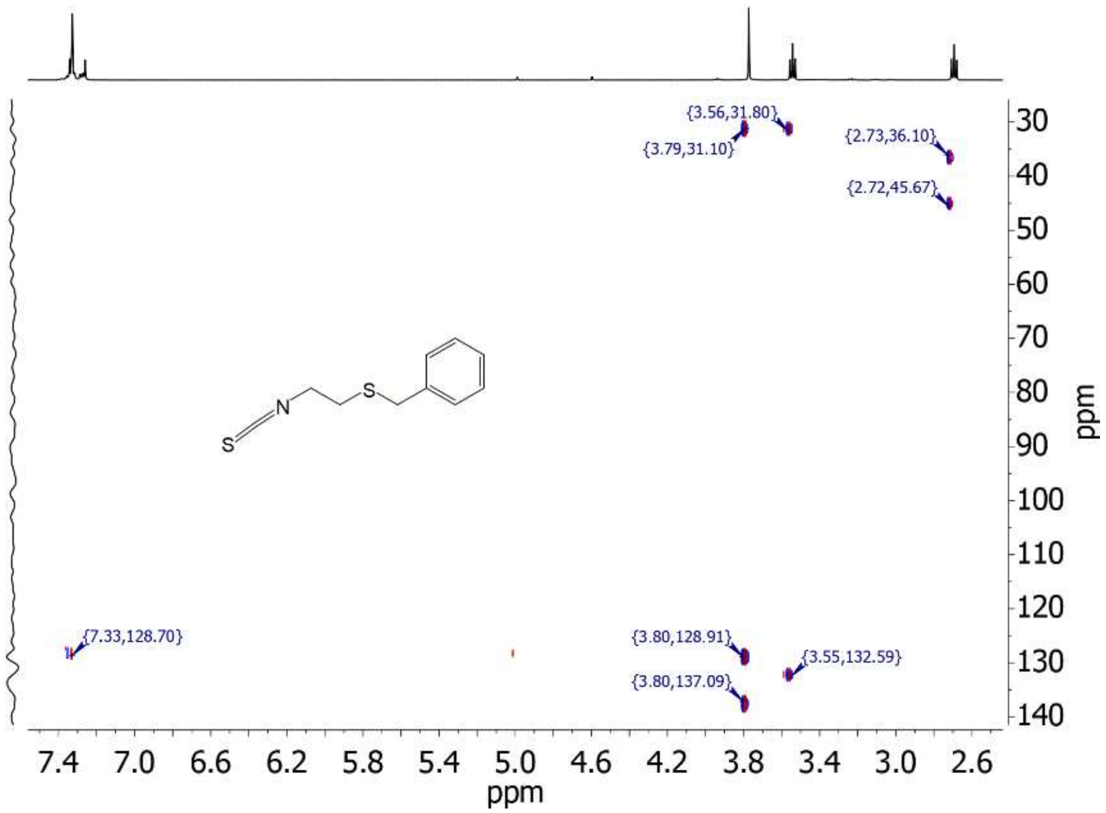

Figure S230. $^{1}H,^{13}C$ HMBC NMR spectrum of 2-benzylsulfanylethyl isothiocyanate in $CDCl_3$ at 499 MHz.

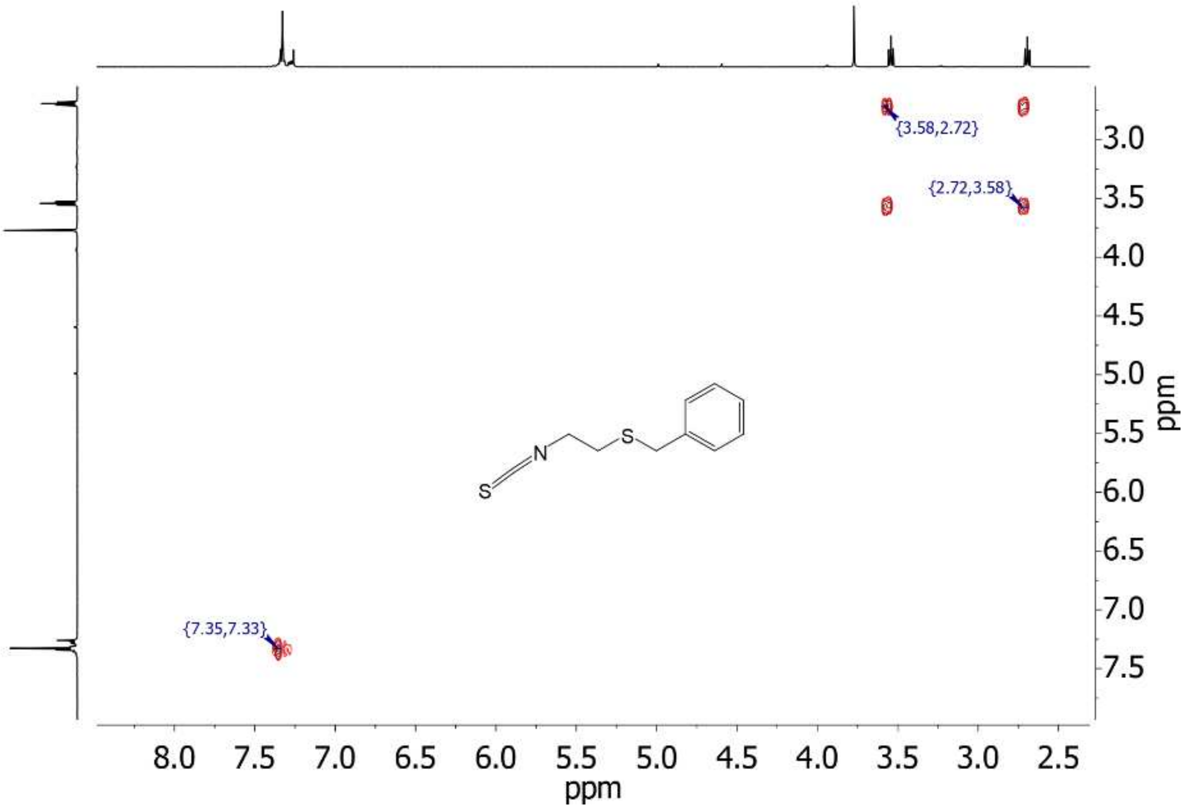

Figure S231. $^{1}H,^{1}H$ COSY NMR spectrum of 2-benzylsulfanylethyl isothiocyanate in DMSO-d_6 at 499 MHz.

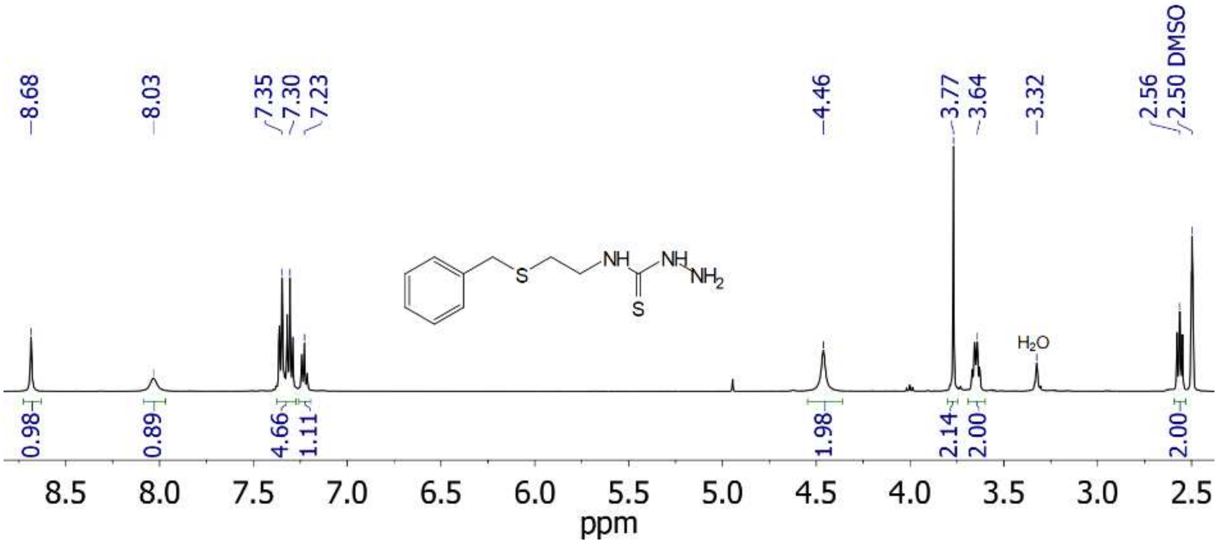

Figure S232. ^{1}H NMR spectrum of 4-(2-benzylsulfanylethyl)-thiosemicarbazide in DMSO-d_6 at 499 MHz.

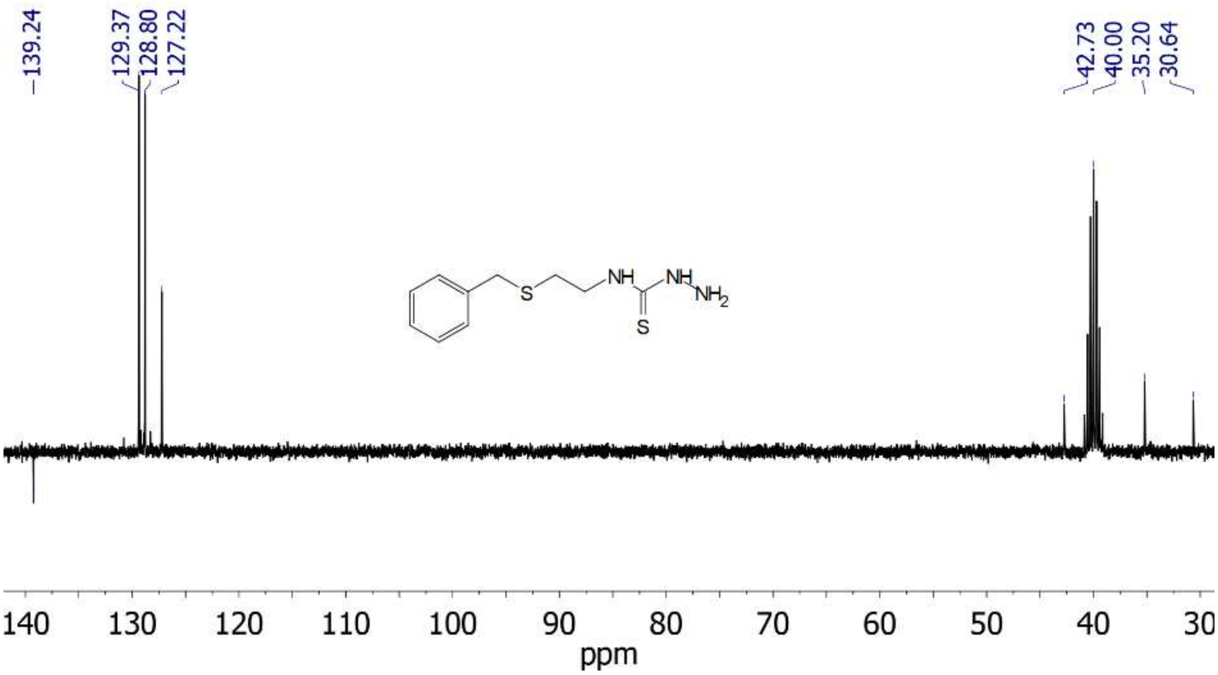

Figure S233. ^{13}C DEPTQ NMR spectrum of 4-(2-benzylsulfanylethyl)-thiosemicarbazide in DMSO-d_6 at 300 MHz.

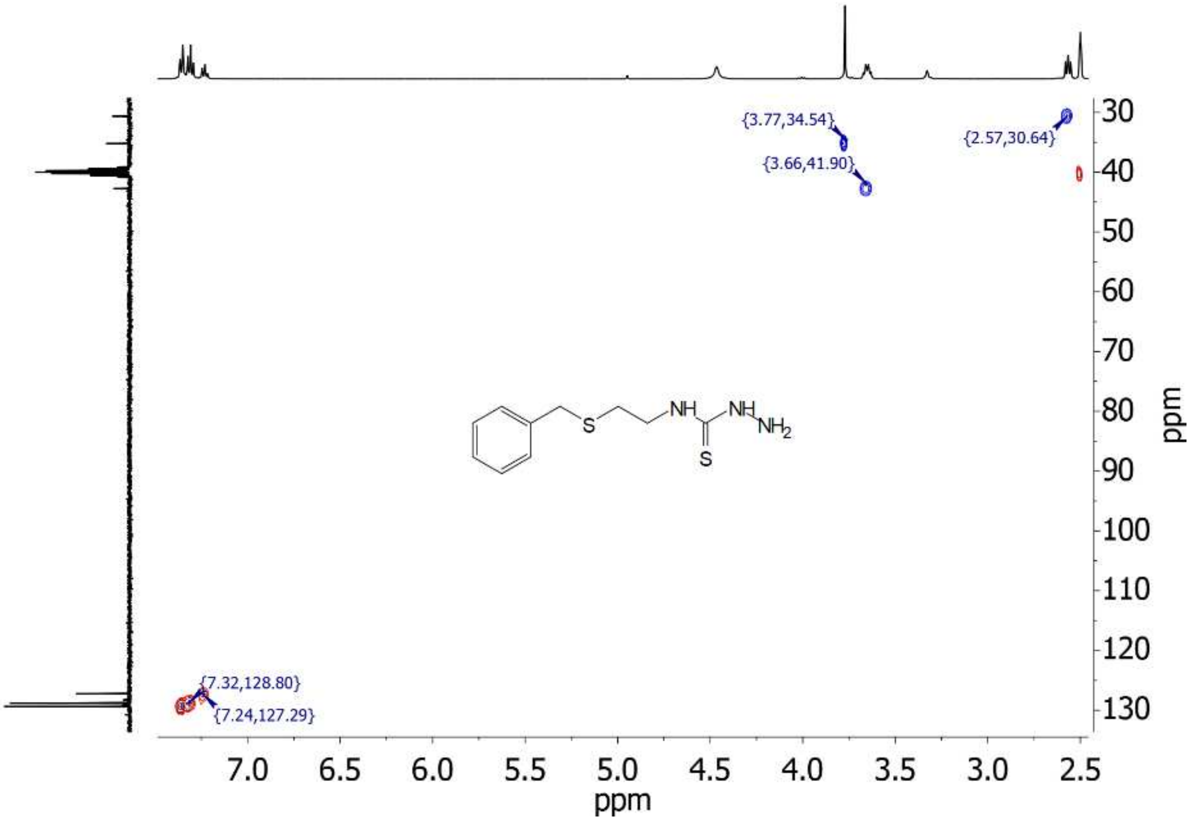

Figure S234. $^{1}H,^{13}C$ HMQC/HSQC NMR spectrum of 4-(2-benzylsulfanylethyl)-thiosemicarbazide in DMSO-d_6 at 499 MHz.

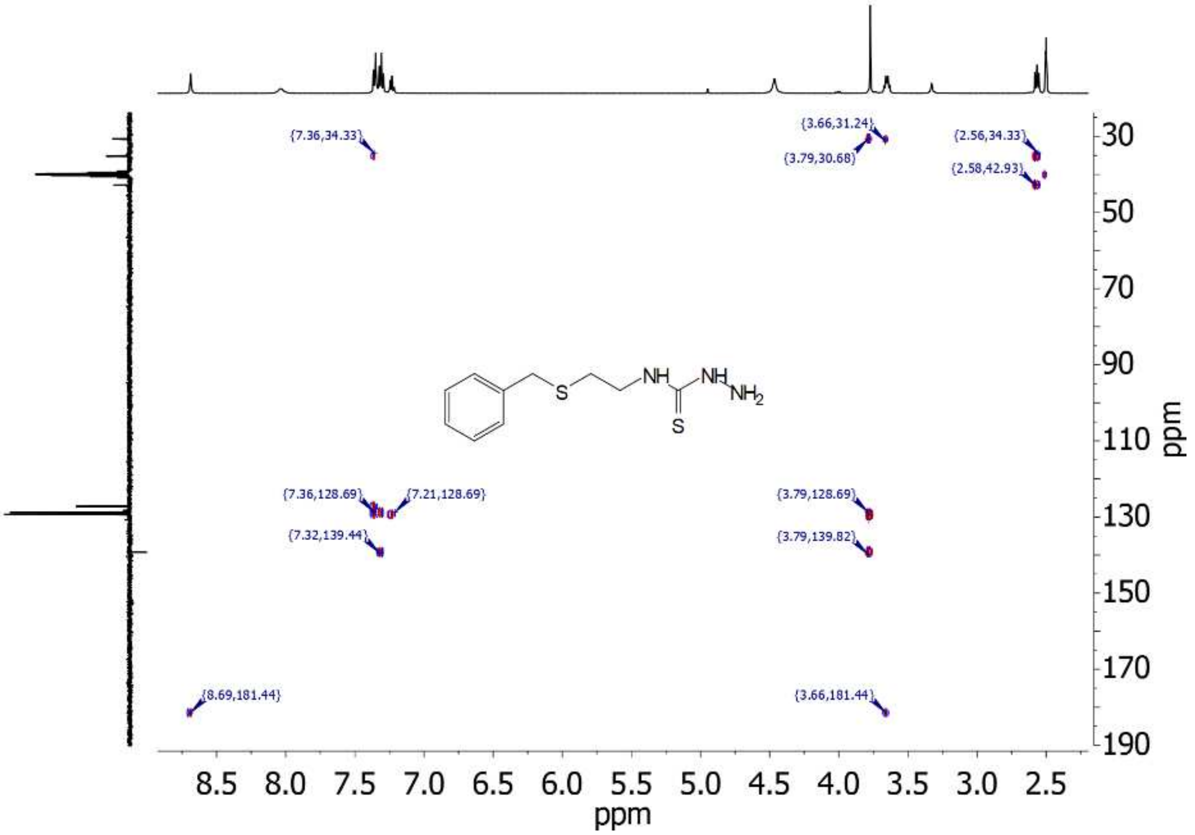

Figure S235. $^{1}H,^{13}C$ HMBC NMR spectrum of 4-(2-benzylsulfanylethyl)-thiosemicarbazide in DMSO-d_6 at 499 MHz.

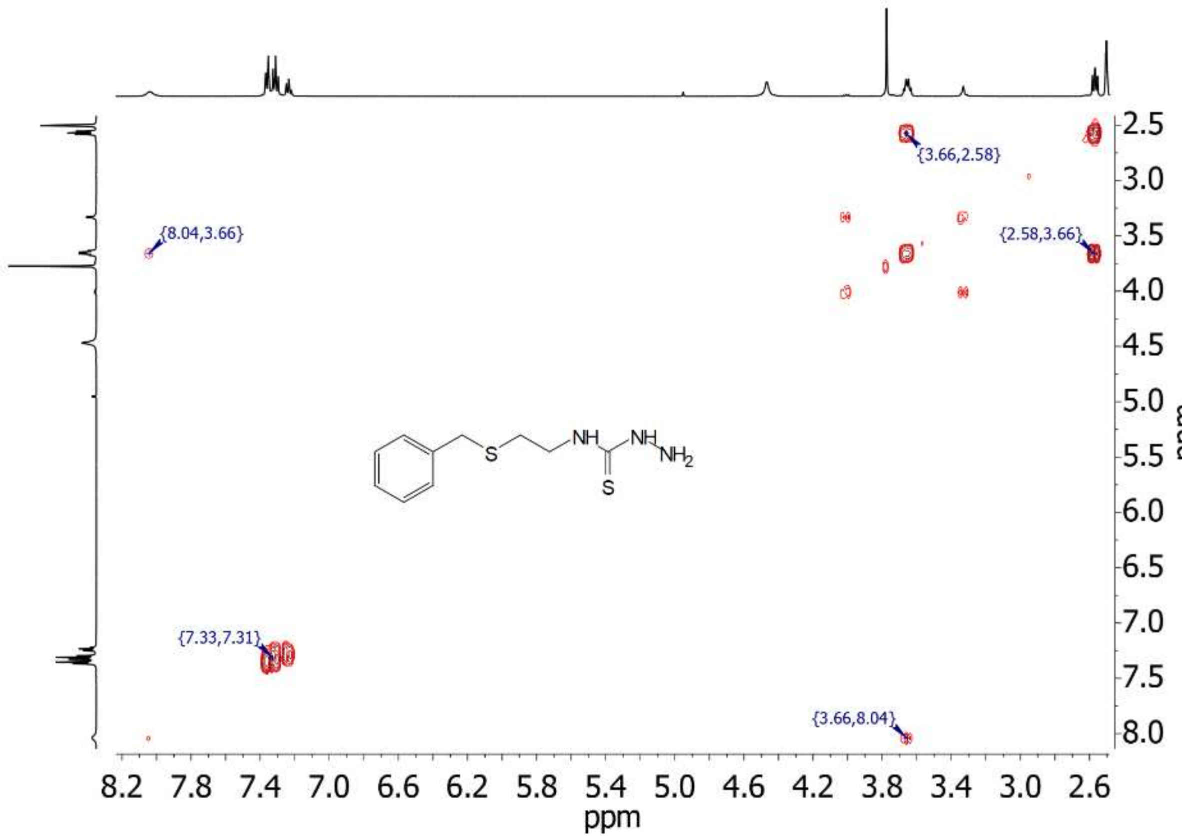

Figure S236. ${}^1H,{}^1H$ COSY NMR spectrum of 4-(2-benzylsulfanylethyl)-thiosemicarbazide in DMSO-d_6 at 499 MHz.

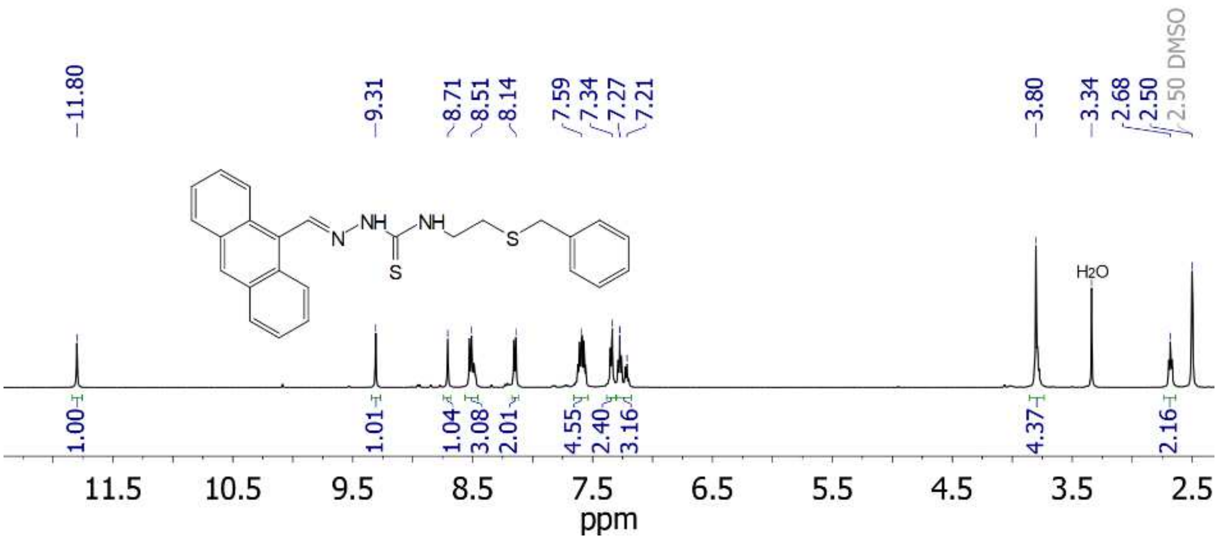

Figure S237. ${}^1H$ NMR spectrum of 9-anthraldehyde-4-(2-benzylsulfanylethyl)-3-thiosemicarbazone in DMSO-d_6 at 499 MHz.

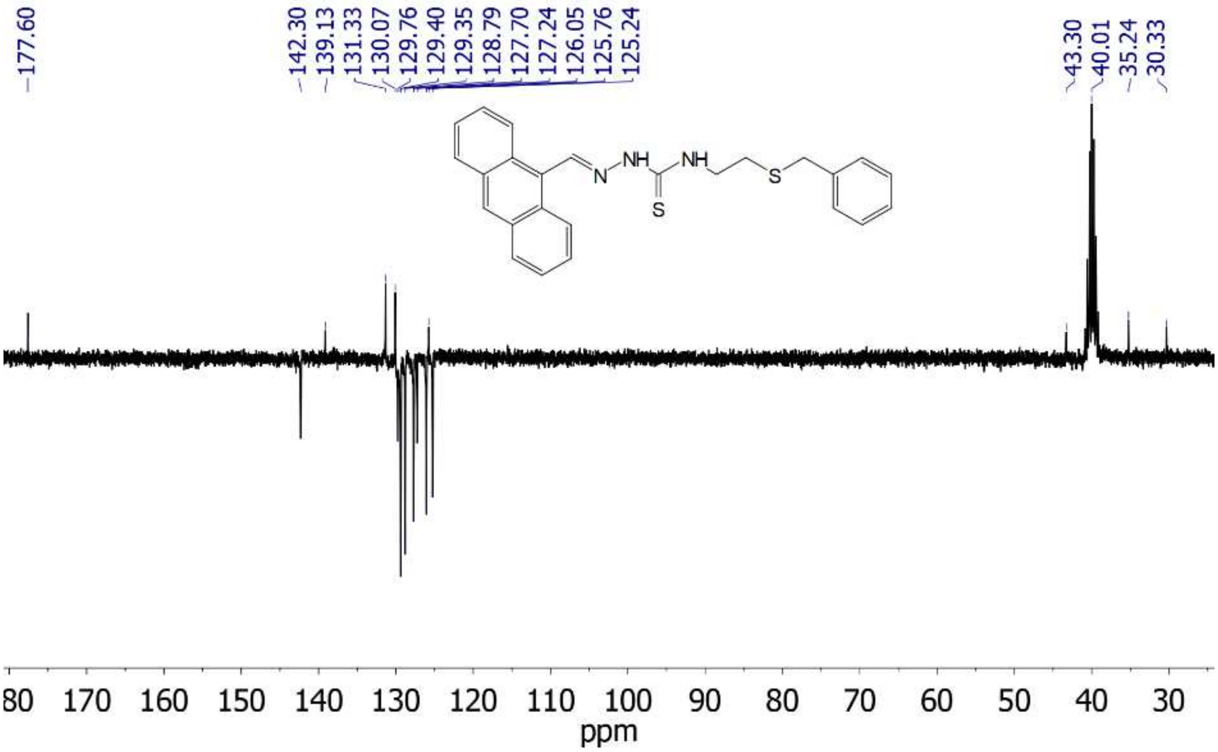

Figure S238. ^{13}C DEPTQ NMR spectrum of 9-anthraldehyde-4-(2-benzylsulfanylethyl)-3-thiosemicarbazone in DMSO-d_6 at 300 MHz.

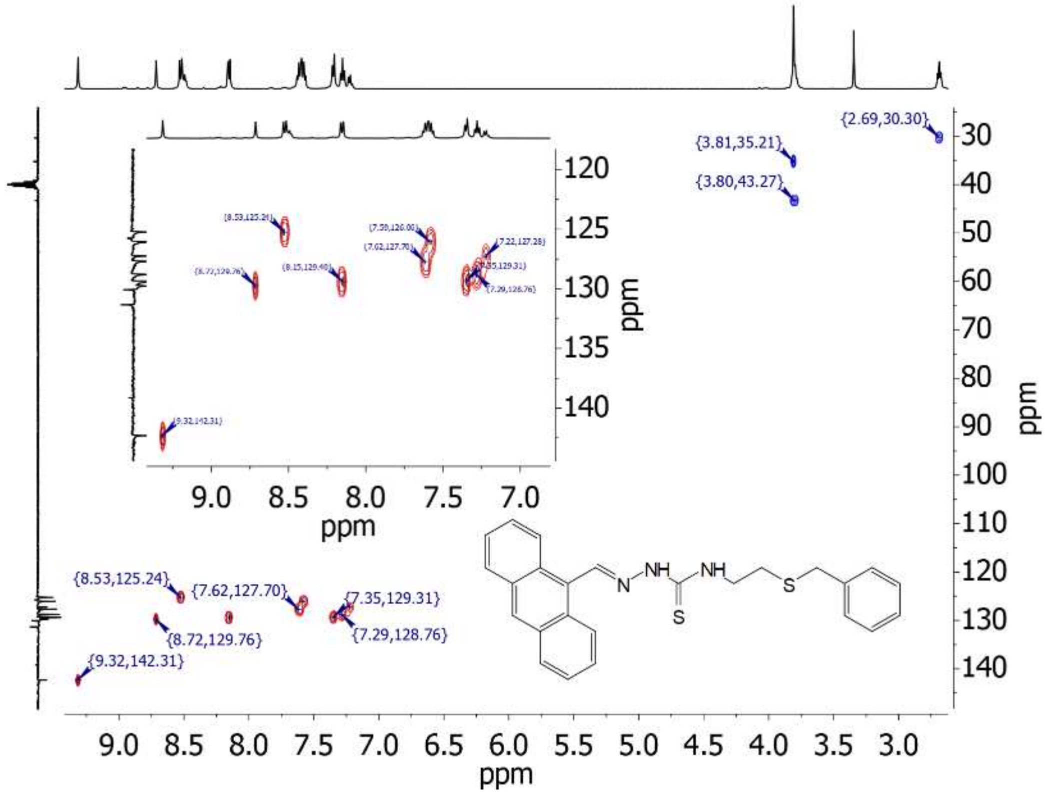

Figure S239. ^{1}H,^{13}C HMQC/HSQC NMR spectrum of 9-anthraldehyde-4-(2-benzylsulfanylethyl)-3-thiosemicarbazone in DMSO-d_6 at 499 MHz.

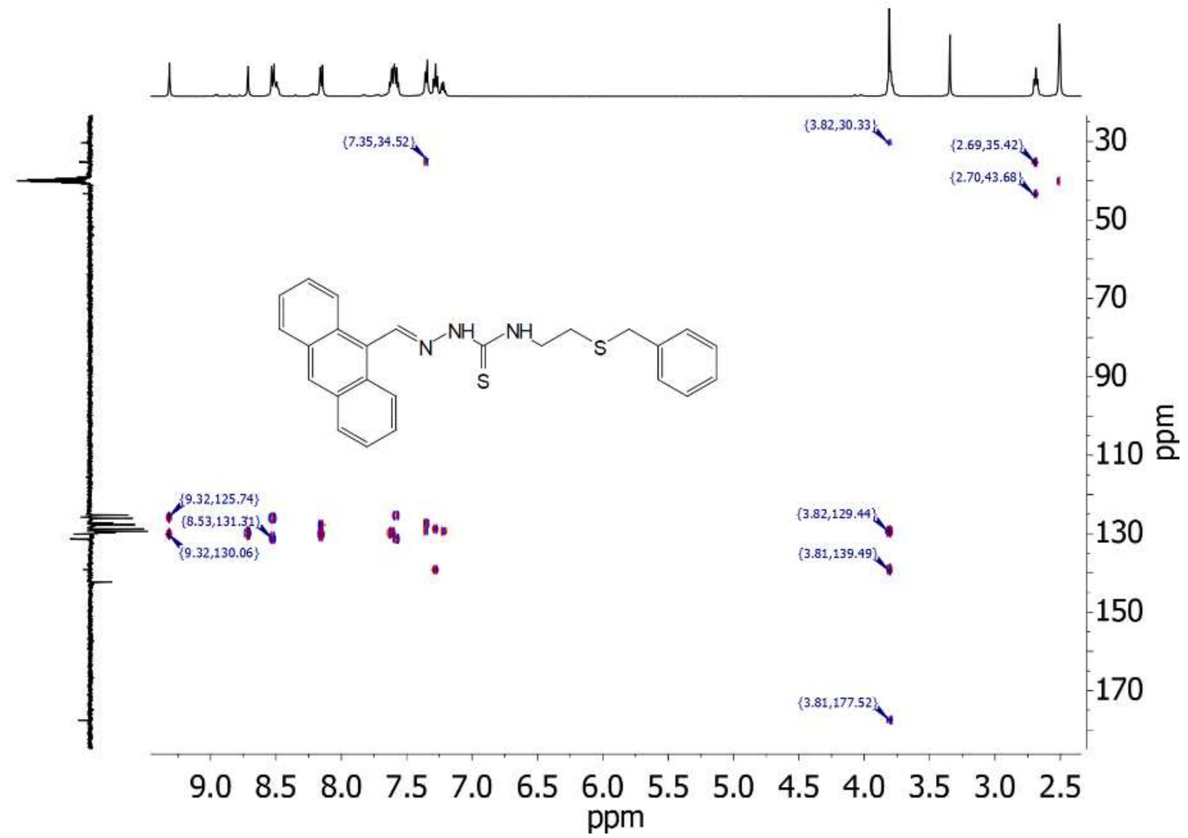

Figure S240. $^{1}H,^{13}C$ HMBC NMR spectrum of 9-anthraldehyde-4-(2-benzylsulfanylethyl)-3-thiosemicarbazone in DMSO-d_6 at 499 MHz.

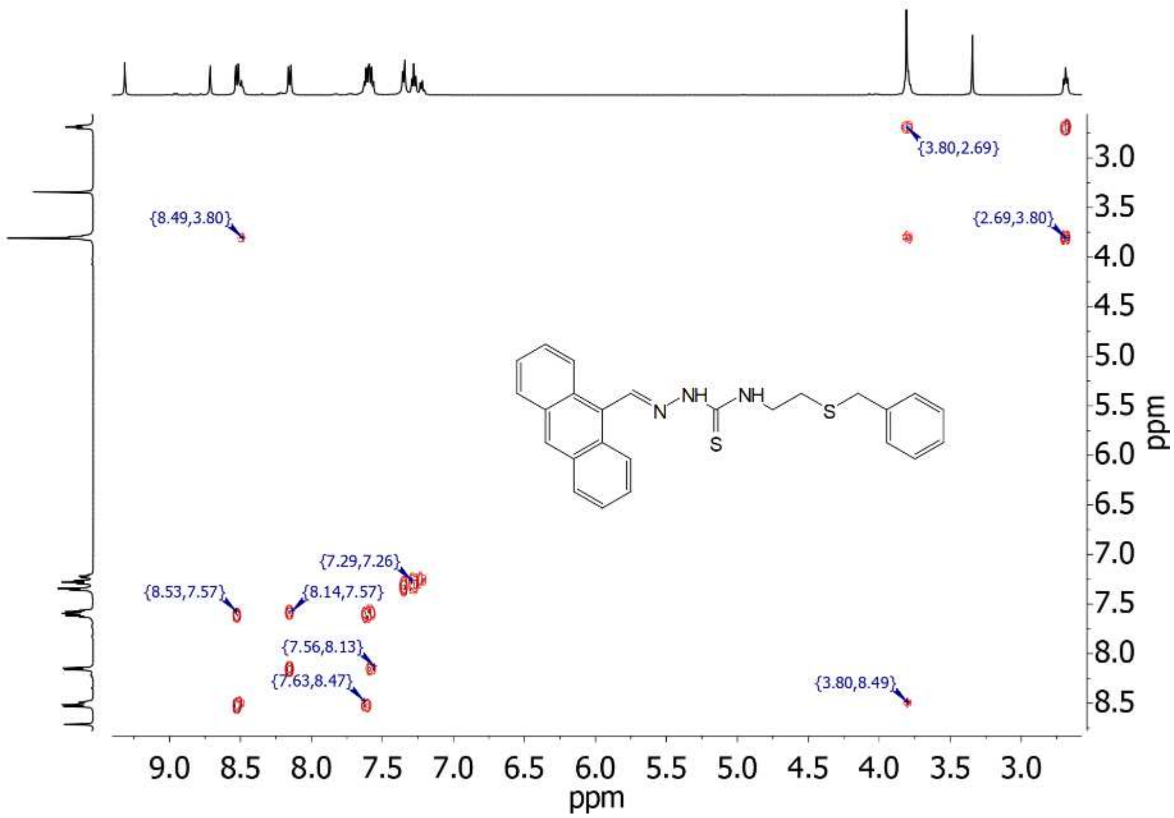

Figure S241. $^{1}H,^{1}H$ COSY NMR spectrum of 9-anthraldehyde-4-(2-benzylsulfanylethyl)-3-thiosemicarbazone in DMSO-d_6 at 499 MHz.

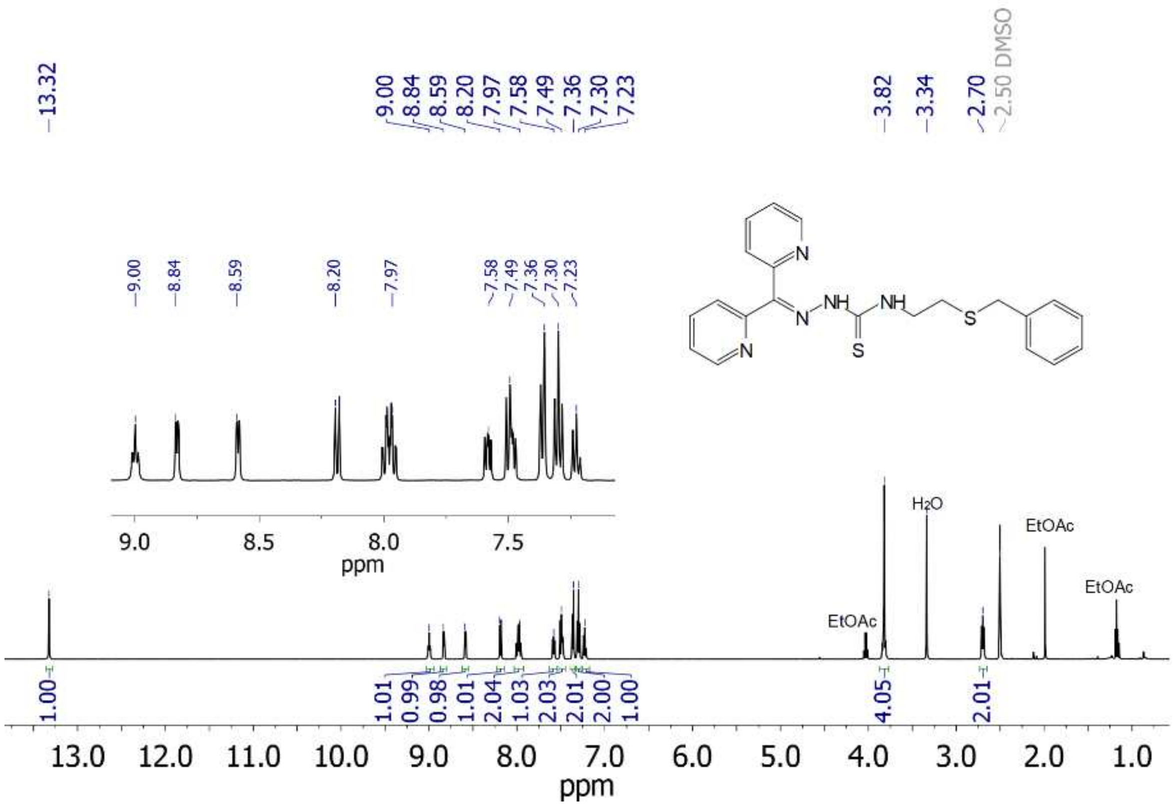

Figure S242. ^{1}H NMR spectrum of di-2-pyridylketone-4-(2-benzylsulfanylethyl)-3-thiosemicarbazone in DMSO-d_6 at 499 MHz.

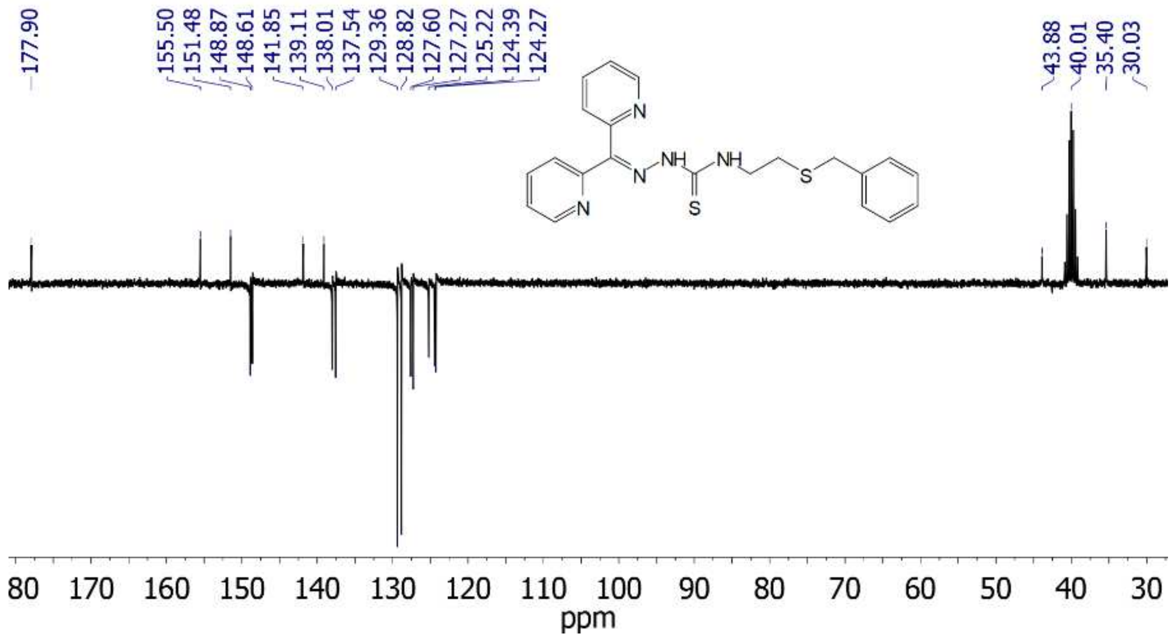

Figure S243. ^{13}C DEPTQ NMR spectrum of di-2-pyridylketone-4-(2-benzylsulfanylethyl)-3-thiosemicarbazone in DMSO-d_6 at 300 MHz.

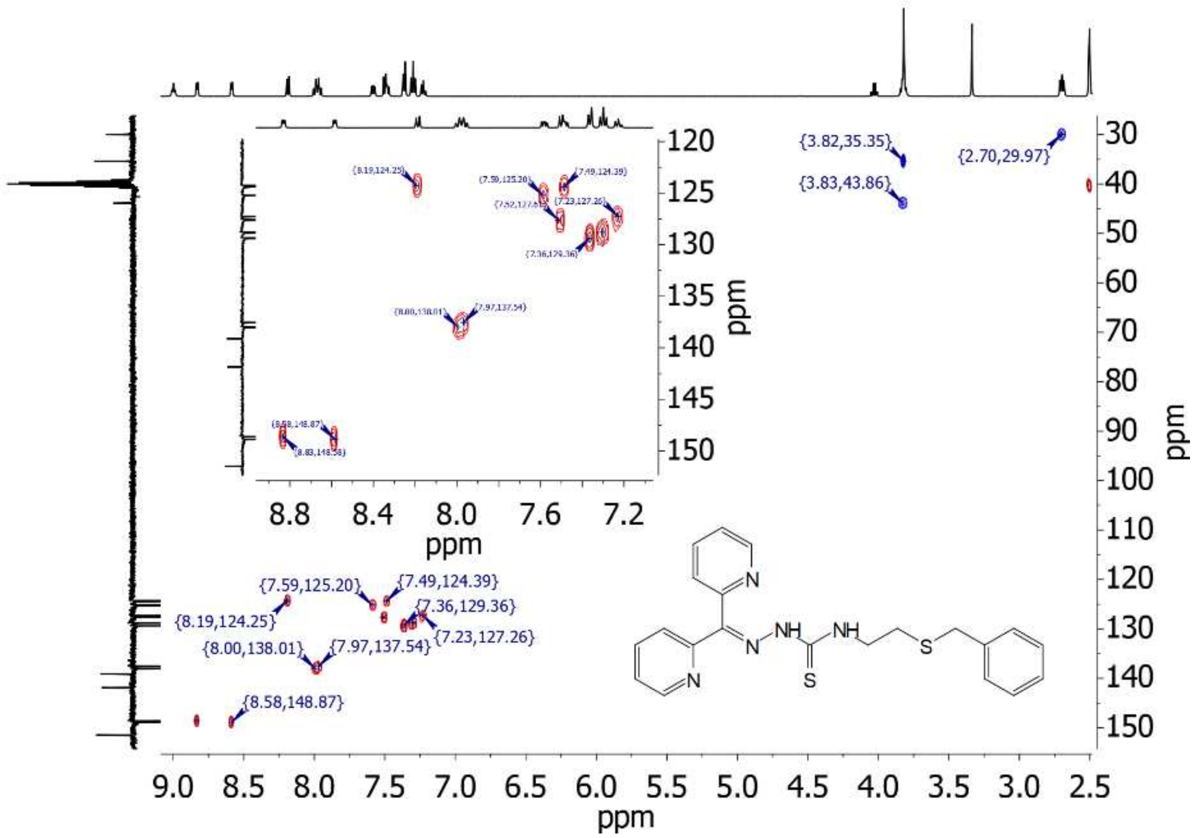

Figure S244. $^{1}H,^{13}C$ HMQC/HSQC NMR spectrum of di-2-pyridylketone-4-(2-benzylsulfanylethyl)-3-thiosemicarbazone in DMSO-d_6 at 499 MHz.

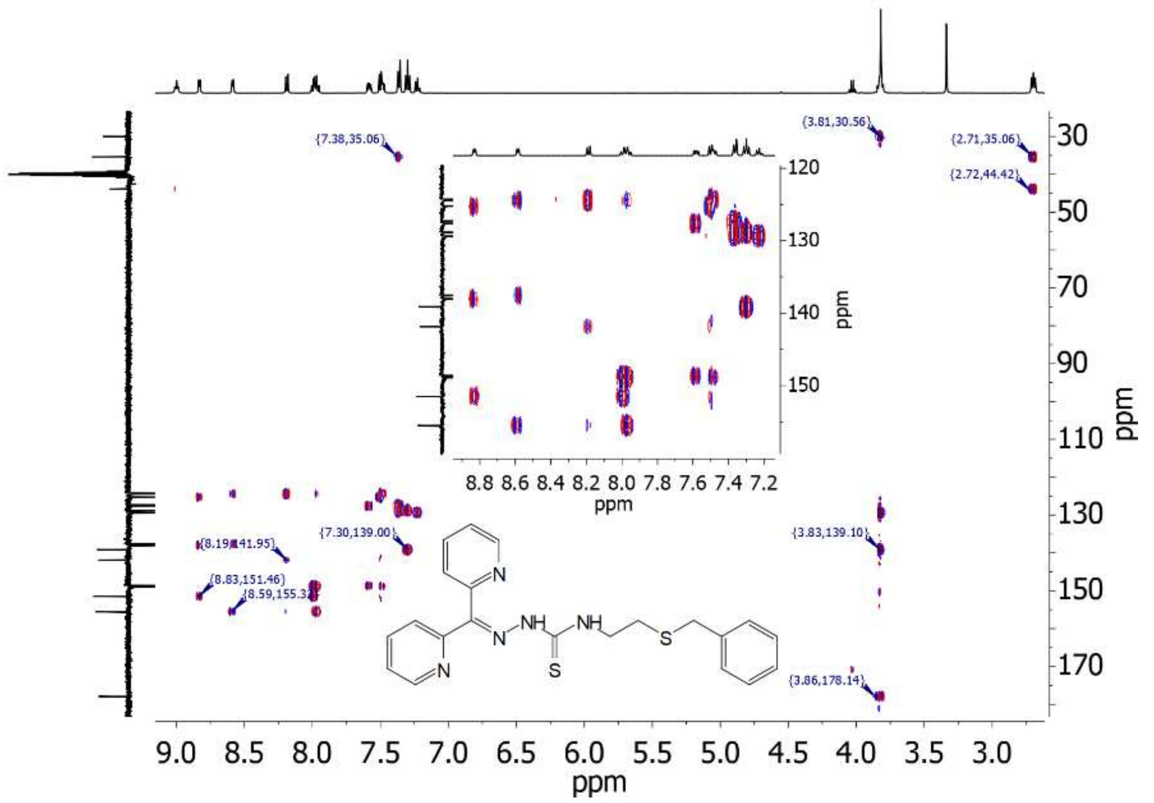

Figure S245. $^{1}H,^{13}C$ HMBC NMR spectrum of di-2-pyridylketone-4-(2-benzylsulfanylethyl)-3-thiosemicarbazone in DMSO-d_6 at 499 MHz.

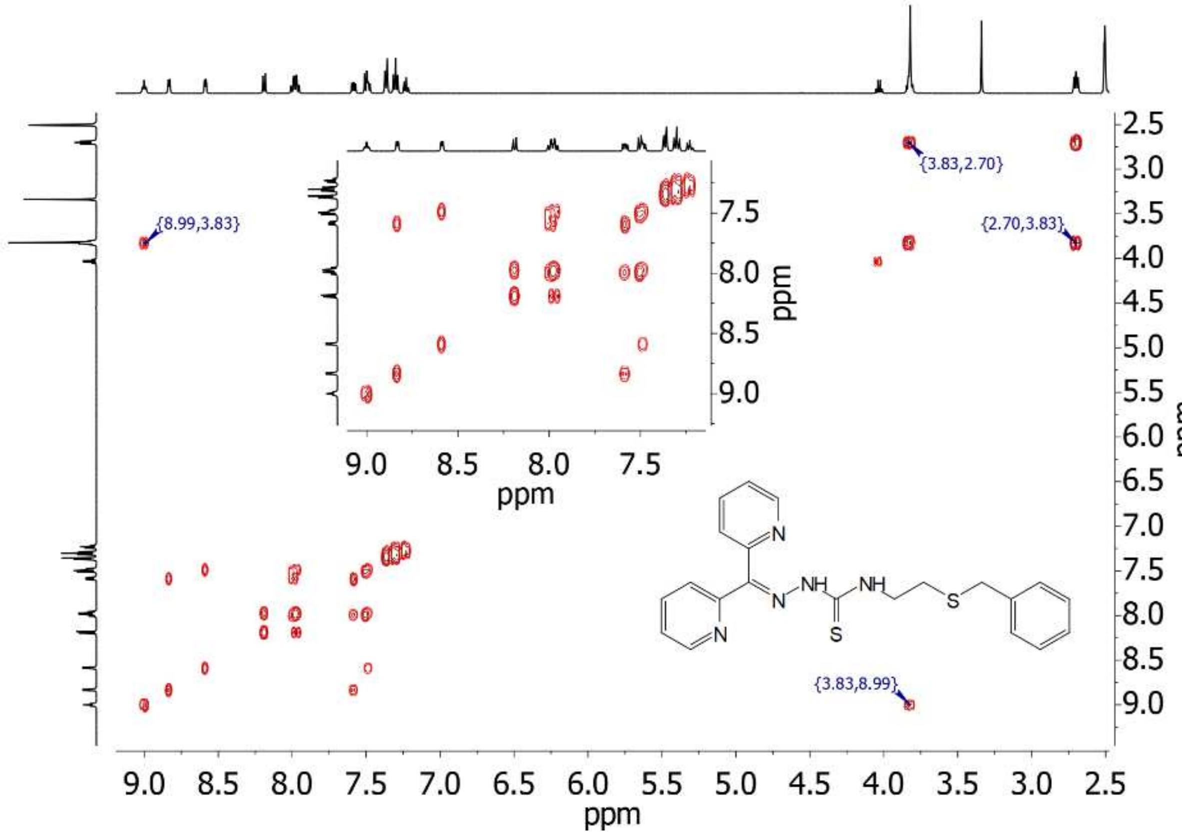

Figure S246. $^{1}H,^{1}H$ COSY NMR spectrum of di-2-pyridylketone-4-(2-benzylsulfanylethyl)-3-thiosemicarbazone in DMSO-d_6 at 499 MHz.

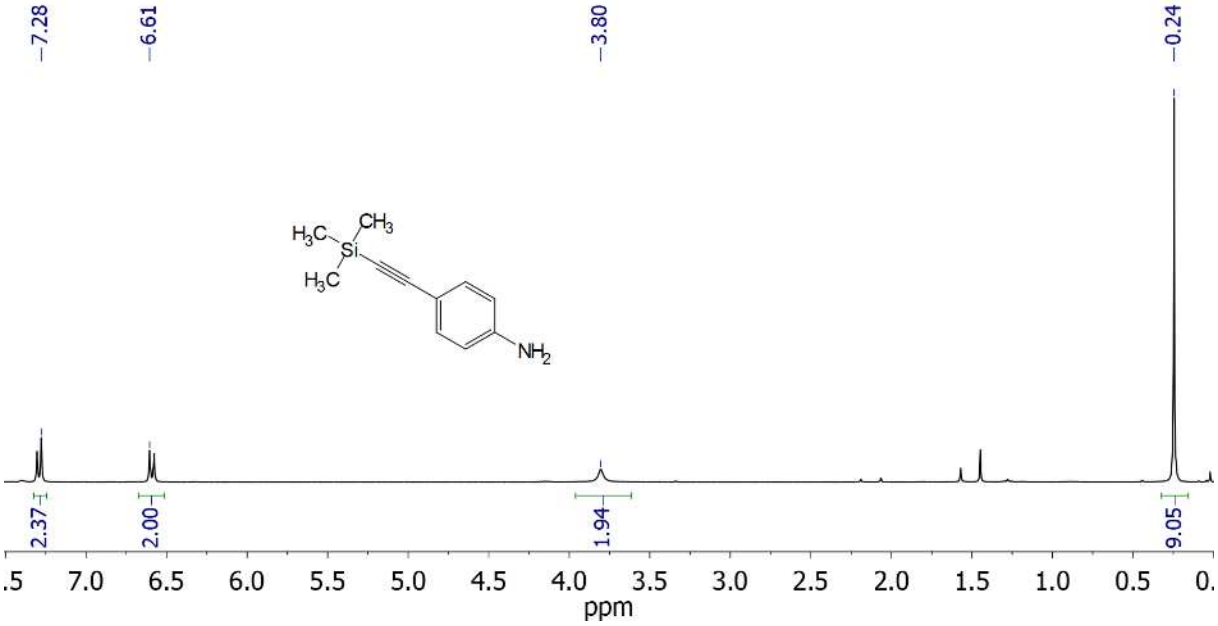

Figure S247. ^{1}H NMR spectrum of 4-[(2-Trimethylsilyl) ethynyl] aniline in $CDCl_3$ at 300 MHz.

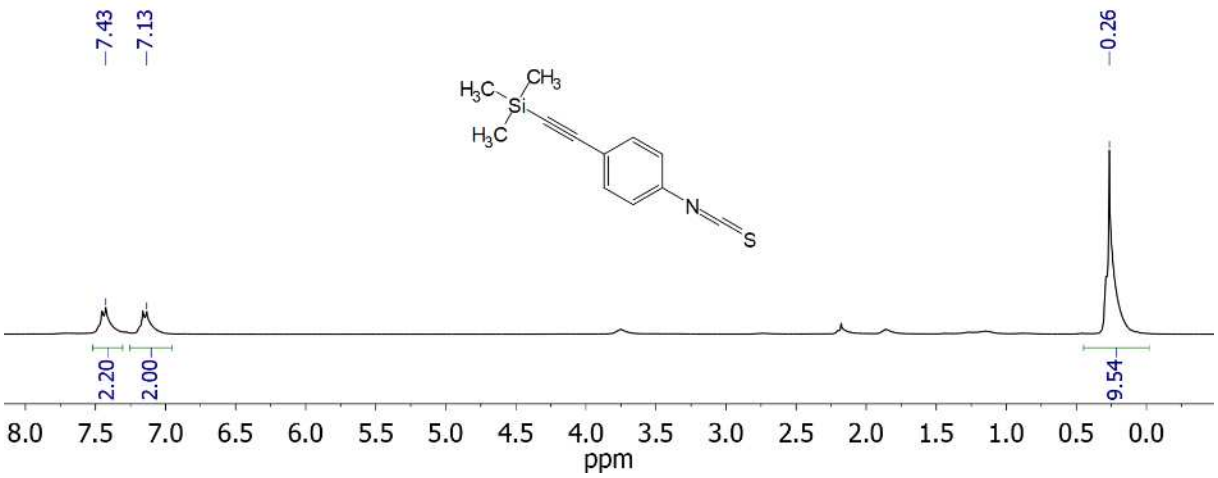

Figure S248. ^{1}H NMR spectrum of 4-[(trimethylsilyl)ethynyl] phenyl isothiocyanate in CDCl$_3$ at 300 MHz.

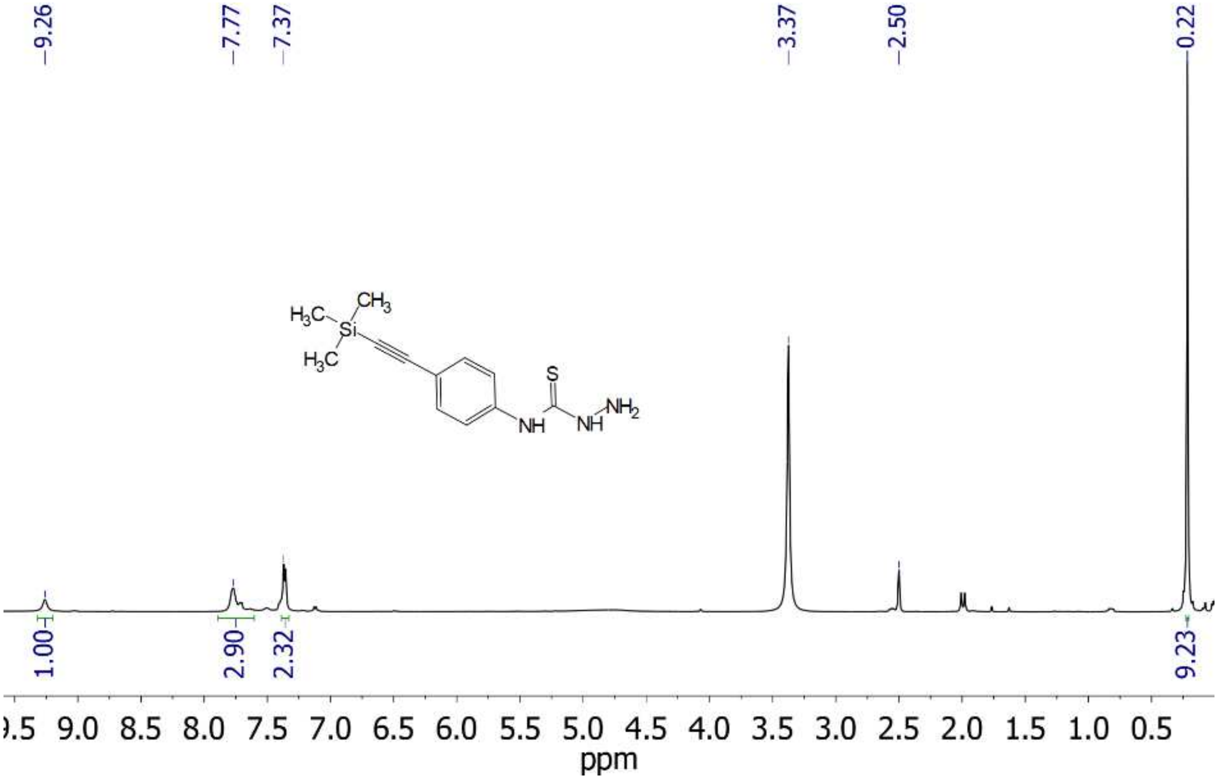

Figure S249. ^{1}H NMR spectrum of *N*-hydrazidecarbothio[4-(trimethylsilyl)ethynylphenyl]amine in DMSO-d_6 at 300 MHz.

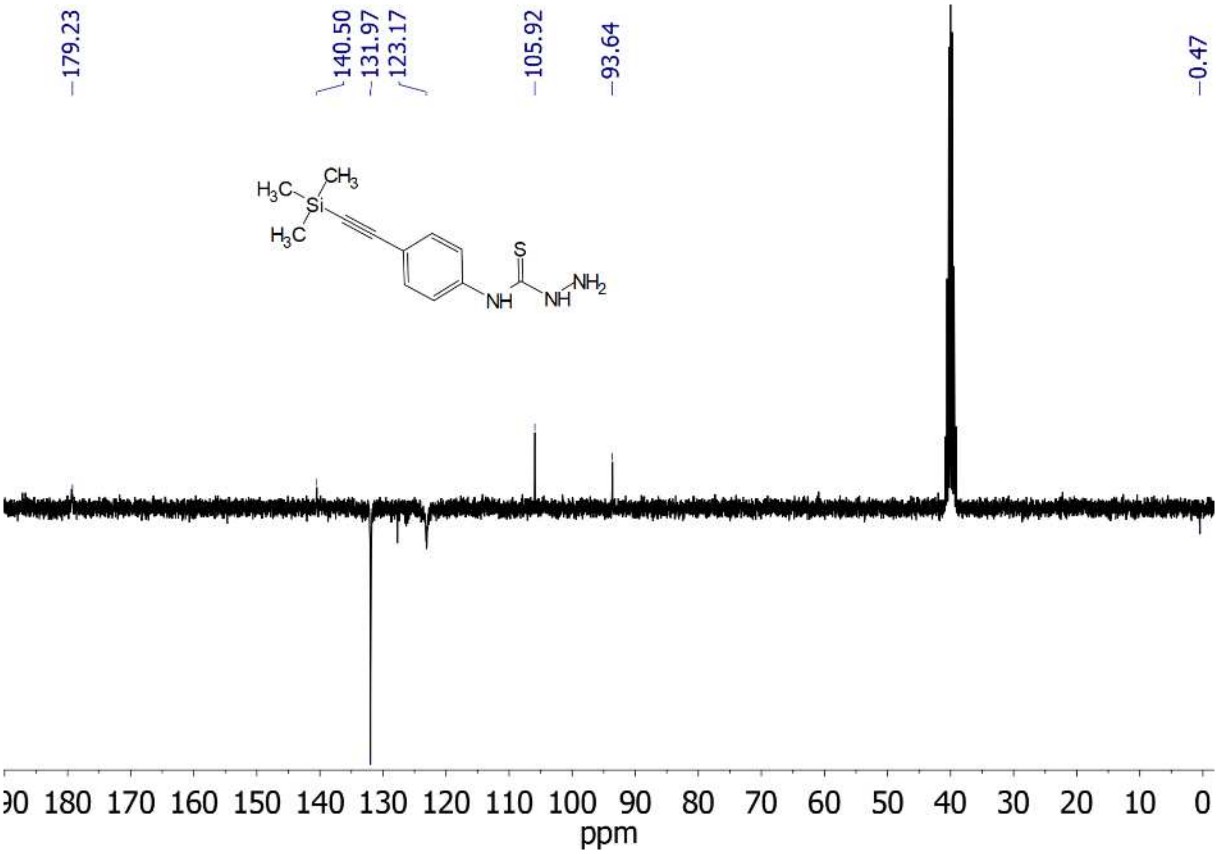

Figure S250. ^{13}C DEPTQ NMR spectrum of *N*-hydrazidecarbothio[4-(trimethylsilyl)ethynylphenyl] amine in DMSO-d_6 at 300 MHz.

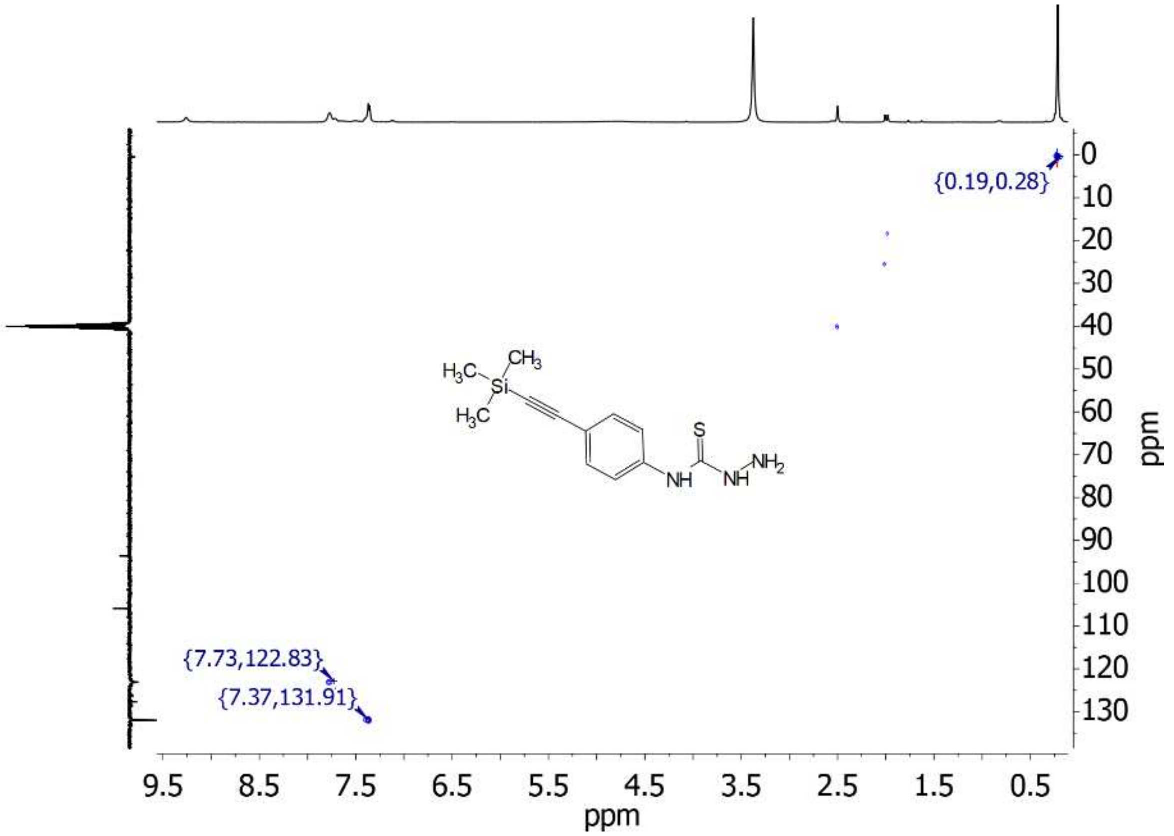

Figure S251. ^{1}H,^{13}C HMQC/HSQC NMR spectrum of *N*-hydrazidecarbothio[4-(trimethylsilyl)ethynyl phenyl]amine in DMSO-d_6 at 300 MHz.

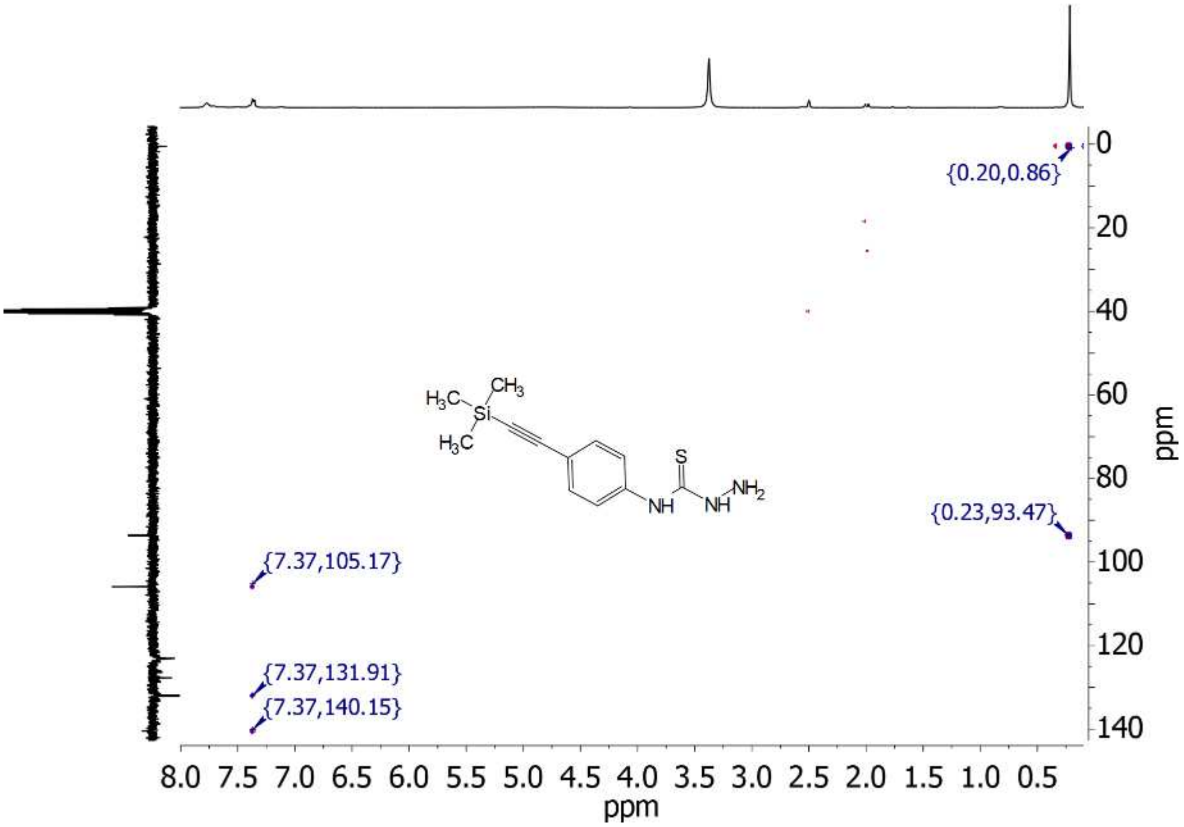

Figure S252. ^{1}H,^{13}C HMBC NMR spectrum of *N*-hydrazidecarbothio[4-(trimethylsilyl)ethynylphenyl] amine in DMSO-d_6 at 300 MHz.

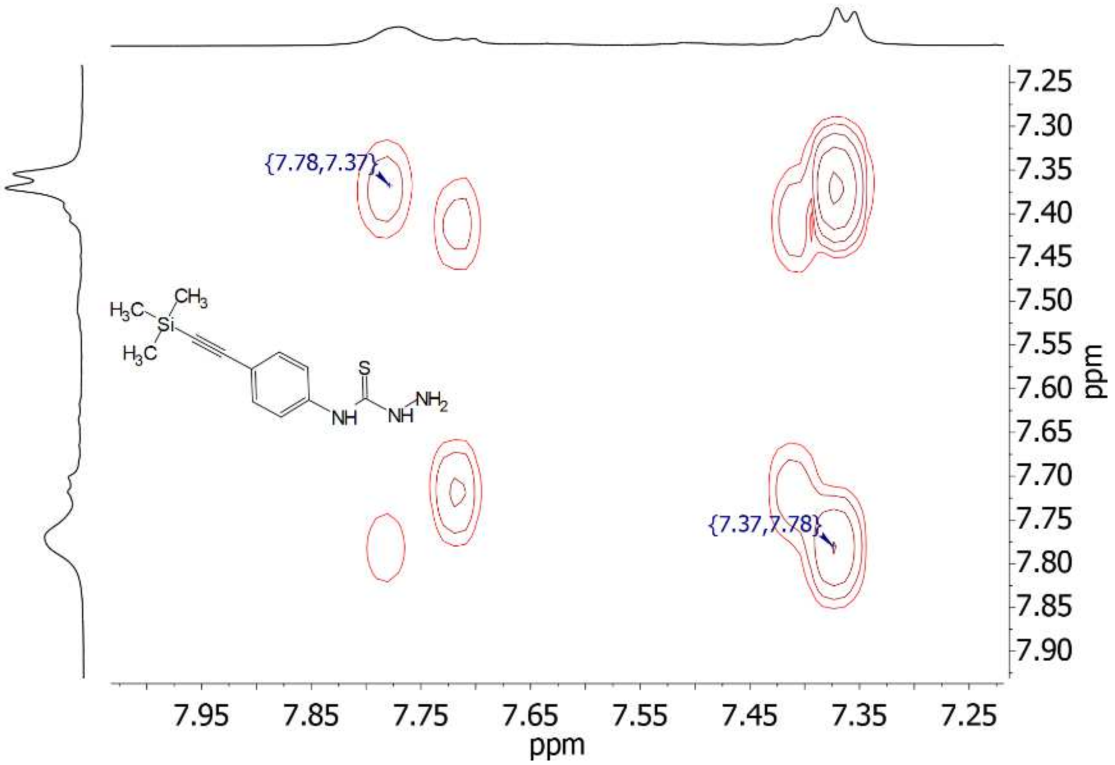

Figure S253. ^{1}H,^{1}H COSY NMR spectrum of *N*-hydrazidecarbothio[4-(trimethylsilyl)ethynylphenyl] amine in DMSO-d_6 at 300 MHz.

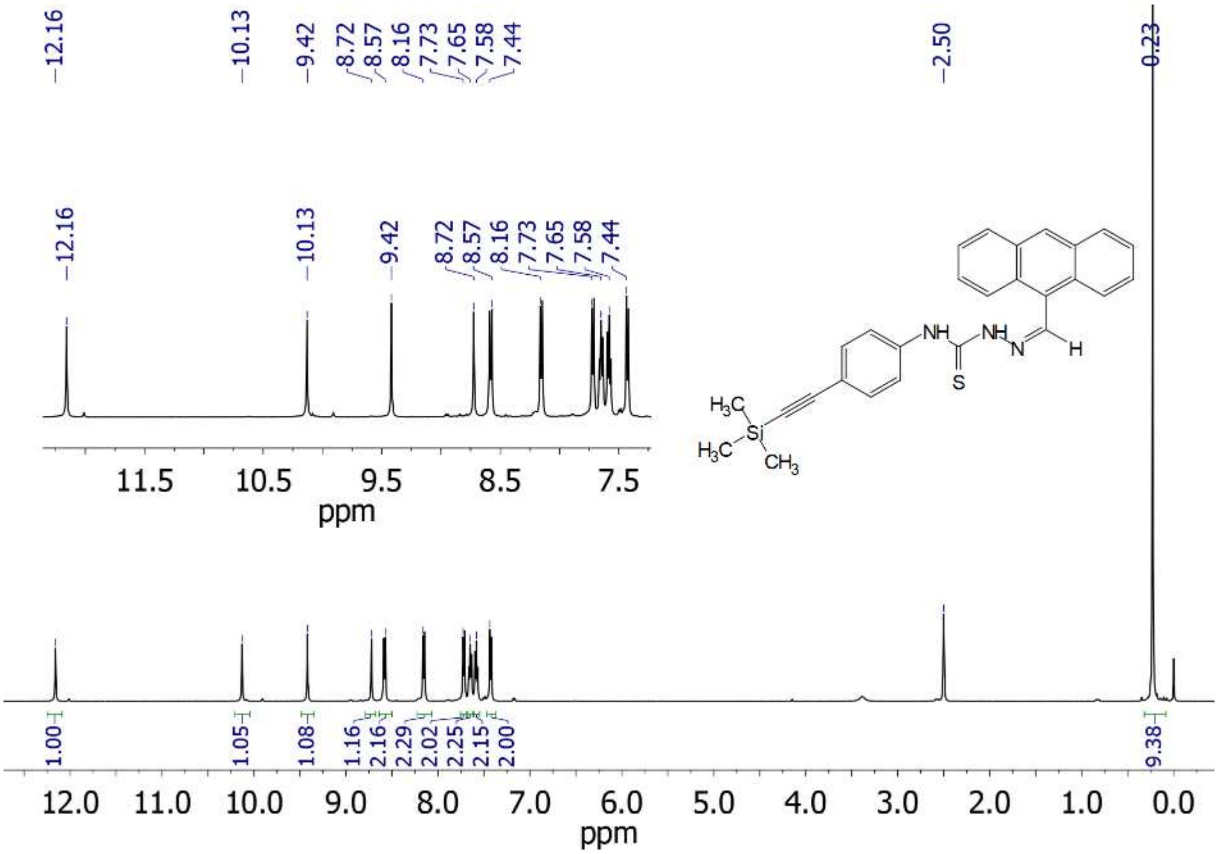

Figure S254. ^{1}H NMR spectrum of [(1*E*)-(anthracen-9-yl)methylideneamino][4-(trimethylsilyl)ethynyl-phenylamino] carbothioamide in DMSO-d_6 at 499 MHz.

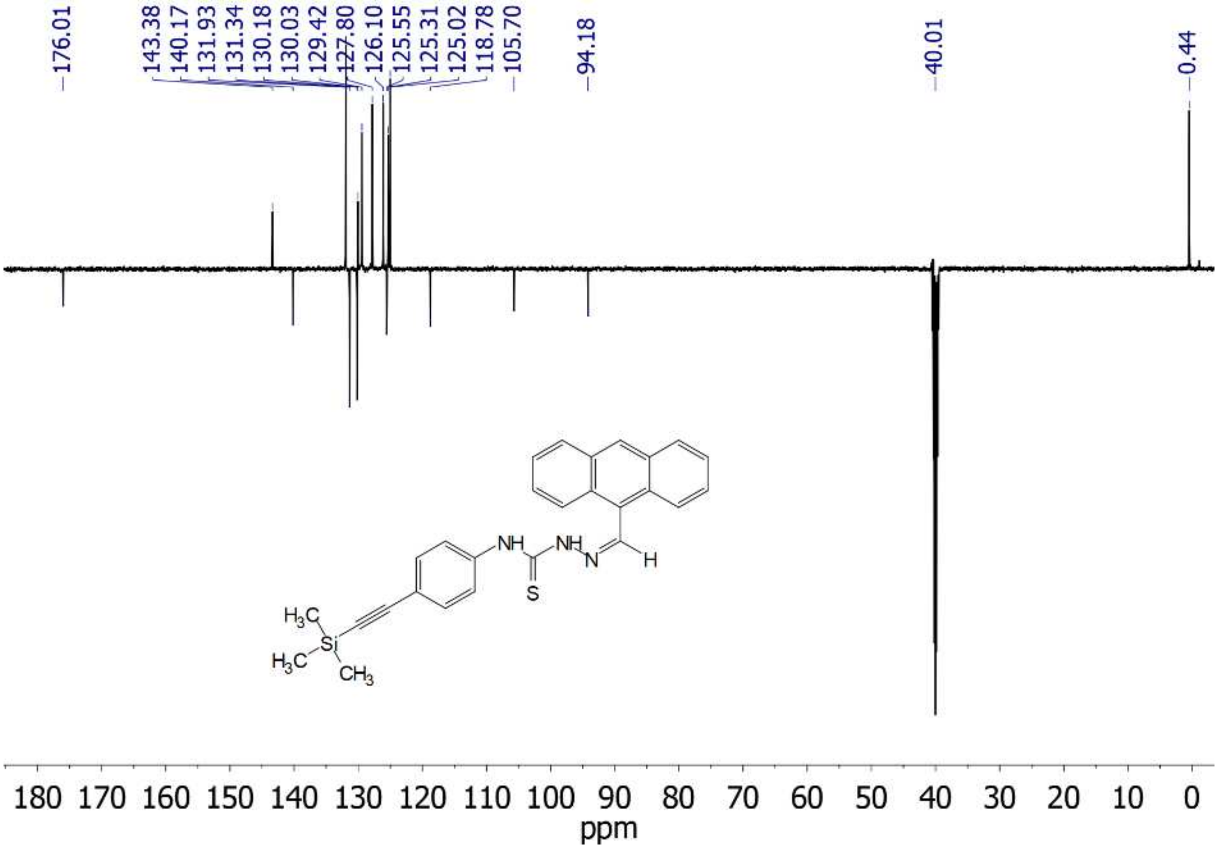

Figure S255. ^{13}C APT NMR spectrum of [(1*E*)-(anthracen-9-yl)methylideneamino][4-(trimethylsilyl)-ethynylphenylamino] carbothioamide in DMSO-d_6 at 499 MHz.

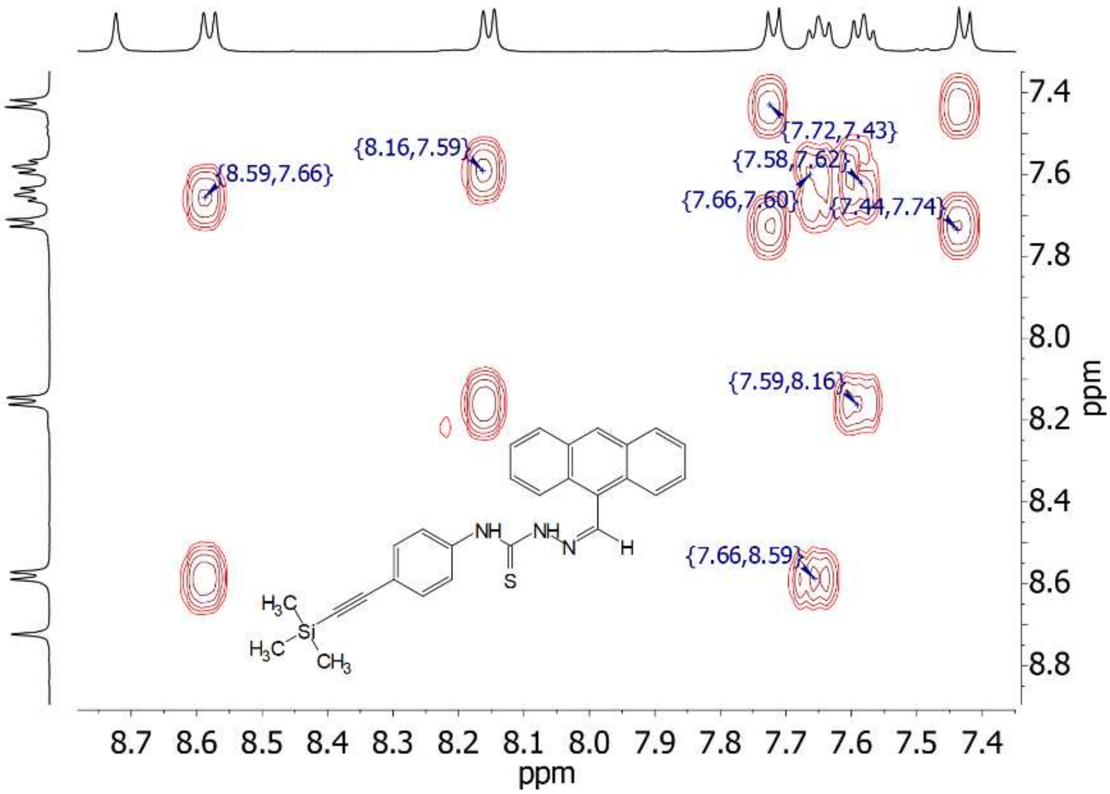

Figure S256. ^1H,^1H COSY NMR spectrum of [(1*E*)-(anthracen-9-yl)methylideneamino][4-(trimethylsilyl)ethynylphenylamino] carbothioamide in DMSO-*d*6 at 499 MHz.

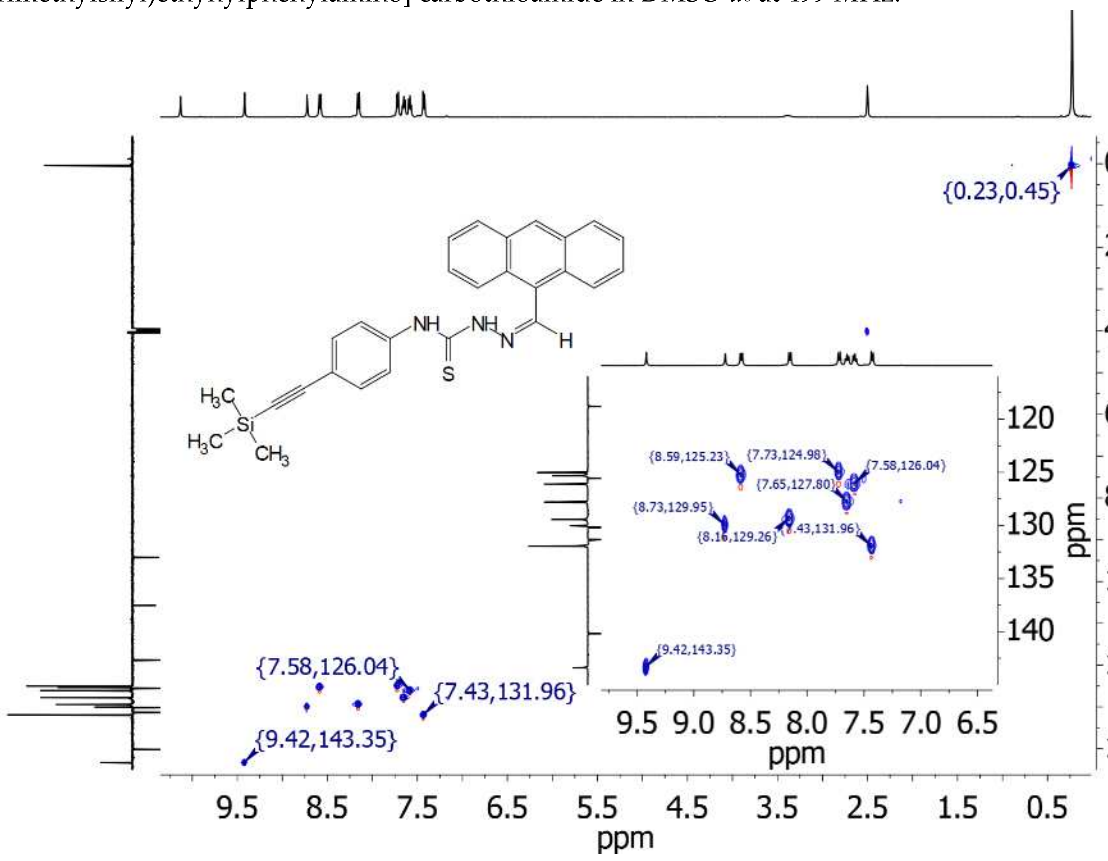

Figure S257. ^1H,^{13}C HMQC/HSQC NMR spectrum of [(1*E*)-(anthracen-9-yl)methylideneamino][4-(trimethylsilyl)ethynylphenylamino] carbothioamide in DMSO-*d*6 at 499 MHz.

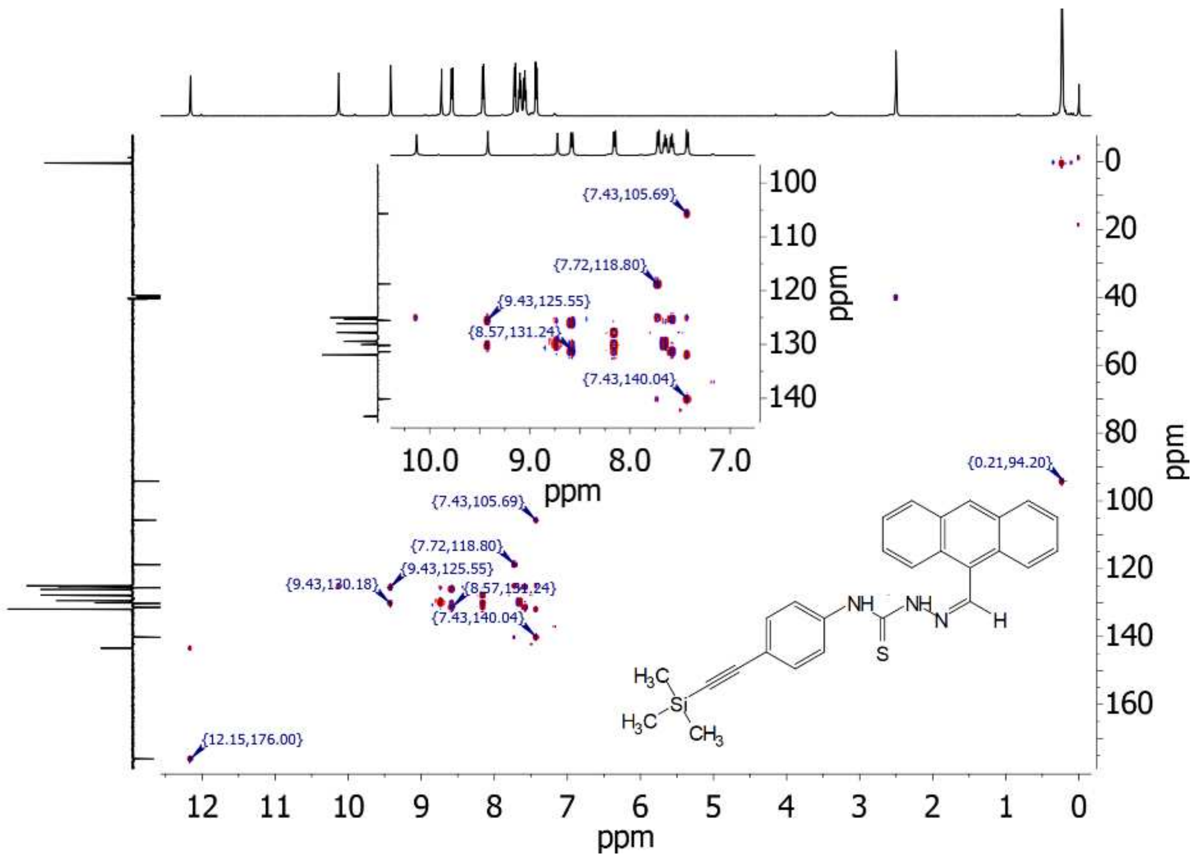

Figure S258. ^{1}H,^{13}C HMBC NMR spectrum of [(1*E*)-(anthracen-9-yl)methylideneamino][4-(trimethylsilyl)ethynylphenylamino] carbothioamide in DMSO-d_6 at 499 MHz.

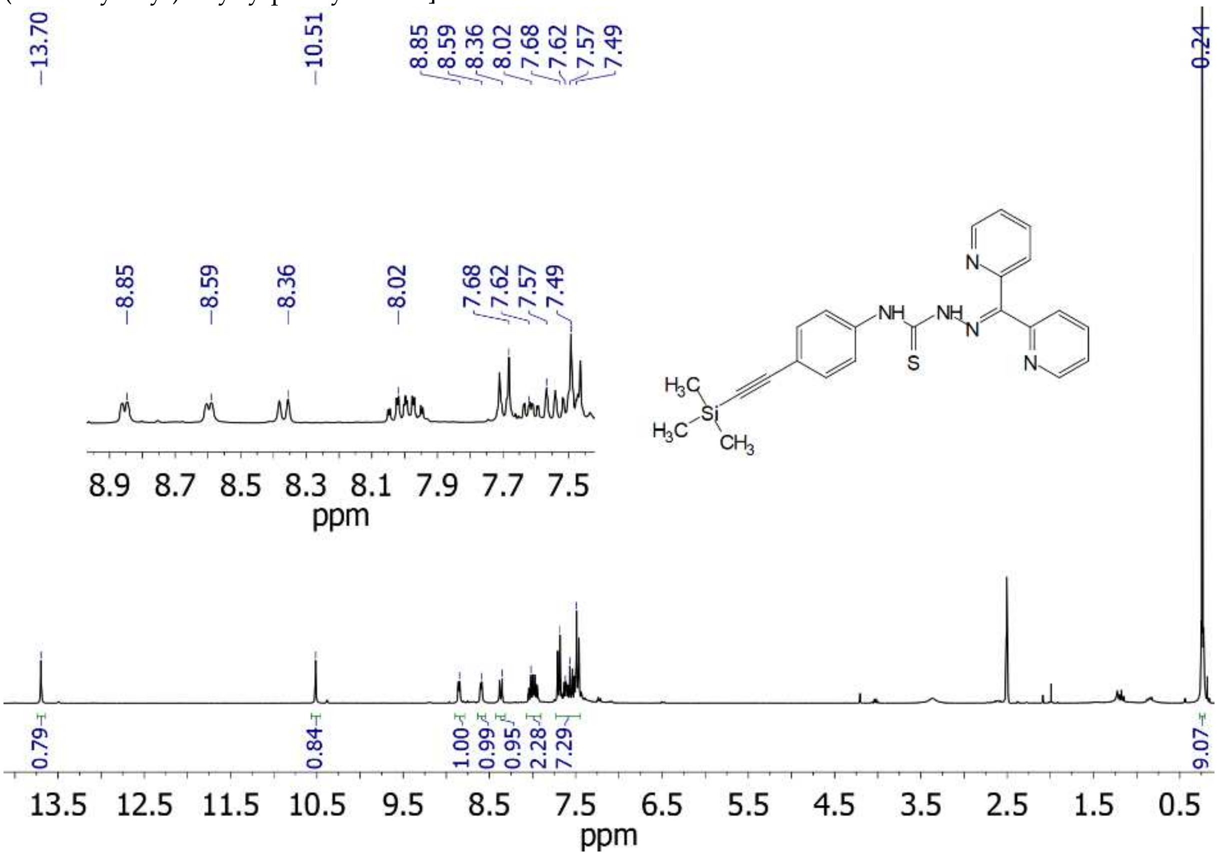

Figure S259. ^{1}H NMR spectrum of [bis(pyridin-2-yl)methylideneamino][4-(trimethylsilyl) ethynylphenylamino]carbothioamide in DMSO-d_6 at 300 MHz.

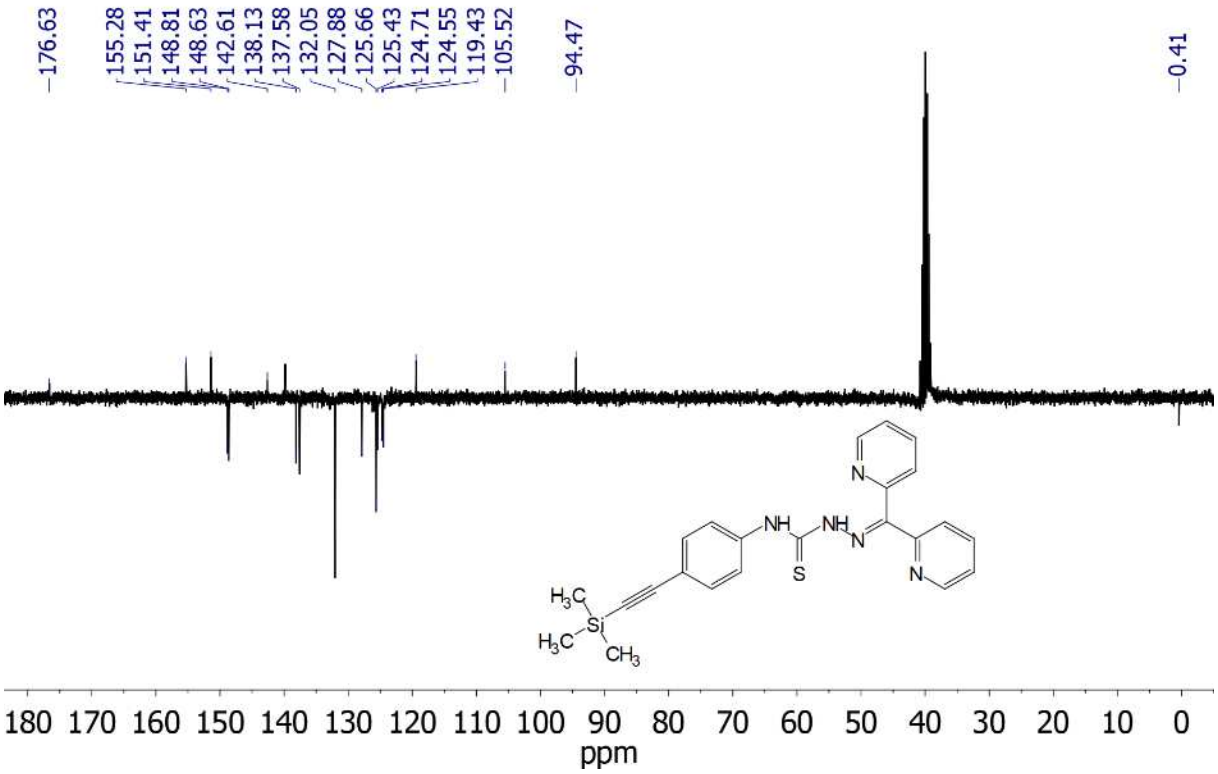

Figure S260. ^{13}C DEPTQ NMR spectrum of [bis(pyridin-2-yl)methylideneamino][4-(trimethylsilyl) ethynylphenylamino]carbothioamide in DMSO-d_6 at 300 MHz.

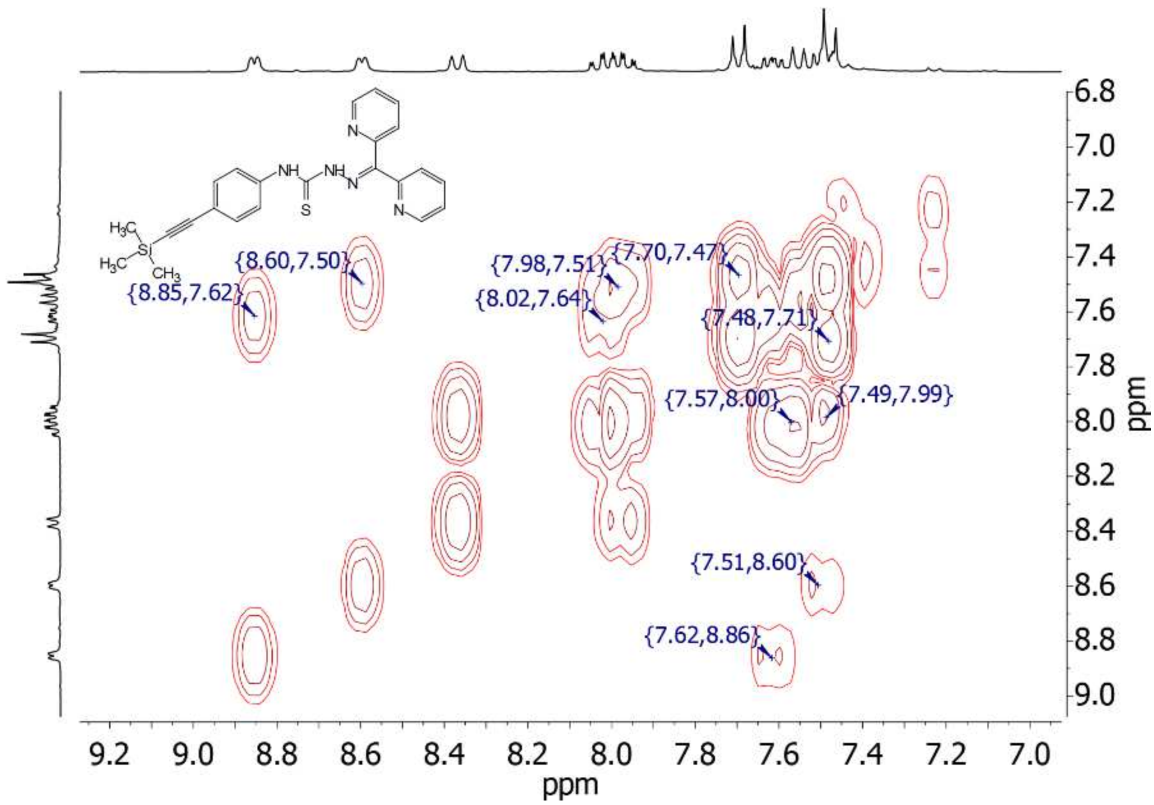

Figure S261. ^{1}H,^{1}H COSY NMR spectrum of [bis(pyridin-2-yl)methylideneamino][4-(trimethylsilyl) ethynylphenylamino]carbothioamide in DMSO-d_6 at 300 MHz.

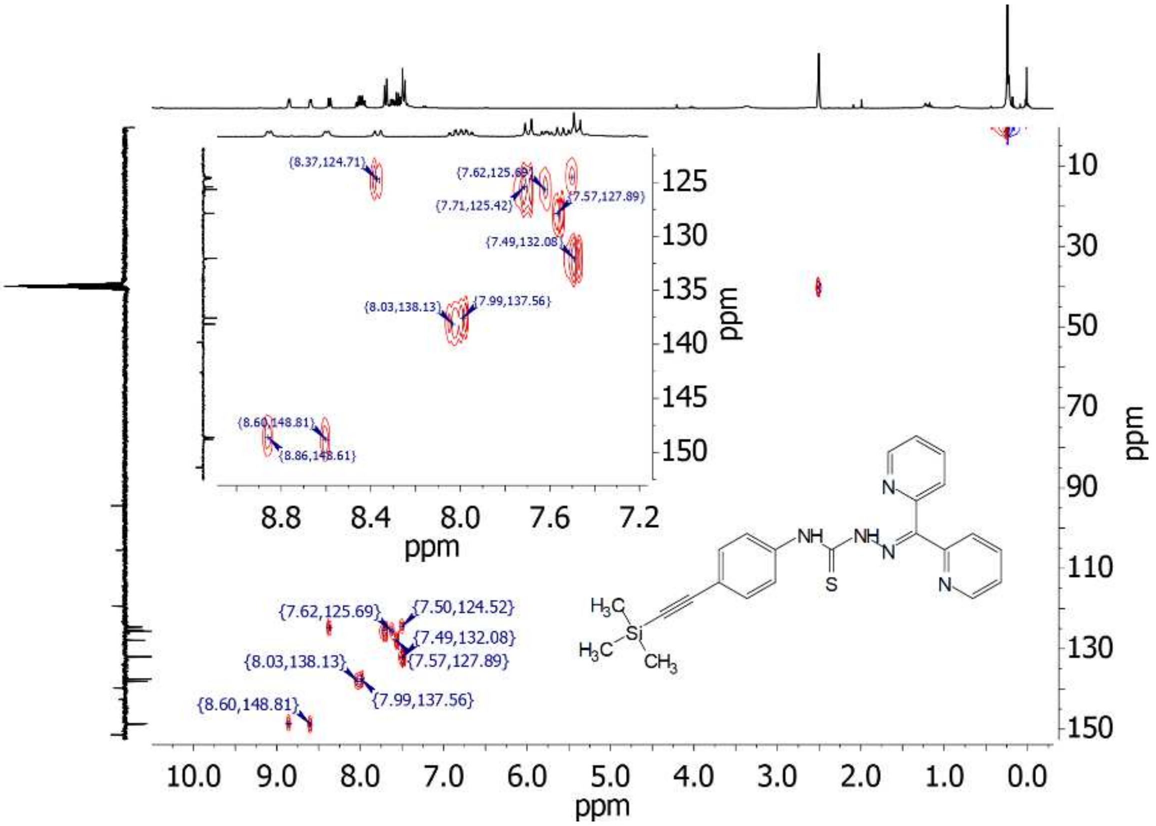

Figure S262. $^{1}H,^{13}C$ HMQC/HSQC NMR spectrum of [bis(pyridin-2-yl)methylideneamino][4-(trimethylsilyl)ethynylphenylamino]carbothioamide in DMSO-d_6 at 300 MHz.

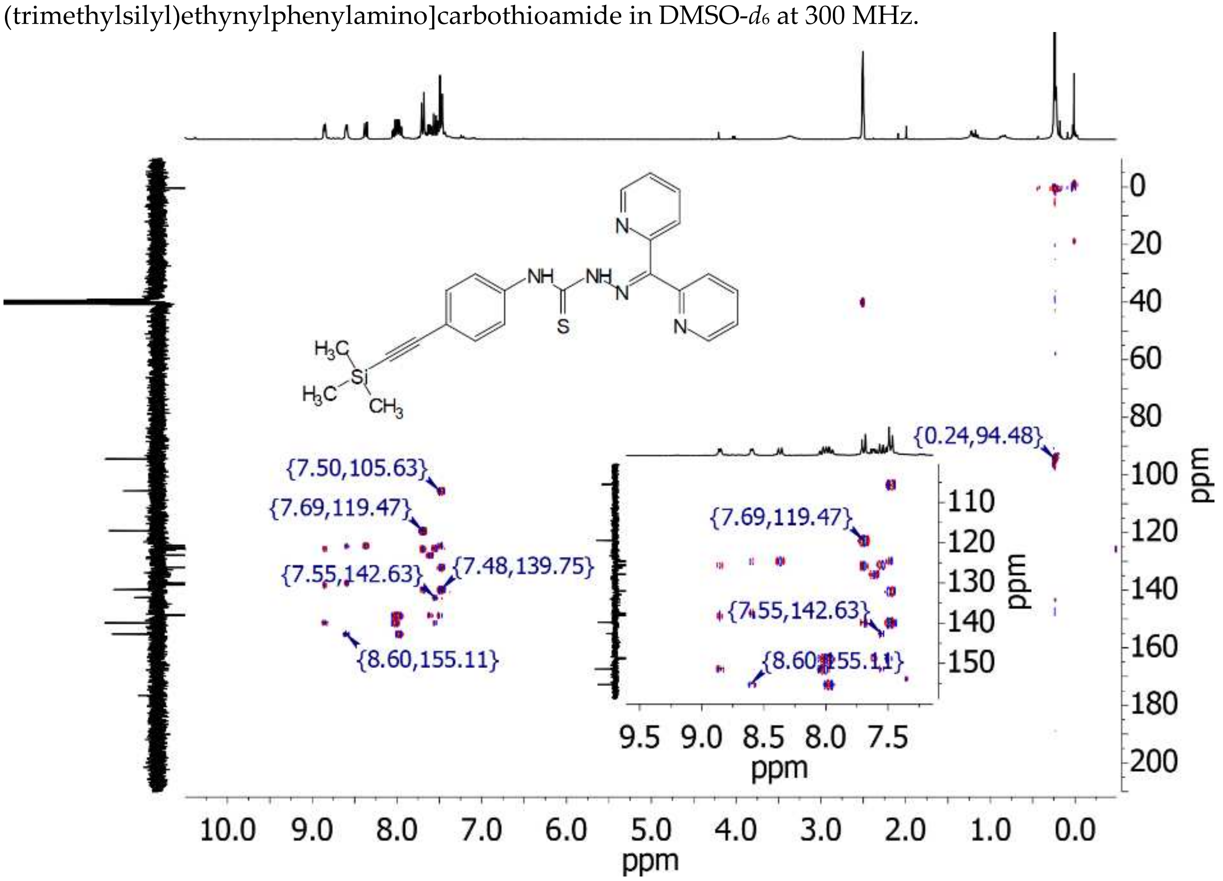

Figure S263. $^{1}H,^{13}C$ HMBC NMR spectrum of [bis(pyridin-2-yl)methylideneamino][4-(trimethylsilyl)ethynylphenylamino]carbothioamide in DMSO-d_6 at 300 MHz.

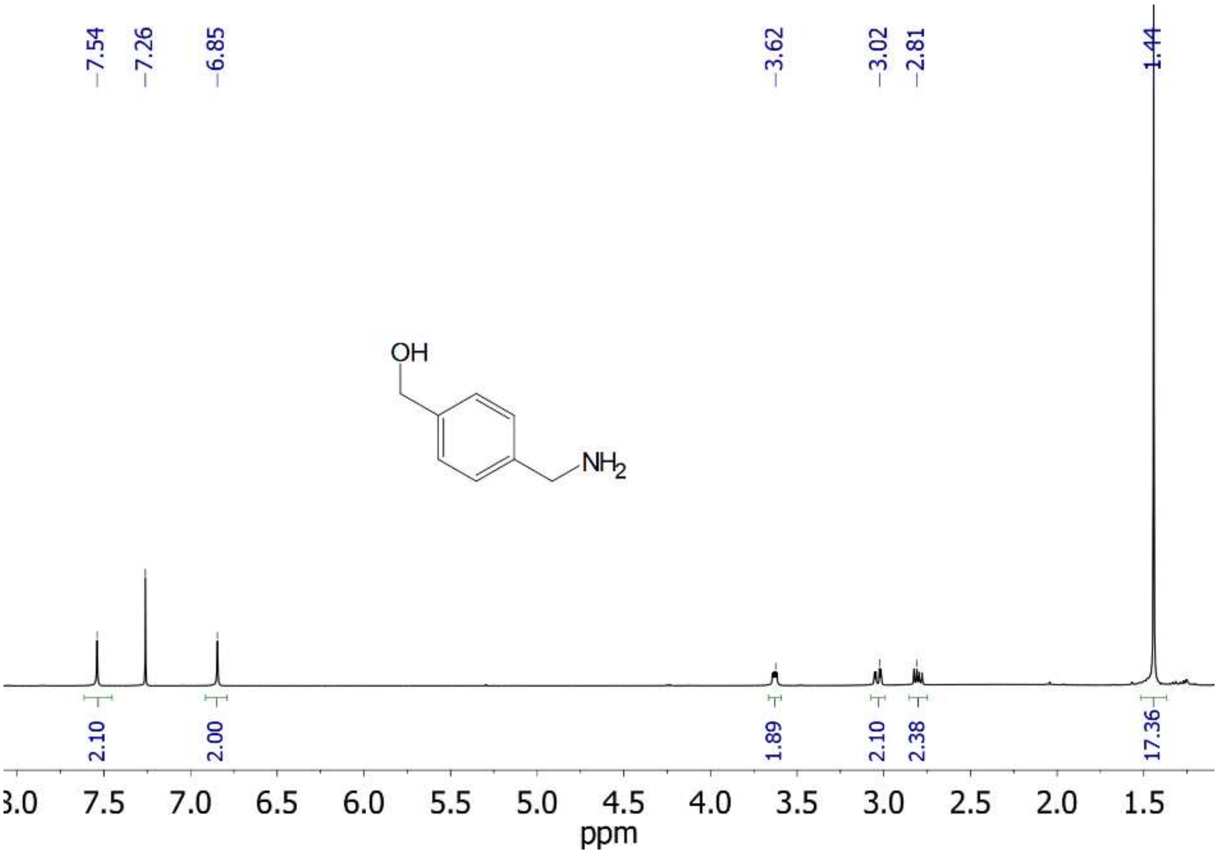

Figure S264. ^{1}H NMR spectrum of 4-(aminomethyl)benzyl alcohol in $CDCl_3$ at 499 MHz.

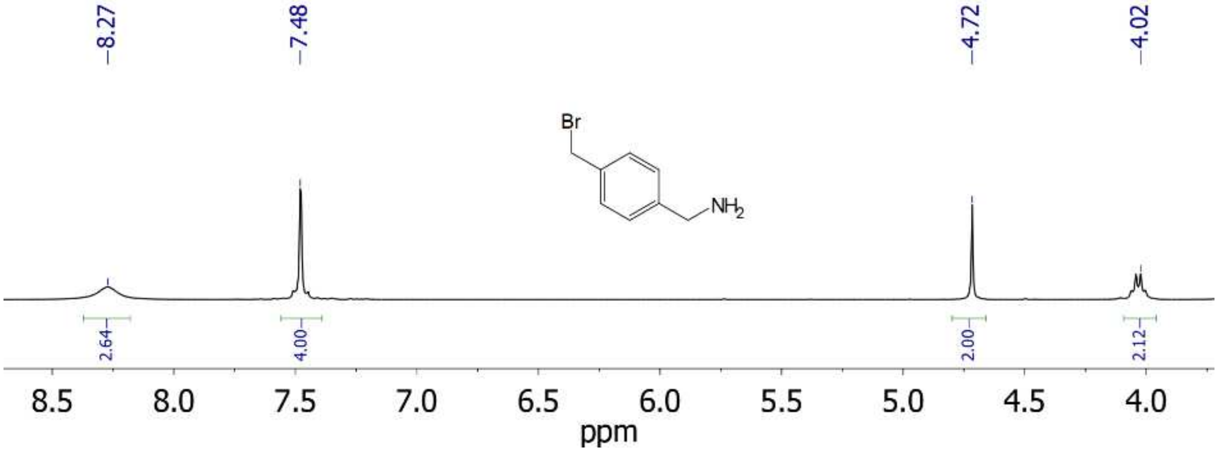

Figure S265. ^{1}H NMR spectrum of 4-(bromomethyl)benzylamine hydrobromide in DMSO-d_6 at 300 MHz.

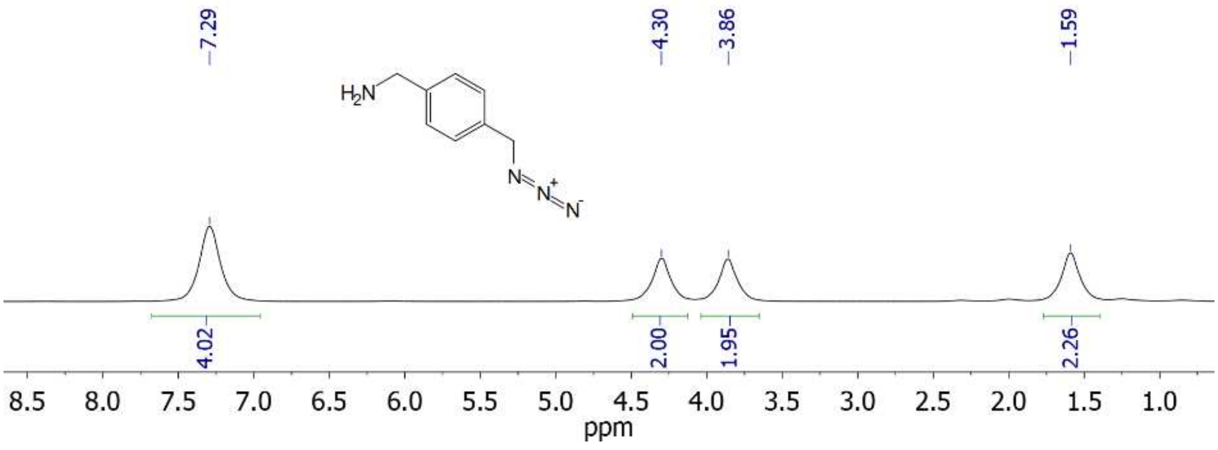

Figure S266. ^{1}H NMR spectrum of 4-(azidomethyl)benzylamine in DMSO-d_6 at 300 MHz

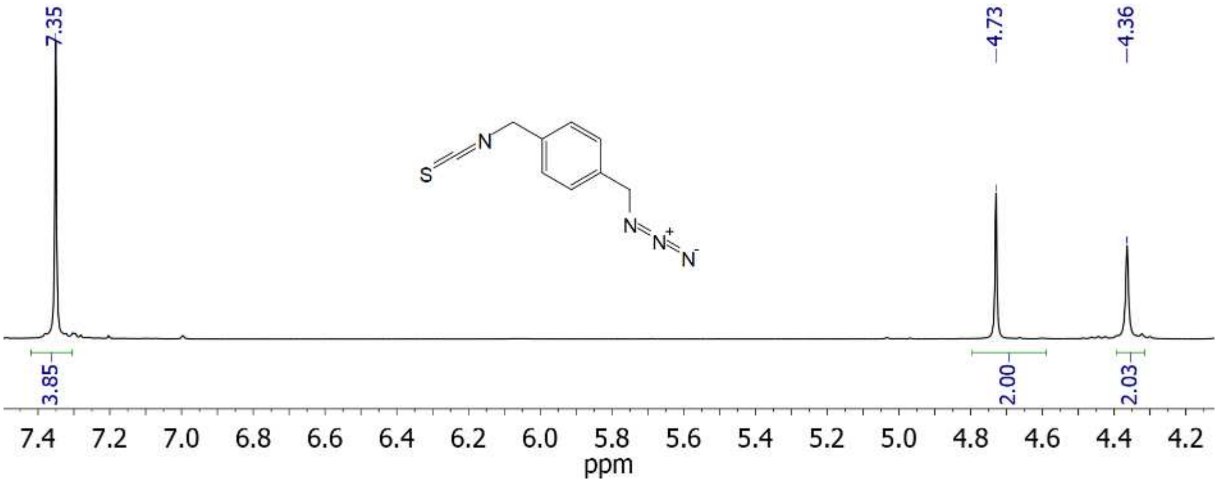

Figure S267. ^{1}H NMR spectrum of 1-(azidomethyl)-4-(isothiocyanatomethyl)benzene in DMSO-d_6 at 300 MHz.

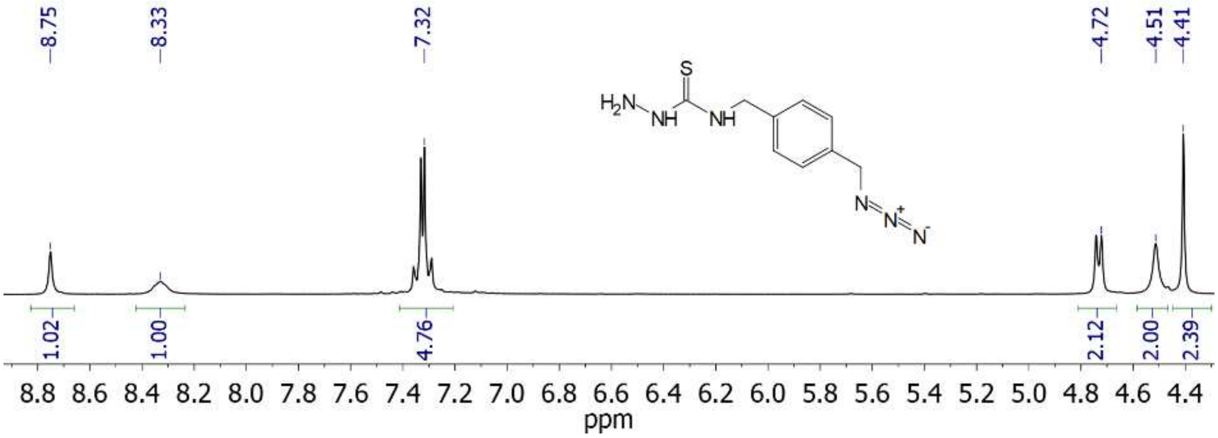

Figure **S268.** ^{1}H NMR spectrum of 4-{[*p*-(azidomethyl)phenyl]methyl}thiosemicarbazide in DMSO-d_6 at 499 MHz.

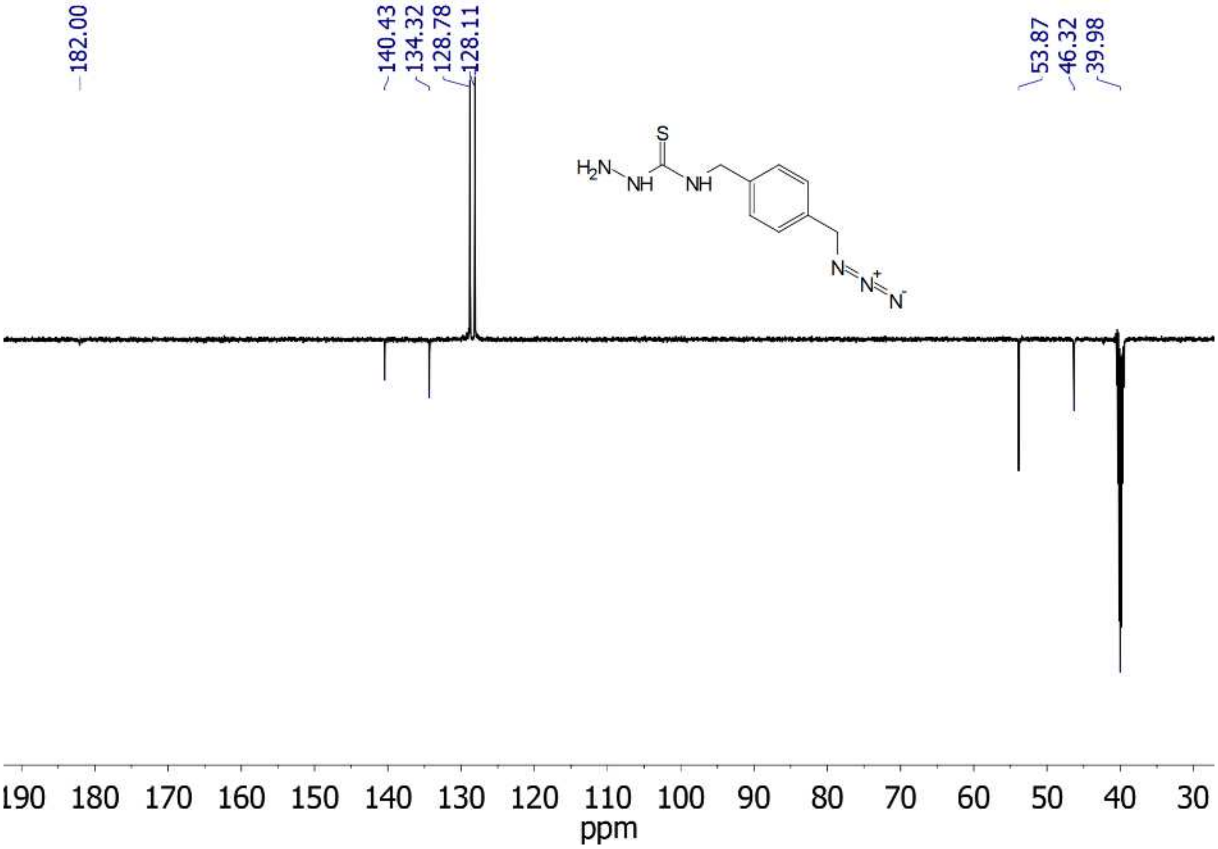

Figure S269. ^{13}C APT NMR spectrum of 4-{[*p*-(azidomethyl)phenyl]methyl}thiosemicarbazide in DMSO-d_6 at 499 MHz.

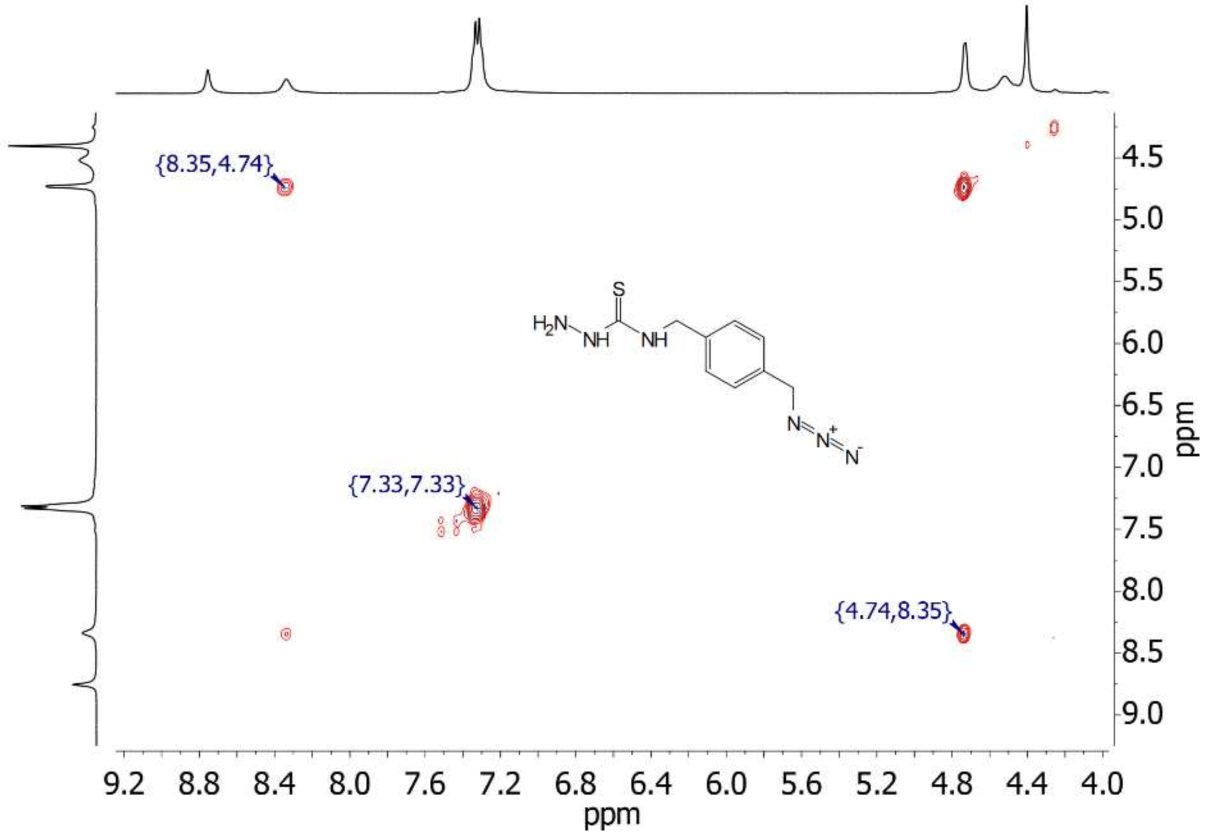

Figure S270. $^1H,^1H$ COSY NMR spectrum of 4-{[*p*-(azidomethyl)phenyl]methyl}thiosemicarbazide in DMSO-d_6 at 499 MHz.

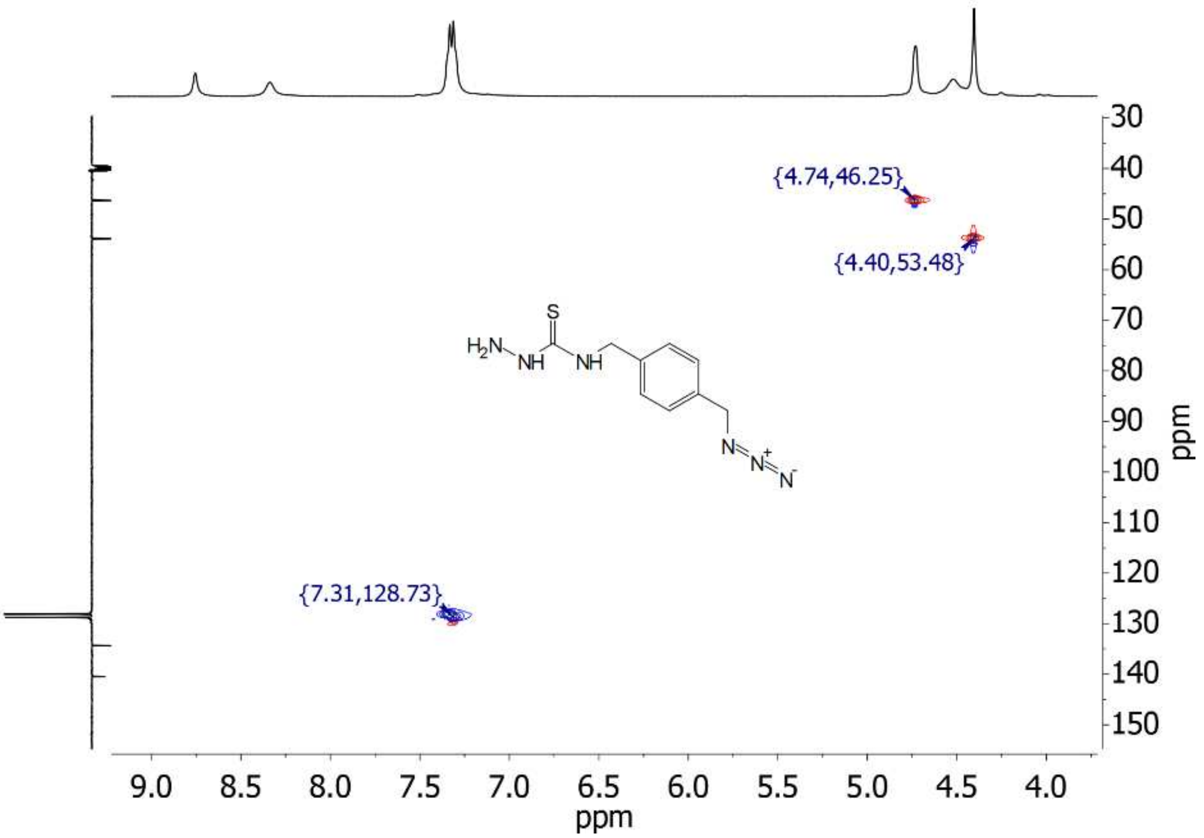

Figure S271. ^1H,^{13}C HMQC/HSQC NMR spectrum of 4-{[*p*-(azidomethyl)phenyl]methyl} thiosemicarbazide in DMSO-d_6 at 499 MHz.

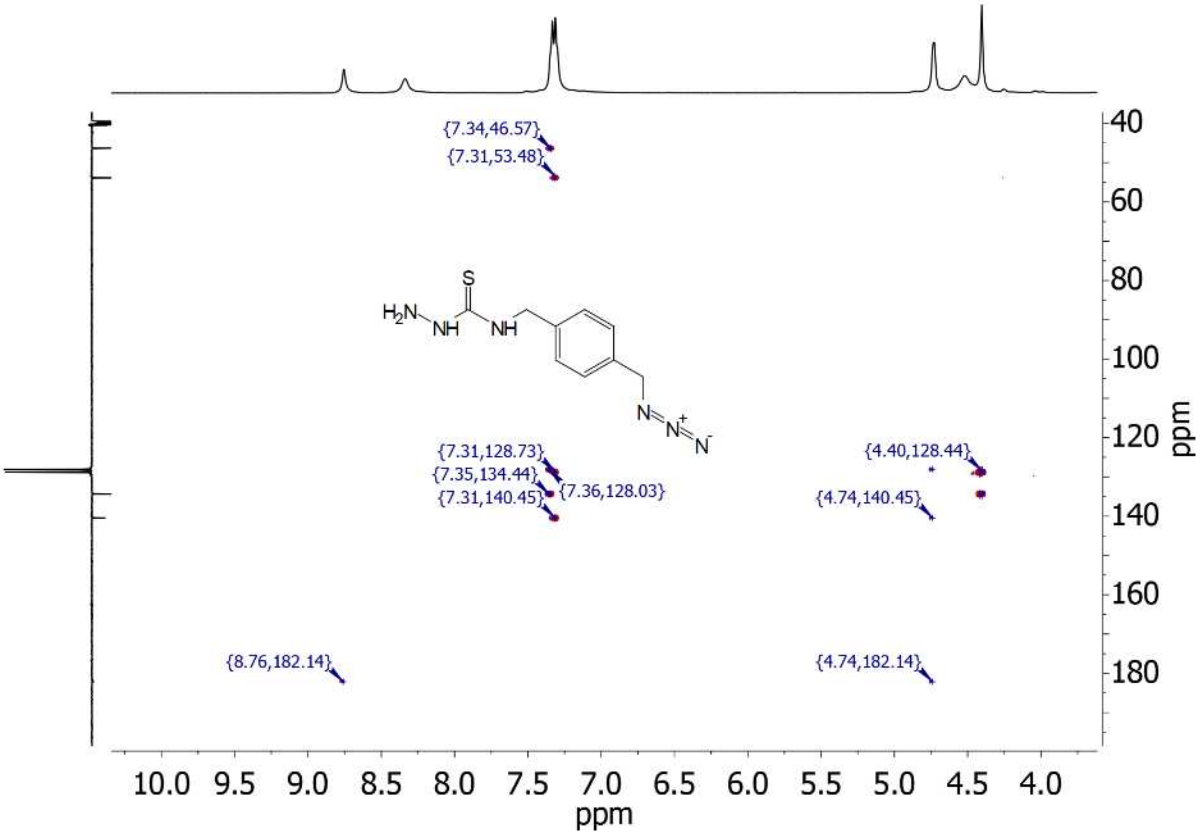

Figure S272. ^1H,^{13}C HMBC NMR spectrum of 4-{[*p*-(azidomethyl)phenyl]methyl}thiosemicarbazide in DMSO-d_6 at 499 MHz.

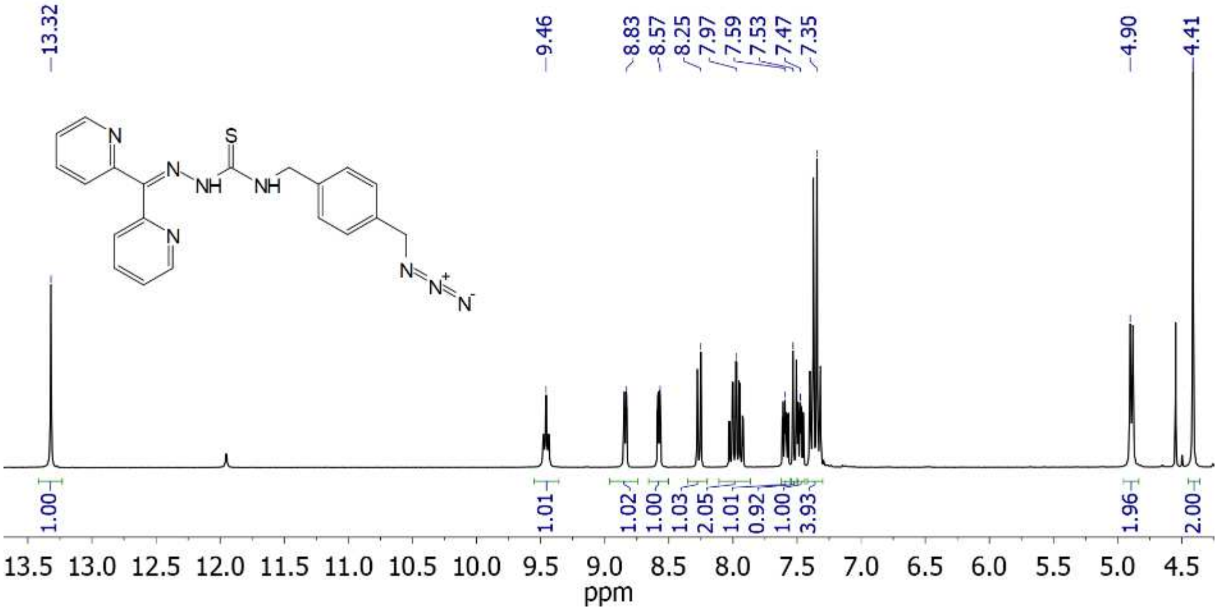

Figure S273. ^{1}H NMR spectrum of {2-[bis(pyridin-2-yl)methylidene]hydrazino}-[4-(azidomethyl) phenyl]ethanethioamide in DMSO-d_6 at 499 MHz.

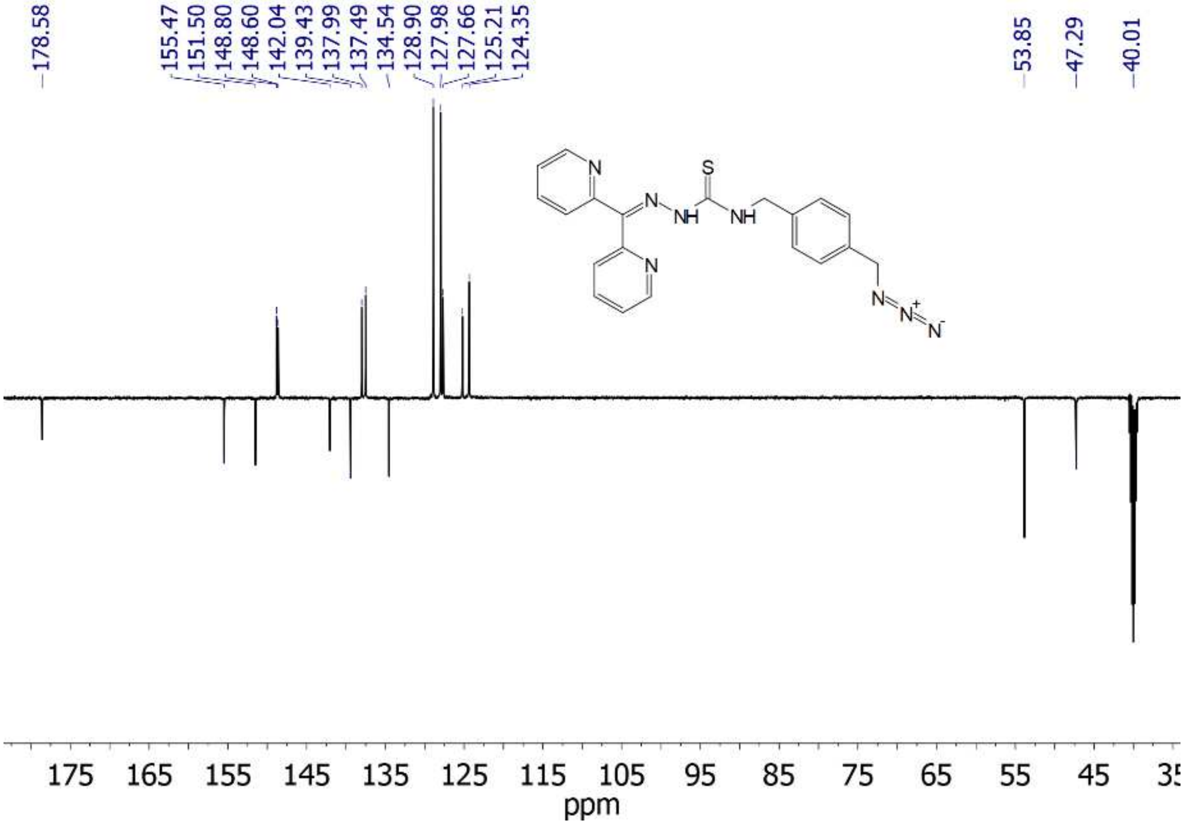

Figure S274. ^{13}C APT NMR spectrum of {2-[bis(pyridin-2-yl)methylidene]hydrazino}-[4-(azidomethyl) phenyl]ethanethioamide in DMSO-d_6 at 499 MHz.

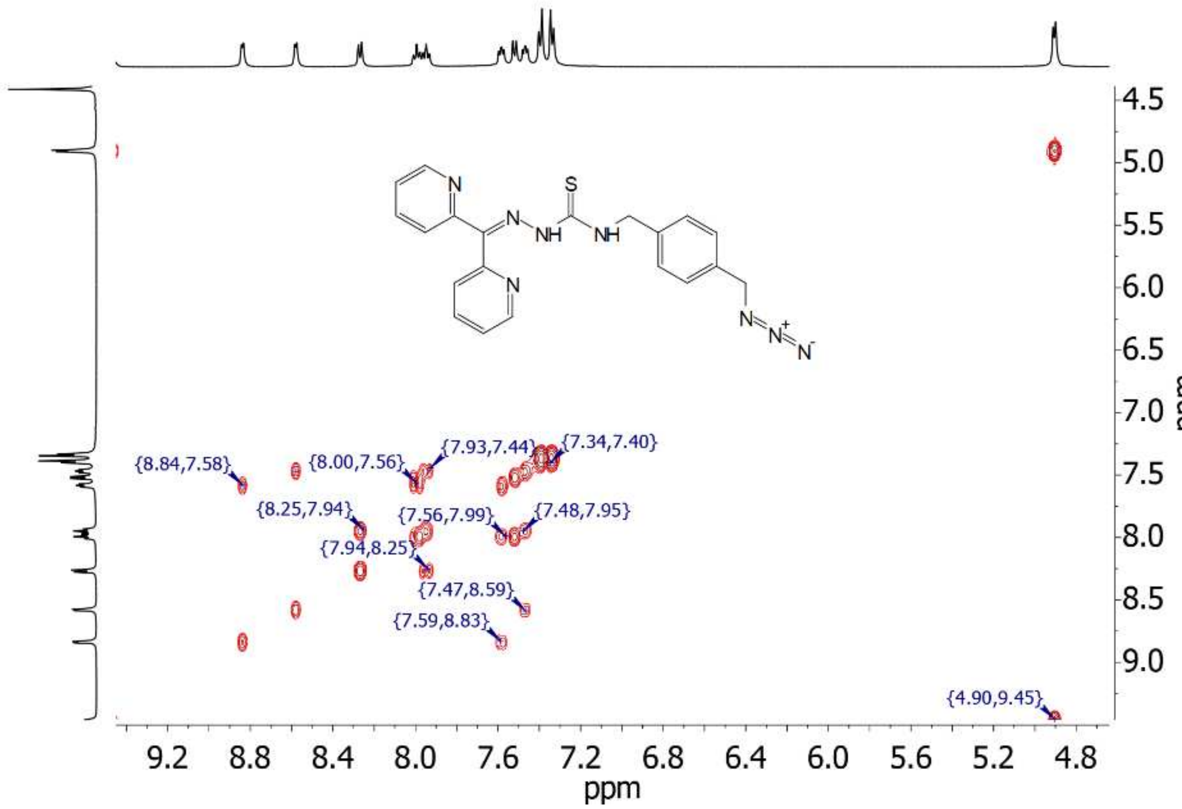

Figure S275. $^{1}H,^{1}H$ COSY NMR spectrum of {2-[bis(pyridin-2-yl)methylidene]hydrazino}-[4-(azidomethyl)phenyl]ethanethioamide in DMSO-d_6 at 499 MHz.

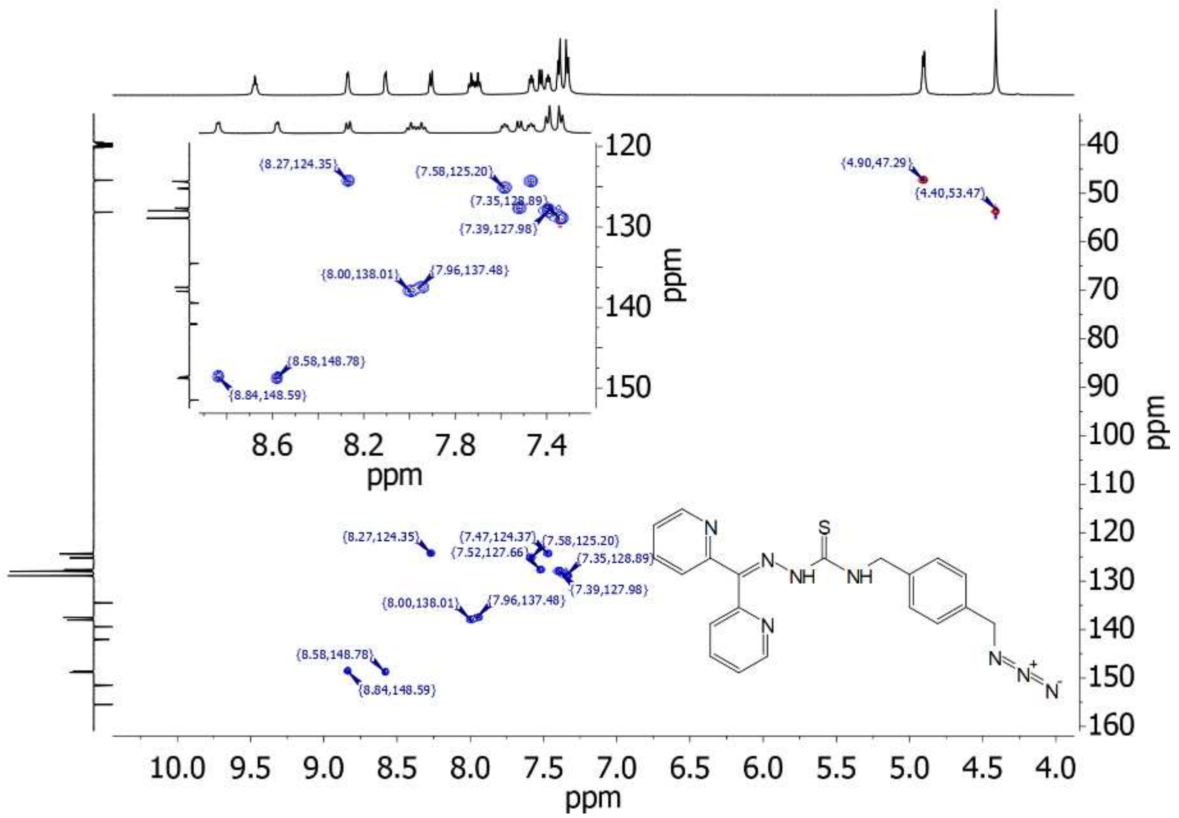

Figure S276. $^{1}H,^{13}C$ HMQC/HSQC NMR spectrum of {2-[bis(pyridin-2-yl)methylidene]hydrazino}-[4-(azidomethyl)phenyl]ethanethioamide in DMSO-d_6 at 499 MHz.

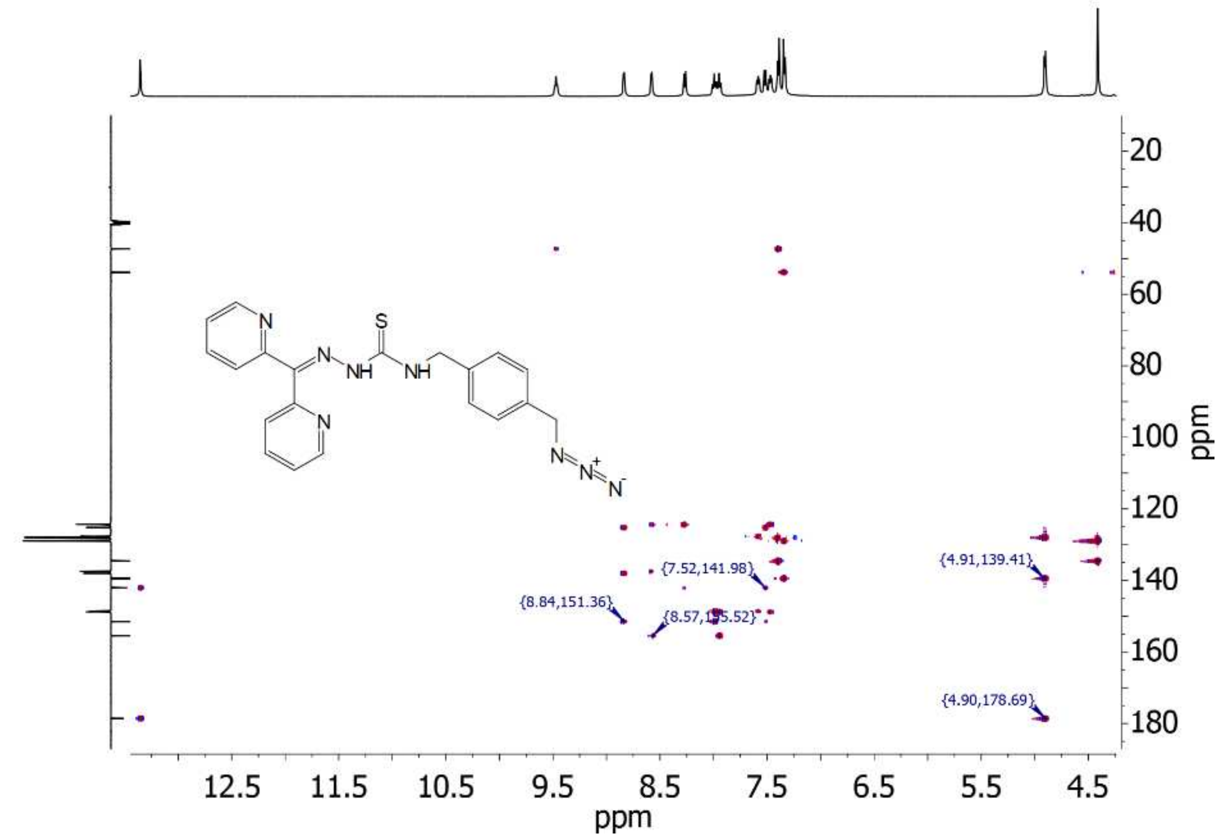

Figure S277. ^1H,^{13}C HMBC NMR spectrum of {2-[bis(pyridin-2-yl)methylidene]hydrazino}-[4-(azidomethyl)phenyl]ethanethioamide in DMSO-d_6 at 499 MHz.

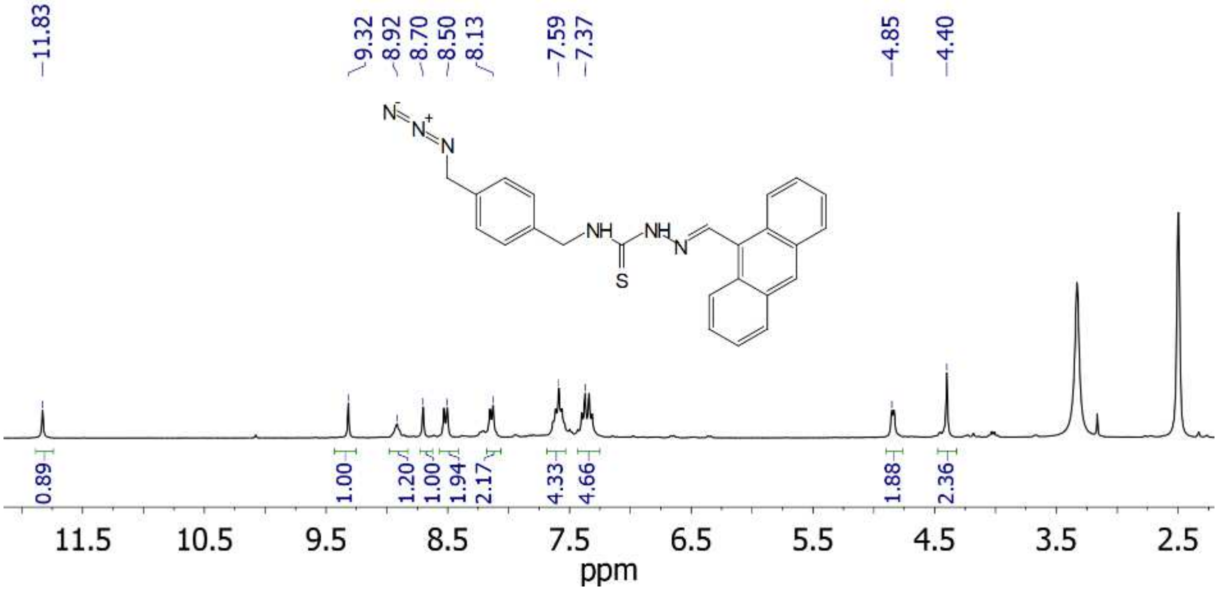

Figure S278. ^1H NMR spectrum of [(2*E*)-2-[(anthracen-9-yl)methylidene]hydrazino]-[4-(azidomethyl) phenyl]ethanethioamide in DMSO-d_6 at 300 MHz.

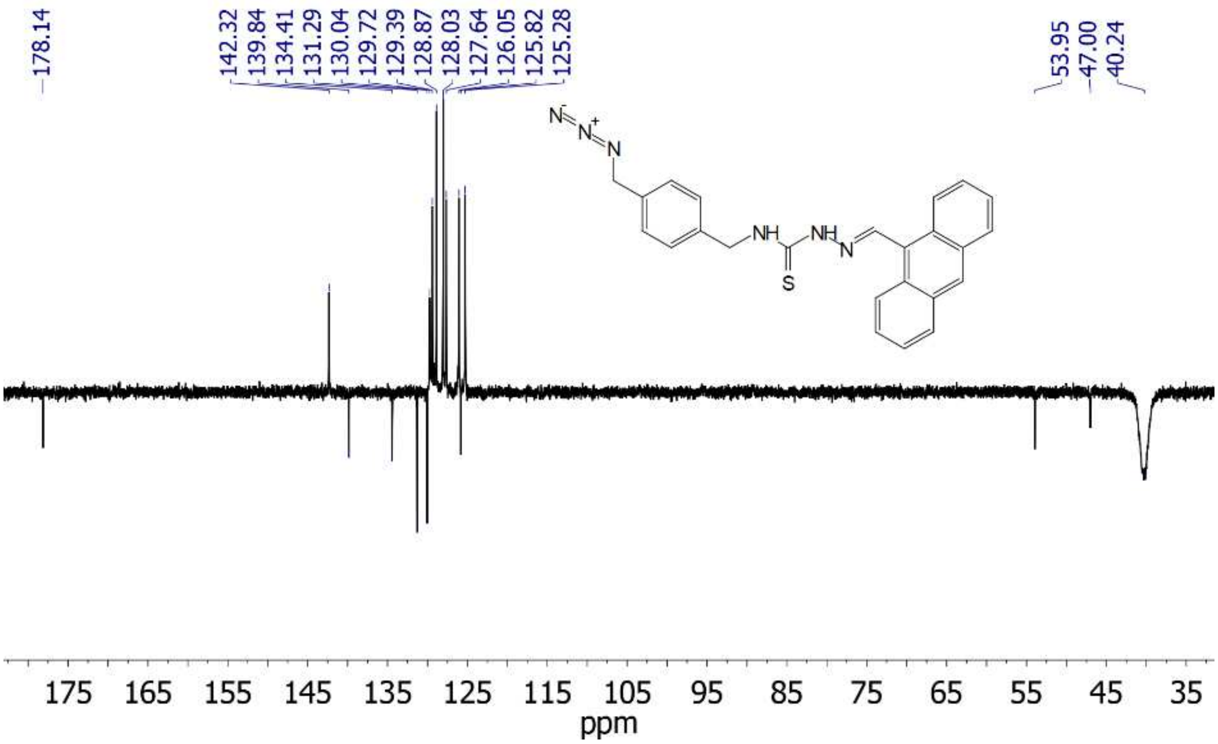

Figure S279. ^{13}C DEPTQ NMR spectrum of [(2*E*)-2-[(anthracen-9-yl)methylidene]hydrazino]-[4-(azidomethyl)phenyl]ethanethioamide in DMSO-d_6 at 300 MHz.

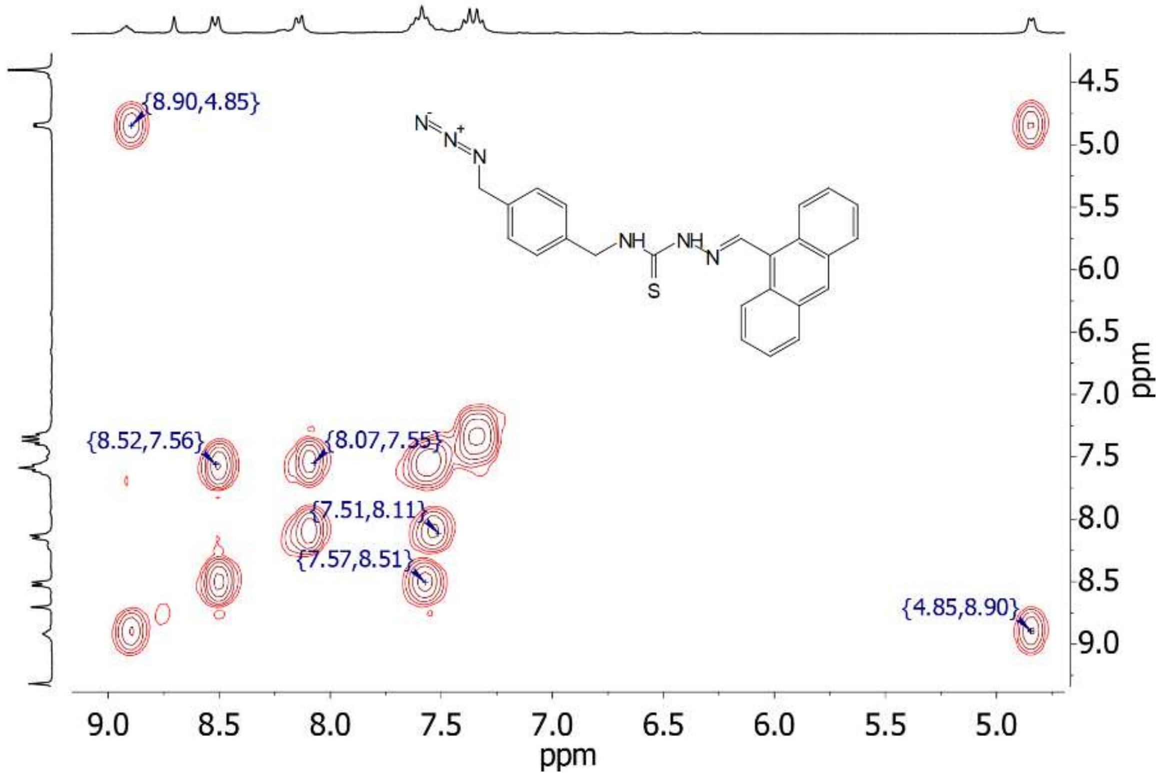

Figure S280. $^{1}H,^{1}H$ COSY NMR spectrum of [(2*E*)-2-[(anthracen-9-yl)methylidene]hydrazino]-[4-(azidomethyl)phenyl]ethanethioamide in DMSO-d_6 at 300 MHz.

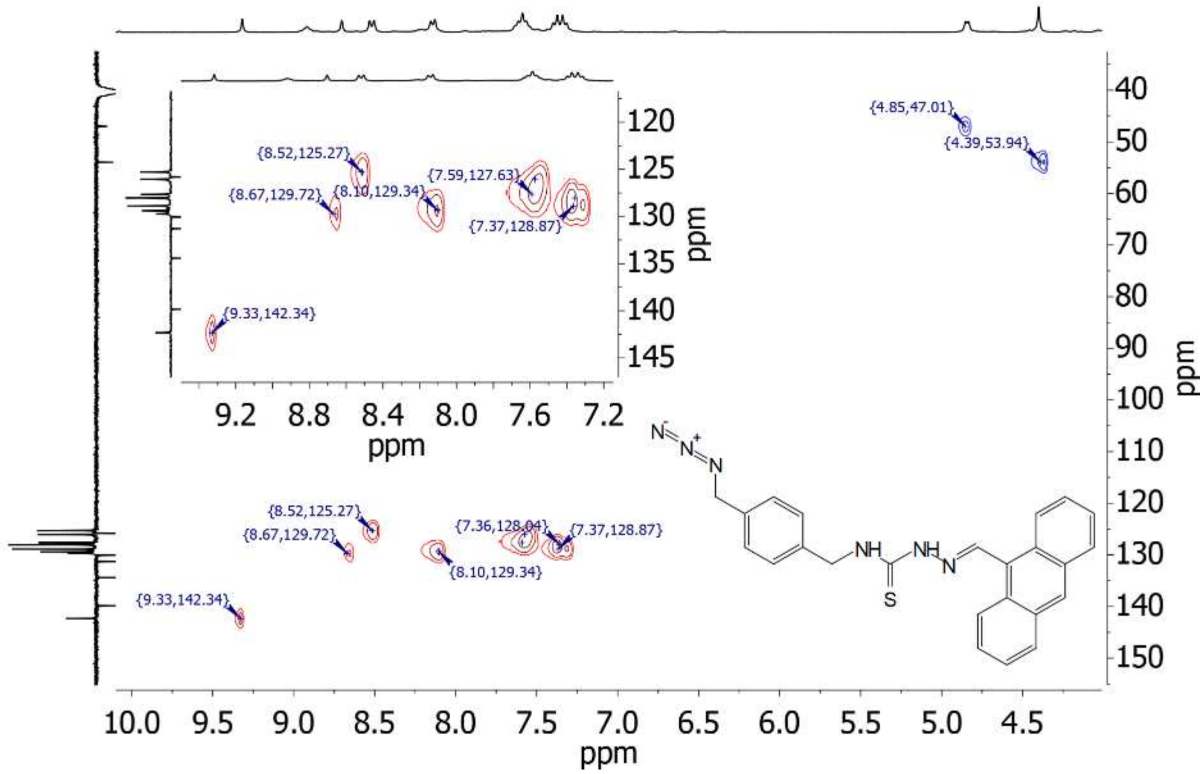

Figure S281. ^{1}H,^{13}C HMQC/HSQC NMR spectrum of [(2*E*)-2-[(anthracen-9-yl)methylidene] hydrazino]-[4-(azidomethyl)phenyl]ethanethioamide in DMSO-d_6 at 300 MHz.

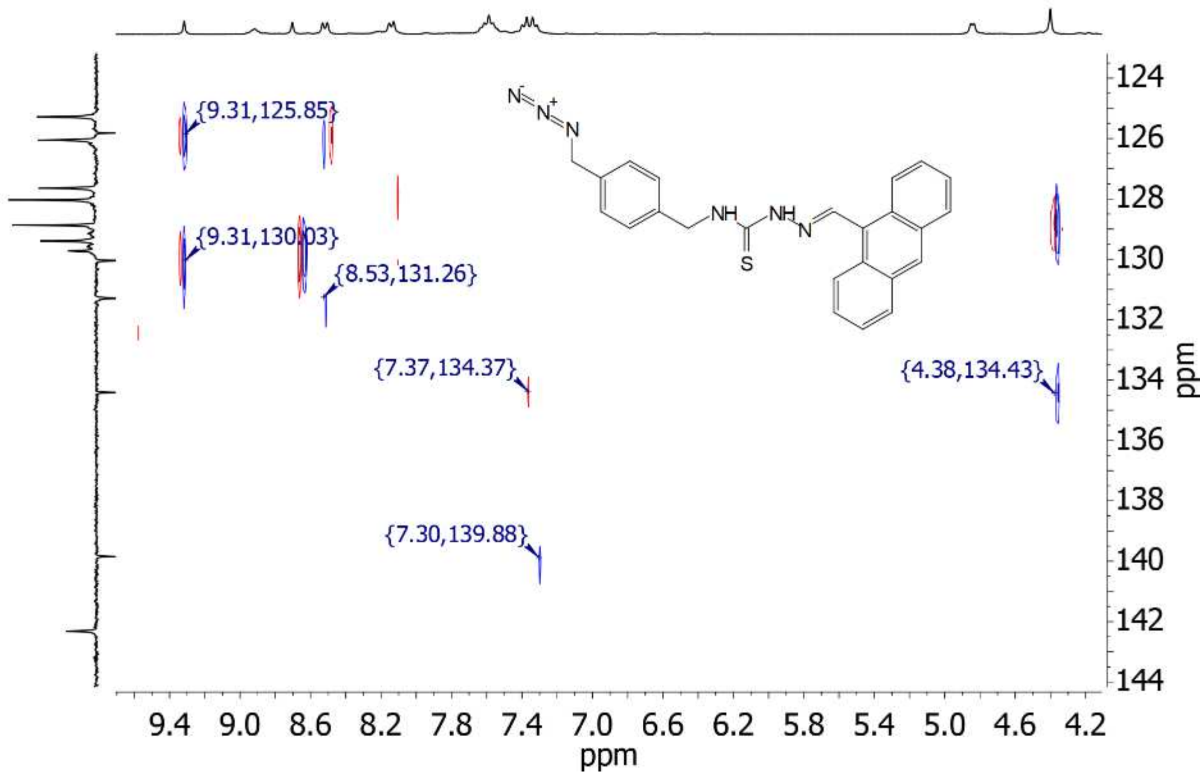

Figure S282. ^{1}H,^{13}C HMBC NMR spectrum of [(2*E*)-2-[(anthracen-9-yl)methylidene]hydrazino]-[4-(azidomethyl)phenyl]ethanethioamide in DMSO-d_6 at 300 MHz.

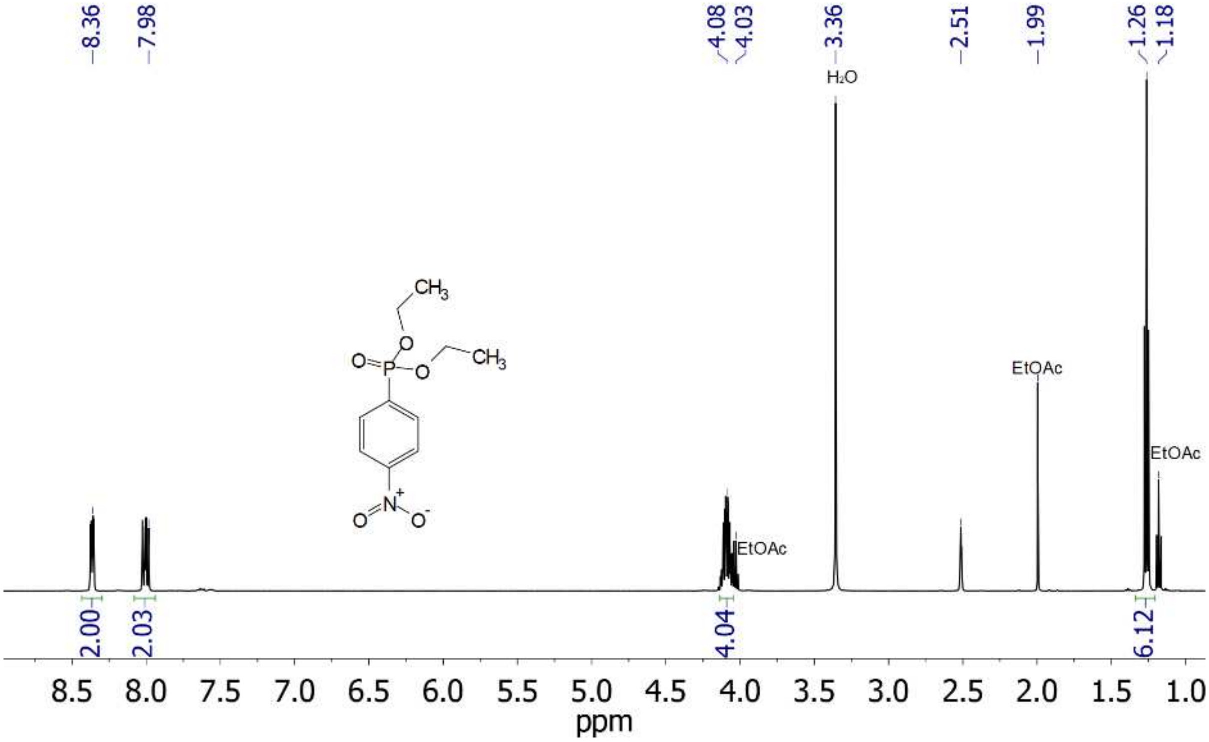

Figure **S283.** ^{1}H NMR spectrum of diethyl(4-nitrophenyl)phosphonate in DMSO-d_6 at 499 MHz.

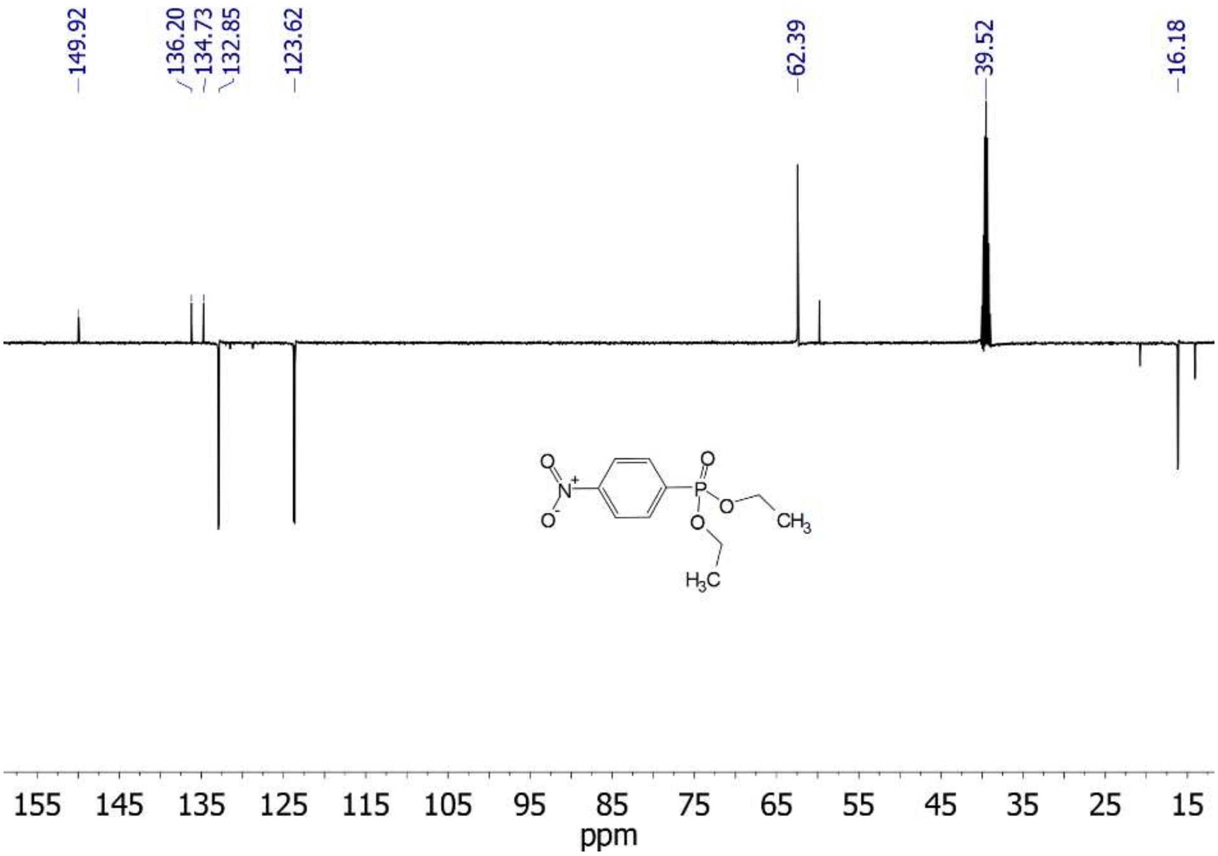

Figure S284. ^{13}C APT NMR spectrum of diethyl(4-nitrophenyl)phosphonate in DMSO-d_6 at 499 MHz.

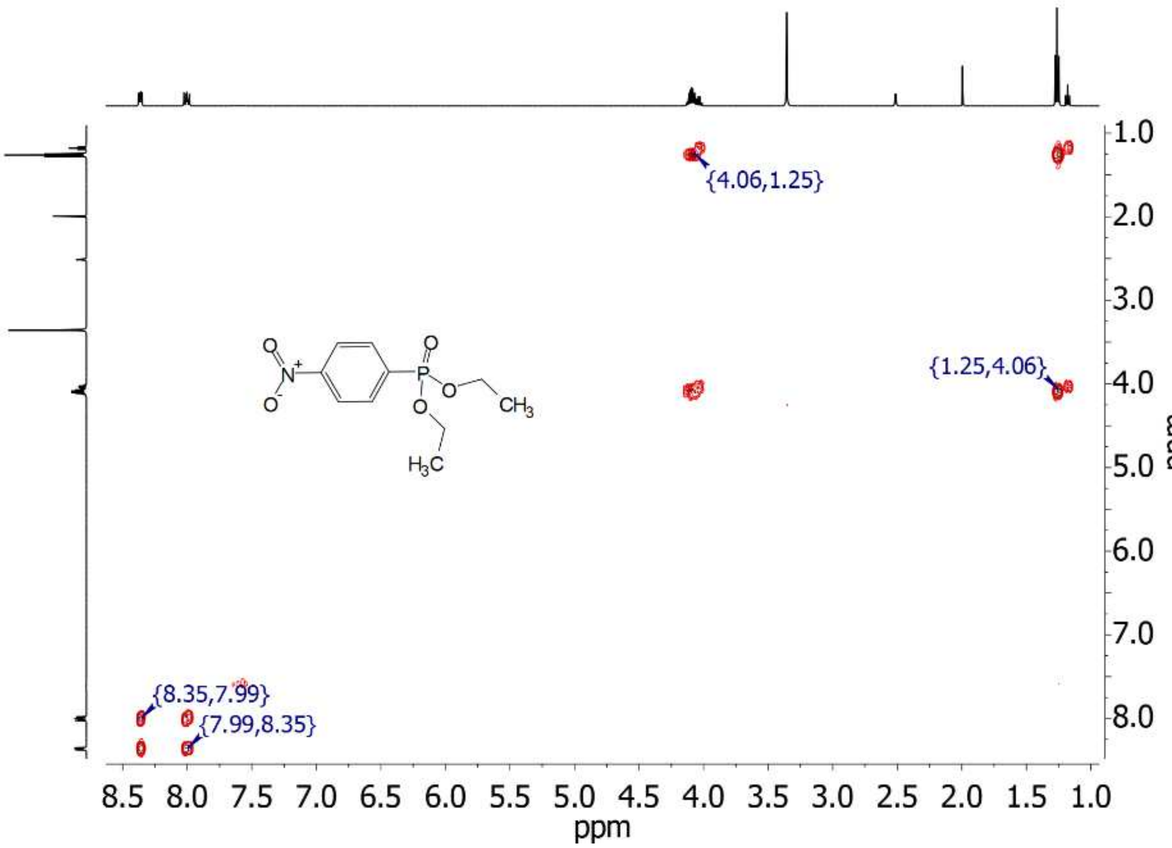

Figure S285. $^{1}H,^{1}H$ COSY NMR spectrum of diethyl(4-nitrophenyl)phosphonate DMSO-d_6 at 499 MHz.

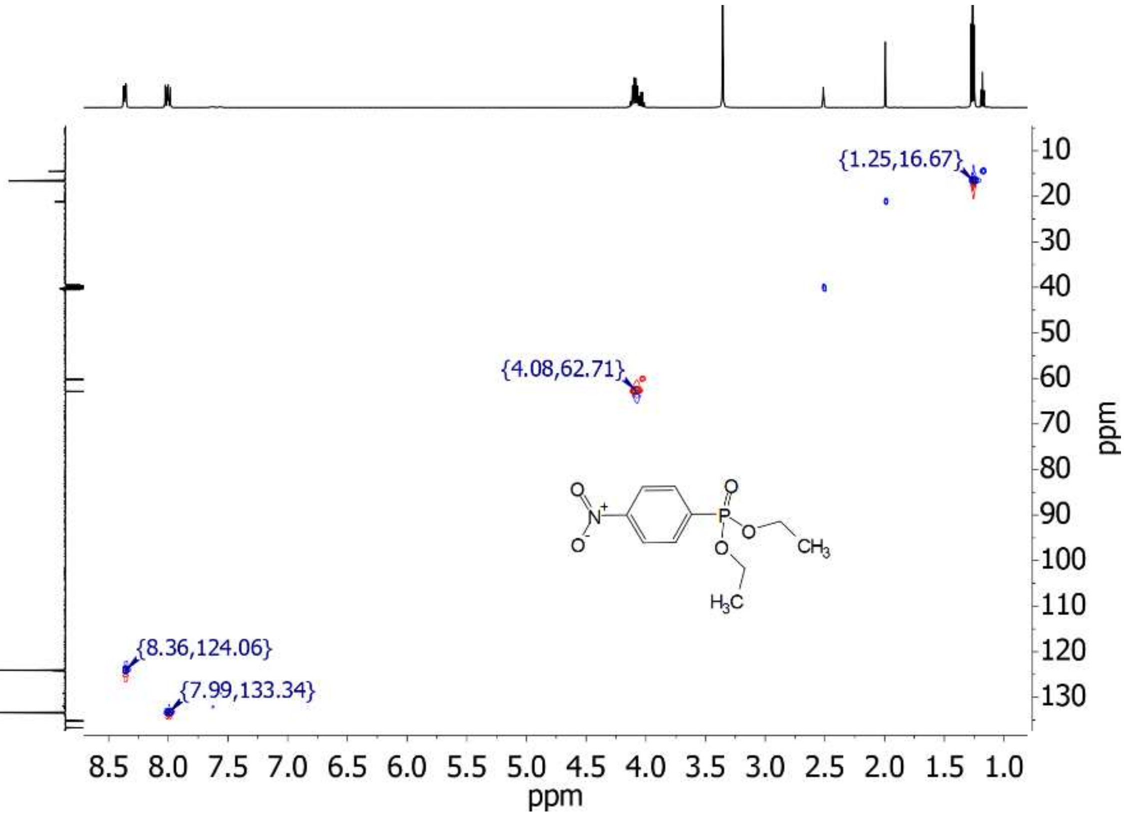

Figure S286. $^{1}H,^{13}C$ HMQC/HSQC NMR spectrum of diethyl(4-nitrophenyl)phosphonate in DMSO-d_6 at 499 MHz.

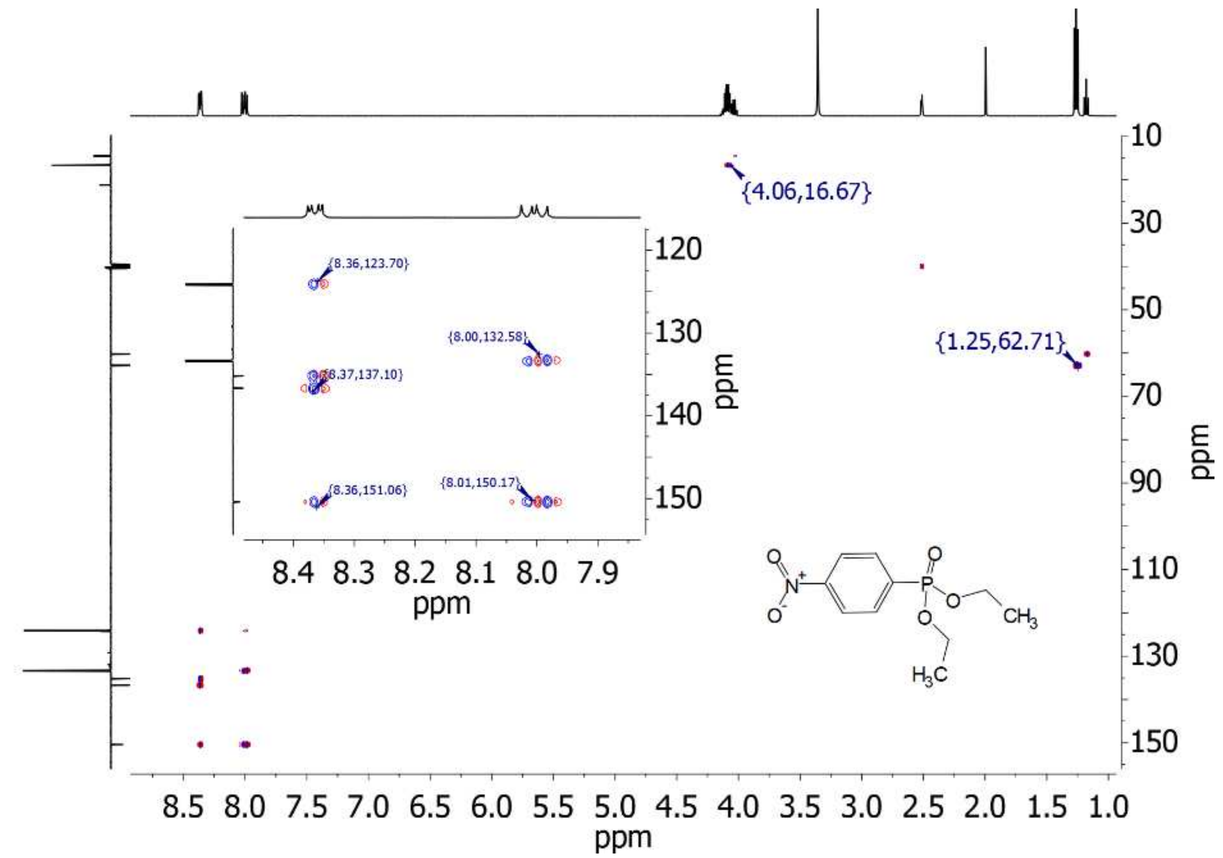

Figure S287. ^{1}H,^{13}C HMBC NMR spectrum of diethyl(4-nitrophenyl)phosphonate in DMSO-d_6 at 300 MHz.

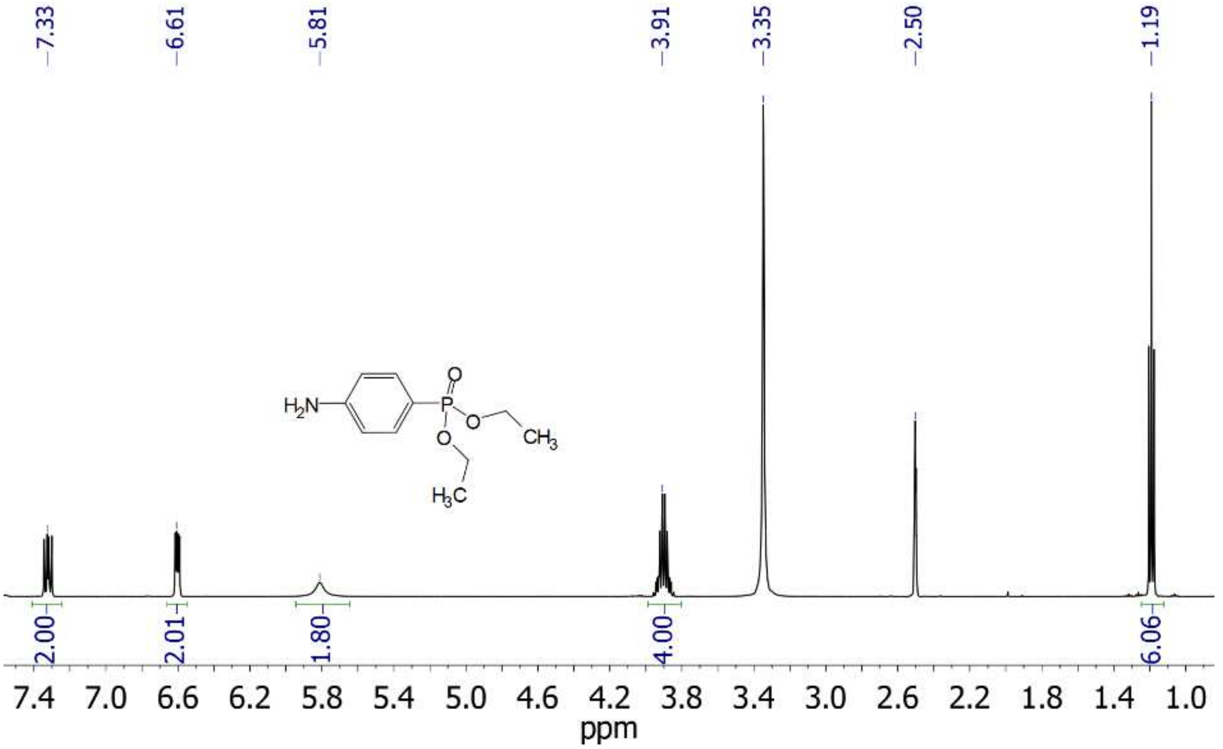

Figure S288. ^{1}H NMR spectrum of diethyl(4-aminophenyl)phosphonate in DMSO-d_6 at 499 MHz.

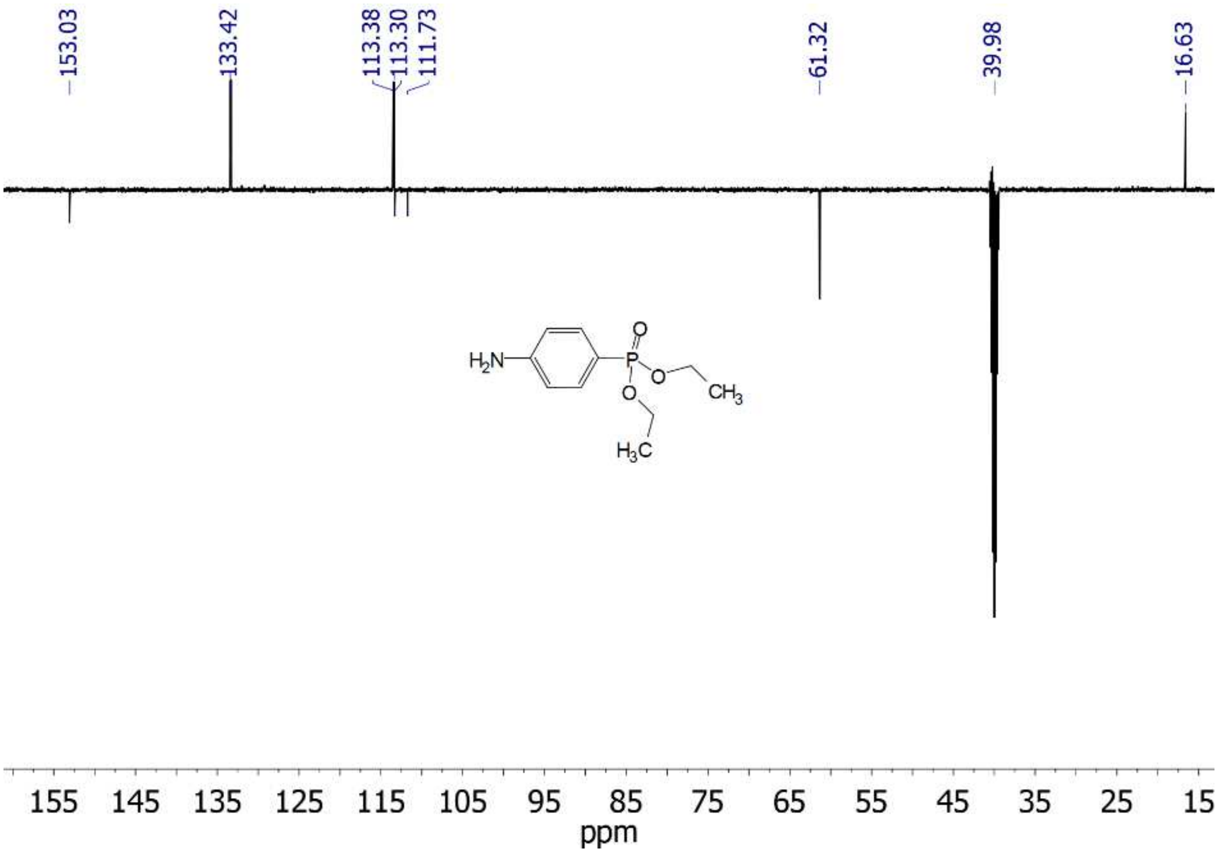

Figure S289. ^{13}C APT NMR spectrum of diethyl(4-aminophenyl)phosphonate in DMSO-d_6 at 499 MHz.

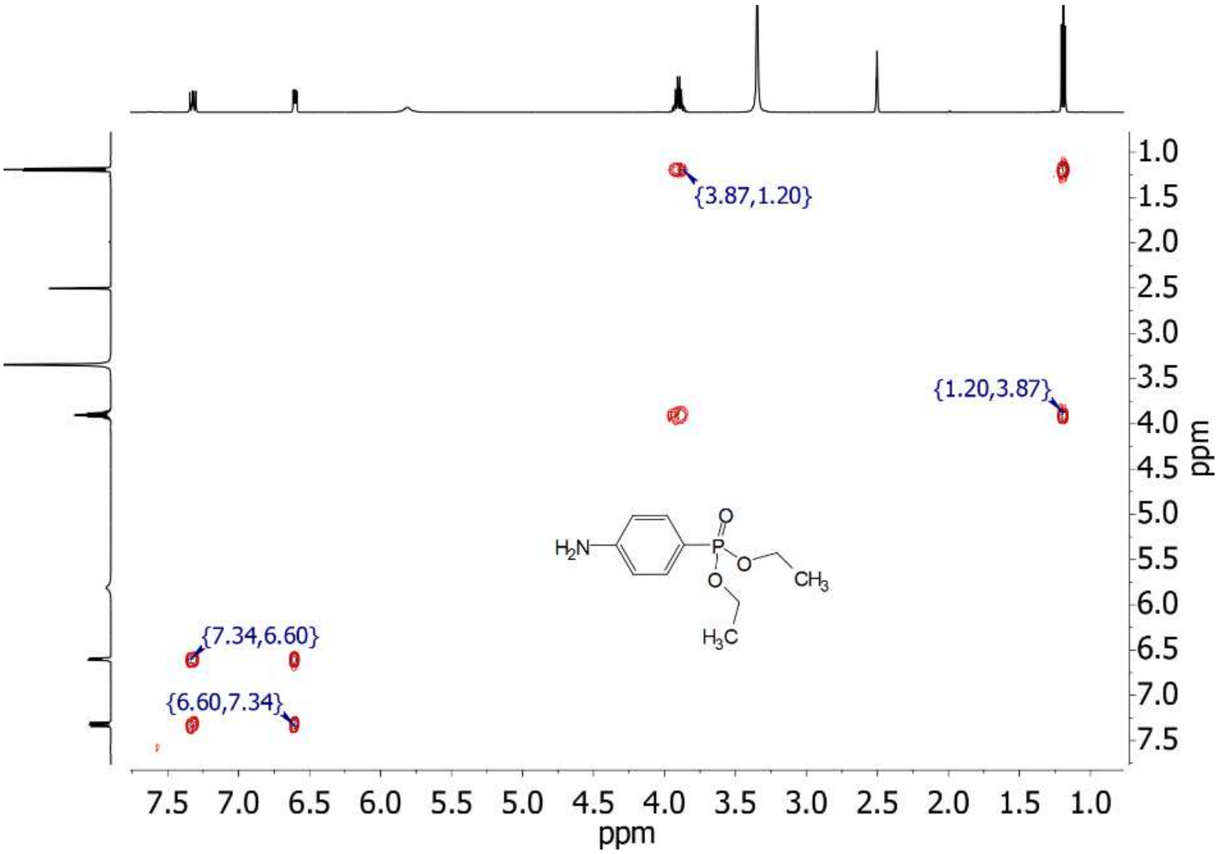

Figure S290. ^{1}H,^{1}H COSY NMR spectrum of diethyl(4-aminophenyl)phosphonate in DMSO-d_6 at 499 MHz.

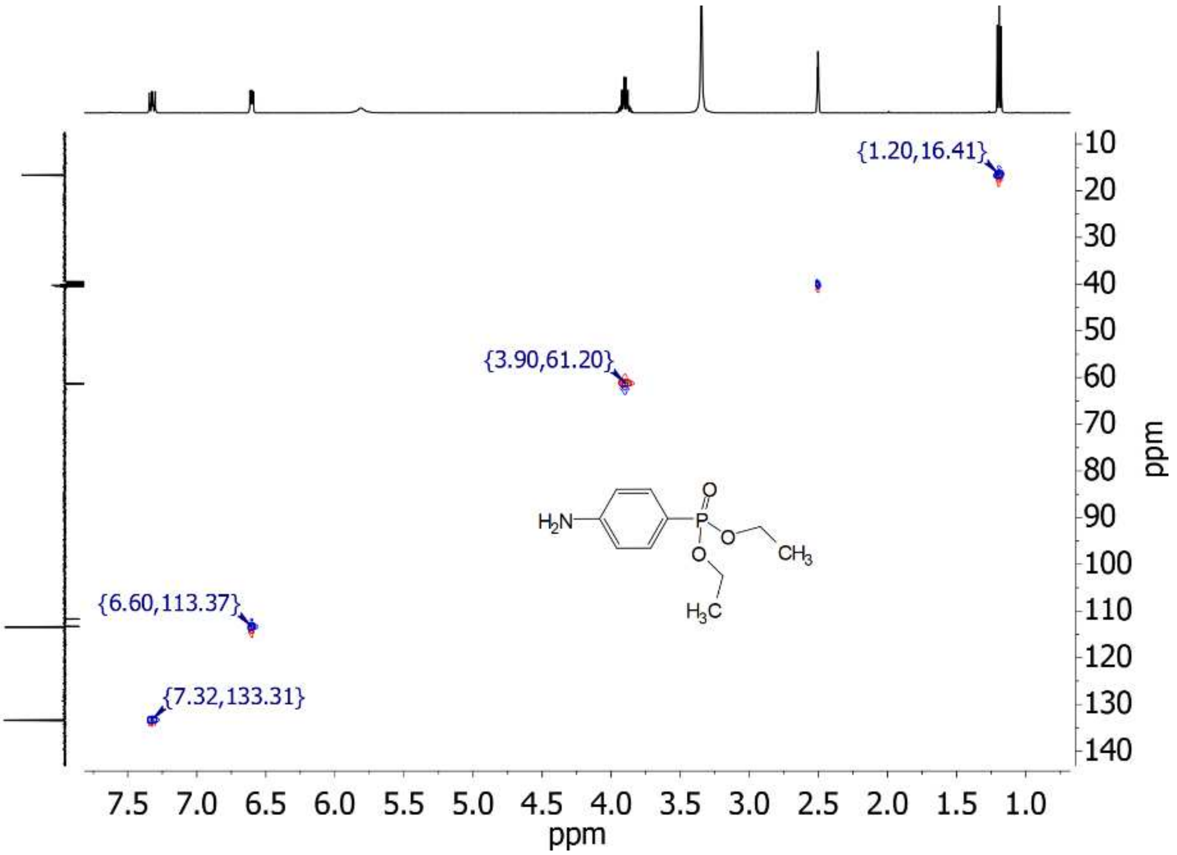

Figure S291. $^{1}H,^{13}C$ HMQC/HSQC NMR spectrum of diethyl(4-aminophenyl)phosphonate in DMSO-d_6 at 499 MHz.

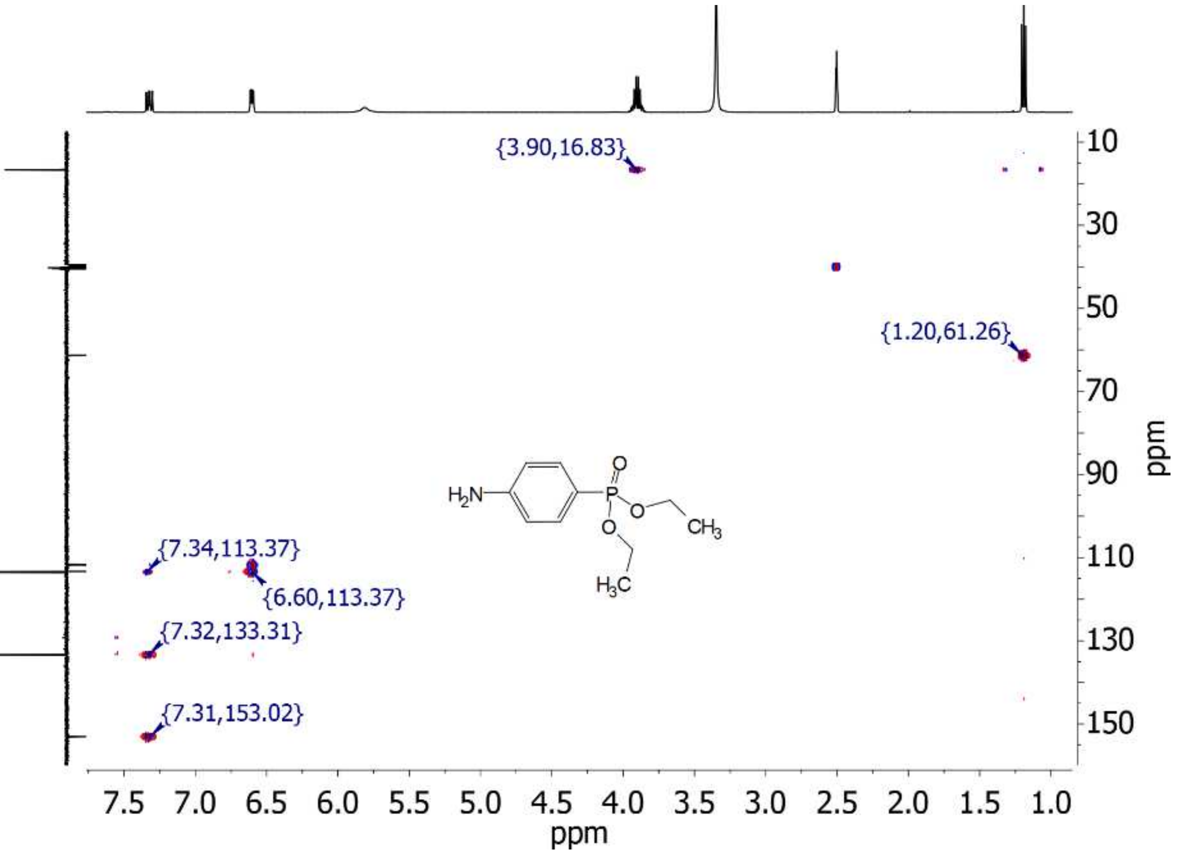

Figure S292. $^{1}H,^{13}C$ HMBC NMR spectrum of diethyl(4-aminophenyl)phosphonate in DMSO-d_6 at 499 MHz.

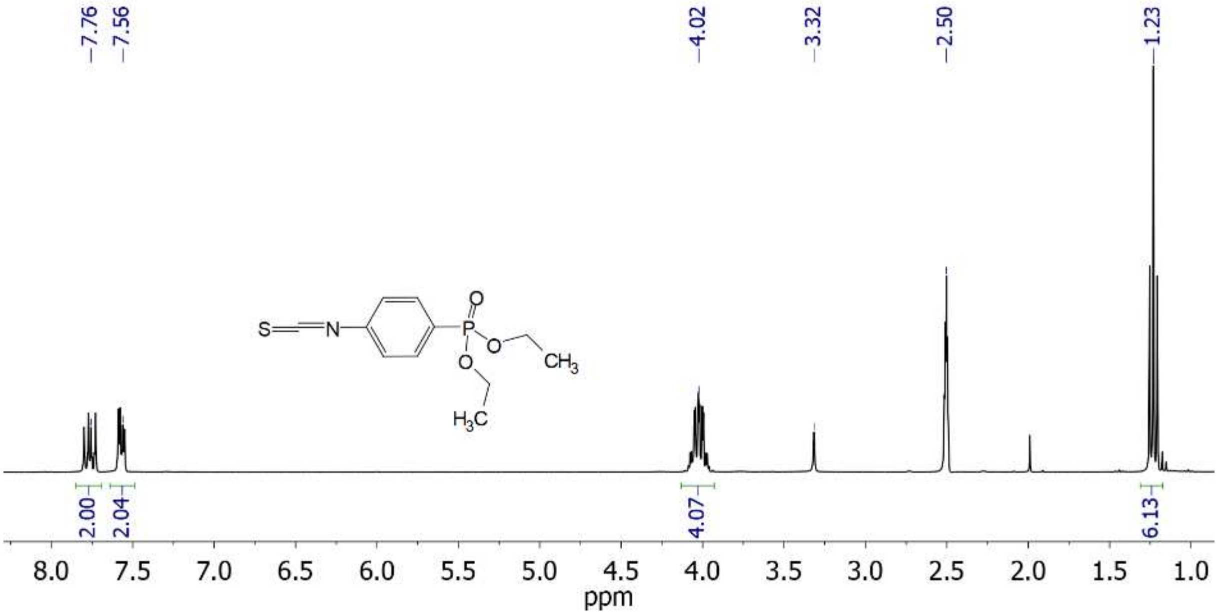

Figure S293. ^{1}H NMR spectrum of diethyl (4-isothiocyanatophenyl)phosphonate in DMSO-d_6 at 300 MHz.

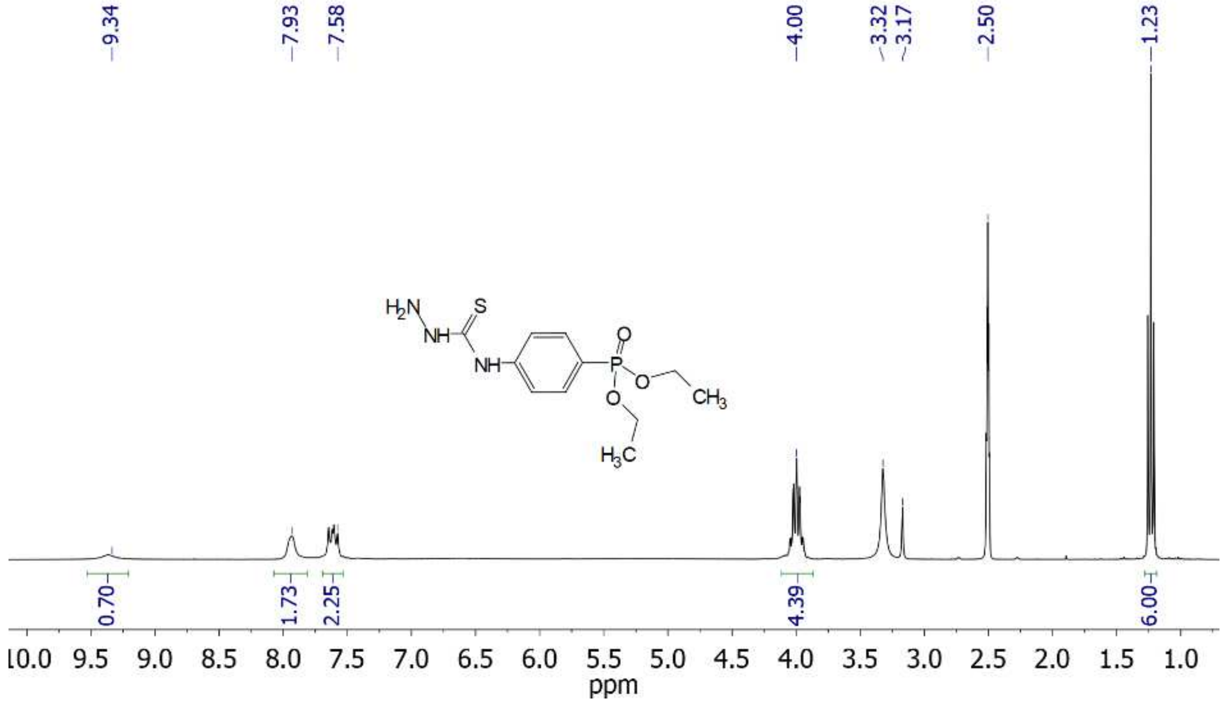

Figure S294. ^{1}H NMR spectrum of diethyl [4-(hydrazidecarbothioamino)phenyl] phosphonate in DMSO-d_6 at 300 MHz.

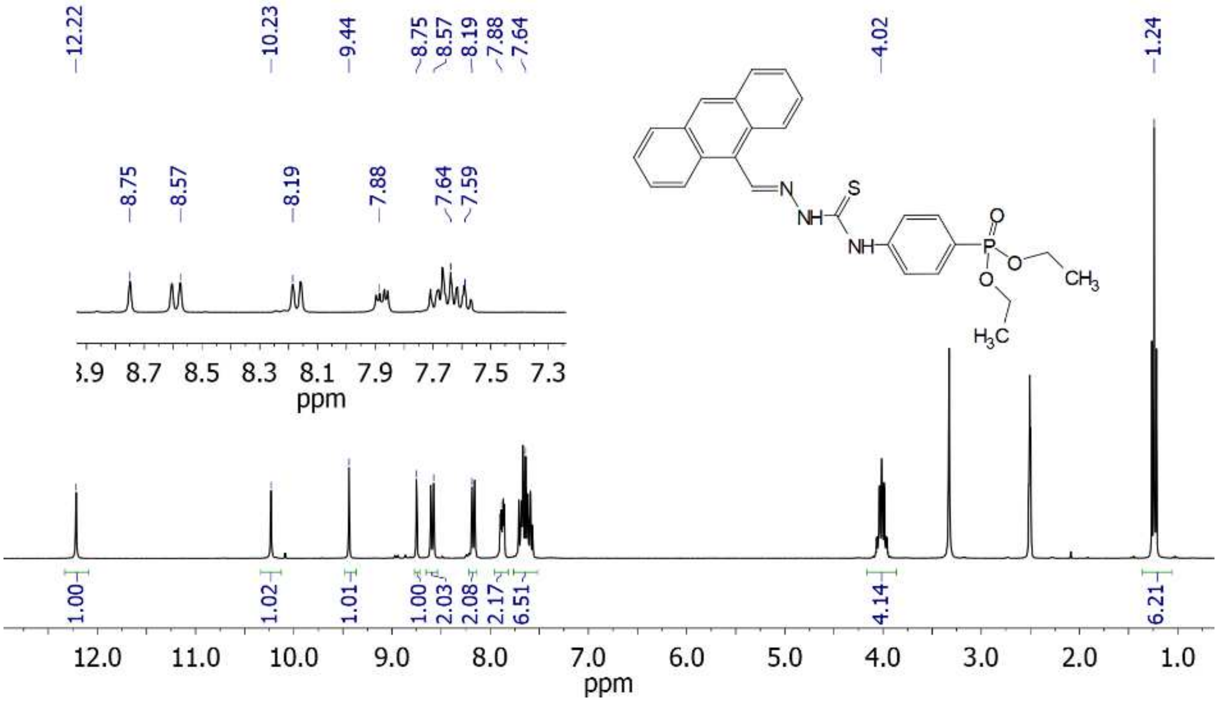

Figure S295. ^{1}H NMR spectrum of [(2*E*)-2-[(anthracen-9-yl)methylidene]hydrazino]-4-(diethoxy-phosphoryl)benzene-1-carbothioamide in DMSO-d_6 at 499 MHz.

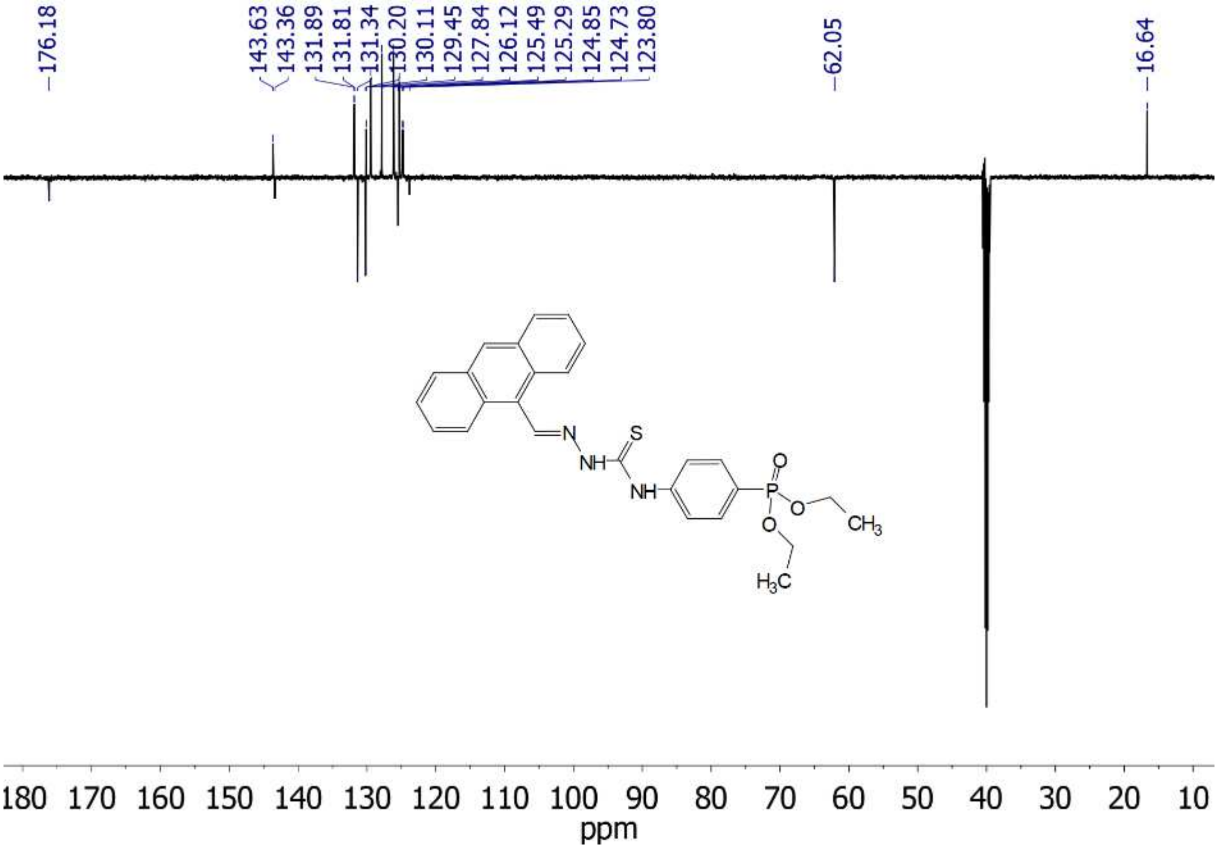

Figure S296. ^{13}C APT NMR spectrum of [(2*E*)-2-[(anthracen-9-yl)methylidene]hydrazino]-4-(diethoxyphosphoryl)benzene-1-carbothioamide in DMSO-d_6 at 499 MHz.

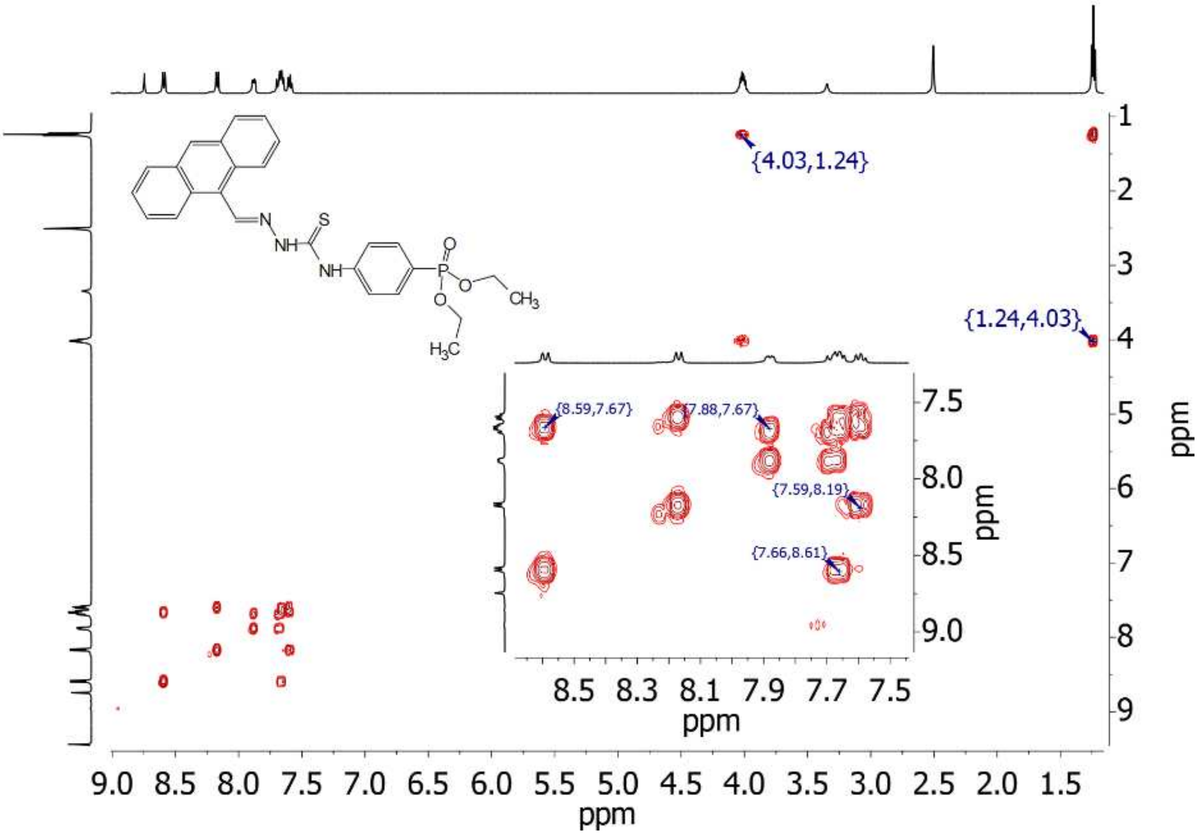

Figure S297. ^{1}H,^{1}H COSY NMR spectrum of [(2*E*)-2-[(anthracen-9-yl)methylidene]hydrazino]-4-(diethoxyphosphoryl)benzene-1-carbothioamide in DMSO-d_6 at 499 MHz.

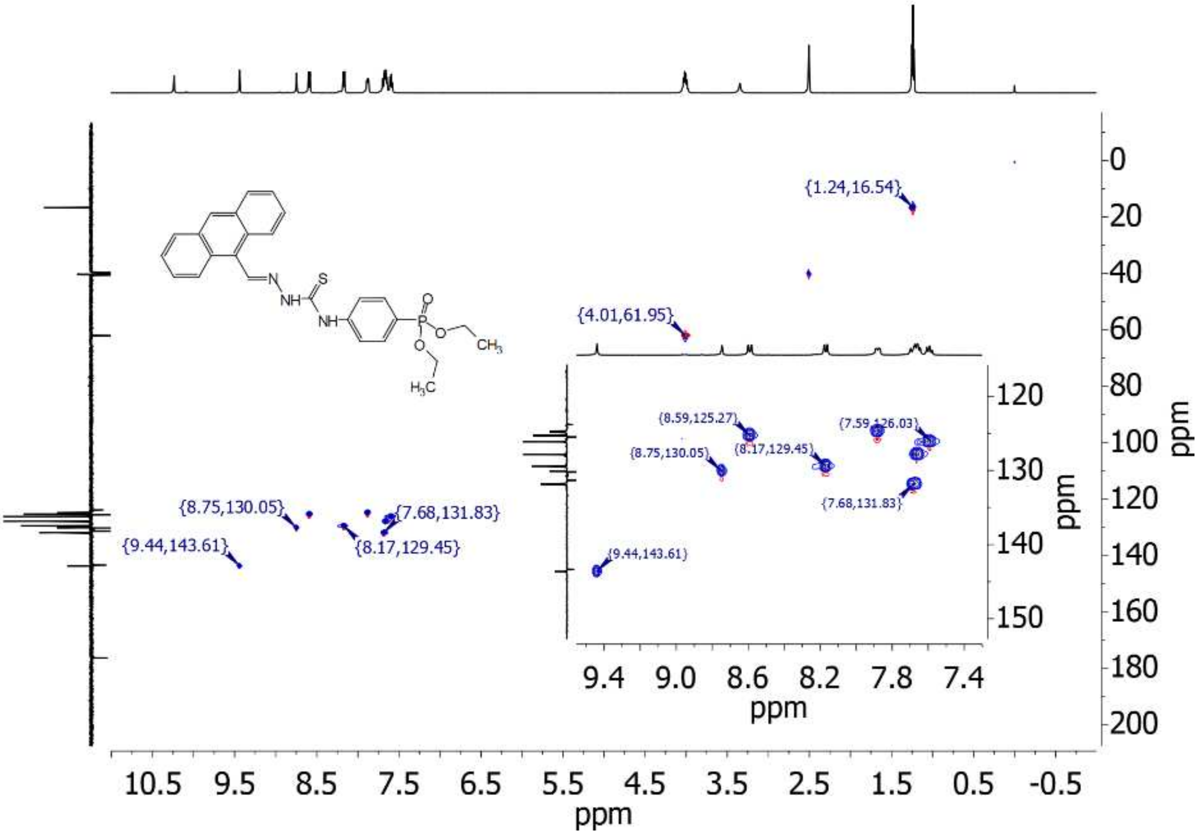

Figure S298. ^{1}H,^{13}C HMQC/HSQC NMR spectrum of [(2*E*)-2-[(anthracen-9-yl)methylidene] hydrazino]-4-(diethoxyphosphoryl)benzene-1-carbothioamide in DMSO-d_6 at 499 MHz.

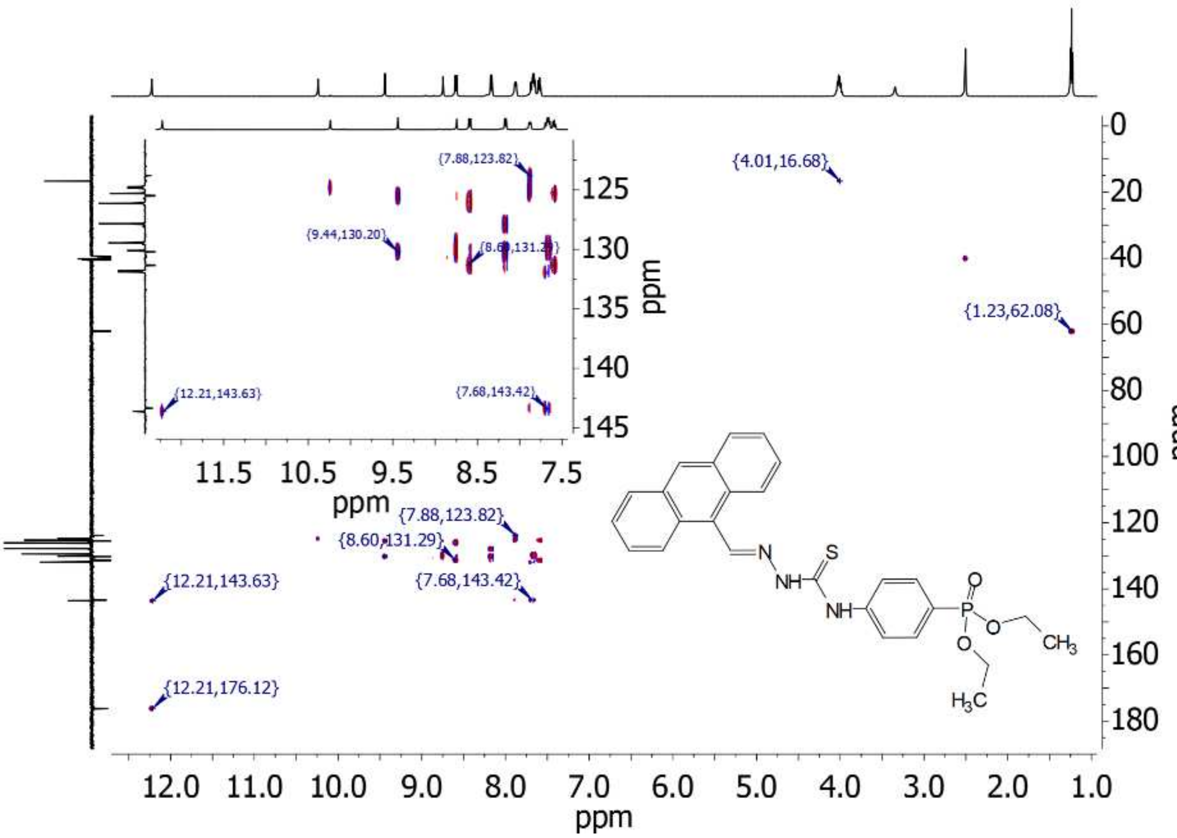

Figure S299. ^{1}H,^{13}C HMBC NMR spectrum of [(2*E*)-2-[(anthracen-9-yl)methylidene]hydrazino]-4-(diethoxyphosphoryl)benzene-1-carbothioamide in DMSO-*d*$_6$ at 499 MHz.

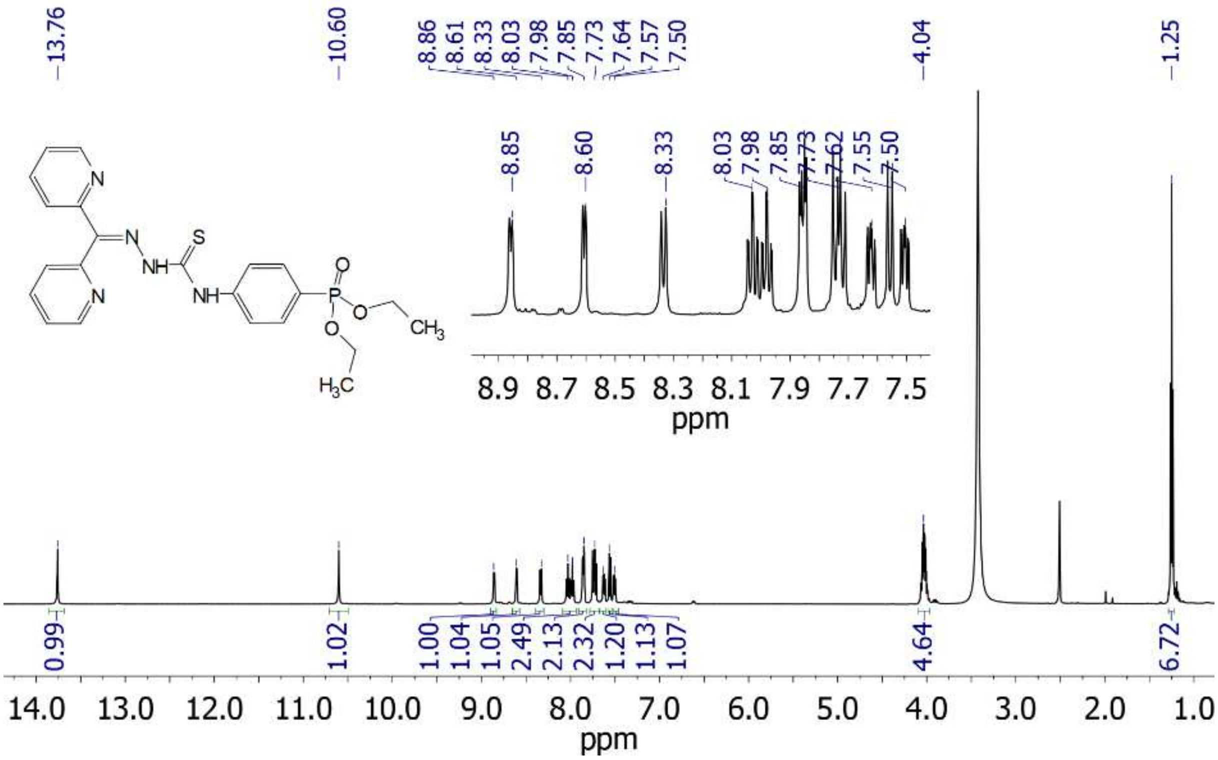

Figure S300. ^{1}H NMR spectrum of {2-[bis(pyridin-2-yl)methylidene]hydrazino}-4-(diethoxy phosphoryl)benzene-1-carbothioamide in DMSO-*d*$_6$ at 499 MHz.

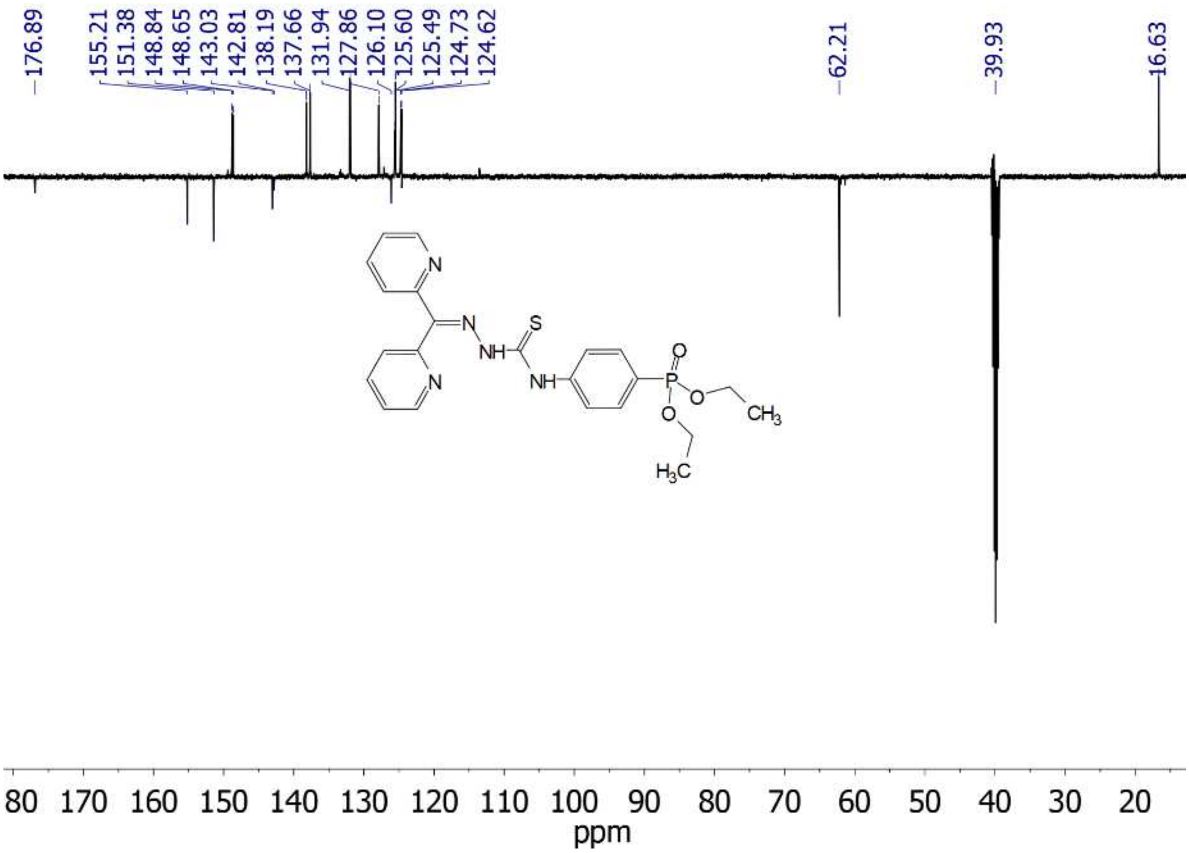

Figure S301. ^{13}C APT NMR spectrum of {2-[bis(pyridin-2-yl)methylidene]hydrazino}-4-(diethoxy-phosphoryl)benzene-1-carbothioamide in DMSO-d_6 at 499 MHz.

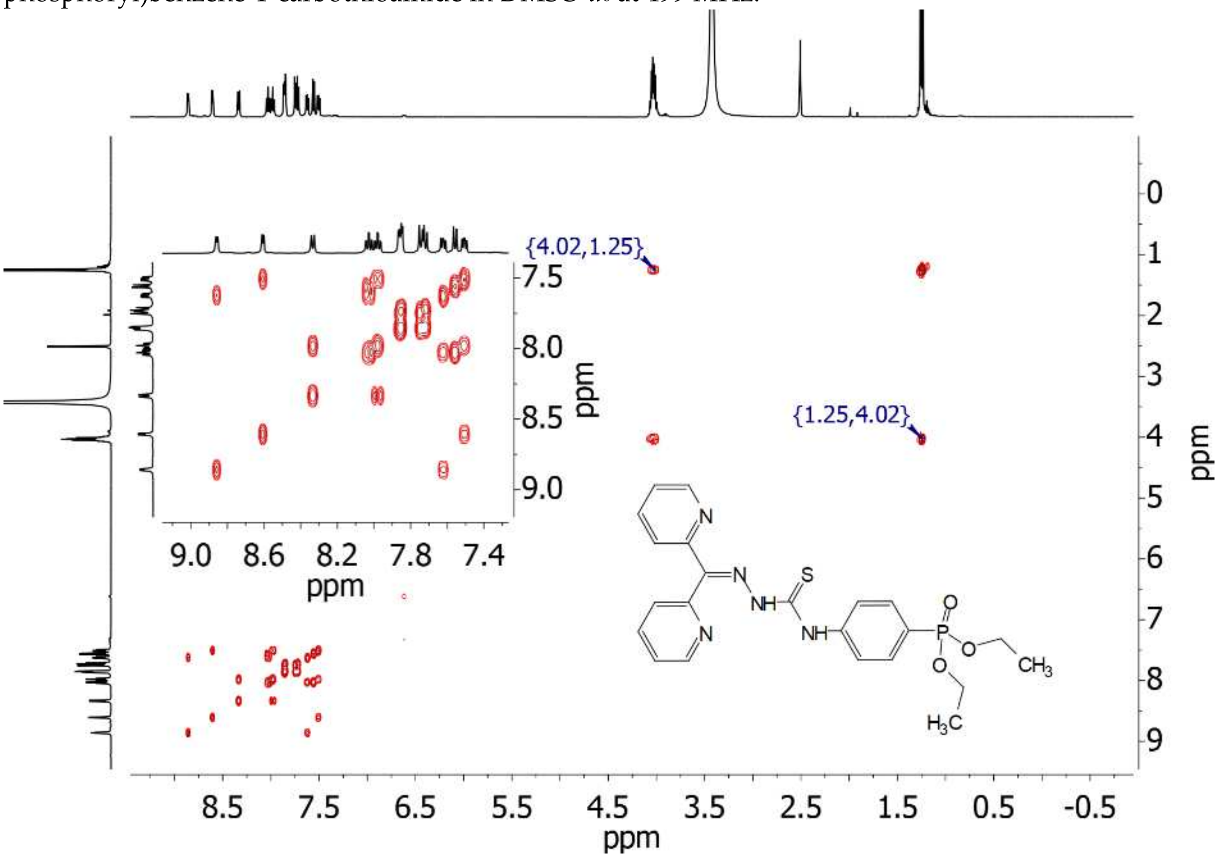

Figure S302. ^{1}H,^{1}H COSY NMR spectrum of {2-[bis(pyridin-2-yl)methylidene]hydrazino}-4-(diethoxy-phosphoryl)benzene-1-carbothioamide in DMSO-d_6 at 499 MHz.

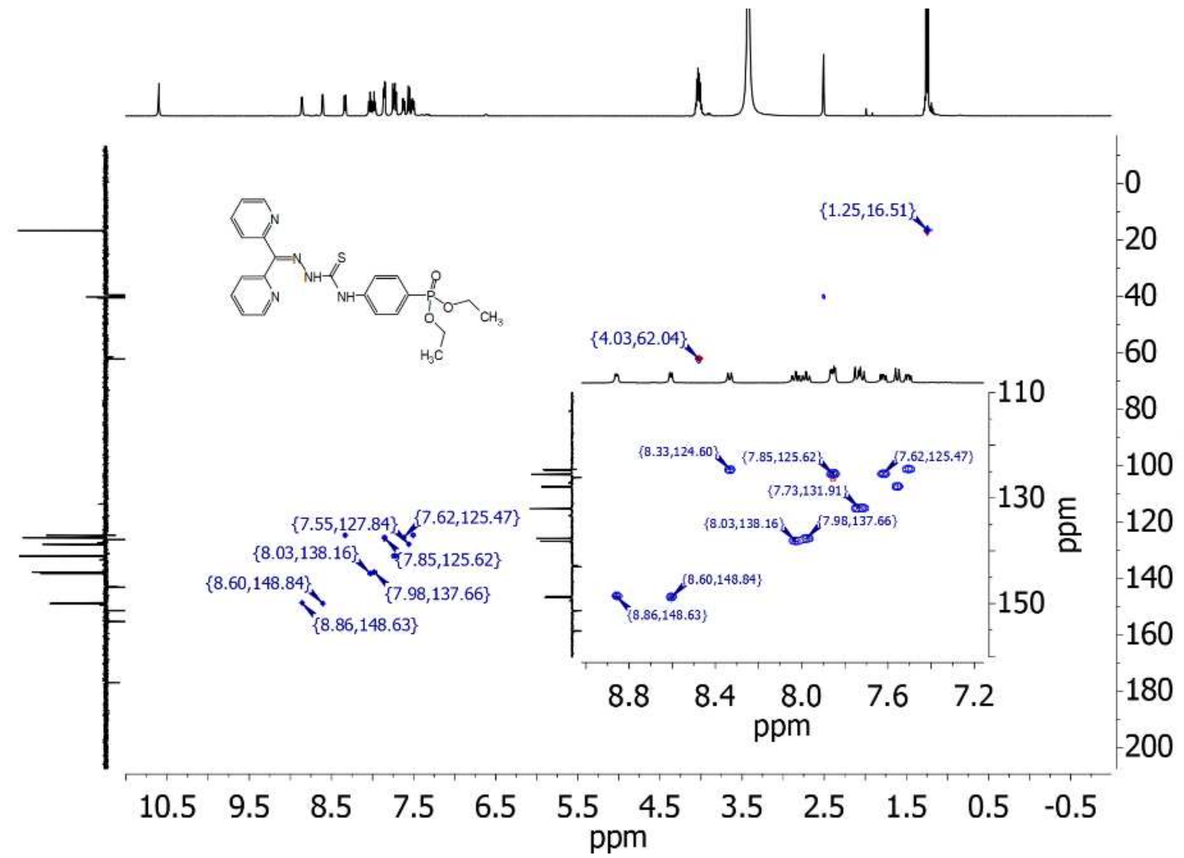

Figure S303. $^{1}H,^{13}C$ HMQC/HSQC NMR spectrum of {2-[bis(pyridin-2-yl)methylidene]hydrazino}-4-(diethoxyphosphoryl)benzene-1-carbothioamide in DMSO-d_6 at 499 MHz.

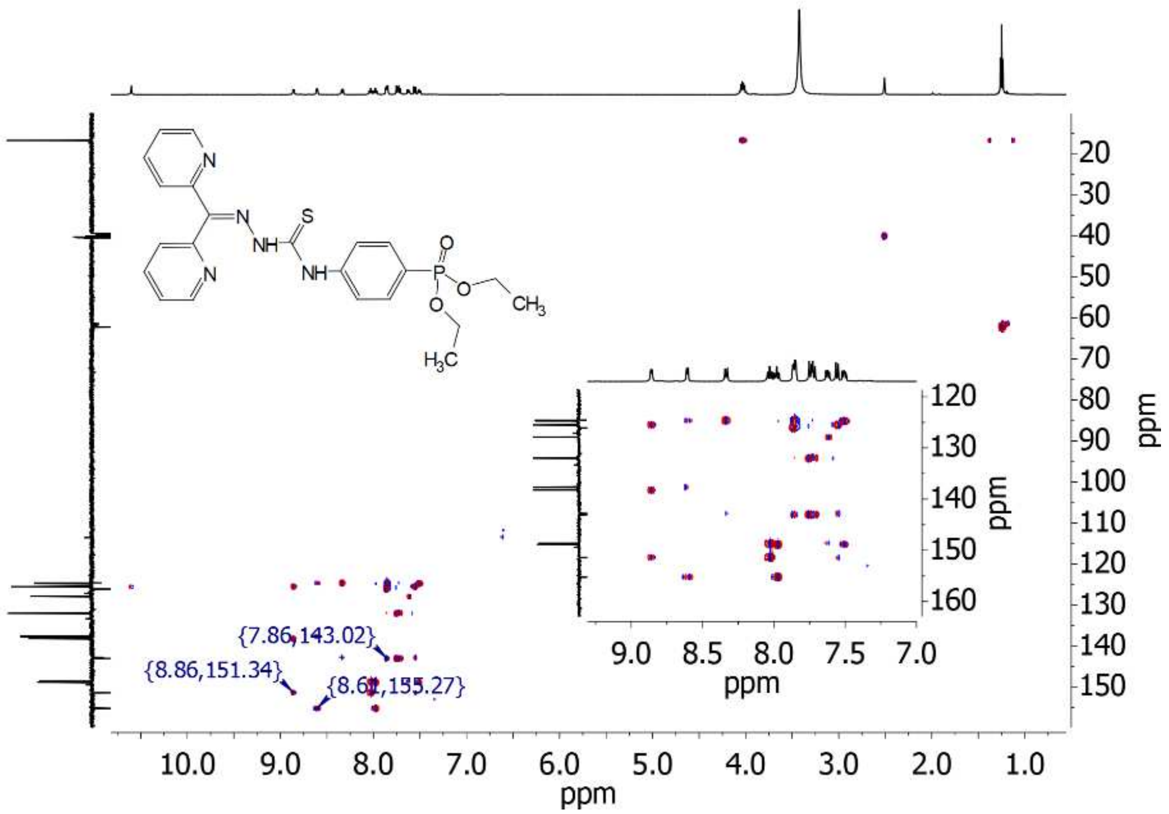

Figure S304. $^{1}H,^{13}C$ HMBC NMR spectrum of {2-[bis(pyridin-2-yl)methylidene]hydrazino}-4-(diethoxyphosphoryl)benzene-1-carbothioamide in DMSO-d_6 at 499 MHz.

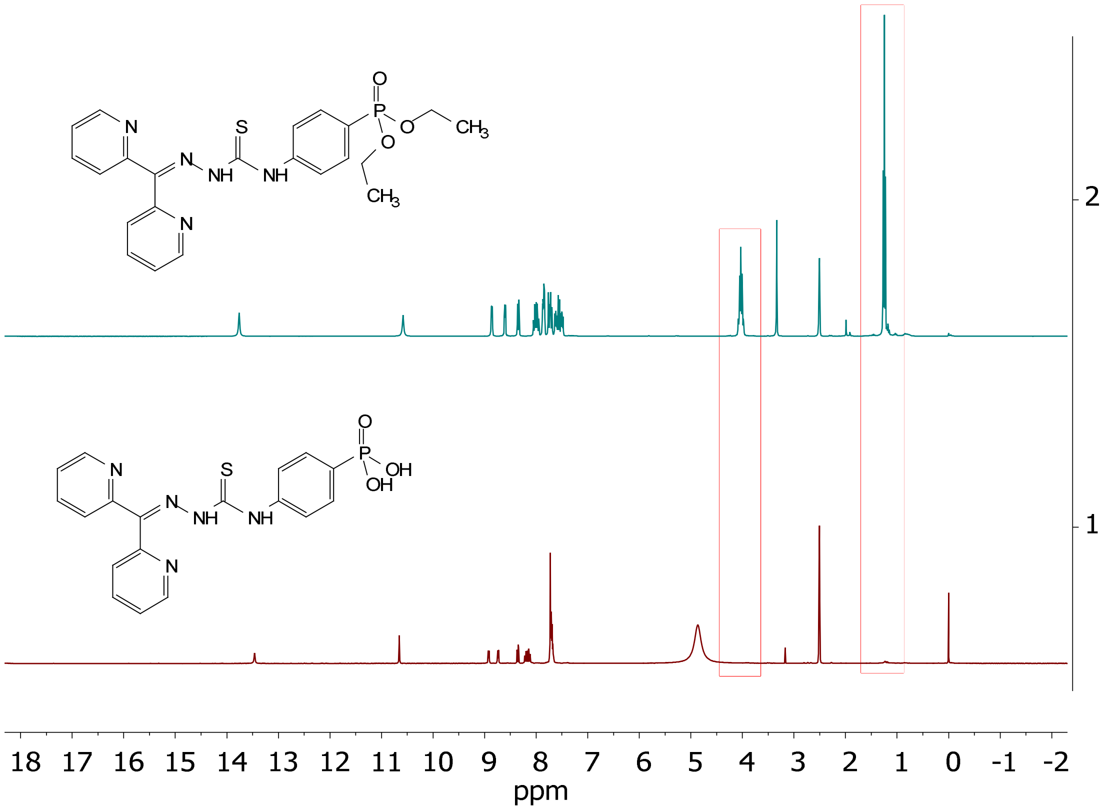

Figure S305. ^{1}H NMR spectra of the dipyridyl-TSC-phosphonate ethyl ester and the deprotected TSC-phosphonic acid in DMSO-d_6 at 499 MHz. The red boxes mark the ethyl signals of the ester.

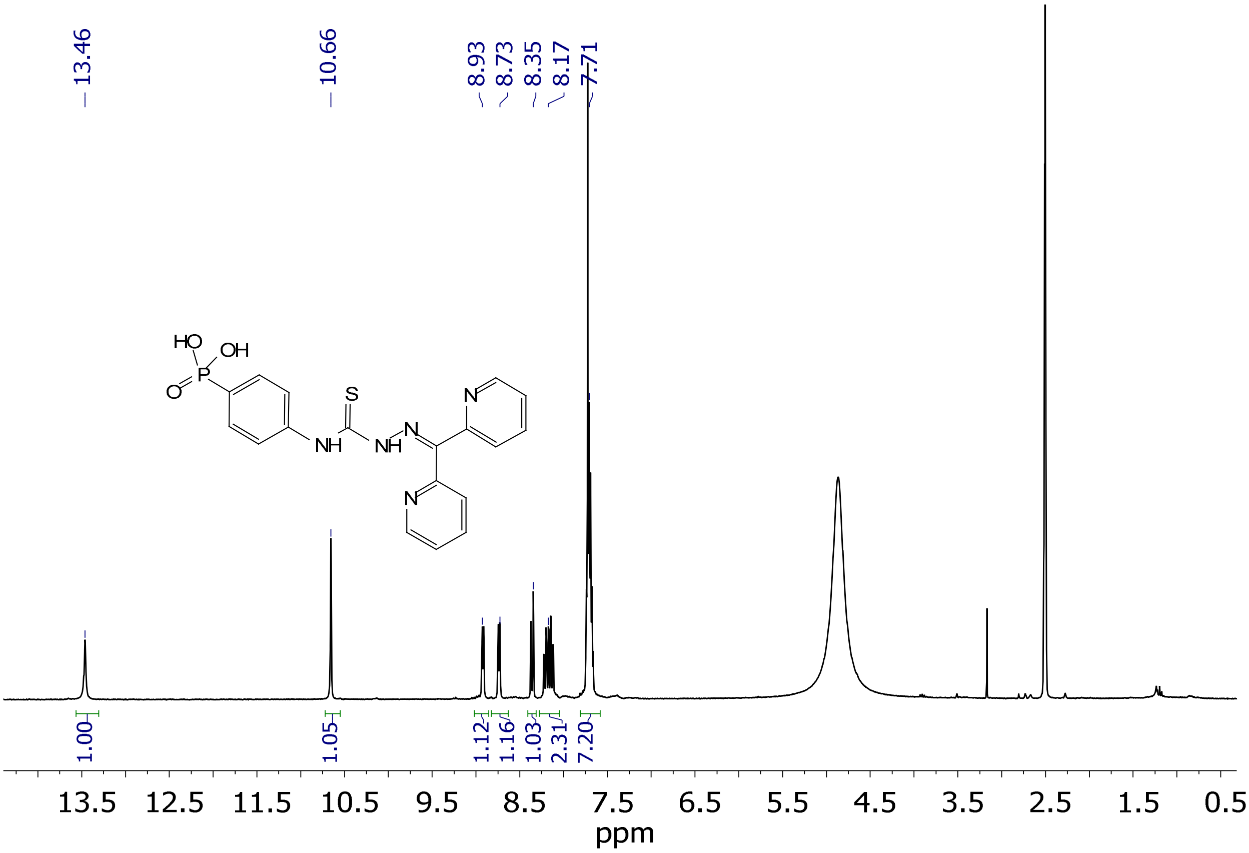

Figure S306. ^{1}H NMR spectrum of (4-{[bis(pyridin-2yl)methylideneaminocarbamthioyl]amino}phenyl) phosphonic acid in DMSO-d_6 at 300 MHz.

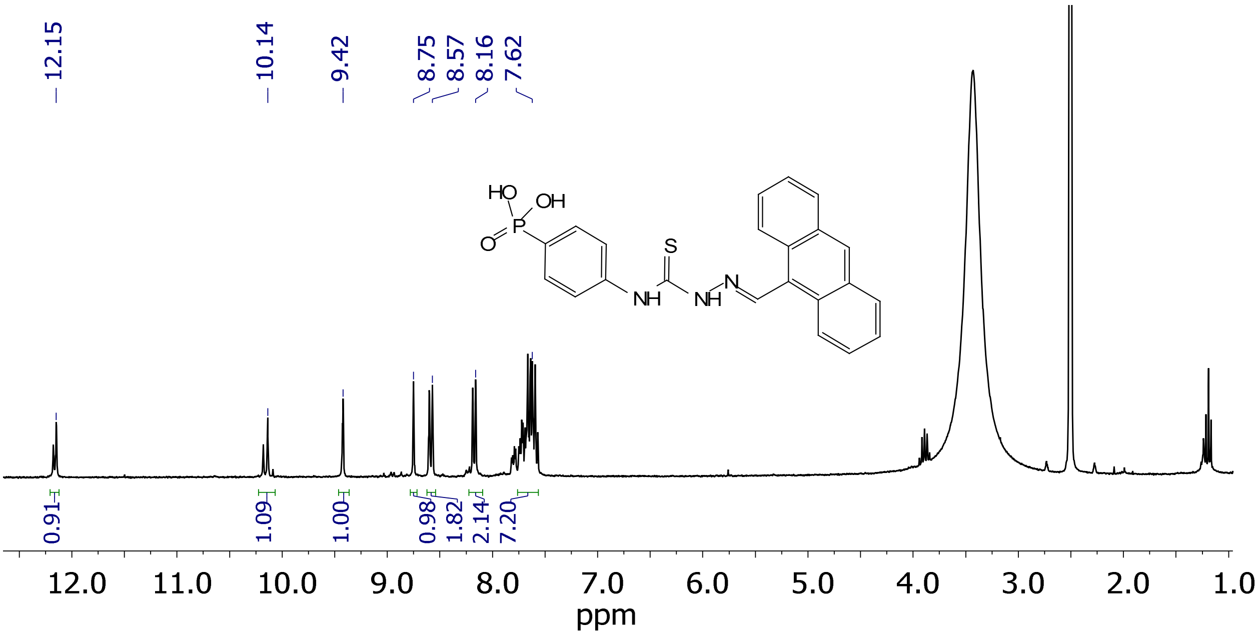

Figure S307. ^{1}H NMR spectrum of [(2Z)-2-[(anthracen-9-yl)methylidene]hydrazino]-4-phosphono benzene-1-carbothioamide in DMSO-d_6 at 300 MHz.

2. Supplementary Tables

Table S1. Specific optical rotation values for the [dipy-TSC-X] conjugates.[a]

			[TSC-amino acid-NHBoc]					[TSC-glucose]
	Phenyl-alanine	Alanine	Leucin	Iso-leucine	Valin	Aspartic acid	Tyrosine	
Conc. [g/100cm³]	0.1160	0.1170	0.1000	0.1045	0.1040	0.1150	0.1045	0.1165
$[\alpha]_{546}^{20}$	+35.6	–	+5.0	+23.9	+29.3	–9.7	+20.1	+95.3
$[\alpha]_{579}^{20}$	+30.5	+15.1	+5.0	+22.6	+29.5	–7.5	+18.5	+77.0
$[\alpha]_{589}^{20}$	+29.3	+13.4	+4.7	+22.0	+25.3	–6.4	+18.8	+74.7

[a] Measured at 20 °C in $CHCl_3$ at λ = 546, 579 or 589 nm. Accuracy: ±0.1.
