# Supplementary material for: Functionalizing Thiosemicarbazones for Covalent Conjugation
Source: Molecules. 2024 Aug 3;29(15):3680. doi: 10.3390/molecules29153680 (PMC11314635; doi:10.3390/molecules29153680)
Supplement: Supplementary file 1 [file molecules-29-03680-s001.zip › molecules-3059644-supplementary Part II.pdf]

Supplementary Materials – Part II

Functionalizing Thiosemicarbazones for Covalent Conjugation

Johannes Hohnsen, Lukas Rryci, Diana Obretenova, Joshua Friedel, Shahab Jouchaghani, and Axel Klein*

University of Cologne, Faculty of Mathematics and Natural Sciences, Department of Chemistry and Biochemistry, Institute for Inorganic and Materials Chemistry, Greinstraße 6, 50939 Koeln, Germany.
* Correspondence: axel.klein@uni-koeln.de; Tel.: +49-221-470-4006; ORCID: 0000-0003-0093-9619

Contents

Figure S308. Photographs of TiO_2 (Aeroxid P25®) and the TSC-phosphonic acid-TiO_2 conjugates.
Figure S309. FT-IR spectra of pristine TiO_2 NPs, the [TSC–Ph–phos–OH] conjugates and the [TSC–Ph–phos–TiO_2] conjugates.
Figure S310. Zoom in to the FT-IR spectra of the [TSC–Ph–phos–OH] conjugates and the [TSC–Ph–phos–TiO_2] conjugates.
Figure S311. UV-vis absorption spectra of pristine TiO_2 NPs, the [TSC–Ph–phos–OH] conjugate and the [TSC–Ph–phos–TiO_2] conjugates.
Figure S312. Photoluminescence spectrum of the [Anthr–TSC–Ph–phos–TiO_2] conjugate in MeOH.
Figure S313. UV-vis absorption spectra of dipyridyl ketone TSCs in MeCN.
Figure S314. UV-vis absorption spectra of 9-anthraaldehyde TSCs in MeCN.
Figure S315. Normalized emission spectra of the 9-antraldehyde TSCs in MeCN ($\lambda_{exc.}$ = 365 nm).
Figure S316. Normalized, concentration-dependent emission spectroscopy of amino[(1*E*)-(anthracen-9-yl)methylideneamino] carbothioamide in MeCN at rt ($\lambda_{exc.}$ = 340 nm).
Figure S317. Normalized, concentration-dependent emission spectroscopy of *tert*-butyl (6-{[(1*E*)-(anthracen-9-yl)methylideneaminocarbamthioyl]amino}hexyl)carbamate in MeCN at rt ($\lambda_{exc.}$ = 340 nm).
Figure S318. Normalized, concentration-dependent emission spectroscopy of *tert*-butyl (4-{[(1*E*)-(anthracen-9-yl)methylideneaminocarbamthioyl]amino}phenyl)carbamate in MeCN at rt ($\lambda_{exc.}$ = 340 nm).

References

Supplementary Figures

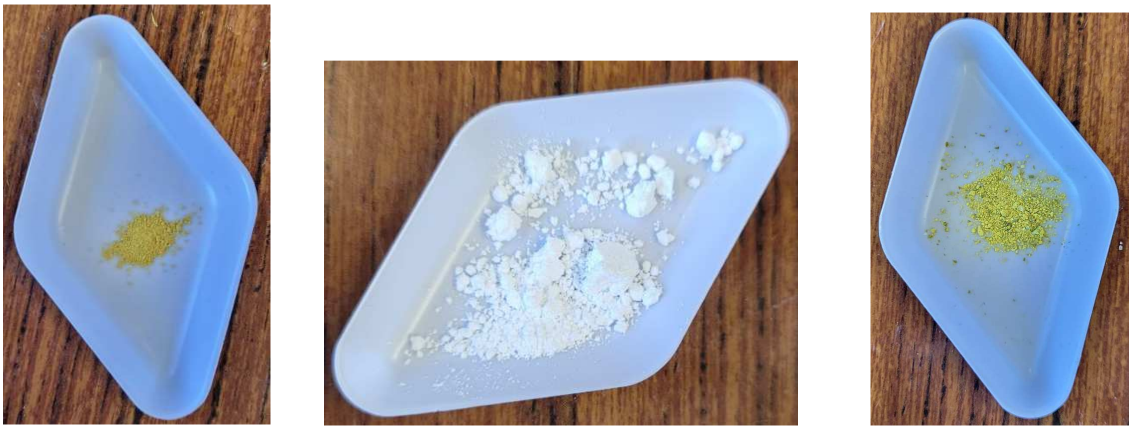

[Anthr-TSC–Ph–phos–TiO_2] TiO_2 (Aeroxid P25) [Dipy–TSC–Ph–Phos–TiO_2]

Figure S308. Photographs of TiO_2 (Aeroxid P25®) and the TSC-phosphonic acid-TiO_2 conjugates.

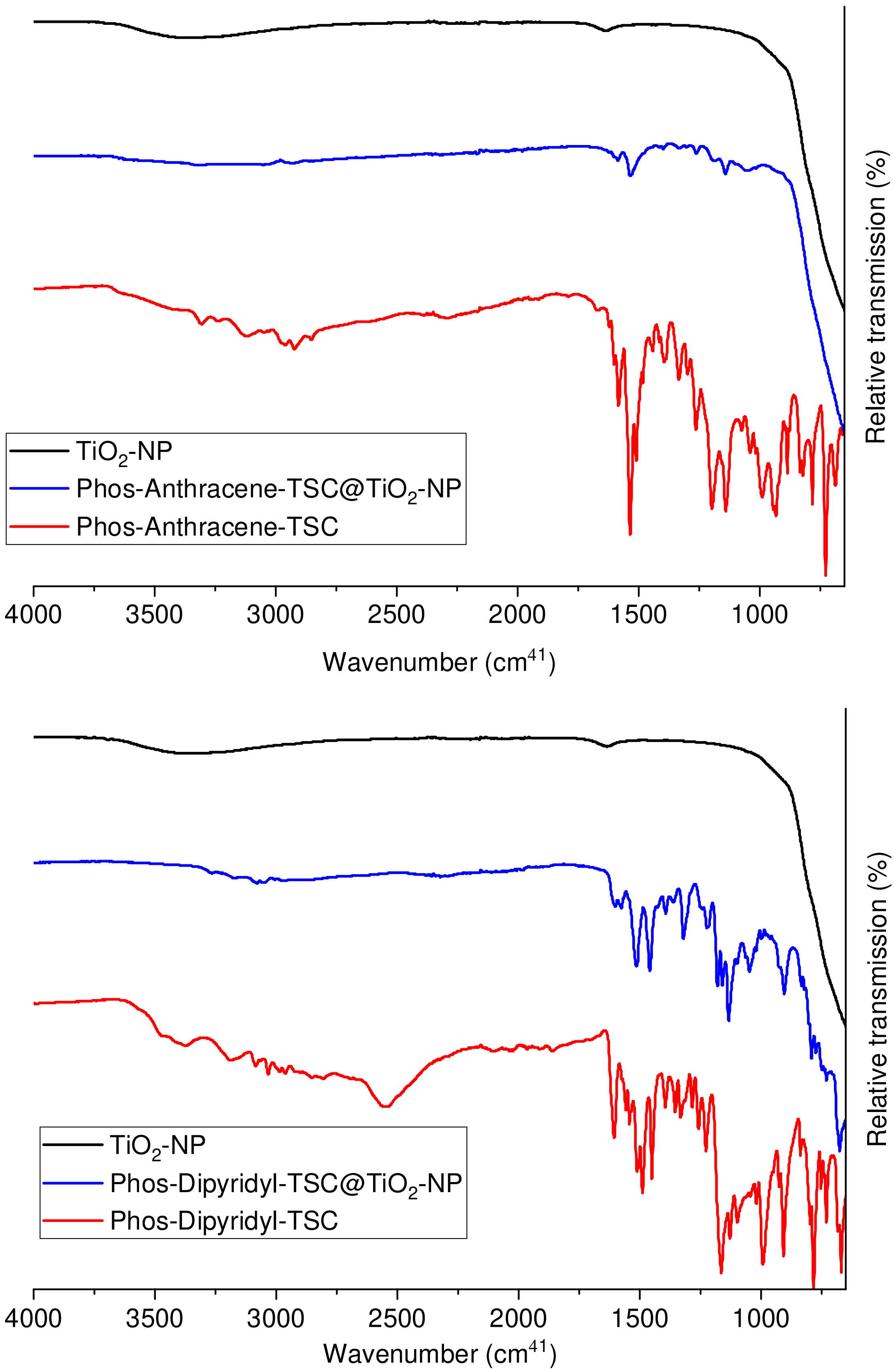

Figure S309. FT-IR spectra of pristine TiO_2 NPs (black), the [TSC–Ph–phos–OH] conjugates (red) and the [TSC–Ph–phos–TiO_2] conjugates (blue) for the anthracene-tagged (top) and the dipyridyl-tagged TSC (bottom). All samples were carefully dried and measure in ATR mode as powders.

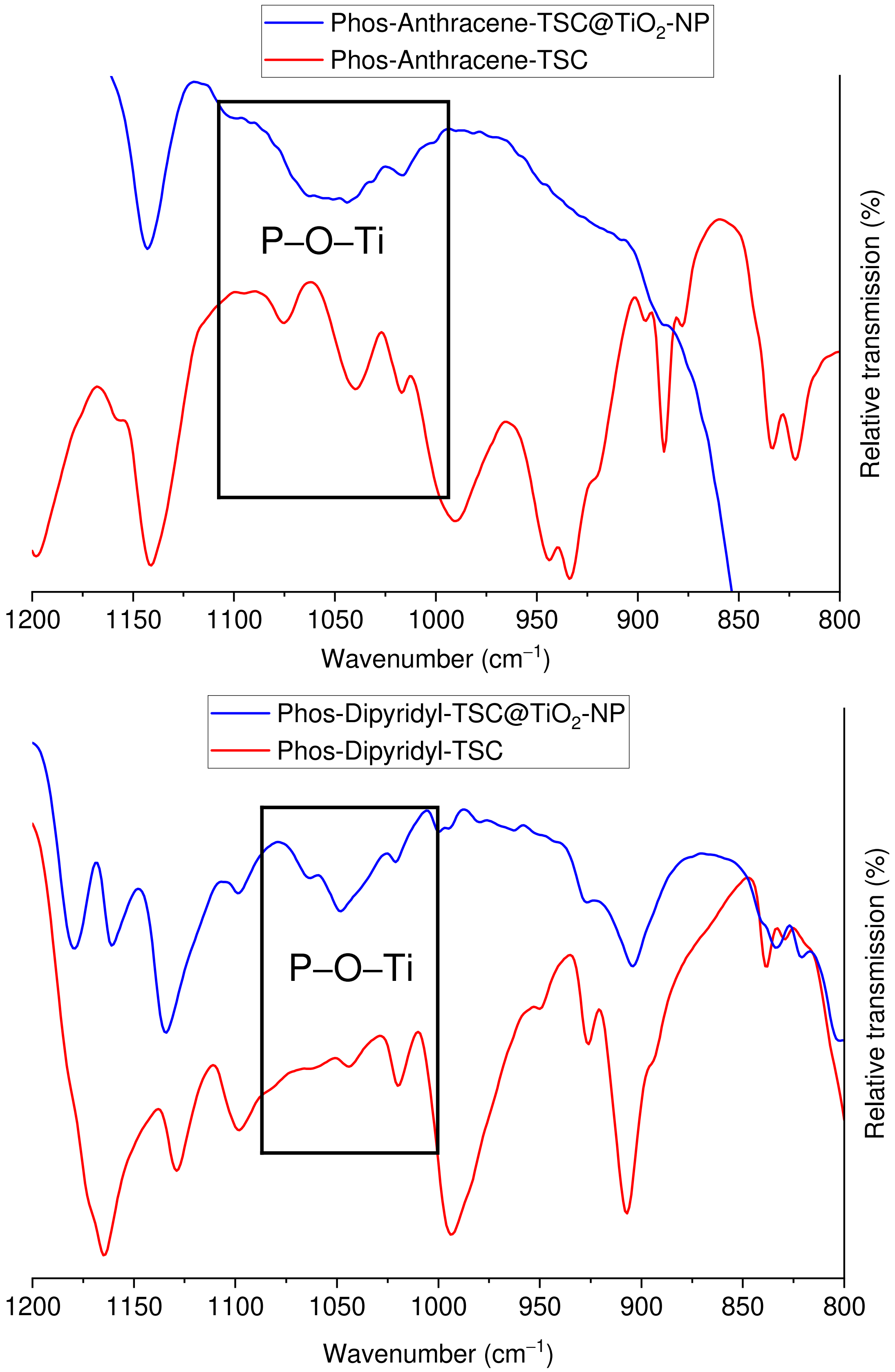

Figure S310. Zoom in to the FT-IR spectra of the [TSC–Ph–phos–OH] conjugates (red) and the [TSC–Ph–phos–TiO_2] conjugates (blue) for the anthracene-tagged (top) and the dipyridyl-tagged TSC (bottom). All samples were carefully dried and measure in ATR mode as powders.

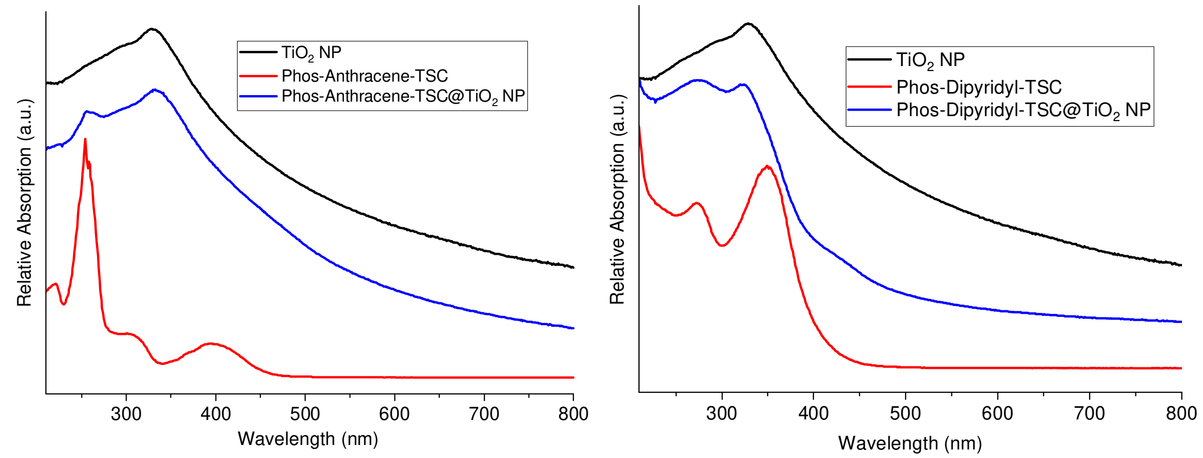

Figure S311. UV-vis absorption spectra of pristine TiO_2 NPs (black), the [TSC–Ph–phos–OH] conjugate (red) and the [TSC–Ph–phos–TiO_2] conjugates (blue).

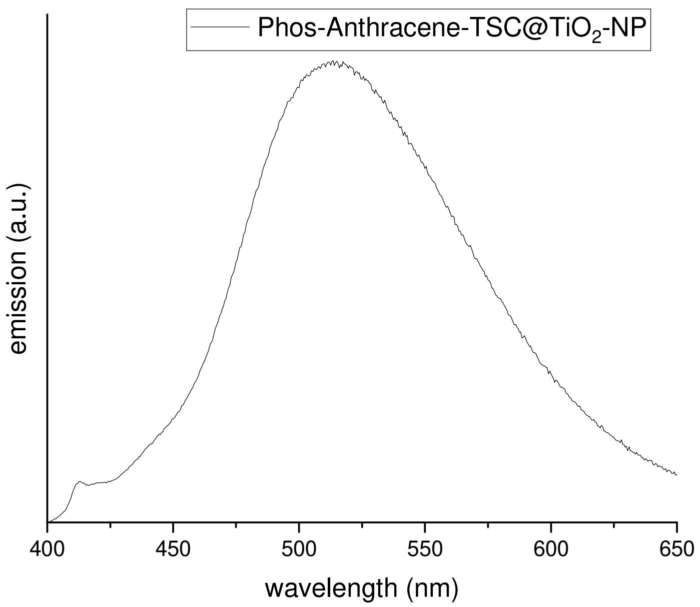

Figure S312. Photoluminescence spectrum of the [Anthr–TSC–Ph–phos–TiO_2] conjugate in MeOH ($\lambda_{exc.}$ = 368 nm).

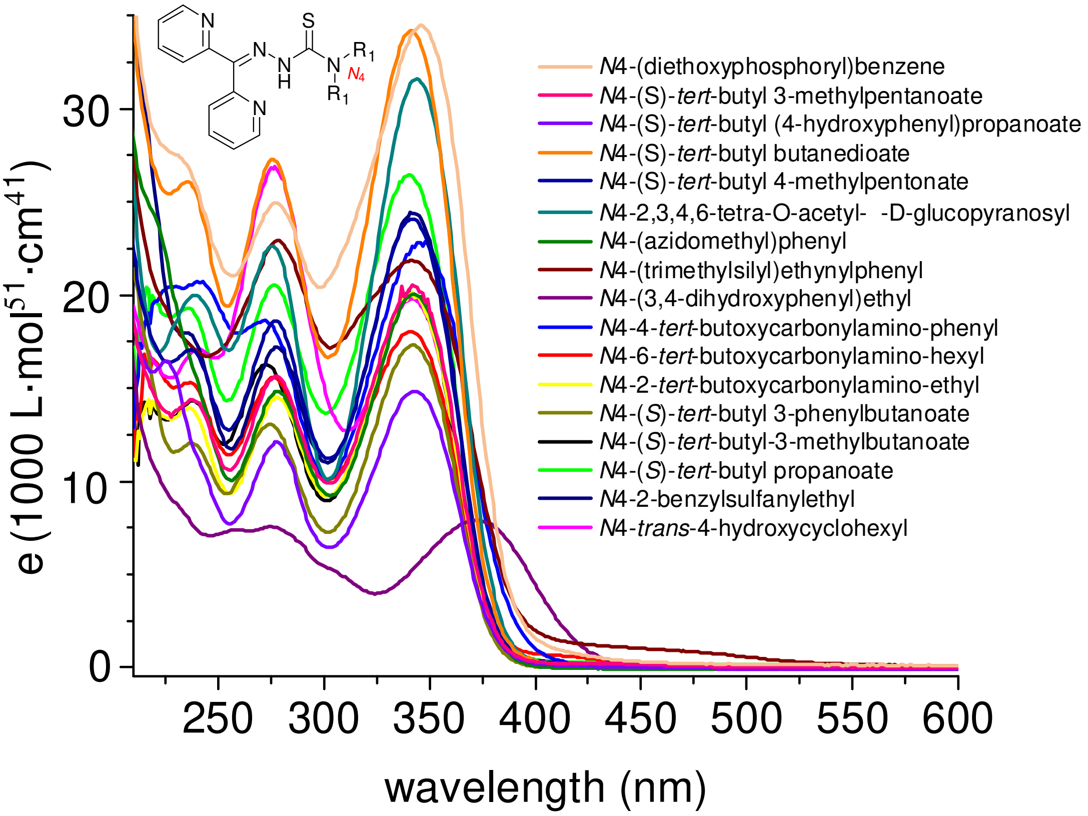

Figure S313. UV-vis absorption spectra of dipyridyl ketone TSCs measured in MeCN.

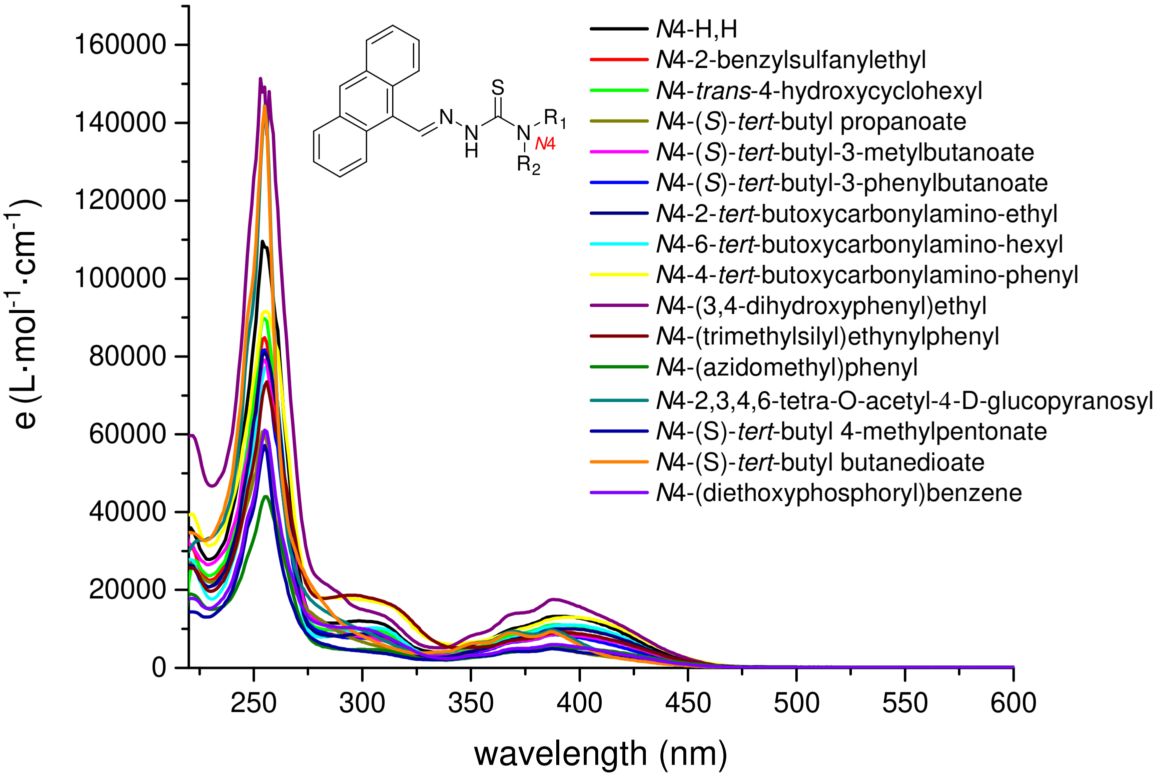

Figure S314. UV-vis absorption spectra of 9-anthraaldehyde TSCs in MeCN.

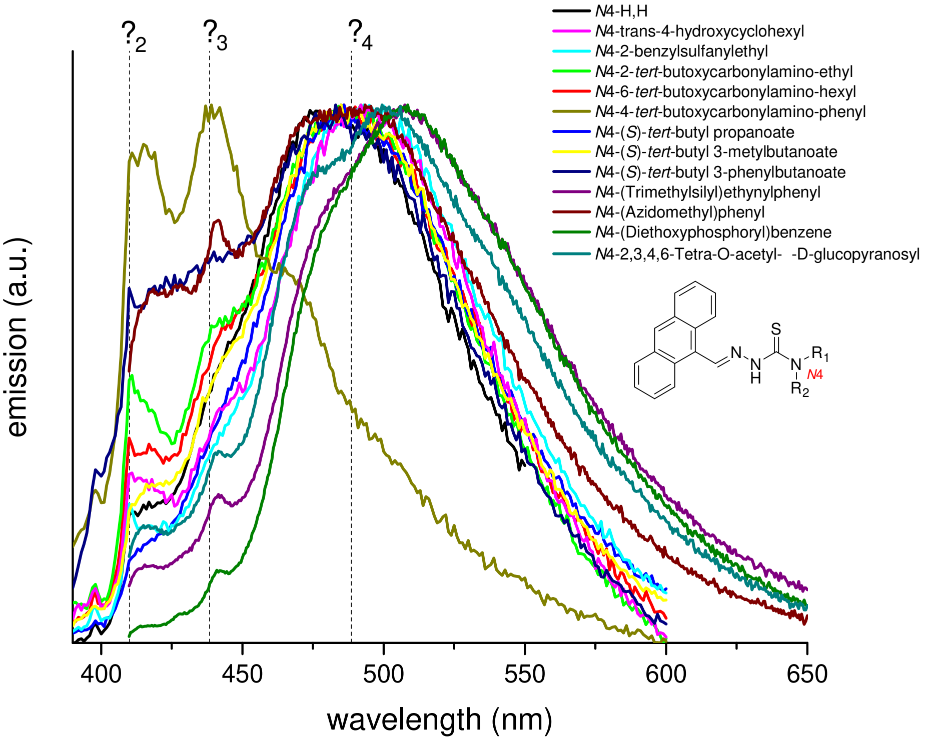

Figure S315. Normalized emission spectra of the 9-antraldehyde TSCs in MeCN ($\lambda_{exc.}$ = 365 nm). Concentrations are arbitrarily chosen. Typical anthracene monomer emission is observed for the *N*4-4-*tert*-butoxycarbonylamino-phenyl derivative, while the typical broad red-shifted emission band recorded for the other derivatives is typical for anthracene excimers [1].

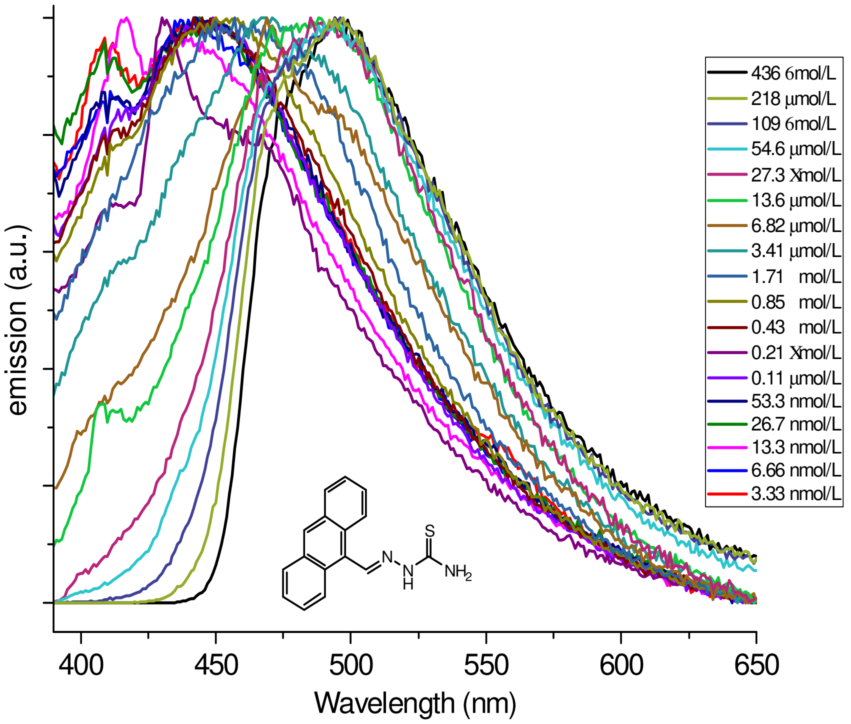

Figure S316. Normalized, concentration-dependent emission spectroscopy of amino[(1*E*)-(anthracen-9-yl)methylideneamino] carbothioamide in MeCN at rt ($\lambda_{exc.}$ = 340 nm). The initial concentration is 436 µmol/L which is diluted to 3.33 nmol/L by halving the concentration in each step.

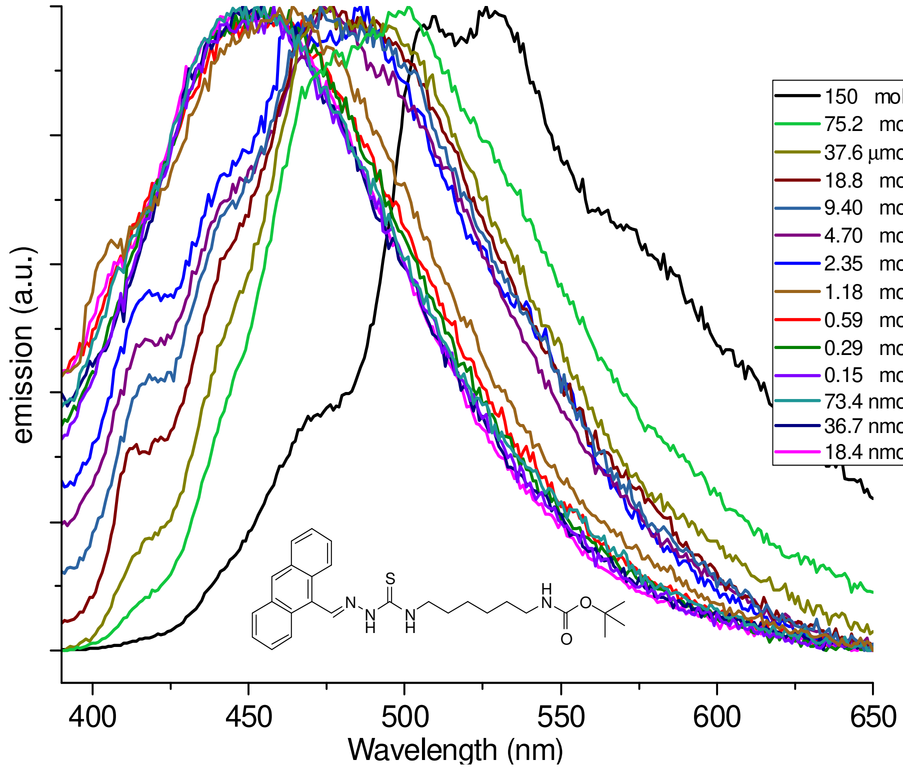

Figure S317. Normalized, concentration-dependent emission spectroscopy of *tert*-butyl (6-{[(1*E*)-(anthracen-9-yl)methylideneaminocarbamthioyl]amino}hexyl)carbamate in MeCN at rt ($\lambda_{exc.}$ = 340 nm). The initial concentration is 150 µmol/L which is diluted to 18.4 nmol/L by halving the concentration in each step.

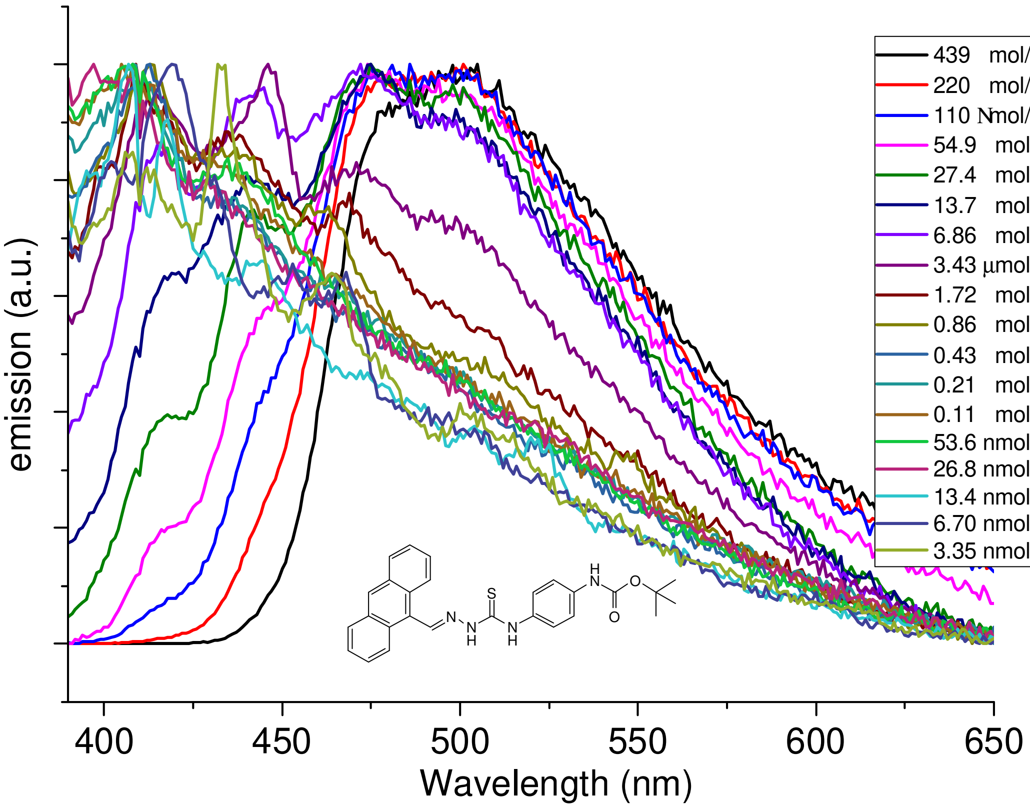

Figure S318. Normalized, concentration-dependent emission spectroscopy of *tert*-butyl (4-{[(1*E*)-(anthracen-9-yl)methylideneaminocarbamthioyl]amino}phenyl)carbamate in MeCN at rt ($\lambda_{exc.}$ = 340 nm). The initial concentration is 439 µmol/L which is diluted to 3.35 nmol/L by halving the concentration in each step.

References

(1) Kastrati, A.; Oswald, F.; Scalabre, A.; Fromm, K.M. Photophysical Properties of Anthracene Derivatives. *Photochem*. **2023**, *3*, 227–273. https://doi.org/10.3390/photochem3020015
